# A multi-platform analysis of human gingival crevicular fluid reveals ferroptosis as a relevant regulated cell death mechanism during the clinical progression of periodontitis

**DOI:** 10.1038/s41368-024-00306-y

**Published:** 2024-05-27

**Authors:** Alfredo Torres, M. Angélica Michea, Ákos Végvári, Marion Arce, Valentina Pérez, Marcela Alcota, Alicia Morales, Rolando Vernal, Mauricio Budini, Roman A. Zubarev, Fermín E. González

**Affiliations:** 1 Laboratory of Experimental Immunology & Cancer, Faculty of Dentistry, University of Chile, Santiago, Chile; 2 Department of Conservative Dentistry, Faculty of Dentistry, University of Chile, Santiago, Chile; 3 Division of Chemistry I, Department of Medical Biochemistry and Biophysics, Karolinska Institutet, Stockholm, Sweden; 4 Periodontal Biology Laboratory, Faculty of Dentistry, Universidad de Chile, Santiago, Chile; 5 Laboratory of Cellular and Molecular Pathology, Institute for Research in Dental Sciences, Faculty of Dentistry, University of Chile, Santiago, Chile

**Keywords:** Periodontitis, High-throughput screening

## Abstract

Ferroptosis is implicated in the pathogenesis of numerous chronic-inflammatory diseases, yet its association with progressive periodontitis remains unexplored. To investigate the involvement and significance of ferroptosis in periodontitis progression, we assessed sixteen periodontitis-diagnosed patients. Disease progression was clinically monitored over twelve weeks via weekly clinical evaluations and gingival crevicular fluid (GCF) collection was performed for further analyses. Clinical metrics, proteomic data, in silico methods, and bioinformatics tools were combined to identify protein profiles linked to periodontitis progression and to explore their potential connection with ferroptosis. Subsequent western blot analyses validated key findings. Finally, a single-cell RNA sequencing (scRNA-seq) dataset (GSE164241) for gingival tissues was analyzed to elucidate cellular dynamics during periodontitis progression. Periodontitis progression was identified as occurring at a faster rate than traditionally thought. GCF samples from progressing and non-progressing periodontal sites showed quantitative and qualitatively distinct proteomic profiles. In addition, specific biological processes and molecular functions during progressive periodontitis were revealed and a set of hub proteins, including SNCA, CA1, HBB, SLC4A1, and ANK1 was strongly associated with the clinical progression status of periodontitis. Moreover, we found specific proteins - drivers or suppressors - associated with ferroptosis (SNCA, FTH1, HSPB1, CD44, and GCLC), revealing the co-occurrence of this specific type of regulated cell death during the clinical progression of periodontitis. Additionally, the integration of quantitative proteomic data with scRNA-seq analysis suggested the susceptibility of fibroblasts to ferroptosis. Our analyses reveal proteins and processes linked to ferroptosis for the first time in periodontal patients, which offer new insights into the molecular mechanisms of progressive periodontal disease. These findings may lead to novel diagnostic and therapeutic strategies.

## Introduction

Periodontitis is a non-communicable chronic multifactorial inflammatory disease linked to dysbiotic biofilms^1^ and marked by the progressive destruction of tooth-supporting tissues. Its defining features are clinical attachment loss (CAL), radiographic alveolar bone loss, periodontal pocketing, and gingival bleeding.^2^

Previously, we identified a specific proteomic profile in gingival crevicular fluid (GCF) associated with the progression of periodontitis in humans.^3^ This profile is characterized by the presence of proteins able to act as damage-associated molecular patterns (DAMPs), suggesting the presence of significant regulated cell death processes associated with the clinical progression of periodontitis. Additionally, the enriched gene ontology terminology associated with periodontitis progression highlighted processes such as “catabolic processes,” “lipid metabolism,” and “binding to hemoglobin and haptoglobin“.^3^ These processes play pivotal roles in the development of ferroptosis, a type of regulated cell death triggered by iron imbalance, depletion of the antioxidant system, and lipid peroxidation.^4^

Recently, the involvement of ferroptosis has been described in the pathogenesis of various chronic inflammatory diseases, cancer, and neurodegenerative disorders, among others.^5–12^ Although some studies have shown that the development of periodontitis is accompanied by iron overload,^13–15^ alterations in antioxidant systems,^16–18^ gingival fibroblast ferroptosis,^19^ and ferroptosis in experimental periodontitis,^20^ the connection between progressive periodontitis and ferroptosis has not been established. Thus, this study aimed to explore the involvement and significance of ferroptosis in the clinical progression of periodontitis.

## Results

### Periodontitis progression can be identified at weekly intervals

**Fig. 1:**
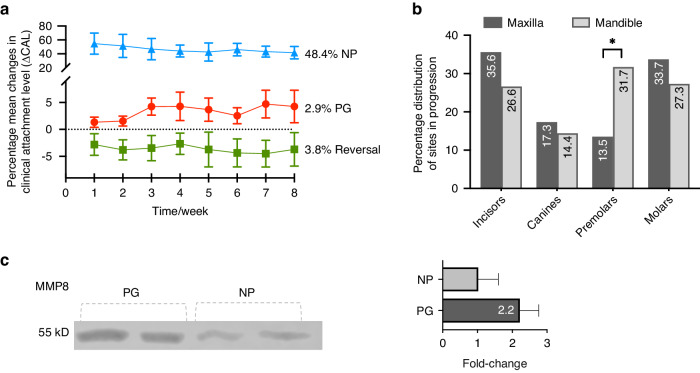
Identification of periodontitis progression and characterization of CAL changes. **a** Linear regression of changes in CAL values for each session; in green, the percentage values of sites that exhibited reversal from the initial evaluation are shown; in red, the percentage values of sites that progressed ≥1 mm weekly are graphed, and in blue, the percentages of periodontal sites that showed no CAL changes during the monitoring sessions are displayed. **b** Distribution by tooth type and arch of periodontal sites that exhibited clinical progression of periodontitis with CAL loss ≥2 mm; * corresponds to *P* < 0.05. **c** Semiquantitative western blot assay of MMP-8 between periodontal sites that remained unchanged in CAL during the monitoring sessions (NP) and those that exhibited CAL loss ≥2 mm (PG)

Demographic and clinically relevant data regarding periodontitis are presented in Table 1 and Supplementary Table 1. The mean number of weekly monitoring sessions for identifying clinical disease progression was 5.6. The full periodontal examination comprised a total of 2 100 periodontal sites that were examined during each patient’s visit to assess the CAL changes (Supplementary Table 1). The linear regression analysis of CAL changes revealed that over an 8-week monitoring period, 48.4% of the sites remained stable (no CAL changes), 2.9% of the sites showed disease progression (increase in CAL ≥2 mm), and 3.8% of the sites exhibited reversal of periodontitis progression when compared to the baseline (Fig. 1a). Significant statistical differences were observed between the upper and lower arch, particularly in the case of the lower premolars (Fig. 1b). When validating the detection of periodontitis progression, western blot analysis revealed that matrix metalloproteinase (MMP) -8 levels were 1.9-fold-change higher in the PG group than in the NP group (Fig. 1c). For additional proteomic analysis, the ‘progression group’ (PG) consisted of GCF samples demonstrating disease progression (increase in CAL ≥ 2 mm) on weekly monitoring sessions, whereas the ‘non-progression group’ (NP) comprised GCF samples from stable sites with no CAL changes over time.

**Table 1:** Demographic characteristics and details of studied groups

Categories	Values	
General characteristics	Results	
Number of participants (sites with GCF sampling)	16 (906)	
Number of follow-up sessions (mean ± SD)	5.6 ± 2.8	
Range of follow-up sessions (min-max)	3–12	
Patients matching increase in CAL ≥ 2 mm and GCF sample in the same periodontal pocket	13	
GCF samples *per* group	18	
Demographic characteristics		
Age (years)	51.2 ± 10	
N° Teeth	21.8 ± 3.2	
PD (mm; mean ± SD)	3.5 ± 0.6	
% sites with PD ≥ 4 mm (mm; mean ± SD)	37.9 ± 17.1	
% sites with PD ≥ 6 mm (mm; mean ± SD)	12.7 ± 10.5	
CAL (mm; mean ± SD)	4.3 ± 1.3	
% sites with CAL ≥ 3 mm (mm; mean ± SD)	72.9 ± 17.1	
% sites with CAL ≥ 5 mm (mm; mean ± SD)	38.4 ± 23.1	
Clinical parameters of sampled pockets	NP group	PG group
Probing depth (mm; mean ± SD)	5.6 ± 2.6	8.4 ± 4.1
CAL (mm; mean ± SD)	6.1 ± 2.4	9.8 ± 2.5
Proteins eluted from GCF samples		
Protein concentration (μg/mL; mean ± SD)	183.0 ± 98.1	240.5 ± 125.8
Range of concentration (μg/mL; min-max)	631.9 – 429.2	71.8 – 608.7
Number of proteins identified	1 543	1 545
Average proteins in samples (mean ± SD)	1 293 ± 71.1	1 320 ± 44.2
Range proteins in samples (min-max)	1 117 – 1 393	1 258 – 1 404
Exclusive proteins *per* group	22	25
MA proteins per group	98	38

*GCF* gingival crevicular fluid, *PD* probing depth, *CAL* clinical attachment loss, *PG* progression, *NP* non-progression, *SD* standard deviation, *MA* more abundant

### A distinctive GCF proteome is found during the progression of periodontitis

**Fig. 2:**
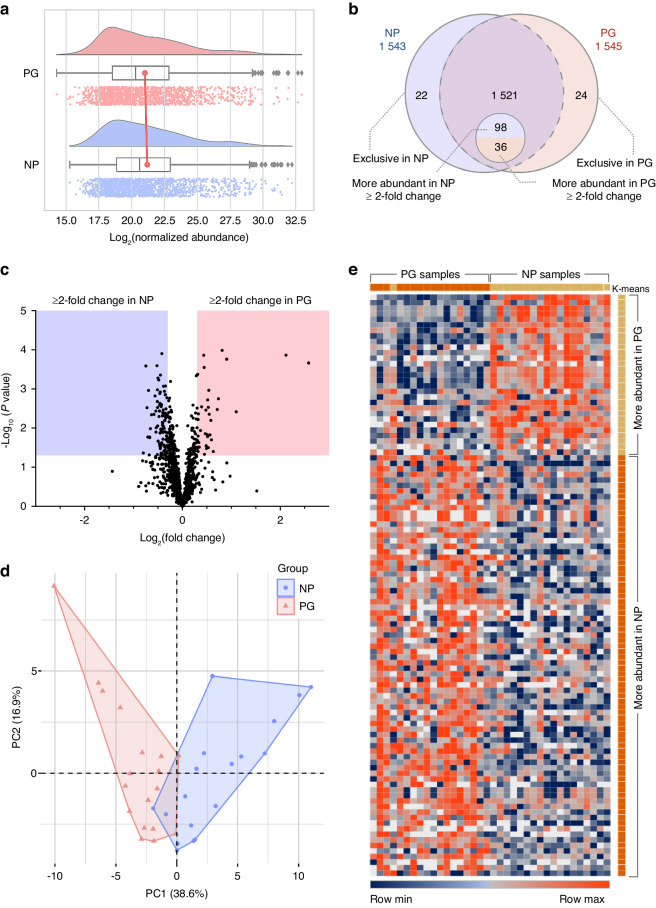
Protein profiles comparison between NP and PG groups. **a** Raincloud plot visualization of normalized relative abundance, including probability density, and summary statistics such as median, mean, and relevant confidence intervals. **b** Venn Diagram: A comprehensive identification of 1 567 proteins is depicted. Specifically, 1 541 proteins were identified in PG, revealing 62 differentially expressed proteins (DEPs) — 24 exclusive and 38 more abundant (fold change ≥2 and *P* < 0.05). In NP, 1 543 proteins were identified, featuring 120 DEPs — 22 exclusive and 98 more abundant (fold change ≥ 2 and *P* < 0.05). **c** Volcano plot: The Log_2_(fold change) indicates the mean expression level for each protein. Each dot represents one single protein. The red and blue areas represent more abundant proteins with significant differences (*P* < 0.05) in the PG- and NP- groups respectively. **d** Factor map of the PCA performed on 36 samples by exclusive and more abundant proteins as variables. Two cluster groups were identified corresponding to NP (cluster 1, blue), and PG (cluster 2, red). **e** Heatmap of gene expression comparisons between NP and PG groups of selected top differentially expressed proteins in each group (FC > 2, *P* < 0.05). The color-coded scale is indicated at the bottom of the chart. Protein abundances were log_2_ transformed and then displayed as colors ranging from orange to blue. Legend: Orange, upregulated; red, downregulated; gray, no modulation; and white, missing values. Both rows and columns were hierarchically clustered using K-means as correlation distance and average linkage using a cut-off at *P* < 0.01

A comprehensive qualitative and quantitative protein analysis was performed to elucidate the dynamic changes in the protein profile during the clinical progression of periodontitis (Fig. 2 and Table 1). The visualization of density plots is shown in Fig. 2a, reflecting the relative abundances of proteins. A total of 1 567 proteins were identified, with 1 545 proteins in the PG group and 1 543 in the NP group. A subset of 24 proteins were exclusively found in the PG group and another 22 proteins exclusively in the NP group (Fig. 2b). According to quantitative analysis, 136 proteins exhibited differences in their relative abundance (fold change ≥2, *P* < 0.05), of which 38 proteins were from the PG group and 98 proteins from the NP group (Fig. 2c and Table 1). PCA revealed a distinct composition for both groups, with an overlap of two samples per group (Fig. 2d). A heatmap was generated using proteins exhibiting the most significant expression changes within each group (fold change ≥ 2, *P* < 0.05). Subsequently, K-means clustering was employed to emphasize the distinctions between these groups (Fig. 2e). These results reveal a distinct protein expression profile for both the PG and NP groups. The complete proteomic profiles derived from the GCF samples are tabulated in Supplementary Table 2.

### Enrichment analysis reveals distinctive biological processes and molecular functions during progressive periodontitis

**Fig. 3:**
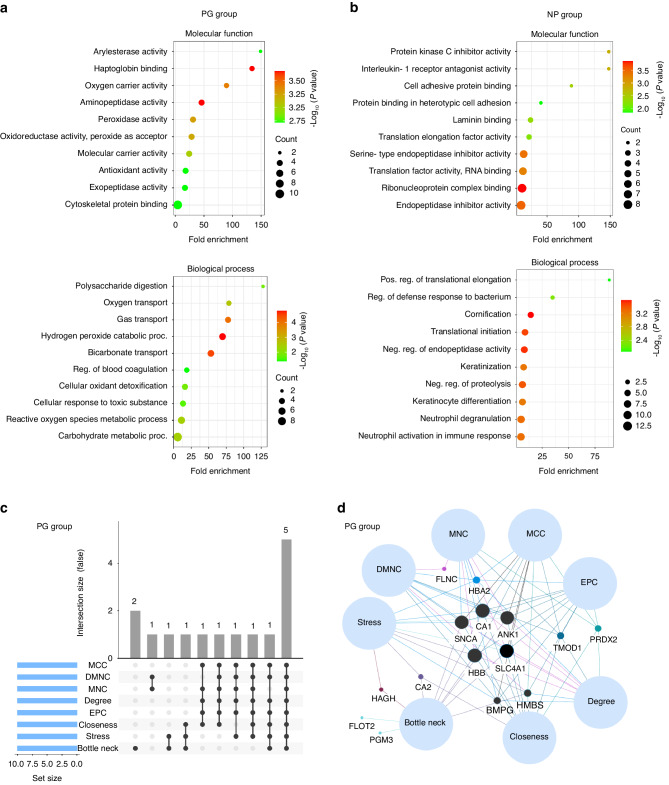
Enrichment analysis of exclusive and more abundant protein sets. **a**, **b** Fold enrichment of functional annotations of gene ontology terms based on molecular function and biological process in PG and NP, respectively. The dot size represents the count of genes associated with each enriched term; a *P* < 0.05 was considered statistically significant. **c** UpSet diagram illustrating the identification of hub genes using different algorithms in PG. **d** Venn networks displaying intersecting hub proteins in PG and NP. Note that five intersected hub proteins were found in PG: CA1, SNCA, HBB, SLC4A1, and ANK1

To elucidate the biological functions and molecular events that characterize the periodontitis progression, bioinformatic enrichment analyses based on the Gene Ontology (GO) classification were conducted. In the case of the PG group, the following molecular functions (MF) emerged among the top 10 most enriched-terms: “Arylesterase activity”, “haptoglobin binding”, “peroxidase activity”, “oxidoreductase activity, peroxide as acceptor”, “amino- and exo-peptidases activity”, and “cytoskeletal protein binding”. Regarding the biological processes (BP) in the GO analysis, several noteworthy terms emerged in the PG group. These include “cellular oxidant detoxification and response to toxic substances”, “reactive oxygen species (ROS) and hydrogen peroxide catabolic process”, “bicarbonate transport”, “oxygen and gas transport”, and “polysaccharide digestion and carbohydrate metabolic process” (Fig. 3a and Supplementary Fig. 1). In contrast, the NP group exhibited a distinct set of GO-BP and GO-MF related to regulatory processes compatible with stable periodontal pockets (Fig. 3b and Supplementary Fig. 1). This functional enrichment analysis highlighted the mechanism underlying the clinical progression of periodontitis and emphasized the regulatory processes of stable periodontal pockets.

### Alpha-synuclein, carbonic anhydrase 1, hemoglobin subunit beta, band 3 anion transport protein, and ankyrin-1 are hub proteins during periodontitis progression

**Fig. 4:**
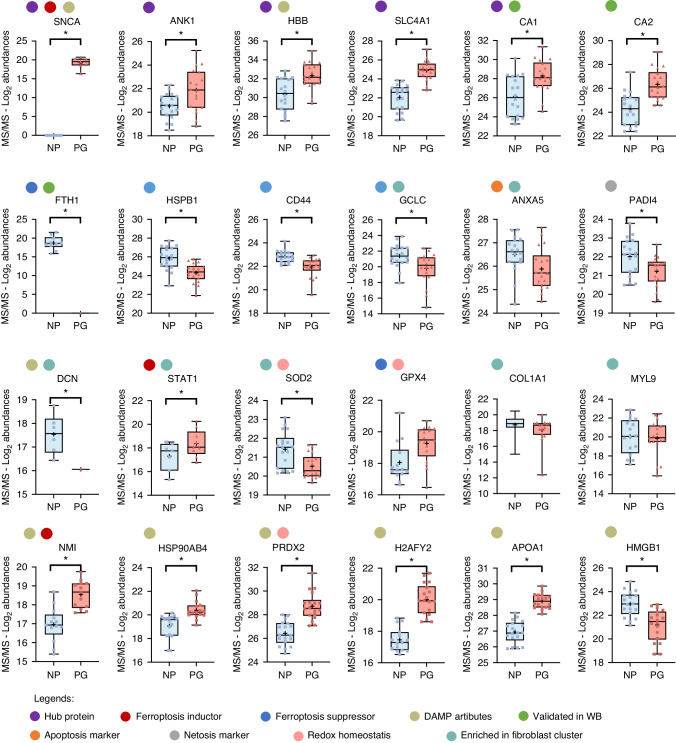
Relative protein abundance plots. Log-transformed (Log_2_) of the relative protein abundances of selected proteins from NP and PG groups. These proteins were identified with ≥2 unique peptides within each protein for identification (double-hit proteins), reducing false-positive hits.^114^ The colored circles overlaid on the plots represent manual annotations detailing the functional classification of proteins. DAMP Damage-associated molecular pattern, WB western blot. *P < 0.05

To gain deeper insights into the functions and mechanisms of periodontitis progression, the hub proteins were identified. The exclusive and most abundant genes of PG and NP groups were imported into the STRING database for protein–protein interaction detection (Supplementary Figs. 2 and 3), and then, networks were visualized in Cytoscape. With the CytoHubba plugin, the top 10 hub proteins were identified in both groups using seven algorithms (Fig. 3 and Supplementary Figs. 4 and 5). In PG, five hub proteins were common to all algorithms: alpha-synuclein (SNCA), carbonic anhydrase 1 (CA1), hemoglobin subunit beta (HBB), band 3 anion transport protein (SLC4A1), and ankyrin-1 (ANK1) (Figs. 3c and 3d). Additionally, individual box plots, constructed with the relative abundance profiles, confirmed the quantitative differences of these proteins between PG and NP groups (Fig. 4). Consistently, these hub proteins reflected the most enriched GO terms of the total set of exclusive and most abundant proteins previously identified in the PG group. Functional annotations of hub genes are listed in Table 2.

**Table 2:** Hub proteins in the PG group

Gene name	Accession	Protein name	Functional annotations (Uniprot)
SNCA	P37840	Alpha-synuclein	Mechanistically, acts by increasing local Ca^2+^ release from microdomains. Participates as a monomer in vesicle exocytosis. Acts also as a molecular chaperone in its multimeric membrane-bound state. Ligand: metal-binding.
CA1	P00915	Carbonic anhydrase 1	Catalyzes the reversible hydration of carbon dioxide. Up-regulated by JAK-STAT signaling following Interleukin-12 stimulation. Ligand: metal-binding and Zinc.
HBB	P68871	Hemoglobin subunit beta	Involved in oxygen transport, Heme signaling, and scavenging of heme from cytoplasm. Involved in cytoprotection by HMOX1. Ligand: Heme, Iron, Metal-binding, Pyruvate.
SLC4A1	P02730	Band 3 anion transport protein	Structural protein and anion exchanger and transporter. Mediates chloride-bicarbonate exchange. Involved in the regulation of intracellular pH.
ANK1	P16157	Ankyrin-1	Attaches integral membrane proteins to cytoskeletal elements. Involved in cytoskeletal organization, signal transduction, and exocytosis.

### Ferroptosis co-occurs with clinical progression of periodontitis

**Fig. 5:**
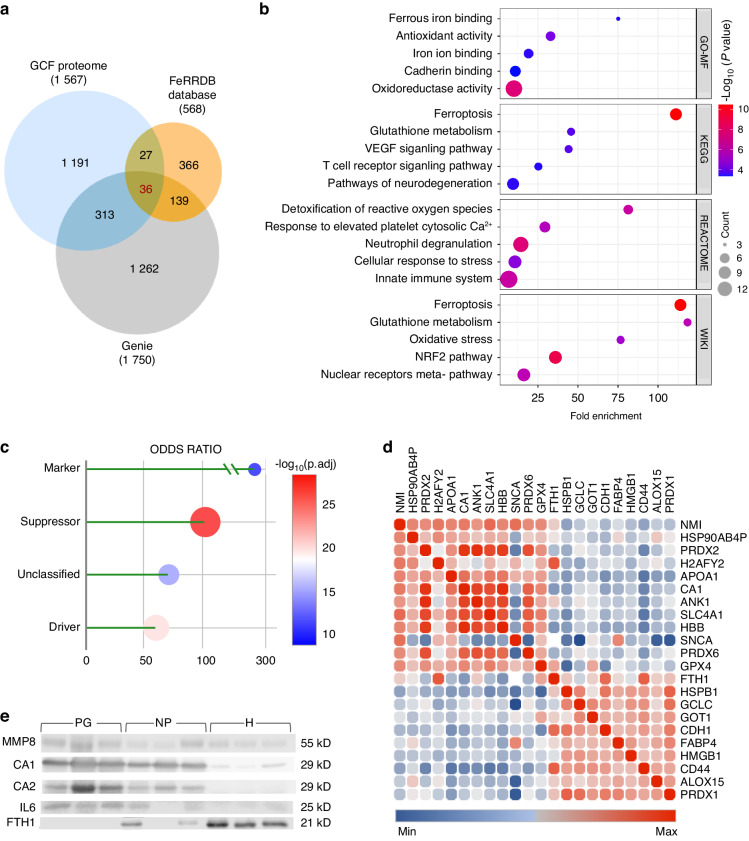
Ferroptosis co-occurs with the clinical progression of periodontitis. **a** Venn diagram illustrating the overlap of proteins among different groups: Gingival Crevicular Fluid (GCF) proteome, curated data set from Génie, and FerrDb gene set. **b** Fold enrichment of functionally enriched terms for the intersected proteins, based on Gene Ontology - Molecular Function (GO-MF), Kyoto Encyclopedia of Genes and Genomes (KEGG database), Reactome, and Wiki database. **c** Over-representation analysis of the intersected proteins according to the FerrDb V2 classification system. **d** Correlation matrix displaying selected proteins that function as DAMPs, Hub genes, and Ferroptosis-related proteins. **e** Western blot analysis for protein validation in GCF from NP (non-progression group), PG (progression group), and healthy (H) controls. Eluted proteins (40 µL) were separated using 10% SDS-PAGE. Duplicated experiments were conducted, and representative results were presented for each experiment. GCF gingival crevicular fluid, NP non-progression group, PG progression group, H healthy group

To explore regulated cell death-related activity in periodontitis progression, data mining and an enrichment analysis were performed. An in silico assay identified 1 750 coding genes linked to periodontitis progression from the GENIE database. Using Cluepedia in the Cytoscape environment, enriched terms related to apoptosis, necroptosis, and ferroptosis were identified (Supplementary Fig. 6). Additionally, the FerrDb database was consulted, which provided 568 genes associated with ferroptosis. From this analysis, 36 GCF proteins were found to overlap with the predictive biomarkers from Génie and the ferroptosis database (Fig. 5a).

A comprehensive pathway analysis utilizing the KEGG, GO, Reactome, and WIKI databases was conducted. The results are depicted in Fig. 5b, showing the top five most enriched terms for each category. According to GO-MF, the most enriched terms included “ferrous and iron ion binding”, “antioxidant activity”, “cadherin binding”, and “oxidoreductase activity”. In the KEGG database, the most enriched terms encompassed “ferroptosis”, “glutathione metabolism”, “VEGF and T cell receptor pathways”, and “pathways of neurodegeneration”. Within the Reactome database, the most enriched terms were “detoxification of ROS”, “response to elevated cytosolic Ca^+^2”, “neutrophil degranulation”, “cellular response to stress”, and “innate immune response”. Finally, in the WIKI database, the most enriched terms included “ferroptosis”, “glutathione metabolism”, “oxidative stress”, “nuclear factor erythroid 2 (NRF2) pathway,” and nuclear receptor meta-pathways”.

Based on this information, an enrichment analysis was conducted in the FerrDb database, identifying proteins classified as markers, drivers, and suppressors of ferroptosis, with the latter exhibiting a more significant over-representation (Fig. 5c). Figure 4 shows individual box plots displaying the relative abundance profiles of the following selected proteins: SNCA (classified as a ferroptosis driver), ferritin heavy chain (FTH1), heat shock protein beta-1 (HSPB1), CD44 antigen (CD44), and glutamate-cysteine ligase catalytic subunit (GCLC) (all classified as ferroptosis suppressors). Additionally, plot graphs were created for annexin A5 (ANXA5) and Protein-arginine deiminase type-4 (PADI4), considered as markers for apoptosis and netosis, respectively.^21–24^

A manual search was performed for DAMPs molecules in the GCF proteome to explore cell death signals. Interestingly, significant differences were found in the abundance of apolipoprotein A-I (APOA1), core histone macro-H2A.2 (H2AFY2), peroxiredoxin-2 (PRDX2), heat shock protein 90 alpha family class B member 4 (HSP90AB4), N-myc-interactor (NMI) (higher in PG), and high mobility group protein B1 (HMGB1) (higher in NP) (Fig. 4). For detailed DAMP functions and functional annotations, refer to Supplementary Table 3. A correlation matrix was created among hub protein abundances, revealing strong associations among ferroptosis-related proteins, DAMP molecules, and hub proteins (Fig. 5d). To explore the potential of CA1, CA2, HSPB1, and SLC4A1 as biomarkers, Receiver Operating Characteristic (ROC) curves were generated for these proteins (Supplementary Fig. 7a). Interestingly, they showed their relevance as potential biomarkers for the progression of periodontitis.

### Levels of CA1, CA2, MMP-8, and IL-6 are increased, while FTH1 is not detected during the progression of periodontitis

To validate the findings from proteomic data and gain deeper insights into the differences with a periodontally healthy condition (H), western blot analyses were conducted comparing H, NP, and PG samples (Fig. 5e). The focus was on the proteins CA1, CA2, MMP-8, IL-6, and ferritin heavy chain 1 (FTH1), the primary intracellular iron storage and a ferroptosis suppressor. MMP-8 and IL-6 are widely utilized for studying GCF across various clinical periodontal conditions.^25–30^

In the PG group, the protein content of CA1, CA2, IL-6, and MMP-8 exhibited the highest levels, followed by NP and H samples. The protein bands detected for CA1 and CA2 showed similar expression levels to those observed in the proteomic analysis (Figs. 4 and 5e and Supplementary Fig. 7b).

Notably, CA2 was not found in H samples, while FTH1 was absent in the PG group and showed the highest expression in the H group (Fig. 5e and Supplementary Fig. 7b). Detailed fold change comparisons of semi-quantitative analysis are provided in Supplementary Fig. 7b.

Importantly, IL-6 and MMP-8 results validated the biological separation of groups associated with the clinical progression of periodontitis, supporting proteomic findings and offering additional protein-level evidence. These results not only validated the differences between healthy and pathological conditions but also correlated with periodontitis progression, strongly aligned with the trends observed in our proteomic data and affirmed the correlation between CAL increases and the proteomic changes identified during periodontitis progression. Taken together, these results underscore the co-occurrence of ferroptosis during the clinical progression of periodontitis.

### Integration of quantitative proteomic data with single-cell RNA sequencing (scRNA-seq) analysis reveals insights into cellular dynamics in periodontitis progression and the susceptibility of fibroblasts to ferroptosis

**Fig. 6:**
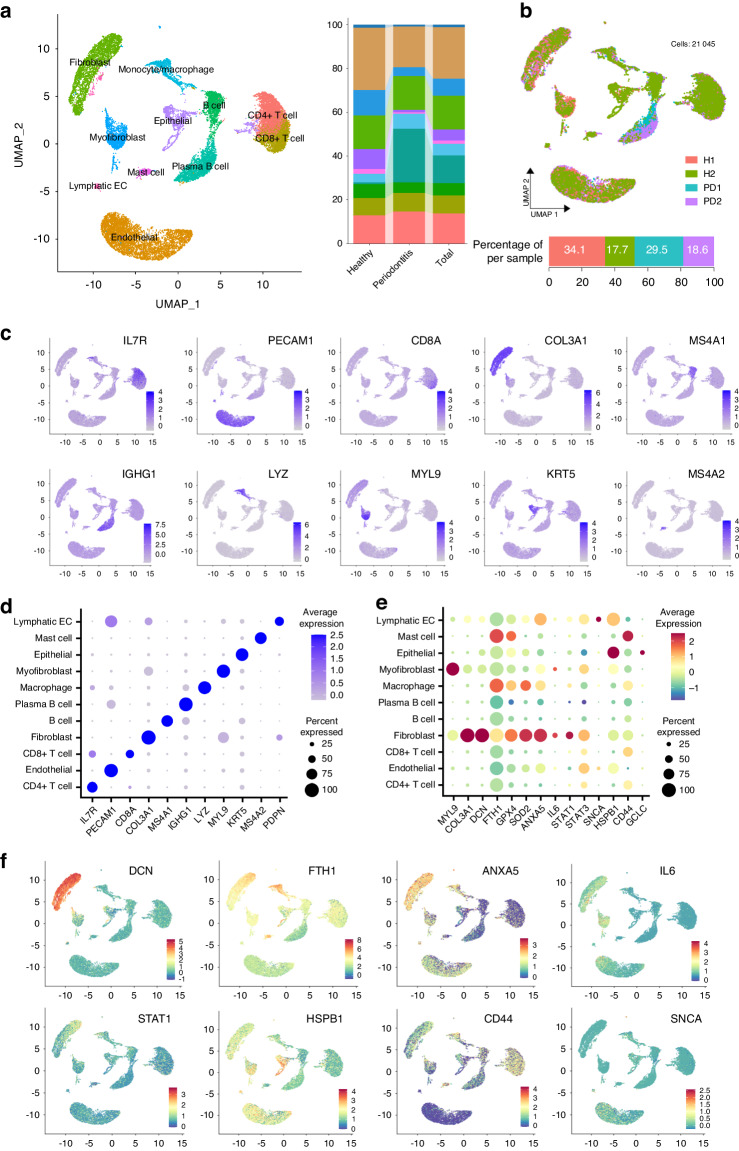
Analysis of selected proteins on an external single-cell RNA-sequencing (scRNA-seq) of human marginal gingival tissue. **a** Twenty-one cell clusters of human marginal gingival tissue were identified based on specific genes, resulting in eleven labeled clusters derived from the pooled data of the whole-tissue scRNA-seq dataset (GES164241). These clusters were visualized using UMAP. Stacked bar plot displaying the percentage distribution of cell clusters for Healthy, Periodontitis, and the total dataset. Each color corresponds to a different cell cluster. Additional pre-processing and quality control analyses are presented in Supplementary Fig. 6a–d. **b** Integrated UMAP projection of cell clusters split by patient, with each color representing a different patient (H1, H2, PD1, and PD2). Filled bars illustrate the percentage of cells from each patient. **c** Feature plot showing differentially expressed marker genes for each cluster. The average expression level is depicted in a blue-gray scale, where gray signifies the minimal expression value and deep blue indicates the highest expression levels. **d** The dot plot illustrates the expression levels of the selected markers across eleven clusters. The average expression level is depicted in a blue-gray scale, where gray signifies the minimal expression value and deep blue indicates the highest expression levels. The size of each dot corresponds to the percentage of cells expressing the gene (feature). **e** The dot plot illustrates the expression levels of the selected genes across eleven clusters. The average expression level is depicted using a color gradient scale. The size of each dot corresponds to the percentage of cells expressing the gene (feature). **f** Feature plot showing selected DEPs from the GCF proteome for each cluster. The average expression level is depicted using a color gradient scale

To further explore the differential susceptibility of specific periodontal cell types to ferroptosis, the scRNA-seq dataset (GSE171213) from the GEO database underwent analysis, encompassing a total of 21 045 cells, including 10 139 cells from two healthy control individuals and 10 906 cells from two patients with periodontitis (Fig. 6a and 6b and Supplementary Table 4). The cell filtering and quality control phases are illustrated in Supplementary Figs. 8 and 9. To achieve a robust compression of the dataset, linear dimensional reduction was applied to scale the data, and 15 principal components (PCs) were selected for uniform manifold approximation and projection (UMAP) analysis (Supplementary Fig. 8b).

The UMAP analysis revealed unbiased clustering of cells, identifying 21 clusters (Supplementary Fig. 8c and Supplementary Table 5). Marker genes were calculated, and each cluster was annotated based on these markers, resulting in a total of 11 different cell types (Fig. 6a and Supplementary Table 4). These clusters were specifically categorized as follows: 1) CD4+ T cell cluster (13.8%); 2) CD8+ T cell cluster (8.2%); 3) B cell cluster (5.6%); 4) plasma cell cluster (12.6%); 5) monocyte/Macrophage cluster (5.%); 6) fibroblast cluster (15.4%); 7) myofibroblast cluster (7.8%); 8) endothelial cell cluster (23.4%); 9) epithelial cell cluster (5.1%); 10) mast cell cluster (1.4%); and 11) lymphatic endothelial cell cluster (1.1%). Notably, it can be observed that the proportion of plasma B cells cluster was significantly higher in the periodontitis group than in the healthy control group, revealing differences in cell fractions, ranging from 0.8% in healthy patients to 24.5% in patients with periodontitis (Fig. 6a). Additional UMAP analyses are shown in Supplementary Fig. 8.

To explore which periodontal cell types are potentially responsible for influencing changes in GCF and can undergo ferroptosis during periodontitis progression, we assessed the expression of a set of selected and differentially expressed proteins (DEPs) in the GCF proteome within different cell clusters by means of visual maps using the Featureplot and Dotplot functions in the Seurat package. Representative images are shown in Fig. 6c–f. It can be seen that along with myosin light chain 9 (MYL9) and collagen type III alpha 1 chain (COL3A1) – fibroblast markers – FTH1, IL-6, ANXA5, decorin (DCN), superoxide dismutase 2 (SOD2), and the signal transducer and activator of transcription 1 and 3 (STAT1 and STAT3, respectively) are significantly distributed in the fibroblast cluster in this dataset (Fig. 6c–f). In addition, FTH1 showed the highest expression levels in the mast cell cluster, followed by the macrophage and fibroblast clusters (Fig. 6e, f). Interestingly, the relative abundance plots of selected DEPs from the GCF shown in Fig. 4 reflect that DCN and FTH1 were expressed exclusively in the NP group (DCN was only found in 1 out of 18 samples from the PG group). The proteins ANXA5, GCLC, CD44, and HSPB1 showed higher relative abundance in the NP group, and STAT1 showed higher relative abundance in the NP group. Finally, MYL9 – a marker for fibroblast and myofibroblast, was found in both groups. This analysis reflected the complexity of GCF as an inflammatory exudate, contingent upon the cellular origin of proteins. Deep interpretation analysis hints that fibroblasts, amongst the non-immune cells, play a pivotal role in both the qualitative and quantitative alterations observed in the proteome of GCF and the expression of IL-6, FTH1, and DCN, at RNA and protein levels, indicating a critical susceptibility of fibroblasts to ferroptosis in progressive sites (PG group) (Figs. 4–6).

## Discussion

Here, we demonstrate the achievability of identifying periodontitis progression within weekly intervals, providing evidence that it occurs within shorter timeframes than conventionally considered.^31–33^ Our study showed that only 2.9% of sites exhibited disease progression at eight weeks, in concordance with Teles^31^ and colleagues, who observed CAL loss ≥2 mm in 2.3% of sampled periodontal pockets after a two-month period,^33^ supporting the findings that that periodontitis progression affects a very small percentage of examined sites.^34,35^ Additionally, the observed coexistence of sites in progression, stability, and regression aligns with other studies reporting that specific sites progress while others keep stable or even improve in a cyclic and asynchronous manner.^33,36^ These results emphasize the dynamic and complex nature of periodontal disease progression in humans.

GCF is constituted by a diverse mixture of components originating from serum, host inflammatory cells, structural cells of the periodontium, and oral bacteria.^37,38^ The GCF is widely accepted as a valuable medium for the assessment of periodontal diseases due to its proximity to periodontal tissues.^37,38^ However, given the fact that GCF has the potential to reflect distal changes derived from serum components, it was essential to pair NP and PG sites based on tooth type and sampling session number (Supplementary Table 1). This pairing ensures that patients’ systemic changes do not influence the differences between PG and NP groups, enabling a more in-depth understanding of the periodontal microenvironment and periodontitis progression mechanisms.

In this study, a label-free, bottom-up MS-based proteomics assay was conducted. Due to the biological complexity of GCF, a single unique peptide was used for protein identification, similar to other research efforts,^3,39,40^ and, to our knowledge, a record-high number of 1567 proteins were identified in human GCF. Such an approach allows us to achieve higher sensitivity, which was particularly valuable in this study.

The fact that samples came not only from the same disease condition and patients but also differed only in CAL stability (NP group) or disease progression (PG group) enabled the identification of a specific group of DEPs associated with the progression of periodontitis. The bioinformatic analysis of functional annotations for DEPs revealed significant molecular functions and biological processes in each group, underlying some of the mechanisms of periodontitis progression. The molecular functions identified include “arylesterase activity”, “haptoglobin binding” and “peroxidase and oxidoreductase activity” (Fig. 3a). In periodontal infected states, higher levels of lipid peroxidation were found.^41–44^ Lipid peroxidation is a pivotal process in ferroptosis involving oxidative damage to lipids in cell membranes, resulting ultimately in cell death.^4,45^ The enriched GO term “arylesterase activity” found in this study suggests a compensatory mechanism aimed to protect against lipoprotein oxidation and the hydrolysis of aromatic carboxylic acid esters.^46^ This finding underscores that lipid peroxidation is indeed a feature of periodontitis progression. On the other hand, the “haptoglobin binding” process plays a crucial role in the extracellular detoxification and clearance of hemoglobin. Evidence exists for the involvement of hemoproteins in the development of periodontitis and some hemoglobin sub-units have been proposed as potential biomarkers for periodontitis.^47^ Furthermore, hemoproteins have been described as DAMP molecules^48,49^ and an up-regulation of iron-containing compounds has been found in periodontitis samples.^13–15,50^ In this study, HBB and HBA2 showed significantly higher relative abundance in PG than NP (>3-fold change, *P* < 0.05). This indicates a potential role of these molecules and iron metabolism in periodontitis progression. “Peroxidase and oxidoreductase activities” are related to the protection against oxidative stress and peroxide-induced cell damage. During infectious diseases, a substantial increase in peroxide production occurs,^51^ and alterations in the antioxidant systems are found in periodontitis.^16–18,43^ The antioxidant system comprises numerous enzymatic and non-enzymatic components to counteract oxidative stress and ROS.^52^ Although oxidative stress and ROS exposure are typically harmful to cells, inducing an excessive production of ROS can be advantageous for the elimination of cancer cells,^53^ reflecting the double-edged nature of cell death which calls for further investigation in periodontitis.

In this study, SNCA, CA1, SLC4A1, ANK1, and HBB were identified as hub proteins associated with periodontitis progression. The aggregation of SNCA is associated with various pathological processes, including mitochondrial dysfunction, increased oxidative stress, and neuroinflammation.^54–57^ SNCA has been also recognized as a DAMP molecule leading to the upregulation of TNFα, IL-6, and CXCL1 expression.^54,58,59^ ANK1 has also been implicated in chronic neurodegenerative diseases,^60,61^ involved in the interferon-γ signaling pathways.^62^ Elevated levels of CA1 have been associated with the exacerbation of joint inflammation, tissue destruction, improper calcification, and bone resorption.^63,64^ Notably, high levels of CA1 have been identified in GCF of periodontal pockets.^3,39,65^ In this study, CA1 was found to be > 3-fold more abundant in the PG group than in the NP group (*P* < 0.05). This finding aligns with the distinctive resorptive and catabolic events of clinical tissue breakdown during periodontitis progression.^2^ As hub proteins have also been linked to the progression of other chronic inflammatory diseases, further in-depth analysis and future investigations are desired.

The co-occurrence of ferroptosis with periodontitis progression is a novel finding. The multi-dimensional analysis combining clinical data, proteomics, bioinformatics, and specialized databases allowed us to identify a subset of 36 GCF proteins associated with periodontitis progression, with their enriched GO terms indicating activities related to oxidative stress and iron imbalance, both crucial in ferroptosis development. KEGG and WIKI databases underscored ferroptosis as the most significant process during periodontitis progression. Additionally, enrichment analysis conducted in FerrDb identified ferroptosis-related proteins, with suppressors being significantly over-represented and with the relative abundance of proteins reflecting specific potential pathways. For instance, FTH1 plays an important role in the maintenance of the cellular iron balance and storage,^66^ which was surprisingly absent in the PG group, potentially hinting at iron imbalance. Moreover, SNCA, which functions as a ferroptosis driver, was exclusively identified in the PG group. HSPB1 serves as a ferroptosis suppressor by reducing the iron-mediated production of lipid reactive oxygen species.^67^ Additionally, it regulates cytoskeleton-mediated iron uptake through its interaction with TFR1.^68^ CD44 is considered a ferroptosis suppressor as it promotes the stability of the SLC7A11 protein. This protein plays a predominant role in conferring ferroptosis resistance by facilitating cystine uptake, which is essential for glutathione (GSH) formation.^69^ GCLC plays a non-canonical role in protecting against ferroptosis by preserving glutamate homeostasis in conditions of cystine starvation, independent of glutathione.^70^ In the present study, the levels of HSPB1, CD44, and GCLC were significantly higher in the NP group. These findings provide insights into the molecular mechanisms underlying ferroptosis during periodontitis progression.

The currently available diagnostic tools for periodontal diseases primarily rely on clinical and radiographic findings, reflecting past disease events only.^71^ In this context, various research approaches emphasize the importance of using biomarkers in the study of periodontitis.^28,72^ Indeed, due to their relevance to the etiopathogenesis of periodontal disease, MMP-8 and IL-6 have been extensively employed in periodontitis studies. The levels of MMP-8 and IL-6 in GCF have shown significant potential to identify individuals with periodontitis and reflect its severity,^25–30^ exhibiting ROC values of 0.92 and 0.93 for MMP-8 and IL-6, respectively,^28^ distinguishing between healthy and periodontitis patients. In this study, and to validate the proteomic findings, western blot analyses were conducted including periodontally healthy patients as well. MMP-8, IL-6, CA1, and CA2 levels yielded higher values in GCF from periodontal patients compared to healthy patients. Notably, the highest levels were observed during the progression of periodontitis (Fig. 5e). Additionally, western blot assays confirmed the absence of FTH1 in PG, a phenomenon closely associated with ferritinophagy, which is considered to be a form of ferroptosis.^73^ The matrix of correlations among the key protein abundances revealed strong connections between ferroptosis-associated proteins, DAMPs molecules, and hub proteins. Considering these findings and protein types, ROC curves were generated, especially focusing on CA1 and CA2, which are crucial hub proteins in disease progression, as found previously.^3^ These enzymes displayed higher abundance in PG compared to NP, in both western blot and mass spectrometry assays, and their AUC values approached 0.8, underscoring their potential as periodontitis progression biomarkers.

Cytokine balance plays a central role in inflammation.^74^ IL-6 is an important cytokine involved in the regulation of the host innate immune response to bacterial infection and its involvement in the onset of inflammatory responses, such as periodontitis, is well recognized.^74,75^ IL-6 has pleiotropic effects on lymphocyte promotion and tissue destruction, predominantly mediating B cell activation and recruitment.^75^ Along with the highest levels of IL-6 in the PG group (Fig. 5e), the present study observed a notably higher representation of plasma B cell clusters (24.5%) in the scRNA-seq dataset from periodontal patients compared to those from periodontally healthy patients (0.8%), as indicated in Fig. 6a and supplementary table 4. It has been described that IL-6 is produced by different cell types,^76^ including gingival fibroblast after stimulation with inflammation-related molecules.^77–79^ It is well accepted that fibroblasts participate in immune, inflammatory, and reparative cascades in the periodontal tissues.^80–83^ Interestingly, in this study, the highest IL-6 expression values were found in the fibroblast clusters (Fig. 6e, f).

DCN, an extracellular matrix protein constitutively synthesized and secreted by fibroblasts,^84^ contributes to immune regulation, inflammatory diseases, autophagy, angiogenesis, cell cycle, wound healing, and fibrosis.^85^ Importantly, recent studies have demonstrated that released DCN acts as a DAMP molecule,^86^ activating downstream MAPK and NF-κB pathways, resulting in an increase in the cytokines TNF-α, IL-6, IL-12, and IL-10, maintaining a pro-inflammatory state.^86–88^ Additionally, pro-Inflammatory cytokines can regulate the expression of DCN,^89–91^ and a marked increase of DCN mRNA expression in tissue with periodontitis has been found.^92^ Besides, gingival fibroblasts exhibited active in vitro expression of IL-6 mRNA when stimulated by IL-1β, as an initial response to inflammation, followed by increased expression of DCN, collagen, and TGF-β over time.^93^ Interestingly, our data showed that DCN was predominantly expressed in the fibroblast cluster (Fig. 6e, f). Regarding GCF protein levels, DCN was detected in all 18 samples of the NP group, while it was found in only one of the 18 samples from the PG group, exhibiting significantly higher relative abundance in NP (*P* < 0.05).

Zhao et al. revealed that gingival fibroblasts undergo ferroptosis and high levels of fibroblast-derived IL-6 were identified as a hallmark of the pro-inflammatory immune responses during periodontitis. This inflammatory reaction, coupled with the tissue damage and bone loss associated with periodontitis, was alleviated through the inhibition of ferroptosis.^19^ Furthermore, in a cellular model, a novel mechanistic understanding of the loss of periodontal-derived fibroblast during the progression of periodontitis was proposed, through the activation of NCOA4-mediated ferritinophagy, ultimately resulting in fibroblasts ferroptosis.^73^ These results are in agreement with our findings, characterized by high levels of IL-6 and the absence of FTH1 found in GCF samples from progressive periodontal pockets. In a recent research, which used a similar bioinformatic approach to identify cell clusters and explore the expression of different genes associated with cell death, specific genes associated with ferroptosis, pyroptosis, and necroptosis were observed in periodontitis patients compared with healthy controls.^94^ Collectively, the observed variations in IL-6, FTH1 and DCN abundance levels between NP and PG groups, combined with the alignment of DEPs with scRNA-seq gene expression levels, indicate a significant susceptibility of fibroblast to undergo ferroptosis during periodontitis progression.

The discovery of a significant type of regulated cell death linked to disease progression presents a novel and previously unexplored perspective for understanding the development of periodontitis. Notably, cell death may elucidate DAMPs release, known for activating or boosting the immune response,^95,96^ potentially contributing to the immune-inflammatory dysregulation seen in periodontitis.

In conclusion, this study presents compelling evidence that periodontitis progresses at a faster rate than traditionally believed, with distinctive proteome profiles in GCF between progressive and non-progressive periodontitis. Herein, SNCA, CA1, HBB, SLC4A1, and ANK1, were identified as hub proteins of periodontitis progression. Furthermore, to the best of our knowledge, for the first time, ferroptosis was associated with the clinical progression of periodontitis. These findings open new avenues for understanding the disease and may lead to innovative diagnostic and therapeutic strategies.

## Material and methods

**Fig. 7:**
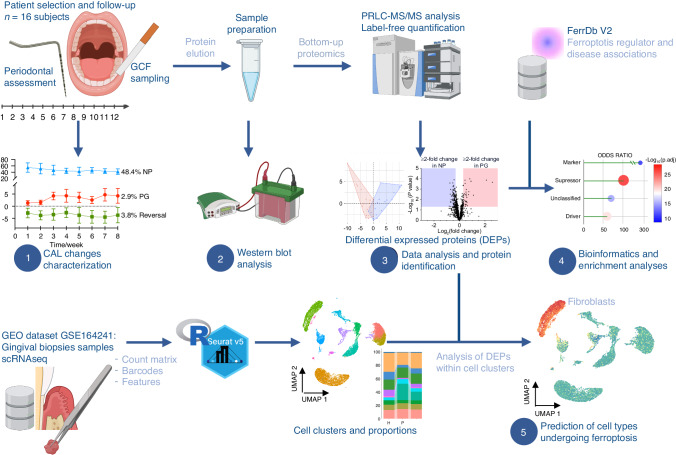
Schematic workflow of the experimental strategy. Sixteen patients diagnosed with periodontitis underwent a twelve-week follow-up to identify disease progression through clinical evaluations. A standardized GCF collection was performed on pre-selected pockets in the same locations over time. Clinical metrics and characterization of clinical CAL changes were conducted, and the proportions of progressing, non-progressing, and regressing sites were determined. The clinical progression of periodontitis was defined at a site level, wherein an increase in CAL ≥ 2 mm was scored. For further analyses, the PG group consisted of GCF-sampled pockets exhibiting disease progression, while the NP group was composed of GCF-sampled pockets exhibiting disease stability (non-progressing pockets). Subsequent mass spectrometry analysis was carried out. Clinical metrics, proteomic data, in silico methods, and bioinformatics tools were integrated to identify differentially expressed proteins (DEPs) linked to periodontitis progression and explore their potential connection to ferroptosis. Western blot analyses were conducted to validate key findings. Finally, on an external scRNA-seq dataset, cell sorting and clustering were performed, and an analysis of the expression levels was conducted to provide a deeper interpretation of the DEPs found in the GCF proteome during the clinical progression of periodontitis

A schematic workflow of the experimental strategy is presented in Fig. 7.

### Subjects

Sixteen subjects ranging in age from 35 to 65 years, registered on the waiting list for periodontal treatment at the Dental Clinic of the Faculty of Dentistry, University of Chile, were included in the study,^3^ all of whom completed the clinical follow-up. To mitigate the influence of factors such as smoking,^97–99^ female sexual hormones,^100^ and systemic chronic conditions^101^ on periodontal tissues, only healthy, non-smoking male patients, possessing a minimum of 14 teeth were included. Additionally, patients with recent antibiotic use or any periodontal treatment within the past 12 months, including supragingival scaling, were excluded from the study.

While these restrictions may limit the external validity of our findings, they are essential for elucidating the molecular events at the proteomic level during the clinical progression of periodontitis.

This study was approved by the Ethical Committee of the Faculty of Dentistry (protocol number 2016/11), University of Chile, and was conducted according to the Helsinki Declaration of 1975, as revised in 2013. Before the initiation of the study, all subjects signed an informed consent form explaining the risks and benefits involved.

### Clinical assessments

Clinical measurements were conducted by two calibrated periodontists using a first-generation 15 UNC periodontal probe (Hu-Friedy, Chicago, IL, USA). Probing depth (PD) and gingival margin (GM) were recorded at six sites on all teeth except the third molars, with measurements rounded to the nearest millimeter. CAL level was determined by subtracting PD from GM. Patients were diagnosed with periodontitis when the loss of periodontal tissue support due to inflammation exceeded the threshold of CAL ≥ 3 mm in ≥2 non-adjacent teeth, and alveolar bone loss was radiographically determined.^2^ Each patient was evaluated by the same periodontist throughout all clinical assessments. For western blot analysis, periodontal healthy subjects were also included. Periodontal health was diagnosed when the following conditions were met: PD < 3 mm, bleeding index <15%, and the absence of gingival inflammatory characteristics.

### Identification of clinical disease progression

All patients underwent a full mouth periodontal examination and GCF collection once a week, consistently on the same day and time, until the clinical progression of periodontitis was identified or until the completion of the 12-week clinical monitoring follow-up. The determination of clinical progression of periodontitis was determined at the site level, where progressive periodontal pockets were defined by an increase in CAL of ≥2 mm compared to the initial periodontal record. Conversely, non-progressive or stable periodontal pockets were defined as periodontal pockets with stable CAL during the entire follow-up period. Patients with two or more progressing sites at any periodontal pocket discontinued the follow-up and received immediate periodontal treatment. Additionally, the patients with no progression after three months also entered the treatment phase.

### GCF samples collection

After the initial periodontal diagnostic evaluation, in each patient, a mean of ten periodontal pockets for convenience were selected for GCF sampling over time. GCF sampling was performed before each weekly full-mouth periodontal evaluation. Once the clinical field isolation was set, GCF samples were collected in duplicates using Periopaper strips (Plainview, NY, USA), with each strip inserted 1 mm within the periodontal pocket for 30 s, followed by a 60-s interval before the next sampling. This method ensured controlled GCF volume collection by time from each periodontal pocket, considering variations in volume and protein concentration between active and inactive sites.^26,102^ Mechanical irritation was avoided, and strips contaminated with blood were discarded. The collected samples were immediately stored at −80 °C for later analysis.

For subsequent analysis, the ‘progression group’ (PG) included GCF samples from eighteen sites exhibiting disease progression (increase in CAL ≥ 2 mm), while the ‘non-progression group’ (NP) included eighteen GCF samples from stable sites (no CAL changes) matched in tooth type and monitoring time with PG sites within the same patients. In contrast to other studies where samples were pooled, we analyzed individually the 18 samples obtained for each group in this label-free quantification study.^3^

### Sample preparation for mass spectrometry analysis

Protein elution and sample preparation were performed as previously explained.^3^ In summary, proteins were extracted from Periopaper strips, and after being eluted in PBS with a protease inhibitor, were processed. Protein concentration was measured using a BCA Protein Assay Kit. Proteins were reduced, alkylated, and digested with trypsin. The digestion was halted with formic acid. Then, samples were cleaned on a C18 Hypersep plate (Thermo Fisher Scientific, Waltham, MA, USA) with 5–7 µL bed volume, dried using a vacuum concentrator (Eppendorf), and resuspended in 10 µL of 0.1% FA and 2% acetonitrile.

### High-pressure reverse-phase liquid chromatography coupled to tandem mass spectrometry (PRLC-MS/MS) analysis

PRLC-MS/MS analysis was conducted as described previously.^3^ Briefly, Peptide separation was achieved using a 50 cm EASY-Spray C18 column connected to a high-performance liquid chromatography system. A 90-min linear gradient from 4% to 26% ACN in 0.1% FA was used at a flow rate of 300 nL/min. Mass spectrometry was conducted using an Orbitrap Q Exactive HF instrument in data-dependent acquisition mode, with a mass range of 375–1 500 m/z for full spectra. The 17 most intense peptide precursors were fragmented by HCD, and analysis parameters included 28% normalized collision energy and 45 s of dynamic exclusion time. Blank runs were added after each analysis of biological replicates to minimize the carry-over.

### Western blot analysis

40 µL of eluted GCF protein samples were separated on a 10% SDS-polyacrylamide gel and transferred to a PVDF membrane using the Trans-Blot Turbo System from Bio-Rad (Bio-Rad Laboratories, Hercules, CA, USA). The PVDF membrane was then incubated in a blocking solution (TBS 0.1% Tween-20, 5% BSA) for 1 hour. Primary antibodies were diluted in a 3% BSA solution and left to incubate overnight at 4 °C. After three washes in a 0.1% TBS-Tween-20 solution, the membrane was exposed to secondary antibodies for 2 hours at room temperature. Following another set of washes, the membranes were scanned on an Odyssey-FC system from LI-COR Biosciences. This experiment was conducted in duplicate. Primary antibodies and their respective concentrations were as follows: anti-CA1 # NBP2-76980, 1:1 000 (Novus Biological), anti-CA2 # NBP2-89522, 1:1 000 (Novus Biological), anti-MMP8 # AB56303, 1:1 000 (Abcam Inc), anti-IL-6 # AB6672, 1:500 (Abcam Inc), anti-FTH1 # 858901, 1:1 000 (BIOLEGEND).

### scRNA sequencing data processing and analysis

The analysis of the single-cell RNA sequencing dataset obtained from the Gene Expression Omnibus (GEO) database (GSE164241, available at: https://www.ncbi.nlm.nih.gov/geo/query/acc.cgi?acc=GSE164241) from human chronic periodontitis and clinically healthy marginal gingival tissues aimed at providing an unbiased assessment of heterogeneous cells at the single-cell level within the molecular complexity context of periodontal tissues.^103^ The primary objective was the exploration and approximation of the origin of proteomic differences at the cellular level. This approach employed the R package Seurat v5.0 for comprehensive data processing, covering quality control, normalization, batch correction, integration, feature selection, data scaling, linear reduction via PCA, cell clustering, UMAP and dimensionality reduction with clustering annotations.^104^

During the quality control phase, cells with fewer than 500 genes, more than 5 000 genes, or more than 15% mitochondrial genes were excluded. Gene expression underwent normalization and scaling using the “LogNormalize” method. Post-normalization, 2 000 highly variable genes (HVG) were identified for each sample using the “vst” method. To overcome extensive technical noise in any single feature for scRNA-seq data, a ranking of PCs based on the percentage of variance explained by each PC was used to scale the data. The FindAllClusters function categorized cells into 21 different clusters with a resolution of 0.2. Using the FindAllMarkers function with logfc.threshold = 0.25, differentially expressed genes (DEGs) were identified for each cluster. Cell type identification relied on the DEGs in each cluster and was validated manually.

### Data analysis, bioinformatics and statistical analysis

Raw data files were analyzed using Proteome Discoverer v2.4 software (Thermo Fisher Scientific, Waltham, MA, USA) and searched against a human protein database^105^ with Mascot Server 2.5.1. Parameters included up to two missed cleavage sites for trypsin, precursor mass tolerance of 10×10^−6^, and 0.02 Da for the HCD fragment ions. Dynamic modifications were set for oxidation on methionine, deamidation of asparagine and glutamine, and acetylation of N-termini. Quantification considered both unique and razor peptides. Relative protein abundances between sample groups were compared using moderated t-tests with Benjamini–Hochberg correction for multiple comparisons.

For in-depth gene and protein analysis based on gene ontology, KEGG, Reactome, and WIKI databases, ShinyGO 0.77^106^ was used. Gene set enrichment analysis and protein-protein interaction networks were generated using the STRING^107^ web tool with an interaction score threshold of >0.4 and further analyzed in Cytoscape.^108^ The top 10 hub proteins in the PPI network were characterized using the CytoHubba plugin.^109^

In silico analysis to identify genes related to periodontitis progression utilized the Génie Data-mining tool.^110^ A search for “biomarkers [Mesh] Periodontitis [Mesh] Progression” identified 184 abstracts as a training set with defined criteria for significance. To find only significant coding genes, a cut-off *P*-value of <0.01 for abstracts and a false positive discovery rate (FDR) of <0.01 for genes were established. Fisher’s exact test was used to define the relationship between genes and search topics.

Human genes associated with ferroptosis were extracted from FerrDb V2.^111^ Overrepresentation enrichment analysis classified genes as markers, suppressors, and drivers of ferroptosis based on a cut-off *P*-value of <0.05.

Western blot band intensities were quantified using ImageJ 1.53 K software, and data analysis was performed using One-way ANOVA with the Holm-Sidak method. For data analysis and visualization, GraphPad Prism 9 software was used (GraphPad Software Inc., San Diego, CA, USA).

Data are presented as the mean ± SEM, Min-Max count, and percentage for clinical evaluation and eluted protein characterization from GCF samples. Proteomic analysis data are presented as Log_2_(relative abundances), Log_2_(fold change), and Log_10_(*P*-value). Comparisons of relative protein abundances between the groups were made using moderated t-tests with Benjamini–Hochberg correction for multiple comparisons. Bioinformatic analysis data is presented as fold enrichment and Log_10_ (*P-*value). Results with *P* < 0.05 were considered statistically significant.

### Supplementary information

Supplemental material

## Data Availability

The proteomic data has been deposited on PRIDE,^112^ (https://www.proteomexchange.org/, accession number: PXD046328). PRIDE is an official member of ProteomeXchange Consortium.^113^ The Consortium coordinates global data submission and dissemination pipelines among key proteomics repositories, promoting open data policies in the field. Deidentified clinical data can be made accessible upon publication, following approval of a proposal along with a signed data access agreement through the corresponding author.
