# Supplementary material for: A multi-platform analysis of human gingival crevicular fluid reveals ferroptosis as a relevant regulated cell death mechanism during the clinical progression of periodontitis
Source: Int J Oral Sci. 2024 May 27;16:43. doi: 10.1038/s41368-024-00306-y (PMC11130186; doi:10.1038/s41368-024-00306-y)
Supplement: Supplementary file 1 — Supplemental material [file 41368_2024_306_MOESM1_ESM.pdf]

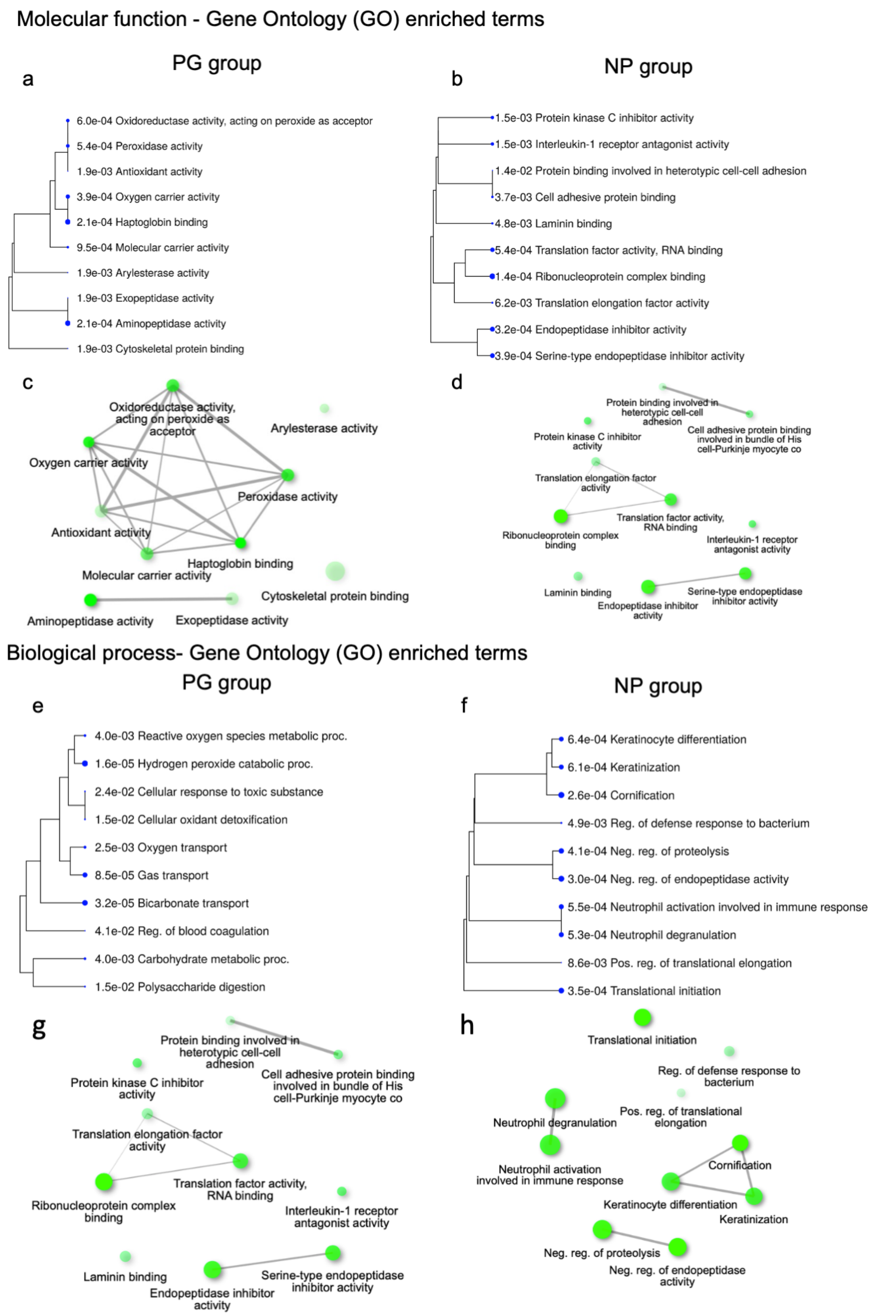

Molecular function - Gene Ontology (GO) enriched terms
PG group
a
6.0e-04 Oxidoreductase activity, acting on peroxide as acceptor
5.4e-04 Peroxidase activity
1.9e-03 Antioxidant activity
3.9e-04 Oxygen carrier activity
2.1e-04 Haptoglobin binding
9.5e-04 Molecular carrier activity
1.9e-03 Arylesterase activity
1.9e-03 Exopeptidase activity
2.1e-04 Aminopeptidase activity
1.9e-03 Cytoskeletal protein binding
NP group
b
1.5e-03 Protein kinase C inhibitor activity
1.5e-03 Interleukin-1 receptor antagonist activity
1.4e-02 Protein binding involved in heterotypic cell-cell adhesion
3.7e-03 Cell adhesive protein binding
4.8e-03 Laminin binding
5.4e-04 Translation factor activity, RNA binding
1.4e-04 Ribonucleoprotein complex binding
6.2e-03 Translation elongation factor activity
3.2e-04 Endopeptidase inhibitor activity
3.9e-04 Serine-type endopeptidase inhibitor activity
c
Oxidoreductase activity, acting on peroxide as acceptor
Arylesterase activity
Oxygen carrier activity
Peroxidase activity
Antioxidant activity
Haptoglobin binding
Molecular carrier activity
Cytoskeletal protein binding
Aminopeptidase activity
Exopeptidase activity
d
Protein binding involved in heterotypic cell-cell adhesion
Protein kinase C inhibitor activity
Cell adhesive protein binding involved in bundle of His cell-Purkinje myocyte co
Translation elongation factor activity
Translation factor activity, RNA binding
Ribonucleoprotein complex binding
Interleukin-1 receptor antagonist activity
Laminin binding
Serine-type endopeptidase inhibitor activity
Endopeptidase inhibitor activity
Biological process- Gene Ontology (GO) enriched terms
PG group
e
4.0e-03 Reactive oxygen species metabolic proc.
1.6e-05 Hydrogen peroxide catabolic proc.
2.4e-02 Cellular response to toxic substance
1.5e-02 Cellular oxidant detoxification
2.5e-03 Oxygen transport
8.5e-05 Gas transport
3.2e-05 Bicarbonate transport
4.1e-02 Reg. of blood coagulation
4.0e-03 Carbohydrate metabolic proc.
1.5e-02 Polysaccharide digestion
NP group
f
6.4e-04 Keratinocyte differentiation
6.1e-04 Keratinization
2.6e-04 Cornification
4.9e-03 Reg. of defense response to bacterium
4.1e-04 Neg. reg. of proteolysis
3.0e-04 Neg. reg. of endopeptidase activity
5.5e-04 Neutrophil activation involved in immune response
5.3e-04 Neutrophil degranulation
8.6e-03 Pos. reg. of translational elongation
3.5e-04 Translational initiation
g
Protein binding involved in heterotypic cell-cell adhesion
Protein kinase C inhibitor activity
Cell adhesive protein binding involved in bundle of His cell-Purkinje myocyte co
Translation elongation factor activity
Translation factor activity, RNA binding
Ribonucleoprotein complex binding
Interleukin-1 receptor antagonist activity
Laminin binding
Serine-type endopeptidase inhibitor activity
Endopeptidase inhibitor activity
h
Translational initiation
Reg. of defense response to bacterium
Pos. reg. of translational elongation
Neutrophil degranulation
Neutrophil activation involved in immune response
Cornification
Keratinocyte differentiation
Keratinization
Neg. reg. of proteolysis
Neg. reg. of endopeptidase activity

PG – Protein-protein interactions according to STRING

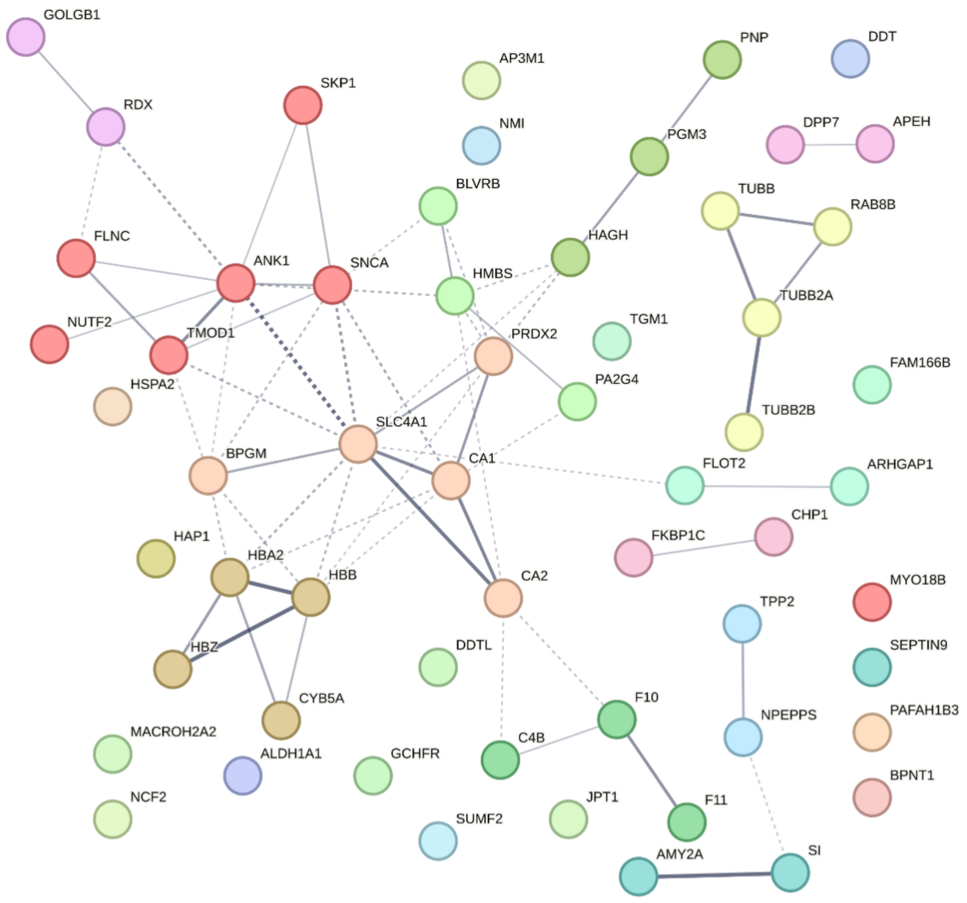

PG – Protein-protein interactions according to STRING

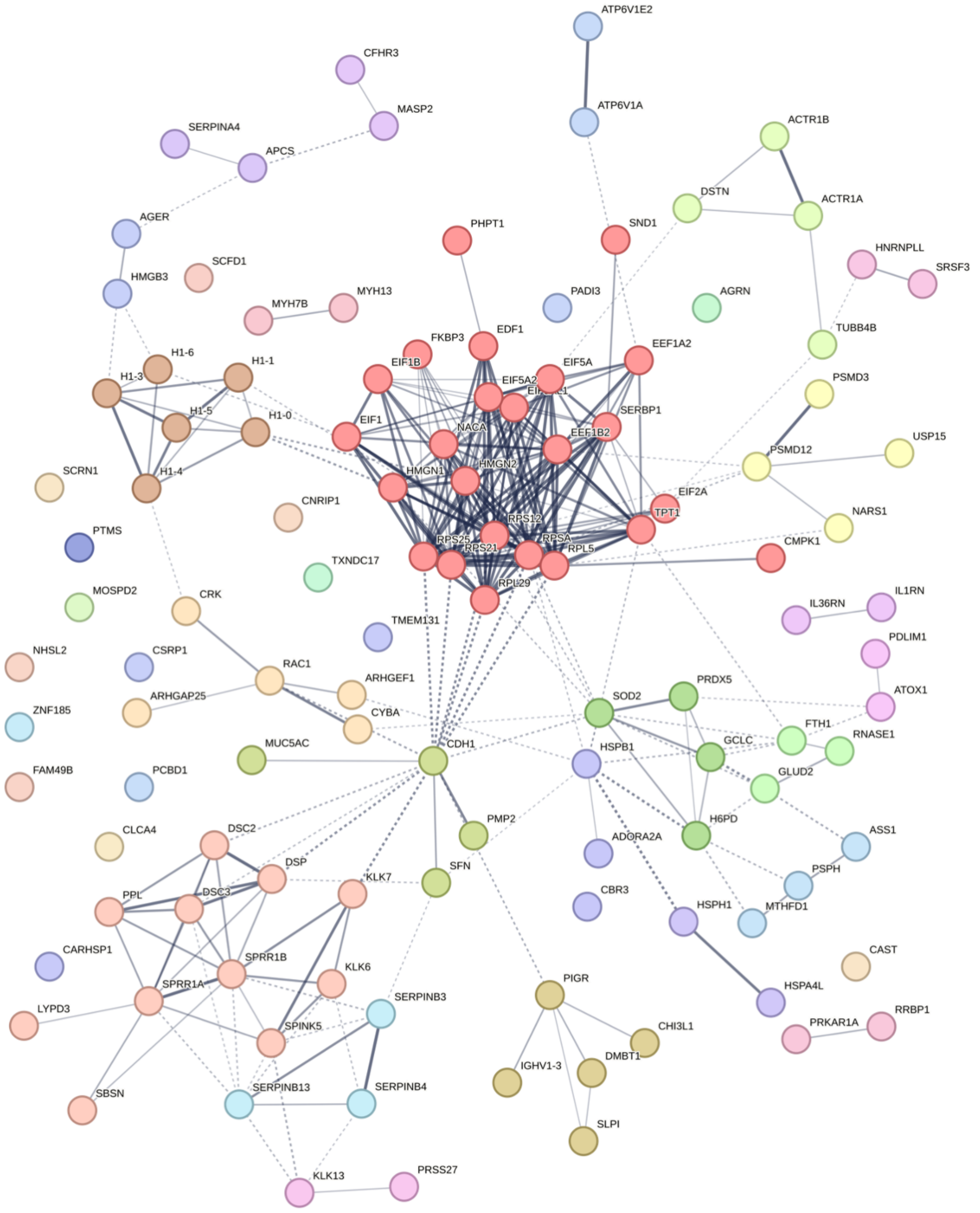

a. PG - Hub genes identificacion by different methods

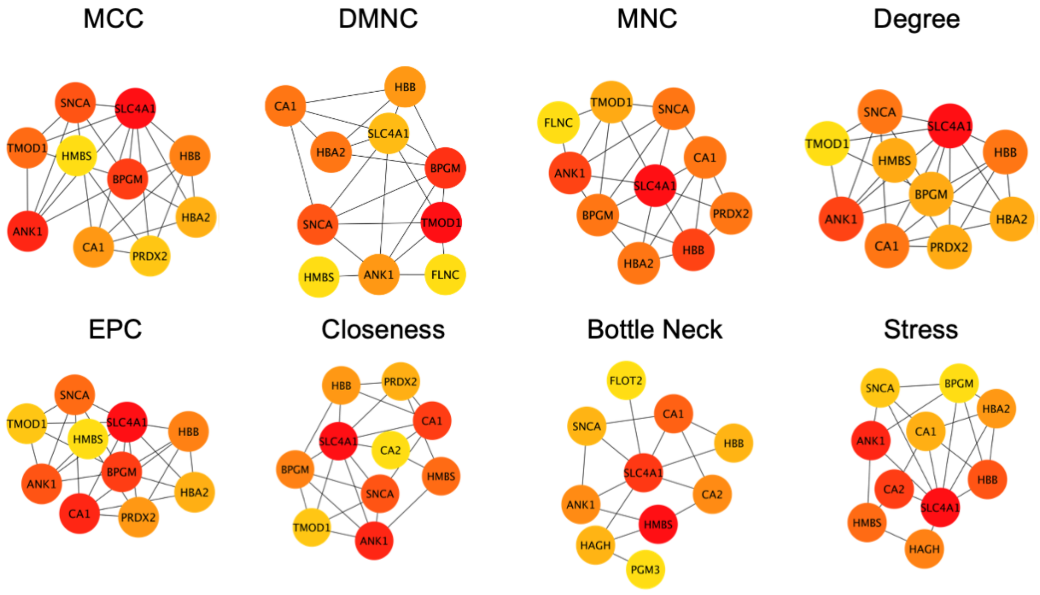

b. NP - Hub genes identificacion by different methods

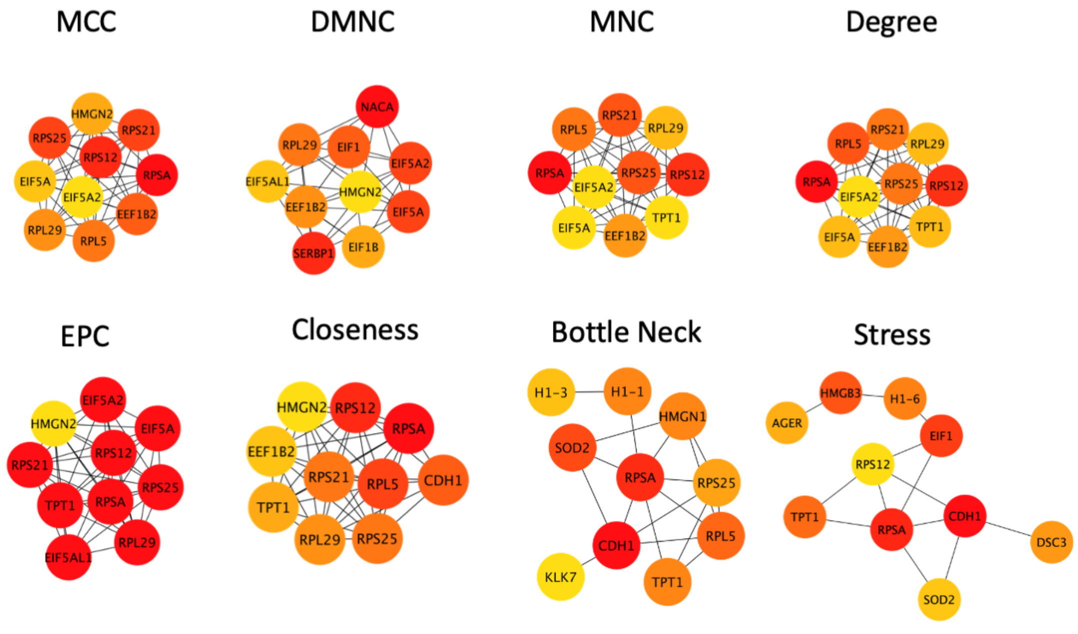

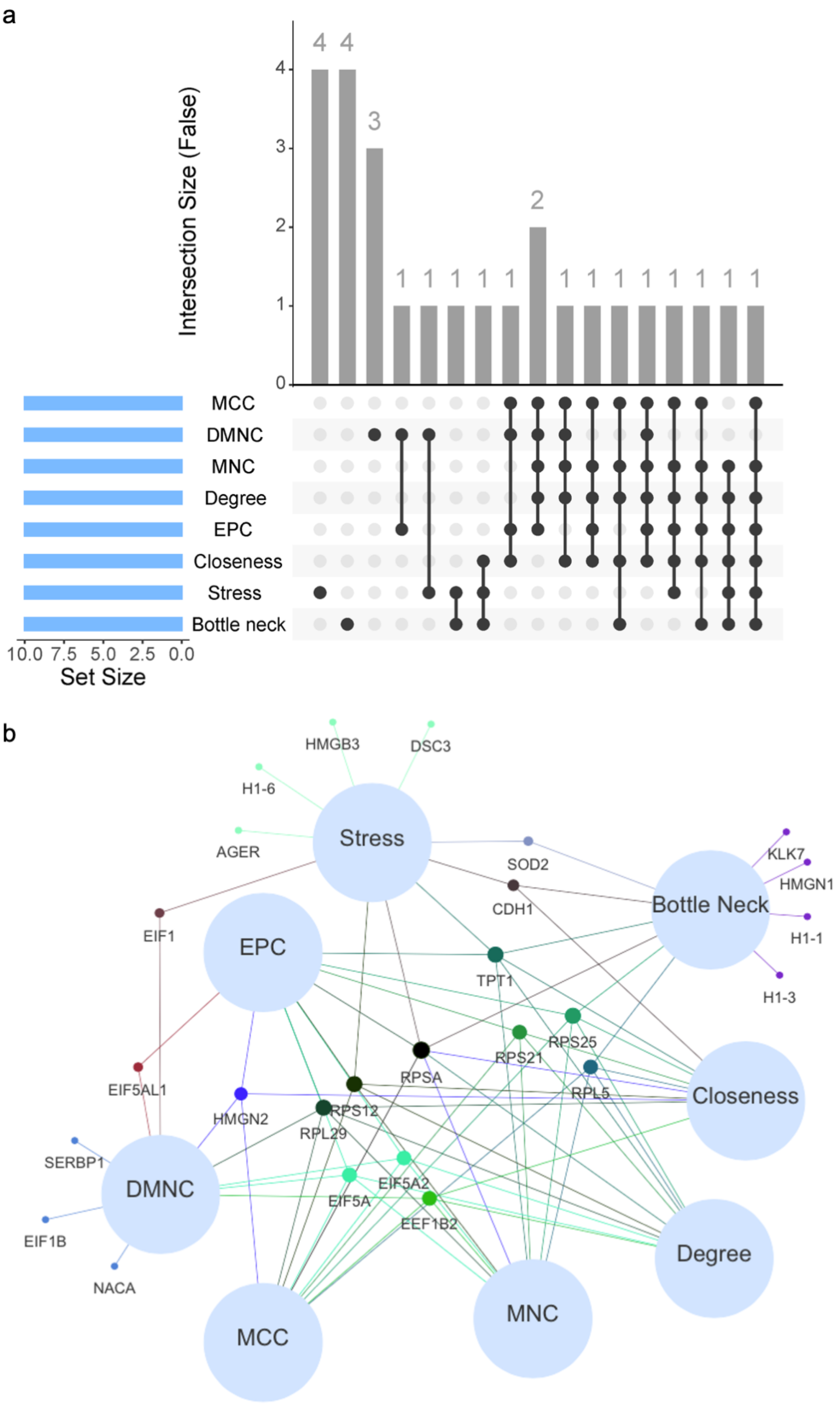
a
Intersection Size (False)
4
4
3
2
1
0
MCC
DMNC
MNC
Degree
EPC
Closeness
Stress
Bottle neck
10.0 7.5 5.0 2.5 0.0
Set Size
b
Stress
Bottle Neck
EPC
Closeness
DMNC
Degree
MCC
MNC
HMGB3
DSC3
H1-6
AGER
SOD2
CDH1
KLK7
HMGN1
H1-1
H1-3
EIF1
TPT1
RPS25
RPS21
RPSA
RPL5
EIF5AL1
HMGN2
RPS12
RPL29
SERBP1
EIF5A
EIF5A2
EEF1B2
EIF1B
NACA

Cytoscape-based ClueGo/CluePedia pathway analysis and visualization

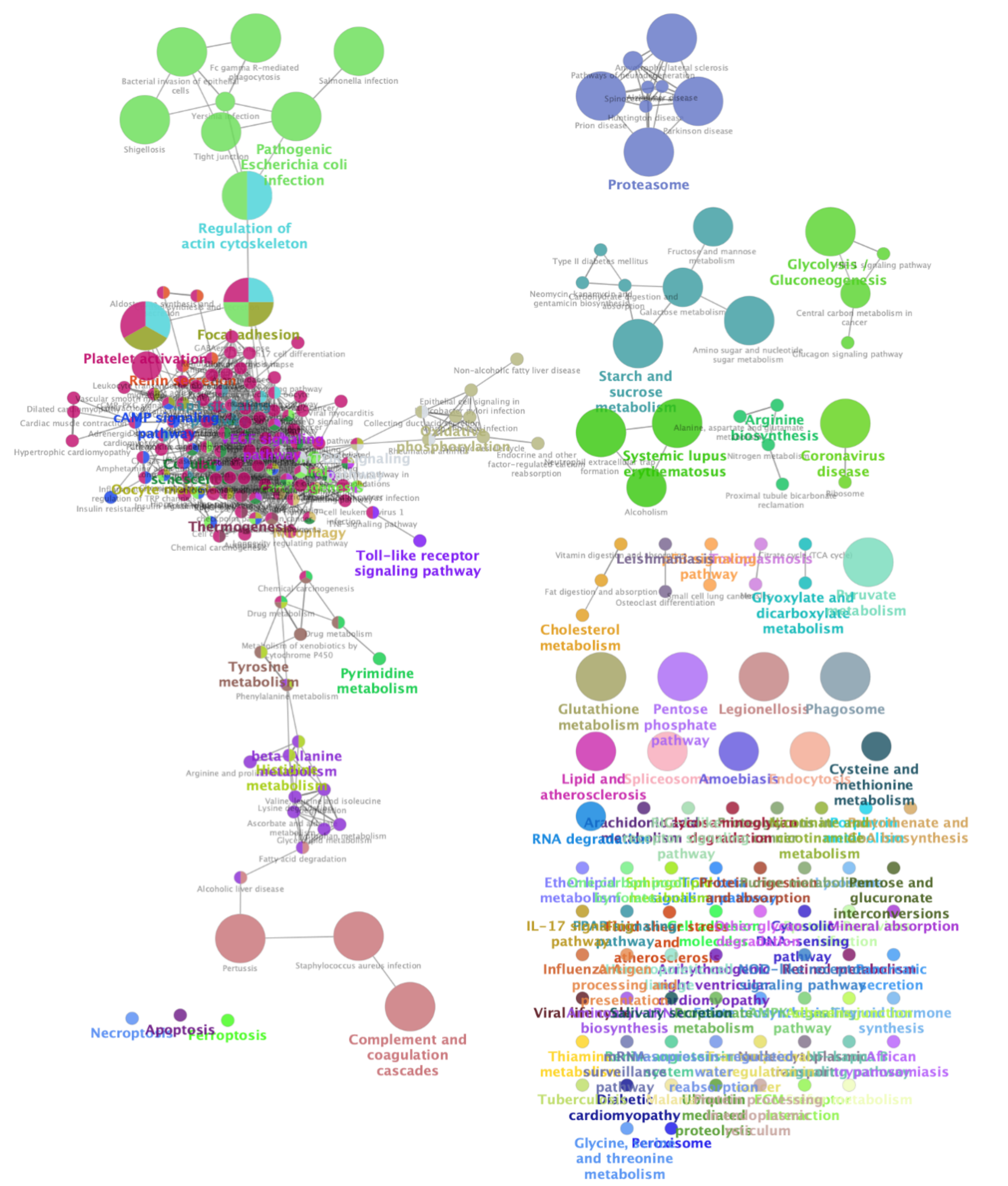

a

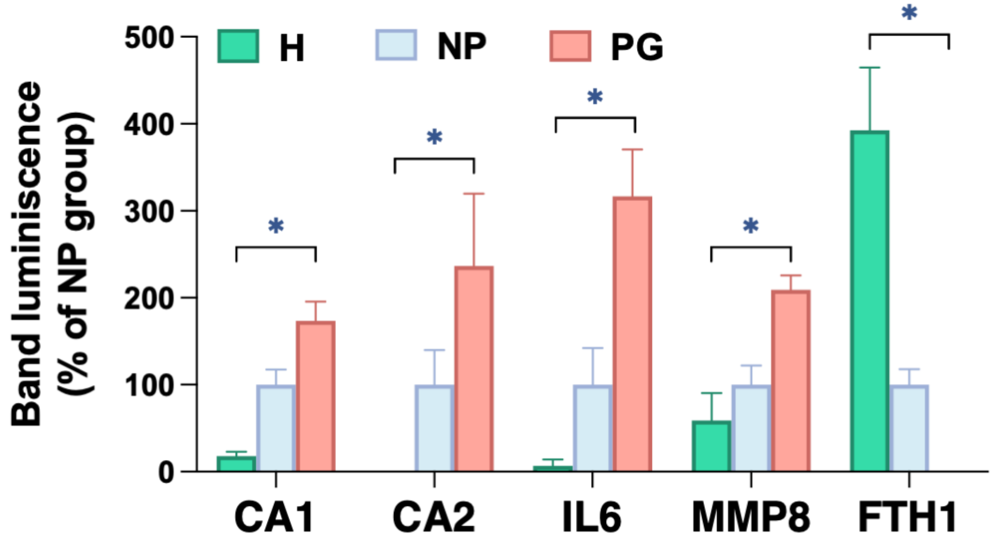

H
NP
PG
Band luminiscence (% of NP group)
500
400
300
200
100
0
*
CA1
CA2
IL6
MMP8
FTH1

b

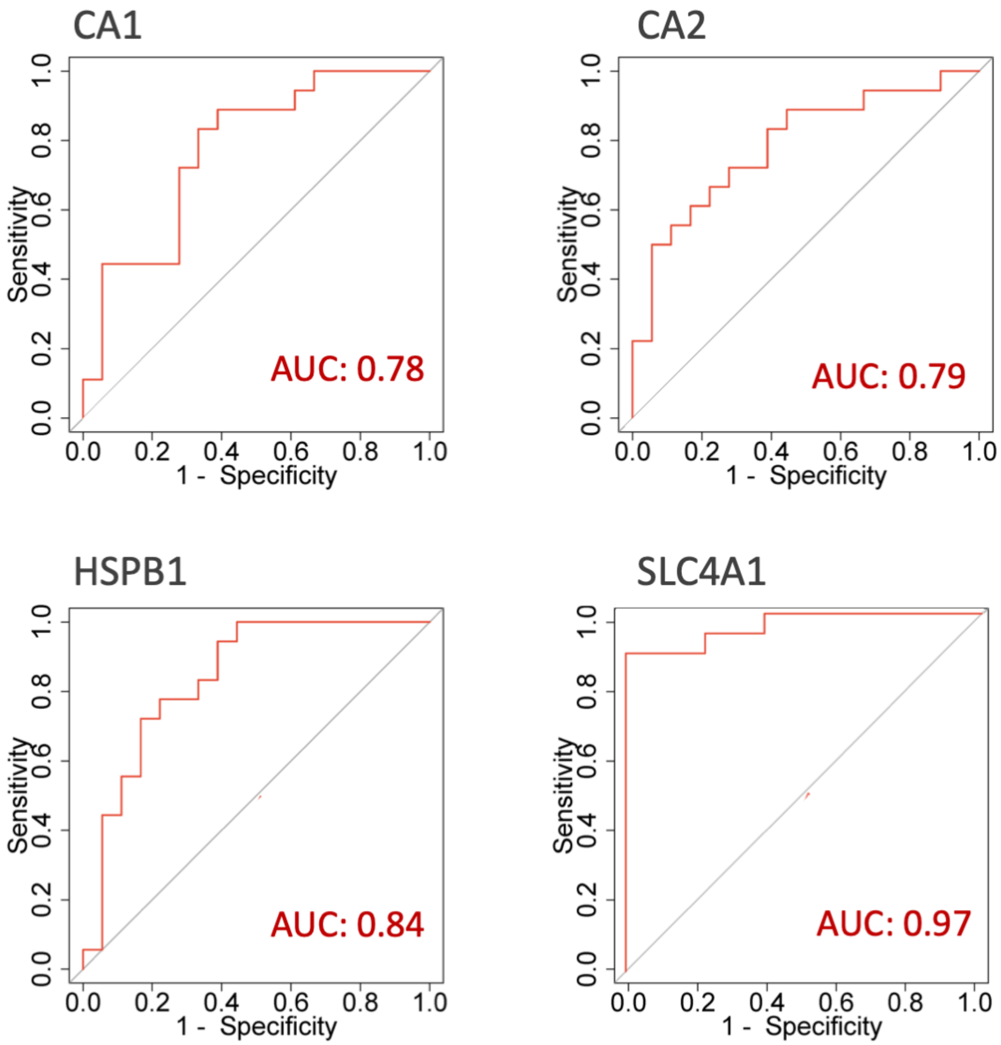

CA1
AUC: 0.78
CA2
AUC: 0.79
HSPB1
AUC: 0.84
SLC4A1
AUC: 0.97
Sensitivity
1 - Specificity
0.0
0.2
0.4
0.6
0.8
1.0

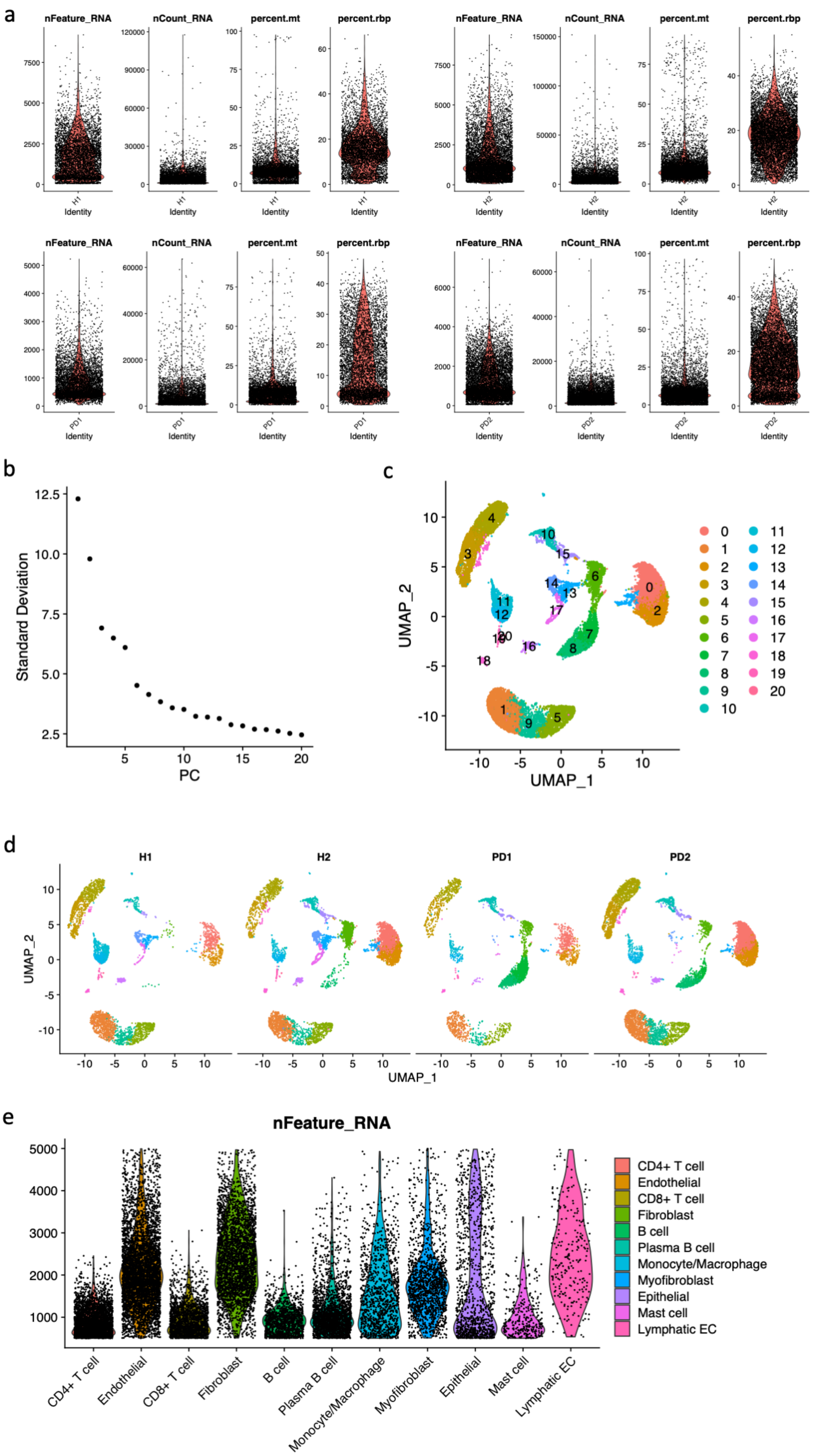
a
nFeature_RNA
nCount_RNA
percent.mt
percent.rbp
H1
H2
PD1
PD2
Identity
b
Standard Deviation
PC
c
UMAP_1
UMAP_2
0 1 2 3 4 5 6 7 8 9 10 11 12 13 14 15 16 17 18 19 20
d
H1
H2
PD1
PD2
UMAP_1
UMAP_2
e
nFeature_RNA
CD4+ T cell
Endothelial
CD8+ T cell
Fibroblast
B cell
Plasma B cell
Monocyte/Macrophage
Myofibroblast
Epithelial
Mast cell
Lymphatic EC

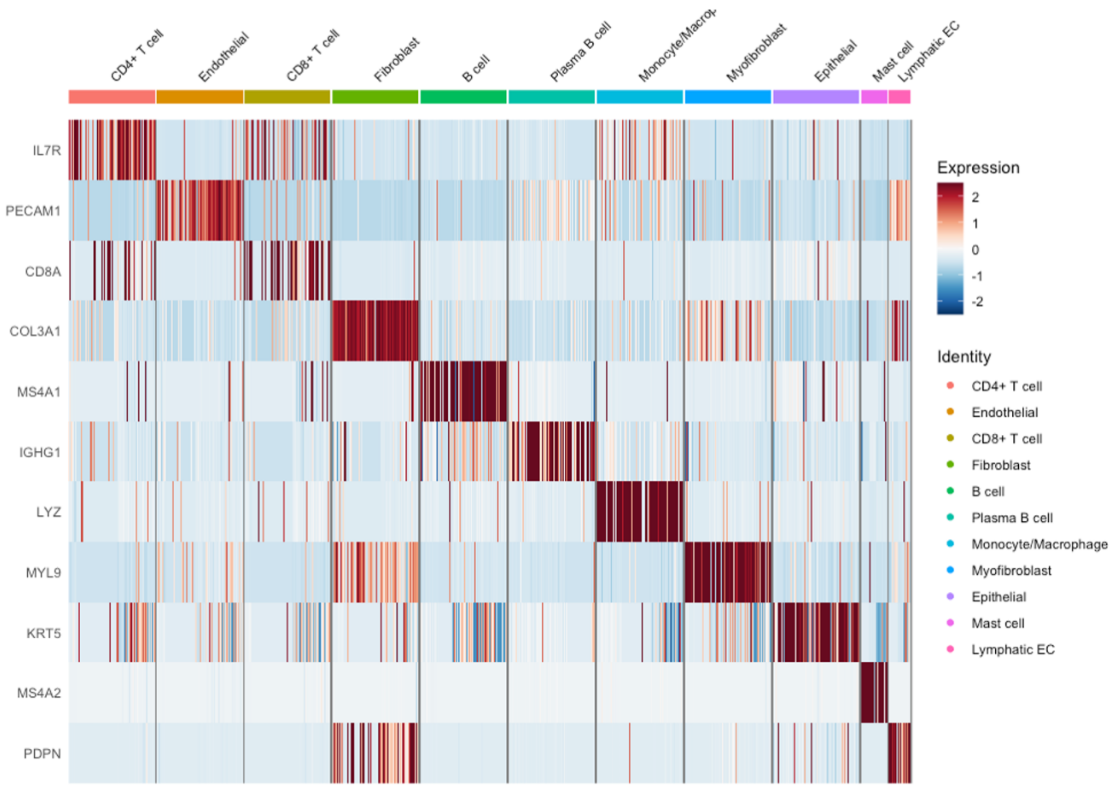
CD4+ T cell
Endothelial
CD8+ T cell
Fibroblast
B cell
Plasma B cell
Monocyte/Macrop
Myofibroblast
Epithelial
Mast cell
Lymphatic EC
IL7R
PECAM1
CD8A
COL3A1
MS4A1
IGHG1
LYZ
MYL9
KRT5
MS4A2
PDPN
Expression
2
1
0
-1
-2
Identity
CD4+ T cell
Endothelial
CD8+ T cell
Fibroblast
B cell
Plasma B cell
Monocyte/Macrophage
Myofibroblast
Epithelial
Mast cell
Lymphatic EC

SUPPLEMENTARY FIGURE LEGENDS

Supplementary Figure 1. Hierarchical clustering tree and interactome map of enriched terms in Gene Ontology.(a, c) Hierarchical clustering tree of molecular function and biological process of the PG group and the NP group, respectively. The hierarchical clustering tree summarizes the correlation among significant pathways related to the gene set. Pathways with many shared genes are clustered together. Larger dots indicate more significant p-values. (c, d) Interactome networks of GO-MF enriched-terms ($p < 0.005$, FDR $Q < 0.05$). (e, f) Interactome networks of GO-BP enriched-terms ($p < 0.005$, FDR $Q < 0.05$). The enrichment interactome map organizes enriched terms into a network with edges connecting overlapping gene sets. In this way, mutually overlapping gene sets tend to cluster together, making it easy to identify functional modules.

Supplementary Figure 2. Construction and analysis of PPI network of the exclusive and differentially expressed proteins in the PG group. Network nodes represent proteins, and edges represent protein-protein associations, with the thickness reflecting biological confidence. The minimum required interaction score was set to medium confidence (0.4), and disconnected nodes in the network were not hidden.

Supplementary Figure 3. Construction and analysis of PPI network of the exclusive and differentially expressed proteins in the NP group. Network nodes represent proteins, and edges represent protein-protein associations, with the thickness reflecting biological confidence. The minimum required interaction score was set to medium confidence (0.4), and disconnected nodes in the network were not hidden.

Supplementary Figure 4. Identification of candidate hub genes. (a, b) PPI network analysis in PG and NP, respectively. PPI interactions were visualized in Cytoscape to identify the top 10 hub genes in network analysis using the MCC, DMNC, MNC, Degree, EPC, Closeness, Bottle Neck, and Stress methods in cytoHubba. The top ten nodes are shown with a color scheme from red (highly important) to yellow (very important) according to each algorithm.

Supplementary Figure 5. Cytoscape-based ClueGo/CluePedia pathway analysis and visualization. The diagram consists of the protein-protein interaction of 1750 coding genes identified in silico in the GENIE database linked to MeSH terms "periodontitis" and "progression." Enriched pathways were obtained from the Kyoto Encyclopedia of Genes and Genome (KEGG) database. Terms are grouped based on shared genes (kappa score) and are shown in different colors. The size of nodes indicates the degree of significance. The most significant term defines the name of the group.

Supplementary Figure 6. Quality Control, Data Processing, and Analysis. (a) Violin plots depicting RNA feature counts, RNA counts, and the percentage of mitochondrial and ribosomal genes for healthy patients (H1 and H2) and periodontal patients (PD1 and PD2). (b) Ranking of principal components (PCs) based on the percentage of variance. (c) Identification of twenty-one cell clusters in human marginal gingival tissue based on specific genes, resulting in eleven labeled clusters derived from the pooled data of the whole-tissue scRNA-seq dataset (GES164241). Visualization of these clusters was achieved using uniform manifold approximation and projection (UMAP).

(d) UMAP projection of twenty-one cell clusters split by patient. (e) Violin plots of RNA feature counts after data processing and scaling, split by cluster after labeling.

Supplementary Figure 7. Heatmap of single-cell feature expression illustrating the cell marker expression for every cluster. High expression of a particular gene is indicated by red, while low expression is indicated by blue.

Supplementary Table 1. Demographic characteristics and details of studied patients

Variable	A	C	D	E	F	G	H	J	K	L	N	O	Q	R	S	T	Mean	SD	Total
Full follow-up	Yes	Yes	Yes	Yes	Yes	Yes	Yes	Yes	Yes	Yes	Yes	Yes	Yes	Yes	Yes	Yes			
Number of follow-up sessions	5	5	6	12	7	12	8	4	3	4	5	4	4	4	4	3	5,6	2,8	
Age, *years*	54	65	50	43	49	42	51	62	64	51	45	58	48	35	28	52	51,2	10	
Number of teeth	21	20	28	22	27	20	21	17	21	23	19	23	18	27	24	19	21,8	3,2	
Number of sites included in CAL assessments (*)	126	120	168	132	162	120	126	102	126	138	114	138	108	162	144	114			2100
Number of sampled sites	10	10	10	10	10	8	10	10	9	8	10	10	12	11	13	11	10,2	1,5	
PD initial	4,8	2,8	2,9	3,0	3,3	2,9	4,2	4,1	2,7	3,0	4,0	3,5	4,1	3,0	3,7	3,4	3,5	0,63	
CAL initial	6,9	4,1	3,2	3,0	3,7	3,0	6,0	5,7	3,8	4,9	5,0	4,2	4,8	2,3	2,8	5,4	4,3	1,30	
Detailed description of pockets at sampled sites	1.6 VD	1.7 PM	1.4 VM	1.7 PD	1.6 VD	1.7 VM	1.5 VM	1.7 VM	2.7 VD	1.7 PD	1.6 VM	2.2 VD	1.5 VM	1.4DV	1.1DP	4.5ML			
	1.6 PM	1.4 PM	1.4 PM	1.3 VD	1.6 PC	1.7 PM	1.4 PD	2.2 PM	2.7 PM	2.6 VD	1.6 PM	2.2 PD	1.5 VD	2.6DV	1.1DV	4.4DL			
	1.3 PM	1.3 PD	1.2 VM	1.2 VD	1.5 VD	1.6 VD	1.3 VM	4.7 VC	2.7 PD	2.6 PM	1.5 PM	2.7 VM	1.3 VM	2.7MV	2.6DV	4.5MV			
	2.4 PD	1.3 PM	2.7 VM	1.2 PM	1.5 PC	1.6 PD	1.1 PD	4.7 LC	2.6 VD	2.6 PC	1.1 VD	2.7 VC	1.3 VD	4.6DV	4.1DV	4.4DV			
	2.6 VC	1.2 VD	2.7 PM	1.1 PD	1.1 VD	2.7 VD	2.1 VD	4.5 VC	2.6 PM	2.7 VM	1.1 PM	2.7 PM	1.3 PM	4.6MV	4.2MV	3.5ML			
	3.7 VC	2.1 VD	3.6 VM	2.1 VM	2.6 VD	3.7 VD	2.2 PM	4.3 VD	2.6 PD	3.6 LC	1.1 PD	4.7 LM	1.2 VM	3.6MV	4.2DV	3.6ML			
	3.7 LD	2.2 VD	3.7 LM	2.1 VD	3.6 VM	3.7 LM	2.3 PM	4.2 LD	2.5 PD	3.6 LD	2.1 VM	4.7 VM	1.2 VD	3.6DV	4.2DL	3.6MV			
	4.3 LM	3.4 LD	3.7 LD	2.2 VD	3.6 LD	3.7 LM	2.7 VM	4.1 LM	4.6 VM	3.7 LD	3.1 VD	4.1 VM	1.1 VM	3.5ML	4.3MV	3.4MV			
	4.4 VM	3.5 LD	4.2 VD	2.3 VM	4.6 LD	-	3.4 LM	3.5 LD	4.6 LM	-	3.2 VD	3.1 VM	1.1 VD	3.6ML	4.3ML	3.4DV			
	4.7 LD	4.7 VD	4.7 LD	4.5 LM	4.7 VM	-	4.2 VD	4.4 LD	-	-	3.7 LM	3.7 VC	2.1 VM	4.5DL	4.6MV	3.5MV			
	-	-	-	-	-	-	-	-	-	-	-	-	2.1 VD	4.6ML	4.6ML	3.5DV			
	-	-	-	-	-	-	-	-	-	-	-	-	4.6 VM		4.6DV				
															4.6DL				
Numer of sampled sites matching clinical progression of periodontitis	2	1	1	1	1	0	1	1	0	0	1	2	1	2	2	2			18
Detailed description of progressive pockets at monitoring time	A4 3.7 LD	C5 1.3 MP	D4 4.7 LD	E3 1.2 VD	F7 1.5 VD	-	H8 2.2 PM	J4 4.7 VC	-	-	N5 1.5 PM	O4 2.2 VD	Q4 1.3 VD	R4 3.6MV	S4 1.1DV	T3 3.6MV			
	A5 1.6 PM	-	-	-	-	-	-	-	-	-	-	O4 3.7 VC	-	R4 3.6DV	S4 4.1DV	T3 3.6ML			
Numer of sampled sites counting for NP group	2	1	1	1	1	-	1	1	-	-	1	2	1	2	2	2			18
Detailed description of progressive pockets at monitoring time	A5 2.6 VC	C5 2.2VD	D4 2.7VM	E3 2.2 VD	F7 1.6PC	-	H8 1.1 DP	J4 4.5VC	-	-	N5 1.1PM	O4 3.1VM	Q4 1.5VD	R4 4.6MV	S4 4.3MV	T3 3.5ML			
	A5 4.7 LD	-	-	-	-	-	-	-	-	-	-	O4 4.1VM	-	R4 4.6ML	S4 4.3ML	T3 3.4MV			
Total sampled sites (**)	50	50	60	120	70	96	80	40	27	32	50	40	48	44	52	33			906

Detailed Description of the Average of Stable Sites (No CAL Changes) for Each Monitoring Time

Monitoring session	A	C	D	E	F	G	H	J	K	L	N	O	Q	R	S	T
0																
1	57,142857	46,666667	56	53,7878788	47,5308642	52,5	37,3015873	31,4	41,2698413	48,6	46,4912281	49,2753623	62,037037	75,9259259	82,638889	85,08772
2	37,301587	40,833333	60,7142857	50	45,6790123	59,1666667	50,7936508	27,5	20,6349206	34,1	54,3859649	68,8405797	50	74,691358	76,388889	70,17544
3	24,603175	39,166667	57,1428571	45,4545455	45,0617284	49,1666667	30,952381	21,6		32,6	68,4210526	50	62,962963	68,5185185	59,027778	
4	33,333333	40,833333	58,3333333	50	40,7407407	48,3333333	36,5079365									
5	17,460318	35,833333	53,5714286	56,8181818	46,2962963	45,8333333	42,8571429									
6			30,952381	51,5151515	48,7654321	54,1666667	45,2380952									
7				53,7878788	37,654321	36,6666667	44,4444444									
8				43,9393939		49,1666667	31,7460317									

Detailed Description of the Average of Progressive Sites (with increase in CAL ≥ 2mm)for Each Monitoring Time

Monitoring session	A	C	D	E	F	G	H	J	K	L	N	O	Q	R	S	T
0																
1	0	0,8333333	2,38095238	4,54545455	0	1,66666667	0,79365079	4,9	3,96825397	2,2	0	0	0	0	0	0
2	0	0,8333333	2	4,54545455	0	1,66666667	0,79365079	4,9	3,17460317	2,9	0	0	0	0,61728395	0	3,508772
3	6,3492064	1,6666667	2,38095238	3,78787879	3,7037037	1,66666667	3,96825397	3,9		3,6	0	9,42028986	3,7037037	5,55555556	9,7222222	
4	3,968254	4,1666667	3,57142857	9,09090909	6,79012346	1,66666667	0,79365079									
5	4,7619048	6,6666667	1,78571429	3,78787879	6,17283951	1,66666667	0,79365079									
6	1,5873016	5	1,78571429	4,54545455	2,4691358	0,83333333	1,58730159									
7			5,95238095	7,57575758	4,32098765	2,5	3,17460317									
8				5,3030303	4,32098765	5,83333333	1,58730159									

Detailed Description of the Average of Sites with gaining in CAL (Regressing sites) for Each Monitoring Time

Monitoring session	A	C	D	E	F	G	H	J	K	L	N	O	Q	R	S	T
0																
1	0	-5,8333333	-4	-0,7575758	0	-3,3333333	0			-8,7	-7,8947368	-0,7246377	-1,8518519	-0,617284		
2	-4,5238095	-9,1666667	-1,7857143	-2,2727273	-1,8518519	-3,3333333	-2,5555556			-9,4	-3,5087719	-1,4492754	-5,5555556	0		
3	-6,4920635	-8,3333333	-1,1904762	-0,7575758	-1,2345679	-4,1666667	-4,7301587			-11,6	-0,877193	-1,4492754	0	-0,617284		
4	-7,0793651	-8,3333333	-1,1904762	-2,2727273	-3,0864198	-3,3333333	-6,6984127			0	0	0	0	0		
5			-1,7857143	-1,5151515	-2,4691358	-7,5	-5,3174603									
6			-7,7380952	-2,2727273	-3,7037037	-3,3333333	-4,7301587									
7				-3,030303	-4,3209877	-6,6666667	-3,9365079									
8					-5	-2,5	-3,5555556									

(*) Number of teeth x 6 sites

(**) Number of sampled sites * numer of follow-up sessions

Supplementary table 2. The complete proteomic profile derived from the GCF samples in NP and PG groups

Checked	Protein FDR Confidence: Combined	Master	Accession	Description	Gene Symbol	MW [kDa]	Abundance Ratio: (Progressive) / (Non-progresive)	Adj. P-Value: (Progressive) / (Non progressive)
FALSO	Medium	Master Protein	Q8WVM8	Sec1 family domain-containing protein 1 OS=Homo sapiens (Human) OX=9606 GN=SCFD1 PE=1 SV=4	SCFD1	72,3	#¡DIV/0!	0,55
FALSO	High	Master Protein	P13498	Cytochrome b-245 light chain OS=Homo sapiens (Human) OX=9606 GN=CYBA PE=1 SV=3	CYBA	21	#¡DIV/0!	0,6
FALSO	High	Master Protein	Q6UWP8	Suprabasin OS=Homo sapiens (Human) OX=9606 GN=SBSN PE=1 SV=2	SBSN	60,5	#¡DIV/0!	#¡DIV/0!
FALSO	High	None	Q9UKX3	Myosin-13 OS=Homo sapiens (Human) OX=9606 GN=MYH13 PE=2 SV=2	MYH13	224	#¡DIV/0!	#¡DIV/0!
FALSO	Medium	Master Protein	P11586	C-1-tetrahydrofolate synthase, cytoplasmic OS=Homo sapiens (Human) OX=9606 GN=MTHFD1 PE=1 SV=3	MTHFD1	102	#¡DIV/0!	#¡DIV/0!
FALSO	High	None	Q14315	Filamin-C OS=Homo sapiens (Human) OX=9606 GN=FLNC PE=1 SV=3	FLNC	291	#¡DIV/0!	#¡DIV/0!
FALSO	High	Master Protein	P04746	Pancreatic alpha-amylase OS=Homo sapiens (Human) OX=9606 GN=AMY2A PE=1 SV=2	AMY2A	57,7	#¡DIV/0!	#¡DIV/0!
FALSO	High	None	A6NEC2	Puromycin-sensitive aminopeptidase-like protein OS=Homo sapiens (Human) OX=9606 GN=NPEPPSL1 PE=2 S	LOC440434	53,7	380,61	0,000216982
FALSO	High	Master Protein	P55786	Puromycin-sensitive aminopeptidase OS=Homo sapiens (Human) OX=9606 GN=NPEPPS PE=1 SV=2	NPEPPS	103	131,62	0,000136793
FALSO	High	Master Protein	Q9Y2T2	AP-3 complex subunit mu-1 OS=Homo sapiens (Human) OX=9606 GN=AP3M1 PE=1 SV=1	AP3M1	46,9	Exclusive in PG	3,20536E-16
FALSO	High	None	P14410	Sucrase-isomaltase, intestinal OS=Homo sapiens (Human) OX=9606 GN=SI PE=1 SV=6	SI	209	Exclusive in PG	3,20536E-16
FALSO	High	Master Protein	P30047	GTP cyclohydrolase 1 feedback regulatory protein OS=Homo sapiens (Human) OX=9606 GN=GCHFR PE=1 SV=	GCHFR	9,7	Exclusive in PG	3,20536E-16
FALSO	Medium	Master Protein	Q8NBJ7	Inactive C-alpha-formylglycine-generating enzyme 2 OS=Homo sapiens (Human) OX=9606 GN=SUMF2 PE=1 S	SUMF2	33,8	Exclusive in PG	3,20536E-16
FALSO	High	Master Protein	P02008	Hemoglobin subunit zeta OS=Homo sapiens (Human) OX=9606 GN=HBZ PE=1 SV=2	HBZ	15,6	Exclusive in PG	3,20536E-16
FALSO	High	Master Protein	P37840	Alpha-synuclein OS=Homo sapiens (Human) OX=9606 GN=SNCA PE=1 SV=1	SNCA	14,5	Exclusive in PG	3,20536E-16
FALSO	Medium	Master Protein	P22735	Protein-glutamine gamma-glutamyltransferase K OS=Homo sapiens (Human) OX=9606 GN=TGM1 PE=1 SV=4	TGM1	89,7	Exclusive in PG	3,20536E-16
FALSO	High	Master Protein	Q15102	Platelet-activating factor acetylhydrolase IB subunit gamma OS=Homo sapiens (Human) OX=9606 GN=PAFAH	PAFAH1B3	25,7	Exclusive in PG	3,20536E-16
FALSO	High	Master Protein	P35241	Radixin OS=Homo sapiens (Human) OX=9606 GN=RDX PE=1 SV=1	RDX	68,5	Exclusive in PG	3,20536E-16
FALSO	Medium	Master Protein	Q9UHD8	Septin-9 OS=Homo sapiens (Human) OX=9606 GN=SEPTIN9 PE=1 SV=2	SEPT9	65,4	Exclusive in PG	3,20536E-16
FALSO	High	Master Protein	P63208	S-phase kinase-associated protein 1 OS=Homo sapiens (Human) OX=9606 GN=SKP1 PE=1 SV=2	SKP1	18,6	Exclusive in PG	3,20536E-16
FALSO	High	Master Protein	P0DP01	Immunoglobulin heavy variable 1-8 OS=Homo sapiens (Human) OX=9606 GN=IGHV1-8 PE=3 SV=1	IGHV1-8	13	Exclusive in PG	3,20536E-16
FALSO	High	Master Protein	P08397	Porphobilinogen deaminase OS=Homo sapiens (Human) OX=9606 GN=HMBS PE=1 SV=2	HMBS	39,3	Exclusive in PG	3,20536E-16
FALSO	High	Master Protein	Q92930	Ras-related protein Rab-8B OS=Homo sapiens (Human) OX=9606 GN=RAB8B PE=1 SV=2	RAB8B	23,6	Exclusive in PG	3,20536E-16
FALSO	High	None	A0A075B6Q5	Immunoglobulin heavy variable 3-64 OS=Homo sapiens (Human) OX=9606 GN=IGHV3-64 PE=3 SV=1	LOC100291056; I	12,9	Exclusive in PG	3,20536E-16
FALSO	High	Master Protein	O95394	Phosphoacetylglucosamine mutase OS=Homo sapiens (Human) OX=9606 GN=PGM3 PE=1 SV=1	PGM3	59,8	Exclusive in PG	3,20536E-16
FALSO	Medium	None	Q9HAU6	Putative translationally-controlled tumor protein-like protein TPT1P8 OS=Homo sapiens (Human) OX=9606 G	FKSG2; TPT1P8	16	Exclusive in PG	3,20536E-16
FALSO	Medium	Master Protein	P54257	Huntingtin-associated protein 1 OS=Homo sapiens (Human) OX=9606 GN=HAP1 PE=1 SV=3	HAP1	75,5	Exclusive in PG	3,592E-16
FALSO	High	Master Protein	P54652	Heat shock-related 70 kDa protein 2 OS=Homo sapiens (Human) OX=9606 GN=HSPA2 PE=1 SV=1	HSPA2	70	Exclusive in PG	3,592E-16
FALSO	High	Master Protein	P00167	Cytochrome b5 OS=Homo sapiens (Human) OX=9606 GN=CYB5A PE=1 SV=2	CYB5A	15,3	Exclusive in PG	0,713015717

FALSO	High	Master Protein	Q14254	Flotillin-2 OS=Homo sapiens (Human) OX=9606 GN=FLOT2 PE=1 SV=2	FLOT2	47	Exclusive in PG	0,992641842
FALSO	High	Master Protein	Q9UK76	Jupiter microtubule associated homolog 1 OS=Homo sapiens (Human) OX=9606 GN=JPT1 PE=1 SV=3	HN1; JPT1	16	Exclusive in PG	0,992641842
FALSO	High	Master Protein	P69892	Hemoglobin subunit gamma-2 OS=Homo sapiens (Human) OX=9606 GN=HBG2 PE=1 SV=2	HBG2	16,1	33,22	0,405540809
FALSO	High	Master Protein	P13798	Acylamino-acid-releasing enzyme OS=Homo sapiens (Human) OX=9606 GN=APEH PE=1 SV=4	APEH	81,2	12,60	0,003836921
FALSO	High	Master Protein	P69891	Hemoglobin subunit gamma-1 OS=Homo sapiens (Human) OX=9606 GN=HBG1 PE=1 SV=2	HBG1	16,1	9,44	0,09076121
FALSO	Medium	Master Protein Candidat	Q9P286	Serine/threonine-protein kinase PAK 5 OS=Homo sapiens (Human) OX=9606 GN=PAK5 PE=1 SV=1	PAK5	80,7	8,08	0,077528934
FALSO	Medium	Master Protein	O96013	Serine/threonine-protein kinase PAK 4 OS=Homo sapiens (Human) OX=9606 GN=PAK4 PE=1 SV=1	PAK4	64	8,08	0,077528934
FALSO	High	None	Q9BVA1	Tubulin beta-2B chain OS=Homo sapiens (Human) OX=9606 GN=TUBB2B PE=1 SV=1	TUBB2B	49,9	8,06	0,000173317
FALSO	High	None	Q13885	Tubulin beta-2A chain OS=Homo sapiens (Human) OX=9606 GN=TUBB2A PE=1 SV=1	TUBB2A	49,9	8,06	0,000173317
FALSO	Medium	Master Protein	Q9NQX4	Unconventional myosin-Vc OS=Homo sapiens (Human) OX=9606 GN=MYO5C PE=1 SV=2	MYO5C	203	6,71	0,237494617
FALSO	High	Master Protein	P02730	Band 3 anion transport protein OS=Homo sapiens (Human) OX=9606 GN=SLC4A1 PE=1 SV=3	SLC4A1	102	6,51	0,000103497
FALSO	High	Master Protein	P07437	Tubulin beta chain OS=Homo sapiens (Human) OX=9606 GN=TUBB PE=1 SV=2	TUBB	49,6	5,45	0,001787645
FALSO	Medium	Master Protein	P00742	Coagulation factor X OS=Homo sapiens (Human) OX=9606 GN=F10 PE=1 SV=2	F10	54,7	5,17	0,003366597
FALSO	High	Master Protein	P02808	Statherin OS=Homo sapiens (Human) OX=9606 GN=STATH PE=1 SV=2	STATH	7,3	4,77	0,384791715
FALSO	High	Master Protein	P34096	Ribonuclease 4 OS=Homo sapiens (Human) OX=9606 GN=RNASE4 PE=1 SV=3	RNASE4	16,8	4,46	0,209700545
FALSO	Medium	Master Protein	Q14789	Golgin subfamily B member 1 OS=Homo sapiens (Human) OX=9606 GN=GOLGB1 PE=1 SV=2	GOLGB1	376	4,28	0,039782458
FALSO	Medium	Master Protein	Q5T750	Skin-specific protein 32 OS=Homo sapiens (Human) OX=9606 GN=XP32 PE=1 SV=1	C1orf68	26,2	4,15	0,130634522
FALSO	High	Master Protein	P00492	Hypoxanthine-guanine phosphoribosyltransferase OS=Homo sapiens (Human) OX=9606 GN=HPRT1 PE=1 SV	HPRT1	24,6	4,09	0,184163861
FALSO	High	Master Protein	P16157	Ankyrin-1 OS=Homo sapiens (Human) OX=9606 GN=ANK1 PE=1 SV=3	ANK1	206	3,94	0,020043812
FALSO	High	Master Protein	P07738	Bisphosphoglycerate mutase OS=Homo sapiens (Human) OX=9606 GN=BPGM PE=1 SV=2	BPGM	30	3,83	0,006357824
FALSO	High	Master Protein	P00918	Carbonic anhydrase 2 OS=Homo sapiens (Human) OX=9606 GN=CA2 PE=1 SV=2	CA2	29,2	3,66	0,014933527
FALSO	High	Master Protein	P68871	Hemoglobin subunit beta OS=Homo sapiens (Human) OX=9606 GN=HBB PE=1 SV=2	HBB	16	3,54	0,00444599
FALSO	High	Master Protein	P69905	Hemoglobin subunit alpha OS=Homo sapiens (Human) OX=9606 GN=HBA2 PE=1 SV=2	HBA2	15,2	3,47	0,001085773
FALSO	High	Master Protein	P02549	Spectrin alpha chain, erythrocytic 1 OS=Homo sapiens (Human) OX=9606 GN=SPTA1 PE=1 SV=5	SPTA1	280	3,45	0,074696202
FALSO	High	None	Q9P0M6	Core histone macro-H2A.2 OS=Homo sapiens (Human) OX=9606 GN=H2AFY2 PE=1 SV=3	H2AFY2	40	3,39	0,00294123
FALSO	High	Master Protein	P32119	Peroxiredoxin-2 OS=Homo sapiens (Human) OX=9606 GN=PRDX2 PE=1 SV=5	PRDX2	21,9	3,39	0,031709353
FALSO	High	Master Protein	P00352	Retinal dehydrogenase 1 OS=Homo sapiens (Human) OX=9606 GN=ALDH1A1 PE=1 SV=2	ALDH1A1	54,8	3,28	0,001910658
FALSO	High	None	A6NHG4	D-dopachrome decarboxylase-like protein OS=Homo sapiens (Human) OX=9606 GN=DDTL PE=2 SV=1	DDTL	14,2	3,27	0,016541409
FALSO	High	Master Protein	P30046	D-dopachrome decarboxylase OS=Homo sapiens (Human) OX=9606 GN=DDT PE=1 SV=3	DDT	12,7	3,21	0,014727892
FALSO	High	Master Protein	P28289	Tropomodulin-1 OS=Homo sapiens (Human) OX=9606 GN=TMOD1 PE=1 SV=1	TMOD1	40,5	3,11	0,033220505
FALSO	High	Master Protein	P00915	Carbonic anhydrase 1 OS=Homo sapiens (Human) OX=9606 GN=CA1 PE=1 SV=2	CA1	28,9	3,06	0,02823759
FALSO	High	Master Protein	Q07960	Rho GTPase-activating protein 1 OS=Homo sapiens (Human) OX=9606 GN=ARHGAP1 PE=1 SV=1	ARHGAP1	50,4	3,02	0,00173784
FALSO	High	Master Protein	P30043	Flavin reductase (NADPH) OS=Homo sapiens (Human) OX=9606 GN=BLVRB PE=1 SV=3	BLVRB	22,1	2,95	0,013960585

FALSO	High	Master Protein	Q08554	Desmocollin-1 OS=Homo sapiens (Human) OX=9606 GN=DSC1 PE=1 SV=2	DSC1	99,9	2,90	0,106146001
FALSO	Medium	Master Protein	A8MTA8	Protein FAM166B OS=Homo sapiens (Human) OX=9606 GN=FAM166B PE=1 SV=1	FAM166B	30,6	2,82	0,047001828
FALSO	High	Master Protein	Q13287	N-myc-interactor OS=Homo sapiens (Human) OX=9606 GN=NMI PE=1 SV=2	NMI	35	2,79	0,000282178
FALSO	High	Master Protein	Q9UHL4	Dipeptidyl peptidase 2 OS=Homo sapiens (Human) OX=9606 GN=DPP7 PE=1 SV=3	DPP7	54,3	2,73	0,000137949
FALSO	High	Master Protein	P28325	Cystatin-D OS=Homo sapiens (Human) OX=9606 GN=CST5 PE=1 SV=1	CST5	16,1	2,61	0,173018466
FALSO	High	Master Protein	O15116	U6 snRNA-associated Sm-like protein LSm1 OS=Homo sapiens (Human) OX=9606 GN=LSM1 PE=1 SV=1	LSM1	15,2	2,47	0,372118205
FALSO	High	Master Protein	Q16775	Hydroxyacylglutathione hydrolase, mitochondrial OS=Homo sapiens (Human) OX=9606 GN=HAGH PE=1 SV=2	HAGH	33,8	2,47	0,002860703
FALSO	High	Master Protein	P02042	Hemoglobin subunit delta OS=Homo sapiens (Human) OX=9606 GN=HBD PE=1 SV=2	HBD	16	2,42	0,067685813
FALSO	Medium	Master Protein	Q8IUG5	Unconventional myosin-XVIIIb OS=Homo sapiens (Human) OX=9606 GN=MYO18B PE=1 SV=2	MYO18B	285	2,35	0,011523942
FALSO	High	None	A6NNZ2	Tubulin beta 8B OS=Homo sapiens (Human) OX=9606 GN=TUBB8B PE=1 SV=1	TUBB8B	49,5	2,34	0,44475693
FALSO	High	Master Protein	Q96IY4	Carboxypeptidase B2 OS=Homo sapiens (Human) OX=9606 GN=CPB2 PE=1 SV=2	CPB2	48,4	2,24	0,279697714
FALSO	High	Master Protein	P03951	Coagulation factor XI OS=Homo sapiens (Human) OX=9606 GN=F11 PE=1 SV=1	F11	70,1	2,21	0,030664092
FALSO	High	None	Q5VVH2	Peptidylprolyl isomerase OS=Homo sapiens (Human) OX=9606 GN=FKBP1C PE=1 SV=1	FKBP1C	12,2	2,17	0,023431428
FALSO	High	Master Protein	P42224	Signal transducer and activator of transcription 1-alpha/beta OS=Homo sapiens (Human) OX=9606 GN=STAT1	STAT1	87,3	2,16	0,145478968
FALSO	High	Master Protein	Q12805	EGF-containing fibulin-like extracellular matrix protein 1 OS=Homo sapiens (Human) OX=9606 GN=EFEMP1 P	EFEMP1	54,6	2,16	0,249339811
FALSO	High	Master Protein	P61970	Nuclear transport factor 2 OS=Homo sapiens (Human) OX=9606 GN=NUTF2 PE=1 SV=1	NUTF2	14,5	2,12	0,004327971
FALSO	High	Master Protein	P0C0L5	Complement C4-B OS=Homo sapiens (Human) OX=9606 GN=C4B_2 PE=1 SV=2	C4B	193	2,10	0,006050684
FALSO	High	Master Protein	Q99653	Calcineurin B homologous protein 1 OS=Homo sapiens (Human) OX=9606 GN=CHP1 PE=1 SV=3	CHP1	22,4	2,10	0,039588518
FALSO	High	Master Protein	P19878	Neutrophil cytosol factor 2 OS=Homo sapiens (Human) OX=9606 GN=NCF2 PE=1 SV=2	NCF2	59,7	2,09	0,015998252
FALSO	High	Master Protein	P00491	Purine nucleoside phosphorylase OS=Homo sapiens (Human) OX=9606 GN=PNP PE=1 SV=2	PNP	32,1	2,06	0,00042415
FALSO	High	Master Protein	A0A0C4DH33	Immunoglobulin heavy variable 1-24 OS=Homo sapiens (Human) OX=9606 GN=IGHV1-24 PE=3 SV=1	IGHV1-24	12,8	2,05	0,156606526
FALSO	High	Master Protein	P29144	Tripeptidyl-peptidase 2 OS=Homo sapiens (Human) OX=9606 GN=TPP2 PE=1 SV=4	TPP2	138	2,05	0,030893478
FALSO	High	Master Protein	Q9UQ80	Proliferation-associated protein 2G4 OS=Homo sapiens (Human) OX=9606 GN=PA2G4 PE=1 SV=3	PA2G4	43,8	2,04	0,020965606
FALSO	High	Master Protein	P11277	Spectrin beta chain, erythrocytic OS=Homo sapiens (Human) OX=9606 GN=SPTB PE=1 SV=5	SPTB	246	2,02	0,079178604
FALSO	High	Master Protein	O95861	3'(2'),5'-bisphosphate nucleotidase 1 OS=Homo sapiens (Human) OX=9606 GN=BPNT1 PE=1 SV=1	BPNT1	33,4	2,01	0,033453274
FALSO	Medium	Master Protein	Q8N5Y2	Male-specific lethal 3 homolog OS=Homo sapiens (Human) OX=9606 GN=MSL3 PE=1 SV=1	MSL3	59,8	1,99	0,009834796
FALSO	High	Master Protein	O75923	Dysferlin OS=Homo sapiens (Human) OX=9606 GN=DYSF PE=1 SV=1	DYSF	237	1,99	0,003091672
FALSO	Medium	Master Protein	P03950	Angiogenin OS=Homo sapiens (Human) OX=9606 GN=ANG PE=1 SV=1	ANG	16,5	1,99	0,164308721
FALSO	Medium	Master Protein	P29353	SHC-transforming protein 1 OS=Homo sapiens (Human) OX=9606 GN=SHC1 PE=1 SV=4	SHC1	62,8	1,96	0,18673658
FALSO	High	Master Protein	P62942	Peptidyl-prolyl cis-trans isomerase FKBP1A OS=Homo sapiens (Human) OX=9606 GN=FKBP1A PE=1 SV=2	FKBP1A	11,9	1,95	0,05343743
FALSO	High	Master Protein	P04406	Glyceraldehyde-3-phosphate dehydrogenase OS=Homo sapiens (Human) OX=9606 GN=GAPDH PE=1 SV=3	GAPDH	36	1,93	0,005900348
FALSO	High	Master Protein	P31939	Bifunctional purine biosynthesis protein PURH OS=Homo sapiens (Human) OX=9606 GN=ATIC PE=1 SV=3	ATIC	64,6	1,93	3,89599E-06
FALSO	High	Master Protein	P19823	Inter-alpha-trypsin inhibitor heavy chain H2 OS=Homo sapiens (Human) OX=9606 GN=ITIH2 PE=1 SV=2	ITIH2	106	1,92	0,048495056

FALSO	Medium	Master Protein	Q14444	Caprin-1 OS=Homo sapiens (Human) OX=9606 GN=CAPRIN1 PE=1 SV=2	CAPRIN1	78,3	1,92	0,000459327
FALSO	High	Master Protein	Q6S8J3	POTE ankyrin domain family member E OS=Homo sapiens (Human) OX=9606 GN=POTEE PE=2 SV=3	POTEE	121	1,91	0,26570863
FALSO	High	None	A5A3E0	POTE ankyrin domain family member F OS=Homo sapiens (Human) OX=9606 GN=POTEF PE=1 SV=2	POTEF	121	1,91	0,26570863
FALSO	High	Master Protein	Q58FF6	Putative heat shock protein HSP 90-beta 4 OS=Homo sapiens (Human) OX=9606 GN=HSP90AB4P PE=5 SV=1	HSP90AB4P	58,2	1,89	0,019438013
FALSO	High	Master Protein	A0A087WW49	Ig-like domain-containing protein OS=Homo sapiens (Human) OX=9606 GN=ENSG00000278263 PE=4 SV=1	ENSG0000027826	12,9	1,88	0,024028802
FALSO	High	Master Protein Candidat	A0A075B6R2	Immunoglobulin heavy variable 4-4 OS=Homo sapiens (Human) OX=9606 GN=IGHV4-4 PE=3 SV=2	IGHV4-4	12,8	1,88	0,024028802
FALSO	Medium	None	A0A075B7B6	Ig-like domain-containing protein OS=Homo sapiens (Human) OX=9606 GN=IGHV4OR15-8 PE=4 SV=1	IGHV4OR15	13	1,88	0,024028802
FALSO	High	Master Protein	Q8IUE6	Histone H2A type 2-B OS=Homo sapiens (Human) OX=9606 GN=HIST2H2AB PE=1 SV=3	HIST2H2AB	14	1,87	0,055908256
FALSO	High	Master Protein	P02647	Apolipoprotein A-I OS=Homo sapiens (Human) OX=9606 GN=APOA1 PE=1 SV=1	APOA1	30,8	1,86	0,004132825
FALSO	High	Master Protein	P15814	Immunoglobulin lambda-like polypeptide 1 OS=Homo sapiens (Human) OX=9606 GN=IGLL1 PE=1 SV=1	IGLL1	22,9	1,86	0,213516943
FALSO	High	Master Protein Candidat	Q6DRA6	Putative histone H2B type 2-D OS=Homo sapiens (Human) OX=9606 GN=HIST2H2BD PE=5 SV=3	HIST2H2BD	18	1,84	0,014016979
FALSO	High	Master Protein	Q6DN03	Putative histone H2B type 2-C OS=Homo sapiens (Human) OX=9606 GN=HIST2H2BC PE=5 SV=3	HIST2H2BC	21,5	1,84	0,014016979
FALSO	Medium	Master Protein	A0A0A0MT69	Immunoglobulin kappa joining 4 OS=Homo sapiens (Human) OX=9606 GN=IGKJ4 PE=1 SV=1	IGKJ4	1,2	1,84	0,030404545
FALSO	High	Master Protein	Q9UJ68	Mitochondrial peptide methionine sulfoxide reductase OS=Homo sapiens (Human) OX=9606 GN=MSRA PE=1	MSRA	26,1	1,84	0,03459934
FALSO	High	None	Q8IZP2	Putative protein FAM10A4 OS=Homo sapiens (Human) OX=9606 GN=ST13P4 PE=5 SV=1	ST13P4	27,4	1,82	0,038543889
FALSO	High	Master Protein	P36969	Phospholipid hydroperoxide glutathione peroxidase OS=Homo sapiens (Human) OX=9606 GN=GPX4 PE=1 S\	GPX4	22,2	1,82	0,071039133
FALSO	High	Master Protein	Q01130	Serine/arginine-rich splicing factor 2 OS=Homo sapiens (Human) OX=9606 GN=SRSF2 PE=1 SV=4	SRSF2	25,5	1,82	0,317392093
FALSO	Medium	Master Protein	Q6PF15	Kelch-like protein 35 OS=Homo sapiens (Human) OX=9606 GN=KLHL35 PE=1 SV=3	KLHL35	62,9	1,81	0,100049004
FALSO	Medium	Master Protein	Q5VWK5	Interleukin-23 receptor OS=Homo sapiens (Human) OX=9606 GN=IL23R PE=1 SV=3	IL23R	71,7	1,80	0,037933477
FALSO	High	Master Protein	Q86UX7	Fermitin family homolog 3 OS=Homo sapiens (Human) OX=9606 GN=FERMT3 PE=1 SV=1	FERMT3	75,9	1,79	0,038721208
FALSO	High	Master Protein	P18669	Phosphoglycerate mutase 1 OS=Homo sapiens (Human) OX=9606 GN=PGAM1 PE=1 SV=2	PGAM1	28,8	1,79	0,04666212
FALSO	Medium	Master Protein	Q9Y2K3	Myosin-15 OS=Homo sapiens (Human) OX=9606 GN=MYH15 PE=1 SV=5	MYH15	225	1,79	0,32674965
FALSO	High	Master Protein	Q92608	Dedicator of cytokinesis protein 2 OS=Homo sapiens (Human) OX=9606 GN=DOCK2 PE=1 SV=2	DOCK2	212	1,76	0,012377816
FALSO	High	Master Protein	Q9NZD4	Alpha-hemoglobin-stabilizing protein OS=Homo sapiens (Human) OX=9606 GN=AHSP PE=1 SV=1	AHSP	11,8	1,76	0,297074644
FALSO	Medium	Master Protein	Q8N3L3	Beta-taxilin OS=Homo sapiens (Human) OX=9606 GN=TXLNB PE=1 SV=3	TXLNB	76,5	1,76	0,035874076
FALSO	High	None	A0A0J9YWU9	Ig-like domain-containing protein OS=Homo sapiens (Human) OX=9606 GN=ENSG00000278082 PE=1 SV=1	ENSG0000027808	12,9	1,75	0,058979056
FALSO	High	Master Protein	Q9UBW5	Bridging integrator 2 OS=Homo sapiens (Human) OX=9606 GN=BIN2 PE=1 SV=3	BIN2	61,8	1,75	0,039846349
FALSO	High	Master Protein	P62333	26S proteasome regulatory subunit 10B OS=Homo sapiens (Human) OX=9606 GN=PSMC6 PE=1 SV=1	PSMC6	44,1	1,75	0,306888052
FALSO	Medium	Master Protein	P51795	H(+)/Cl(-) exchange transporter 5 OS=Homo sapiens (Human) OX=9606 GN=CLCN5 PE=1 SV=1	CLCN5	83,1	1,74	0,183189217
FALSO	High	None	Q9H254	Spectrin beta chain, non-erythrocytic 4 OS=Homo sapiens (Human) OX=9606 GN=SPTBN4 PE=1 SV=2	SPTBN4	289	1,73	0,024513173
FALSO	High	None	P62745	Rho-related GTP-binding protein RhoB OS=Homo sapiens (Human) OX=9606 GN=RHOB PE=1 SV=1	RHOB	22,1	1,72	0,018870139
FALSO	High	Master Protein	Q5TDH0	Protein DDI1 homolog 2 OS=Homo sapiens (Human) OX=9606 GN=DDI2 PE=1 SV=1	DDI2	44,5	1,71	0,165450576
FALSO	High	Master Protein	P01817	Immunoglobulin heavy variable 2-5 OS=Homo sapiens (Human) OX=9606 GN=IGHV2-5 PE=1 SV=2	IGHV2-5	13,2	1,71	#¡DIV/0!

FALSO	High	Master Protein	Q9BRF8	Serine/threonine-protein phosphatase CPPED1 OS=Homo sapiens (Human) OX=9606 GN=CPPED1 PE=1 SV=3	CPPED1	35,5	1,71	0,072413476
FALSO	High	Master Protein	P46926	Glucosamine-6-phosphate isomerase 1 OS=Homo sapiens (Human) OX=9606 GN=GNPDA1 PE=1 SV=1	GNPDA1	32,6	1,70	0,026135041
FALSO	High	Master Protein	Q96HC4	PDZ and LIM domain protein 5 OS=Homo sapiens (Human) OX=9606 GN=PDLIM5 PE=1 SV=5	PDLIM5	63,9	1,70	0,153836555
FALSO	High	None	Q8N0Y7	Probable phosphoglycerate mutase 4 OS=Homo sapiens (Human) OX=9606 GN=PGAM4 PE=3 SV=1	PGAM4	28,8	1,69	0,078234695
FALSO	Medium	Master Protein	O75436	Vacuolar protein sorting-associated protein 26A OS=Homo sapiens (Human) OX=9606 GN=VPS26A PE=1 SV=1	VPS26A	38,1	1,68	0,030393742
FALSO	High	Master Protein	Q9BWD1	Acetyl-CoA acetyltransferase, cytosolic OS=Homo sapiens (Human) OX=9606 GN=ACAT2 PE=1 SV=2	ACAT2	41,3	1,67	0,108111763
FALSO	High	Master Protein	P30041	Peroxiredoxin-6 OS=Homo sapiens (Human) OX=9606 GN=PRDX6 PE=1 SV=3	PRDX6	25	1,67	0,041442858
FALSO	High	Master Protein Candidat	Q9H8S9	MOB kinase activator 1A OS=Homo sapiens (Human) OX=9606 GN=MOB1A PE=1 SV=4	MOB1A	25,1	1,66	0,300375356
FALSO	High	Master Protein	Q7L9L4	MOB kinase activator 1B OS=Homo sapiens (Human) OX=9606 GN=MOB1B PE=1 SV=3	MOB1B	25,1	1,66	0,300375356
FALSO	High	Master Protein	P22061	Protein-L-isoaspartate(D-aspartate) O-methyltransferase OS=Homo sapiens (Human) OX=9606 GN=PCMT1 P	PCMT1	24,6	1,66	0,00556318
FALSO	High	Master Protein	P50502	Hsc70-interacting protein OS=Homo sapiens (Human) OX=9606 GN=ST13 PE=1 SV=2	ST13	41,3	1,66	0,028924061
FALSO	High	Master Protein	P05386	60S acidic ribosomal protein P1 OS=Homo sapiens (Human) OX=9606 GN=RPLP1 PE=1 SV=1	RPLP1	11,5	1,65	0,010078375
FALSO	High	Master Protein	P68036	Ubiquitin-conjugating enzyme E2 L3 OS=Homo sapiens (Human) OX=9606 GN=UBE2L3 PE=1 SV=1	UBE2L3	17,9	1,65	0,140403322
FALSO	High	Master Protein	Q15181	Inorganic pyrophosphatase OS=Homo sapiens (Human) OX=9606 GN=PPA1 PE=1 SV=2	PPA1	32,6	1,65	0,200220495
FALSO	High	Master Protein	P60174	Triosephosphate isomerase OS=Homo sapiens (Human) OX=9606 GN=TPI1 PE=1 SV=3	TPI1	30,8	1,65	0,002591394
FALSO	High	Master Protein	Q92820	Gamma-glutamyl hydrolase OS=Homo sapiens (Human) OX=9606 GN=GGH PE=1 SV=2	GGH	35,9	1,64	0,034911834
FALSO	High	Master Protein	P49247	Ribose-5-phosphate isomerase OS=Homo sapiens (Human) OX=9606 GN=RPIA PE=1 SV=3	RPIA	33,2	1,64	0,325308384
FALSO	Medium	Master Protein	Q9P1W9	Serine/threonine-protein kinase pim-2 OS=Homo sapiens (Human) OX=9606 GN=PIM2 PE=1 SV=1	PIM2	34,2	1,63	#¡DIV/0!
FALSO	High	Master Protein	Q15404	Ras suppressor protein 1 OS=Homo sapiens (Human) OX=9606 GN=RSU1 PE=1 SV=3	RSU1	31,5	1,62	0,128532967
FALSO	High	Master Protein	O60701	UDP-glucose 6-dehydrogenase OS=Homo sapiens (Human) OX=9606 GN=UGDH PE=1 SV=1	UGDH	55	1,62	0,36566231
FALSO	High	Master Protein	Q9UL46	Proteasome activator complex subunit 2 OS=Homo sapiens (Human) OX=9606 GN=PSME2 PE=1 SV=4	PSME2	27,4	1,61	0,121362809
FALSO	High	Master Protein	Q9H9S4	Calcium-binding protein 39-like OS=Homo sapiens (Human) OX=9606 GN=CAB39L PE=1 SV=3	CAB39L	39,1	1,61	0,27943637
FALSO	High	Master Protein	P61077	Ubiquitin-conjugating enzyme E2 D3 OS=Homo sapiens (Human) OX=9606 GN=UBE2D3 PE=1 SV=1	UBE2D3	16,7	1,61	0,087405668
FALSO	High	Master Protein Candidat	P62837	Ubiquitin-conjugating enzyme E2 D2 OS=Homo sapiens (Human) OX=9606 GN=UBE2D2 PE=1 SV=1	UBE2D2	16,7	1,61	0,087405668
FALSO	High	Master Protein	Q92954	Proteoglycan 4 OS=Homo sapiens (Human) OX=9606 GN=PRG4 PE=1 SV=3	PRG4	151	1,61	0,401669751
FALSO	High	Master Protein	P09758	Tumor-associated calcium signal transducer 2 OS=Homo sapiens (Human) OX=9606 GN=TACSTD2 PE=1 SV=3	TACSTD2	35,7	1,61	0,409805556
FALSO	High	Master Protein	P04114	Apolipoprotein B-100 OS=Homo sapiens (Human) OX=9606 GN=APOB PE=1 SV=2	APOB	515	1,60	0,064197869
FALSO	High	Master Protein	P35858	Insulin-like growth factor-binding protein complex acid labile subunit OS=Homo sapiens (Human) OX=9606 G	IGFALS	66	1,60	0,084199266
FALSO	Medium	Master Protein Candidat	Q86YV6	Myosin light chain kinase family member 4 OS=Homo sapiens (Human) OX=9606 GN=MYLK4 PE=1 SV=2	MYLK4	44,5	1,60	0,046306164
FALSO	Medium	Master Protein Candidat	Q9H1R3	Myosin light chain kinase 2, skeletal/cardiac muscle OS=Homo sapiens (Human) OX=9606 GN=MYLK2 PE=1 S\	MYLK2	64,6	1,60	0,046306164
FALSO	Medium	Master Protein	Q32MK0	Myosin light chain kinase 3 OS=Homo sapiens (Human) OX=9606 GN=MYLK3 PE=1 SV=3	MYLK3	88,3	1,60	0,046306164
FALSO	High	Master Protein	Q9HDC9	Adipocyte plasma membrane-associated protein OS=Homo sapiens (Human) OX=9606 GN=APMAP PE=1 SV=2	APMAP	46,5	1,60	0,363620441
FALSO	High	Master Protein	P62308	Small nuclear ribonucleoprotein G OS=Homo sapiens (Human) OX=9606 GN=SNRPG PE=1 SV=1	SNRPG	8,5	1,59	0,017144972

FALSO	High	Master Protein Candidat	A8MWD9	Putative small nuclear ribonucleoprotein G-like protein 15 OS=Homo sapiens (Human) OX=9606 GN=SNRPGF	SNRPGP15	8,5	1,59	0,017144972
FALSO	High	Master Protein	Q9BR76	Coronin-1B OS=Homo sapiens (Human) OX=9606 GN=CORO1B PE=1 SV=1	CORO1B	54,2	1,58	0,477391539
FALSO	Medium	Master Protein	Q15413	Ryanodine receptor 3 OS=Homo sapiens (Human) OX=9606 GN=RYR3 PE=1 SV=3	RYR3	552	1,58	0,116426234
FALSO	High	Master Protein	P43405	Tyrosine-protein kinase SYK OS=Homo sapiens (Human) OX=9606 GN=SYK PE=1 SV=1	SYK	72	1,58	0,771509267
FALSO	High	Master Protein	P46109	Crk-like protein OS=Homo sapiens (Human) OX=9606 GN=CRKL PE=1 SV=1	CRKL	33,8	1,58	0,010393898
FALSO	High	Master Protein	P23526	Adenosylhomocysteinase OS=Homo sapiens (Human) OX=9606 GN=AHCY PE=1 SV=4	AHCY	47,7	1,58	0,00997734
FALSO	High	Master Protein	P05387	60S acidic ribosomal protein P2 OS=Homo sapiens (Human) OX=9606 GN=RPLP2 PE=1 SV=1	RPLP2	11,7	1,58	0,034544183
FALSO	High	Master Protein	P61081	NEDD8-conjugating enzyme Ubc12 OS=Homo sapiens (Human) OX=9606 GN=UBE2M PE=1 SV=1	UBE2M	20,9	1,57	0,088954197
FALSO	High	Master Protein	P42785	Lysosomal Pro-X carboxypeptidase OS=Homo sapiens (Human) OX=9606 GN=PRCP PE=1 SV=1	PRCP	55,8	1,56	0,108011502
FALSO	High	Master Protein	O75367	Core histone macro-H2A.1 OS=Homo sapiens (Human) OX=9606 GN=MACROH2A1 PE=1 SV=4	H2AFY	39,6	1,56	0,011414659
FALSO	High	Master Protein	Q16777	Histone H2A type 2-C OS=Homo sapiens (Human) OX=9606 GN=HIST2H2AC PE=1 SV=4	HIST2H2AC	14	1,56	0,035153248
FALSO	High	Master Protein Candidat	Q6FI13	Histone H2A type 2-A OS=Homo sapiens (Human) OX=9606 GN=HIST2H2AA3 PE=1 SV=3	HIST2H2AA4	14,1	1,56	0,035153248
FALSO	High	Master Protein	P12956	X-ray repair cross-complementing protein 6 OS=Homo sapiens (Human) OX=9606 GN=XRCC6 PE=1 SV=2	XRCC6	69,8	1,55	0,183855676
FALSO	High	Master Protein	Q9GZN8	UPF0687 protein C20orf27 OS=Homo sapiens (Human) OX=9606 GN=C20orf27 PE=1 SV=3	C20orf27	19,3	1,55	0,046283686
FALSO	High	Master Protein	Q9Y3I1	F-box only protein 7 OS=Homo sapiens (Human) OX=9606 GN=FBXO7 PE=1 SV=1	FBXO7	58,5	1,54	0,440511243
FALSO	High	None	P63096	Guanine nucleotide-binding protein G(i) subunit alpha-1 OS=Homo sapiens (Human) OX=9606 GN=GNAI1 PE	GNAI1	40,3	1,54	0,296150669
FALSO	High	None	P08754	Guanine nucleotide-binding protein G(i) subunit alpha OS=Homo sapiens (Human) OX=9606 GN=GNAI3 PE=1	GNAI3	40,5	1,54	0,296150669
FALSO	High	Master Protein	P02775	Platelet basic protein OS=Homo sapiens (Human) OX=9606 GN=PPBP PE=1 SV=3	PPBP	13,9	1,54	0,296126506
FALSO	High	None	P48741	Putative heat shock 70 kDa protein 7 OS=Homo sapiens (Human) OX=9606 GN=HSPA7 PE=5 SV=2	HSPA7	40,2	1,53	0,075622893
FALSO	High	Master Protein	P05090	Apolipoprotein D OS=Homo sapiens (Human) OX=9606 GN=APOD PE=1 SV=1	APOD	21,3	1,53	0,00486703
FALSO	High	None	A0A0J9YVY3	Immunoglobulin heavy variable 7-4-1 OS=Homo sapiens (Human) OX=9606 GN=IGHV7-4-1 PE=3 SV=1	IGHV7-4-1	12,8	1,53	0,115804532
FALSO	High	Master Protein	P32456	Guanylate-binding protein 2 OS=Homo sapiens (Human) OX=9606 GN=GBP2 PE=1 SV=3	GBP2	67,2	1,53	0,414984955
FALSO	High	None	P07196	Neurofilament light polypeptide OS=Homo sapiens (Human) OX=9606 GN=NEFL PE=1 SV=3	NEFL	61,5	1,52	0,189317992
FALSO	High	Master Protein	Q13043	Serine/threonine-protein kinase 4 OS=Homo sapiens (Human) OX=9606 GN=STK4 PE=1 SV=2	STK4	55,6	1,52	0,324113829
FALSO	High	Master Protein	P26599	Polypyrimidine tract-binding protein 1 OS=Homo sapiens (Human) OX=9606 GN=PTBP1 PE=1 SV=1	PTBP1	57,2	1,51	0,069161307
FALSO	High	Master Protein	Q13228	Methanethiol oxidase OS=Homo sapiens (Human) OX=9606 GN=SELENBP1 PE=1 SV=2	SELENBP1	52,4	1,51	0,096389281
FALSO	High	Master Protein	P28074	Proteasome subunit beta type-5 OS=Homo sapiens (Human) OX=9606 GN=PSMB5 PE=1 SV=3	PSMB5	28,5	1,51	0,094458861
FALSO	High	Master Protein	Q13630	GDP-L-fucose synthase OS=Homo sapiens (Human) OX=9606 GN=TSTA3 PE=1 SV=1	TSTA3	35,9	1,50	0,196020197
FALSO	High	Master Protein	Q9UGM5	Fetuin-B OS=Homo sapiens (Human) OX=9606 GN=FETUB PE=1 SV=2	FETUB	42	1,50	0,068345526
FALSO	High	Master Protein	P49720	Proteasome subunit beta type-3 OS=Homo sapiens (Human) OX=9606 GN=PSMB3 PE=1 SV=2	PSMB3	22,9	1,50	0,094454361
FALSO	High	Master Protein	P48735	Isocitrate dehydrogenase [NADP], mitochondrial OS=Homo sapiens (Human) OX=9606 GN=IDH2 PE=1 SV=2	IDH2	50,9	1,50	0,175473286
FALSO	High	Master Protein	Q05315	Galectin-10 OS=Homo sapiens (Human) OX=9606 GN=CLC PE=1 SV=3	CLC	16,4	1,50	0,278244419
FALSO	High	Master Protein	P01019	Angiotensinogen OS=Homo sapiens (Human) OX=9606 GN=AGT PE=1 SV=1	AGT	53,1	1,49	0,170660447

FALSO	High	Master Protein	P53041	Serine/threonine-protein phosphatase 5 OS=Homo sapiens (Human) OX=9606 GN=PPP5C PE=1 SV=1	PPP5C	56,8	1,49	0,175835649
FALSO	High	Master Protein	Q8IWT0	Protein archease OS=Homo sapiens (Human) OX=9606 GN=ZBTB8OS PE=1 SV=2	ZBTB8OS	19,5	1,49	0,202859606
FALSO	High	Master Protein	Q9UHY7	Enolase-phosphatase E1 OS=Homo sapiens (Human) OX=9606 GN=ENOPH1 PE=1 SV=1	ENOPH1	28,9	1,49	0,033395824
FALSO	High	Master Protein	P50991	T-complex protein 1 subunit delta OS=Homo sapiens (Human) OX=9606 GN=CCT4 PE=1 SV=4	CCT4	57,9	1,49	0,243928135
FALSO	High	Master Protein	Q14141	Septin-6 OS=Homo sapiens (Human) OX=9606 GN=SEPTIN6 PE=1 SV=4	SEPT6	49,7	1,48	0,18063985
FALSO	High	None	Q2VIR3	Eukaryotic translation initiation factor 2 subunit 3B OS=Homo sapiens (Human) OX=9606 GN=EIF2S3B PE=2 S	LOC255308	51,2	1,48	0,227922609
FALSO	Medium	Master Protein	Q14919	Dr1-associated corepressor OS=Homo sapiens (Human) OX=9606 GN=DRAP1 PE=1 SV=3	DRAP1	22,3	1,47	0,16116097
FALSO	High	Master Protein	P20933	N(4)-(beta-N-acetylglucosaminyl)-L-asparaginase OS=Homo sapiens (Human) OX=9606 GN=AGA PE=1 SV=2	AGA	37,2	1,47	0,180888687
FALSO	High	None	Q8NFI4	Putative protein FAM10A5 OS=Homo sapiens (Human) OX=9606 GN=ST13P5 PE=5 SV=1	ST13P5	41,4	1,47	0,080469928
FALSO	High	Master Protein	P48147	Prolyl endopeptidase OS=Homo sapiens (Human) OX=9606 GN=PREP PE=1 SV=2	PREP	80,6	1,45	0,360263612
FALSO	High	Master Protein	P05546	Heparin cofactor 2 OS=Homo sapiens (Human) OX=9606 GN=SERPIND1 PE=1 SV=3	SERPIND1	57	1,45	0,035756682
FALSO	High	Master Protein	P09455	Retinol-binding protein 1 OS=Homo sapiens (Human) OX=9606 GN=RBP1 PE=1 SV=2	RBP1	15,8	1,45	0,337096321
FALSO	High	Master Protein	Q12931	Heat shock protein 75 kDa, mitochondrial OS=Homo sapiens (Human) OX=9606 GN=TRAP1 PE=1 SV=3	TRAP1	80,1	1,45	0,162086807
FALSO	High	Master Protein	A0A075B7B8	IGv domain-containing protein OS=Homo sapiens (Human) OX=9606 GN=IGHV3OR16-12 PE=1 SV=1	IGHV3OR16-12	12,9	1,45	0,110973363
FALSO	High	Master Protein	Q1KMD3	Heterogeneous nuclear ribonucleoprotein U-like protein 2 OS=Homo sapiens (Human) OX=9606 GN=HNRNPU	HNRNPUL2	85,1	1,44	0,109320507
FALSO	Medium	Master Protein	A0AVT1	Ubiquitin-like modifier-activating enzyme 6 OS=Homo sapiens (Human) OX=9606 GN=UBA6 PE=1 SV=1	UBA6	118	1,44	0,385294696
FALSO	High	Master Protein	P61769	Beta-2-microglobulin OS=Homo sapiens (Human) OX=9606 GN=B2M PE=1 SV=1	B2M	13,7	1,44	0,129546206
FALSO	High	Master Protein	O00764	Pyridoxal kinase OS=Homo sapiens (Human) OX=9606 GN=PDXK PE=1 SV=1	PDXK	35,1	1,44	0,040938003
FALSO	Medium	Master Protein	P34896	Serine hydroxymethyltransferase, cytosolic OS=Homo sapiens (Human) OX=9606 GN=SHMT1 PE=1 SV=1	SHMT1	53	1,44	#¡DIV/0!
FALSO	High	Master Protein	O95319	CUGBP Elav-like family member 2 OS=Homo sapiens (Human) OX=9606 GN=CELF2 PE=1 SV=1	CELF2	54,3	1,44	0,117156916
FALSO	High	Master Protein	P11171	Protein 4.1 OS=Homo sapiens (Human) OX=9606 GN=EPB41 PE=1 SV=4	EPB41	97	1,44	0,462309929
FALSO	High	Master Protein	P37802	Transgelin-2 OS=Homo sapiens (Human) OX=9606 GN=TAGLN2 PE=1 SV=3	TAGLN2	22,4	1,44	0,05091538
FALSO	Medium	Master Protein	Q96JD6	1,5-anhydro-D-fructose reductase OS=Homo sapiens (Human) OX=9606 GN=AKR1E2 PE=1 SV=2	AKR1E2	36,6	1,43	0,183591411
FALSO	Medium	Master Protein	Q9Y625	Glypican-6 OS=Homo sapiens (Human) OX=9606 GN=GPC6 PE=1 SV=1	GPC6	62,7	1,43	0,440310752
FALSO	High	Master Protein	Q9HC38	Glyoxalase domain-containing protein 4 OS=Homo sapiens (Human) OX=9606 GN=GLOD4 PE=1 SV=1	GLOD4	34,8	1,43	0,075936168
FALSO	Medium	Master Protein	P09172	Dopamine beta-hydroxylase OS=Homo sapiens (Human) OX=9606 GN=DBH PE=1 SV=3	DBH	69	1,43	0,192284102
FALSO	High	Master Protein	P27169	Serum paraoxonase/arylesterase 1 OS=Homo sapiens (Human) OX=9606 GN=PON1 PE=1 SV=3	PON1	39,7	1,43	0,123763981
FALSO	High	Master Protein	P07358	Complement component C8 beta chain OS=Homo sapiens (Human) OX=9606 GN=C8B PE=1 SV=3	C8B	67	1,43	0,085684832
FALSO	Medium	Master Protein	Q13418	Integrin-linked protein kinase OS=Homo sapiens (Human) OX=9606 GN=ILK PE=1 SV=2	ILK	51,4	1,43	0,621169633
FALSO	High	Master Protein	P01780	Immunoglobulin heavy variable 3-7 OS=Homo sapiens (Human) OX=9606 GN=IGHV3-7 PE=1 SV=2	IGHV3-7	12,9	1,41	0,041972445
FALSO	High	Master Protein	A0A0G2JRQ6	Ig-like domain-containing protein OS=Homo sapiens (Human) OX=9606 GN=ENSG00000281759 PE=1 SV=4	ENSG0000028175	12,7	1,41	0,30775411
FALSO	High	Master Protein	P43251	Biotinidase OS=Homo sapiens (Human) OX=9606 GN=BTD PE=1 SV=2	BTD	61,1	1,41	0,047096647
FALSO	High	Master Protein	P24592	Insulin-like growth factor-binding protein 6 OS=Homo sapiens (Human) OX=9606 GN=IGFBP6 PE=1 SV=1	IGFBP6	25,3	1,41	0,192793509

FALSO	High	Master Protein	P17931	Galectin-3 OS=Homo sapiens (Human) OX=9606 GN=LGALS3 PE=1 SV=5	LGALS3	26,1	1,41	0,078304196
FALSO	High	Master Protein	P09382	Galectin-1 OS=Homo sapiens (Human) OX=9606 GN=LGALS1 PE=1 SV=2	LGALS1	14,7	1,40	0,119762533
FALSO	High	Master Protein	P19367	Hexokinase-1 OS=Homo sapiens (Human) OX=9606 GN=HK1 PE=1 SV=3	HK1	102	1,40	0,200787845
FALSO	High	Master Protein	P61088	Ubiquitin-conjugating enzyme E2 N OS=Homo sapiens (Human) OX=9606 GN=UBE2N PE=1 SV=1	UBE2N	17,1	1,40	0,182223833
FALSO	High	Master Protein	P31151	Protein S100-A7 OS=Homo sapiens (Human) OX=9606 GN=S100A7 PE=1 SV=4	S100A7	11,5	1,40	0,468859579
FALSO	High	Master Protein	P08631	Tyrosine-protein kinase HCK OS=Homo sapiens (Human) OX=9606 GN=HCK PE=1 SV=5	HCK	59,6	1,40	0,216643931
FALSO	High	Master Protein	P08575	Receptor-type tyrosine-protein phosphatase C OS=Homo sapiens (Human) OX=9606 GN=PTPRC PE=1 SV=3	PTPRC	147	1,39	0,297272681
FALSO	High	Master Protein	P40121	Macrophage-capping protein OS=Homo sapiens (Human) OX=9606 GN=CAPG PE=1 SV=2	CAPG	38,5	1,39	0,064335992
FALSO	High	None	Q2M2H8	Probable maltase-glucoamylase 2 OS=Homo sapiens (Human) OX=9606 GN=MGAM2 PE=2 SV=3	LOC93432; MGAM	278	1,39	0,192844927
FALSO	High	None	P01762	Immunoglobulin heavy variable 3-11 OS=Homo sapiens (Human) OX=9606 GN=IGHV3-11 PE=1 SV=2	IGHV3-11	12,9	1,39	0,070741465
FALSO	High	None	P01763	Immunoglobulin heavy variable 3-48 OS=Homo sapiens (Human) OX=9606 GN=IGHV3-48 PE=1 SV=2	IGHV3-48	12,8	1,39	0,070741465
FALSO	High	Master Protein	O95466	Formin-like protein 1 OS=Homo sapiens (Human) OX=9606 GN=FMNL1 PE=1 SV=3	FMNL1	122	1,39	0,337048008
FALSO	High	Master Protein	P08185	Corticosteroid-binding globulin OS=Homo sapiens (Human) OX=9606 GN=SERPINA6 PE=1 SV=1	SERPINA6	45,1	1,39	0,232457418
FALSO	High	Master Protein	Q9Y3C8	Ubiquitin-fold modifier-conjugating enzyme 1 OS=Homo sapiens (Human) OX=9606 GN=UFC1 PE=1 SV=3	UFC1	19,4	1,39	0,225455171
FALSO	High	Master Protein	P02652	Apolipoprotein A-II OS=Homo sapiens (Human) OX=9606 GN=APOA2 PE=1 SV=1	APOA2	11,2	1,39	0,060869879
FALSO	Medium	Master Protein	Q66K66	Transmembrane protein 198 OS=Homo sapiens (Human) OX=9606 GN=TMEM198 PE=1 SV=1	TMEM198	39,4	1,39	0,55419046
FALSO	High	None	Q8NHP1	Aflatoxin B1 aldehyde reductase member 4 OS=Homo sapiens (Human) OX=9606 GN=AKR7L PE=2 SV=7	AKR7L	36,9	1,38	0,162247515
FALSO	High	Master Protein	P41091	Eukaryotic translation initiation factor 2 subunit 3 OS=Homo sapiens (Human) OX=9606 GN=EIF2S3 PE=1 SV=	EIF2S3	51,1	1,38	0,247962311
FALSO	Medium	Master Protein	A0A0B4J1Y8	Immunoglobulin lambda variable 9-49 OS=Homo sapiens (Human) OX=9606 GN=IGLV9-49 PE=1 SV=1	IGLV9-49	13	1,38	0,572582425
FALSO	Medium	Master Protein	Q13045	Protein flightless-1 homolog OS=Homo sapiens (Human) OX=9606 GN=FLII PE=1 SV=2	FLII	145	1,38	0,315059988
FALSO	High	Master Protein	P53999	Activated RNA polymerase II transcriptional coactivator p15 OS=Homo sapiens (Human) OX=9606 GN=SUB1 F	SUB1	14,4	1,38	0,182264508
FALSO	Medium	None	Q9Y2J2	Band 4.1-like protein 3 OS=Homo sapiens (Human) OX=9606 GN=EPB41L3 PE=1 SV=2	EPB41L3	121	1,38	0,571583481
FALSO	Medium	None	Q9H4G0	Band 4.1-like protein 1 OS=Homo sapiens (Human) OX=9606 GN=EPB41L1 PE=1 SV=2	EPB41L1	98,4	1,38	0,571583481
FALSO	High	Master Protein	P25325	3-mercaptopyruvate sulfurtransferase OS=Homo sapiens (Human) OX=9606 GN=MPST PE=1 SV=3	MPST	33,2	1,38	0,098431268
FALSO	High	None	Q86SG5	Protein S100-A7A OS=Homo sapiens (Human) OX=9606 GN=S100A7A PE=1 SV=3	S100A7A	11,3	1,37	0,491875563
FALSO	High	None	P52789	Hexokinase-2 OS=Homo sapiens (Human) OX=9606 GN=HK2 PE=1 SV=2	HK2	102	1,37	0,24973274
FALSO	High	Master Protein	P07737	Profilin-1 OS=Homo sapiens (Human) OX=9606 GN=PFN1 PE=1 SV=2	PFN1	15	1,37	0,049545442
FALSO	High	None	P51668	Ubiquitin-conjugating enzyme E2 D1 OS=Homo sapiens (Human) OX=9606 GN=UBE2D1 PE=1 SV=1	UBE2D1	16,6	1,37	0,233059874
FALSO	High	None	Q9Y2X8	Ubiquitin-conjugating enzyme E2 D4 OS=Homo sapiens (Human) OX=9606 GN=UBE2D4 PE=1 SV=1	UBE2D4	16,6	1,37	0,233059874
FALSO	High	None	Q8TAA3	Proteasome subunit alpha-type 8 OS=Homo sapiens (Human) OX=9606 GN=PSMA8 PE=2 SV=3	PSMA8	28,5	1,37	0,158438721
FALSO	High	Master Protein	P35542	Serum amyloid A-4 protein OS=Homo sapiens (Human) OX=9606 GN=SAA4 PE=1 SV=2	SAA4	14,7	1,37	0,398008691
FALSO	High	Master Protein	Q9UKA9	Polypyrimidine tract-binding protein 2 OS=Homo sapiens (Human) OX=9606 GN=PTBP2 PE=1 SV=1	PTBP2	57,5	1,37	0,439804469
FALSO	High	Master Protein	O15212	Prefoldin subunit 6 OS=Homo sapiens (Human) OX=9606 GN=PFDN6 PE=1 SV=1	PFDN6	14,6	1,36	0,161219999

FALSO	High	Master Protein	A0A0A0MS15	Immunoglobulin heavy variable 3-49 OS=Homo sapiens (Human) OX=9606 GN=IGHV3-49 PE=3 SV=1	IGHV3-49	13	1,35	0,127806246
FALSO	High	Master Protein	P06312	Immunoglobulin kappa variable 4-1 OS=Homo sapiens (Human) OX=9606 GN=IGKV4-1 PE=1 SV=1	IGKV4-1	13,4	1,35	0,089635979
FALSO	High	Master Protein	P06865	Beta-hexosaminidase subunit alpha OS=Homo sapiens (Human) OX=9606 GN=HEXA PE=1 SV=2	HEXA	60,7	1,35	0,316552449
FALSO	High	Master Protein	O75695	Protein XRP2 OS=Homo sapiens (Human) OX=9606 GN=RP2 PE=1 SV=4	RP2	39,6	1,35	0,151504161
FALSO	High	Master Protein	P54578	Ubiquitin carboxyl-terminal hydrolase 14 OS=Homo sapiens (Human) OX=9606 GN=USP14 PE=1 SV=3	USP14	56	1,35	0,348118628
FALSO	High	Master Protein	P62328	Thymosin beta-4 OS=Homo sapiens (Human) OX=9606 GN=TMSB4X PE=1 SV=2	TMSB4X	5,1	1,35	0,368019738
FALSO	High	Master Protein	P04180	Phosphatidylcholine-sterol acyltransferase OS=Homo sapiens (Human) OX=9606 GN=LCAT PE=1 SV=1	LCAT	49,5	1,35	0,391707652
FALSO	High	Master Protein	P62993	Growth factor receptor-bound protein 2 OS=Homo sapiens (Human) OX=9606 GN=GRB2 PE=1 SV=1	GRB2	25,2	1,35	0,174603435
FALSO	Medium	Master Protein	P15428	15-hydroxyprostaglandin dehydrogenase [NAD(+)] OS=Homo sapiens (Human) OX=9606 GN=HPGD PE=1 SV	HPGD	29	1,34	0,368528026
FALSO	Medium	Master Protein Candidat	Q53S08	Ras-related protein Rab-6D OS=Homo sapiens (Human) OX=9606 GN=RAB6D PE=2 SV=1	RAB6D	28,2	1,34	0,540258598
FALSO	Medium	Master Protein	Q9H0N0	Ras-related protein Rab-6C OS=Homo sapiens (Human) OX=9606 GN=RAB6C PE=1 SV=2	RAB6C	28,3	1,34	0,540258598
FALSO	High	Master Protein	Q9H4M9	EH domain-containing protein 1 OS=Homo sapiens (Human) OX=9606 GN=EHD1 PE=1 SV=2	EHD1	60,6	1,34	0,203588428
FALSO	High	Master Protein	P01871	Immunoglobulin heavy constant mu OS=Homo sapiens (Human) OX=9606 GN=IGHM PE=1 SV=4	IGHM	49,4	1,34	0,146143781
FALSO	Medium	Master Protein	Q86Y82	Syntaxin-12 OS=Homo sapiens (Human) OX=9606 GN=STX12 PE=1 SV=1	STX12	31,6	1,34	0,581748575
FALSO	High	Master Protein	P06703	Protein S100-A6 OS=Homo sapiens (Human) OX=9606 GN=S100A6 PE=1 SV=1	S100A6	10,2	1,34	0,238100085
FALSO	High	Master Protein	Q92688	Acidic leucine-rich nuclear phosphoprotein 32 family member B OS=Homo sapiens (Human) OX=9606 GN=AN	ANP32B	28,8	1,34	0,074606399
FALSO	High	Master Protein	Q3LXA3	Triokinase/FMN cyclase OS=Homo sapiens (Human) OX=9606 GN=TKFC PE=1 SV=2	TKFC	58,9	1,33	0,291407866
FALSO	High	Master Protein	Q96PD5	N-acetylmuramoyl-L-alanine amidase OS=Homo sapiens (Human) OX=9606 GN=PGLYRP2 PE=1 SV=1	PGLYRP2	62,2	1,33	0,035406283
FALSO	High	Master Protein	Q6UX06	Olfactomedin-4 OS=Homo sapiens (Human) OX=9606 GN=OLFM4 PE=1 SV=1	OLFM4	57,2	1,33	0,238533343
FALSO	High	Master Protein	P04040	Catalase OS=Homo sapiens (Human) OX=9606 GN=CAT PE=1 SV=3	CAT	59,7	1,33	0,055690751
FALSO	High	Master Protein	Q9BS40	Latexin OS=Homo sapiens (Human) OX=9606 GN=LXN PE=1 SV=2	LXN	25,7	1,33	0,653404204
FALSO	High	Master Protein	Q9Y6R7	IgGFc-binding protein OS=Homo sapiens (Human) OX=9606 GN=FCGBP PE=1 SV=3	FCGBP	572	1,33	0,234371571
FALSO	High	Master Protein	Q9H4G4	Golgi-associated plant pathogenesis-related protein 1 OS=Homo sapiens (Human) OX=9606 GN=GLIPR2 PE=1	GLIPR2	17,2	1,33	0,1265481
FALSO	High	Master Protein	P22792	Carboxypeptidase N subunit 2 OS=Homo sapiens (Human) OX=9606 GN=CPN2 PE=1 SV=3	CPN2	60,5	1,33	0,043071836
FALSO	High	Master Protein	P00739	Haptoglobin-related protein OS=Homo sapiens (Human) OX=9606 GN=HPR PE=2 SV=2	HPR	39	1,33	0,392281643
FALSO	High	Master Protein	P47929	Galectin-7 OS=Homo sapiens (Human) OX=9606 GN=LGALS7B PE=1 SV=2	LGALS7	15,1	1,33	0,321189779
FALSO	High	Master Protein	Q13813	Spectrin alpha chain, non-erythrocytic 1 OS=Homo sapiens (Human) OX=9606 GN=SPTAN1 PE=1 SV=3	SPTAN1	284	1,32	0,345405315
FALSO	High	Master Protein	Q6P4A8	Phospholipase B-like 1 OS=Homo sapiens (Human) OX=9606 GN=PLBD1 PE=1 SV=2	PLBD1	63,2	1,32	0,17968488
FALSO	High	Master Protein	P50395	Rab GDP dissociation inhibitor beta OS=Homo sapiens (Human) OX=9606 GN=GDI2 PE=1 SV=2	GDI2	50,6	1,32	0,136431966
FALSO	High	Master Protein	Q9H0W9	Ester hydrolase C11orf54 OS=Homo sapiens (Human) OX=9606 GN=C11orf54 PE=1 SV=1	C11orf54	35,1	1,32	0,179570121
FALSO	High	Master Protein	Q9UL25	Ras-related protein Rab-21 OS=Homo sapiens (Human) OX=9606 GN=RAB21 PE=1 SV=3	RAB21	24,3	1,32	0,298505074
FALSO	High	Master Protein	O15511	Actin-related protein 2/3 complex subunit 5 OS=Homo sapiens (Human) OX=9606 GN=ARPC5 PE=1 SV=3	ARPC5	16,3	1,32	0,175726315
FALSO	High	Master Protein	P26447	Protein S100-A4 OS=Homo sapiens (Human) OX=9606 GN=S100A4 PE=1 SV=1	S100A4	11,7	1,31	0,196374761

FALSO	High	Master Protein	O43488	Aflatoxin B1 aldehyde reductase member 2 OS=Homo sapiens (Human) OX=9606 GN=AKR7A2 PE=1 SV=3	AKR7A2	39,6	1,31	0,268863529
FALSO	High	Master Protein	P06702	Protein S100-A9 OS=Homo sapiens (Human) OX=9606 GN=S100A9 PE=1 SV=1	S100A9	13,2	1,31	0,386087401
FALSO	High	None	A8MVU1	Putative neutrophil cytosol factor 1C OS=Homo sapiens (Human) OX=9606 GN=NCF1C PE=5 SV=1	NCF1C	41,8	1,31	0,120381301
FALSO	High	None	A6NI72	Putative neutrophil cytosol factor 1B OS=Homo sapiens (Human) OX=9606 GN=NCF1B PE=5 SV=2	NCF1B	44,8	1,31	0,120381301
FALSO	High	Master Protein	P27695	DNA-(apurinic or apyrimidinic site) lyase OS=Homo sapiens (Human) OX=9606 GN=APEX1 PE=1 SV=2	APEX1	35,5	1,31	0,142931323
FALSO	High	Master Protein	Q9UHA4	Ragulator complex protein LAMTOR3 OS=Homo sapiens (Human) OX=9606 GN=LAMTOR3 PE=1 SV=1	LAMTOR3	13,6	1,31	0,286997461
FALSO	High	Master Protein	Q99536	Synaptic vesicle membrane protein VAT-1 homolog OS=Homo sapiens (Human) OX=9606 GN=VAT1 PE=1 SV=2	VAT1	41,9	1,31	0,23471095
FALSO	High	Master Protein	P31949	Protein S100-A11 OS=Homo sapiens (Human) OX=9606 GN=S100A11 PE=1 SV=2	S100A11	11,7	1,30	0,599620439
FALSO	Medium	Master Protein Candidat	P36873	Serine/threonine-protein phosphatase PP1-gamma catalytic subunit OS=Homo sapiens (Human) OX=9606 GI	PPP1CC	37	1,30	0,196208799
FALSO	Medium	Master Protein	P62136	Serine/threonine-protein phosphatase PP1-alpha catalytic subunit OS=Homo sapiens (Human) OX=9606 GN=	PPP1CA	37,5	1,30	0,196208799
FALSO	Medium	Master Protein Candidat	P62140	Serine/threonine-protein phosphatase PP1-beta catalytic subunit OS=Homo sapiens (Human) OX=9606 GN=I	PPP1CB	37,2	1,30	0,196208799
FALSO	High	Master Protein	P55072	Transitional endoplasmic reticulum ATPase OS=Homo sapiens (Human) OX=9606 GN=VCP PE=1 SV=4	VCP	89,3	1,30	0,285011847
FALSO	High	Master Protein	A0A0C4DH35	Probable non-functional immunoglobulin heavy variable 3-35 OS=Homo sapiens (Human) OX=9606 GN=IGH	IGHV3-35	12,8	1,30	0,286085351
FALSO	High	Master Protein	P20073	Annexin A7 OS=Homo sapiens (Human) OX=9606 GN=ANXA7 PE=1 SV=3	ANXA7	52,7	1,30	0,181153081
FALSO	High	Master Protein	O14818	Proteasome subunit alpha type-7 OS=Homo sapiens (Human) OX=9606 GN=PSMA7 PE=1 SV=1	PSMA7	27,9	1,30	0,184376991
FALSO	High	Master Protein	P53004	Biliverdin reductase A OS=Homo sapiens (Human) OX=9606 GN=BLVRA PE=1 SV=2	BLVRA	33,4	1,29	0,100863572
FALSO	High	Master Protein	P14598	Neutrophil cytosol factor 1 OS=Homo sapiens (Human) OX=9606 GN=NCF1 PE=1 SV=4	NCF1	44,7	1,29	0,14618155
FALSO	High	Master Protein	Q8IYS5	Osteoclast-associated immunoglobulin-like receptor OS=Homo sapiens (Human) OX=9606 GN=OSCAR PE=1 S	OSCAR	30,5	1,29	0,230834362
FALSO	High	None	A0A0C4DH32	Immunoglobulin heavy variable 3-20 OS=Homo sapiens (Human) OX=9606 GN=IGHV3-20 PE=3 SV=2	IGHV3-20	12,7	1,29	0,141724235
FALSO	High	None	A0A075B7F0	Ig-like domain-containing protein OS=Homo sapiens (Human) OX=9606 GN=IGHV3OR16-10 PE=1 SV=1	IGHV3OR16-10	12,6	1,29	0,141724235
FALSO	High	Master Protein	P02654	Apolipoprotein C-I OS=Homo sapiens (Human) OX=9606 GN=APOC1 PE=1 SV=1	APOC1	9,3	1,29	0,314427321
FALSO	High	Master Protein	Q14624	Inter-alpha-trypsin inhibitor heavy chain H4 OS=Homo sapiens (Human) OX=9606 GN=ITIH4 PE=1 SV=4	ITIH4	103	1,29	0,008753027
FALSO	High	Master Protein	Q9Y2Q5	Ragulator complex protein LAMTOR2 OS=Homo sapiens (Human) OX=9606 GN=LAMTOR2 PE=1 SV=1	LAMTOR2	13,5	1,28	0,37641597
FALSO	High	Master Protein	P00568	Adenylate kinase isoenzyme 1 OS=Homo sapiens (Human) OX=9606 GN=AK1 PE=1 SV=3	AK1	21,6	1,28	0,372815996
FALSO	High	Master Protein	Q04446	1,4-alpha-glucan-branching enzyme OS=Homo sapiens (Human) OX=9606 GN=GBE1 PE=1 SV=3	GBE1	80,4	1,28	0,39535016
FALSO	High	Master Protein	P07359	Platelet glycoprotein Ib alpha chain OS=Homo sapiens (Human) OX=9606 GN=GP1BA PE=1 SV=2	GP1BA	71,5	1,28	0,23233765
FALSO	High	Master Protein	P04196	Histidine-rich glycoprotein OS=Homo sapiens (Human) OX=9606 GN=HRG PE=1 SV=1	HRG	59,5	1,28	0,060919012
FALSO	High	Master Protein	Q9BYX7	Putative beta-actin-like protein 3 OS=Homo sapiens (Human) OX=9606 GN=POTEKP PE=5 SV=1	POTEKP	42	1,28	0,683591584
FALSO	High	None	P40123	Adenylyl cyclase-associated protein 2 OS=Homo sapiens (Human) OX=9606 GN=CAP2 PE=1 SV=1	CAP2	52,8	1,27	0,424505036
FALSO	High	Master Protein	P47755	F-actin-capping protein subunit alpha-2 OS=Homo sapiens (Human) OX=9606 GN=CAPZA2 PE=1 SV=3	CAPZA2	32,9	1,27	0,3294294
FALSO	High	Master Protein	Q16881	Thioredoxin reductase 1, cytoplasmic OS=Homo sapiens (Human) OX=9606 GN=TXNRD1 PE=1 SV=3	TXNRD1	70,9	1,27	0,334339989
FALSO	Medium	Master Protein	Q96SI9	Spermatid perinuclear RNA-binding protein OS=Homo sapiens (Human) OX=9606 GN=STRBP PE=1 SV=1	STRBP	73,6	1,27	0,45552436
FALSO	High	None	Q5JXB2	Putative ubiquitin-conjugating enzyme E2 N-like OS=Homo sapiens (Human) OX=9606 GN=UBE2NL PE=1 SV=	UBE2NL	17,4	1,26	0,377344896

FALSO	High	Master Protein	O75015	Low affinity immunoglobulin gamma Fc region receptor III-B OS=Homo sapiens (Human) OX=9606 GN=FCGR3	FCGR3B	26,2	1,26	0,215973588
FALSO	High	Master Protein	P07900	Heat shock protein HSP 90-alpha OS=Homo sapiens (Human) OX=9606 GN=HSP90AA1 PE=1 SV=5	HSP90AA1	47,7	1,26	0,546193714
FALSO	High	Master Protein	Q15424	Scaffold attachment factor B1 OS=Homo sapiens (Human) OX=9606 GN=SAFB PE=1 SV=4	SAFB	103	1,26	0,521903511
FALSO	High	Master Protein	Q14019	Coactosin-like protein OS=Homo sapiens (Human) OX=9606 GN=COTL1 PE=1 SV=3	COTL1	15,9	1,26	0,274065693
FALSO	High	Master Protein	A0A0J9YY99	Ig-like domain-containing protein OS=Homo sapiens (Human) OX=9606 GN=ENSG00000282150 PE=1 SV=1	ENSG00000282150	13	1,25	0,188198034
FALSO	High	Master Protein Candidat	A0A0C4DH41	Immunoglobulin heavy variable 4-61 OS=Homo sapiens (Human) OX=9606 GN=IGHV4-61 PE=3 SV=1	IGHV4-61	13,1	1,25	0,191196039
FALSO	High	Master Protein Candidat	P01824	Immunoglobulin heavy variable 4-39 OS=Homo sapiens (Human) OX=9606 GN=IGHV4-39 PE=1 SV=2	IGHV4-39	13,9	1,25	0,191196039
FALSO	High	Master Protein Candidat	P0DP06	Immunoglobulin heavy variable 4-30-4 OS=Homo sapiens (Human) OX=9606 GN=IGHV4-30-4 PE=3 SV=1	IGHV4-30-4	13,1	1,25	0,191196039
FALSO	High	Master Protein	P06331	Immunoglobulin heavy variable 4-34 OS=Homo sapiens (Human) OX=9606 GN=IGHV4-34 PE=1 SV=2	IGHV4-34	13,8	1,25	0,191196039
FALSO	High	Master Protein Candidat	P0DP08	Immunoglobulin heavy variable 4-38-2 OS=Homo sapiens (Human) OX=9606 GN=IGHV4-38-2 PE=3 SV=1	IGHV4-B; IGHV4-3	13	1,25	0,191196039
FALSO	High	Master Protein Candidat	P01825	Immunoglobulin heavy variable 4-59 OS=Homo sapiens (Human) OX=9606 GN=IGHV4-59 PE=1 SV=2	IGHV4-59	12,9	1,25	0,191196039
FALSO	High	Master Protein	O00391	Sulfhydryl oxidase 1 OS=Homo sapiens (Human) OX=9606 GN=QSOX1 PE=1 SV=3	QSOX1	82,5	1,25	0,245949924
FALSO	High	Master Protein	P62826	GTP-binding nuclear protein Ran OS=Homo sapiens (Human) OX=9606 GN=RAN PE=1 SV=3	RAN	24,4	1,24	0,371475407
FALSO	High	None	Q32P51	Heterogeneous nuclear ribonucleoprotein A1-like 2 OS=Homo sapiens (Human) OX=9606 GN=HNRNPA1L2 P	HNRNPA1L2	34,2	1,24	0,234447737
FALSO	High	Master Protein	P19827	Inter-alpha-trypsin inhibitor heavy chain H1 OS=Homo sapiens (Human) OX=9606 GN=ITIH1 PE=1 SV=3	ITIH1	101	1,24	0,350723157
FALSO	High	Master Protein	P05109	Protein S100-A8 OS=Homo sapiens (Human) OX=9606 GN=S100A8 PE=1 SV=1	S100A8	10,8	1,24	0,504926962
FALSO	High	Master Protein	P15169	Carboxypeptidase N catalytic chain OS=Homo sapiens (Human) OX=9606 GN=CPN1 PE=1 SV=1	CPN1	52,3	1,24	0,346744921
FALSO	High	Master Protein	O75629	Protein CREG1 OS=Homo sapiens (Human) OX=9606 GN=CREG1 PE=1 SV=1	CREG1	24,1	1,24	0,203758351
FALSO	High	Master Protein	O75563	Src kinase-associated phosphoprotein 2 OS=Homo sapiens (Human) OX=9606 GN=SKAP2 PE=1 SV=1	SKAP2	41,2	1,23	0,468209151
FALSO	High	None	Q6ZMR3	L-lactate dehydrogenase A-like 6A OS=Homo sapiens (Human) OX=9606 GN=LDHAL6A PE=2 SV=1	LDHAL6A	36,5	1,23	0,346808122
FALSO	High	None	P07864	L-lactate dehydrogenase C chain OS=Homo sapiens (Human) OX=9606 GN=LDHC PE=1 SV=4	LDHC	36,3	1,23	0,346808122
FALSO	High	Master Protein	P01742	Immunoglobulin heavy variable 1-69 OS=Homo sapiens (Human) OX=9606 GN=IGHV1-69 PE=1 SV=2	IGHV1-69	12,7	1,23	0,271633727
FALSO	High	Master Protein Candidat	A0A0B4J2H0	Immunoglobulin heavy variable 1-69D OS=Homo sapiens (Human) OX=9606 GN=IGHV1-69D PE=1 SV=1	IGHV1-69D	12,7	1,23	0,271633727
FALSO	High	None	Q7L7L0	Histone H2A type 3 OS=Homo sapiens (Human) OX=9606 GN=HIST3H2A PE=1 SV=3	HIST3H2A	14,1	1,23	0,387823511
FALSO	High	None	P04908	Histone H2A type 1-B/E OS=Homo sapiens (Human) OX=9606 GN=H2AC8 PE=1 SV=2	HIST1H2AB; HIST1	14,1	1,23	0,387823511
FALSO	High	Master Protein	P29692	Elongation factor 1-delta OS=Homo sapiens (Human) OX=9606 GN=EEF1D PE=1 SV=5	EEF1D	31,1	1,23	0,223679742
FALSO	High	Master Protein	P22234	Multifunctional protein ADE2 OS=Homo sapiens (Human) OX=9606 GN=PAICS PE=1 SV=3	PAICS	47	1,23	0,322194081
FALSO	High	Master Protein	Q93077	Histone H2A type 1-C OS=Homo sapiens (Human) OX=9606 GN=HIST1H2AC PE=1 SV=3	HIST1H2AC	14,1	1,23	0,386244839
FALSO	High	Master Protein	P30153	Serine/threonine-protein phosphatase 2A 65 kDa regulatory subunit A alpha isoform OS=Homo sapiens (Hum	PPP2R1A	65,3	1,23	0,432908397
FALSO	High	Master Protein	Q9BTT0	Acidic leucine-rich nuclear phosphoprotein 32 family member E OS=Homo sapiens (Human) OX=9606 GN=AN	ANP32E	30,7	1,23	0,342666888
FALSO	High	Master Protein	P11413	Glucose-6-phosphate 1-dehydrogenase OS=Homo sapiens (Human) OX=9606 GN=G6PD PE=1 SV=4	G6PD	59,2	1,22	0,317034084
FALSO	High	Master Protein	P52907	F-actin-capping protein subunit alpha-1 OS=Homo sapiens (Human) OX=9606 GN=CAPZA1 PE=1 SV=3	CAPZA1	32,9	1,22	0,16847731
FALSO	High	Master Protein	P0DMV8	Heat shock 70 kDa protein 1A OS=Homo sapiens (Human) OX=9606 GN=HSPA1A PE=1 SV=1	HSPA1B	70	1,22	0,30083097

FALSO	High	Master Protein	P30048	Thioredoxin-dependent peroxide reductase, mitochondrial OS=Homo sapiens (Human) OX=9606 GN=PRDX3	PRDX3	27,7	1,22	0,40490706
FALSO	Medium	Master Protein	Q6UWV6	Ectonucleotide pyrophosphatase/phosphodiesterase family member 7 OS=Homo sapiens (Human) OX=9606	ENPP7	51,5	1,22	0,421784461
FALSO	High	Master Protein	P29373	Cellular retinoic acid-binding protein 2 OS=Homo sapiens (Human) OX=9606 GN=CRABP2 PE=1 SV=2	CRABP2	15,7	1,22	0,044931731
FALSO	High	Master Protein	P30086	Phosphatidylethanolamine-binding protein 1 OS=Homo sapiens (Human) OX=9606 GN=PEBP1 PE=1 SV=3	PEBP1	21	1,22	0,247452609
FALSO	High	Master Protein	P39687	Acidic leucine-rich nuclear phosphoprotein 32 family member A OS=Homo sapiens (Human) OX=9606 GN=AN	ANP32A	28,6	1,22	0,272581497
FALSO	High	Master Protein	P26927	Hepatocyte growth factor-like protein OS=Homo sapiens (Human) OX=9606 GN=MST1 PE=1 SV=2	MST1	80,3	1,22	0,670599229
FALSO	Medium	Master Protein	Q9NQH7	Xaa-Pro aminopeptidase 3 OS=Homo sapiens (Human) OX=9606 GN=XPNPEP3 PE=1 SV=1	XPNPEP3	57	1,22	0,396400869
FALSO	High	None	P01768	Immunoglobulin heavy variable 3-30 OS=Homo sapiens (Human) OX=9606 GN=IGHV3-30 PE=1 SV=2	IGHV3-30	12,9	1,21	0,191272192
FALSO	High	Master Protein	P59190	Ras-related protein Rab-15 OS=Homo sapiens (Human) OX=9606 GN=RAB15 PE=1 SV=1	RAB15	24,4	1,21	0,559814294
FALSO	High	Master Protein	P68402	Platelet-activating factor acetylhydrolase IB subunit beta OS=Homo sapiens (Human) OX=9606 GN=PAFAH1B	PAFAH1B2	25,6	1,21	0,264914922
FALSO	High	Master Protein	P08571	Monocyte differentiation antigen CD14 OS=Homo sapiens (Human) OX=9606 GN=CD14 PE=1 SV=2	CD14	40,1	1,21	0,66802881
FALSO	High	Master Protein	P06744	Glucose-6-phosphate isomerase OS=Homo sapiens (Human) OX=9606 GN=GPI PE=1 SV=4	GPI	63,1	1,21	0,245183176
FALSO	High	Master Protein	P02679	Fibrinogen gamma chain OS=Homo sapiens (Human) OX=9606 GN=FGG PE=1 SV=3	FGG	51,5	1,21	0,334458731
FALSO	High	Master Protein	P04278	Sex hormone-binding globulin OS=Homo sapiens (Human) OX=9606 GN=SHBG PE=1 SV=2	SHBG	43,8	1,20	0,54599598
FALSO	High	Master Protein	P00441	Superoxide dismutase [Cu-Zn] OS=Homo sapiens (Human) OX=9606 GN=SOD1 PE=1 SV=2	SOD1	15,9	1,20	0,148129166
FALSO	High	Master Protein	Q15286	Ras-related protein Rab-35 OS=Homo sapiens (Human) OX=9606 GN=RAB35 PE=1 SV=1	RAB35	23	1,20	0,43007609
FALSO	Medium	Master Protein	A5D8W1	Cilia- and flagella-associated protein 69 OS=Homo sapiens (Human) OX=9606 GN=CFAP69 PE=1 SV=3	C7orf63; CFAP69	106	1,20	0,796803715
FALSO	High	None	P01764	Immunoglobulin heavy variable 3-23 OS=Homo sapiens (Human) OX=9606 GN=IGHV3-23 PE=1 SV=2	IGHV3-23	12,6	1,20	0,215240027
FALSO	High	Master Protein	Q99426	Tubulin-folding cofactor B OS=Homo sapiens (Human) OX=9606 GN=TBCB PE=1 SV=2	TBCB	27,3	1,20	0,438228671
FALSO	High	Master Protein	P02745	Complement C1q subcomponent subunit A OS=Homo sapiens (Human) OX=9606 GN=C1QA PE=1 SV=2	C1QA	26	1,19	0,428569904
FALSO	High	Master Protein	P20742	Pregnancy zone protein OS=Homo sapiens (Human) OX=9606 GN=PZP PE=1 SV=4	PZP	164	1,19	0,475174782
FALSO	High	Master Protein	P02753	Retinol-binding protein 4 OS=Homo sapiens (Human) OX=9606 GN=RBP4 PE=1 SV=3	RBP4	23	1,19	0,183325299
FALSO	High	Master Protein	Q99439	Calponin-2 OS=Homo sapiens (Human) OX=9606 GN=CNN2 PE=1 SV=4	CNN2	33,7	1,19	0,350544127
FALSO	High	Master Protein	P01602	Immunoglobulin kappa variable 1-5 OS=Homo sapiens (Human) OX=9606 GN=IGKV1-5 PE=1 SV=2	IGKV1-5	12,8	1,19	0,23650147
FALSO	High	Master Protein	P02750	Leucine-rich alpha-2-glycoprotein OS=Homo sapiens (Human) OX=9606 GN=LRG1 PE=1 SV=2	LRG1	38,2	1,19	0,251702873
FALSO	High	None	P16104	Histone H2AX OS=Homo sapiens (Human) OX=9606 GN=H2AX PE=1 SV=2	H2AFX	15,1	1,19	0,485968639
FALSO	High	Master Protein	P62269	40S ribosomal protein S18 OS=Homo sapiens (Human) OX=9606 GN=RPS18 PE=1 SV=3	RPS18	17,7	1,19	0,606445922
FALSO	High	Master Protein	P80511	Protein S100-A12 OS=Homo sapiens (Human) OX=9606 GN=S100A12 PE=1 SV=2	S100A12	10,6	1,19	0,417131028
FALSO	High	Master Protein	P04080	Cystatin-B OS=Homo sapiens (Human) OX=9606 GN=CSTB PE=1 SV=2	CSTB	11,1	1,19	0,680931911
FALSO	High	Master Protein	Q15631	Translin OS=Homo sapiens (Human) OX=9606 GN=TSN PE=1 SV=1	TSN	26,2	1,19	0,541680266
FALSO	High	Master Protein	O75608	Acyl-protein thioesterase 1 OS=Homo sapiens (Human) OX=9606 GN=LYPLA1 PE=1 SV=1	LYPLA1	24,7	1,18	0,583910563
FALSO	High	Master Protein	P52566	Rho GDP-dissociation inhibitor 2 OS=Homo sapiens (Human) OX=9606 GN=ARHGDIB PE=1 SV=3	ARHGDIB	23	1,18	0,271610209
FALSO	High	Master Protein	Q16539	Mitogen-activated protein kinase 14 OS=Homo sapiens (Human) OX=9606 GN=MAPK14 PE=1 SV=3	MAPK14	41,3	1,18	0,591808599

FALSO	High	Master Protein	Q6ZVX7	F-box only protein 50 OS=Homo sapiens (Human) OX=9606 GN=NCCRP1 PE=1 SV=1	NCCRP1	30,8	1,18	0,588840397
FALSO	High	Master Protein	P33121	Long-chain-fatty-acid--CoA ligase 1 OS=Homo sapiens (Human) OX=9606 GN=ACSL1 PE=1 SV=1	ACSL1	77,9	1,18	0,492842088
FALSO	High	Master Protein	O43866	CD5 antigen-like OS=Homo sapiens (Human) OX=9606 GN=CD5L PE=1 SV=1	CD5L	38,1	1,18	0,42240343
FALSO	High	Master Protein	Q06323	Proteasome activator complex subunit 1 OS=Homo sapiens (Human) OX=9606 GN=PSME1 PE=1 SV=1	PSME1	28,7	1,18	0,495676458
FALSO	High	Master Protein	P0C0L4	Complement C4-A OS=Homo sapiens (Human) OX=9606 GN=C4A PE=1 SV=2	C4A	193	1,18	0,079903137
FALSO	High	Master Protein	A0A075B6J9	Immunoglobulin lambda variable 2-18 OS=Homo sapiens (Human) OX=9606 GN=IGLV2-18 PE=3 SV=2	IGLV2-18	12,4	1,18	0,468483826
FALSO	Medium	Master Protein	P30519	Heme oxygenase 2 OS=Homo sapiens (Human) OX=9606 GN=HMOX2 PE=1 SV=2	HMOX2	36	1,17	0,773564264
FALSO	High	Master Protein	P29218	Inositol monophosphatase 1 OS=Homo sapiens (Human) OX=9606 GN=IMPA1 PE=1 SV=1	IMPA1	30,2	1,17	0,418602707
FALSO	Medium	None	A0A075B759	Peptidyl-prolyl cis-trans isomerase A-like 4E OS=Homo sapiens (Human) OX=9606 GN=PPIAL4E PE=3 SV=1	PPIAL4E	18,2	1,17	0,499229736
FALSO	Medium	None	A0A0B4J2A2	Peptidyl-prolyl cis-trans isomerase A-like 4C OS=Homo sapiens (Human) OX=9606 GN=PPIAL4C PE=2 SV=1	PPIAL4C	18,1	1,17	0,499229736
FALSO	Medium	None	P0DN37	Peptidyl-prolyl cis-trans isomerase A-like 4G OS=Homo sapiens (Human) OX=9606 GN=PPIAL4G PE=3 SV=1	PPIAL4G	18,2	1,17	0,499229736
FALSO	Medium	None	F5H284	Peptidyl-prolyl cis-trans isomerase A-like 4D OS=Homo sapiens (Human) OX=9606 GN=PPIAL4D PE=3 SV=1	PPIAL4D	18,2	1,17	0,499229736
FALSO	Medium	None	A0A075B767	Peptidyl-prolyl cis-trans isomerase A-like 4H OS=Homo sapiens (Human) OX=9606 GN=PPIAL4H PE=3 SV=1	LOC101060723	18,2	1,17	0,499229736
FALSO	High	Master Protein	P04275	von Willebrand factor OS=Homo sapiens (Human) OX=9606 GN=VWF PE=1 SV=4	VWF	309	1,17	0,686329359
FALSO	High	Master Protein	A0A0B4J1V6	Immunoglobulin heavy variable 3-73 OS=Homo sapiens (Human) OX=9606 GN=IGHV3-73 PE=3 SV=1	IGHV3-73	12,8	1,17	0,706355898
FALSO	High	Master Protein	Q9NR45	Sialic acid synthase OS=Homo sapiens (Human) OX=9606 GN=NANS PE=1 SV=2	NANS	40,3	1,17	0,531731073
FALSO	High	Master Protein	Q01459	Di-N-acetylchitobiase OS=Homo sapiens (Human) OX=9606 GN=CTBS PE=1 SV=1	CTBS	43,7	1,17	0,347151135
FALSO	High	Master Protein	Q13510	Acid ceramidase OS=Homo sapiens (Human) OX=9606 GN=ASAH1 PE=1 SV=5	ASAH1	44,6	1,17	0,567453953
FALSO	High	Master Protein	Q16543	Hsp90 co-chaperone Cdc37 OS=Homo sapiens (Human) OX=9606 GN=CDC37 PE=1 SV=1	CDC37	44,4	1,17	0,60178789
FALSO	High	Master Protein	P07948	Tyrosine-protein kinase Lyn OS=Homo sapiens (Human) OX=9606 GN=LYN PE=1 SV=3	LYN	58,5	1,17	0,569796488
FALSO	High	None	Q96QV6	Histone H2A type 1-A OS=Homo sapiens (Human) OX=9606 GN=HIST1H2AA PE=1 SV=3	HIST1H2AA	14,2	1,17	0,536085623
FALSO	High	Master Protein	P02751	Fibronectin OS=Homo sapiens (Human) OX=9606 GN=FN1 PE=1 SV=5	FN1	272	1,16	0,205756173
FALSO	High	Master Protein	P24666	Low molecular weight phosphotyrosine protein phosphatase OS=Homo sapiens (Human) OX=9606 GN=ACP1	ACP1	18	1,16	0,596849005
FALSO	High	Master Protein	P14678	Small nuclear ribonucleoprotein-associated proteins B and B' OS=Homo sapiens (Human) OX=9606 GN=SNRF	SNRPB	24,6	1,16	0,536566618
FALSO	High	Master Protein Candidat	P63162	Small nuclear ribonucleoprotein-associated protein N OS=Homo sapiens (Human) OX=9606 GN=SNRPN PE=1	SNRPN	24,6	1,16	0,536566618
FALSO	High	Master Protein	P00450	Ceruloplasmin OS=Homo sapiens (Human) OX=9606 GN=CP PE=1 SV=1	CP	122	1,16	0,201343724
FALSO	High	Master Protein	O15145	Actin-related protein 2/3 complex subunit 3 OS=Homo sapiens (Human) OX=9606 GN=ARPC3 PE=1 SV=3	ARPC3	20,5	1,16	0,473756652
FALSO	High	Master Protein	P28062	Proteasome subunit beta type-8 OS=Homo sapiens (Human) OX=9606 GN=PSMB8 PE=1 SV=3	PSMB8	30,3	1,16	0,540260761
FALSO	Medium	Master Protein	Q9ULV0	Unconventional myosin-Vb OS=Homo sapiens (Human) OX=9606 GN=MYO5B PE=1 SV=3	MYO5B	214	1,16	0,629968422
FALSO	High	Master Protein	P09651	Heterogeneous nuclear ribonucleoprotein A1 OS=Homo sapiens (Human) OX=9606 GN=HNRNPA1 PE=1 SV=5	HNRNPA1	38,7	1,16	0,414386009
FALSO	High	Master Protein	P29466	Caspase-1 OS=Homo sapiens (Human) OX=9606 GN=CASP1 PE=1 SV=1	CASP1	45,1	1,16	0,577364565
FALSO	High	Master Protein	P05154	Plasma serine protease inhibitor OS=Homo sapiens (Human) OX=9606 GN=SERPINA5 PE=1 SV=3	SERPINA5	45,6	1,15	0,638707278
FALSO	High	Master Protein	P01743	Immunoglobulin heavy variable 1-46 OS=Homo sapiens (Human) OX=9606 GN=IGHV1-46 PE=1 SV=2	IGHV1-46	12,9	1,15	0,526544194

FALSO	High	Master Protein	Q12882	Dihydropyrimidine dehydrogenase [NADP(+)] OS=Homo sapiens (Human) OX=9606 GN=DPYD PE=1 SV=2	DPYD	111	1,15	0,738865142
FALSO	High	Master Protein	P52597	Heterogeneous nuclear ribonucleoprotein F OS=Homo sapiens (Human) OX=9606 GN=HNRNPF PE=1 SV=3	HNRNPF	45,6	1,15	0,446383534
FALSO	High	Master Protein	P48595	Serpin B10 OS=Homo sapiens (Human) OX=9606 GN=SERPINB10 PE=1 SV=1	SERPINB10	45,4	1,15	0,56821083
FALSO	High	Master Protein	Q15257	Serine/threonine-protein phosphatase 2A activator OS=Homo sapiens (Human) OX=9606 GN=PTPA PE=1 SV=:	PTPA	40,6	1,15	0,458261511
FALSO	High	Master Protein	P43490	Nicotinamide phosphoribosyltransferase OS=Homo sapiens (Human) OX=9606 GN=NAMPT PE=1 SV=1	NAMPT	55,5	1,15	0,636731396
FALSO	High	Master Protein	P31150	Rab GDP dissociation inhibitor alpha OS=Homo sapiens (Human) OX=9606 GN=GDI1 PE=1 SV=2	GDI1	50,6	1,15	0,535034042
FALSO	High	Master Protein	Q96FW1	Ubiquitin thioesterase OTUB1 OS=Homo sapiens (Human) OX=9606 GN=OTUB1 PE=1 SV=2	OTUB1	31,3	1,15	0,59379977
FALSO	High	Master Protein	P0DP04	Immunoglobulin heavy variable 3-43D OS=Homo sapiens (Human) OX=9606 GN=IGHV3-43D PE=3 SV=1	IGHV3-43D	13	1,14	0,626050192
FALSO	High	Master Protein	Q96C86	m7GpppX diphosphatase OS=Homo sapiens (Human) OX=9606 GN=DCPS PE=1 SV=2	DCPS	38,6	1,14	0,688983138
FALSO	High	Master Protein	Q9H0Q0	Protein FAM49A OS=Homo sapiens (Human) OX=9606 GN=FAM49A PE=2 SV=1	FAM49A	37,3	1,14	0,652728427
FALSO	High	Master Protein	A0A0J9YX35	Immunoglobulin heavy variable 3-64D OS=Homo sapiens (Human) OX=9606 GN=IGHV3-64D PE=3 SV=1	IGHV3-64D	12,8	1,14	0,42527175
FALSO	High	Master Protein	Q07020	60S ribosomal protein L18 OS=Homo sapiens (Human) OX=9606 GN=RPL18 PE=1 SV=2	RPL18	21,6	1,14	0,70028603
FALSO	High	Master Protein	P24158	Myeloblastin OS=Homo sapiens (Human) OX=9606 GN=PRTN3 PE=1 SV=3	PRTN3	27,8	1,14	0,25761863
FALSO	High	Master Protein	P27797	Calreticulin OS=Homo sapiens (Human) OX=9606 GN=CALR PE=1 SV=1	CALR	48,1	1,14	0,519916867
FALSO	High	Master Protein	P46459	Vesicle-fusing ATPase OS=Homo sapiens (Human) OX=9606 GN=NSF PE=1 SV=3	NSF	82,5	1,14	0,610874615
FALSO	High	Master Protein	O75083	WD repeat-containing protein 1 OS=Homo sapiens (Human) OX=9606 GN=WDR1 PE=1 SV=4	WDR1	66,2	1,13	0,554928694
FALSO	High	Master Protein	P38117	Electron transfer flavoprotein subunit beta OS=Homo sapiens (Human) OX=9606 GN=ETFB PE=1 SV=3	ETFB	27,8	1,13	0,538608134
FALSO	High	Master Protein	P07686	Beta-hexosaminidase subunit beta OS=Homo sapiens (Human) OX=9606 GN=HEXB PE=1 SV=3	HEXB	63,1	1,13	0,5631835
FALSO	High	Master Protein	P50995	Annexin A11 OS=Homo sapiens (Human) OX=9606 GN=ANXA11 PE=1 SV=1	ANXA11	54,4	1,13	0,602183022
FALSO	Medium	Master Protein	Q96AZ6	Interferon-stimulated gene 20 kDa protein OS=Homo sapiens (Human) OX=9606 GN=ISG20 PE=1 SV=2	ISG20	20,4	1,13	0,568033206
FALSO	High	Master Protein	O14579	Coatomer subunit epsilon OS=Homo sapiens (Human) OX=9606 GN=COPE PE=1 SV=3	COPE	34,5	1,13	0,763232922
FALSO	High	Master Protein	P11215	Integrin alpha-M OS=Homo sapiens (Human) OX=9606 GN=ITGAM PE=1 SV=2	ITGAM	127	1,13	0,700095444
FALSO	High	Master Protein	P25815	Protein S100-P OS=Homo sapiens (Human) OX=9606 GN=S100P PE=1 SV=2	S100P	10,4	1,13	0,665338668
FALSO	High	Master Protein	P49458	Signal recognition particle 9 kDa protein OS=Homo sapiens (Human) OX=9606 GN=SRP9 PE=1 SV=2	SRP9; SRP9P1	10,1	1,13	0,568287913
FALSO	High	Master Protein	Q15057	Arf-GAP with coiled-coil, ANK repeat and PH domain-containing protein 2 OS=Homo sapiens (Human) OX=96	ACAP2	88	1,12	0,660679279
FALSO	High	Master Protein	Q01518	Adenylyl cyclase-associated protein 1 OS=Homo sapiens (Human) OX=9606 GN=CAP1 PE=1 SV=5	CAP1	51,9	1,12	0,50527783
FALSO	High	Master Protein	P10720	Platelet factor 4 variant OS=Homo sapiens (Human) OX=9606 GN=PF4V1 PE=1 SV=1	PF4V1	11,5	1,12	0,771776811
FALSO	High	Master Protein Candidat	P02776	Platelet factor 4 OS=Homo sapiens (Human) OX=9606 GN=PF4 PE=1 SV=2	PF4	10,8	1,12	0,771776811
FALSO	High	Master Protein	P07360	Complement component C8 gamma chain OS=Homo sapiens (Human) OX=9606 GN=C8G PE=1 SV=3	C8G	22,3	1,12	0,356512763
FALSO	High	Master Protein	P08133	Annexin A6 OS=Homo sapiens (Human) OX=9606 GN=ANXA6 PE=1 SV=3	ANXA6	75,8	1,12	0,637513938
FALSO	High	Master Protein	Q9Y333	U6 snRNA-associated Sm-like protein LSm2 OS=Homo sapiens (Human) OX=9606 GN=LSM2 PE=1 SV=1	LSM2	10,8	1,12	0,604213228
FALSO	High	Master Protein	P25789	Proteasome subunit alpha type-4 OS=Homo sapiens (Human) OX=9606 GN=PSMA4 PE=1 SV=1	PSMA4	29,5	1,12	0,608195169
FALSO	High	Master Protein	P35754	Glutaredoxin-1 OS=Homo sapiens (Human) OX=9606 GN=GLRX PE=1 SV=2	GLRX	11,8	1,12	0,550612682

FALSO	High	Master Protein	Q9UBQ7	Glyoxylate reductase/hydroxypyruvate reductase OS=Homo sapiens (Human) OX=9606 GN=GRHPR PE=1 SV=	GRHPR	35,6	1,12	0,660703118
FALSO	Medium	Master Protein	Q9H939	Proline-serine-threonine phosphatase-interacting protein 2 OS=Homo sapiens (Human) OX=9606 GN=PSTPIP	PSTPIP2	38,8	1,11	0,619512942
FALSO	High	Master Protein	P62318	Small nuclear ribonucleoprotein Sm D3 OS=Homo sapiens (Human) OX=9606 GN=SNRPD3 PE=1 SV=1	SNRPD3	13,9	1,11	0,558289179
FALSO	High	Master Protein	P63313	Thymosin beta-10 OS=Homo sapiens (Human) OX=9606 GN=TMSB10 PE=1 SV=2	TMSB10	5	1,11	0,750659052
FALSO	High	Master Protein	P22626	Heterogeneous nuclear ribonucleoproteins A2/B1 OS=Homo sapiens (Human) OX=9606 GN=HNRNPA2B1 PE	HNRNPA2B1	37,4	1,11	0,495265903
FALSO	High	Master Protein	P25786	Proteasome subunit alpha type-1 OS=Homo sapiens (Human) OX=9606 GN=PSMA1 PE=1 SV=1	PSMA1	29,5	1,11	0,635540673
FALSO	High	Master Protein	P01024	Complement C3 OS=Homo sapiens (Human) OX=9606 GN=C3 PE=1 SV=2	C3	187	1,11	0,248779754
FALSO	High	Master Protein	P09211	Glutathione S-transferase P OS=Homo sapiens (Human) OX=9606 GN=GSTP1 PE=1 SV=2	GSTP1	23,3	1,11	0,450536047
FALSO	High	Master Protein	P46976	Glycogenin-1 OS=Homo sapiens (Human) OX=9606 GN=GYG1 PE=1 SV=4	GYG1	39,4	1,10	0,777967966
FALSO	High	Master Protein	P34931	Heat shock 70 kDa protein 1-like OS=Homo sapiens (Human) OX=9606 GN=HSPA1L PE=1 SV=2	HSPA1L	70,3	1,10	0,84833474
FALSO	High	Master Protein	P04899	Guanine nucleotide-binding protein G(i) subunit alpha-2 OS=Homo sapiens (Human) OX=9606 GN=GNAI2 PE	GNAI2	40,4	1,10	0,722279589
FALSO	High	Master Protein	P01860	Immunoglobulin heavy constant gamma 3 OS=Homo sapiens (Human) OX=9606 GN=IGHG3 PE=1 SV=2	IGHG3	41,3	1,10	0,523191852
FALSO	High	Master Protein	P25787	Proteasome subunit alpha type-2 OS=Homo sapiens (Human) OX=9606 GN=PSMA2 PE=1 SV=2	PSMA2	25,9	1,10	0,476388666
FALSO	High	Master Protein	P08236	Beta-glucuronidase OS=Homo sapiens (Human) OX=9606 GN=GUSB PE=1 SV=2	GUSB	74,7	1,10	0,693366395
FALSO	Medium	Master Protein	O76096	Cystatin-F OS=Homo sapiens (Human) OX=9606 GN=CST7 PE=1 SV=1	CST7	16,4	1,10	0,658914364
FALSO	High	None	A0A075B6R9	Probable non-functional immunoglobulin kappa variable 2D-24 OS=Homo sapiens (Human) OX=9606 GN=IG	IGKV2D-24	13,1	1,09	0,59473688
FALSO	High	Master Protein	Q9UBG3	Cornulin OS=Homo sapiens (Human) OX=9606 GN=CRNN PE=1 SV=1	CRNN	53,5	1,09	0,808382988
FALSO	High	Master Protein	Q9P1F3	Costars family protein ABRACL OS=Homo sapiens (Human) OX=9606 GN=ABRACL PE=1 SV=1	ABRACL	9,1	1,09	0,843235263
FALSO	High	Master Protein	A0A0C4DH68	Immunoglobulin kappa variable 2-24 OS=Homo sapiens (Human) OX=9606 GN=IGKV2-24 PE=3 SV=1	IGKV2-24	13,1	1,09	0,604993122
FALSO	High	Master Protein	Q14677	Clathrin interactor 1 OS=Homo sapiens (Human) OX=9606 GN=CLINT1 PE=1 SV=1	CLINT1	68,2	1,09	0,742661117
FALSO	High	Master Protein	A0A0C4DH38	Immunoglobulin heavy variable 5-51 OS=Homo sapiens (Human) OX=9606 GN=IGHV5-51 PE=3 SV=1	IGHV5-51	12,7	1,09	0,7303249
FALSO	High	Master Protein	Q03591	Complement factor H-related protein 1 OS=Homo sapiens (Human) OX=9606 GN=CFHR1 PE=1 SV=2	CFHR1	37,6	1,09	0,69209172
FALSO	Medium	Master Protein	P26439	3 beta-hydroxysteroid dehydrogenase/Delta 5-->4-isomerase type 2 OS=Homo sapiens (Human) OX=9606 GN	HSD3B2	42	1,09	0,776693485
FALSO	Medium	Master Protein Candidat	P14060	3 beta-hydroxysteroid dehydrogenase/Delta 5-->4-isomerase type 1 OS=Homo sapiens (Human) OX=9606 GN	HSD3B1	42,2	1,09	0,776693485
FALSO	High	Master Protein	P09104	Gamma-enolase OS=Homo sapiens (Human) OX=9606 GN=ENO2 PE=1 SV=3	ENO2	47,2	1,09	0,828575229
FALSO	High	Master Protein	P07203	Glutathione peroxidase 1 OS=Homo sapiens (Human) OX=9606 GN=GPX1 PE=1 SV=4	GPX1	22,1	1,09	0,623762391
FALSO	High	Master Protein	Q9NQR4	Omega-amidase NIT2 OS=Homo sapiens (Human) OX=9606 GN=NIT2 PE=1 SV=1	NIT2	30,6	1,09	0,647270294
FALSO	High	Master Protein	P06396	Gelsolin OS=Homo sapiens (Human) OX=9606 GN=GSN PE=1 SV=1	GSN	85,6	1,08	0,49832412
FALSO	High	Master Protein	O96019	Actin-like protein 6A OS=Homo sapiens (Human) OX=9606 GN=ACTL6A PE=1 SV=1	ACTL6A	47,4	1,08	0,655550457
FALSO	High	Master Protein	O14618	Copper chaperone for superoxide dismutase OS=Homo sapiens (Human) OX=9606 GN=CCS PE=1 SV=1	CCS	29	1,08	0,780785334
FALSO	High	Master Protein	O14950	Myosin regulatory light chain 12B OS=Homo sapiens (Human) OX=9606 GN=MYL12B PE=1 SV=2	MYL12B; MYL12A	19,8	1,08	0,746291916
FALSO	High	Master Protein Candidat	P19105	Myosin regulatory light chain 12A OS=Homo sapiens (Human) OX=9606 GN=MYL12A PE=1 SV=2	MYL12A	19,8	1,08	0,746291916
FALSO	High	Master Protein	P02649	Apolipoprotein E OS=Homo sapiens (Human) OX=9606 GN=APOE PE=1 SV=1	APOE	36,1	1,08	0,691836734

FALSO	High	Master Protein	P29034	Protein S100-A2 OS=Homo sapiens (Human) OX=9606 GN=S100A2 PE=1 SV=3	S100A2	11,1	1,08	0,678967755
FALSO	High	Master Protein	P32926	Desmoglein-3 OS=Homo sapiens (Human) OX=9606 GN=DSG3 PE=1 SV=2	DSG3	108	1,08	0,796472875
FALSO	High	Master Protein	P07384	Calpain-1 catalytic subunit OS=Homo sapiens (Human) OX=9606 GN=CAPN1 PE=1 SV=1	CAPN1	81,8	1,07	0,685142099
FALSO	Medium	Master Protein	P02741	C-reactive protein OS=Homo sapiens (Human) OX=9606 GN=CRP PE=1 SV=1	CRP	25	1,07	0,856914216
FALSO	High	Master Protein Candidat	P28161	Glutathione S-transferase Mu 2 OS=Homo sapiens (Human) OX=9606 GN=GSTM2 PE=1 SV=2	GSTM2	25,7	1,07	0,843615445
FALSO	High	Master Protein Candidat	P09488	Glutathione S-transferase Mu 1 OS=Homo sapiens (Human) OX=9606 GN=GSTM1 PE=1 SV=3	GSTM1	25,7	1,07	0,843615445
FALSO	High	Master Protein	Q03013	Glutathione S-transferase Mu 4 OS=Homo sapiens (Human) OX=9606 GN=GSTM4 PE=1 SV=3	GSTM4	25,5	1,07	0,843615445
FALSO	High	Master Protein	P28799	Progranulin OS=Homo sapiens (Human) OX=9606 GN=GRN PE=1 SV=2	GRN	63,5	1,07	0,718132254
FALSO	High	Master Protein	P38646	Stress-70 protein, mitochondrial OS=Homo sapiens (Human) OX=9606 GN=HSPA9 PE=1 SV=2	HSPA9	73,6	1,07	0,822872656
FALSO	High	Master Protein	Q5TEC6	Histone H3 OS=Homo sapiens (Human) OX=9606 GN=H3-2 PE=1 SV=1	HIST2H3PS2	15,4	1,07	0,796552497
FALSO	High	None	A0A075B6S6	Immunoglobulin kappa variable 2D-30 OS=Homo sapiens (Human) OX=9606 GN=IGKV2D-30 PE=3 SV=1	IGKV2D-30	13,2	1,07	0,733158037
FALSO	High	Master Protein	P30520	Adenylosuccinate synthetase isozyme 2 OS=Homo sapiens (Human) OX=9606 GN=ADSS2 PE=1 SV=3	ADSS	50,1	1,07	0,702674002
FALSO	High	Master Protein	Q10588	ADP-ribosyl cyclase/cyclic ADP-ribose hydrolase 2 OS=Homo sapiens (Human) OX=9606 GN=BST1 PE=1 SV=2	BST1	35,7	1,06	0,650689669
FALSO	Medium	Master Protein	P17927	Complement receptor type 1 OS=Homo sapiens (Human) OX=9606 GN=CR1 PE=1 SV=3	CR1	224	1,06	0,889192751
FALSO	Medium	Master Protein Candidat	Q2VPA4	Complement component receptor 1-like protein OS=Homo sapiens (Human) OX=9606 GN=CR1L PE=1 SV=3	CR1L	62,7	1,06	0,889192751
FALSO	High	None	P0DP07	Immunoglobulin heavy variable 4-31 OS=Homo sapiens (Human) OX=9606 GN=IGHV4-31 PE=3 SV=1	IGHV4-31	13,1	1,06	0,699353193
FALSO	High	None	A0A087WSY4	Immunoglobulin heavy variable 4-30-2 OS=Homo sapiens (Human) OX=9606 GN=IGHV4-30-2 PE=3 SV=1	IGHV4-31	13	1,06	0,699353193
FALSO	High	Master Protein	Q6IA69	Glutamine-dependent NAD(+) synthetase OS=Homo sapiens (Human) OX=9606 GN=NADSYN1 PE=1 SV=3	NADSYN1	79,2	1,06	0,843312776
FALSO	High	Master Protein	O75368	SH3 domain-binding glutamic acid-rich-like protein OS=Homo sapiens (Human) OX=9606 GN=SH3BGRL PE=1	SH3BGRL	12,8	1,06	0,799021363
FALSO	Medium	None	Q58FG0	Putative heat shock protein HSP 90-alpha A5 OS=Homo sapiens (Human) OX=9606 GN=HSP90AA5P PE=2 SV	HSP90AA5P	38,7	1,06	0,828799261
FALSO	High	Master Protein	P60660	Myosin light polypeptide 6 OS=Homo sapiens (Human) OX=9606 GN=MYL6 PE=1 SV=2	MYL6	16,9	1,06	0,800574834
FALSO	High	Master Protein	O75223	Gamma-glutamylcyclotransferase OS=Homo sapiens (Human) OX=9606 GN=GGCT PE=1 SV=1	GGCT	21	1,06	0,836337096
FALSO	High	Master Protein	P07108	Acyl-CoA-binding protein OS=Homo sapiens (Human) OX=9606 GN=DBI PE=1 SV=2	DBI	10	1,06	0,833366911
FALSO	High	Master Protein	P09769	Tyrosine-protein kinase Fgr OS=Homo sapiens (Human) OX=9606 GN=FGR PE=1 SV=2	FGR	59,4	1,06	0,846524368
FALSO	High	Master Protein	P31943	Heterogeneous nuclear ribonucleoprotein H OS=Homo sapiens (Human) OX=9606 GN=HNRNPH1 PE=1 SV=4	HNRNPH1	49,2	1,06	0,746891407
FALSO	High	None	P55795	Heterogeneous nuclear ribonucleoprotein H2 OS=Homo sapiens (Human) OX=9606 GN=HNRNPH2 PE=1 SV=	HNRNPH2	49,2	1,06	0,746891407
FALSO	High	None	O95758	Polypyrimidine tract-binding protein 3 OS=Homo sapiens (Human) OX=9606 GN=PTBP3 PE=1 SV=2	PTBP3	59,7	1,06	0,767533454
FALSO	High	Master Protein	P63279	SUMO-conjugating enzyme UBC9 OS=Homo sapiens (Human) OX=9606 GN=UBE2I PE=1 SV=1	UBE2I	18	1,06	0,832174356
FALSO	High	Master Protein	Q16719	Kynureninase OS=Homo sapiens (Human) OX=9606 GN=KYNU PE=1 SV=1	KYNU	52,3	1,05	0,819360323
FALSO	High	Master Protein	P46940	Ras GTPase-activating-like protein IQGAP1 OS=Homo sapiens (Human) OX=9606 GN=IQGAP1 PE=1 SV=1	IQGAP1	189	1,05	0,818310242
FALSO	High	Master Protein	O43765	Small glutamine-rich tetratricopeptide repeat-containing protein alpha OS=Homo sapiens (Human) OX=9606 (	SGTA	34	1,05	0,794898811
FALSO	High	Master Protein	P01031	Complement C5 OS=Homo sapiens (Human) OX=9606 GN=C5 PE=1 SV=4	C5	188	1,05	0,545713461
FALSO	High	Master Protein	Q13404	Ubiquitin-conjugating enzyme E2 variant 1 OS=Homo sapiens (Human) OX=9606 GN=UBE2V1 PE=1 SV=2	UBE2V1	16,5	1,05	0,864936178

FALSO	High	Master Protein	P27824	Calnexin OS=Homo sapiens (Human) OX=9606 GN=CANX PE=1 SV=2	CANX	67,5	1,05	0,771503783
FALSO	High	Master Protein	P51665	26S proteasome non-ATPase regulatory subunit 7 OS=Homo sapiens (Human) OX=9606 GN=PSMD7 PE=1 SV	PSMD7	37	1,05	0,874650021
FALSO	Medium	Master Protein	P26196	Probable ATP-dependent RNA helicase DDX6 OS=Homo sapiens (Human) OX=9606 GN=DDX6 PE=1 SV=2	DDX6	54,4	1,05	0,891660798
FALSO	High	Master Protein	Q15027	Arf-GAP with coiled-coil, ANK repeat and PH domain-containing protein 1 OS=Homo sapiens (Human) OX=96	ACAP1	81,5	1,05	0,89312298
FALSO	High	Master Protein	P22352	Glutathione peroxidase 3 OS=Homo sapiens (Human) OX=9606 GN=GPX3 PE=1 SV=2	GPX3	25,5	1,05	0,746615606
FALSO	High	Master Protein	A0A0B4J1Y9	Immunoglobulin heavy variable 3-72 OS=Homo sapiens (Human) OX=9606 GN=IGHV3-72 PE=3 SV=1	IGHV3-72	13,2	1,05	0,839259454
FALSO	High	Master Protein	P49721	Proteasome subunit beta type-2 OS=Homo sapiens (Human) OX=9606 GN=PSMB2 PE=1 SV=1	PSMB2	22,8	1,05	0,834836402
FALSO	High	Master Protein Candidat	O60814	Histone H2B type 1-K OS=Homo sapiens (Human) OX=9606 GN=H2BC12 PE=1 SV=3	HIST1H2BK	13,9	1,05	0,813024914
FALSO	High	Master Protein Candidat	Q93079	Histone H2B type 1-H OS=Homo sapiens (Human) OX=9606 GN=HIST1H2BH PE=1 SV=3	HIST1H2BH	13,9	1,05	0,813024914
FALSO	High	Master Protein	Q5QNW6	Histone H2B type 2-F OS=Homo sapiens (Human) OX=9606 GN=HIST2H2BF PE=1 SV=3	HIST2H2BF	13,9	1,05	0,813024914
FALSO	High	Master Protein Candidat	Q99880	Histone H2B type 1-L OS=Homo sapiens (Human) OX=9606 GN=H2BC13 PE=1 SV=3	HIST1H2BL	13,9	1,05	0,813024914
FALSO	High	Master Protein Candidat	P58876	Histone H2B type 1-D OS=Homo sapiens (Human) OX=9606 GN=HIST1H2BD PE=1 SV=2	HIST1H2BD	13,9	1,05	0,813024914
FALSO	High	Master Protein Candidat	P62807	Histone H2B type 1-C/E/F/G/I OS=Homo sapiens (Human) OX=9606 GN=H2BC6 PE=1 SV=4	HIST1H2BI	13,9	1,05	0,813024914
FALSO	High	Master Protein Candidat	Q99877	Histone H2B type 1-N OS=Homo sapiens (Human) OX=9606 GN=H2BC15 PE=1 SV=3	HIST1H2BN	13,9	1,05	0,813024914
FALSO	High	Master Protein Candidat	Q99879	Histone H2B type 1-M OS=Homo sapiens (Human) OX=9606 GN=H2BC14 PE=1 SV=3	HIST1H2BM	14	1,05	0,813024914
FALSO	High	None	P57053	Histone H2B type F-S OS=Homo sapiens (Human) OX=9606 GN=H2BFS PE=1 SV=2	H2BFS	13,9	1,05	0,814783193
FALSO	High	Master Protein	Q16555	Dihydropyrimidinase-related protein 2 OS=Homo sapiens (Human) OX=9606 GN=DPYSL2 PE=1 SV=1	DPYSL2	62,3	1,04	0,872275051
FALSO	High	Master Protein	O75882	Attractin OS=Homo sapiens (Human) OX=9606 GN=ATRN PE=1 SV=2	ATRN	158	1,04	0,776705324
FALSO	High	Master Protein	P36955	Pigment epithelium-derived factor OS=Homo sapiens (Human) OX=9606 GN=SERPINF1 PE=1 SV=4	SERPINF1	46,3	1,04	0,899882203
FALSO	Medium	Master Protein	Q5SYB0	FERM and PDZ domain-containing protein 1 OS=Homo sapiens (Human) OX=9606 GN=FRMPD1 PE=1 SV=1	FRMPD1	173	1,04	0,825033797
FALSO	High	Master Protein	P06737	Glycogen phosphorylase, liver form OS=Homo sapiens (Human) OX=9606 GN=PYGL PE=1 SV=4	PYGL	97,1	1,04	0,878099247
FALSO	High	Master Protein	Q9NR12	PDZ and LIM domain protein 7 OS=Homo sapiens (Human) OX=9606 GN=PDLIM7 PE=1 SV=1	PDLIM7	49,8	1,04	0,884564118
FALSO	High	Master Protein	P05089	Arginase-1 OS=Homo sapiens (Human) OX=9606 GN=ARG1 PE=1 SV=2	ARG1	34,7	1,04	0,860658498
FALSO	High	Master Protein	P01023	Alpha-2-macroglobulin OS=Homo sapiens (Human) OX=9606 GN=A2M PE=1 SV=3	A2M	163	1,04	0,748803256
FALSO	High	Master Protein	P09960	Leukotriene A-4 hydrolase OS=Homo sapiens (Human) OX=9606 GN=LTA4H PE=1 SV=2	LTA4H	69,2	1,03	0,885690863
FALSO	High	Master Protein	P05198	Eukaryotic translation initiation factor 2 subunit 1 OS=Homo sapiens (Human) OX=9606 GN=EIF2S1 PE=1 SV=	EIF2S1	36,1	1,03	0,855686606
FALSO	High	Master Protein	P49773	Histidine triad nucleotide-binding protein 1 OS=Homo sapiens (Human) OX=9606 GN=HINT1 PE=1 SV=2	HINT1	13,8	1,03	0,893174414
FALSO	Medium	Master Protein	P14854	Cytochrome c oxidase subunit 6B1 OS=Homo sapiens (Human) OX=9606 GN=COX6B1 PE=1 SV=2	COX6B1	10,2	1,03	0,912534597
FALSO	High	Master Protein	P08246	Neutrophil elastase OS=Homo sapiens (Human) OX=9606 GN=ELANE PE=1 SV=1	ELANE	28,5	1,03	0,842907043
FALSO	High	Master Protein	Q15485	Ficolin-2 OS=Homo sapiens (Human) OX=9606 GN=FCN2 PE=1 SV=2	FCN2	34	1,03	0,914585205
FALSO	Medium	Master Protein	P67775	Serine/threonine-protein phosphatase 2A catalytic subunit alpha isoform OS=Homo sapiens (Human) OX=96(	PPP2CA	35,6	1,03	0,955581428
FALSO	High	Master Protein	O43451	Maltase-glucoamylase, intestinal OS=Homo sapiens (Human) OX=9606 GN=MGAM PE=1 SV=5	MGAM	210	1,03	0,921663725
FALSO	High	Master Protein	O15144	Actin-related protein 2/3 complex subunit 2 OS=Homo sapiens (Human) OX=9606 GN=ARPC2 PE=1 SV=1	ARPC2	34,3	1,03	0,923139923

FALSO	High	Master Protein	P61160	Actin-related protein 2 OS=Homo sapiens (Human) OX=9606 GN=ACTR2 PE=1 SV=1	ACTR2	44,7	1,02	0,898826601
FALSO	High	Master Protein	P52788	Spermine synthase OS=Homo sapiens (Human) OX=9606 GN=SMS PE=1 SV=2	SMS	41,2	1,02	0,941237552
FALSO	High	Master Protein	P09622	Dihydrolipoyl dehydrogenase, mitochondrial OS=Homo sapiens (Human) OX=9606 GN=DLD PE=1 SV=2	DLD	54,1	1,02	0,927228481
FALSO	High	Master Protein	P62312	U6 snRNA-associated Sm-like protein LSm6 OS=Homo sapiens (Human) OX=9606 GN=LSM6 PE=1 SV=1	LSM6	9,1	1,02	0,934486751
FALSO	High	Master Protein	P11142	Heat shock cognate 71 kDa protein OS=Homo sapiens (Human) OX=9606 GN=HSPA8 PE=1 SV=1	HSPA8	70,9	1,02	0,884089614
FALSO	High	Master Protein	P49902	Cytosolic purine 5'-nucleotidase OS=Homo sapiens (Human) OX=9606 GN=NT5C2 PE=1 SV=1	NT5C2	64,9	1,02	0,954306918
FALSO	High	Master Protein	Q6XQN6	Nicotinate phosphoribosyltransferase OS=Homo sapiens (Human) OX=9606 GN=NAPRT PE=1 SV=2	NAPRT	57,5	1,02	0,936794367
FALSO	High	Master Protein	P22894	Neutrophil collagenase OS=Homo sapiens (Human) OX=9606 GN=MMP8 PE=1 SV=1	MMP8	53,4	1,02	0,916373118
FALSO	Medium	Master Protein	Q96CW1	AP-2 complex subunit mu OS=Homo sapiens (Human) OX=9606 GN=AP2M1 PE=1 SV=2	AP2M1	49,6	1,02	0,959361541
FALSO	High	Master Protein	P47756	F-actin-capping protein subunit beta OS=Homo sapiens (Human) OX=9606 GN=CAPZB PE=1 SV=4	CAPZB	31,3	1,02	0,935135489
FALSO	High	Master Protein	P08697	Alpha-2-antiplasmin OS=Homo sapiens (Human) OX=9606 GN=SERPINF2 PE=1 SV=3	SERPINF2	54,5	1,02	0,949002641
FALSO	High	Master Protein	P23368	NAD-dependent malic enzyme, mitochondrial OS=Homo sapiens (Human) OX=9606 GN=ME2 PE=1 SV=1	ME2	65,4	1,02	0,950340267
FALSO	High	Master Protein	P62820	Ras-related protein Rab-1A OS=Homo sapiens (Human) OX=9606 GN=RAB1A PE=1 SV=3	RAB1A	22,7	1,02	0,950996521
FALSO	High	Master Protein	Q9BUL8	Programmed cell death protein 10 OS=Homo sapiens (Human) OX=9606 GN=PDCD10 PE=1 SV=1	PDCD10	24,7	1,02	0,938895674
FALSO	High	Master Protein	P53634	Dipeptidyl peptidase 1 OS=Homo sapiens (Human) OX=9606 GN=CTSC PE=1 SV=2	CTSC	51,8	1,02	0,936846981
FALSO	High	Master Protein	A0A0B4J1U3	Immunoglobulin lambda variable 1-36 OS=Homo sapiens (Human) OX=9606 GN=IGLV1-36 PE=1 SV=5	IGLV1-36	12,5	1,02	0,974365098
FALSO	High	Master Protein	P55735	Protein SEC13 homolog OS=Homo sapiens (Human) OX=9606 GN=SEC13 PE=1 SV=3	SEC13	35,5	1,02	0,970217455
FALSO	High	Master Protein	P00558	Phosphoglycerate kinase 1 OS=Homo sapiens (Human) OX=9606 GN=PGK1 PE=1 SV=3	PGK1	44,6	1,01	0,925175887
FALSO	High	Master Protein	A0A075B6K4	Immunoglobulin lambda variable 3-10 OS=Homo sapiens (Human) OX=9606 GN=IGLV3-10 PE=3 SV=2	IGLV3-10	12,4	1,01	0,939871721
FALSO	High	Master Protein	O14791	Apolipoprotein L1 OS=Homo sapiens (Human) OX=9606 GN=APOL1 PE=1 SV=5	APOL1	43,9	1,01	0,962286246
FALSO	High	Master Protein	P04430	Immunoglobulin kappa variable 1-16 OS=Homo sapiens (Human) OX=9606 GN=IGKV1-16 PE=1 SV=2	IGKV1-16	12,6	1,01	0,96778728
FALSO	High	Master Protein	O60506	Heterogeneous nuclear ribonucleoprotein Q OS=Homo sapiens (Human) OX=9606 GN=SYNCRIP PE=1 SV=2	SYNCRIP	69,6	1,01	0,962789248
FALSO	High	Master Protein	P05160	Coagulation factor XIII B chain OS=Homo sapiens (Human) OX=9606 GN=F13B PE=1 SV=3	F13B	75,5	1,01	0,948134822
FALSO	High	Master Protein	Q15691	Microtubule-associated protein RP/EB family member 1 OS=Homo sapiens (Human) OX=9606 GN=MAPRE1 P	MAPRE1	30	1,01	0,92516074
FALSO	High	Master Protein	P07195	L-lactate dehydrogenase B chain OS=Homo sapiens (Human) OX=9606 GN=LDHB PE=1 SV=2	LDHB	36,6	1,01	0,965676829
FALSO	High	None	Q9Y281	Cofilin-2 OS=Homo sapiens (Human) OX=9606 GN=CFL2 PE=1 SV=1	CFL2	18,7	1,01	0,958921636
FALSO	High	Master Protein	P06276	Cholinesterase OS=Homo sapiens (Human) OX=9606 GN=BCHE PE=1 SV=1	BCHE	68,4	1,01	0,973510507
FALSO	High	Master Protein	P54577	Tyrosine--tRNA ligase, cytoplasmic OS=Homo sapiens (Human) OX=9606 GN=YARS1 PE=1 SV=4	YARS	59,1	1,01	0,979319542
FALSO	High	Master Protein	O43390	Heterogeneous nuclear ribonucleoprotein R OS=Homo sapiens (Human) OX=9606 GN=HNRNPR PE=1 SV=1	HNRNPR	70,9	1,01	0,979711986
FALSO	Medium	Master Protein Candidat	Q96A72	Protein mago nashi homolog 2 OS=Homo sapiens (Human) OX=9606 GN=MAGOHB PE=1 SV=1	MAGOHB	17,3	1,00	0,99106948
FALSO	Medium	Master Protein	P61326	Protein mago nashi homolog OS=Homo sapiens (Human) OX=9606 GN=MAGOH PE=1 SV=1	MAGOH	17,2	1,00	0,99106948
FALSO	High	Master Protein	O15143	Actin-related protein 2/3 complex subunit 1B OS=Homo sapiens (Human) OX=9606 GN=ARPC1B PE=1 SV=3	ARPC1B	40,9	1,00	0,988434775
FALSO	High	None	O95626	Acidic leucine-rich nuclear phosphoprotein 32 family member D OS=Homo sapiens (Human) OX=9606 GN=AI	ANP32D	14,8	1,00	0,993548744

FALSO	High	None	O43423	Acidic leucine-rich nuclear phosphoprotein 32 family member C OS=Homo sapiens (Human) OX=9606 GN=AN	ANP32C	26,7	1,00	0,993548744
FALSO	High	Master Protein	Q12907	Vesicular integral-membrane protein VIP36 OS=Homo sapiens (Human) OX=9606 GN=LMAN2 PE=1 SV=1	LMAN2	40,2	1,00	0,993502495
FALSO	Medium	Master Protein	Q13835	Plakophilin-1 OS=Homo sapiens (Human) OX=9606 GN=PKP1 PE=1 SV=2	PKP1	82,8	1,00	#¡DIV/0!
FALSO	High	Master Protein	P26641	Elongation factor 1-gamma OS=Homo sapiens (Human) OX=9606 GN=EEF1G PE=1 SV=3	EEF1G	50,1	1,00	0,998150223
FALSO	High	Master Protein	Q6EMK4	Vasorin OS=Homo sapiens (Human) OX=9606 GN=VASN PE=1 SV=1	VASN	71,7	1,00	0,996803718
FALSO	High	Master Protein	Q04760	Lactoylglutathione lyase OS=Homo sapiens (Human) OX=9606 GN=GLO1 PE=1 SV=4	GLO1	20,8	1,00	0,990826792
FALSO	High	Master Protein	O95498	Vascular non-inflammatory molecule 2 OS=Homo sapiens (Human) OX=9606 GN=VNN2 PE=1 SV=3	VNN2	58,5	1,00	0,992336182
FALSO	High	Master Protein	P08311	Cathepsin G OS=Homo sapiens (Human) OX=9606 GN=CTSG PE=1 SV=2	CTSG	28,8	1,00	0,989705286
FALSO	High	Master Protein	P10909	Clusterin OS=Homo sapiens (Human) OX=9606 GN=CLU PE=1 SV=1	CLU	52,5	1,00	0,993146546
FALSO	Medium	Master Protein	Q15750	TGF-beta-activated kinase 1 and MAP3K7-binding protein 1 OS=Homo sapiens (Human) OX=9606 GN=TAB1 P	TAB1	54,6	1,00	0,991616872
FALSO	High	None	A6NIZ1	Ras-related protein Rap-1b-like protein OS=Homo sapiens (Human) OX=9606 GN=- PE=2 SV=1	RAP1BL	20,9	1,00	0,990161635
FALSO	High	Master Protein	Q96G03	Phosphoglucomutase-2 OS=Homo sapiens (Human) OX=9606 GN=PGM2 PE=1 SV=4	PGM2	68,2	1,00	0,993970617
FALSO	High	Master Protein	P59998	Actin-related protein 2/3 complex subunit 4 OS=Homo sapiens (Human) OX=9606 GN=ARPC4 PE=1 SV=3	ARPC4	19,7	1,00	0,986289189
FALSO	High	None	P14649	Myosin light chain 6B OS=Homo sapiens (Human) OX=9606 GN=MYL6B PE=1 SV=1	MYL6B	22,8	1,00	0,989120216
FALSO	Medium	Master Protein	P62304	Small nuclear ribonucleoprotein E OS=Homo sapiens (Human) OX=9606 GN=SNRPE PE=1 SV=1	SNRPE	10,8	1,00	0,982123335
FALSO	High	Master Protein	P0DOY3	Immunoglobulin lambda constant 3 OS=Homo sapiens (Human) OX=9606 GN=IGLC3 PE=1 SV=1	IGLC3	11,3	1,00	0,981332193
FALSO	High	Master Protein	P14174	Macrophage migration inhibitory factor OS=Homo sapiens (Human) OX=9606 GN=MIF PE=1 SV=4	MIF	12,5	0,99	0,972134556
FALSO	High	Master Protein	Q15084	Protein disulfide-isomerase A6 OS=Homo sapiens (Human) OX=9606 GN=PDIA6 PE=1 SV=1	PDIA6	48,1	0,99	0,971557897
FALSO	High	Master Protein	P09525	Annexin A4 OS=Homo sapiens (Human) OX=9606 GN=ANXA4 PE=1 SV=4	ANXA4	35,9	0,99	0,979182088
FALSO	High	Master Protein	P23381	Tryptophan--tRNA ligase, cytoplasmic OS=Homo sapiens (Human) OX=9606 GN=WARS1 PE=1 SV=2	WARS	53,1	0,99	0,96119119
FALSO	High	Master Protein	P10643	Complement component C7 OS=Homo sapiens (Human) OX=9606 GN=C7 PE=1 SV=2	C7	93,5	0,99	0,960121322
FALSO	High	Master Protein	A0A0C4DH34	Immunoglobulin heavy variable 4-28 OS=Homo sapiens (Human) OX=9606 GN=IGHV4-28 PE=3 SV=1	IGHV4-28	13,1	0,99	0,963508913
FALSO	High	Master Protein	P28838	Cytosol aminopeptidase OS=Homo sapiens (Human) OX=9606 GN=LAP3 PE=1 SV=3	LAP3	56,1	0,99	0,951084923
FALSO	High	Master Protein	P14151	L-selectin OS=Homo sapiens (Human) OX=9606 GN=SELL PE=1 SV=2	SELL	42,2	0,99	0,907619982
FALSO	High	Master Protein	P04264	Keratin, type II cytoskeletal 1 OS=Homo sapiens (Human) OX=9606 GN=KRT1 PE=1 SV=6	KRT1	66	0,99	0,971196047
FALSO	High	Master Protein	P06748	Nucleophosmin OS=Homo sapiens (Human) OX=9606 GN=NPM1 PE=1 SV=2	NPM1	32,6	0,99	0,934498967
FALSO	High	Master Protein	P01857	Immunoglobulin heavy constant gamma 1 OS=Homo sapiens (Human) OX=9606 GN=IGHG1 PE=1 SV=1	IGHG1	36,1	0,99	0,883610871
FALSO	High	None	P62834	Ras-related protein Rap-1A OS=Homo sapiens (Human) OX=9606 GN=RAP1A PE=1 SV=1	RAP1A	21	0,98	0,949059648
FALSO	High	Master Protein	P00751	Complement factor B OS=Homo sapiens (Human) OX=9606 GN=CFB PE=1 SV=2	CFB	85,5	0,98	0,911444994
FALSO	High	Master Protein	Q15080	Neutrophil cytosol factor 4 OS=Homo sapiens (Human) OX=9606 GN=NCF4 PE=1 SV=2	NCF4	39	0,98	0,9157874
FALSO	High	Master Protein	P52790	Hexokinase-3 OS=Homo sapiens (Human) OX=9606 GN=HK3 PE=1 SV=2	HK3	99	0,98	0,933476334
FALSO	High	Master Protein Candidat	Q8NHW5	60S acidic ribosomal protein P0-like OS=Homo sapiens (Human) OX=9606 GN=RPLP0P6 PE=5 SV=1	RPLP0P6	34,3	0,98	0,949911365
FALSO	High	Master Protein	P05388	60S acidic ribosomal protein P0 OS=Homo sapiens (Human) OX=9606 GN=RPLP0 PE=1 SV=1	RPLP0	34,3	0,98	0,949911365

FALSO	High	Master Protein Candidat	P55854	Small ubiquitin-related modifier 3 OS=Homo sapiens (Human) OX=9606 GN=SUMO3 PE=1 SV=2	SUMO3	11,6		0,98	0,942143812
FALSO	High	Master Protein Candidat	P61956	Small ubiquitin-related modifier 2 OS=Homo sapiens (Human) OX=9606 GN=SUMO2 PE=1 SV=3	SUMO2	10,9		0,98	0,942143812
FALSO	High	Master Protein	Q6EEV6	Small ubiquitin-related modifier 4 OS=Homo sapiens (Human) OX=9606 GN=SUMO4 PE=1 SV=2	SUMO4	10,7		0,98	0,942143812
FALSO	High	Master Protein	P02675	Fibrinogen beta chain OS=Homo sapiens (Human) OX=9606 GN=FGB PE=1 SV=2	FGB	55,9		0,98	0,933050025
FALSO	High	Master Protein	O60235	Transmembrane protease serine 11D OS=Homo sapiens (Human) OX=9606 GN=TMPRSS11D PE=1 SV=1	TMPRSS11D	46,2		0,98	0,951106513
FALSO	High	Master Protein	P42768	Wiskott-Aldrich syndrome protein OS=Homo sapiens (Human) OX=9606 GN=WAS PE=1 SV=4	WAS	52,9		0,98	0,939025697
FALSO	High	None	P11233	Ras-related protein Ral-A OS=Homo sapiens (Human) OX=9606 GN=RALA PE=1 SV=1	RALA	23,6		0,98	0,92503345
FALSO	High	Master Protein	P31146	Coronin-1A OS=Homo sapiens (Human) OX=9606 GN=CORO1A PE=1 SV=4	CORO1A	51		0,98	0,911768378
FALSO	High	Master Protein	P04350	Tubulin beta-4A chain OS=Homo sapiens (Human) OX=9606 GN=TUBB4A PE=1 SV=2	TUBB4A	49,6		0,98	0,958734316
FALSO	Medium	Master Protein	P47895	Aldehyde dehydrogenase family 1 member A3 OS=Homo sapiens (Human) OX=9606 GN=ALDH1A3 PE=1 SV=2	ALDH1A3	56,1		0,98	0,959688466
FALSO	High	Master Protein	P28070	Proteasome subunit beta type-4 OS=Homo sapiens (Human) OX=9606 GN=PSMB4 PE=1 SV=4	PSMB4	29,2		0,97	0,910552216
FALSO	High	Master Protein	Q96DA0	Zymogen granule protein 16 homolog B OS=Homo sapiens (Human) OX=9606 GN=ZG16B PE=1 SV=3	ZG16B	22,7		0,97	0,949424701
FALSO	High	Master Protein	O75340	Programmed cell death protein 6 OS=Homo sapiens (Human) OX=9606 GN=PDCD6 PE=1 SV=1	PDCD6	21,9		0,97	0,919901989
FALSO	High	Master Protein	Q9Y490	Talin-1 OS=Homo sapiens (Human) OX=9606 GN=TLN1 PE=1 SV=3	TLN1	270		0,97	0,861791629
FALSO	High	Master Protein	P68032	Actin, alpha cardiac muscle 1 OS=Homo sapiens (Human) OX=9606 GN=ACTC1 PE=1 SV=1	ACTC1	42		0,97	0,937255403
FALSO	High	Master Protein	P36543	V-type proton ATPase subunit E 1 OS=Homo sapiens (Human) OX=9606 GN=ATP6V1E1 PE=1 SV=1	ATP6V1E1	26,1		0,97	0,933514409
FALSO	Medium	Master Protein	Q8IV08	5'-3' exonuclease PLD3 OS=Homo sapiens (Human) OX=9606 GN=PLD3 PE=1 SV=1	PLD3	54,7		0,97	0,935484074
FALSO	High	Master Protein	P48637	Glutathione synthetase OS=Homo sapiens (Human) OX=9606 GN=GSS PE=1 SV=1	GSS	52,4		0,97	0,855958662
FALSO	Medium	Master Protein	P49748	Very long-chain specific acyl-CoA dehydrogenase, mitochondrial OS=Homo sapiens (Human) OX=9606 GN=A(	ACADVL	70,3		0,97	0,917619373
FALSO	High	Master Protein	P26022	Pentraxin-related protein PTX3 OS=Homo sapiens (Human) OX=9606 GN=PTX3 PE=1 SV=3	PTX3	41,9		0,97	0,888448303
FALSO	High	Master Protein	P11234	Ras-related protein Ral-B OS=Homo sapiens (Human) OX=9606 GN=RALB PE=1 SV=1	RALB	23,4		0,97	0,884729042
FALSO	High	Master Protein	P21333	Filamin-A OS=Homo sapiens (Human) OX=9606 GN=FLNA PE=1 SV=4	FLNA	281		0,96	0,873384873
FALSO	High	Master Protein	P52565	Rho GDP-dissociation inhibitor 1 OS=Homo sapiens (Human) OX=9606 GN=ARHGDIA PE=1 SV=3	ARHGDIA	23,2		0,96	0,830512103
FALSO	High	Master Protein	P01042	Kininogen-1 OS=Homo sapiens (Human) OX=9606 GN=KNG1 PE=1 SV=2	KNG1	71,9		0,96	0,665348594
FALSO	High	Master Protein	P0DP02	Immunoglobulin heavy variable 3-30-3 OS=Homo sapiens (Human) OX=9606 GN=IGHV3-30-3 PE=3 SV=1	IGHV3-30; IGHV3-	13		0,96	0,888451142
FALSO	Medium	Master Protein	Q9Y2J8	Protein-arginine deiminase type-2 OS=Homo sapiens (Human) OX=9606 GN=PADI2 PE=1 SV=2	PADI2	75,5		0,96	0,881506682
FALSO	High	Master Protein	P61923	Coatomer subunit zeta-1 OS=Homo sapiens (Human) OX=9606 GN=COPZ1 PE=1 SV=1	COPZ1	20,2		0,96	0,915131606
FALSO	High	Master Protein	O00233	26S proteasome non-ATPase regulatory subunit 9 OS=Homo sapiens (Human) OX=9606 GN=PSMD9 PE=1 SV	PSMD9	24,7		0,96	0,897018511
FALSO	High	Master Protein	P12955	Xaa-Pro dipeptidase OS=Homo sapiens (Human) OX=9606 GN=PEPD PE=1 SV=3	PEPD	54,5		0,96	0,83955141
FALSO	High	Master Protein	Q9NTK5	Obg-like ATPase 1 OS=Homo sapiens (Human) OX=9606 GN=OLA1 PE=1 SV=2	OLA1	44,7		0,96	0,816746246
FALSO	Medium	Master Protein	Q99490	Arf-GAP with GTPase, ANK repeat and PH domain-containing protein 2 OS=Homo sapiens (Human) OX=9606	AGAP2	125		0,96	0,864490783
FALSO	High	Master Protein	P14780	Matrix metalloproteinase-9 OS=Homo sapiens (Human) OX=9606 GN=MMP9 PE=1 SV=3	MMP9	78,4		0,96	0,844706026
FALSO	High	None	P07205	Phosphoglycerate kinase 2 OS=Homo sapiens (Human) OX=9606 GN=PGK2 PE=1 SV=3	PGK2	44,8		0,96	0,80781701

FALSO	High	Master Protein	P22314	Ubiquitin-like modifier-activating enzyme 1 OS=Homo sapiens (Human) OX=9606 GN=UBA1 PE=1 SV=3	UBA1	118	0,96	0,747313763
FALSO	High	Master Protein	Q14974	Importin subunit beta-1 OS=Homo sapiens (Human) OX=9606 GN=KPNB1 PE=1 SV=2	KPNB1	97,1	0,95	0,856690956
FALSO	Medium	Master Protein	O14802	DNA-directed RNA polymerase III subunit RPC1 OS=Homo sapiens (Human) OX=9606 GN=POLR3A PE=1 SV=2	POLR3A	156	0,95	0,891057809
FALSO	High	Master Protein	Q10567	AP-1 complex subunit beta-1 OS=Homo sapiens (Human) OX=9606 GN=AP1B1 PE=1 SV=2	AP1B1	105	0,95	0,811607891
FALSO	High	None	P11055	Myosin-3 OS=Homo sapiens (Human) OX=9606 GN=MYH3 PE=1 SV=3	MYH3	224	0,95	#¡DIV/0!
FALSO	High	None	P68133	Actin, alpha skeletal muscle OS=Homo sapiens (Human) OX=9606 GN=ACTA1 PE=1 SV=1	ACTA1	42	0,95	0,884059014
FALSO	Medium	Master Protein	A0A075B6K0	Immunoglobulin lambda variable 3-16 OS=Homo sapiens (Human) OX=9606 GN=IGLV3-16 PE=3 SV=2	IGLV3-16	12,5	0,95	0,889806832
FALSO	High	Master Protein	O95445	Apolipoprotein M OS=Homo sapiens (Human) OX=9606 GN=APOM PE=1 SV=2	APOM	21,2	0,95	0,795755479
FALSO	High	Master Protein	P61224	Ras-related protein Rap-1b OS=Homo sapiens (Human) OX=9606 GN=RAP1B PE=1 SV=1	RAP1B	20,8	0,95	0,810701269
FALSO	High	Master Protein	Q14203	Dynactin subunit 1 OS=Homo sapiens (Human) OX=9606 GN=DCTN1 PE=1 SV=3	DCTN1	142	0,95	0,776635371
FALSO	High	Master Protein	P09668	Pro-cathepsin H OS=Homo sapiens (Human) OX=9606 GN=CTSH PE=1 SV=4	CTSH	37,4	0,95	0,814457467
FALSO	Medium	Master Protein	P25098	Beta-adrenergic receptor kinase 1 OS=Homo sapiens (Human) OX=9606 GN=GRK2 PE=1 SV=2	GRK2	79,5	0,95	0,79992953
FALSO	Medium	Master Protein	Q8TAX7	Mucin-7 OS=Homo sapiens (Human) OX=9606 GN=MUC7 PE=1 SV=2	MUC7	39,1	0,94	0,90458207
FALSO	High	Master Protein	P49913	Cathelicidin antimicrobial peptide OS=Homo sapiens (Human) OX=9606 GN=CAMP PE=1 SV=1	CAMP	19,3	0,94	0,842498341
FALSO	High	None	P49590	Histidine--tRNA ligase, mitochondrial OS=Homo sapiens (Human) OX=9606 GN=HARS2 PE=1 SV=1	HARS2	56,9	0,94	0,82131856
FALSO	High	None	P60763	Ras-related C3 botulinum toxin substrate 3 OS=Homo sapiens (Human) OX=9606 GN=RAC3 PE=1 SV=1	RAC3	21,4	0,94	0,784183493
FALSO	High	None	P08134	Rho-related GTP-binding protein RhoC OS=Homo sapiens (Human) OX=9606 GN=RHOC PE=1 SV=1	RHOC	22	0,94	0,832007651
FALSO	High	Master Protein	Q15166	Serum paraoxonase/lactonase 3 OS=Homo sapiens (Human) OX=9606 GN=PON3 PE=1 SV=3	PON3	39,6	0,94	0,910154688
FALSO	High	Master Protein	P04004	Vitronectin OS=Homo sapiens (Human) OX=9606 GN=VTN PE=1 SV=1	VTN	54,3	0,94	0,662031053
FALSO	High	Master Protein	P80108	Phosphatidylinositol-glycan-specific phospholipase D OS=Homo sapiens (Human) OX=9606 GN=GPLD1 PE=1	GPLD1	92,3	0,94	0,784202924
FALSO	High	Master Protein	P48444	Coatomer subunit delta OS=Homo sapiens (Human) OX=9606 GN=ARCN1 PE=1 SV=1	ARCN1	57,2	0,94	0,715764337
FALSO	High	Master Protein	P00738	Haptoglobin OS=Homo sapiens (Human) OX=9606 GN=HP PE=1 SV=1	HP	45,2	0,94	0,712754427
FALSO	High	Master Protein	P78417	Glutathione S-transferase omega-1 OS=Homo sapiens (Human) OX=9606 GN=GSTO1 PE=1 SV=2	GSTO1	27,5	0,94	0,750884016
FALSO	High	Master Protein	P78380	Oxidized low-density lipoprotein receptor 1 OS=Homo sapiens (Human) OX=9606 GN=OLR1 PE=1 SV=1	OLR1	30,9	0,94	0,806061887
FALSO	High	Master Protein	P18206	Vinculin OS=Homo sapiens (Human) OX=9606 GN=VCL PE=1 SV=4	VCL	124	0,94	0,759338125
FALSO	High	Master Protein	P02790	Hemopexin OS=Homo sapiens (Human) OX=9606 GN=HPX PE=1 SV=2	HPX	51,6	0,94	0,513075697
FALSO	High	Master Protein	P06310	Immunoglobulin kappa variable 2-30 OS=Homo sapiens (Human) OX=9606 GN=IGKV2-30 PE=3 SV=2	IGKV2-30	13,2	0,94	0,64129946
FALSO	High	Master Protein	P23083	Immunoglobulin heavy variable 1-2 OS=Homo sapiens (Human) OX=9606 GN=IGHV1-2 PE=1 SV=2	IGHV1OR15-1	13,1	0,94	0,737812283
FALSO	High	Master Protein	P06733	Alpha-enolase OS=Homo sapiens (Human) OX=9606 GN=ENO1 PE=1 SV=2	ENO1	47,1	0,93	0,61122582
FALSO	High	Master Protein	P33241	Lymphocyte-specific protein 1 OS=Homo sapiens (Human) OX=9606 GN=LSP1 PE=1 SV=1	LSP1	37,2	0,93	0,802788234
FALSO	High	Master Protein	Q06033	Inter-alpha-trypsin inhibitor heavy chain H3 OS=Homo sapiens (Human) OX=9606 GN=ITIH3 PE=1 SV=2	ITIH3	99,8	0,93	0,729248484
FALSO	High	Master Protein	Q15369	Elongin-C OS=Homo sapiens (Human) OX=9606 GN=ELOC PE=1 SV=1	TCEB1; ELOC	12,5	0,93	0,801295197
FALSO	High	Master Protein	P58546	Myotrophin OS=Homo sapiens (Human) OX=9606 GN=MTPN PE=1 SV=2	MTPN	12,9	0,93	0,756209712

FALSO	High	Master Protein	Q99497	Protein/nucleic acid deglycase DJ-1 OS=Homo sapiens (Human) OX=9606 GN=PARK7 PE=1 SV=2	PARK7	19,9	0,93	0,625298416
FALSO	High	Master Protein	A0A0C4DH69	Immunoglobulin kappa variable 1-9 OS=Homo sapiens (Human) OX=9606 GN=IGKV1-9 PE=3 SV=1	IGKV1-9	12,7	0,93	0,764376566
FALSO	High	Master Protein Candidat	A0A0C4DH67	Immunoglobulin kappa variable 1-8 OS=Homo sapiens (Human) OX=9606 GN=IGKV1-8 PE=3 SV=1	IGKV1-8	12,5	0,93	0,764376566
FALSO	Medium	Master Protein	P10619	Lysosomal protective protein OS=Homo sapiens (Human) OX=9606 GN=CTSA PE=1 SV=2	CTSA	54,4	0,93	0,792617497
FALSO	High	Master Protein	P14550	Aldo-keto reductase family 1 member A1 OS=Homo sapiens (Human) OX=9606 GN=AKR1A1 PE=1 SV=3	AKR1A1	36,6	0,93	0,742387149
FALSO	High	Master Protein	P15144	Aminopeptidase N OS=Homo sapiens (Human) OX=9606 GN=ANPEP PE=1 SV=4	ANPEP	110	0,93	0,770038757
FALSO	High	None	Q16695	Histone H3.1t OS=Homo sapiens (Human) OX=9606 GN=HIST3H3 PE=1 SV=3	HIST3H3	15,5	0,93	0,79416014
FALSO	High	Master Protein	P42679	Megakaryocyte-associated tyrosine-protein kinase OS=Homo sapiens (Human) OX=9606 GN=MATK PE=1 SV=:	MATK	56,4	0,93	0,714152666
FALSO	High	Master Protein Candidat	P41240	Tyrosine-protein kinase CSK OS=Homo sapiens (Human) OX=9606 GN=CSK PE=1 SV=1	CSK	50,7	0,93	0,714152666
FALSO	High	Master Protein	Q99829	Copine-1 OS=Homo sapiens (Human) OX=9606 GN=CPNE1 PE=1 SV=1	CPNE1	59	0,93	0,830553962
FALSO	High	Master Protein	P63173	60S ribosomal protein L38 OS=Homo sapiens (Human) OX=9606 GN=RPL38 PE=1 SV=2	RPL38	8,2	0,93	#¡DIV/0!
FALSO	High	Master Protein	P32942	Intercellular adhesion molecule 3 OS=Homo sapiens (Human) OX=9606 GN=ICAM3 PE=1 SV=2	ICAM3	59,5	0,93	0,695821682
FALSO	High	Master Protein	O00299	Chloride intracellular channel protein 1 OS=Homo sapiens (Human) OX=9606 GN=CLIC1 PE=1 SV=4	CLIC1	26,9	0,93	0,663430408
FALSO	High	None	A0A075B6S5	Immunoglobulin kappa variable 1-27 OS=Homo sapiens (Human) OX=9606 GN=IGKV1-27 PE=3 SV=1	IGKV1-27	12,7	0,93	0,764299525
FALSO	High	None	Q92928	Putative Ras-related protein Rab-1C OS=Homo sapiens (Human) OX=9606 GN=RAB1C PE=5 SV=2	RAB1C	22	0,93	0,776187582
FALSO	High	None	Q9H0U4	Ras-related protein Rab-1B OS=Homo sapiens (Human) OX=9606 GN=RAB1B PE=1 SV=1	RAB1B	22,2	0,93	0,776187582
FALSO	High	None	P84243	Histone H3.3 OS=Homo sapiens (Human) OX=9606 GN=H3-3B PE=1 SV=2	H3F3A	15,3	0,93	0,786743111
FALSO	High	Master Protein	P68431	Histone H3.1 OS=Homo sapiens (Human) OX=9606 GN=H3C2 PE=1 SV=2	HIST1H3F	15,4	0,93	0,78651696
FALSO	High	Master Protein Candidat	Q71DI3	Histone H3.2 OS=Homo sapiens (Human) OX=9606 GN=HIST2H3C PE=1 SV=3	HIST2H3A	15,4	0,93	0,78651696
FALSO	High	Master Protein	Q13126	S-methyl-5'-thioadenosine phosphorylase OS=Homo sapiens (Human) OX=9606 GN=MTAP PE=1 SV=2	MTAP	31,2	0,93	0,654002218
FALSO	High	Master Protein	P28065	Proteasome subunit beta type-9 OS=Homo sapiens (Human) OX=9606 GN=PSMB9 PE=1 SV=2	PSMB9	23,3	0,93	0,752891569
FALSO	High	None	Q9Y4G6	Talin-2 OS=Homo sapiens (Human) OX=9606 GN=TLN2 PE=1 SV=4	TLN2	271	0,93	0,659465424
FALSO	High	Master Protein	Q70J99	Protein unc-13 homolog D OS=Homo sapiens (Human) OX=9606 GN=UNC13D PE=1 SV=1	UNC13D	123	0,93	0,786825448
FALSO	High	Master Protein	P14625	Endoplasmin OS=Homo sapiens (Human) OX=9606 GN=HSP90B1 PE=1 SV=1	HSP90B1	92,4	0,93	0,741357838
FALSO	High	Master Protein	P26038	Moesin OS=Homo sapiens (Human) OX=9606 GN=MSN PE=1 SV=3	MSN	67,8	0,93	0,622825591
FALSO	High	Master Protein	P15153	Ras-related C3 botulinum toxin substrate 2 OS=Homo sapiens (Human) OX=9606 GN=RAC2 PE=1 SV=1	RAC2	21,4	0,92	0,71569292
FALSO	High	Master Protein	Q16769	Glutaminyl-peptide cyclotransferase OS=Homo sapiens (Human) OX=9606 GN=QPCT PE=1 SV=1	QPCT	40,9	0,92	0,783102696
FALSO	High	Master Protein	P30101	Protein disulfide-isomerase A3 OS=Homo sapiens (Human) OX=9606 GN=PDIA3 PE=1 SV=4	PDIA3	56,7	0,92	0,616447201
FALSO	Medium	Master Protein	O60888	Protein CutA OS=Homo sapiens (Human) OX=9606 GN=CUTA PE=1 SV=2	CUTA	19,1	0,92	0,788198401
FALSO	High	Master Protein	P40925	Malate dehydrogenase, cytoplasmic OS=Homo sapiens (Human) OX=9606 GN=MDH1 PE=1 SV=4	MDH1	36,4	0,92	0,672829007
FALSO	High	Master Protein	O75636	Ficolin-3 OS=Homo sapiens (Human) OX=9606 GN=FCN3 PE=1 SV=2	FCN3	32,9	0,92	0,624829988
FALSO	High	Master Protein	O43399	Tumor protein D54 OS=Homo sapiens (Human) OX=9606 GN=TPD52L2 PE=1 SV=2	TPD52L2	22,2	0,92	0,682256325
FALSO	High	Master Protein	P01593	Immunoglobulin kappa variable 1D-33 OS=Homo sapiens (Human) OX=9606 GN=IGKV1D-33 PE=1 SV=2	IGKV1D-33	12,8	0,92	0,608422657

FALSO	High	Master Protein	P17213	Bactericidal permeability-increasing protein OS=Homo sapiens (Human) OX=9606 GN=BPI PE=1 SV=4	BPI	53,9	0,92	0,78518493
FALSO	High	Master Protein	O75937	DnaJ homolog subfamily C member 8 OS=Homo sapiens (Human) OX=9606 GN=DNAJC8 PE=1 SV=2	DNAJC8	29,8	0,92	0,715265671
FALSO	High	Master Protein	P61019	Ras-related protein Rab-2A OS=Homo sapiens (Human) OX=9606 GN=RAB2A PE=1 SV=1	RAB2A	23,5	0,92	0,72880464
FALSO	High	Master Protein	Q71UI9	Histone H2A.V OS=Homo sapiens (Human) OX=9606 GN=H2AFV PE=1 SV=3	H2AFV	13,5	0,92	0,790013887
FALSO	High	Master Protein Candidat	P0C0S5	Histone H2A.Z OS=Homo sapiens (Human) OX=9606 GN=H2AZ1 PE=1 SV=2	H2AFZ	13,5	0,92	0,790013887
FALSO	High	Master Protein	O75531	Barrier-to-autointegration factor OS=Homo sapiens (Human) OX=9606 GN=BANF1 PE=1 SV=1	BANF1	10,1	0,92	0,650124923
FALSO	Medium	Master Protein	A0A0C4DH43	Immunoglobulin heavy variable 2-70D OS=Homo sapiens (Human) OX=9606 GN=IGHV2-70D PE=3 SV=1	IGHV2-70D	13,3	0,92	0,78360944
FALSO	High	Master Protein	P84090	Enhancer of rudimentary homolog OS=Homo sapiens (Human) OX=9606 GN=ERH PE=1 SV=1	ERH	12,3	0,92	0,683857975
FALSO	High	Master Protein	A0M8Q6	Immunoglobulin lambda constant 7 OS=Homo sapiens (Human) OX=9606 GN=IGLC7 PE=1 SV=3	IGLC7	11,2	0,91	0,856922726
FALSO	High	Master Protein	P25774	Cathepsin S OS=Homo sapiens (Human) OX=9606 GN=CTSS PE=1 SV=3	CTSS	37,5	0,91	0,70532567
FALSO	High	None	Q9BXR6	Complement factor H-related protein 5 OS=Homo sapiens (Human) OX=9606 GN=CFHR5 PE=1 SV=1	CFHR5	64,4	0,91	0,699540356
FALSO	High	Master Protein	P06132	Uroporphyrinogen decarboxylase OS=Homo sapiens (Human) OX=9606 GN=UROD PE=1 SV=2	UROD	40,8	0,91	0,775499785
FALSO	High	Master Protein	Q01105	Protein SET OS=Homo sapiens (Human) OX=9606 GN=SET PE=1 SV=3	SET	33,5	0,91	0,613173576
FALSO	High	None	Q9Y536	Peptidyl-prolyl cis-trans isomerase A-like 4A OS=Homo sapiens (Human) OX=9606 GN=PPIAL4A PE=2 SV=1	PPIAL4A	18,2	0,91	0,526296607
FALSO	High	Master Protein	Q9NPY3	Complement component C1q receptor OS=Homo sapiens (Human) OX=9606 GN=CD93 PE=1 SV=3	CD93	68,5	0,91	0,650712127
FALSO	High	None	P0DME0	Protein SETSIP OS=Homo sapiens (Human) OX=9606 GN=SETSIP PE=1 SV=1	SETSIP	34,9	0,91	0,606050176
FALSO	High	Master Protein	P13010	X-ray repair cross-complementing protein 5 OS=Homo sapiens (Human) OX=9606 GN=XRCC5 PE=1 SV=3	XRCC5	82,7	0,91	0,655541655
FALSO	High	Master Protein	P61026	Ras-related protein Rab-10 OS=Homo sapiens (Human) OX=9606 GN=RAB10 PE=1 SV=1	RAB10	22,5	0,91	0,466331702
FALSO	High	Master Protein	P01619	Immunoglobulin kappa variable 3-20 OS=Homo sapiens (Human) OX=9606 GN=IGKV3-20 PE=1 SV=2	IGKV3-20	12,5	0,91	0,347848062
FALSO	High	Master Protein	P23142	Fibulin-1 OS=Homo sapiens (Human) OX=9606 GN=FBLN1 PE=1 SV=4	FBLN1	77,2	0,91	0,544487916
FALSO	High	Master Protein	P07814	Bifunctional glutamate/proline--tRNA ligase OS=Homo sapiens (Human) OX=9606 GN=EPRS1 PE=1 SV=5	EPRS	171	0,91	0,76414514
FALSO	High	Master Protein	P02765	Alpha-2-HS-glycoprotein OS=Homo sapiens (Human) OX=9606 GN=AHSG PE=1 SV=2	AHSG	39,3	0,91	0,501306531
FALSO	High	Master Protein	P14923	Junction plakoglobin OS=Homo sapiens (Human) OX=9606 GN=JUP PE=1 SV=3	JUP	81,7	0,91	0,848993979
FALSO	High	Master Protein	P23528	Cofilin-1 OS=Homo sapiens (Human) OX=9606 GN=CFL1 PE=1 SV=3	CFL1	18,5	0,91	0,468236594
FALSO	High	Master Protein	P13473	Lysosome-associated membrane glycoprotein 2 OS=Homo sapiens (Human) OX=9606 GN=LAMP2 PE=1 SV=2	LAMP2	44,9	0,91	0,706948658
FALSO	High	Master Protein	P61916	NPC intracellular cholesterol transporter 2 OS=Homo sapiens (Human) OX=9606 GN=NPC2 PE=1 SV=1	NPC2	16,6	0,91	0,569755486
FALSO	High	Master Protein	P50990	T-complex protein 1 subunit theta OS=Homo sapiens (Human) OX=9606 GN=CCT8 PE=1 SV=4	CCT8	59,6	0,90	0,589780787
FALSO	High	Master Protein	P52209	6-phosphogluconate dehydrogenase, decarboxylating OS=Homo sapiens (Human) OX=9606 GN=PGD PE=1 S	PGD	53,1	0,90	0,67184774
FALSO	High	Master Protein	O75874	Isocitrate dehydrogenase [NADP] cytoplasmic OS=Homo sapiens (Human) OX=9606 GN=IDH1 PE=1 SV=2	IDH1	46,6	0,90	0,527706658
FALSO	High	Master Protein	P62258	14-3-3 protein epsilon OS=Homo sapiens (Human) OX=9606 GN=YWHAE PE=1 SV=1	YWHAE	29,2	0,90	0,545941149
FALSO	High	Master Protein	P99999	Cytochrome c OS=Homo sapiens (Human) OX=9606 GN=CYCS PE=1 SV=2	CYCS	11,7	0,90	0,773973784
FALSO	High	Master Protein	P08603	Complement factor H OS=Homo sapiens (Human) OX=9606 GN=CFH PE=1 SV=4	CFH	139	0,90	0,294487617
FALSO	High	None	A0A2R8Y4L2	Heterogeneous nuclear ribonucleoprotein A1 pseudogene 48 OS=Homo sapiens (Human) OX=9606 GN=HNR	HNRNPA1P48	29,2	0,90	0,600987969

FALSO	High	Master Protein	P09972	Fructose-bisphosphate aldolase C OS=Homo sapiens (Human) OX=9606 GN=ALDOC PE=1 SV=2	ALDOC	39,4	0,90	0,674036509
FALSO	High	Master Protein	P25788	Proteasome subunit alpha type-3 OS=Homo sapiens (Human) OX=9606 GN=PSMA3 PE=1 SV=2	PSMA3	28,4	0,90	0,557951845
FALSO	Medium	None	A0A2R8Y7G9	Histone domain-containing protein OS=Homo sapiens (Human) OX=9606 GN=H3.Y PE=3 SV=1	H3.Y	15,4	0,90	0,787238761
FALSO	Medium	None	P0DPK5	Putative histone H3.X OS=Homo sapiens (Human) OX=9606 GN=- PE=5 SV=1	H3.X	16,5	0,90	0,787238761
FALSO	High	Master Protein	P01861	Immunoglobulin heavy constant gamma 4 OS=Homo sapiens (Human) OX=9606 GN=IGHG4 PE=1 SV=1	IGHG4	35,9	0,90	0,733080012
FALSO	High	Master Protein	Q9UJU6	Drebrin-like protein OS=Homo sapiens (Human) OX=9606 GN=DBNL PE=1 SV=1	DBNL	48,2	0,90	0,532834093
FALSO	Medium	Master Protein	P17858	ATP-dependent 6-phosphofructokinase, liver type OS=Homo sapiens (Human) OX=9606 GN=PFKL PE=1 SV=6	PFKL	85	0,90	0,786611525
FALSO	High	Master Protein	Q9UMS4	Pre-mRNA-processing factor 19 OS=Homo sapiens (Human) OX=9606 GN=PRPF19 PE=1 SV=1	PRPF19	55,1	0,90	0,719623023
FALSO	High	Master Protein	P61586	Transforming protein RhoA OS=Homo sapiens (Human) OX=9606 GN=RHOA PE=1 SV=1	RHOA	21,8	0,90	0,708728903
FALSO	High	Master Protein	O95989	Diphosphoinositol polyphosphate phosphohydrolase 1 OS=Homo sapiens (Human) OX=9606 GN=NUDT3 P	NUDT3	19,5	0,89	#¡DIV/0!
FALSO	High	Master Protein	Q8WUM4	Programmed cell death 6-interacting protein OS=Homo sapiens (Human) OX=9606 GN=PDCD6IP PE=1 SV=1	PDCD6IP	96	0,89	0,680183461
FALSO	High	Master Protein	P00338	L-lactate dehydrogenase A chain OS=Homo sapiens (Human) OX=9606 GN=LDHA PE=1 SV=2	LDHA	36,7	0,89	0,586083649
FALSO	High	Master Protein	P43652	Afamin OS=Homo sapiens (Human) OX=9606 GN=AFM PE=1 SV=1	AFM	69	0,89	0,320478314
FALSO	High	Master Protein	P00390	Glutathione reductase, mitochondrial OS=Homo sapiens (Human) OX=9606 GN=GSR PE=1 SV=2	GSR	56,2	0,89	0,463943897
FALSO	Medium	Master Protein	Q13740	CD166 antigen OS=Homo sapiens (Human) OX=9606 GN=ALCAM PE=1 SV=2	ALCAM	65,1	0,89	0,761666314
FALSO	High	Master Protein	P62241	40S ribosomal protein S8 OS=Homo sapiens (Human) OX=9606 GN=RPS8 PE=1 SV=2	RPS8	24,2	0,89	0,703022171
FALSO	High	Master Protein	P12429	Annexin A3 OS=Homo sapiens (Human) OX=9606 GN=ANXA3 PE=1 SV=3	ANXA3	36,4	0,89	0,545607933
FALSO	High	Master Protein	P62491	Ras-related protein Rab-11A OS=Homo sapiens (Human) OX=9606 GN=RAB11A PE=1 SV=3	RAB11A	24,4	0,89	0,592985674
FALSO	High	Master Protein Candidat	Q15907	Ras-related protein Rab-11B OS=Homo sapiens (Human) OX=9606 GN=RAB11B PE=1 SV=4	RAB11B	24,5	0,89	0,592985674
FALSO	High	Master Protein	A0A0C4DH36	Probable non-functional immunoglobulin heavy variable 3-38 OS=Homo sapiens (Human) OX=9606 GN=IGH	IGHV3-38	12,8	0,89	0,641268754
FALSO	High	None	A0A075B6P5	Immunoglobulin kappa variable 2-28 OS=Homo sapiens (Human) OX=9606 GN=IGKV2-28 PE=3 SV=1	IGKV2D-28	12,9	0,89	0,489701176
FALSO	High	Master Protein	P60900	Proteasome subunit alpha type-6 OS=Homo sapiens (Human) OX=9606 GN=PSMA6 PE=1 SV=1	PSMA6	27,4	0,89	0,462972858
FALSO	High	Master Protein	P16083	Ribosyldihydronicotinamide dehydrogenase [quinone] OS=Homo sapiens (Human) OX=9606 GN=NQO2 PE=1	NQO2	25,9	0,89	0,706695247
FALSO	High	Master Protein	Q12905	Interleukin enhancer-binding factor 2 OS=Homo sapiens (Human) OX=9606 GN=ILF2 PE=1 SV=2	ILF2	43	0,89	0,659989529
FALSO	High	None	P11217	Glycogen phosphorylase, muscle form OS=Homo sapiens (Human) OX=9606 GN=PYGM PE=1 SV=6	PYGM	97	0,89	0,736559659
FALSO	High	None	P11216	Glycogen phosphorylase, brain form OS=Homo sapiens (Human) OX=9606 GN=PYGB PE=1 SV=5	PYGB	96,6	0,89	0,736559659
FALSO	High	Master Protein	A0A087WSY6	Immunoglobulin kappa variable 3D-15 OS=Homo sapiens (Human) OX=9606 GN=IGKV3D-15 PE=3 SV=6	IGKV3D-15	12,5	0,89	0,424124196
FALSO	High	None	Q96A08	Histone H2B type 1-A OS=Homo sapiens (Human) OX=9606 GN=H2BC1 PE=1 SV=3	HIST1H2BA	14,2	0,89	0,564769082
FALSO	High	Master Protein	P63010	AP-2 complex subunit beta OS=Homo sapiens (Human) OX=9606 GN=AP2B1 PE=1 SV=1	AP2B1	105	0,89	0,596861113
FALSO	Medium	Master Protein	Q9GZR7	ATP-dependent RNA helicase DDX24 OS=Homo sapiens (Human) OX=9606 GN=DDX24 PE=1 SV=1	DDX24	96,3	0,89	0,619102506
FALSO	High	Master Protein Candidat	P01111	GTPase NRas OS=Homo sapiens (Human) OX=9606 GN=NRAS PE=1 SV=1	NRAS	21,2	0,89	0,694580948
FALSO	High	Master Protein Candidat	P01112	GTPase HRas OS=Homo sapiens (Human) OX=9606 GN=HRAS PE=1 SV=1	HRAS	21,3	0,89	0,694580948
FALSO	High	Master Protein	P01116	GTPase KRas OS=Homo sapiens (Human) OX=9606 GN=KRAS PE=1 SV=1	KRAS	21,6	0,89	0,694580948

FALSO	High	None	P01717	Immunoglobulin lambda variable 3-25 OS=Homo sapiens (Human) OX=9606 GN=IGLV3-25 PE=1 SV=2	IGLV3-25	12	0,89	0,512532625
FALSO	High	Master Protein	Q96HE7	ERO1-like protein alpha OS=Homo sapiens (Human) OX=9606 GN=ERO1A PE=1 SV=2	ERO1A	54,4	0,88	0,564639696
FALSO	Medium	Master Protein	Q14376	UDP-glucose 4-epimerase OS=Homo sapiens (Human) OX=9606 GN=GALE PE=1 SV=2	GALE	38,3	0,88	0,582201475
FALSO	High	Master Protein	P02763	Alpha-1-acid glycoprotein 1 OS=Homo sapiens (Human) OX=9606 GN=ORM1 PE=1 SV=1	ORM1	23,5	0,88	0,422746388
FALSO	High	Master Protein	P15586	N-acetylglucosamine-6-sulfatase OS=Homo sapiens (Human) OX=9606 GN=GNS PE=1 SV=3	GNS	62	0,88	0,507863802
FALSO	High	None	P07197	Neurofilament medium polypeptide OS=Homo sapiens (Human) OX=9606 GN=NEFM PE=1 SV=3	NEFM	102	0,88	0,655854545
FALSO	High	None	Q16352	Alpha-internexin OS=Homo sapiens (Human) OX=9606 GN=INA PE=1 SV=2	INA	55,4	0,88	0,655854545
FALSO	High	Master Protein	P07357	Complement component C8 alpha chain OS=Homo sapiens (Human) OX=9606 GN=C8A PE=1 SV=2	C8A	65,1	0,88	0,44812851
FALSO	High	Master Protein	O95777	U6 snRNA-associated Sm-like protein LSm8 OS=Homo sapiens (Human) OX=9606 GN=LSM8 PE=1 SV=3	NAA38; LSM8	10,4	0,88	0,653601416
FALSO	High	Master Protein	P00746	Complement factor D OS=Homo sapiens (Human) OX=9606 GN=CFD PE=1 SV=5	CFD	27	0,88	0,544586656
FALSO	High	Master Protein	A0A087WW87	Immunoglobulin kappa variable 2-40 OS=Homo sapiens (Human) OX=9606 GN=IGKV2-40 PE=3 SV=2	IGKV2-40	13,3	0,88	0,454418929
FALSO	High	Master Protein	Q96AT9	Ribulose-phosphate 3-epimerase OS=Homo sapiens (Human) OX=9606 GN=RPE PE=1 SV=1	RPE	24,9	0,88	0,53001802
FALSO	High	Master Protein	P12814	Alpha-actinin-1 OS=Homo sapiens (Human) OX=9606 GN=ACTN1 PE=1 SV=2	ACTN1	103	0,88	0,578358275
FALSO	High	None	P17661	Desmin OS=Homo sapiens (Human) OX=9606 GN=DES PE=1 SV=3	DES	53,5	0,88	0,635863686
FALSO	High	None	P41219	Peripherin OS=Homo sapiens (Human) OX=9606 GN=PRPH PE=1 SV=2	PRPH	53,6	0,88	0,594018262
FALSO	High	Master Protein	P51148	Ras-related protein Rab-5C OS=Homo sapiens (Human) OX=9606 GN=RAB5C PE=1 SV=2	RAB5C	23,5	0,88	0,602953735
FALSO	High	Master Protein	P10153	Non-secretory ribonuclease OS=Homo sapiens (Human) OX=9606 GN=RNASE2 PE=1 SV=2	RNASE2	18,3	0,88	0,450450478
FALSO	High	Master Protein	Q9H299	SH3 domain-binding glutamic acid-rich-like protein 3 OS=Homo sapiens (Human) OX=9606 GN=SH3BGRL3 P	SH3BGRL3	10,4	0,88	0,423786909
FALSO	High	Master Protein	Q13191	E3 ubiquitin-protein ligase CBL-B OS=Homo sapiens (Human) OX=9606 GN=CBLB PE=1 SV=2	CBLB	109	0,88	0,68856194
FALSO	High	None	P05062	Fructose-bisphosphate aldolase B OS=Homo sapiens (Human) OX=9606 GN=ALDOB PE=1 SV=2	ALDOB	39,4	0,88	0,604733322
FALSO	High	Master Protein	P07225	Vitamin K-dependent protein S OS=Homo sapiens (Human) OX=9606 GN=PROS1 PE=1 SV=1	PROS1	75,1	0,88	0,381940141
FALSO	High	Master Protein	P54819	Adenylate kinase 2, mitochondrial OS=Homo sapiens (Human) OX=9606 GN=AK2 PE=1 SV=2	AK2	26,5	0,88	0,483343428
FALSO	High	Master Protein	Q00610	Clathrin heavy chain 1 OS=Homo sapiens (Human) OX=9606 GN=CLTC PE=1 SV=5	CLTC	192	0,88	0,670443586
FALSO	High	Master Protein	Q9HC35	Echinoderm microtubule-associated protein-like 4 OS=Homo sapiens (Human) OX=9606 GN=EML4 PE=1 SV=3	EML4	109	0,88	0,635308387
FALSO	High	Master Protein	P07602	Prosaposin OS=Homo sapiens (Human) OX=9606 GN=PSAP PE=1 SV=2	PSAP	58,1	0,88	0,529679171
FALSO	High	Master Protein	P02788	Lactotransferrin OS=Homo sapiens (Human) OX=9606 GN=LTF PE=1 SV=6	LTF	78,1	0,88	0,490416979
FALSO	High	None	P84085	ADP-ribosylation factor 5 OS=Homo sapiens (Human) OX=9606 GN=ARF5 PE=1 SV=2	ARF5	20,5	0,88	0,662956041
FALSO	High	Master Protein	Q9HB07	UPF0160 protein MYG1, mitochondrial OS=Homo sapiens (Human) OX=9606 GN=C12orf10 PE=1 SV=2	C12orf10	42,4	0,88	0,742989174
FALSO	High	Master Protein	Q14011	Cold-inducible RNA-binding protein OS=Homo sapiens (Human) OX=9606 GN=CIRBP PE=1 SV=1	CIRBP	18,6	0,87	0,73137394
FALSO	High	Master Protein	P00747	Plasminogen OS=Homo sapiens (Human) OX=9606 GN=PLG PE=1 SV=2	PLG	90,5	0,87	0,26047147
FALSO	High	Master Protein	O94903	Pyridoxal phosphate homeostasis protein OS=Homo sapiens (Human) OX=9606 GN=PLPBP PE=1 SV=1	PLPBP	30,3	0,87	0,54745982
FALSO	High	Master Protein	Q9H0E2	Toll-interacting protein OS=Homo sapiens (Human) OX=9606 GN=TOLLIP PE=1 SV=1	TOLLIP	30,3	0,87	0,471631044
FALSO	High	Master Protein	Q9P2T1	GMP reductase 2 OS=Homo sapiens (Human) OX=9606 GN=GMPR2 PE=1 SV=1	GMPR2	37,9	0,87	0,70948226

FALSO	High	Master Protein	P01834	Immunoglobulin kappa constant OS=Homo sapiens (Human) OX=9606 GN=IGKC PE=1 SV=2	IGKC	11,8	0,87	0,239375215
FALSO	High	Master Protein	B9A064	Immunoglobulin lambda-like polypeptide 5 OS=Homo sapiens (Human) OX=9606 GN=IGLL5 PE=2 SV=2	IGLL5	23	0,87	0,215285626
FALSO	High	Master Protein	P67809	Y-box-binding protein 1 OS=Homo sapiens (Human) OX=9606 GN=YBX1 PE=1 SV=3	YBX1	35,9	0,87	0,761599238
FALSO	High	Master Protein	Q15370	Elongin-B OS=Homo sapiens (Human) OX=9606 GN=ELOB PE=1 SV=1	ELOB	13,1	0,87	0,438482628
FALSO	High	Master Protein	P01599	Immunoglobulin kappa variable 1-17 OS=Homo sapiens (Human) OX=9606 GN=IGKV1-17 PE=1 SV=2	IGKV1-17	12,8	0,87	0,472097527
FALSO	Medium	Master Protein	Q14165	Malectin OS=Homo sapiens (Human) OX=9606 GN=MLEC PE=1 SV=1	MLEC	32,2	0,87	0,675247699
FALSO	High	None	O00148	ATP-dependent RNA helicase DDX39A OS=Homo sapiens (Human) OX=9606 GN=DDX39A PE=1 SV=2	DDX39A	49,1	0,87	0,448731481
FALSO	High	Master Protein	Q05655	Protein kinase C delta type OS=Homo sapiens (Human) OX=9606 GN=PRKCD PE=1 SV=2	PRKCD	77,5	0,87	0,545014739
FALSO	High	Master Protein	A0A0B4J1U7	Immunoglobulin heavy variable 6-1 OS=Homo sapiens (Human) OX=9606 GN=IGHV6-1 PE=3 SV=1	IGHV6-1	13,5	0,87	0,533221267
FALSO	High	Master Protein	P78371	T-complex protein 1 subunit beta OS=Homo sapiens (Human) OX=9606 GN=CCT2 PE=1 SV=4	CCT2	57,5	0,87	0,556960489
FALSO	High	Master Protein	P35527	Keratin, type I cytoskeletal 9 OS=Homo sapiens (Human) OX=9606 GN=KRT9 PE=1 SV=3	KRT9	62	0,86	0,745392824
FALSO	High	Master Protein	P25311	Zinc-alpha-2-glycoprotein OS=Homo sapiens (Human) OX=9606 GN=AZGP1 PE=1 SV=2	AZGP1	34,2	0,86	0,247843286
FALSO	High	Master Protein	P52272	Heterogeneous nuclear ribonucleoprotein M OS=Homo sapiens (Human) OX=9606 GN=HNRNPM PE=1 SV=3	HNRNPM	77,5	0,86	0,407224653
FALSO	High	Master Protein	Q969H8	Myeloid-derived growth factor OS=Homo sapiens (Human) OX=9606 GN=MYDGF PE=1 SV=1	MYDGF	18,8	0,86	0,531322238
FALSO	High	Master Protein	P49419	Alpha-aminoadipic semialdehyde dehydrogenase OS=Homo sapiens (Human) OX=9606 GN=ALDH7A1 PE=1 S	ALDH7A1	58,5	0,86	0,764631663
FALSO	High	Master Protein	Q9NTM9	Copper homeostasis protein cutC homolog OS=Homo sapiens (Human) OX=9606 GN=CUTC PE=1 SV=1	CUTC	29,3	0,86	0,420564199
FALSO	High	Master Protein	P51884	Lumican OS=Homo sapiens (Human) OX=9606 GN=LUM PE=1 SV=2	LUM	38,4	0,86	0,419346817
FALSO	High	Master Protein	P05452	Tetranectin OS=Homo sapiens (Human) OX=9606 GN=CLEC3B PE=1 SV=3	CLEC3B	22,5	0,86	0,419098901
FALSO	High	Master Protein	P06753	Tropomyosin alpha-3 chain OS=Homo sapiens (Human) OX=9606 GN=TPM3 PE=1 SV=2	TPM3	32,9	0,86	0,497471391
FALSO	High	Master Protein	P01859	Immunoglobulin heavy constant gamma 2 OS=Homo sapiens (Human) OX=9606 GN=IGHG2 PE=1 SV=2	IGHG2	35,9	0,86	0,142130311
FALSO	High	None	P35749	Myosin-11 OS=Homo sapiens (Human) OX=9606 GN=MYH11 PE=1 SV=3	MYH11	227	0,86	0,595381838
FALSO	High	None	P0CG04	Immunoglobulin lambda constant 1 OS=Homo sapiens (Human) OX=9606 GN=IGLC1 PE=1 SV=1	IGLC1	11,3	0,86	0,176839258
FALSO	High	Master Protein	P25705	ATP synthase subunit alpha, mitochondrial OS=Homo sapiens (Human) OX=9606 GN=ATP5F1A PE=1 SV=1	ATP5A1	59,7	0,86	0,549537496
FALSO	Medium	Master Protein	P26368	Splicing factor U2AF 65 kDa subunit OS=Homo sapiens (Human) OX=9606 GN=U2AF2 PE=1 SV=4	U2AF2	53,5	0,85	0,624098763
FALSO	High	None	P11678	Eosinophil peroxidase OS=Homo sapiens (Human) OX=9606 GN=EPX PE=1 SV=2	EPX	81	0,85	0,492224763
FALSO	High	Master Protein	P37837	Transaldolase OS=Homo sapiens (Human) OX=9606 GN=TALDO1 PE=1 SV=2	TALDO1	37,5	0,85	0,36446384
FALSO	High	Master Protein	Q96C19	EF-hand domain-containing protein D2 OS=Homo sapiens (Human) OX=9606 GN=EFHD2 PE=1 SV=1	EFHD2	26,7	0,85	0,537367105
FALSO	Medium	Master Protein	P46939	Utrophin OS=Homo sapiens (Human) OX=9606 GN=UTRN PE=1 SV=2	UTRN	394	0,85	0,634898334
FALSO	High	Master Protein	Q08ET2	Sialic acid-binding Ig-like lectin 14 OS=Homo sapiens (Human) OX=9606 GN=SIGLEC14 PE=1 SV=1	SIGLEC14	43,9	0,85	0,642723176
FALSO	High	Master Protein	O95336	6-phosphogluconolactonase OS=Homo sapiens (Human) OX=9606 GN=PGLS PE=1 SV=2	PGLS	27,5	0,85	0,27838608
FALSO	High	Master Protein	O95881	Thioredoxin domain-containing protein 12 OS=Homo sapiens (Human) OX=9606 GN=TXNDC12 PE=1 SV=1	TXNDC12	19,2	0,85	0,494262915
FALSO	High	Master Protein	P02749	Beta-2-glycoprotein 1 OS=Homo sapiens (Human) OX=9606 GN=APOH PE=1 SV=3	APOH	38,3	0,85	0,238267462
FALSO	High	Master Protein	P60842	Eukaryotic initiation factor 4A-I OS=Homo sapiens (Human) OX=9606 GN=EIF4A1 PE=1 SV=1	EIF4A1	46,1	0,85	0,241205514

FALSO	High	None	Q8WUD1	Ras-related protein Rab-2B OS=Homo sapiens (Human) OX=9606 GN=RAB2B PE=1 SV=1	RAB2B	24,2	0,85	0,521112938
FALSO	High	None	P24844	Myosin regulatory light polypeptide 9 OS=Homo sapiens (Human) OX=9606 GN=MYL9 PE=1 SV=4	MYL9	19,8	0,85	0,655583188
FALSO	High	Master Protein	O43504	Ragulator complex protein LAMTOR5 OS=Homo sapiens (Human) OX=9606 GN=LAMTOR5 PE=1 SV=1	LAMTOR5	9,6	0,85	0,615883326
FALSO	High	Master Protein	Q5T749	Keratinocyte proline-rich protein OS=Homo sapiens (Human) OX=9606 GN=KPRP PE=1 SV=1	KPRP	64,1	0,85	0,759309898
FALSO	High	Master Protein	Q16531	DNA damage-binding protein 1 OS=Homo sapiens (Human) OX=9606 GN=DDB1 PE=1 SV=1	DDB1	127	0,85	0,419973186
FALSO	High	Master Protein	P13796	Plastin-2 OS=Homo sapiens (Human) OX=9606 GN=LCP1 PE=1 SV=6	LCP1	70,2	0,85	0,220964697
FALSO	High	Master Protein	P03952	Plasma kallikrein OS=Homo sapiens (Human) OX=9606 GN=KLKB1 PE=1 SV=1	KLKB1	71,3	0,85	0,152402466
FALSO	High	Master Protein	P50552	Vasodilator-stimulated phosphoprotein OS=Homo sapiens (Human) OX=9606 GN=VASP PE=1 SV=3	VASP	39,8	0,85	0,498518106
FALSO	High	Master Protein	P05156	Complement factor I OS=Homo sapiens (Human) OX=9606 GN=CFI PE=1 SV=2	CFI	65,7	0,85	0,19635068
FALSO	High	Master Protein	P13671	Complement component C6 OS=Homo sapiens (Human) OX=9606 GN=C6 PE=1 SV=3	C6	105	0,85	0,192697667
FALSO	High	None	A0A2R8Y619	Histone H2B OS=Homo sapiens (Human) OX=9606 GN=LOC114483833 PE=1 SV=1	LOC114483833	13,5	0,85	0,431834752
FALSO	High	Master Protein	Q9NY33	Dipeptidyl peptidase 3 OS=Homo sapiens (Human) OX=9606 GN=DPP3 PE=1 SV=2	DPP3	82,5	0,85	0,459956759
FALSO	High	Master Protein	P36871	Phosphoglucomutase-1 OS=Homo sapiens (Human) OX=9606 GN=PGM1 PE=1 SV=3	PGM1	61,4	0,84	0,49770918
FALSO	High	Master Protein	P27635	60S ribosomal protein L10 OS=Homo sapiens (Human) OX=9606 GN=RPL10 PE=1 SV=4	RPL10	24,6	0,84	0,536717291
FALSO	High	Master Protein	Q9UNF0	Protein kinase C and casein kinase substrate in neurons protein 2 OS=Homo sapiens (Human) OX=9606 GN=I	PACSIN2	55,7	0,84	0,391059083
FALSO	High	Master Protein	P28072	Proteasome subunit beta type-6 OS=Homo sapiens (Human) OX=9606 GN=PSMB6 PE=1 SV=4	PSMB6	25,3	0,84	0,41490747
FALSO	High	Master Protein	O75348	V-type proton ATPase subunit G 1 OS=Homo sapiens (Human) OX=9606 GN=ATP6V1G1 PE=1 SV=3	ATP6V1G1	13,7	0,84	0,767154851
FALSO	High	Master Protein	Q9NP79	Vacuolar protein sorting-associated protein VTA1 homolog OS=Homo sapiens (Human) OX=9606 GN=VTA1 P	VTA1	33,9	0,84	0,57616332
FALSO	High	Master Protein	Q9UJ70	N-acetyl-D-glucosamine kinase OS=Homo sapiens (Human) OX=9606 GN=NAGK PE=1 SV=4	NAGK	37,4	0,84	0,449914239
FALSO	High	Master Protein	P05164	Myeloperoxidase OS=Homo sapiens (Human) OX=9606 GN=MPO PE=1 SV=1	MPO	83,8	0,84	0,354123672
FALSO	High	Master Protein	P78324	Tyrosine-protein phosphatase non-receptor type substrate 1 OS=Homo sapiens (Human) OX=9606 GN=SIRP/	SIRPA	54,9	0,84	0,511095143
FALSO	High	Master Protein	Q9NQ79	Cartilage acidic protein 1 OS=Homo sapiens (Human) OX=9606 GN=CRTAC1 PE=1 SV=2	CRTAC1	71,4	0,84	0,371382856
FALSO	High	Master Protein	P02774	Vitamin D-binding protein OS=Homo sapiens (Human) OX=9606 GN=GC PE=1 SV=2	GC	52,9	0,84	0,109917447
FALSO	High	Master Protein	P29401	Transketolase OS=Homo sapiens (Human) OX=9606 GN=TKT PE=1 SV=3	TKT	67,8	0,84	0,302611574
FALSO	High	None	Q96L46	Calpain small subunit 2 OS=Homo sapiens (Human) OX=9606 GN=CAPNS2 PE=2 SV=2	CAPNS2	27,6	0,84	0,533592432
FALSO	Medium	Master Protein	Q9Y6W5	Wiskott-Aldrich syndrome protein family member 2 OS=Homo sapiens (Human) OX=9606 GN=WASF2 PE=1 S\	WASF2	54,3	0,84	0,655404163
FALSO	High	Master Protein	P19652	Alpha-1-acid glycoprotein 2 OS=Homo sapiens (Human) OX=9606 GN=ORM2 PE=1 SV=2	ORM2	23,6	0,83	0,376171207
FALSO	High	Master Protein	P40227	T-complex protein 1 subunit zeta OS=Homo sapiens (Human) OX=9606 GN=CCT6A PE=1 SV=3	CCT6A	58	0,83	0,505389489
FALSO	Medium	Master Protein	P78406	mRNA export factor OS=Homo sapiens (Human) OX=9606 GN=RAE1 PE=1 SV=1	RAE1	40,9	0,83	0,384856448
FALSO	High	Master Protein	Q92597	Protein NDRG1 OS=Homo sapiens (Human) OX=9606 GN=NDRG1 PE=1 SV=1	NDRG1	42,8	0,83	0,843471735
FALSO	High	Master Protein	P04217	Alpha-1B-glycoprotein OS=Homo sapiens (Human) OX=9606 GN=A1BG PE=1 SV=4	A1BG	54,2	0,83	0,053174175
FALSO	Medium	Master Protein	Q16204	Coiled-coil domain-containing protein 6 OS=Homo sapiens (Human) OX=9606 GN=CCDC6 PE=1 SV=2	CCDC6	53,3	0,83	0,522831107
FALSO	High	Master Protein	P00748	Coagulation factor XII OS=Homo sapiens (Human) OX=9606 GN=F12 PE=1 SV=3	F12	67,7	0,83	0,317979703

FALSO	High	Master Protein	P30040	Endoplasmic reticulum resident protein 29 OS=Homo sapiens (Human) OX=9606 GN=ERP29 PE=1 SV=4	ERP29	29	0,83	0,189035314
FALSO	High	Master Protein	O75369	Filamin-B OS=Homo sapiens (Human) OX=9606 GN=FLNB PE=1 SV=2	FLNB	278	0,83	0,428443147
FALSO	High	Master Protein	P80188	Neutrophil gelatinase-associated lipocalin OS=Homo sapiens (Human) OX=9606 GN=LCN2 PE=1 SV=2	LCN2	22,6	0,83	0,551733293
FALSO	High	Master Protein	P05107	Integrin beta-2 OS=Homo sapiens (Human) OX=9606 GN=ITGB2 PE=1 SV=2	ITGB2	84,7	0,83	0,550746189
FALSO	High	Master Protein	P28066	Proteasome subunit alpha type-5 OS=Homo sapiens (Human) OX=9606 GN=PSMA5 PE=1 SV=3	PSMA5	26,4	0,83	0,326514733
FALSO	High	Master Protein	Q9NZK5	Adenosine deaminase 2 OS=Homo sapiens (Human) OX=9606 GN=ADA2 PE=1 SV=2	ADA2	58,9	0,83	0,356693234
FALSO	High	Master Protein	P02787	Serotransferrin OS=Homo sapiens (Human) OX=9606 GN=TF PE=1 SV=3	TF	77	0,83	0,037213586
FALSO	High	Master Protein	P51991	Heterogeneous nuclear ribonucleoprotein A3 OS=Homo sapiens (Human) OX=9606 GN=HNRNPA3 PE=1 SV=2	HNRNPA3	39,6	0,83	0,334152285
FALSO	High	Master Protein	P48507	Glutamate--cysteine ligase regulatory subunit OS=Homo sapiens (Human) OX=9606 GN=GCLM PE=1 SV=1	GCLM	30,7	0,83	0,394998675
FALSO	High	Master Protein	O15400	Syntaxin-7 OS=Homo sapiens (Human) OX=9606 GN=STX7 PE=1 SV=4	STX7	29,8	0,83	0,472142464
FALSO	High	Master Protein	Q15365	Poly(rC)-binding protein 1 OS=Homo sapiens (Human) OX=9606 GN=PCBP1 PE=1 SV=2	PCBP1	37,5	0,83	0,128160112
FALSO	High	None	P35580	Myosin-10 OS=Homo sapiens (Human) OX=9606 GN=MYH10 PE=1 SV=3	MYH10	229	0,83	0,544104361
FALSO	High	Master Protein	P20618	Proteasome subunit beta type-1 OS=Homo sapiens (Human) OX=9606 GN=PSMB1 PE=1 SV=2	PSMB1	26,5	0,83	0,326607846
FALSO	High	None	A0A0A0MRZ8	Immunoglobulin kappa variable 3D-11 OS=Homo sapiens (Human) OX=9606 GN=IGKV3D-11 PE=3 SV=6	IGKV3D-11	12,6	0,83	0,359732203
FALSO	Medium	None	Q9P1W8	Signal-regulatory protein gamma OS=Homo sapiens (Human) OX=9606 GN=SIRPG PE=1 SV=3	SIRPG	42,5	0,83	0,519915583
FALSO	Medium	None	O00241	Signal-regulatory protein beta-1 OS=Homo sapiens (Human) OX=9606 GN=SIRPB1 PE=1 SV=5	SIRPB1	43,2	0,83	0,519915583
FALSO	High	Master Protein	P0DP23	Calmodulin-1 OS=Homo sapiens (Human) OX=9606 GN=CALM1 PE=1 SV=1	CALM1	16,8	0,82	0,477835782
FALSO	High	Master Protein	P48740	Mannan-binding lectin serine protease 1 OS=Homo sapiens (Human) OX=9606 GN=MASP1 PE=1 SV=3	MASP1	79,2	0,82	0,508835797
FALSO	High	Master Protein	Q3ZCW2	Galectin-related protein OS=Homo sapiens (Human) OX=9606 GN=LGALSL PE=1 SV=2	LGALSL	19	0,82	0,305510658
FALSO	Medium	Master Protein	P50583	Bis(5'-nucleosyl)-tetraphosphatase [asymmetrical] OS=Homo sapiens (Human) OX=9606 GN=NUDT2 PE=1 SV	NUDT2	16,8	0,82	0,467140985
FALSO	High	Master Protein	P41218	Myeloid cell nuclear differentiation antigen OS=Homo sapiens (Human) OX=9606 GN=MNDA PE=1 SV=1	MNDA	45,8	0,82	0,254542026
FALSO	Medium	Master Protein	O00560	Syntenin-1 OS=Homo sapiens (Human) OX=9606 GN=SDCBP PE=1 SV=1	SDCBP	32,4	0,82	0,489882472
FALSO	Medium	None	Q2KJY2	Kinesin-like protein KIF26B OS=Homo sapiens (Human) OX=9606 GN=KIF26B PE=1 SV=1	KIF26B	224	0,82	0,491876815
FALSO	High	Master Protein	P04433	Immunoglobulin kappa variable 3-11 OS=Homo sapiens (Human) OX=9606 GN=IGKV3-11 PE=1 SV=1	IGKV3D-11	12,6	0,82	0,339661082
FALSO	Medium	Master Protein	O43598	2'-deoxynucleoside 5'-phosphate N-hydrolase 1 OS=Homo sapiens (Human) OX=9606 GN=DNPH1 PE=1 SV=	C6orf108; DNPH1	19,1	0,82	0,580358378
FALSO	High	Master Protein	P09871	Complement C1s subcomponent OS=Homo sapiens (Human) OX=9606 GN=C1S PE=1 SV=1	C1S	76,6	0,82	0,135509115
FALSO	High	Master Protein	O00754	Lysosomal alpha-mannosidase OS=Homo sapiens (Human) OX=9606 GN=MAN2B1 PE=1 SV=3	MAN2B1	114	0,82	0,668802246
FALSO	High	None	P20339	Ras-related protein Rab-5A OS=Homo sapiens (Human) OX=9606 GN=RAB5A PE=1 SV=2	RAB5A	23,6	0,82	0,449381316
FALSO	High	Master Protein	P16035	Metalloproteinase inhibitor 2 OS=Homo sapiens (Human) OX=9606 GN=TIMP2 PE=1 SV=2	TIMP2	24,4	0,82	0,349486881
FALSO	High	None	P62987	Ubiquitin-60S ribosomal protein L40 OS=Homo sapiens (Human) OX=9606 GN=UBA52 PE=1 SV=2	UBA52	14,7	0,82	0,632592084
FALSO	High	None	P0CG48	Polyubiquitin-C OS=Homo sapiens (Human) OX=9606 GN=UBC PE=1 SV=3	UBC	77	0,82	0,632592084
FALSO	High	None	P0CG47	Polyubiquitin-B OS=Homo sapiens (Human) OX=9606 GN=UBB PE=1 SV=1	UBB	25,7	0,82	0,632592084
FALSO	High	Master Protein	P80748	Immunoglobulin lambda variable 3-21 OS=Homo sapiens (Human) OX=9606 GN=IGLV3-21 PE=1 SV=2	IGLV3-21	12,4	0,82	0,40078191

FALSO	High	Master Protein	P51149	Ras-related protein Rab-7a OS=Homo sapiens (Human) OX=9606 GN=RAB7A PE=1 SV=1	RAB7A	23,5	0,82	0,265000824
FALSO	High	Master Protein	P84095	Rho-related GTP-binding protein RhoG OS=Homo sapiens (Human) OX=9606 GN=RHOG PE=1 SV=1	RHOG	21,3	0,82	0,572724806
FALSO	High	Master Protein	Q99832	T-complex protein 1 subunit eta OS=Homo sapiens (Human) OX=9606 GN=CCT7 PE=1 SV=2	CCT7	59,3	0,82	0,671909581
FALSO	High	Master Protein Candidat	A0A0A0MRZ7	Immunoglobulin kappa variable 2D-26 OS=Homo sapiens (Human) OX=9606 GN=IGKV2D-26 PE=3 SV=1	IGKV2D-26	13,3	0,82	0,487998588
FALSO	High	Master Protein	A2NJV5	Immunoglobulin kappa variable 2-29 OS=Homo sapiens (Human) OX=9606 GN=IGKV2-29 PE=3 SV=2	IGKV2-29	13,1	0,82	0,487998588
FALSO	High	Master Protein Candidat	A0A075B6S2	Immunoglobulin kappa variable 2D-29 OS=Homo sapiens (Human) OX=9606 GN=IGKV2D-29 PE=3 SV=1	IGKV2D-29	13,1	0,82	0,487998588
FALSO	High	Master Protein	P00734	Prothrombin OS=Homo sapiens (Human) OX=9606 GN=F2 PE=1 SV=2	F2	70	0,81	0,112105566
FALSO	High	Master Protein	P01880	Immunoglobulin heavy constant delta OS=Homo sapiens (Human) OX=9606 GN=IGHD PE=1 SV=3	IGHD	42,3	0,81	0,38923102
FALSO	High	Master Protein Candidat	P59666	Neutrophil defensin 3 OS=Homo sapiens (Human) OX=9606 GN=DEFA3 PE=1 SV=1	DEFA3	10,2	0,81	0,41356372
FALSO	High	Master Protein	P59665	Neutrophil defensin 1 OS=Homo sapiens (Human) OX=9606 GN=DEFA1 PE=1 SV=1	DEFA1	10,2	0,81	0,41356372
FALSO	High	Master Protein	Q03252	Lamin-B2 OS=Homo sapiens (Human) OX=9606 GN=LMNB2 PE=1 SV=4	LMNB2	69,9	0,81	0,436023489
FALSO	High	Master Protein	Q9P289	Serine/threonine-protein kinase 26 OS=Homo sapiens (Human) OX=9606 GN=STK26 PE=1 SV=2	MST4; STK26	46,5	0,81	0,470286806
FALSO	High	Master Protein	P11021	Endoplasmic reticulum chaperone BiP OS=Homo sapiens (Human) OX=9606 GN=HSPA5 PE=1 SV=2	HSPA5	72,3	0,81	0,218080274
FALSO	High	Master Protein	Q14103	Heterogeneous nuclear ribonucleoprotein D0 OS=Homo sapiens (Human) OX=9606 GN=HNRNPD PE=1 SV=1	HNRNPD	38,4	0,81	0,233145065
FALSO	High	Master Protein	P01877	Immunoglobulin heavy constant alpha 2 OS=Homo sapiens (Human) OX=9606 GN=IGHA2 PE=1 SV=4	IGHA2	36,6	0,81	0,320091723
FALSO	High	Master Protein	P02748	Complement component C9 OS=Homo sapiens (Human) OX=9606 GN=C9 PE=1 SV=2	C9	63,1	0,81	0,457985178
FALSO	High	Master Protein	P62937	Peptidyl-prolyl cis-trans isomerase A OS=Homo sapiens (Human) OX=9606 GN=PPIA PE=1 SV=2	PPIA	18	0,81	0,079908187
FALSO	High	Master Protein	P01876	Immunoglobulin heavy constant alpha 1 OS=Homo sapiens (Human) OX=9606 GN=IGHA1 PE=1 SV=2	IGHA1	37,6	0,81	0,138179125
FALSO	High	Master Protein	Q58FG1	Putative heat shock protein HSP 90-alpha A4 OS=Homo sapiens (Human) OX=9606 GN=HSP90AA4P PE=5 SV:	HSP90AA4P	84,6	0,81	0,333377012
FALSO	High	Master Protein	P13716	Delta-aminolevulinic acid dehydratase OS=Homo sapiens (Human) OX=9606 GN=ALAD PE=1 SV=1	ALAD	36,3	0,81	0,597598059
FALSO	High	Master Protein	P36578	60S ribosomal protein L4 OS=Homo sapiens (Human) OX=9606 GN=RPL4 PE=1 SV=5	RPL4	47,7	0,81	0,478715471
FALSO	High	Master Protein	P10599	Thioredoxin OS=Homo sapiens (Human) OX=9606 GN=TXN PE=1 SV=3	TXN	11,7	0,81	0,188775856
FALSO	High	Master Protein	P22392	Nucleoside diphosphate kinase B OS=Homo sapiens (Human) OX=9606 GN=NME2 PE=1 SV=1	NME2	17,3	0,81	0,297763474
FALSO	High	Master Protein	Q14247	Src substrate cortactin OS=Homo sapiens (Human) OX=9606 GN=CTTN PE=1 SV=2	CTTN	61,5	0,81	0,58847906
FALSO	High	Master Protein	P09467	Fructose-1,6-bisphosphatase 1 OS=Homo sapiens (Human) OX=9606 GN=FBP1 PE=1 SV=5	FBP1	36,8	0,81	0,326487846
FALSO	High	Master Protein	Q00839	Heterogeneous nuclear ribonucleoprotein U OS=Homo sapiens (Human) OX=9606 GN=HNRNPU PE=1 SV=6	HNRNPU	90,5	0,80	0,275307135
FALSO	High	Master Protein	P11279	Lysosome-associated membrane glycoprotein 1 OS=Homo sapiens (Human) OX=9606 GN=LAMP1 PE=1 SV=3	LAMP1	44,9	0,80	0,344267927
FALSO	High	Master Protein	Q01082	Spectrin beta chain, non-erythrocytic 1 OS=Homo sapiens (Human) OX=9606 GN=SPTBN1 PE=1 SV=2	SPTBN1	274	0,80	0,62748458
FALSO	High	Master Protein	Q9Y5X3	Sorting nexin-5 OS=Homo sapiens (Human) OX=9606 GN=SNX5 PE=1 SV=1	SNX5	46,8	0,80	0,330223867
FALSO	High	Master Protein	O60664	Perilipin-3 OS=Homo sapiens (Human) OX=9606 GN=PLIN3 PE=1 SV=3	PLIN3	47	0,80	0,37056416
FALSO	Medium	Master Protein	Q9Y266	Nuclear migration protein nudC OS=Homo sapiens (Human) OX=9606 GN=NUDC PE=1 SV=1	NUDC	38,2	0,80	0,44588595
FALSO	Medium	Master Protein	Q00013	55 kDa erythrocyte membrane protein OS=Homo sapiens (Human) OX=9606 GN=MPP1 PE=1 SV=2	MPP1	52,3	0,80	0,547043092
FALSO	High	Master Protein	P55263	Adenosine kinase OS=Homo sapiens (Human) OX=9606 GN=ADK PE=1 SV=2	ADK	40,5	0,80	0,288515911

FALSO	High	Master Protein	Q14651	Plastin-1 OS=Homo sapiens (Human) OX=9606 GN=PLS1 PE=1 SV=2	PLS1	70,2	0,80	0,439811191
FALSO	High	Master Protein	P38159	RNA-binding motif protein, X chromosome OS=Homo sapiens (Human) OX=9606 GN=RBMX PE=1 SV=3	RBMX	42,3	0,80	0,489169988
FALSO	High	Master Protein Candidat	P06731	Carcinoembryonic antigen-related cell adhesion molecule 5 OS=Homo sapiens (Human) OX=9606 GN=CEACAI	CEACAM5	76,7	0,80	0,2843165
FALSO	High	Master Protein Candidat	P31997	Carcinoembryonic antigen-related cell adhesion molecule 8 OS=Homo sapiens (Human) OX=9606 GN=CEACAI	CEACAM8	38,1	0,80	0,2843165
FALSO	High	Master Protein	P13688	Carcinoembryonic antigen-related cell adhesion molecule 1 OS=Homo sapiens (Human) OX=9606 GN=CEACAI	CEACAM1	57,5	0,80	0,2843165
FALSO	High	None	P04211	Immunoglobulin lambda variable 7-43 OS=Homo sapiens (Human) OX=9606 GN=IGLV7-43 PE=3 SV=2	IGLV7-43	12,4	0,80	0,324216092
FALSO	Medium	Master Protein	P20848	Putative alpha-1-antitrypsin-related protein OS=Homo sapiens (Human) OX=9606 GN=SERPINA2 PE=1 SV=1	SERPINA2	47,9	0,80	0,638130776
FALSO	High	Master Protein	A0A0J9YXX1	Immunoglobulin heavy variable 5-10-1 OS=Homo sapiens (Human) OX=9606 GN=IGHV5-10-1 PE=3 SV=1	IGHV5-A	12,8	0,80	0,220637863
FALSO	High	None	Q9NNW7	Thioredoxin reductase 2, mitochondrial OS=Homo sapiens (Human) OX=9606 GN=TXNRD2 PE=1 SV=3	TXNRD2	56,5	0,80	0,575268615
FALSO	High	Master Protein	P01601	Immunoglobulin kappa variable 1D-16 OS=Homo sapiens (Human) OX=9606 GN=IGKV1D-16 PE=3 SV=2	IGKV1D-16	12,7	0,80	0,38447341
FALSO	High	Master Protein	P08670	Vimentin OS=Homo sapiens (Human) OX=9606 GN=VIM PE=1 SV=4	VIM	53,6	0,79	0,365250964
FALSO	High	Master Protein	P32320	Cytidine deaminase OS=Homo sapiens (Human) OX=9606 GN=CDA PE=1 SV=2	CDA	16,2	0,79	0,233494064
FALSO	High	Master Protein	P17987	T-complex protein 1 subunit alpha OS=Homo sapiens (Human) OX=9606 GN=TCP1 PE=1 SV=1	TCP1	60,3	0,79	0,696334955
FALSO	High	Master Protein	P01709	Immunoglobulin lambda variable 2-8 OS=Homo sapiens (Human) OX=9606 GN=IGLV2-8 PE=1 SV=2	IGLV2-8	12,4	0,79	0,197331625
FALSO	High	Master Protein	P35579	Myosin-9 OS=Homo sapiens (Human) OX=9606 GN=MYH9 PE=1 SV=4	MYH9	226	0,79	0,394742907
FALSO	High	Master Protein	P62805	Histone H4 OS=Homo sapiens (Human) OX=9606 GN=H4C14 PE=1 SV=2	HIST1H4A	11,4	0,79	0,458293128
FALSO	High	Master Protein	Q12913	Receptor-type tyrosine-protein phosphatase eta OS=Homo sapiens (Human) OX=9606 GN=PTPRJ PE=1 SV=3	PTPRJ	146	0,79	0,455577496
FALSO	High	Master Protein	P20160	Azurocidin OS=Homo sapiens (Human) OX=9606 GN=AZU1 PE=1 SV=3	AZU1	26,9	0,79	0,390979282
FALSO	High	Master Protein	P01011	Alpha-1-antichymotrypsin OS=Homo sapiens (Human) OX=9606 GN=SERPINA3 PE=1 SV=2	SERPINA3	47,6	0,79	0,413127784
FALSO	High	Master Protein	P20061	Transcobalamin-1 OS=Homo sapiens (Human) OX=9606 GN=TCN1 PE=1 SV=2	TCN1	48,2	0,79	0,516368967
FALSO	High	Master Protein	P30566	Adenylosuccinate lyase OS=Homo sapiens (Human) OX=9606 GN=ADSL PE=1 SV=2	ADSL	54,9	0,79	0,386680268
FALSO	High	Master Protein	P60953	Cell division control protein 42 homolog OS=Homo sapiens (Human) OX=9606 GN=CDC42 PE=1 SV=2	CDC42	21,2	0,79	0,405910577
FALSO	High	Master Protein	P04083	Annexin A1 OS=Homo sapiens (Human) OX=9606 GN=ANXA1 PE=1 SV=2	ANXA1	38,7	0,79	0,210097846
FALSO	High	Master Protein	Q15843	NEDD8 OS=Homo sapiens (Human) OX=9606 GN=NEDD8 PE=1 SV=1	NEDD8	9,1	0,79	0,29221075
FALSO	High	Master Protein	P00736	Complement C1r subcomponent OS=Homo sapiens (Human) OX=9606 GN=C1R PE=1 SV=2	C1R	80,1	0,79	0,11701959
FALSO	High	Master Protein Candidat	Q9UKX2	Myosin-2 OS=Homo sapiens (Human) OX=9606 GN=MYH2 PE=1 SV=1	MYH2	223	0,79	0,431416078
FALSO	High	Master Protein	P12882	Myosin-1 OS=Homo sapiens (Human) OX=9606 GN=MYH1 PE=1 SV=3	MYH1	223	0,79	0,431416078
FALSO	High	Master Protein	P12838	Neutrophil defensin 4 OS=Homo sapiens (Human) OX=9606 GN=DEFA4 PE=1 SV=2	DEFA4	10,5	0,79	0,363820311
FALSO	High	Master Protein	Q99584	Protein S100-A13 OS=Homo sapiens (Human) OX=9606 GN=S100A13 PE=1 SV=1	S100A13	11,5	0,79	0,378045656
FALSO	High	Master Protein	P07954	Fumarate hydratase, mitochondrial OS=Homo sapiens (Human) OX=9606 GN=FH PE=1 SV=3	FH	54,6	0,79	0,399730673
FALSO	High	None	A0A2R8Y422	Ubiquitin-like domain-containing protein OS=Homo sapiens (Human) OX=9606 GN=ENSG00000231767 PE=4	ENSG0000023176	18	0,79	0,476919879
FALSO	Medium	Master Protein	A8TX70	Collagen alpha-5(VI) chain OS=Homo sapiens (Human) OX=9606 GN=COL6A5 PE=1 SV=1	COL6A5	290	0,79	0,503989623
FALSO	High	Master Protein	A0A075B6I9	Immunoglobulin lambda variable 7-46 OS=Homo sapiens (Human) OX=9606 GN=IGLV7-46 PE=3 SV=4	IGLV7-46	12,5	0,78	0,284970968

FALSO	High	Master Protein	P17936	Insulin-like growth factor-binding protein 3 OS=Homo sapiens (Human) OX=9606 GN=IGFBP3 PE=1 SV=2	IGFBP3	31,7	0,78	0,168626256
FALSO	High	None	O14556	Glyceraldehyde-3-phosphate dehydrogenase, testis-specific OS=Homo sapiens (Human) OX=9606 GN=GAPD	GAPDHS	44,5	0,78	0,386832009
FALSO	High	Master Protein	Q13464	Rho-associated protein kinase 1 OS=Homo sapiens (Human) OX=9606 GN=ROCK1 PE=1 SV=1	ROCK1	158	0,78	0,646894075
FALSO	High	Master Protein	O14974	Protein phosphatase 1 regulatory subunit 12A OS=Homo sapiens (Human) OX=9606 GN=PPP1R12A PE=1 SV	PPP1R12A	115	0,78	0,319055188
FALSO	High	Master Protein	Q13838	Spliceosome RNA helicase DDX39B OS=Homo sapiens (Human) OX=9606 GN=DDX39B PE=1 SV=1	DDX39B	49	0,78	0,159656803
FALSO	Medium	Master Protein	Q9Y5P6	Mannose-1-phosphate guanyltransferase beta OS=Homo sapiens (Human) OX=9606 GN=GMPPB PE=1 SV=2	GMPPB	39,8	0,78	0,342350597
FALSO	High	Master Protein	Q86V81	THO complex subunit 4 OS=Homo sapiens (Human) OX=9606 GN=ALYREF PE=1 SV=3	ALYREF	26,9	0,78	0,332368273
FALSO	Medium	Master Protein	Q8NFU4	Follicular dendritic cell secreted peptide OS=Homo sapiens (Human) OX=9606 GN=FDCSP PE=1 SV=1	FDCSP	9,7	0,78	0,592154969
FALSO	Medium	Master Protein	Q7Z5R6	Amyloid beta A4 precursor protein-binding family B member 1-interacting protein OS=Homo sapiens (Human	APBB1IP	73,1	0,78	0,528241247
FALSO	Medium	Master Protein	P13861	cAMP-dependent protein kinase type II-alpha regulatory subunit OS=Homo sapiens (Human) OX=9606 GN=P	PRKAR2A	45,5	0,78	0,215813421
FALSO	High	Master Protein	P42574	Caspase-3 OS=Homo sapiens (Human) OX=9606 GN=CASP3 PE=1 SV=2	CASP3	31,6	0,78	0,127505733
FALSO	High	Master Protein	Q14697	Neutral alpha-glucosidase AB OS=Homo sapiens (Human) OX=9606 GN=GANAB PE=1 SV=3	GANAB	107	0,78	0,309928231
FALSO	High	Master Protein	A0A0B4J1X8	Immunoglobulin heavy variable 3-43 OS=Homo sapiens (Human) OX=9606 GN=IGHV3-43 PE=3 SV=1	IGHV3-43	13,1	0,78	0,382585686
FALSO	Medium	Master Protein	P0C7U0	Protein ELFN1 OS=Homo sapiens (Human) OX=9606 GN=ELFN1 PE=1 SV=2	ELFN1	90,4	0,78	0,374238904
FALSO	High	Master Protein	Q13011	Delta(3,5)-Delta(2,4)-dienoyl-CoA isomerase, mitochondrial OS=Homo sapiens (Human) OX=9606 GN=ECH1	ECH1	35,8	0,78	0,380110181
FALSO	High	Master Protein	P04839	Cytochrome b-245 heavy chain OS=Homo sapiens (Human) OX=9606 GN=CYBB PE=1 SV=2	CYBB	65,3	0,78	0,385540672
FALSO	High	None	Q9P1U1	Actin-related protein 3B OS=Homo sapiens (Human) OX=9606 GN=ACTR3B PE=2 SV=1	ACTR3B	47,6	0,78	0,703522996
FALSO	High	Master Protein	P00367	Glutamate dehydrogenase 1, mitochondrial OS=Homo sapiens (Human) OX=9606 GN=GLUD1 PE=1 SV=2	GLUD1	61,4	0,77	0,168535056
FALSO	Medium	Master Protein	A0A0B4J1V2	Immunoglobulin heavy variable 2-26 OS=Homo sapiens (Human) OX=9606 GN=IGHV2-26 PE=3 SV=1	IGHV2-26	13,2	0,77	0,420794024
FALSO	High	Master Protein	P36980	Complement factor H-related protein 2 OS=Homo sapiens (Human) OX=9606 GN=CFHR2 PE=1 SV=1	CFHR2	30,6	0,77	0,309241093
FALSO	High	Master Protein	P35609	Alpha-actinin-2 OS=Homo sapiens (Human) OX=9606 GN=ACTN2 PE=1 SV=1	ACTN2	104	0,77	0,345158457
FALSO	High	None	Q08043	Alpha-actinin-3 OS=Homo sapiens (Human) OX=9606 GN=ACTN3 PE=1 SV=2	ACTN3	103	0,77	0,340698208
FALSO	High	Master Protein	Q13185	Chromobox protein homolog 3 OS=Homo sapiens (Human) OX=9606 GN=CBX3 PE=1 SV=4	CBX3	20,8	0,77	0,311872775
FALSO	High	Master Protein	Q5TBC7	Bcl-2-like protein 15 OS=Homo sapiens (Human) OX=9606 GN=BCL2L15 PE=1 SV=1	BCL2L15	17,7	0,77	0,392330295
FALSO	High	Master Protein	P02760	Protein AMBP OS=Homo sapiens (Human) OX=9606 GN=AMBP PE=1 SV=1	AMBP	39	0,77	0,008677416
FALSO	Medium	None	Q2QD12	Ribulose-phosphate 3-epimerase-like protein 1 OS=Homo sapiens (Human) OX=9606 GN=RPEL1 PE=2 SV=1	RPEL1	25	0,77	0,326690811
FALSO	High	Master Protein	A0A0C4DH31	Immunoglobulin heavy variable 1-18 OS=Homo sapiens (Human) OX=9606 GN=IGHV1-18 PE=3 SV=1	IGHV1-18	12,8	0,77	0,34626393
FALSO	High	None	P62736	Actin, aortic smooth muscle OS=Homo sapiens (Human) OX=9606 GN=ACTA2 PE=1 SV=1	ACTA2	42	0,77	0,323839184
FALSO	High	None	P63267	Actin, gamma-enteric smooth muscle OS=Homo sapiens (Human) OX=9606 GN=ACTG2 PE=1 SV=1	ACTG2	41,9	0,77	0,323839184
FALSO	High	None	Q58FF7	Putative heat shock protein HSP 90-beta-3 OS=Homo sapiens (Human) OX=9606 GN=HSP90AB3P PE=5 SV=1	HSP90AB3P	68,3	0,77	0,56771641
FALSO	High	Master Protein	P37108	Signal recognition particle 14 kDa protein OS=Homo sapiens (Human) OX=9606 GN=SRP14 PE=1 SV=2	SRP14	14,6	0,77	0,38596459
FALSO	High	Master Protein	O60218	Aldo-keto reductase family 1 member B10 OS=Homo sapiens (Human) OX=9606 GN=AKR1B10 PE=1 SV=2	AKR1B10	36	0,77	0,504981994
FALSO	High	Master Protein	Q9UBR2	Cathepsin Z OS=Homo sapiens (Human) OX=9606 GN=CTSZ PE=1 SV=1	CTSZ	33,8	0,77	0,156286941

FALSO	High	Master Protein	P02656	Apolipoprotein C-III OS=Homo sapiens (Human) OX=9606 GN=APOC3 PE=1 SV=1	APOC3	10,8	0,77	0,655637893
FALSO	High	Master Protein	Q16629	Serine/arginine-rich splicing factor 7 OS=Homo sapiens (Human) OX=9606 GN=SRSF7 PE=1 SV=1	SRSF7	27,4	0,77	0,305599905
FALSO	High	Master Protein	P13929	Beta-enolase OS=Homo sapiens (Human) OX=9606 GN=ENO3 PE=1 SV=5	ENO3	47	0,77	0,146302798
FALSO	High	Master Protein	Q08211	ATP-dependent RNA helicase A OS=Homo sapiens (Human) OX=9606 GN=DHX9 PE=1 SV=4	DHX9	141	0,77	0,284490914
FALSO	High	Master Protein	A0A075B6I0	Immunoglobulin lambda variable 8-61 OS=Homo sapiens (Human) OX=9606 GN=IGLV8-61 PE=3 SV=7	IGLV8-61	12,8	0,76	0,398322645
FALSO	Medium	Master Protein	Q9NPH3	Interleukin-1 receptor accessory protein OS=Homo sapiens (Human) OX=9606 GN=IL1RAP PE=1 SV=2	IL1RAP	65,4	0,76	0,428368938
FALSO	Medium	Master Protein	Q96A33	Coiled-coil domain-containing protein 47 OS=Homo sapiens (Human) OX=9606 GN=CCDC47 PE=1 SV=1	CCDC47	55,8	0,76	0,627534975
FALSO	High	Master Protein	Q15942	Zyxin OS=Homo sapiens (Human) OX=9606 GN=ZYX PE=1 SV=1	ZYX	61,2	0,76	0,30208351
FALSO	High	Master Protein	P05455	Lupus La protein OS=Homo sapiens (Human) OX=9606 GN=SSB PE=1 SV=2	SSB	46,8	0,76	0,103125772
FALSO	High	None	Q6NXT2	Histone H3.3C OS=Homo sapiens (Human) OX=9606 GN=H3F3C PE=1 SV=3	H3F3C	15,2	0,76	0,429788239
FALSO	Medium	Master Protein	P55212	Caspase-6 OS=Homo sapiens (Human) OX=9606 GN=CASP6 PE=1 SV=2	CASP6	33,3	0,76	0,098376948
FALSO	High	Master Protein	Q9P0L0	Vesicle-associated membrane protein-associated protein A OS=Homo sapiens (Human) OX=9606 GN=VAPA P	VAPA	27,9	0,76	0,102565729
FALSO	Medium	Master Protein	P48643	T-complex protein 1 subunit epsilon OS=Homo sapiens (Human) OX=9606 GN=CCT5 PE=1 SV=1	CCT5	59,6	0,76	0,359405162
FALSO	High	Master Protein	P07237	Protein disulfide-isomerase OS=Homo sapiens (Human) OX=9606 GN=P4HB PE=1 SV=3	P4HB	57,1	0,76	0,134211296
FALSO	High	Master Protein	P61020	Ras-related protein Rab-5B OS=Homo sapiens (Human) OX=9606 GN=RAB5B PE=1 SV=1	RAB5B	23,7	0,76	0,323029259
FALSO	Medium	Master Protein	Q08378	Golgin subfamily A member 3 OS=Homo sapiens (Human) OX=9606 GN=GOLGA3 PE=1 SV=2	GOLGA3	167	0,76	0,338649
FALSO	High	Master Protein	P61981	14-3-3 protein gamma OS=Homo sapiens (Human) OX=9606 GN=YWHAG PE=1 SV=2	YWHAG	28,3	0,76	0,163185892
FALSO	High	Master Protein	Q05707	Collagen alpha-1(XIV) chain OS=Homo sapiens (Human) OX=9606 GN=COL14A1 PE=1 SV=3	COL14A1	193	0,76	0,187379089
FALSO	Medium	Master Protein	Q92611	ER degradation-enhancing alpha-mannosidase-like protein 1 OS=Homo sapiens (Human) OX=9606 GN=EDEM	EDEM1	73,7	0,76	0,376817148
FALSO	Medium	Master Protein	P62906	60S ribosomal protein L10a OS=Homo sapiens (Human) OX=9606 GN=RPL10A PE=1 SV=2	RPL10A	24,8	0,76	0,302078626
FALSO	High	Master Protein	O00602	Ficolin-1 OS=Homo sapiens (Human) OX=9606 GN=FCN1 PE=1 SV=2	FCN1	35,1	0,76	0,303424635
FALSO	High	Master Protein	O43396	Thioredoxin-like protein 1 OS=Homo sapiens (Human) OX=9606 GN=TXNL1 PE=1 SV=3	TXNL1	32,2	0,76	0,077176769
FALSO	High	Master Protein	Q9BS26	Endoplasmic reticulum resident protein 44 OS=Homo sapiens (Human) OX=9606 GN=ERP44 PE=1 SV=1	ERP44	46,9	0,76	0,034466108
FALSO	High	None	P01767	Immunoglobulin heavy variable 3-53 OS=Homo sapiens (Human) OX=9606 GN=IGHV3-53 PE=1 SV=2	IGHV3-53	12,8	0,76	0,285282603
FALSO	High	None	A0A0C4DH42	Immunoglobulin heavy variable 3-66 OS=Homo sapiens (Human) OX=9606 GN=IGHV3-66 PE=3 SV=1	IGHV3-66	12,7	0,76	0,285282603
FALSO	High	Master Protein	P06727	Apolipoprotein A-IV OS=Homo sapiens (Human) OX=9606 GN=APOA4 PE=1 SV=3	APOA4	45,4	0,76	0,158785665
FALSO	High	Master Protein	A0A087WSX0	Immunoglobulin lambda variable 5-45 OS=Homo sapiens (Human) OX=9606 GN=IGLV5-45 PE=3 SV=1	IGLV5-45	13,2	0,76	0,359412774
FALSO	High	Master Protein Candidat	P20340	Ras-related protein Rab-6A OS=Homo sapiens (Human) OX=9606 GN=RAB6A PE=1 SV=3	RAB6A	23,6	0,75	0,12792659
FALSO	High	Master Protein	Q9NRW1	Ras-related protein Rab-6B OS=Homo sapiens (Human) OX=9606 GN=RAB6B PE=1 SV=1	RAB6B	23,4	0,75	0,12792659
FALSO	Medium	None	O95568	Histidine protein methyltransferase 1 homolog OS=Homo sapiens (Human) OX=9606 GN=METTL18 PE=1 SV=	METTL18	42,1	0,75	0,328976156
FALSO	High	Master Protein	P40926	Malate dehydrogenase, mitochondrial OS=Homo sapiens (Human) OX=9606 GN=MDH2 PE=1 SV=3	MDH2	35,5	0,75	0,124106349
FALSO	Medium	Master Protein	Q13303	Voltage-gated potassium channel subunit beta-2 OS=Homo sapiens (Human) OX=9606 GN=KCNAB2 PE=1 SV	KCNAB2	41	0,75	0,415131974
FALSO	Medium	Master Protein	P62280	40S ribosomal protein S11 OS=Homo sapiens (Human) OX=9606 GN=RPS11 PE=1 SV=3	RPS11	18,4	0,75	0,330642366

FALSO	High	None	Q14240	Eukaryotic initiation factor 4A-II OS=Homo sapiens (Human) OX=9606 GN=EIF4A2 PE=1 SV=2	EIF4A2	46,4	0,75	0,037558751
FALSO	Medium	Master Protein	O14841	5-oxoprolinase OS=Homo sapiens (Human) OX=9606 GN=OPLAH PE=1 SV=3	OPLAH	137	0,75	0,786715543
FALSO	High	Master Protein	P35268	60S ribosomal protein L22 OS=Homo sapiens (Human) OX=9606 GN=RPL22 PE=1 SV=2	RPL22	14,8	0,75	0,286691504
FALSO	High	Master Protein	Q9ULZ3	Apoptosis-associated speck-like protein containing a CARD OS=Homo sapiens (Human) OX=9606 GN=PYCAR	PYCARD	21,6	0,75	0,311325217
FALSO	High	Master Protein	P30838	Aldehyde dehydrogenase, dimeric NADP-preferring OS=Homo sapiens (Human) OX=9606 GN=ALDH3A1 PE=1	ALDH3A1	50,4	0,75	0,451031363
FALSO	High	Master Protein	P62979	Ubiquitin-40S ribosomal protein S27a OS=Homo sapiens (Human) OX=9606 GN=RPS27A PE=1 SV=2	RPS27A	18	0,75	0,322150919
FALSO	High	Master Protein	P27348	14-3-3 protein theta OS=Homo sapiens (Human) OX=9606 GN=YWHAQ PE=1 SV=1	YWHAQ	27,7	0,75	0,256858265
FALSO	High	Master Protein	Q04756	Hepatocyte growth factor activator OS=Homo sapiens (Human) OX=9606 GN=HGFAC PE=1 SV=1	HGFAC	70,6	0,75	0,053689723
FALSO	High	Master Protein	P04632	Calpain small subunit 1 OS=Homo sapiens (Human) OX=9606 GN=CAPNS1 PE=1 SV=1	CAPNS1	28,3	0,75	0,173241427
FALSO	High	None	Q9BUF5	Tubulin beta-6 chain OS=Homo sapiens (Human) OX=9606 GN=TUBB6 PE=1 SV=1	TUBB6	49,8	0,74	0,289046107
FALSO	High	Master Protein	P10809	60 kDa heat shock protein, mitochondrial OS=Homo sapiens (Human) OX=9606 GN=HSPD1 PE=1 SV=2	HSPD1	61	0,74	0,256517958
FALSO	High	Master Protein	Q14764	Major vault protein OS=Homo sapiens (Human) OX=9606 GN=MVP PE=1 SV=4	MVP	99,3	0,74	0,236463294
FALSO	High	Master Protein	P49747	Cartilage oligomeric matrix protein OS=Homo sapiens (Human) OX=9606 GN=COMP PE=1 SV=2	COMP	82,8	0,74	0,239366861
FALSO	High	Master Protein	Q9NP72	Ras-related protein Rab-18 OS=Homo sapiens (Human) OX=9606 GN=RAB18 PE=1 SV=1	RAB18	23	0,74	0,215162039
FALSO	High	Master Protein	P10768	S-formylglutathione hydrolase OS=Homo sapiens (Human) OX=9606 GN=ESD PE=1 SV=2	ESD	31,4	0,74	0,176325274
FALSO	High	None	Q14568	Heat shock protein HSP 90-alpha A2 OS=Homo sapiens (Human) OX=9606 GN=HSP90AA2P PE=1 SV=2	HSP90AA2	39,3	0,74	0,17241699
FALSO	High	Master Protein	P61247	40S ribosomal protein S3a OS=Homo sapiens (Human) OX=9606 GN=RPS3A PE=1 SV=2	RPS3A	29,9	0,74	0,476178709
FALSO	High	Master Protein	Q9Y4Z0	U6 snRNA-associated Sm-like protein LSm4 OS=Homo sapiens (Human) OX=9606 GN=LSM4 PE=1 SV=1	LSM4	15,3	0,74	0,091252009
FALSO	High	Master Protein	Q15149	Plectin OS=Homo sapiens (Human) OX=9606 GN=PLEC PE=1 SV=3	PLEC	532	0,74	0,321240639
FALSO	Medium	Master Protein	Q969S9	Ribosome-releasing factor 2, mitochondrial OS=Homo sapiens (Human) OX=9606 GN=GFM2 PE=1 SV=1	GFM2	86,5	0,74	0,345346839
FALSO	High	Master Protein	P10253	Lysosomal alpha-glucosidase OS=Homo sapiens (Human) OX=9606 GN=GAA PE=1 SV=4	GAA	105	0,74	0,077519179
FALSO	High	Master Protein	P02671	Fibrinogen alpha chain OS=Homo sapiens (Human) OX=9606 GN=FGA PE=1 SV=2	FGA	94,9	0,74	0,152817274
FALSO	High	Master Protein	Q14314	Fibroleukin OS=Homo sapiens (Human) OX=9606 GN=FGL2 PE=1 SV=1	FGL2	50,2	0,74	0,278610564
FALSO	High	Master Protein	P08637	Low affinity immunoglobulin gamma Fc region receptor III-A OS=Homo sapiens (Human) OX=9606 GN=FCGR3	FCGR3A	29,1	0,74	0,286227017
FALSO	High	Master Protein	Q03113	Guanine nucleotide-binding protein subunit alpha-12 OS=Homo sapiens (Human) OX=9606 GN=GNA12 PE=1	GNA12	44,3	0,74	0,16401271
FALSO	High	Master Protein Candidat	Q14344	Guanine nucleotide-binding protein subunit alpha-13 OS=Homo sapiens (Human) OX=9606 GN=GNA13 PE=1	GNA13	44	0,74	0,16401271
FALSO	High	Master Protein	Q96KN2	Beta-Ala-His dipeptidase OS=Homo sapiens (Human) OX=9606 GN=CNDP1 PE=1 SV=4	CNDP1	56,7	0,74	0,183887744
FALSO	High	Master Protein	O75390	Citrate synthase, mitochondrial OS=Homo sapiens (Human) OX=9606 GN=CS PE=1 SV=2	CS	51,7	0,74	0,2924613
FALSO	High	Master Protein	O00743	Serine/threonine-protein phosphatase 6 catalytic subunit OS=Homo sapiens (Human) OX=9606 GN=PPP6C P	PPP6C	35,1	0,73	0,098505767
FALSO	High	Master Protein	Q13231	Chitotriosidase-1 OS=Homo sapiens (Human) OX=9606 GN=CHIT1 PE=1 SV=1	CHIT1	51,6	0,73	0,456398814
FALSO	High	Master Protein	P07355	Annexin A2 OS=Homo sapiens (Human) OX=9606 GN=ANXA2 PE=1 SV=2	ANXA2	38,6	0,73	0,098082107
FALSO	High	Master Protein	P61626	Lysozyme C OS=Homo sapiens (Human) OX=9606 GN=LYZ PE=1 SV=1	LYZ	16,5	0,73	0,053870378
FALSO	High	Master Protein	S4R460	Ig-like domain-containing protein OS=Homo sapiens (Human) OX=9606 GN=IGHV3OR16-9 PE=1 SV=2	IGHV3OR16-9	10,4	0,73	0,116705004

FALSO	High	Master Protein	P08519	Apolipoprotein(a) OS=Homo sapiens (Human) OX=9606 GN=LPA PE=1 SV=1	LPA	501	0,73	0,358373724
FALSO	High	Master Protein	P04075	Fructose-bisphosphate aldolase A OS=Homo sapiens (Human) OX=9606 GN=ALDOA PE=1 SV=2	ALDOA	39,4	0,73	0,065890787
FALSO	High	Master Protein	P57721	Poly(rC)-binding protein 3 OS=Homo sapiens (Human) OX=9606 GN=PCBP3 PE=2 SV=2	PCBP3	39,4	0,73	0,327980737
FALSO	High	Master Protein Candidat	Q15366	Poly(rC)-binding protein 2 OS=Homo sapiens (Human) OX=9606 GN=PCBP2 PE=1 SV=1	PCBP2	38,6	0,73	0,327980737
FALSO	High	Master Protein	P29350	Tyrosine-protein phosphatase non-receptor type 6 OS=Homo sapiens (Human) OX=9606 GN=PTPN6 PE=1 SV	PTPN6	67,5	0,73	0,38114947
FALSO	High	Master Protein	P09497	Clathrin light chain B OS=Homo sapiens (Human) OX=9606 GN=CLTB PE=1 SV=1	CLTB	25,2	0,73	0,263835323
FALSO	High	Master Protein	P56537	Eukaryotic translation initiation factor 6 OS=Homo sapiens (Human) OX=9606 GN=EIF6 PE=1 SV=1	EIF6	26,6	0,73	0,250988558
FALSO	High	Master Protein	Q9GZP4	PITH domain-containing protein 1 OS=Homo sapiens (Human) OX=9606 GN=PITHD1 PE=1 SV=1	PITHD1	24,2	0,73	0,298502458
FALSO	High	Master Protein	P18827	Syndecan-1 OS=Homo sapiens (Human) OX=9606 GN=SDC1 PE=1 SV=3	SDC1	32,4	0,73	0,418608364
FALSO	High	Master Protein	P01703	Immunoglobulin lambda variable 1-40 OS=Homo sapiens (Human) OX=9606 GN=IGLV1-40 PE=1 SV=2	IGLV1-40	12,3	0,73	0,226053645
FALSO	High	Master Protein	P15121	Aldo-keto reductase family 1 member B1 OS=Homo sapiens (Human) OX=9606 GN=AKR1B1 PE=1 SV=3	AKR1B1	35,8	0,72	0,130599134
FALSO	High	Master Protein	P61978	Heterogeneous nuclear ribonucleoprotein K OS=Homo sapiens (Human) OX=9606 GN=HNRNPK PE=1 SV=1	HNRNPK	50,9	0,72	0,103781816
FALSO	High	Master Protein	P61158	Actin-related protein 3 OS=Homo sapiens (Human) OX=9606 GN=ACTR3 PE=1 SV=3	ACTR3	47,3	0,72	0,194271582
FALSO	High	Master Protein	Q9H4A4	Aminopeptidase B OS=Homo sapiens (Human) OX=9606 GN=RNPEP PE=1 SV=2	RNPEP	72,5	0,72	0,195853651
FALSO	High	Master Protein	Q99729	Heterogeneous nuclear ribonucleoprotein A/B OS=Homo sapiens (Human) OX=9606 GN=HNRNPAB PE=1 SV=	HNRNPAB	36,2	0,72	0,1713185
FALSO	High	None	P83916	Chromobox protein homolog 1 OS=Homo sapiens (Human) OX=9606 GN=CBX1 PE=1 SV=1	CBX1	21,4	0,72	0,39765567
FALSO	High	Master Protein	Q6WKZ4	Rab11 family-interacting protein 1 OS=Homo sapiens (Human) OX=9606 GN=RAB11FIP1 PE=1 SV=3	RAB11FIP1	137	0,72	0,192959214
FALSO	High	Master Protein	P62263	40S ribosomal protein S14 OS=Homo sapiens (Human) OX=9606 GN=RPS14 PE=1 SV=3	RPS14	16,3	0,72	0,33519547
FALSO	High	Master Protein	P34059	N-acetylgalactosamine-6-sulfatase OS=Homo sapiens (Human) OX=9606 GN=GALNS PE=1 SV=1	GALNS	58	0,72	0,28874533
FALSO	High	Master Protein	P39023	60S ribosomal protein L3 OS=Homo sapiens (Human) OX=9606 GN=RPL3 PE=1 SV=2	RPL3	46,1	0,72	0,477922988
FALSO	High	Master Protein	P01034	Cystatin-C OS=Homo sapiens (Human) OX=9606 GN=CST3 PE=1 SV=1	CST3	15,8	0,72	0,117825856
FALSO	High	Master Protein	P16403	Histone H1.2 OS=Homo sapiens (Human) OX=9606 GN=H1-2 PE=1 SV=2	HIST1H1C	21,4	0,72	0,274354992
FALSO	High	Master Protein	O00584	Ribonuclease T2 OS=Homo sapiens (Human) OX=9606 GN=RNASET2 PE=1 SV=2	RNASET2	29,5	0,72	0,106909668
FALSO	High	Master Protein	Q14847	LIM and SH3 domain protein 1 OS=Homo sapiens (Human) OX=9606 GN=LASP1 PE=1 SV=2	LASP1	29,7	0,72	0,144364973
FALSO	High	Master Protein	Q14498	RNA-binding protein 39 OS=Homo sapiens (Human) OX=9606 GN=RBM39 PE=1 SV=2	RBM39	59,3	0,71	0,056963114
FALSO	High	Master Protein	P01766	Immunoglobulin heavy variable 3-13 OS=Homo sapiens (Human) OX=9606 GN=IGHV3-13 PE=1 SV=2	IGHV3-13	12,5	0,71	0,117539547
FALSO	Medium	Master Protein	Q8NI51	Transcriptional repressor CTCFL OS=Homo sapiens (Human) OX=9606 GN=CTCFL PE=1 SV=2	CTCFL	75,7	0,71	0,193232273
FALSO	High	Master Protein	O95817	BAG family molecular chaperone regulator 3 OS=Homo sapiens (Human) OX=9606 GN=BAG3 PE=1 SV=3	BAG3	61,6	0,71	0,331201924
FALSO	High	Master Protein	P01699	Immunoglobulin lambda variable 1-44 OS=Homo sapiens (Human) OX=9606 GN=IGLV1-44 PE=1 SV=2	IGLV1-44	12,2	0,71	0,456295205
FALSO	High	Master Protein	P49189	4-trimethylaminobutyraldehyde dehydrogenase OS=Homo sapiens (Human) OX=9606 GN=ALDH9A1 PE=1 SV	ALDH9A1	53,8	0,71	0,164404012
FALSO	High	Master Protein	O60763	General vesicular transport factor p115 OS=Homo sapiens (Human) OX=9606 GN=USO1 PE=1 SV=2	USO1	108	0,71	0,146942599
FALSO	High	Master Protein	Q9ULV4	Coronin-1C OS=Homo sapiens (Human) OX=9606 GN=CORO1C PE=1 SV=1	CORO1C	53,2	0,71	0,168945363
FALSO	High	Master Protein	Q02413	Desmoglein-1 OS=Homo sapiens (Human) OX=9606 GN=DSG1 PE=1 SV=2	DSG1	114	0,71	0,326076743

FALSO	High	Master Protein	P34932	Heat shock 70 kDa protein 4 OS=Homo sapiens (Human) OX=9606 GN=HSPA4 PE=1 SV=4	HSPA4	94,3	0,71	0,124103681
FALSO	High	Master Protein	Q04917	14-3-3 protein eta OS=Homo sapiens (Human) OX=9606 GN=YWHAH PE=1 SV=4	YWHAH	28,2	0,71	0,181958268
FALSO	High	Master Protein	Q7Z4W1	L-xylulose reductase OS=Homo sapiens (Human) OX=9606 GN=DCXR PE=1 SV=2	DCXR	25,9	0,71	0,14507745
FALSO	High	Master Protein	P63104	14-3-3 protein zeta/delta OS=Homo sapiens (Human) OX=9606 GN=YWHAZ PE=1 SV=1	YWHAZ	27,7	0,71	0,095496647
FALSO	High	Master Protein	P31153	S-adenosylmethionine synthase isoform type-2 OS=Homo sapiens (Human) OX=9606 GN=MAT2A PE=1 SV=1	MAT2A	43,6	0,71	0,251842774
FALSO	High	Master Protein	P07339	Cathepsin D OS=Homo sapiens (Human) OX=9606 GN=CTSD PE=1 SV=1	CTSD	44,5	0,71	0,143512426
FALSO	High	Master Protein	Q13347	Eukaryotic translation initiation factor 3 subunit I OS=Homo sapiens (Human) OX=9606 GN=EIF3I PE=1 SV=1	EIF3I	36,5	0,71	0,270932274
FALSO	High	None	A6NMY6	Putative annexin A2-like protein OS=Homo sapiens (Human) OX=9606 GN=ANXA2P2 PE=5 SV=2	ANXA2P2	38,6	0,71	0,069396426
FALSO	High	Master Protein	P13639	Elongation factor 2 OS=Homo sapiens (Human) OX=9606 GN=EEF2 PE=1 SV=4	EEF2	95,3	0,71	0,034580753
FALSO	High	Master Protein	P14618	Pyruvate kinase PKM OS=Homo sapiens (Human) OX=9606 GN=PKM PE=1 SV=4	PKM	57,9	0,71	0,029893451
FALSO	High	Master Protein	P30419	Glycylpeptide N-tetradecanoyltransferase 1 OS=Homo sapiens (Human) OX=9606 GN=NMT1 PE=1 SV=2	NMT1	56,8	0,70	0,074866994
FALSO	High	Master Protein	Q09666	Neuroblast differentiation-associated protein AHNAK OS=Homo sapiens (Human) OX=9606 GN=AHNAK PE=1	AHNAK	629	0,70	0,070756655
FALSO	High	None	Q58FF8	Putative heat shock protein HSP 90-beta 2 OS=Homo sapiens (Human) OX=9606 GN=HSP90AB2P PE=1 SV=2	HSP90AB2P	44,3	0,70	0,116227781
FALSO	High	Master Protein	P02747	Complement C1q subcomponent subunit C OS=Homo sapiens (Human) OX=9606 GN=C1QC PE=1 SV=3	C1QC	25,8	0,70	0,277682605
FALSO	High	Master Protein	P08238	Heat shock protein HSP 90-beta OS=Homo sapiens (Human) OX=9606 GN=HSP90AB1 PE=1 SV=4	HSP90AB1	83,2	0,70	0,296528558
FALSO	High	Master Protein	P35659	Protein DEK OS=Homo sapiens (Human) OX=9606 GN=DEK PE=1 SV=1	DEK	42,6	0,70	0,169485015
FALSO	High	None	Q9BYE4	Small proline-rich protein 2G OS=Homo sapiens (Human) OX=9606 GN=SPRR2G PE=3 SV=1	SPRR2G	8,2	0,70	0,179218856
FALSO	High	Master Protein	Q6JBY9	CapZ-interacting protein OS=Homo sapiens (Human) OX=9606 GN=RCSD1 PE=1 SV=1	RCSD1	44,5	0,70	0,379790784
FALSO	High	Master Protein	P08758	Annexin A5 OS=Homo sapiens (Human) OX=9606 GN=ANXA5 PE=1 SV=2	ANXA5	35,9	0,70	0,064410231
FALSO	High	Master Protein	P62753	40S ribosomal protein S6 OS=Homo sapiens (Human) OX=9606 GN=RPS6 PE=1 SV=1	RPS6	28,7	0,70	0,24119829
FALSO	High	None	A0A0C4DH55	Immunoglobulin kappa variable 3D-7 OS=Homo sapiens (Human) OX=9606 GN=IGKV3D-7 PE=3 SV=5	IGKV3D-7	13,1	0,70	0,061859597
FALSO	High	None	A0A0C4DH90	Ig-like domain-containing protein OS=Homo sapiens (Human) OX=9606 GN=IGKV3OR2-268 PE=4 SV=5	IGKV3OR2-268	12,6	0,70	0,061859597
FALSO	High	Master Protein	Q9UBC9	Small proline-rich protein 3 OS=Homo sapiens (Human) OX=9606 GN=SPRR3 PE=1 SV=2	SPRR3	18,1	0,70	0,222337026
FALSO	High	Master Protein	A0A0B4J2D9	Immunoglobulin kappa variable 1D-13 OS=Homo sapiens (Human) OX=9606 GN=IGKV1D-13 PE=3 SV=1	IGKV1-13	12,6	0,70	0,473399282
FALSO	High	Master Protein	P35908	Keratin, type II cytoskeletal 2 epidermal OS=Homo sapiens (Human) OX=9606 GN=KRT2 PE=1 SV=2	KRT2	65,4	0,70	0,240117569
FALSO	Medium	None	Q9Y3E1	Hepatoma-derived growth factor-related protein 3 OS=Homo sapiens (Human) OX=9606 GN=HDGFL3 PE=1 S	HDGFRP3; HDGFL	22,6	0,70	0,411176517
FALSO	Medium	None	O75475	PC4 and SFRS1-interacting protein OS=Homo sapiens (Human) OX=9606 GN=PSIP1 PE=1 SV=1	PSIP1	60,1	0,70	0,411176517
FALSO	Medium	None	Q7Z4V5	Hepatoma-derived growth factor-related protein 2 OS=Homo sapiens (Human) OX=9606 GN=HDGFL2 PE=1 S	HDGFRP2; HDGFL	74,3	0,70	0,411176517
FALSO	High	Master Protein	P01008	Antithrombin-III OS=Homo sapiens (Human) OX=9606 GN=SERPINC1 PE=1 SV=1	SERPINC1	52,6	0,69	0,095044486
FALSO	Medium	Master Protein	Q96C10	Probable ATP-dependent RNA helicase DHX58 OS=Homo sapiens (Human) OX=9606 GN=DHX58 PE=1 SV=1	DHX58	76,6	0,69	0,342705245
FALSO	High	Master Protein	P28676	Grancalcin OS=Homo sapiens (Human) OX=9606 GN=GCA PE=1 SV=2	GCA	24	0,69	0,168919479
FALSO	High	Master Protein	P02452	Collagen alpha-1(I) chain OS=Homo sapiens (Human) OX=9606 GN=COL1A1 PE=1 SV=5	COL1A1	139	0,69	0,141947855
FALSO	High	Master Protein	P13489	Ribonuclease inhibitor OS=Homo sapiens (Human) OX=9606 GN=RNH1 PE=1 SV=2	RNH1	49,9	0,69	0,192788963

FALSO	Medium	Master Protein	P53609	Geranylgeranyl transferase type-1 subunit beta OS=Homo sapiens (Human) OX=9606 GN=PGGT1B PE=1 SV=2	PGGT1B	42,3	0,69	0,163771183
FALSO	High	Master Protein	A0A075B6H7	Probable non-functional immunoglobulin kappa variable 3-7 OS=Homo sapiens (Human) OX=9606 GN=IGKV3	IGKV3-7	12,8	0,69	0,026581117
FALSO	High	Master Protein	P16152	Carbonyl reductase [NADPH] 1 OS=Homo sapiens (Human) OX=9606 GN=CBR1 PE=1 SV=3	CBR1	30,4	0,69	0,036418114
FALSO	High	Master Protein	P13928	Annexin A8 OS=Homo sapiens (Human) OX=9606 GN=ANXA8 PE=1 SV=3	ANXA8; ANXA8L1	36,9	0,69	0,035172408
FALSO	High	Master Protein	Q8WU39	Marginal zone B- and B1-cell-specific protein OS=Homo sapiens (Human) OX=9606 GN=MZB1 PE=1 SV=1	MZB1	20,7	0,69	0,350168494
FALSO	Medium	Master Protein	O43670	BUB3-interacting and GLEBS motif-containing protein ZNF207 OS=Homo sapiens (Human) OX=9606 GN=ZNF	ZNF207	50,7	0,69	0,191773855
FALSO	High	Master Protein	O14745	Na(+)/H(+) exchange regulatory cofactor NHE-RF1 OS=Homo sapiens (Human) OX=9606 GN=SLC9A3R1 PE=1	SLC9A3R1	38,8	0,69	0,021432997
FALSO	High	Master Protein	P31946	14-3-3 protein beta/alpha OS=Homo sapiens (Human) OX=9606 GN=YWHAB PE=1 SV=3	YWHAB	28,1	0,69	0,127008131
FALSO	High	Master Protein	P09493	Tropomyosin alpha-1 chain OS=Homo sapiens (Human) OX=9606 GN=TPM1 PE=1 SV=2	TPM1	32,7	0,69	0,292935325
FALSO	High	Master Protein	P13667	Protein disulfide-isomerase A4 OS=Homo sapiens (Human) OX=9606 GN=PDIA4 PE=1 SV=2	PDIA4	72,9	0,69	0,067558518
FALSO	High	Master Protein	Q9UKM9	RNA-binding protein Raly OS=Homo sapiens (Human) OX=9606 GN=RALY PE=1 SV=1	RALY	32,4	0,69	0,24606924
FALSO	High	Master Protein	Q9UHV9	Prefoldin subunit 2 OS=Homo sapiens (Human) OX=9606 GN=PFDN2 PE=1 SV=1	PFDN2	16,6	0,68	0,045085485
FALSO	High	Master Protein	P20851	C4b-binding protein beta chain OS=Homo sapiens (Human) OX=9606 GN=C4BPB PE=1 SV=1	C4BPB	28,3	0,68	0,084089831
FALSO	High	None	P01597	Immunoglobulin kappa variable 1-39 OS=Homo sapiens (Human) OX=9606 GN=IGKV1-39 PE=1 SV=2	IGKV1-39	12,7	0,68	0,229874167
FALSO	High	Master Protein Candidat	P01611	Immunoglobulin kappa variable 1D-12 OS=Homo sapiens (Human) OX=9606 GN=IGKV1D-12 PE=1 SV=2	IGKV1D-12	12,6	0,68	0,226034443
FALSO	High	Master Protein	A0A0C4DH73	Immunoglobulin kappa variable 1-12 OS=Homo sapiens (Human) OX=9606 GN=IGKV1-12 PE=3 SV=1	IGKV1-12	12,6	0,68	0,226034443
FALSO	High	Master Protein	P01700	Immunoglobulin lambda variable 1-47 OS=Homo sapiens (Human) OX=9606 GN=IGLV1-47 PE=1 SV=2	IGLV1-47	12,3	0,68	0,055036241
FALSO	High	Master Protein	P61604	10 kDa heat shock protein, mitochondrial OS=Homo sapiens (Human) OX=9606 GN=HSPE1 PE=1 SV=2	HSPE1	10,9	0,68	0,043764221
FALSO	High	Master Protein	O75131	Copine-3 OS=Homo sapiens (Human) OX=9606 GN=CPNE3 PE=1 SV=1	CPNE3	60,1	0,68	0,334426313
FALSO	High	Master Protein	P23284	Peptidyl-prolyl cis-trans isomerase B OS=Homo sapiens (Human) OX=9606 GN=PPIB PE=1 SV=2	PPIB	23,7	0,68	0,030538288
FALSO	High	Master Protein	Q01469	Fatty acid-binding protein 5 OS=Homo sapiens (Human) OX=9606 GN=FABP5 PE=1 SV=3	FABP5	15,2	0,68	0,283481852
FALSO	High	None	Q96E39	RNA binding motif protein, X-linked-like-1 OS=Homo sapiens (Human) OX=9606 GN=RBMXL1 PE=1 SV=1	RBMXL1	42,1	0,68	0,180762836
FALSO	High	Master Protein	Q15052	Rho guanine nucleotide exchange factor 6 OS=Homo sapiens (Human) OX=9606 GN=ARHGEF6 PE=1 SV=2	ARHGEF6	87,4	0,68	0,081787932
FALSO	High	Master Protein	P04070	Vitamin K-dependent protein C OS=Homo sapiens (Human) OX=9606 GN=PROC PE=1 SV=1	PROC	52	0,67	0,507792734
FALSO	High	Master Protein	P0DJI8	Serum amyloid A-1 protein OS=Homo sapiens (Human) OX=9606 GN=SAA1 PE=1 SV=1	SAA1	13,5	0,67	0,533406454
FALSO	High	Master Protein	P04003	C4b-binding protein alpha chain OS=Homo sapiens (Human) OX=9606 GN=C4BPA PE=1 SV=2	C4BPA	67	0,67	0,21765777
FALSO	High	None	P0DJI9	Serum amyloid A-2 protein OS=Homo sapiens (Human) OX=9606 GN=SAA2 PE=1 SV=1	SAA2	13,5	0,67	0,47797439
FALSO	High	Master Protein	O43516	WAS/WASL-interacting protein family member 1 OS=Homo sapiens (Human) OX=9606 GN=WIPF1 PE=1 SV=3	WIPF1	51,2	0,67	0,132753081
FALSO	High	Master Protein	A0A075B7D0	Ig-like domain-containing protein OS=Homo sapiens (Human) OX=9606 GN=IGHV1OR15-1 PE=1 SV=1	IGHV1OR15-1	13	0,67	0,136819804
FALSO	High	Master Protein	P01706	Immunoglobulin lambda variable 2-11 OS=Homo sapiens (Human) OX=9606 GN=IGLV2-11 PE=1 SV=2	IGLV2-11	12,6	0,67	0,277945948
FALSO	High	Master Protein	Q9UK45	U6 snRNA-associated Sm-like protein LSm7 OS=Homo sapiens (Human) OX=9606 GN=LSM7 PE=1 SV=1	LSM7	11,6	0,67	0,194088183
FALSO	High	Master Protein	P43034	Platelet-activating factor acetylhydrolase IB subunit alpha OS=Homo sapiens (Human) OX=9606 GN=PAFAH1I	PAFAH1B1	46,6	0,67	0,176918119
FALSO	High	Master Protein	P02746	Complement C1q subcomponent subunit B OS=Homo sapiens (Human) OX=9606 GN=C1QB PE=1 SV=3	C1QB	26,7	0,67	0,073153244

FALSO	High	Master Protein	P19338	Nucleolin OS=Homo sapiens (Human) OX=9606 GN=NCL PE=1 SV=3	NCL	76,6	0,67	0,025975526
FALSO	Medium	Master Protein	Q9UJ72	Annexin A10 OS=Homo sapiens (Human) OX=9606 GN=ANXA10 PE=1 SV=3	ANXA10	37,3	0,67	0,065678395
FALSO	High	Master Protein	P55008	Allograft inflammatory factor 1 OS=Homo sapiens (Human) OX=9606 GN=AIF1 PE=1 SV=1	AIF1	16,7	0,67	0,335069331
FALSO	High	Master Protein	Q9UNZ2	NSFL1 cofactor p47 OS=Homo sapiens (Human) OX=9606 GN=NSFL1C PE=1 SV=2	NSFL1C	40,5	0,67	0,065893655
FALSO	High	Master Protein	P15289	Arylsulfatase A OS=Homo sapiens (Human) OX=9606 GN=ARSA PE=1 SV=3	ARSA	53,6	0,66	0,162156883
FALSO	Medium	Master Protein	A4D0S4	Laminin subunit beta-4 OS=Homo sapiens (Human) OX=9606 GN=LAMB4 PE=2 SV=1	LAMB4	193	0,66	0,118294604
FALSO	Medium	Master Protein	O95477	Phospholipid-transporting ATPase ABCA1 OS=Homo sapiens (Human) OX=9606 GN=ABCA1 PE=1 SV=3	ABCA1	254	0,66	0,18230729
FALSO	High	Master Protein	P19971	Thymidine phosphorylase OS=Homo sapiens (Human) OX=9606 GN=TYMP PE=1 SV=2	TYMP	49,9	0,66	0,076759626
FALSO	High	Master Protein	Q9BUJ2	Heterogeneous nuclear ribonucleoprotein U-like protein 1 OS=Homo sapiens (Human) OX=9606 GN=HNRNPU	HNRNPUL1	95,7	0,66	0,042843908
FALSO	High	Master Protein	Q14914	Prostaglandin reductase 1 OS=Homo sapiens (Human) OX=9606 GN=PTGR1 PE=1 SV=2	PTGR1	35,8	0,66	0,080884311
FALSO	High	None	O75830	Serpin I2 OS=Homo sapiens (Human) OX=9606 GN=SERPINI2 PE=1 SV=1	SERPINI2	46,1	0,66	0,234424971
FALSO	High	None	P50452	Serpin B8 OS=Homo sapiens (Human) OX=9606 GN=SERPINB8 PE=1 SV=2	SERPINB8	42,7	0,66	0,234424971
FALSO	High	None	P50453	Serpin B9 OS=Homo sapiens (Human) OX=9606 GN=SERPINB9 PE=1 SV=1	SERPINB9	42,4	0,66	0,234424971
FALSO	Medium	Master Protein	A0A0A0MT96	Immunoglobulin kappa joining 3 OS=Homo sapiens (Human) OX=9606 GN=IGKJ3 PE=4 SV=1	IGKJ3	1,4	0,66	0,253037343
FALSO	High	Master Protein	P67936	Tropomyosin alpha-4 chain OS=Homo sapiens (Human) OX=9606 GN=TPM4 PE=1 SV=3	TPM4	28,5	0,66	0,101030424
FALSO	High	Master Protein	P07858	Cathepsin B OS=Homo sapiens (Human) OX=9606 GN=CTSB PE=1 SV=3	CTSB	37,8	0,66	0,113486614
FALSO	Medium	Master Protein	Q9Y4Y9	U6 snRNA-associated Sm-like protein LSm5 OS=Homo sapiens (Human) OX=9606 GN=LSM5 PE=1 SV=3	LSM5	9,9	0,65	0,243085844
FALSO	Medium	Master Protein	Q96B97	SH3 domain-containing kinase-binding protein 1 OS=Homo sapiens (Human) OX=9606 GN=SH3KBP1 PE=1 SV	SH3KBP1	73,1	0,65	0,155888779
FALSO	High	Master Protein	Q92882	Osteoclast-stimulating factor 1 OS=Homo sapiens (Human) OX=9606 GN=OSTF1 PE=1 SV=2	OSTF1	23,8	0,65	0,015062337
FALSO	High	Master Protein	P43487	Ran-specific GTPase-activating protein OS=Homo sapiens (Human) OX=9606 GN=RANBP1 PE=1 SV=1	RANBP1	23,3	0,65	0,082343865
FALSO	High	Master Protein Candidat	P22105	Tenascin-X OS=Homo sapiens (Human) OX=9606 GN=TNXB PE=1 SV=5	TNCX	458	0,65	#¡DIV/0!
FALSO	High	Master Protein	Q16473	Putative tenascin-XA OS=Homo sapiens (Human) OX=9606 GN=TNXA PE=5 SV=2	TNXA	33,7	0,65	#¡DIV/0!
FALSO	Medium	Master Protein	Q9Y5S9	RNA-binding protein 8A OS=Homo sapiens (Human) OX=9606 GN=RBM8A PE=1 SV=1	RBM8A	19,9	0,65	#¡DIV/0!
FALSO	High	Master Protein	P20700	Lamin-B1 OS=Homo sapiens (Human) OX=9606 GN=LMNB1 PE=1 SV=2	LMNB1	66,4	0,65	0,083889471
FALSO	High	Master Protein	Q9BPX5	Actin-related protein 2/3 complex subunit 5-like protein OS=Homo sapiens (Human) OX=9606 GN=ARPC5L P	ARPC5L	16,9	0,65	0,243073371
FALSO	High	None	P17081	Rho-related GTP-binding protein RhoQ OS=Homo sapiens (Human) OX=9606 GN=RHOQ PE=1 SV=2	RHOQ	22,6	0,65	0,194141547
FALSO	High	None	Q9H4E5	Rho-related GTP-binding protein RhoJ OS=Homo sapiens (Human) OX=9606 GN=RHOJ PE=1 SV=1	RHOJ	23,8	0,65	0,194141547
FALSO	Medium	Master Protein	Q8TDG2	Actin-related protein T1 OS=Homo sapiens (Human) OX=9606 GN=ACTRT1 PE=2 SV=2	ACTRT1	41,7	0,65	0,341210141
FALSO	High	None	P20702	Integrin alpha-X OS=Homo sapiens (Human) OX=9606 GN=ITGAX PE=1 SV=3	ITGAX	128	0,65	0,120624657
FALSO	High	None	Q13349	Integrin alpha-D OS=Homo sapiens (Human) OX=9606 GN=ITGAD PE=1 SV=2	ITGAD	127	0,65	0,120624657
FALSO	High	Master Protein	Q9Y5Z4	Heme-binding protein 2 OS=Homo sapiens (Human) OX=9606 GN=HEBP2 PE=1 SV=1	HEBP2	22,9	0,65	0,049964532
FALSO	High	Master Protein	P05155	Plasma protease C1 inhibitor OS=Homo sapiens (Human) OX=9606 GN=SERPING1 PE=1 SV=2	SERPING1	55,1	0,64	0,038329844
FALSO	High	Master Protein	Q08380	Galectin-3-binding protein OS=Homo sapiens (Human) OX=9606 GN=LGALS3BP PE=1 SV=1	LGALS3BP	65,3	0,64	0,039933427

FALSO	High	Master Protein	A0A0B4J1X5	Immunoglobulin heavy variable 3-74 OS=Homo sapiens (Human) OX=9606 GN=IGHV3-74 PE=3 SV=1	IGHV3-74	12,8	0,64	0,009346281
FALSO	High	Master Protein	P23396	40S ribosomal protein S3 OS=Homo sapiens (Human) OX=9606 GN=RPS3 PE=1 SV=2	RPS3	26,7	0,64	0,297726333
FALSO	Medium	Master Protein	Q9Y5Y7	Lymphatic vessel endothelial hyaluronic acid receptor 1 OS=Homo sapiens (Human) OX=9606 GN=LYVE1 PE=1	LYVE1	35,2	0,64	0,025704935
FALSO	High	Master Protein	O75832	26S proteasome non-ATPase regulatory subunit 10 OS=Homo sapiens (Human) OX=9606 GN=PSMD10 PE=1	PSMD10	24,4	0,64	0,055182328
FALSO	High	Master Protein	P21399	Cytoplasmic aconitate hydratase OS=Homo sapiens (Human) OX=9606 GN=ACO1 PE=1 SV=3	ACO1	98,3	0,64	0,100195398
FALSO	High	Master Protein	P01701	Immunoglobulin lambda variable 1-51 OS=Homo sapiens (Human) OX=9606 GN=IGLV1-51 PE=1 SV=2	IGLV1-51	12,2	0,64	0,159680408
FALSO	High	Master Protein	P62310	U6 snRNA-associated Sm-like protein LSm3 OS=Homo sapiens (Human) OX=9606 GN=LSM3 PE=1 SV=2	LSM3	11,8	0,64	0,023596155
FALSO	High	Master Protein	P21281	V-type proton ATPase subunit B, brain isoform OS=Homo sapiens (Human) OX=9606 GN=ATP6V1B2 PE=1 SV	ATP6V1B2	56,5	0,64	0,030176336
FALSO	High	None	Q58FF3	Putative endoplasmin-like protein OS=Homo sapiens (Human) OX=9606 GN=HSP90B2P PE=5 SV=1	HSP90B2P	45,8	0,64	0,176314576
FALSO	High	Master Protein	P01591	Immunoglobulin J chain OS=Homo sapiens (Human) OX=9606 GN=JCHAIN PE=1 SV=4	IGJ; JCHAIN	18,1	0,64	0,010536595
FALSO	Medium	None	O95741	Copine-6 OS=Homo sapiens (Human) OX=9606 GN=CPNE6 PE=1 SV=3	CPNE6	62	0,63	0,536846134
FALSO	Medium	None	Q86YQ8	Copine-8 OS=Homo sapiens (Human) OX=9606 GN=CPNE8 PE=1 SV=2	CPNE8	63,1	0,63	0,536846134
FALSO	Medium	None	Q9UBL6	Copine-7 OS=Homo sapiens (Human) OX=9606 GN=CPNE7 PE=1 SV=1	CPNE7	70,2	0,63	0,536846134
FALSO	Medium	None	Q8IYJ1	Copine-9 OS=Homo sapiens (Human) OX=9606 GN=CPNE9 PE=1 SV=3	CPNE9	61,8	0,63	0,536846134
FALSO	Medium	None	Q9HCH3	Copine-5 OS=Homo sapiens (Human) OX=9606 GN=CPNE5 PE=1 SV=2	CPNE5	65,7	0,63	0,536846134
FALSO	Medium	None	Q96FN4	Copine-2 OS=Homo sapiens (Human) OX=9606 GN=CPNE2 PE=1 SV=3	CPNE2	61,2	0,63	0,536846134
FALSO	Medium	None	Q96A23	Copine-4 OS=Homo sapiens (Human) OX=9606 GN=CPNE4 PE=1 SV=1	CPNE4	62,4	0,63	0,536846134
FALSO	High	Master Protein	Q9NZP8	Complement C1r subcomponent-like protein OS=Homo sapiens (Human) OX=9606 GN=C1RL PE=1 SV=2	C1RL	53,5	0,63	0,393472277
FALSO	High	Master Protein	Q06830	Peroxiredoxin-1 OS=Homo sapiens (Human) OX=9606 GN=PRDX1 PE=1 SV=1	PRDX1	22,1	0,63	0,028291521
FALSO	High	Master Protein	P55145	Mesencephalic astrocyte-derived neurotrophic factor OS=Homo sapiens (Human) OX=9606 GN=MANF PE=1 S	MANF	20,7	0,63	0,307758668
FALSO	High	Master Protein	Q12906	Interleukin enhancer-binding factor 3 OS=Homo sapiens (Human) OX=9606 GN=ILF3 PE=1 SV=3	ILF3	95,3	0,63	0,141291734
FALSO	High	Master Protein	P02766	Transthyretin OS=Homo sapiens (Human) OX=9606 GN=TTR PE=1 SV=1	TTR	15,9	0,63	0,025848852
FALSO	High	None	Q13162	Peroxiredoxin-4 OS=Homo sapiens (Human) OX=9606 GN=PRDX4 PE=1 SV=1	PRDX4	30,5	0,63	0,10880674
FALSO	High	Master Protein	Q8NBS9	Thioredoxin domain-containing protein 5 OS=Homo sapiens (Human) OX=9606 GN=TXNDC5 PE=1 SV=2	TXNDC5	47,6	0,63	0,087077836
FALSO	High	Master Protein	P12081	Histidine--tRNA ligase, cytoplasmic OS=Homo sapiens (Human) OX=9606 GN=HARS1 PE=1 SV=2	HARS	57,4	0,63	0,023851924
FALSO	High	Master Protein	Q8N6Q3	CD177 antigen OS=Homo sapiens (Human) OX=9606 GN=CD177 PE=1 SV=2	CD177	46,3	0,63	0,239753242
FALSO	High	Master Protein	Q2TAA2	Isoamyl acetate-hydrolyzing esterase 1 homolog OS=Homo sapiens (Human) OX=9606 GN=IAH1 PE=1 SV=1	IAH1	27,6	0,63	0,171466505
FALSO	High	Master Protein	Q02750	Dual specificity mitogen-activated protein kinase kinase 1 OS=Homo sapiens (Human) OX=9606 GN=MAP2K1	MAP2K1	43,4	0,63	0,226911808
FALSO	High	Master Protein	P30613	Pyruvate kinase PKLR OS=Homo sapiens (Human) OX=9606 GN=PKLR PE=1 SV=2	PKLR	61,8	0,63	0,293376885
FALSO	High	Master Protein	P07476	Involucrin OS=Homo sapiens (Human) OX=9606 GN=IVL PE=1 SV=2	IVL	68,4	0,63	0,497760839
FALSO	High	Master Protein	Q08188	Protein-glutamine gamma-glutamyltransferase E OS=Homo sapiens (Human) OX=9606 GN=TGM3 PE=1 SV=4	TGM3	76,6	0,63	0,017394317
FALSO	High	Master Protein	P05543	Thyroxine-binding globulin OS=Homo sapiens (Human) OX=9606 GN=SERPINA7 PE=1 SV=2	SERPINA7	46,3	0,63	0,18345648
FALSO	Medium	Master Protein	Q8NBN3	Transmembrane protein 87A OS=Homo sapiens (Human) OX=9606 GN=TMEM87A PE=1 SV=3	TMEM87A	63,4	0,63	0,067546838

FALSO	High	Master Protein	Q9HD89	Resistin OS=Homo sapiens (Human) OX=9606 GN=RETN PE=1 SV=1	RETN	11,4	0,62	0,08708652
FALSO	High	Master Protein	P11766	Alcohol dehydrogenase class-3 OS=Homo sapiens (Human) OX=9606 GN=ADH5 PE=1 SV=4	ADH5	39,7	0,62	0,123177132
FALSO	High	Master Protein	P51159	Ras-related protein Rab-27A OS=Homo sapiens (Human) OX=9606 GN=RAB27A PE=1 SV=3	RAB27A	24,9	0,62	0,200071401
FALSO	High	Master Protein	P60983	Glia maturation factor beta OS=Homo sapiens (Human) OX=9606 GN=GMFB PE=1 SV=2	GMFB	16,7	0,62	0,070086588
FALSO	High	Master Protein	P49591	Serine--tRNA ligase, cytoplasmic OS=Homo sapiens (Human) OX=9606 GN=SARS1 PE=1 SV=3	SARS	58,7	0,62	0,02102007
FALSO	High	Master Protein	P01009	Alpha-1-antitrypsin OS=Homo sapiens (Human) OX=9606 GN=SERPINA1 PE=1 SV=3	SERPINA1	46,7	0,62	0,008628278
FALSO	High	Master Protein	P09012	U1 small nuclear ribonucleoprotein A OS=Homo sapiens (Human) OX=9606 GN=SNRPA PE=1 SV=3	SNRPA	31,3	0,62	0,164852526
FALSO	High	Master Protein	P80217	Interferon-induced 35 kDa protein OS=Homo sapiens (Human) OX=9606 GN=IFI35 PE=1 SV=5	IFI35	31,5	0,62	0,223746413
FALSO	High	Master Protein	P14317	Hematopoietic lineage cell-specific protein OS=Homo sapiens (Human) OX=9606 GN=HCLS1 PE=1 SV=3	HCLS1	54	0,62	0,337553796
FALSO	High	Master Protein	Q9NZH8	Interleukin-36 gamma OS=Homo sapiens (Human) OX=9606 GN=IL36G PE=1 SV=1	IL36G	18,7	0,61	0,339621133
FALSO	High	Master Protein	Q12904	Aminoacyl tRNA synthase complex-interacting multifunctional protein 1 OS=Homo sapiens (Human) OX=960	AIMP1	34,3	0,61	0,015164918
FALSO	High	Master Protein	P62857	40S ribosomal protein S28 OS=Homo sapiens (Human) OX=9606 GN=RPS28 PE=1 SV=1	RPS28	7,8	0,61	0,047268103
FALSO	High	Master Protein	P14314	Glucosidase 2 subunit beta OS=Homo sapiens (Human) OX=9606 GN=PRKCSH PE=1 SV=2	PRKCSH	59,4	0,61	0,051889679
FALSO	High	Master Protein	Q16658	Fascin OS=Homo sapiens (Human) OX=9606 GN=FSCN1 PE=1 SV=3	FSCN1	54,5	0,61	0,051436009
FALSO	High	Master Protein	Q13561	Dynactin subunit 2 OS=Homo sapiens (Human) OX=9606 GN=DCTN2 PE=1 SV=4	DCTN2	44,2	0,61	0,185818322
FALSO	High	Master Protein	Q9BQE3	Tubulin alpha-1C chain OS=Homo sapiens (Human) OX=9606 GN=TUBA1C PE=1 SV=1	TUBA1C	49,9	0,61	0,033723604
FALSO	High	Master Protein	P22079	Lactoperoxidase OS=Homo sapiens (Human) OX=9606 GN=LPO PE=1 SV=2	LPO	80,2	0,61	0,229427159
FALSO	High	Master Protein	Q16610	Extracellular matrix protein 1 OS=Homo sapiens (Human) OX=9606 GN=ECM1 PE=1 SV=2	ECM1	60,6	0,61	0,025519023
FALSO	High	Master Protein Candidat	P84077	ADP-ribosylation factor 1 OS=Homo sapiens (Human) OX=9606 GN=ARF1 PE=1 SV=2	ARF1	20,7	0,61	0,210775791
FALSO	High	Master Protein	P61204	ADP-ribosylation factor 3 OS=Homo sapiens (Human) OX=9606 GN=ARF3 PE=1 SV=2	ARF3	20,6	0,61	0,210775791
FALSO	High	Master Protein	Q7L591	Docking protein 3 OS=Homo sapiens (Human) OX=9606 GN=DOK3 PE=1 SV=2	DOK3	53,3	0,61	0,019819843
FALSO	High	Master Protein	P80723	Brain acid soluble protein 1 OS=Homo sapiens (Human) OX=9606 GN=BASP1 PE=1 SV=2	BASP1	22,7	0,60	0,118546259
FALSO	High	Master Protein	P35573	Glycogen debranching enzyme OS=Homo sapiens (Human) OX=9606 GN=AGL PE=1 SV=3	AGL	175	0,60	0,154602643
FALSO	High	Master Protein	P13797	Plastin-3 OS=Homo sapiens (Human) OX=9606 GN=PLS3 PE=1 SV=4	PLS3	70,8	0,60	0,06359277
FALSO	High	Master Protein	Q13177	Serine/threonine-protein kinase PAK 2 OS=Homo sapiens (Human) OX=9606 GN=PAK2 PE=1 SV=3	PAK2	58	0,60	0,080103883
FALSO	High	Master Protein	P16050	Arachidonate 15-lipoxygenase OS=Homo sapiens (Human) OX=9606 GN=ALOX15 PE=1 SV=3	ALOX15	74,8	0,60	0,173128508
FALSO	High	None	A0A075B7E8	Ig-like domain-containing protein OS=Homo sapiens (Human) OX=9606 GN=IGHV3OR16-13 PE=1 SV=1	IGHV3OR16-13	12,9	0,60	0,003881138
FALSO	High	Master Protein	Q9Y2B0	Protein canopy homolog 2 OS=Homo sapiens (Human) OX=9606 GN=CNPY2 PE=1 SV=1	CNPY2	20,6	0,60	0,015267016
FALSO	High	Master Protein	O43707	Alpha-actinin-4 OS=Homo sapiens (Human) OX=9606 GN=ACTN4 PE=1 SV=2	ACTN4	105	0,60	0,223676918
FALSO	High	Master Protein	O60925	Prefoldin subunit 1 OS=Homo sapiens (Human) OX=9606 GN=PFDN1 PE=1 SV=2	PFDN1	14,2	0,60	0,013460757
FALSO	High	Master Protein	P15848	Arylsulfatase B OS=Homo sapiens (Human) OX=9606 GN=ARSB PE=1 SV=1	ARSB	59,6	0,60	0,061171225
FALSO	High	Master Protein	P12883	Myosin-7 OS=Homo sapiens (Human) OX=9606 GN=MYH7 PE=1 SV=5	MYH7	223	0,60	0,05642175
FALSO	High	Master Protein	P13987	CD59 glycoprotein OS=Homo sapiens (Human) OX=9606 GN=CD59 PE=1 SV=1	CD59	14,2	0,60	0,230625935

FALSO	High	Master Protein	O75594	Peptidoglycan recognition protein 1 OS=Homo sapiens (Human) OX=9606 GN=PGLYRP1 PE=1 SV=1	PGLYRP1	21,7	0,59	0,014926337
FALSO	High	None	Q02325	Plasminogen-like protein B OS=Homo sapiens (Human) OX=9606 GN=PLGLB2 PE=3 SV=1	PLGLB2	11	0,59	0,237546961
FALSO	High	Master Protein	P49908	Selenoprotein P OS=Homo sapiens (Human) OX=9606 GN=SELENOP PE=1 SV=3	SELENOP	43,2	0,59	0,056881599
FALSO	High	Master Protein	Q96CX2	BTB/POZ domain-containing protein KCTD12 OS=Homo sapiens (Human) OX=9606 GN=KCTD12 PE=1 SV=1	KCTD12	35,7	0,59	0,102557089
FALSO	High	Master Protein	P07910	Heterogeneous nuclear ribonucleoproteins C1/C2 OS=Homo sapiens (Human) OX=9606 GN=HNRNPC PE=1 S	HNRNPC	33,7	0,59	0,016912679
FALSO	High	Master Protein	P09417	Dihydropteridine reductase OS=Homo sapiens (Human) OX=9606 GN=QDPR PE=1 SV=2	QDPR	25,8	0,59	0,003493727
FALSO	Medium	Master Protein	P16989	Y-box-binding protein 3 OS=Homo sapiens (Human) OX=9606 GN=YBX3 PE=1 SV=4	CSDA; YBX3	40,1	0,59	0,241325819
FALSO	High	Master Protein	P0CF74	Immunoglobulin lambda constant 6 OS=Homo sapiens (Human) OX=9606 GN=IGLC6 PE=1 SV=1	IGLC6	11,3	0,58	0,144105421
FALSO	High	None	O75914	Serine/threonine-protein kinase PAK 3 OS=Homo sapiens (Human) OX=9606 GN=PAK3 PE=1 SV=2	PAK3	62,3	0,58	#¡DIV/0!
FALSO	High	Master Protein	P15104	Glutamine synthetase OS=Homo sapiens (Human) OX=9606 GN=GLUL PE=1 SV=4	GLUL	42	0,58	0,139093627
FALSO	High	Master Protein	Q9NYL9	Tropomodulin-3 OS=Homo sapiens (Human) OX=9606 GN=TMOD3 PE=1 SV=1	TMOD3	39,6	0,58	0,021069301
FALSO	High	Master Protein	Q9BT09	Protein canopy homolog 3 OS=Homo sapiens (Human) OX=9606 GN=CNPY3 PE=1 SV=1	CNPY3	30,7	0,58	0,053620677
FALSO	High	Master Protein	P35326	Small proline-rich protein 2A OS=Homo sapiens (Human) OX=9606 GN=SPRR2A PE=1 SV=1	SPRR2A	8	0,58	0,018346564
FALSO	High	Master Protein	P06576	ATP synthase subunit beta, mitochondrial OS=Homo sapiens (Human) OX=9606 GN=ATP5F1B PE=1 SV=3	ATP5B	56,5	0,57	0,012803038
FALSO	High	Master Protein	P16070	CD44 antigen OS=Homo sapiens (Human) OX=9606 GN=CD44 PE=1 SV=3	CD44	81,5	0,57	0,00064468
FALSO	High	None	O60812	Heterogeneous nuclear ribonucleoprotein C-like 1 OS=Homo sapiens (Human) OX=9606 GN=HNRNPCL1 PE=1	HNRNPCL1	32,1	0,57	0,014405553
FALSO	High	None	A0A0G2JNQ3	RRM domain-containing protein OS=Homo sapiens (Human) OX=9606 GN=HNRNPCL2 PE=4 SV=1	HNRNPCL2	32	0,57	0,014405553
FALSO	High	None	B2RXH8	Heterogeneous nuclear ribonucleoprotein C-like 2 OS=Homo sapiens (Human) OX=9606 GN=HNRNPCL2 PE=1	HNRNPCP5	32,1	0,57	0,014405553
FALSO	High	Master Protein	P02545	Prelamin-A/C OS=Homo sapiens (Human) OX=9606 GN=LMNA PE=1 SV=1	LMNA	74,1	0,57	0,143453244
FALSO	High	Master Protein	P61106	Ras-related protein Rab-14 OS=Homo sapiens (Human) OX=9606 GN=RAB14 PE=1 SV=4	RAB14	23,9	0,57	0,051848711
FALSO	High	Master Protein	O60234	Glia maturation factor gamma OS=Homo sapiens (Human) OX=9606 GN=GMFG PE=1 SV=1	GMFG	16,8	0,57	0,028275011
FALSO	High	Master Protein	Q07654	Trefoil factor 3 OS=Homo sapiens (Human) OX=9606 GN=TFF3 PE=1 SV=2	TFF3	10,2	0,57	0,21212479
FALSO	High	Master Protein	P36952	Serpin B5 OS=Homo sapiens (Human) OX=9606 GN=SERPINB5 PE=1 SV=2	SERPINB5	42,1	0,57	0,009008696
FALSO	High	Master Protein	P30050	60S ribosomal protein L12 OS=Homo sapiens (Human) OX=9606 GN=RPL12 PE=1 SV=1	RPL12	17,8	0,57	0,024657711
FALSO	High	Master Protein	Q02790	Peptidyl-prolyl cis-trans isomerase FKBP4 OS=Homo sapiens (Human) OX=9606 GN=FKBP4 PE=1 SV=3	FKBP4	51,8	0,57	0,228562185
FALSO	High	Master Protein	O75347	Tubulin-specific chaperone A OS=Homo sapiens (Human) OX=9606 GN=TBCA PE=1 SV=3	TBCA	12,8	0,57	0,005722222
FALSO	High	None	Q4VXU2	Polyadenylate-binding protein 1-like OS=Homo sapiens (Human) OX=9606 GN=PABPC1L PE=2 SV=1	PABPC1L	68,3	0,57	0,043423732
FALSO	High	Master Protein	Q14520	Hyaluronan-binding protein 2 OS=Homo sapiens (Human) OX=9606 GN=HABP2 PE=1 SV=1	HABP2	62,6	0,57	0,02621079
FALSO	High	Master Protein	P12724	Eosinophil cationic protein OS=Homo sapiens (Human) OX=9606 GN=RNASE3 PE=1 SV=2	RNASE3	18,4	0,57	0,062903742
FALSO	Medium	Master Protein	Q9BPY8	Homeodomain-only protein OS=Homo sapiens (Human) OX=9606 GN=HOPX PE=1 SV=1	HOPX	8,3	0,56	#¡DIV/0!
FALSO	High	Master Protein	Q96RM1	Small proline-rich protein 2F OS=Homo sapiens (Human) OX=9606 GN=SPRR2F PE=3 SV=1	SPRR2F	7,8	0,56	0,046584902
FALSO	High	Master Protein	P26885	Peptidyl-prolyl cis-trans isomerase FKBP2 OS=Homo sapiens (Human) OX=9606 GN=FKBP2 PE=1 SV=2	FKBP2	15,6	0,56	0,015765451
FALSO	High	None	P13533	Myosin-6 OS=Homo sapiens (Human) OX=9606 GN=MYH6 PE=1 SV=5	MYH6	224	0,56	0,023986748

FALSO	High	Master Protein	P02655	Apolipoprotein C-II OS=Homo sapiens (Human) OX=9606 GN=APOC2 PE=1 SV=1	APOC2	11,3	0,56	0,319844597
FALSO	High	Master Protein	Q07021	Complement component 1 Q subcomponent-binding protein, mitochondrial OS=Homo sapiens (Human) OX=	C1QBP	31,3	0,56	0,021199047
FALSO	Medium	Master Protein	Q8WUW1	Protein BRICK1 OS=Homo sapiens (Human) OX=9606 GN=BRK1 PE=1 SV=1	BRK1	8,7	0,56	0,195375975
FALSO	High	Master Protein	P16949	Stathmin OS=Homo sapiens (Human) OX=9606 GN=STMN1 PE=1 SV=3	STMN1	17,3	0,56	0,028556235
FALSO	High	Master Protein	Q14005	Pro-interleukin-16 OS=Homo sapiens (Human) OX=9606 GN=IL16 PE=1 SV=4	IL16	142	0,56	0,027389804
FALSO	High	None	Q9H361	Polyadenylate-binding protein 3 OS=Homo sapiens (Human) OX=9606 GN=PABPC3 PE=1 SV=2	PABPC3	70	0,56	0,005292421
FALSO	High	Master Protein	P27816	Microtubule-associated protein 4 OS=Homo sapiens (Human) OX=9606 GN=MAP4 PE=1 SV=3	MAP4	121	0,56	0,221742958
FALSO	High	Master Protein	Q08257	Quinone oxidoreductase OS=Homo sapiens (Human) OX=9606 GN=CRYZ PE=1 SV=1	CRYZ	35,2	0,55	0,002844666
FALSO	High	None	B2RPK0	Putative high mobility group protein B1-like 1 OS=Homo sapiens (Human) OX=9606 GN=HMGB1P1 PE=5 SV=	CTCFL	24,2	0,55	0,055371377
FALSO	High	Master Protein	P01704	Immunoglobulin lambda variable 2-14 OS=Homo sapiens (Human) OX=9606 GN=IGLV2-14 PE=1 SV=2	IGLV2-14	12,6	0,55	0,120639688
FALSO	High	Master Protein	Q6P5S2	Protein LEG1 homolog OS=Homo sapiens (Human) OX=9606 GN=LEG1 PE=1 SV=2	C6orf58	37,9	0,55	0,128098336
FALSO	High	Master Protein	O43278	Kunitz-type protease inhibitor 1 OS=Homo sapiens (Human) OX=9606 GN=SPINT1 PE=1 SV=2	SPINT1	58,4	0,55	0,038628939
FALSO	High	Master Protein	Q9UBG0	C-type mannose receptor 2 OS=Homo sapiens (Human) OX=9606 GN=MRC2 PE=1 SV=2	MRC2	167	0,55	0,181106402
FALSO	High	None	A0A0G2JPF8	RRM domain-containing protein OS=Homo sapiens (Human) OX=9606 GN=HNRNPCL4 PE=4 SV=1	HNRNPCL4	32	0,55	0,010496732
FALSO	High	None	B7ZW38	Heterogeneous nuclear ribonucleoprotein C-like 3 OS=Homo sapiens (Human) OX=9606 GN=HNRNPCL3 PE=2	HNRNPCL3	32	0,55	0,010496732
FALSO	High	Master Protein	A0A075B6S9	Probable non-functional immunoglobulinn kappa variable 1-37 OS=Homo sapiens (Human) OX=9606 GN=IG	IGKV1-37	12,7	0,55	0,070090342
FALSO	High	Master Protein	P51858	Hepatoma-derived growth factor OS=Homo sapiens (Human) OX=9606 GN=HDGF PE=1 SV=1	HDGF	26,8	0,55	0,001727525
FALSO	Medium	Master Protein	P43243	Matrin-3 OS=Homo sapiens (Human) OX=9606 GN=MATR3 PE=1 SV=2	MATR3	94,6	0,55	0,0364788
FALSO	High	Master Protein	P09429	High mobility group protein B1 OS=Homo sapiens (Human) OX=9606 GN=HMGB1 PE=1 SV=3	HMGB1	24,9	0,55	0,04541211
FALSO	High	Master Protein	Q9UM07	Protein-arginine deiminase type-4 OS=Homo sapiens (Human) OX=9606 GN=PADI4 PE=1 SV=2	PADI4	74	0,55	0,010892753
FALSO	High	Master Protein	P15531	Nucleoside diphosphate kinase A OS=Homo sapiens (Human) OX=9606 GN=NME1 PE=1 SV=1	NME1	17,1	0,55	0,15197597
FALSO	High	Master Protein	P26583	High mobility group protein B2 OS=Homo sapiens (Human) OX=9606 GN=HMGB2 PE=1 SV=2	HMGB2	24	0,54	0,072965841
FALSO	Medium	Master Protein	Q9BUQ8	Probable ATP-dependent RNA helicase DDX23 OS=Homo sapiens (Human) OX=9606 GN=DDX23 PE=1 SV=3	DDX23	95,5	0,54	0,098980439
FALSO	High	Master Protein	P22532	Small proline-rich protein 2D OS=Homo sapiens (Human) OX=9606 GN=SPRR2D PE=2 SV=2	SPRR2D	7,9	0,54	0,059076258
FALSO	High	Master Protein	P33176	Kinesin-1 heavy chain OS=Homo sapiens (Human) OX=9606 GN=KIF5B PE=1 SV=1	KIF5B	110	0,54	0,073625181
FALSO	High	Master Protein	O60361	Putative nucleoside diphosphate kinase OS=Homo sapiens (Human) OX=9606 GN=NME2P1 PE=5 SV=1	NME2P1	15,5	0,54	0,337468843
FALSO	High	Master Protein	P15090	Fatty acid-binding protein, adipocyte OS=Homo sapiens (Human) OX=9606 GN=FABP4 PE=1 SV=3	FABP4	14,7	0,54	0,126338876
FALSO	Medium	Master Protein	Q8NE18	Putative methyltransferase NSUN7 OS=Homo sapiens (Human) OX=9606 GN=NSUN7 PE=2 SV=4	NSUN7	81	0,54	0,187112269
FALSO	High	Master Protein	P15311	Ezrin OS=Homo sapiens (Human) OX=9606 GN=EZR PE=1 SV=4	EZR	69,4	0,54	0,005571731
FALSO	High	Master Protein	P12277	Creatine kinase B-type OS=Homo sapiens (Human) OX=9606 GN=CKB PE=1 SV=1	CKB	42,6	0,54	0,126679031
FALSO	High	Master Protein	P27918	Properdin OS=Homo sapiens (Human) OX=9606 GN=CFP PE=1 SV=2	CFP	51,2	0,54	0,031979605
FALSO	Medium	Master Protein Candidat	Q8N6C8	Leukocyte immunoglobulin-like receptor subfamily A member 3 OS=Homo sapiens (Human) OX=9606 GN=LIL	LILRA3	47,4	0,54	0,006935669
FALSO	Medium	Master Protein Candidat	Q8NHL6	Leukocyte immunoglobulin-like receptor subfamily B member 1 OS=Homo sapiens (Human) OX=9606 GN=LIL	LILRB1	70,8	0,54	0,006935669

FALSO	Medium	Master Protein	O75019	Leukocyte immunoglobulin-like receptor subfamily A member 1 OS=Homo sapiens (Human) OX=9606 GN=LIL	LILRA1	53,2	0,54	0,006935669
FALSO	High	Master Protein	Q16851	UTP--glucose-1-phosphate uridylyltransferase OS=Homo sapiens (Human) OX=9606 GN=UGP2 PE=1 SV=5	UGP2	56,9	0,54	0,037729142
FALSO	High	Master Protein	P27482	Calmodulin-like protein 3 OS=Homo sapiens (Human) OX=9606 GN=CALML3 PE=1 SV=2	CALML3	16,9	0,54	0,012754397
FALSO	High	Master Protein	Q8NFN8	Probable G-protein coupled receptor 156 OS=Homo sapiens (Human) OX=9606 GN=GPR156 PE=2 SV=2	GPR156	89	0,53	0,080616088
FALSO	High	Master Protein	Q86SE5	RNA-binding Raly-like protein OS=Homo sapiens (Human) OX=9606 GN=RALYL PE=1 SV=2	RALYL	32,3	0,53	0,013379694
FALSO	High	None	Q93045	Stathmin-2 OS=Homo sapiens (Human) OX=9606 GN=STMN2 PE=1 SV=3	STMN2	20,8	0,53	0,018949249
FALSO	High	Master Protein	P35611	Alpha-adducin OS=Homo sapiens (Human) OX=9606 GN=ADD1 PE=1 SV=2	ADD1	80,9	0,53	#¡DIV/0!
FALSO	High	None	Q5JQF8	Polyadenylate-binding protein 1-like 2 OS=Homo sapiens (Human) OX=9606 GN=PABPC1L2B PE=2 SV=1	PABPC1L2A	22,8	0,53	0,005165476
FALSO	High	None	Q96DU9	Polyadenylate-binding protein 5 OS=Homo sapiens (Human) OX=9606 GN=PABPC5 PE=2 SV=1	PABPC5	43,3	0,53	0,123877025
FALSO	High	Master Protein	O00160	Unconventional myosin-If OS=Homo sapiens (Human) OX=9606 GN=MYO1F PE=1 SV=3	MYO1F	125	0,53	0,003991724
FALSO	High	Master Protein	P14866	Heterogeneous nuclear ribonucleoprotein L OS=Homo sapiens (Human) OX=9606 GN=HNRNPL PE=1 SV=2	HNRNPL	64,1	0,52	0,002004088
FALSO	Medium	Master Protein	A0A075B7D8	Ig-like domain-containing protein OS=Homo sapiens (Human) OX=9606 GN=IGHV3OR15-7 PE=1 SV=1	IGHV3OR15	13,1	0,52	0,053381027
FALSO	High	Master Protein	Q9UHA7	Interleukin-36 alpha OS=Homo sapiens (Human) OX=9606 GN=IL36A PE=1 SV=1	IL36A	17,7	0,52	0,031790502
FALSO	High	Master Protein	A0A0B4J1V0	Immunoglobulin heavy variable 3-15 OS=Homo sapiens (Human) OX=9606 GN=IGHV3-15 PE=3 SV=1	IGHV3-15	12,9	0,52	0,020895848
FALSO	High	Master Protein	P62424	60S ribosomal protein L7a OS=Homo sapiens (Human) OX=9606 GN=RPL7A PE=1 SV=2	RPL7A	30	0,52	0,032916607
FALSO	High	Master Protein	Q15008	26S proteasome non-ATPase regulatory subunit 6 OS=Homo sapiens (Human) OX=9606 GN=PSMD6 PE=1 SV	PSMD6	45,5	0,52	0,027715634
FALSO	Medium	Master Protein	Q8TE73	Dynein heavy chain 5, axonemal OS=Homo sapiens (Human) OX=9606 GN=DNAH5 PE=1 SV=3	DNAH5	529	0,52	0,160257234
FALSO	High	Master Protein	P30740	Leukocyte elastase inhibitor OS=Homo sapiens (Human) OX=9606 GN=SERPINB1 PE=1 SV=1	SERPINB1	42,7	0,52	0,02134709
FALSO	High	Master Protein	P04259	Keratin, type II cytoskeletal 6B OS=Homo sapiens (Human) OX=9606 GN=KRT6B PE=1 SV=5	KRT6B	60	0,52	0,088276983
FALSO	High	Master Protein	P68104	Elongation factor 1-alpha 1 OS=Homo sapiens (Human) OX=9606 GN=EEF1A1 PE=1 SV=1	EEF1A1	50,1	0,52	0,013371358
FALSO	High	Master Protein Candidat	Q5VTE0	Putative elongation factor 1-alpha-like 3 OS=Homo sapiens (Human) OX=9606 GN=EEF1A1P5 PE=5 SV=1	EEF1A1P5	50,2	0,52	0,013371358
FALSO	High	Master Protein	P54108	Cysteine-rich secretory protein 3 OS=Homo sapiens (Human) OX=9606 GN=CRISP3 PE=1 SV=1	CRISP3	27,6	0,51	0,021700015
FALSO	High	None	Q3ZCM7	Tubulin beta-8 chain OS=Homo sapiens (Human) OX=9606 GN=TUBB8 PE=1 SV=2	TUBB8	49,7	0,51	0,167553091
FALSO	High	Master Protein	Q96KP4	Cytosolic non-specific dipeptidase OS=Homo sapiens (Human) OX=9606 GN=CNDP2 PE=1 SV=2	CNDP2	52,8	0,51	0,017797215
FALSO	High	Master Protein	P53396	ATP-citrate synthase OS=Homo sapiens (Human) OX=9606 GN=ACLY PE=1 SV=3	ACLY	121	0,51	0,06325179
FALSO	High	Master Protein	Q9NQW7	Xaa-Pro aminopeptidase 1 OS=Homo sapiens (Human) OX=9606 GN=XPNPEP1 PE=1 SV=3	XPNPEP1	69,9	0,51	0,175752894
FALSO	High	Master Protein	P02812	Basic salivary proline-rich protein 2 OS=Homo sapiens (Human) OX=9606 GN=PRB2 PE=1 SV=3	PRB2	40,8	0,51	0,524406732
FALSO	High	Master Protein	Q9H098	Protein FAM107B OS=Homo sapiens (Human) OX=9606 GN=FAM107B PE=1 SV=1	FAM107B	15,5	0,51	0,119635509
FALSO	High	Master Protein	A8K2U0	Alpha-2-macroglobulin-like protein 1 OS=Homo sapiens (Human) OX=9606 GN=A2ML1 PE=1 SV=3	A2ML1	161	0,50	0,022420866
FALSO	High	Master Protein	Q96C90	Protein phosphatase 1 regulatory subunit 14B OS=Homo sapiens (Human) OX=9606 GN=PPP1R14B PE=1 SV	PPP1R14B	15,9	0,50	0,40097197
FALSO	Medium	None	P02585	Troponin C, skeletal muscle OS=Homo sapiens (Human) OX=9606 GN=TNNC2 PE=1 SV=2	TNNC2	18,1	0,50	0,018124009
FALSO	Medium	Master Protein	P23610	40-kDa huntingtin-associated protein OS=Homo sapiens (Human) OX=9606 GN=F8A1 PE=1 SV=2	F8A2; F8A1; F8A3	39,1	0,50	0,216164539
FALSO	High	None	P14207	Folate receptor beta OS=Homo sapiens (Human) OX=9606 GN=FOLR2 PE=1 SV=4	FOLR2	29,3	0,50	#¡DIV/0!

FALSO	High	None	Q15195	Plasminogen-like protein A OS=Homo sapiens (Human) OX=9606 GN=PLGLA PE=2 SV=1	PLGLA; PLGLB2	10,9	0,50	0,174389362
FALSO	High	Master Protein	P11940	Polyadenylate-binding protein 1 OS=Homo sapiens (Human) OX=9606 GN=PABPC1 PE=1 SV=2	PABPC1	70,6	0,50	0,017615883
FALSO	High	Master Protein	Q8IZ83	Aldehyde dehydrogenase family 16 member A1 OS=Homo sapiens (Human) OX=9606 GN=ALDH16A1 PE=1 SV	ALDH16A1	85,1	0,49	0,154803244
FALSO	High	Master Protein	Q9NUQ9	Protein FAM49B OS=Homo sapiens (Human) OX=9606 GN=FAM49B PE=1 SV=1	FAM49B	36,7	0,49	0,021293704
FALSO	High	Master Protein	P04179	Superoxide dismutase [Mn], mitochondrial OS=Homo sapiens (Human) OX=9606 GN=SOD2 PE=1 SV=3	SOD2	24,7	0,49	0,004567956
FALSO	High	Master Protein	P41439	Folate receptor gamma OS=Homo sapiens (Human) OX=9606 GN=FOLR3 PE=1 SV=2	FOLR3	27,9	0,49	0,395731837
FALSO	High	Master Protein	Q8NCW5	NAD(P)H-hydrate epimerase OS=Homo sapiens (Human) OX=9606 GN=NAXE PE=1 SV=2	NAXE	31,7	0,49	0,055921184
FALSO	High	Master Protein	Q9HC84	Mucin-5B OS=Homo sapiens (Human) OX=9606 GN=MUC5B PE=1 SV=3	MUC5B	596	0,49	0,058271897
FALSO	High	Master Protein	Q9BQR3	Serine protease 27 OS=Homo sapiens (Human) OX=9606 GN=PRSS27 PE=1 SV=1	PRSS27	31,9	0,49	0,027890991
FALSO	High	Master Protein	P30044	Peroxiredoxin-5, mitochondrial OS=Homo sapiens (Human) OX=9606 GN=PRDX5 PE=1 SV=4	PRDX5	22,1	0,48	0,001680342
FALSO	High	Master Protein	Q9BRA2	Thioredoxin domain-containing protein 17 OS=Homo sapiens (Human) OX=9606 GN=TXNDC17 PE=1 SV=1	TXNDC17	13,9	0,48	0,004724822
FALSO	High	None	Q05639	Elongation factor 1-alpha 2 OS=Homo sapiens (Human) OX=9606 GN=EEF1A2 PE=1 SV=1	EEF1A2	50,4	0,48	0,005168086
FALSO	High	Master Protein	P15259	Phosphoglycerate mutase 2 OS=Homo sapiens (Human) OX=9606 GN=PGAM2 PE=1 SV=3	PGAM2	28,7	0,48	0,06216635
FALSO	High	Master Protein	P60981	Destrin OS=Homo sapiens (Human) OX=9606 GN=DSTN PE=1 SV=3	DSTN	18,5	0,48	0,01306944
FALSO	High	Master Protein	Q9Y4E8	Ubiquitin carboxyl-terminal hydrolase 15 OS=Homo sapiens (Human) OX=9606 GN=USP15 PE=1 SV=3	USP15	112	0,48	0,0428333
FALSO	High	Master Protein	P17174	Aspartate aminotransferase, cytoplasmic OS=Homo sapiens (Human) OX=9606 GN=GOT1 PE=1 SV=3	GOT1	46,2	0,48	0,05906446
FALSO	High	Master Protein	P29508	Serpin B3 OS=Homo sapiens (Human) OX=9606 GN=SERPINB3 PE=1 SV=2	SERPINB3	44,5	0,48	0,044611926
FALSO	High	Master Protein	Q14508	WAP four-disulfide core domain protein 2 OS=Homo sapiens (Human) OX=9606 GN=WFDC2 PE=1 SV=2	WFDC2	13	0,47	0,444038093
FALSO	High	Master Protein	Q00688	Peptidyl-prolyl cis-trans isomerase FKBP3 OS=Homo sapiens (Human) OX=9606 GN=FKBP3 PE=1 SV=1	FKBP3	25,2	0,47	0,015942822
FALSO	High	Master Protein	E9PAV3	Nascent polypeptide-associated complex subunit alpha, muscle-specific form OS=Homo sapiens (Human) OX	NACA	205	0,47	0,039320556
FALSO	Medium	Master Protein	Q5THJ4	Vacuolar protein sorting-associated protein 13D OS=Homo sapiens (Human) OX=9606 GN=VPS13D PE=1 SV=	VPS13D	492	0,47	0,064972715
FALSO	High	Master Protein	P63000	Ras-related C3 botulinum toxin substrate 1 OS=Homo sapiens (Human) OX=9606 GN=RAC1 PE=1 SV=1	RAC1	21,4	0,46	0,003238282
FALSO	High	Master Protein	Q7KZF4	Staphylococcal nuclease domain-containing protein 1 OS=Homo sapiens (Human) OX=9606 GN=SND1 PE=1 S	SND1	102	0,46	0,019954333
FALSO	High	Master Protein	A0A0C4DH25	Immunoglobulin kappa variable 3D-20 OS=Homo sapiens (Human) OX=9606 GN=IGKV3D-20 PE=3 SV=1	IGKV3D-20	12,5	0,46	0,092345017
FALSO	High	Master Protein	O60869	Endothelial differentiation-related factor 1 OS=Homo sapiens (Human) OX=9606 GN=EDF1 PE=1 SV=1	EDF1	16,4	0,46	0,029998549
FALSO	High	Master Protein	P46777	60S ribosomal protein L5 OS=Homo sapiens (Human) OX=9606 GN=RPL5 PE=1 SV=3	RPL5	34,3	0,46	0,039521584
FALSO	High	Master Protein	A8MUU1	Putative fatty acid-binding protein 5-like protein 3 OS=Homo sapiens (Human) OX=9606 GN=FABP5P3 PE=5 S	FABP5P3	11,3	0,46	0,21969592
FALSO	High	None	Q02985	Complement factor H-related protein 3 OS=Homo sapiens (Human) OX=9606 GN=CFHR3 PE=1 SV=2	CFHR3	37,3	0,46	0,004405729
FALSO	High	Master Protein	Q6DCA0	AMMECR1-like protein OS=Homo sapiens (Human) OX=9606 GN=AMMECR1L PE=1 SV=1	AMMECR1L	34,5	0,46	0,082647027
FALSO	High	Master Protein	P07741	Adenine phosphoribosyltransferase OS=Homo sapiens (Human) OX=9606 GN=APRT PE=1 SV=2	APRT	19,6	0,46	0,05152291
FALSO	High	None	P98088	Mucin-5AC OS=Homo sapiens (Human) OX=9606 GN=MUC5AC PE=1 SV=4	MUC5AC	585	0,45	0,031193573
FALSO	High	None	P49448	Glutamate dehydrogenase 2, mitochondrial OS=Homo sapiens (Human) OX=9606 GN=GLUD2 PE=1 SV=2	GLUD2	61,4	0,45	0,005408524
FALSO	High	Master Protein	Q9H6Z4	Ran-binding protein 3 OS=Homo sapiens (Human) OX=9606 GN=RANBP3 PE=1 SV=1	RANBP3	60,2	0,45	0,267520004

FALSO	High	Master Protein	Q15109	Advanced glycosylation end product-specific receptor OS=Homo sapiens (Human) OX=9606 GN=AGER PE=1 S	AGER	42,8	0,45	0,000840186
FALSO	Medium	Master Protein Candidat	P42025	Beta-centractin OS=Homo sapiens (Human) OX=9606 GN=ACTR1B PE=1 SV=1	ACTR1B	42,3	0,45	0,001405324
FALSO	Medium	Master Protein	P61163	Alpha-centractin OS=Homo sapiens (Human) OX=9606 GN=ACTR1A PE=1 SV=1	ACTR1A	42,6	0,45	0,001405324
FALSO	High	Master Protein	Q92598	Heat shock protein 105 kDa OS=Homo sapiens (Human) OX=9606 GN=HSPH1 PE=1 SV=1	HSPH1	96,8	0,45	0,004185914
FALSO	High	Master Protein	Q8NHP6	Motile sperm domain-containing protein 2 OS=Homo sapiens (Human) OX=9606 GN=MOSPD2 PE=1 SV=1	MOSPD2	59,7	0,44	0,03633485
FALSO	High	Master Protein	Q9UBF2	Coatomer subunit gamma-2 OS=Homo sapiens (Human) OX=9606 GN=COPG2 PE=1 SV=1	COPG2	97,6	0,44	0,198360895
FALSO	High	Master Protein	P18510	Interleukin-1 receptor antagonist protein OS=Homo sapiens (Human) OX=9606 GN=IL1RN PE=1 SV=1	IL1RN	20	0,44	0,006347305
FALSO	High	Master Protein	O15347	High mobility group protein B3 OS=Homo sapiens (Human) OX=9606 GN=HMGB3 PE=1 SV=4	HMGB3	23	0,44	0,012440154
FALSO	High	None	O95757	Heat shock 70 kDa protein 4L OS=Homo sapiens (Human) OX=9606 GN=HSPA4L PE=1 SV=3	HSPA4L	94,5	0,44	0,044330511
FALSO	Medium	Master Protein	Q92545	Transmembrane protein 131 OS=Homo sapiens (Human) OX=9606 GN=TMEM131 PE=1 SV=3	TMEM131	205	0,44	0,006383976
FALSO	High	Master Protein	P03973	Antileukoproteinase OS=Homo sapiens (Human) OX=9606 GN=SLPI PE=1 SV=2	SLPI	14,3	0,44	0,030193914
FALSO	High	Master Protein	P19957	Elafin OS=Homo sapiens (Human) OX=9606 GN=PI3 PE=1 SV=3	PI3	12,3	0,43	0,131653865
FALSO	High	Master Protein	P07451	Carbonic anhydrase 3 OS=Homo sapiens (Human) OX=9606 GN=CA3 PE=1 SV=3	CA3	29,5	0,43	0,332864133
FALSO	High	Master Protein	P13693	Translationally-controlled tumor protein OS=Homo sapiens (Human) OX=9606 GN=TPT1 PE=1 SV=1	TPT1	19,6	0,43	0,00114996
FALSO	High	Master Protein	Q9NRX4	14 kDa phosphohistidine phosphatase OS=Homo sapiens (Human) OX=9606 GN=PHPT1 PE=1 SV=1	PHPT1	13,8	0,43	0,048726169
FALSO	High	Master Protein	P12830	Cadherin-1 OS=Homo sapiens (Human) OX=9606 GN=CDH1 PE=1 SV=3	CDH1	97,4	0,43	0,010181276
FALSO	High	Master Protein	O95274	Ly6/PLAUR domain-containing protein 3 OS=Homo sapiens (Human) OX=9606 GN=LYPD3 PE=1 SV=2	LYPD3	35,9	0,42	0,004724891
FALSO	High	None	Q13310	Polyadenylate-binding protein 4 OS=Homo sapiens (Human) OX=9606 GN=PABPC4 PE=1 SV=1	PABPC4	70,7	0,42	0,060576399
FALSO	High	Master Protein	P01833	Polymeric immunoglobulin receptor OS=Homo sapiens (Human) OX=9606 GN=PIGR PE=1 SV=4	PIGR	83,2	0,42	0,027363187
FALSO	High	Master Protein	P63220	40S ribosomal protein S21 OS=Homo sapiens (Human) OX=9606 GN=RPS21 PE=1 SV=1	RPS21	9,1	0,42	0,038473081
FALSO	High	Master Protein	P02814	Submaxillary gland androgen-regulated protein 3B OS=Homo sapiens (Human) OX=9606 GN=SMR3B PE=1 SV=	SMR3B	8,2	0,42	0,347195496
FALSO	High	Master Protein	P57737	Coronin-7 OS=Homo sapiens (Human) OX=9606 GN=CORO7 PE=1 SV=2	CORO7	101	0,42	0,132340831
FALSO	High	Master Protein	P12273	Prolactin-inducible protein OS=Homo sapiens (Human) OX=9606 GN=PIP PE=1 SV=1	PIP	16,6	0,41	0,182220379
FALSO	High	Master Protein	P46108	Adapter molecule crk OS=Homo sapiens (Human) OX=9606 GN=CRK PE=1 SV=2	CRK	33,8	0,41	0,049112963
FALSO	High	Master Protein	Q92522	Histone H1x OS=Homo sapiens (Human) OX=9606 GN=H1FX PE=1 SV=1	H1FX	22,5	0,41	0,108623344
FALSO	High	Master Protein	P20810	Calpastatin OS=Homo sapiens (Human) OX=9606 GN=CAST PE=1 SV=4	CAST	76,5	0,41	0,000855011
FALSO	High	Master Protein	P50570	Dynamin-2 OS=Homo sapiens (Human) OX=9606 GN=DNM2 PE=1 SV=2	DNM2	98	0,41	0,26569533
FALSO	High	Master Protein	P68371	Tubulin beta-4B chain OS=Homo sapiens (Human) OX=9606 GN=TUBB4B PE=1 SV=1	TUBB4B	49,8	0,41	0,008912812
FALSO	High	Master Protein	O00244	Copper transport protein ATOX1 OS=Homo sapiens (Human) OX=9606 GN=ATOX1 PE=1 SV=1	ATOX1	7,4	0,41	0,003436153
FALSO	High	Master Protein	P31948	Stress-induced-phosphoprotein 1 OS=Homo sapiens (Human) OX=9606 GN=STIP1 PE=1 SV=1	STIP1	62,6	0,41	0,167526438
FALSO	High	Master Protein	Q16774	Guanylate kinase OS=Homo sapiens (Human) OX=9606 GN=GUK1 PE=1 SV=2	GUK1	21,7	0,40	0,43745584
FALSO	High	Master Protein	P78330	Phosphoserine phosphatase OS=Homo sapiens (Human) OX=9606 GN=PSPH PE=1 SV=2	PSPH	25	0,40	0,00420121
FALSO	High	Master Protein	P48506	Glutamate--cysteine ligase catalytic subunit OS=Homo sapiens (Human) OX=9606 GN=GCLC PE=1 SV=2	GCLC	72,7	0,40	0,019117612

FALSO	High	Master Protein	O43776	Asparagine--tRNA ligase, cytoplasmic OS=Homo sapiens (Human) OX=9606 GN=NARS1 PE=1 SV=1	NARS	62,9	0,40	0,012925271
FALSO	High	Master Protein	P22528	Cornifin-B OS=Homo sapiens (Human) OX=9606 GN=SPRR1B PE=1 SV=2	SPRR1B	9,9	0,40	0,003944475
FALSO	High	Master Protein	P42331	Rho GTPase-activating protein 25 OS=Homo sapiens (Human) OX=9606 GN=ARHGAP25 PE=1 SV=2	ARHGAP25	73,4	0,40	0,007504069
FALSO	High	Master Protein	O00291	Huntingtin-interacting protein 1 OS=Homo sapiens (Human) OX=9606 GN=HIP1 PE=1 SV=5	HIP1	116	0,40	0,083290439
FALSO	High	Master Protein	Q92888	Rho guanine nucleotide exchange factor 1 OS=Homo sapiens (Human) OX=9606 GN=ARHGEF1 PE=1 SV=2	ARHGEF1	102	0,39	0,012196644
FALSO	High	Master Protein	P08865	40S ribosomal protein SA OS=Homo sapiens (Human) OX=9606 GN=RPSA PE=1 SV=4	RPSA	32,8	0,39	0,008466713
FALSO	High	Master Protein	A0A0B4J1V1	Immunoglobulin heavy variable 3-21 OS=Homo sapiens (Human) OX=9606 GN=IGHV3-21 PE=1 SV=1	IGHV3-21	12,8	0,39	#¡DIV/0!
FALSO	Medium	Master Protein	A0A075B6K6	Immunoglobulin lambda variable 4-3 OS=Homo sapiens (Human) OX=9606 GN=IGLV4-3 PE=3 SV=1	IGLV4-3	13,3	0,39	0,004897899
FALSO	High	Master Protein	Q9Y2V2	Calcium-regulated heat-stable protein 1 OS=Homo sapiens (Human) OX=9606 GN=CARHSP1 PE=1 SV=2	CARHSP1	15,9	0,39	0,00717421
FALSO	Medium	None	Q96A05	V-type proton ATPase subunit E 2 OS=Homo sapiens (Human) OX=9606 GN=ATP6V1E2 PE=1 SV=1	ATP6V1E2	26,1	0,39	0,010506206
FALSO	Medium	Master Protein	P01718	Immunoglobulin lambda variable 3-27 OS=Homo sapiens (Human) OX=9606 GN=IGLV3-27 PE=1 SV=2	IGLV3-27	12,2	0,39	0,058012425
FALSO	High	Master Protein	Q8N4F0	BPI fold-containing family B member 2 OS=Homo sapiens (Human) OX=9606 GN=BPIFB2 PE=1 SV=2	BPIFB2	49,1	0,38	0,116284665
FALSO	High	Master Protein	O00468	Agrin OS=Homo sapiens (Human) OX=9606 GN=AGRN PE=1 SV=6	AGRN	217	0,38	0,023752259
FALSO	Medium	Master Protein	P25398	40S ribosomal protein S12 OS=Homo sapiens (Human) OX=9606 GN=RPS12 PE=1 SV=3	RPS12	14,5	0,38	0,000125196
FALSO	High	Master Protein	P18085	ADP-ribosylation factor 4 OS=Homo sapiens (Human) OX=9606 GN=ARF4 PE=1 SV=3	ARF4	20,5	0,38	0,084658855
FALSO	Medium	Master Protein	O00515	Ladinin-1 OS=Homo sapiens (Human) OX=9606 GN=LAD1 PE=1 SV=2	LAD1	57,1	0,38	0,327670573
FALSO	High	Master Protein	P01037	Cystatin-SN OS=Homo sapiens (Human) OX=9606 GN=CST1 PE=1 SV=3	CST1	16,4	0,38	0,262721292
FALSO	High	Master Protein	P10412	Histone H1.4 OS=Homo sapiens (Human) OX=9606 GN=H1-4 PE=1 SV=2	HIST1H1E	21,9	0,37	0,043666083
FALSO	High	Master Protein	P63241	Eukaryotic translation initiation factor 5A-1 OS=Homo sapiens (Human) OX=9606 GN=EIF5A PE=1 SV=2	EIF5A	16,8	0,37	0,002535372
FALSO	High	None	O75828	Carbonyl reductase [NADPH] 3 OS=Homo sapiens (Human) OX=9606 GN=CBR3 PE=1 SV=3	CBR3	30,8	0,37	0,024740609
FALSO	High	Master Protein	P35321	Cornifin-A OS=Homo sapiens (Human) OX=9606 GN=SPRR1A PE=1 SV=2	SPRR1A	9,9	0,37	0,012934408
FALSO	High	Master Protein	P29622	Kallistatin OS=Homo sapiens (Human) OX=9606 GN=SERPINA4 PE=1 SV=3	SERPINA4	48,5	0,37	0,002825271
FALSO	High	Master Protein	P61457	Pterin-4-alpha-carbinolamine dehydratase OS=Homo sapiens (Human) OX=9606 GN=PCBD1 PE=1 SV=2	PCBD1	12	0,37	0,01236648
FALSO	High	None	P16402	Histone H1.3 OS=Homo sapiens (Human) OX=9606 GN=H1-3 PE=1 SV=2	HIST1H1D	22,3	0,37	0,043903922
FALSO	Medium	Master Protein	O95922	Probable tubulin polyglutamylase TTLL1 OS=Homo sapiens (Human) OX=9606 GN=TTLL1 PE=2 SV=1	TTLL1	49	0,36	0,463705434
FALSO	High	Master Protein	Q14CN2	Calcium-activated chloride channel regulator 4 OS=Homo sapiens (Human) OX=9606 GN=CLCA4 PE=1 SV=2	CLCA4	101	0,36	0,029170303
FALSO	High	Master Protein	P06870	Kallikrein-1 OS=Homo sapiens (Human) OX=9606 GN=KLK1 PE=1 SV=2	KLK1	28,9	0,36	0,050215136
FALSO	High	None	A7E2Y1	Myosin-7B OS=Homo sapiens (Human) OX=9606 GN=MYH7B PE=1 SV=4	MYH7B	226	0,36	0,010660758
FALSO	High	Master Protein	Q15582	Transforming growth factor-beta-induced protein ig-h3 OS=Homo sapiens (Human) OX=9606 GN=TGFBI PE=1	TGFBI	74,6	0,36	0,111612069
FALSO	High	Master Protein	Q9UKR3	Kallikrein-13 OS=Homo sapiens (Human) OX=9606 GN=KLK13 PE=2 SV=1	KLK13	30,6	0,36	0,000443388
FALSO	High	Master Protein	P02743	Serum amyloid P-component OS=Homo sapiens (Human) OX=9606 GN=APCS PE=1 SV=2	APCS	25,4	0,35	0,025057628
FALSO	High	Master Protein	P31947	14-3-3 protein sigma OS=Homo sapiens (Human) OX=9606 GN=SFN PE=1 SV=1	SFN	27,8	0,35	0,00120083
FALSO	High	Master Protein	P20962	Parathymosin OS=Homo sapiens (Human) OX=9606 GN=PTMS PE=1 SV=2	PTMS	11,5	0,35	0,033511616

FALSO	High	None	Q9GZV4	Eukaryotic translation initiation factor 5A-2 OS=Homo sapiens (Human) OX=9606 GN=EIF5A2 PE=1 SV=3	EIF5A2	16,8	0,35	0,002441613
FALSO	High	Master Protein	O60437	Periplakin OS=Homo sapiens (Human) OX=9606 GN=PPL PE=1 SV=4	PPL	205	0,35	0,013352749
FALSO	High	None	Q6IS14	Eukaryotic translation initiation factor 5A-1-like OS=Homo sapiens (Human) OX=9606 GN=EIF5AL1 PE=2 SV=2	EIF5AL1	16,8	0,35	0,002338056
FALSO	High	Master Protein	P35030	Trypsin-3 OS=Homo sapiens (Human) OX=9606 GN=PRSS3 PE=1 SV=2	PRSS3	32,5	0,35	0,236497461
FALSO	Medium	Master Protein	Q12765	Secernin-1 OS=Homo sapiens (Human) OX=9606 GN=SCRN1 PE=1 SV=2	SCRN1	46,4	0,34	0,028975032
FALSO	High	None	Q56UQ5	TPT1-like protein OS=Homo sapiens (Human) OX=9606 GN=- PE=2 SV=2	FLJ44635	15,7	0,34	0,00130961
FALSO	High	Master Protein	P10644	cAMP-dependent protein kinase type I-alpha regulatory subunit OS=Homo sapiens (Human) OX=9606 GN=PF	PRKAR1A	43	0,34	0,0025863
FALSO	High	Master Protein	Q02487	Desmocollin-2 OS=Homo sapiens (Human) OX=9606 GN=DSC2 PE=1 SV=1	DSC2	99,9	0,34	0,025486649
FALSO	Medium	Master Protein	Q9BXT4	Tudor domain-containing protein 1 OS=Homo sapiens (Human) OX=9606 GN=TDRD1 PE=1 SV=2	TDRD1	132	0,34	0,061905197
FALSO	High	Master Protein	P16401	Histone H1.5 OS=Homo sapiens (Human) OX=9606 GN=H1-5 PE=1 SV=3	HIST1H1B	22,6	0,34	0,00367062
FALSO	High	Master Protein	P36222	Chitinase-3-like protein 1 OS=Homo sapiens (Human) OX=9606 GN=CHI3L1 PE=1 SV=2	CHI3L1	42,6	0,34	0,003668347
FALSO	High	Master Protein	P08567	Pleckstrin OS=Homo sapiens (Human) OX=9606 GN=PLEK PE=1 SV=3	PLEK	40,1	0,34	0,333793462
FALSO	High	Master Protein	P20231	Tryptase beta-2 OS=Homo sapiens (Human) OX=9606 GN=TPSB2 PE=1 SV=2	TPSAB1; TPSB2	30,5	0,34	0,058741239
FALSO	High	Master Protein	Q92876	Kallikrein-6 OS=Homo sapiens (Human) OX=9606 GN=KLK6 PE=1 SV=1	KLK6	26,8	0,33	0,005068324
FALSO	High	Master Protein	Q9UIV8	Serpin B13 OS=Homo sapiens (Human) OX=9606 GN=SERPINB13 PE=1 SV=2	SERPINB13	44,2	0,33	0,000590907
FALSO	High	None	Q02539	Histone H1.1 OS=Homo sapiens (Human) OX=9606 GN=H1-1 PE=1 SV=3	HIST1H1A	21,8	0,32	0,021581792
FALSO	High	None	P22492	Histone H1t OS=Homo sapiens (Human) OX=9606 GN=HIST1H1T PE=2 SV=4	HIST1H1T	22	0,32	0,021581792
FALSO	High	Master Protein	P04792	Heat shock protein beta-1 OS=Homo sapiens (Human) OX=9606 GN=HSPB1 PE=1 SV=2	HSPB1	22,8	0,32	0,000527119
FALSO	High	Master Protein	P40306	Proteasome subunit beta type-10 OS=Homo sapiens (Human) OX=9606 GN=PSMB10 PE=1 SV=1	PSMB10	28,9	0,32	0,084727213
FALSO	Medium	Master Protein	Q96F85	CB1 cannabinoid receptor-interacting protein 1 OS=Homo sapiens (Human) OX=9606 GN=CNRIP1 PE=1 SV=1	CNRIP1	18,6	0,31	0,008813248
FALSO	High	Master Protein	P07585	Decorin OS=Homo sapiens (Human) OX=9606 GN=DCN PE=1 SV=1	DCN	39,7	0,31	#¡DIV/0!
FALSO	High	Master Protein	P98179	RNA-binding protein 3 OS=Homo sapiens (Human) OX=9606 GN=RBM3 PE=1 SV=1	RBM3	17,2	0,31	0,179513535
FALSO	High	Master Protein	P81605	Dermcidin OS=Homo sapiens (Human) OX=9606 GN=DCD PE=1 SV=2	DCD	11,3	0,31	0,29180805
FALSO	High	Master Protein	P84103	Serine/arginine-rich splicing factor 3 OS=Homo sapiens (Human) OX=9606 GN=SRSF3 PE=1 SV=1	SRSF3	19,3	0,31	0,00299594
FALSO	High	Master Protein	Q9UGM3	Deleted in malignant brain tumors 1 protein OS=Homo sapiens (Human) OX=9606 GN=DMBT1 PE=1 SV=2	DMBT1	261	0,31	0,001052059
FALSO	High	None	Q9ULW8	Protein-arginine deiminase type-3 OS=Homo sapiens (Human) OX=9606 GN=PADI3 PE=1 SV=2	PADI3	74,7	0,31	0,047723909
FALSO	High	Master Protein	P30085	UMP-CMP kinase OS=Homo sapiens (Human) OX=9606 GN=CMPK1 PE=1 SV=3	CMPK1	22,2	0,31	0,002095707
FALSO	High	Master Protein	Q7Z406	Myosin-14 OS=Homo sapiens (Human) OX=9606 GN=MYH14 PE=1 SV=2	MYH14	228	0,30	#¡DIV/0!
FALSO	High	Master Protein	P24534	Elongation factor 1-beta OS=Homo sapiens (Human) OX=9606 GN=EEF1B2 PE=1 SV=3	EEF1B2	24,7	0,30	0,000254527
FALSO	High	Master Protein	A0A075B6K5	Immunoglobulin lambda variable 3-9 OS=Homo sapiens (Human) OX=9606 GN=IGLV3-9 PE=3 SV=1	IGLV3-9	12,3	0,29	0,009781276
FALSO	High	Master Protein	P15924	Desmoplakin OS=Homo sapiens (Human) OX=9606 GN=DSP PE=1 SV=3	DSP	332	0,29	0,012642202
FALSO	High	Master Protein	O15231	Zinc finger protein 185 OS=Homo sapiens (Human) OX=9606 GN=ZNF185 PE=1 SV=3	ZNF185	73,5	0,29	0,003690979
FALSO	High	Master Protein	Q16762	Thiosulfate sulfurtransferase OS=Homo sapiens (Human) OX=9606 GN=TST PE=1 SV=4	TST	33,4	0,28	0,05712567

FALSO	High	Master Protein	P39019	40S ribosomal protein S19 OS=Homo sapiens (Human) OX=9606 GN=RPS19 PE=1 SV=2	RPS19	16,1	0,28	0,053175834
FALSO	Medium	Master Protein	O00232	26S proteasome non-ATPase regulatory subunit 12 OS=Homo sapiens (Human) OX=9606 GN=PSMD12 PE=1	PSMD12	52,9	0,28	0,001513607
FALSO	High	Master Protein	P18065	Insulin-like growth factor-binding protein 2 OS=Homo sapiens (Human) OX=9606 GN=IGFBP2 PE=1 SV=2	IGFBP2	34,8	0,27	0,118564505
FALSO	High	Master Protein	P06681	Complement C2 OS=Homo sapiens (Human) OX=9606 GN=C2 PE=1 SV=2	C2	83,2	0,27	0,18585711
FALSO	High	Master Protein	P00966	Argininosuccinate synthase OS=Homo sapiens (Human) OX=9606 GN=ASS1 PE=1 SV=2	ASS1	46,5	0,26	0,004471859
FALSO	High	Master Protein	P09228	Cystatin-SA OS=Homo sapiens (Human) OX=9606 GN=CST2 PE=1 SV=1	CST2	16,4	0,25	0,263393409
FALSO	High	None	P43353	Aldehyde dehydrogenase family 3 member B1 OS=Homo sapiens (Human) OX=9606 GN=ALDH3B1 PE=1 SV=1	ALDH3B1	51,8	0,25	0,113661737
FALSO	High	None	P51648	Aldehyde dehydrogenase family 3 member A2 OS=Homo sapiens (Human) OX=9606 GN=ALDH3A2 PE=1 SV=1	ALDH3A2	54,8	0,25	0,113661737
FALSO	High	None	P48448	Aldehyde dehydrogenase family 3 member B2 OS=Homo sapiens (Human) OX=9606 GN=ALDH3B2 PE=2 SV=3	ALDH3B2	42,6	0,25	0,113661737
FALSO	High	Master Protein	P04745	Alpha-amylase 1 OS=Homo sapiens (Human) OX=9606 GN=AMY1A PE=1 SV=2	AMY1A	57,7	0,25	0,081885186
FALSO	High	None	P19961	Alpha-amylase 2B OS=Homo sapiens (Human) OX=9606 GN=AMY2B PE=1 SV=1	AMY2B	57,7	0,24	0,073053744
FALSO	Medium	Master Protein	O43242	26S proteasome non-ATPase regulatory subunit 3 OS=Homo sapiens (Human) OX=9606 GN=PSMD3 PE=1 SV	PSMD3	60,9	0,23	0,004559753
FALSO	High	Master Protein	Q9HCY8	Protein S100-A14 OS=Homo sapiens (Human) OX=9606 GN=S100A14 PE=1 SV=1	S100A14	11,7	0,23	0,093954579
FALSO	High	Master Protein	P05114	Non-histone chromosomal protein HMG-14 OS=Homo sapiens (Human) OX=9606 GN=HMGN1 PE=1 SV=3	HMGN1	10,7	0,22	0,010340093
FALSO	High	Master Protein	Q15847	Adipogenesis regulatory factor OS=Homo sapiens (Human) OX=9606 GN=ADIRF PE=1 SV=1	ADIRF	7,9	0,22	0,076489682
FALSO	High	Master Protein	P55000	Secreted Ly-6/uPAR-related protein 1 OS=Homo sapiens (Human) OX=9606 GN=SLURP1 PE=1 SV=2	SLURP1	11,2	0,22	#¡DIV/0!
FALSO	High	None	P02689	Myelin P2 protein OS=Homo sapiens (Human) OX=9606 GN=PMP2 PE=1 SV=3	PMP2	14,9	0,21	0,016985284
FALSO	High	Master Protein	Q14574	Desmocollin-3 OS=Homo sapiens (Human) OX=9606 GN=DSC3 PE=1 SV=3	DSC3	99,9	0,21	0,017557151
FALSO	High	Master Protein	Q9NQ38	Serine protease inhibitor Kazal-type 5 OS=Homo sapiens (Human) OX=9606 GN=SPINK5 PE=1 SV=2	SPINK5	121	0,21	0,048562701
FALSO	High	Master Protein	Q96NY8	Nectin-4 OS=Homo sapiens (Human) OX=9606 GN=NECTIN4 PE=1 SV=1	NECTIN4	55,4	0,20	0,256242125
FALSO	High	Master Protein	O00151	PDZ and LIM domain protein 1 OS=Homo sapiens (Human) OX=9606 GN=PDLIM1 PE=1 SV=4	PDLIM1	36	0,20	0,001091554
FALSO	Medium	Master Protein	A0A075B6K2	Immunoglobulin lambda variable 3-12 OS=Homo sapiens (Human) OX=9606 GN=IGLV3-12 PE=3 SV=2	IGLV3-12	12,4	0,20	#¡DIV/0!
FALSO	High	Master Protein	O95479	GDH/6PGL endoplasmic bifunctional protein OS=Homo sapiens (Human) OX=9606 GN=H6PD PE=1 SV=2	H6PD	88,8	0,20	0,016991447
FALSO	High	Master Protein	P48594	Serpin B4 OS=Homo sapiens (Human) OX=9606 GN=SERPINB4 PE=1 SV=2	SERPINB4	44,8	0,19	0,00302466
FALSO	High	Master Protein	P02810	Salivary acidic proline-rich phosphoprotein 1/2 OS=Homo sapiens (Human) OX=9606 GN=PRH1 PE=1 SV=2	PRH1	17	0,18	0,409207409
FALSO	High	Master Protein	Q5VT79	Annexin A8-like protein 1 OS=Homo sapiens (Human) OX=9606 GN=ANXA8L1 PE=2 SV=2	ANXA8L1	36,9	0,18	0,072265628
FALSO	High	Master Protein	P07305	Histone H1.0 OS=Homo sapiens (Human) OX=9606 GN=H1-0 PE=1 SV=3	H1F0	20,9	0,18	0,000258552
FALSO	High	Master Protein	P01036	Cystatin-S OS=Homo sapiens (Human) OX=9606 GN=CST4 PE=1 SV=3	CST4	16,2	0,18	0,299867684
FALSO	High	Master Protein	P38606	V-type proton ATPase catalytic subunit A OS=Homo sapiens (Human) OX=9606 GN=ATP6V1A PE=1 SV=2	ATP6V1A	68,3	0,16	0,043345482
FALSO	High	Master Protein	Q0VD83	Apolipoprotein B receptor OS=Homo sapiens (Human) OX=9606 GN=APOBR PE=1 SV=3	APOBR	116	0,15	0,068673736
FALSO	High	Master Protein	P68366	Tubulin alpha-4A chain OS=Homo sapiens (Human) OX=9606 GN=TUBA4A PE=1 SV=1	TUBA4A	49,9	0,14	0,247620101
FALSO	High	Master Protein	P31025	Lipocalin-1 OS=Homo sapiens (Human) OX=9606 GN=LCN1 PE=1 SV=1	LCN1	19,2	0,13	0,311026633
FALSO	High	Master Protein Candidat	Q5VSP4	Putative lipocalin 1-like protein 1 OS=Homo sapiens (Human) OX=9606 GN=LCN1P1 PE=5 SV=1	LCN1P1	17,9	0,13	0,311026633

FALSO	Medium	Master Protein	O14737	Programmed cell death protein 5 OS=Homo sapiens (Human) OX=9606 GN=PDCD5 PE=1 SV=3	PDCD5	14,3	0,12	#¡DIV/0!
FALSO	High	Master Protein	Q32MZ4	Leucine-rich repeat flightless-interacting protein 1 OS=Homo sapiens (Human) OX=9606 GN=LRRFIP1 PE=1 S\	LRRFIP1	89,2	0,08	#¡DIV/0!
FALSO	High	Master Protein	Q15185	Prostaglandin E synthase 3 OS=Homo sapiens (Human) OX=9606 GN=PTGES3 PE=1 SV=1	PTGES3	18,7	0,04	0,127720566
FALSO	High	Master Protein	O00187	Mannan-binding lectin serine protease 2 OS=Homo sapiens (Human) OX=9606 GN=MASP2 PE=1 SV=4	MASP2	75,7	Exclusive in NP	3,20536E-16
FALSO	Medium	Master Protein	P49862	Kallikrein-7 OS=Homo sapiens (Human) OX=9606 GN=KLK7 PE=1 SV=1	KLK7	27,5	Exclusive in NP	3,20536E-16
FALSO	High	Master Protein	P02794	Ferritin heavy chain OS=Homo sapiens (Human) OX=9606 GN=FTH1 PE=1 SV=2	FTH1	21,2	Exclusive in NP	3,20536E-16
FALSO	High	Master Protein	P47914	60S ribosomal protein L29 OS=Homo sapiens (Human) OX=9606 GN=RPL29 PE=1 SV=2	RPL29	17,7	Exclusive in NP	3,20536E-16
FALSO	High	Master Protein	P62851	40S ribosomal protein S25 OS=Homo sapiens (Human) OX=9606 GN=RPS25 PE=1 SV=1	RPS25	13,7	Exclusive in NP	3,20536E-16
FALSO	Medium	Master Protein	Q9P2E9	Ribosome-binding protein 1 OS=Homo sapiens (Human) OX=9606 GN=RRBP1 PE=1 SV=5	RRBP1	152	Exclusive in NP	3,20536E-16
FALSO	Medium	Master Protein	Q9UBH0	Interleukin-36 receptor antagonist protein OS=Homo sapiens (Human) OX=9606 GN=IL36RN PE=1 SV=1	IL36RN	17	Exclusive in NP	3,20536E-16
FALSO	Medium	Master Protein	P21291	Cysteine and glycine-rich protein 1 OS=Homo sapiens (Human) OX=9606 GN=CSRP1 PE=1 SV=3	CSRP1	20,6	Exclusive in NP	3,20536E-16
FALSO	Medium	None	Q8WVV9	Heterogeneous nuclear ribonucleoprotein L-like OS=Homo sapiens (Human) OX=9606 GN=HNRNPLL PE=1 SV	HNRNPLL	60	Exclusive in NP	3,20536E-16
FALSO	Medium	Master Protein	P05204	Non-histone chromosomal protein HMG-17 OS=Homo sapiens (Human) OX=9606 GN=HMGN2 PE=1 SV=3	HMGN2	9,4	Exclusive in NP	3,20536E-16
FALSO	High	Master Protein	Q9BY44	Eukaryotic translation initiation factor 2A OS=Homo sapiens (Human) OX=9606 GN=EIF2A PE=1 SV=3	EIF2A	64,9	Exclusive in NP	3,20536E-16
FALSO	Medium	Master Protein	P29274	Adenosine receptor A2a OS=Homo sapiens (Human) OX=9606 GN=ADORA2A PE=1 SV=2	ADORA2A	44,7	Exclusive in NP	3,20536E-16
FALSO	Medium	Master Protein	Q8NC51	Plasminogen activator inhibitor 1 RNA-binding protein OS=Homo sapiens (Human) OX=9606 GN=SERBP1 PE=	SERBP1	44,9	Exclusive in NP	3,20536E-16
FALSO	High	Master Protein	P07998	Ribonuclease pancreatic OS=Homo sapiens (Human) OX=9606 GN=RNASE1 PE=1 SV=4	RNASE1	17,6	Exclusive in NP	#¡DIV/0!
FALSO	High	Master Protein	A0A0C4DH29	Immunoglobulin heavy variable 1-3 OS=Homo sapiens (Human) OX=9606 GN=IGHV1-3 PE=3 SV=1	IGHV1-3	13	Exclusive in NP	#¡DIV/0!
FALSO	High	Master Protein	P41567	Eukaryotic translation initiation factor 1 OS=Homo sapiens (Human) OX=9606 GN=EIF1 PE=1 SV=1	EIF1	12,7	Exclusive in NP	#¡DIV/0!
FALSO	High	Master Protein Candidat	O60739	Eukaryotic translation initiation factor 1b OS=Homo sapiens (Human) OX=9606 GN=EIF1B PE=1 SV=2	EIF1B	12,8	Exclusive in NP	#¡DIV/0!

Supplementary Table 3. Proteins identified in the GCF proteome with DAMP attributions

Origin	Gene Name	Immunogenic action	Receptor	Refrences
Released	Alpha synuclein (SNCA)	SNCA aggregation is related to amyloid plaque and pathological process, including mitochondrial dysfunction, increased oxidative stress, and neuroinflammation. SNCA activate immune cells via TLR2 and TLR4, increasing the expression of TNFα, IL-6, and CXCL1.	TLR2, TLR4	Béraud et al. 2011; Stefanova et al. 2011; Béraud and Maguire-Zeiss 2012; Fellner et al. 2013; Kim et al. 2013
Cytoplasm	Heme (HBD)	Activates innate immune cells via TLR4 y NRLP3 acting as a chemoattractant, inducing cytokine production, ROS generation, and cell death.	TLR4, CD91, CD163	Arruda et al. 2006; Figueiredo et al. 2007; Porto et al. 2007; Dutra et al. 2014; Dutra and Bozza 2014; Soares and Bozza 2016
Nucleus	N-myc and STAT interactor (NMI)	NMI can activate NF-κB signaling pathway of adjacent macrophages through TLR4 and TLR3.	TLR4, TLR3	Imaizumi et al. 2016; Xiahou et al. 2017
Released /Secreted	Apolipoprotein A-I (APOA1)	Induces leukocyte recruitment and expression of proinflammatory cytokines via NF-κB.	TLR4	de Seny et al. 2013; de Seny et al. 2015
Cytoplasm/ nucleus	Heat shock 27 kDa protein (HSPB1)	<u>Anti-inflammatory effects:</u> Induces IL-10, reduces DC differentiation and CD86 expression. Antiapoptotic effect via Caspase3-AKT. It reduces TNF-α but not IL-1β-induced NFκB activation, potentiating LPS-induced NF-κB activation. Inhibit ferroptosis via PKC phosphorylation by reducing iron production by ROS.<br><u>Pro-inflammatory effects:</u> Promotes expression of IL-1β via of NF-κB.	TLR2/4	Laudanski et al. 2007; Salari et al. 2013; Jin et al. 2014; Sun et al. 2015
Released /Secreted	Heat Shock proteins 90kDa (HSP90s)	HSP90s are able to activate the NF-κB pathway release results in the progression of cartilage degeneration and activation of the synovium in osteoarthritis	TLR7/9	Arya et al. 2007; Siebelt et al. 2013; Nefla et al. 2016; Mišunová et al.
Nucleus	Histones (H1.4, H2A1, H2B, H4)	Histones Induce pro-inflammatory innate immune responses via TLR2, TLR4 and TLR9	TLR2, TLR4, TLR9	Huang et al. 2011; Xu et al. 2011; Allam et al. 2012; Rosin and Okusa 2012; Michels et al. 2016
Cytoplasm	Thioredoxins (PRDX1, PRDX2, PRDX3, PRDX5, PRDX6)	Peroxiredoxins are redox-signaling modulators in innate immunity and inflammation. Induces IL-23 via TLR2 and TLR4 .	TLR2 TLR4	Diet et al. 2007; Robinson et al. 2010; Shichita et al. 2012; Knoops et al. 2016
Released /Secreted	High mobility group Box 1 (HMGB1)	Activates NF-κB and MAPK signaling pathway and induces secretion of pro-inflammatory cytokines like TNF-α, IL-1α/β, IL-6 and IL-8. Intracellular oxHMGB1 is an inducer of apoptosis. HMGB1 loses its inflammatory functions when all three cysteines become oxidized.	TLR2/3/4/5/7/9, RAGE, Mac1, IL1-R1 and CXCR4	Yang et al. 2015; Das et al. 2016; Yang et al. 2018; Li et al. 2020

References

Allam R, Scherbaum CR, Darisipudi MN, Mulay SR, Hägele H, Lichtnekert J, Hagemann JH, Rupanagudi KV, Ryu M, Schwarzenberger C, et al. 2012. Histones from Dying Renal Cells Aggravate Kidney Injury via TLR2 and TLR4. Journal of the American Society of Nephrology. 23(8):1375–1388. https://jasn.asnjournals.org/lookup/doi/10.1681/ASN.2011111077.

Arruda MA, Barcellos-de-Souza P, Sampaio ALF, Rossi AG, Graça-Souza A V., Barja-Fidalgo C. 2006. NADPH oxidase-derived ROS: Key modulators of heme-induced mitochondrial stability in human neutrophils. Exp Cell Res. 312(19):3939–3948. https://linkinghub.elsevier.com/retrieve/pii/S0014482706003594.

Arya R, Mallik M, Lakhotia SC. 2007. Heat shock genes — integrating cell survival and death. J Biosci. 32(3):595–610. http://link.springer.com/10.1007/s12038-007-0059-3.

Béraud D, Maguire-Zeiss KA. 2012. Misfolded α-synuclein and toll-like receptors: therapeutic targets for Parkinson's disease. Parkinsonism Relat Disord. 18:S17–S20. https://linkinghub.elsevier.com/retrieve/pii/S1353802011700086.

Béraud D, Twomey M, Bloom B, Mittereder A, Ton V, Neitzke K, Chasovskikh S, Mhyre TR, Maguire-Zeiss KA. 2011. α-Synuclein Alters Toll-Like Receptor Expression. Front Neurosci. 5. http://journal.frontiersin.org/article/10.3389/fnins.2011.00080/abstract.

Das N, Dewan V, Grace PM, Gunn RJ, Tamura R, Tzarum N, Watkins LR, Wilson IA, Yin H. 2016. HMGB1 Activates Proinflammatory Signaling via TLR5 Leading to Allodynia. Cell Rep. 17(4):1128–1140.

Diet A, Abbas K, Bouton C, Guillon B, Tomasello F, Fourquet S, Toledano MB, Drapier J-C. 2007. Regulation of Peroxiredoxins by Nitric Oxide in Immunostimulated Macrophages. Journal of Biological Chemistry. 282(50):36199–36205. https://linkinghub.elsevier.com/retrieve/pii/S002192581846070X.

Dutra FF, Alves LS, Rodrigues D, Fernandez PL, de Oliveira RB, Golenbock DT, Zamboni DS, Bozza MT. 2014. Hemolysis-induced lethality involves inflammasome activation by heme. Proceedings of the National Academy of Sciences. 111(39):E4110–E4118. http://www.pnas.org/lookup/doi/10.1073/pnas.1405023111.

Dutra FF, Bozza MT. 2014. Heme on innate immunity and inflammation. Front Pharmacol. 5. http://journal.frontiersin.org/article/10.3389/fphar.2014.00115/abstract.

Fellner L, Irschick R, Schanda K, Reindl M, Klimaschewski L, Poewe W, Wenning GK, Stefanova N. 2013. Toll-like receptor 4 is required for α-synuclein dependent activation of microglia and astroglia. Glia. 61(3):349–360. https://onlinelibrary.wiley.com/doi/10.1002/glia.22437.

Figueiredo RT, Fernandez PL, Mourao-Sa DS, Porto BN, Dutra FF, Alves LS, Oliveira MF, Oliveira PL, Graça-Souza A V., Bozza MT. 2007. Characterization of Heme as Activator of Toll-like Receptor 4. Journal of Biological Chemistry. 282(28):20221–20229. https://linkinghub.elsevier.com/retrieve/pii/S0021925819780144.

Huang H, Evankovich J, Yan W, Nace G, Zhang L, Ross M, Liao X, Billiar T, Xu J, Esmon CT, et al. 2011. Endogenous histones function as alarmins in sterile inflammatory liver injury through Toll-like receptor 9 in mice. Hepatology. 54(3):999–1008. https://onlinelibrary.wiley.com/doi/10.1002/hep.24501.

Imaizumi T, Yano C, Numata A, Tsugawa K, Hayakari R, Matsumiya T, Yoshida H, Watanabe S, Tsuruga K, Kawaguchi S, et al. 2016. Interferon (IFN)-Induced Protein 35 (IFI35), a Type I Interferon-Dependent Transcript, Upregulates Inflammatory Signaling Pathways by Activating Toll-Like Receptor 3 in Human Mesangial Cells. Kidney Blood Press Res. 41(5):635–642. https://www.karger.com/Article/FullText/447932.

Jin C, Cleveland JC, Ao L, Li J, Zeng Q, Fullerton DA, Meng X. 2014. Human Myocardium Releases Heat Shock Protein 27 (HSP27) after Global Ischemia: The Proinflammatory Effect of Extracellular HSP27 through Toll-like Receptor (TLR)-2 and TLR4. Molecular Medicine. 20(1):280–289. https://molmed.biomedcentral.com/articles/10.2119/molmed.2014.00058.

Kim C, Ho D-H, Suk J-E, You S, Michael S, Kang J, Joong Lee S, Masliah E, Hwang D, Lee H-J, et al. 2013. Neuron-released oligomeric α-synuclein is an endogenous agonist of TLR2 for paracrine activation of microglia. Nat Commun. 4(1):1562. http://www.nature.com/articles/ncomms2534.

Knoops B, Argyropoulou V, Becker S, Ferté L, Kuznetsova O. 2016. Multiple Roles of Peroxiredoxins in Inflammation. Mol Cells. 39(1):60–4. http://www.molcells.org/journal/view.html?doi=10.14348/molcells.2016.2341.

Laudanski K, De A, Miller-Graziano C. 2007. Exogenous heat shock protein 27 uniquely blocks differentiation of monocytes to dendritic cells. Eur J Immunol. 37(10):2812–2824. https://onlinelibrary.wiley.com/doi/10.1002/eji.200636993.

Li W, Deng M, Loughran PA, Yang M, Lin M, Yang C, Gao W, Jin S, Li S, Cai J, et al. 2020. LPS Induces Active HMGB1 Release From Hepatocytes Into Exosomes Through the Coordinated Activities of TLR4 and Caspase-11/GSDMD Signaling. Front Immunol. 11.

Michels A, Albánez S, Mewburn J, Nesbitt K, Gould TJ, Liaw PC, James PD, Swystun LL, Lillicrap D. 2016. Histones link inflammation and thrombosis through the induction of Weibel-Palade body exocytosis. Journal of Thrombosis and Haemostasis. 14(11):2274–2286. https://onlinelibrary.wiley.com/doi/10.1111/jth.13493.

Mišunová M, Svitálková T, Pleštilová L, Kryštufková O, Tegzová D, Svobodová R, Hušáková M, Tomčík M, Bečvář R, Závada J, et al. Molecular markers of systemic autoimmune disorders: the expression of MHC-located HSP70 genes is significantly associated with autoimmunity development. Clin Exp Rheumatol. 35(1):33–42. http://www.ncbi.nlm.nih.gov/pubmed/28032847.

Nefla M, Holzinger D, Berenbaum F, Jacques C. 2016. The danger from within: alarmins in arthritis. Nat Rev Rheumatol. 12(11):669–683. http://www.nature.com/articles/nrrheum.2016.162.

Porto BN, Alves LS, Fernández PL, Dutra TP, Figueiredo RT, Graça-Souza A V., Bozza MT. 2007. Heme Induces Neutrophil Migration and Reactive Oxygen Species Generation through Signaling Pathways Characteristic of Chemotactic Receptors. Journal of Biological Chemistry. 282(33):24430–24436. https://linkinghub.elsevier.com/retrieve/pii/S002192582054427X.

Robinson MW, HUTCHINSON AT, DALTON JP, DONNELLY S. 2010. Peroxiredoxin: a central player in immune modulation. Parasite Immunol. 32(5):305–313. https://onlinelibrary.wiley.com/doi/10.1111/j.1365-3024.2010.01201.x.

Rosin DL, Okusa MD. 2012. Dying Cells and Extracellular Histones in AKI: Beyond a NET Effect? Journal of the American Society of Nephrology. 23(8):1275–1277. https://jasn.asnjournals.org/lookup/doi/10.1681/ASN.2012060615.

Salari S, Seibert T, Chen Y-X, Hu T, Shi C, Zhao X, Cuerrier CM, Raizman JE, O'Brien ER. 2013. Extracellular HSP27 acts as a signaling molecule to activate NF-κB in macrophages. Cell Stress Chaperones. 18(1):53–63. http://link.springer.com/10.1007/s12192-012-0356-0.

de Seny D, Cobraiville G, Charlier E, Neuville S, Esser N, Malaise D, Malaise O, Calvo FQ, Relic B, Malaise MG. 2013. Acute-Phase Serum Amyloid A in Osteoarthritis: Regulatory Mechanism and Proinflammatory Properties. Bobé P, editor. PLoS One. 8(6):e66769. https://dx.plos.org/10.1371/journal.pone.0066769.

de Seny D, Cobraiville G, Charlier E, Neuville S, Lutteri L, Le Goff C, Malaise D, Malaise O, Chapelle J-P, Relic B, et al. 2015. Apolipoprotein-A1 as a Damage-Associated Molecular Patterns Protein in Osteoarthritis: Ex Vivo and In Vitro Pro-Inflammatory Properties. Feng Y, editor. PLoS One. 10(4):e0122904. https://dx.plos.org/10.1371/journal.pone.0122904.

Shichita T, Hasegawa E, Kimura A, Morita R, Sakaguchi R, Takada I, Sekiya T, Ooboshi H, Kitazono T, Yanagawa T, et al. 2012. Peroxiredoxin family proteins are key initiators of post-ischemic inflammation in the brain. Nat Med. 18(6):911–917. http://www.nature.com/articles/nm.2749.

Siebelt M, Jahr H, Groen HC, Sandker M, Waarsing JH, Kops N, Müller C, van Eden W, de Jong M, Weinans H. 2013. Hsp90 Inhibition Protects Against Biomechanically Induced Osteoarthritis in Rats. Arthritis Rheum. 65(8):2102–2112. https://onlinelibrary.wiley.com/doi/10.1002/art.38000.

Soares MP, Bozza MT. 2016. Red alert: labile heme is an alarmin. Curr Opin Immunol. 38:94–100. https://linkinghub.elsevier.com/retrieve/pii/S0952791515001636.

Stefanova N, Fellner L, Reindl M, Masliah E, Poewe W, Wenning GK. 2011. Toll-Like Receptor 4 Promotes α-Synuclein Clearance and Survival of Nigral Dopaminergic Neurons. Am J Pathol. 179(2):954–963. https://linkinghub.elsevier.com/retrieve/pii/S0002944011004172.

Sun X, Ou Z, Xie M, Kang R, Fan Y, Niu X, Wang H, Cao L, Tang D. 2015. HSPB1 as a novel regulator of ferroptotic cancer cell death. Oncogene. 34(45):5617–5625. http://www.nature.com/articles/onc201532.

Xiahou Z, Wang X, Shen J, Zhu X, Xu F, Hu R, Guo D, Li H, Tian Y, Liu Y, et al. 2017. NMI and IFP35 serve as proinflammatory DAMPs during cellular infection and injury. Nat Commun. 8(1):950. http://www.nature.com/articles/s41467-017-00930-9.

Xu J, Zhang X, Monestier M, Esmon NL, Esmon CT. 2011. Extracellular Histones Are Mediators of Death through TLR2 and TLR4 in Mouse Fatal Liver Injury. The Journal of Immunology. 187(5):2626–2631. http://www.jimmunol.org/lookup/doi/10.4049/jimmunol.1003930.

Yang H, Wang H, Chavan SS, Andersson U. 2015. High Mobility Group Box Protein 1 (HMGB1): The Prototypical Endogenous Danger Molecule. Molecular Medicine. 21(S1):S6–S12.

Yang Y, Han C, Guo L, Guan Q. 2018. High expression of the HMGB1-TLR4 axis and its downstream signaling factors in patients with Parkinson's disease and the relationship of pathological staging. Brain Behav. 8(4):e00948. https://onlinelibrary.wiley.com/doi/10.1002/brb3.948.

Supplementary table 4. Cell counts per cluster

	CD4+ T cell	CD8+ T cell	B cell	Plasma B cell	Monocyte/Macrophage	Mast cell	Epithelial	Fibroblast	Myofibroblast	Endothelial	Lymphatic EC	Total
H1	268	156	18	6	111	89	271	847	716	1385	62	3929
H2	1190	721	766	91	311	126	713	570	310	1337	75	6210
PD1	292	176	264	1489	330	13	42	434	160	516	21	3737
PD2	1535	890	186	659	349	76	76	1381	270	1663	84	7169
Total	3285	1943	1234	2245	1101	304	1102	3232	1456	4901	242	21045

Supplementary Table 5-All_markers_21clusters

	p_val	avg_log2FC	pct.1	pct.2	p_val_adj	cluster	gene
**IGLV2-14**	0	-0.312288458637691	471	184	0	0	IGLV2-14
**FAT1**	0	-0.324578438411268	76	437	0	0	FAT1
**PLAU**	0	-0.354509367623892	82	536	0	0	PLAU
**PIK3R3**	0	-0.377842903219457	157	563	0	0	PIK3R3
**TUBA1C**	0	-0.387804970788395	332	708	0	0	TUBA1C
**SFRP1**	0	-0.390793644273782	0.08	495	0	0	SFRP1
**STAB1**	0	-0.393005030923755	328	592	0	0	STAB1
**TNFRSF12A**	0	-0.446013544667282	359	668	0	0	TNFRSF12A
**SLC6A6**	0	-0.535469372014079	267	639	0	0	SLC6A6
**RGS3**	0	-0.579756440893223	286	643	0	0	RGS3
**ABL2**	0	-0.61261943182186	319	664	0	0	ABL2
**SLIT3**	0	-0.621591716509173	85	494	0	0	SLIT3
**COL11A1**	0	-0.631734143507753	136	462	0	0	COL11A1
**CSF2RB**	0	-0.685876314235373	318	614	0	0	CSF2RB
**C2CD4B**	0	-0.78697961877159	0.27	589	0	0	C2CD4B
**MTUS1**	0	-0.822236230146804	145	577	0	0	MTUS1
**ESAM**	0	-0.832064629169561	193	593	0	0	ESAM
**AKAP12**	0	-0.861803211435932	125	673	0	0	AKAP12
**NAMPT**	0	-0.871674277201443	493	809	0	0	NAMPT
**TNFAIP2**	0	-0.875518976506826	386	0.68	0	0	TNFAIP2
**CPXM2**	0	-0.879999360113069	388	688	0	0	CPXM2
**XBP1**	0	-0.884073168148977	307	725	0	0	XBP1
**CYP1B1**	0	-0.897984983253856	236	625	0	0	CYP1B1
**RAB31**	0	-0.90601379226497	409	668	0	0	RAB31
**PDGFRA**	0	-0.917994889291516	314	651	0	0	PDGFRA
**CALU**	0	-0.935146234727214	342	674	0	0	CALU
**GGT5**	0	-0.943391533188198	298	642	0	0	GGT5
**PXDN**	0	-0.966833080464809	472	732	0	0	PXDN
**HSP90B1**	0	-0.979081118599904	0.55	888	0	0	HSP90B1

**DLC1**	0	-0.988129963070696	0.38	677	0	0	DLC1
**LAMA4**	0	-0.994563032253195	148	649	0	0	LAMA4
**VCAM1**	0	-1.01734462178196	379	706	0	0	VCAM1
**WWTR1**	0	-1.05044307445815	0.26	0.65	0	0	WWTR1
**CTSB**	0	-1.05335704467001	0.28	696	0	0	CTSB
**DST**	0	-1.05441079898011	172	598	0	0	DST
**S100A13**	0	-1.0601221758404	166	614	0	0	S100A13
**HSPB1**	0	-1.07539205098021	254	684	0	0	HSPB1
**BMPR2**	0	-1.07936637069216	346	672	0	0	BMPR2
**CRIM1**	0	-1.12620960584544	395	693	0	0	CRIM1
**GRN**	0	-1.13926011922372	269	685	0	0	GRN
**ATF3**	0	-1.14002076821855	497	807	0	0	ATF3
**EMCN**	0	-1.15496245211993	198	586	0	0	EMCN
**RAMP3**	0	-1.16121720837547	0.15	555	0	0	RAMP3
**HSPA1A**	0	-1.18435739657576	386	721	0	0	HSPA1A
**RHOB**	0	-1.19068654815173	392	671	0	0	RHOB
**MAP1B**	0	-1.20364799533926	161	596	0	0	MAP1B
**NFIB**	0	-1.21936035634689	208	672	0	0	NFIB
**MCAM**	0	-1.24533472505853	227	668	0	0	MCAM
**CFH**	0	-1.25911182414477	216	661	0	0	CFH
**SERPINH1**	0	-1.26835931436225	405	768	0	0	SERPINH1
**HTRA1**	0	-1.28191262798085	289	664	0	0	HTRA1
**GLUL**	0	-1.29831511550418	226	0.68	0	0	GLUL
**TSC22D1**	0	-1.38020609986731	357	716	0	0	TSC22D1
**FILIP1L**	0	-1.4416037599606	0.29	0.65	0	0	FILIP1L
**HES1**	0	-1.45548413805426	403	708	0	0	HES1
**SERPING1**	0	-1.46863283439741	366	679	0	0	SERPING1
**LGALS3**	0	-1.48778492349218	361	761	0	0	LGALS3
**KLF4**	0	-1.52867273163306	465	763	0	0	KLF4
**ICAM1**	0	-1.53519429808947	497	0.78	0	0	ICAM1
**SERPINF1**	0	-1.57681753412978	246	0.62	0	0	SERPINF1
**NR2F2**	0	-1.58890625774671	0.2	599	0	0	NR2F2

**FSTL1**	0	-1.60844739637636	447	764	0	0	FSTL1
**FBN1**	0	-1.64446265938497	425	713	0	0	FBN1
**GNG11**	0	-1.64768197577667	244	646	0	0	GNG11
**ENG**	0	-1.70161903367149	305	658	0	0	ENG
**EGR1**	0	-1.71522643613302	554	839	0	0	EGR1
**PDLIM1**	0	-1.72236204799044	294	699	0	0	PDLIM1
**COL4A2**	0	-1.72273488051621	428	761	0	0	COL4A2
**ADAMTS1**	0	-1.72995556406749	418	736	0	0	ADAMTS1
**TIMP1**	0	-1.7444025373731	349	779	0	0	TIMP1
**MYL9**	0	-1.75466501053166	264	634	0	0	MYL9
**PLPP3**	0	-1.80941543482772	426	779	0	0	PLPP3
**LGALS1**	0	-1.81032035254868	489	852	0	0	LGALS1
**CD9**	0	-1.84428244125691	247	739	0	0	CD9
**PRSS23**	0	-1.88940401685333	519	773	0	0	PRSS23
**PECAM1**	0	-1.89953881721261	393	726	0	0	PECAM1
**TIMP3**	0	-1.90758615956746	441	704	0	0	TIMP3
**CEBPD**	0	-1.91849347576202	272	693	0	0	CEBPD
**EPAS1**	0	-1.93511701392541	0.47	0.76	0	0	EPAS1
**HSPG2**	0	-1.96285050445549	416	0.74	0	0	HSPG2
**CD74**	0	-2.00732342003426	529	841	0	0	CD74
**COL4A1**	0	-2.01371563698762	0.41	772	0	0	COL4A1
**CAV1**	0	-2.02542518659617	319	714	0	0	CAV1
**CYR61**	0	-2.04361943812036	446	767	0	0	CYR61
**EMP1**	0	-2.15376470804377	485	815	0	0	EMP1
**CTGF**	0	-2.15974069895193	0.51	0.76	0	0	CTGF
**IER3**	0	-2.18713467596484	0.49	824	0	0	IER3
**GSN**	0	-2.2014354604753	0.33	762	0	0	GSN
**C1R**	0	-2.23636484370369	458	0.73	0	0	C1R
**IFI27**	0	-2.25954428030766	279	664	0	0	IFI27
**NNMT**	0	-2.27384734334526	461	782	0	0	NNMT
**COL6A2**	0	-2.37213786861469	394	697	0	0	COL6A2
**ADIRF**	0	-2.48165124278898	304	731	0	0	ADIRF

**A2M**	0	-2.48650588836392	362	712	0	0	A2M
**C11orf96**	0	-2.53227538404561	0.44	708	0	0	C11orf96
**IGFBP4**	0	-2.56262919592996	441	779	0	0	IGFBP4
**CALD1**	0	-2.74283801017476	332	745	0	0	CALD1
**CST3**	0	-2.77842779905837	348	806	0	0	CST3
**AQP1**	0	-2.8448882764279	445	0.73	0	0	AQP1
**SPARC**	0	-3.01124304949733	512	832	0	0	SPARC
**SPARCL1**	0	-3.08952437410546	352	0.7	0	0	SPARCL1
**IGFBP7**	0	-3.51485709707162	0.36	803	0	0	IGFBP7
**GJA1**	4.815	-0.717362257077565	114	541	9.6301327	0	GJA1
**MAFF**	6.687	-0.322261912757325	434	0.73	1.3375626	0	MAFF
**RND3**	4.262	-0.99324370429617	291	609	8.5245640	0	RND3
**SLC39A14**	4.234	-0.496360908649129	0.32	611	8.4699666	0	SLC39A14
**EDNRB**	1.875	-0.393925560353247	59	495	3.7518873	0	EDNRB
**TINAGL1**	2.067	-0.873423404108299	337	607	4.1346710	0	TINAGL1
**PLAUR**	2.143	-0.707233997287491	363	648	4.2870759	0	PLAUR
**COL14A1**	7.678	-1.65319903269286	413	687	1.5357001	0	COL14A1
**FAM107A**	7.5991	-0.617425389062373	142	529	1.5198370	0	FAM107A
**PAPPA**	1.878	-0.732646972360067	349	609	3.7561576	0	PAPPA
**CRYBG3**	6.621	-0.651842689666646	283	603	1.3242693	0	CRYBG3
**HLA-DQB1**	2.814	-1.38137136754872	267	628	5.6292242	0	HLA-DQB1
**ANPEP**	3.146	-0.368288897260152	96	432	6.2931711	0	ANPEP
**JDP2**	1.210	-0.628707283788525	301	602	2.4212653	0	JDP2
**TM4SF1**	1.840	-2.13844426121786	366	666	3.6801066	0	TM4SF1
**RAMP2**	3.3231	-1.61902125706125	159	545	6.6462719	0	RAMP2
**HYAL2**	2.044	-1.29805755595967	192	0.56	4.0897993	0	HYAL2
**MMRN2**	5.2501	-0.757254424732458	79	513	1.0500271	0	MMRN2
**PPFIBP1**	2.284	-0.707709469796347	158	524	4.5690365	0	PPFIBP1
**LTBP2**	2.549	-0.624209357177869	346	619	5.0992267	0	LTBP2
**FBLN1**	6.655	-1.34451878245965	371	0.66	1.3311689	0	FBLN1
**KIAA1217**	1.5557	-0.393946645369268	153	508	3.1114237	0	KIAA1217
**STEAP1**	9.804	-0.47525451031163	156	467	1.9609793	0	STEAP1

**EPHA2**	8.9130	-0.259905457945894	65	491	1.7826101	0	EPHA2
**GSTP1**	4.1777	-0.889699506424494	411	762	8.3555485	0	GSTP1
**SPRY4**	3.7637	-0.274362120077854	156	514	7.5274987	0	SPRY4
**RAI14**	4.5432	-0.972850478455649	0.4	663	9.0864874	0	RAI14
**TSPAN7**	5.3725	-0.816985934839789	185	524	1.0745096	0	TSPAN7
**SH3PXD2A**	8.4755	-0.43311730473078	309	613	1.6951149	0	SH3PXD2A
**HLA-DRA**	2.7538	-2.41753935585953	426	745	5.5077963	0	HLA-DRA
**PRRX2**	7.8184	-0.663746793151086	129	528	1.5636952	0	PRRX2
**CTHRC1**	2.0403	-1.0517191336296	306	611	4.0807749	0	CTHRC1
**C10orf10**	2.3887	-0.679485603729732	0.1	472	4.7774437	0	C10orf10
**CSF3**	1.8379	-0.57995725657101	302	0.57	3.6759627	0	CSF3
**CARHSP1**	8.2047	-0.444153470816547	236	588	1.6409534	0	CARHSP1
**SPATS2L**	1.1595	-0.854418501254408	237	586	2.3191539	0	SPATS2L
**S100A16**	1.5552	-0.976205105194211	222	566	3.1104550	0	S100A16
**HLA-DRB1**	2.3304	-1.51988209081173	411	723	4.6609108	0	HLA-DRB1
**ANK2**	2.2826	-0.268540354609787	19	367	4.5653648	0	ANK2
**HEG1**	5.5915	-1.19099654453525	374	657	1.1183199	0	HEG1
**SELE**	5.6195	-2.49090358710632	418	684	1.1239101	0	SELE
**ADAM15**	3.5917	-0.941420053511974	275	579	7.1834029	0	ADAM15
**TPM1**	2.3740	-1.47588099285092	0.22	559	4.7481535	0	TPM1
**HSPA1B**	2.6769	-0.517602302166489	407	667	5.3538629	0	HSPA1B
**GRASP**	4.4697	-0.757208577651962	333	0.62	8.9394665	0	GRASP
**TEK**	4.7067	-0.394316757610241	186	492	9.4135769	0	TEK
**C1QB**	8.9001	-0.464001565679831	261	0.53	1.7800350	0	C1QB
**CSRP2**	2.1599	-0.703293453649577	79	477	4.3199013	0	CSRP2
**SRPX**	3.0873	-0.30121807677003	68	462	6.1747132	0	SRPX
**DKK 3**	1.9355	-0.574951719360703	319	593	3.8710731	0	DKK 3
**MFAP2**	3.7956	-0.307229933044809	0.16	415	7.5912956	0	MFAP2
**MT2A**	6.2104	-1.6146293501777	375	709	1.2420913	0	MT2A
**JAG2**	8.1559	-0.331749489516405	85	425	1.6311827	0	JAG2
**VWF**	1.1772	-1.922289883909	348	623	2.3544831	0	VWF
**HLA-DPA1**	2.4582	-1.86388154383811	388	695	4.9165397	0	HLA-DPA1

**ADGRL4**	4.213(	-1.22681800787052	372	633	8.4261943;	0	ADGRL4
**RGS16**	5.5719	-1.21883153666501	453	705	1.1143898	0	RGS16
**TYMP**	2.5511	-0.72327735671078	0.47	751	5.1023833	0	TYMP
**ZNF503**	3.7607	-0.512944472414007	201	504	7.5214910	0	ZNF503
**HMOX1**	3.705(	-0.26680688052276	212	505	7.4100921	0	HMOX1
**IL1R1**	8.9807	-0.727754359856699	379	645	1.7961491	0	IL1R1
**UNC5B**	2.7257	-0.331892909290895	183	491	5.4514227	0	UNC5B
**CFI**	1.2853	-0.426978056938427	149	482	2.5707383	0	CFI
**ROBO4**	6.0746	-0.546760558333983	0.17	492	1.2149272	0	ROBO4
**TNS3**	2.0417	-0.350695908452956	231	552	4.0835781	0	TNS3
**PTGDS**	9.203(	-1.92437623259459	308	599	1.8406024	0	PTGDS
**TAGLN**	1.3166	-2.54845659468208	196	502	2.6332276	0	TAGLN
**IGFBP2**	1.1986	-1.99149052868121	0.37	633	2.3973862	0	IGFBP2
**CRIP2**	2.4808	-1.05788217372274	383	0.65	4.9617403	0	CRIP2
**TNFSF10**	1.5731	-0.445753256217911	269	608	3.1463644	0	TNFSF10
**CALCRL**	7.3019	-0.882509268931296	216	513	1.4603891	0	CALCRL
**PRRX1**	1.1965	-1.40708241059812	0.27	564	2.3931915	0	PRRX1
**SDC2**	6.2348	-0.51140730494667	65	424	1.2469730	0	SDC2
**FKBP11**	4.1549	-0.39938859702304	241	567	8.3098438	0	FKBP11
**PTK7**	1.7561	-0.418215111299886	282	551	3.5122528	0	PTK7
**ITGBL1**	2.5307	-0.658868414310304	118	398	5.0614941	0	ITGBL1
**PLEK**	4.4475	-0.337664690389533	176	452	8.8950702	0	PLEK
**PRELP**	1.045(	-0.327808915539217	33	402	2.0901811	0	PRELP
**HSPA5**	1.0963	-0.446132761452516	461	825	2.1927845	0	HSPA5
**POSTN**	1.2616	-1.32116949647623	336	595	2.5232835	0	POSTN
**EMP2**	1.8888	-0.958079211399794	302	569	3.7777338	0	EMP2
**FAM167B**	2.9082	-0.375640950346795	0.2	516	5.8165990	0	FAM167B
**BHLHE41**	1.5794	-0.273964946244695	206	482	3.1588658	0	BHLHE41
**PHLDA2**	3.755(	-0.370636788997278	313	577	7.5101986	0	PHLDA2
**ITGA6**	8.5937	-1.1945838012165	0.4	662	1.7187473	0	ITGA6
**PLXNA2**	6.7171	-0.383069700724669	96	508	1.3434375	0	PLXNA2
**NPDC1**	7.7768	-1.24827412310671	323	605	1.5553780	0	NPDC1

**C1QC**	7.9128	-0.327118971972669	86	423	1.58256928	0	C1QC
**TNC**	4.4543	-0.353332927495202	72	482	8.9086759	0	TNC
**TXN**	4.7384	-0.730505741098429	406	734	9.4768168	0	TXN
**MECOM**	8.5460	-0.34488486913154	123	458	1.70920928	0	MECOM
**RALGPS2**	1.8713	-0.311577570048998	201	484	3.7426807	0	RALGPS2
**MEG3**	1.6751	-0.902024687128384	0.17	458	3.3503780	0	MEG3
**NUPR1**	2.2143	-0.582810392124301	0.1	463	4.4286393	0	NUPR1
**LOXL1**	7.8193	-0.279304847707856	253	521	1.5638656	0	LOXL1
**DUSP23**	9.3011	-0.841222158633006	225	0.53	1.8602393	0	DUSP23
**STEAP4**	1.5768	-0.509632037848057	166	456	3.1537417	0	STEAP4
**ITGA2**	3.6634	-0.25156657732101	138	426	7.3268774	0	ITGA2
**ECM1**	4.4453	-0.558165117093634	0.19	0.48	8.8906036	0	ECM1
**SPNS2**	4.2094	-0.507829409677643	244	533	8.4188080	0	SPNS2
**NCOA7**	2.6064	-0.929044680480557	402	669	5.2128593	0	NCOA7
**PRDX4**	1.0316	-0.693786872327757	241	575	2.0632598	0	PRDX4
**CRABP2**	2.2921	-0.631899176235093	161	433	4.5843557	0	CRABP2
**HLA-DPB1**	8.0531	-1.64485474128863	349	652	1.6106318	0	HLA-DPB1
**LIMCH1**	1.0537	-0.350388126822634	0.17	459	2.1074824	0	LIMCH1
**IL33**	4.0315	-0.56528695233688	177	0.46	8.0630516	0	IL33
**LOX**	1.8898	-0.294047670125432	95	424	3.7797179	0	LOX
**ENPP2**	3.9223	-0.302476588639593	185	485	7.8447938	0	ENPP2
**CTSS**	5.5235	-0.591143640476019	0.39	676	1.1047093	0	CTSS
**DSE**	1.0141	-0.284245792070797	0.2	461	2.0282069	0	DSE
**MXRA8**	1.2579	-0.706645516000525	251	513	2.5159684	0	MXRA8
**GBP 1**	2.6033	-0.272814846831775	343	611	5.2066278	0	GBP 1
**SCPEP1**	1.7405	-0.512648487799071	172	478	3.4810663	0	SCPEP1
**BASP1**	2.1641	-0.733800086473679	261	0.52	4.3282827	0	BASP1
**CLDN5**	2.3055	-1.55080121077143	225	485	4.6110729	0	CLDN5
**MFGE8**	8.0327	-0.763249321689174	268	533	1.6065497	0	MFGE8
**CXCL14**	1.9263	-1.70209312693096	0.18	453	3.8527921	0	CXCL14
**GLIS3**	1.5993	-0.310752476633004	0.2	519	3.1987116	0	GLIS3
**ITGA8**	6.4550	-0.301691560648596	157	488	1.2910175	0	ITGA8

**DNASE1L3**	1.0670	-0.71711955472216	184	434	2.13417884	0	DNASE1L3
**MT1M**	3.4099	-0.675248527302217	117	455	6.81988305	0	MT1M
**FILIP1**	7.7104	-0.436169005431376	66	396	1.54209054	0	FILIP1
**TPST1**	1.6925	-0.271259894518554	135	486	3.38508269	0	TPST1
**HAS2**	8.2173	-0.255062205732699	0.01	374	1.64346393	0	HAS2
**SGCB**	2.1120	-0.416731549383821	221	474	4.22416719	0	SGCB
**LAMP3**	8.5712	-0.26599763565594	99	413	1.71425365	0	LAMP3
**SFRP2**	2.0292	-0.95139085924541	304	565	4.05856930	0	SFRP2
**OGN**	4.0879	-0.3216571367615	171	443	8.17580001	0	OGN
**IGFBP6**	9.4654	-0.580808621414691	229	489	1.89309078	0	IGFBP6
**MASP1**	3.2200	-0.424141812260412	206	478	6.44011800	0	MASP1
**SFRP4**	5.0067	-0.444928111554449	194	445	1.00134882	0	SFRP4
**FAM13C**	2.8358	-0.491298861562503	144	411	5.67165477	0	FAM13C
**TPM2**	6.2185	-1.5528956698723	181	463	1.24371795	0	TPM2
**CLTB**	1.4079	-0.328736593156034	328	603	2.81582975	0	CLTB
**IFI6**	5.4083	-0.427285351621913	222	498	1.08167313	0	IFI6
**SEMA5A**	1.1162	-0.302069066300211	89	388	2.23250802	0	SEMA5A
**JUP**	1.4507	-0.265057194264727	137	422	2.90153094	0	JUP
**ELOVL7**	1.6694	-0.362500815444093	31	395	3.33891703	0	ELOVL7
**PROCR**	3.8585	-0.28858290446697	126	399	7.71711480	0	PROCR
**CTSD**	5.1266	-0.420217036134659	313	582	1.02532883	0	CTSD
**CCDC3**	9.5729	-0.626852964127097	236	511	1.91459274	0	CCDC3
**PLCB4**	2.1797	-0.275648730341246	195	451	4.35950785	0	PLCB4
**PDGFRL**	3.5490	-0.260782008491173	47	407	7.09818631	0	PDGFRL
**PHLDA3**	4.2266	-0.264228294536406	148	424	8.45336015	0	PHLDA3
**ISLR**	3.2180	-0.42166111800323	121	426	6.43607938	0	ISLR
**NUAK1**	3.5795	-0.691461190727834	241	507	7.15901669	0	NUAK1
**OLFM1**	2.5062	-0.480990327098213	185	437	5.01247919	0	OLFM1
**NES**	2.2163	-0.464560156792492	96	417	4.43263945	0	NES
**EDN1**	9.8233	-0.468282761588507	117	397	1.96467304	0	EDN1
**CRYAB**	5.8959	-0.405084226590948	77	334	1.17919478	0	CRYAB
**TMEM45A**	5.5061	-0.25962558126049	58	374	1.10123802	0	TMEM45A

**GNAI1**	3.4578	-0.259052831167674	87	349	6.9156040	0	GNAI1
**EML1**	3.4283	-0.278144700659036	75	364	6.8567525	0	EML1
**SLC7A2**	1.9438	-0.435819163158466	37	308	3.8877087	0	SLC7A2
**SELE.1**	0	4.22020204135694	987	595	0	1	SELE
**ACKR1**	0	4.15157626972762	999	493	0	1	ACKR1
**VWF.1**	0	3.13971151382202	993	523	0	1	VWF
**COL15A1**	0	2.69168544638327	986	622	0	1	COL15A1
**AQP1.1**	0	2.54310340399752	999	642	0	1	AQP1
**ICAM1.1**	0	2.51117972932219	971	703	0	1	ICAM1
**SELP**	0	2.42078318443592	925	493	0	1	SELP
**TM4SF1.1**	0	2.38538802701513	992	567	0	1	TM4SF1
**SLCO2A1**	0	2.23529569728835	951	583	0	1	SLCO2A1
**ENG.1**	0	2.1875564153023	969	552	0	1	ENG
**PRCP**	0	2.15335955916773	958	577	0	1	PRCP
**HSPG2.1**	0	2.14838175238174	985	648	0	1	HSPG2
**PECAM1.1**	0	2.13779261147477	989	0.63	0	1	PECAM1
**PDLIM1.1**	0	2.06828608570068	986	587	0	1	PDLIM1
**C2CD4B.1**	0	2.05655813283628	862	494	0	1	C2CD4B
**RAMP3.1**	0	1.97782835235647	927	431	0	1	RAMP3
**DUSP23.1**	0	1.96497217436837	927	0.42	0	1	DUSP23
**NCOA7.1**	0	1.95642312830553	953	582	0	1	NCOA7
**SPARCL1.1**	0	1.92365495653373	995	597	0	1	SPARCL1
**NPDC1.1**	0	1.91300383024509	965	504	0	1	NPDC1
**ADGRL4.1**	0	1.89252789939221	955	542	0	1	ADGRL4
**ECSCR.1**	0	1.89112504505654	967	0.52	0	1	ECSCR.1
**CD93**	0	1.88393384010253	969	628	0	1	CD93
**TSPAN7.1**	0	1.85499833199084	897	411	0	1	TSPAN7
**CSF3.1**	0	1.85128743678847	813	488	0	1	CSF3
**ZNF385D**	0	1.84160088544129	894	447	0	1	ZNF385D
**EMCN.1**	0	1.81664918546014	938	468	0	1	EMCN
**BMPR2.1**	0	1.80865328933969	0.95	575	0	1	BMPR2
**CSF2RB.1**	0	1.75481235654809	851	529	0	1	CSF2RB

**TGM2**	0	1.73223443510385	888	575	0	1	TGM2
**ADAMTS9**	0	1.69275907233116	908	589	0	1	ADAMTS9
**PLVAP**	0	1.69214031452433	962	527	0	1	PLVAP
**CLU**	0	1.66036531625471	919	576	0	1	CLU
**IGFBP7.1**	0	1.64141586734172	999	696	0	1	IGFBP7
**CRIM1.1**	0	1.62250765495672	941	605	0	1	CRIM1
**RAI14.1**	0	1.62203661990034	933	579	0	1	RAI14
**RCAN1**	0	1.58732880257417	873	482	0	1	RCAN1
**ITGA6.1**	0	1.5714114482382	939	577	0	1	ITGA6
**PKP4**	0	1.54587956173135	878	523	0	1	PKP4
**CLDN5.1**	0	1.54130301246469	883	383	0	1	CLDN5
**HEG1.1**	0	1.53818098483153	923	0.57	0	1	HEG1
**IFI27.1**	0	1.53158477923948	992	549	0	1	IFI27
**PALMD**	0	1.53105661408755	919	497	0	1	PALMD
**WWTR1.1**	0	1.52267503592842	905	545	0	1	WWTR1
**RNASE1**	0	1.51909672781326	903	532	0	1	RNASE1
**HYAL2.1**	0	1.51600583507797	894	448	0	1	HYAL2
**VWA1**	0	1.48548353346284	823	413	0	1	VWA1
**IL33.1**	0	1.48489696762152	775	366	0	1	IL33
**MCTP1**	0	1.47862975773867	866	516	0	1	MCTP1
**CAV1.1**	0	1.46416280810736	971	607	0	1	CAV1
**NUAK1.1**	0	1.46042857278824	779	421	0	1	NUAK1
**RAMP2.1**	0	1.44279936883325	929	423	0	1	RAMP2
**CALCRL.1**	0	1.43844974598941	853	413	0	1	CALCRL
**IL3RA**	0	1.43096379841875	884	538	0	1	IL3RA
**ADAM15.1**	0	1.42095942264859	917	478	0	1	ADAM15
**DLC1.1**	0	1.41392498021641	917	0.59	0	1	DLC1
**NFIB.1**	0	1.40481867418384	922	555	0	1	NFIB
**A2M.1**	0	1.39112916363807	0.99	611	0	1	A2M
**MMRN2.1**	0	1.388154263357	883	384	0	1	MMRN2
**OLFM1.1**	0	1.38194244997391	781	344	0	1	OLFM1
**PCDH17**	0	1.38054191387648	877	506	0	1	PCDH17

**TGFBR3**	0	1.36240064780069	842	0.54	0	1	TGFBR3
**GNG11.1**	0	1.34497137828365	958	531	0	1	GNG11
**CPXM2.1**	0	1.32796382179713	901	605	0	1	CPXM2
**CD34**	0	1.3030283241629	863	464	0	1	CD34
**CLEC14A**	0	1.29879507291479	875	483	0	1	CLEC14A
**SPNS2.1**	0	1.28120926731775	861	435	0	1	SPNS2
**MEOX1**	0	1.27933144342714	737	327	0	1	MEOX1
**LIFR**	0	1.2753276454315	726	402	0	1	LIFR
**CNKSR3**	0	1.27375330556159	835	445	0	1	CNKSR3
**THBD**	0	1.27364793518934	841	513	0	1	THBD
**CXorf36**	0	1.27242550954649	786	413	0	1	CXorf36
**MALL**	0	1.26823536001754	852	537	0	1	MALL
**HES1.1**	0	1.25861023784991	915	625	0	1	HES1
**ADIRF.1**	0	1.25707538916858	964	623	0	1	ADIRF
**SNCG**	0	1.2548593042215	779	417	0	1	SNCG
**DNASE1L3.1**	0	1.24259636736386	0.77	343	0	1	DNASE1L3
**CDH5**	0	1.22595143084884	893	571	0	1	CDH5
**PREX2**	0	1.22319779578383	782	0.38	0	1	PREX2
**EPAS1.1**	0	1.21896829388764	974	679	0	1	EPAS1
**KCTD12**	0	1.1861462325507	843	0.56	0	1	KCTD12
**NOSTRIN**	0	1.18427903248599	735	442	0	1	NOSTRIN
**CYP1B1.1**	0	1.16796465795867	822	529	0	1	CYP1B1
**SPRY1**	0	1.15220146420527	843	561	0	1	SPRY1
**RND1**	0	1.14872374845307	791	491	0	1	RND1
**ITGB4**	0	1.13377286369962	775	385	0	1	ITGB4
**TM4SF18**	0	1.13332593042042	737	447	0	1	TM4SF18
**CD200**	0	1.12958087463718	815	485	0	1	CD200
**DUSP6**	0	1.10714293022597	884	606	0	1	DUSP6
**CRIP2.1**	0	1.08959190716467	881	571	0	1	CRIP2
**EGFL7**	0	1.08742334379054	779	382	0	1	EGFL7
**ARHGAP29**	0	1.0860021569838	824	0.49	0	1	ARHGAP29
**SOX18**	0	1.07715907559783	737	418	0	1	SOX18

**APLNR**	0	1.07650522840619	708	439	0	1	APLNR
**ERG**	0	1.05879789341662	766	396	0	1	ERG
**BCAM**	0	1.05679115440429	0.82	479	0	1	BCAM
**ELOVL7.1**	0	1.05244609401286	756	0.28	0	1	ELOVL7
**ABLIM1**	0	1.04323171071941	789	0.5	0	1	ABLIM1
**LMO2**	0	1.04118979819287	0.73	0.37	0	1	LMO2
**SDPR**	0	1.03990261302726	742	404	0	1	SDPR
**ROBO4.1**	0	1.031300189438	848	385	0	1	ROBO4
**CFI.1**	0	1.02381416147669	698	393	0	1	CFI
**FLNB**	0	1.02092573634747	862	549	0	1	FLNB
**NR2F2.1**	0	1.01848617116903	861	491	0	1	NR2F2
**MMRN1**	0	1.01729544556857	709	364	0	1	MMRN1
**GJA1.1**	0	1.01561094453252	838	424	0	1	GJA1
**NEURL1B**	0	1.01317395353185	804	472	0	1	NEURL1B
**RHOJ**	0	0.994139971519883	829	518	0	1	RHOJ
**RAB3C**	0	0.99344787047802	781	297	0	1	RAB3C
**THBS1**	0	0.992793829470814	822	564	0	1	THBS1
**S100A16.1**	0	0.990917287393245	853	465	0	1	S100A16
**APOLD1**	0	0.987083662430413	867	0.61	0	1	APOLD1
**CYYR1**	0	0.986139505377325	733	375	0	1	CYYR1
**FZD4**	0	0.956085088494893	739	339	0	1	FZD4
**MARCKSL1**	0	0.943134219939898	861	589	0	1	MARCKSL1
**MPZL2**	0	0.931447067367831	824	381	0	1	MPZL2
**FAM167B.1**	0	0.92608291269905	778	424	0	1	FAM167B
**GRASP.1**	0	0.911682733136187	839	538	0	1	GRASP
**CCL14**	0	0.882875440608596	0.61	0.2	0	1	CCL14
**CLDN11**	0	0.87620010408683	0.67	306	0	1	CLDN11
**HLA-DRB5**	0	0.874077454487912	965	713	0	1	HLA-DRB5
**LAMP3.1**	0	0.845049135676968	763	309	0	1	LAMP3
**ESAM.1**	0	0.83419649547192	803	492	0	1	ESAM
**TNFSF10.1**	0	0.826911036071254	818	518	0	1	TNFSF10
**CD9.1**	0	0.811524215410742	943	623	0	1	CD9

**SVIL**	0	0.80318488558415	795	532	0	1	SVIL
**FLT1**	0	0.783564891648248	0.83	0.45	0	1	FLT1
**TEK.1**	0	0.782540015025374	773	398	0	1	TEK
**THSD7A**	0	0.779852649995028	727	373	0	1	THSD7A
**PPFIBP1.1**	0	0.778624474291084	0.78	423	0	1	PPFIBP1
**ABL2.1**	0	0.769762023694793	849	577	0	1	ABL2
**S1PR1**	0	0.764533529615719	799	493	0	1	S1PR1
**COL17A1**	0	0.749238984321532	769	264	0	1	COL17A1
**MCAM.1**	0	0.725795129720518	887	559	0	1	MCAM
**RAPGEF5**	0	0.714584246845356	678	275	0	1	RAPGEF5
**HLA-DRB1.1**	0	0.710888013664933	978	632	0	1	HLA-DRB1
**TNXB**	0	0.687420907403315	771	499	0	1	TNXB
**CST3.1**	0	0.677433821578258	993	698	0	1	CST3
**LAMC2**	0	0.676747329144498	766	311	0	1	LAMC2
**TINAGL1.1**	0	0.66409786801199	809	531	0	1	TINAGL1
**LHX6**	0	0.663780450624841	0.7	352	0	1	LHX6
**CCDC3.1**	0	0.661551179246904	0.74	0.43	0	1	CCDC3
**FAM107A.1**	0	0.630333795536719	801	422	0	1	FAM107A
**C7**	0	0.629075037545869	663	0.24	0	1	C7
**PTAFR**	0	0.604731130103252	727	334	0	1	PTAFR
**ADGRG1**	0	0.589313402308036	794	531	0	1	ADGRG1
**CDKN3**	0	0.579876751508613	692	0.36	0	1	CDKN3
**MED24**	0	0.560446138199573	715	442	0	1	MED24
**INHBB**	0	0.552857051048439	762	504	0	1	INHBB
**EPHA2.1**	0	0.538304810212827	758	378	0	1	EPHA2
**JAG1**	0	0.413635586549429	0.81	521	0	1	JAG1
**NOS3**	0	0.378595259419093	0.64	301	0	1	NOS3
**CYGB**	0	0.348344033674586	708	419	0	1	CYGB
**PDLIM4**	0	0.347433654076499	747	408	0	1	PDLIM4
**INSR**	0	0.343549672914206	792	539	0	1	INSR
**DYSF**	0	0.307287771037114	662	394	0	1	DYSF
**APLN**	0	0.258208103899894	614	184	0	1	APLN

**PTGFR**	0	-0.332283595592174	0.15	461	0	1	PTGFR
**LCP1**	0	-1.29279586276511	303	615	0	1	LCP1
**ZNF331**	0	-1.75320274537844	396	734	0	1	ZNF331
**CYBA**	0	-2.3724829807019	531	833	0	1	CYBA
**S100A4**	0	-3.29989450782192	469	791	0	1	S100A4
**S100A13.1**	2.2756	0.746664656337684	813	506	4.5512423	1	S100A13
**ALDH1A3**	3.0604	0.267373248718596	636	282	6.1209295	1	ALDH1A3
**IGFBP4.1**	2.2540	0.298828571830604	972	692	4.5080089	1	IGFBP4
**CD8A**	1.8918	-0.973372402587507	354	608	3.7837735	1	CD8A
**CDH13**	1.1252	0.306912914932284	724	0.47	2.2505058	1	CDH13
**SNCA**	1.7283	0.560673244457478	622	334	3.4567996	1	SNCA
**CRABP1**	1.6769	-0.519211530452803	56	351	3.3539228	1	CRABP1
**GSN.1**	2.2727	0.461143839387094	916	664	4.5454595	1	GSN
**HSPA1A.1**	7.3776	0.751496650374768	0.89	638	1.4755350	1	HSPA1A
**YPEL2**	8.0384	0.90263739741959	684	433	1.6076889	1	YPEL2
**PDGFRB**	8.9954	-1.08117865095689	339	592	1.7990828	1	PDGFRB
**EFHD2**	1.4887	0.349448054876679	811	538	2.9774432	1	EFHD2
**TBX1**	4.6192	0.361038215087532	619	323	9.2384481	1	TBX1
**HLA-DPB1.1**	4.2159	-0.479873192551703	878	566	8.4319001	1	HLA-DPB1
**FBLIM1**	6.9807	-0.293801487827796	261	522	1.3961590	1	FBLIM1
**C5AR1**	1.9643	-0.257462406653096	237	508	3.9286699	1	C5AR1
**IGLV1-40**	2.4977	-0.405165789910082	448	144	4.9955988	1	IGLV1-40
**HSPB1.1**	1.4302	0.563037157626858	871	581	2.8604556	1	HSPB1
**RCN3**	3.2499	-0.371424196790401	159	431	6.4999333	1	RCN3
**HLA-DMA**	8.5284	0.453852140295721	756	434	1.7056859	1	HLA-DMA
**ICAM2**	1.2192	0.26773534405988	0.76	505	2.4384701	1	ICAM2
**ABCB1**	1.1803	0.900000708306466	635	359	2.3606551	1	ABCB1
**FRZB**	1.0324	-0.718492767414562	98	0.45	2.0649108	1	FRZB
**EPSTI1**	2.7025	-0.361126873582442	182	467	5.4051954	1	EPSTI1
**COL5A3**	4.6527	-0.323318575228529	181	0.5	9.3055938	1	COL5A3
**MARCH1**	2.1317	-0.257353305054591	73	394	4.2635720	1	MARCH1
**PLXNA2.1**	1.5102	0.456956055881962	666	413	3.0205283	1	PLXNA2

**ITGA2.1**	1.7642	0.607740956591753	616	348	3.5284002	1	ITGA2
**LRG1**	6.0486	0.397297350985219	565	0.27	1.2097258	1	LRG1
**TNFSF13B**	4.2345	-0.335489596140583	0.18	487	8.4690936	1	TNFSF13B
**PLEKHA5**	6.7368	-0.576263524003983	247	525	1.3473664	1	PLEKHA5
**TACR1**	2.0669	0.696956235373212	583	261	4.1339670	1	TACR1
**ADRA2A**	1.5779	-0.340155664201202	0.04	354	3.1559383	1	ADRA2A
**PRRX1.1**	1.9061	-1.43174590120779	296	549	3.8122319	1	PRRX1
**CARHSP1.1**	6.4779	0.42915579259988	0.76	501	1.2955935	1	CARHSP1
**SPON2**	1.5341	-0.50089595691467	123	477	3.0683554	1	SPON2
**FBLN2**	1.7513	-0.396493040174292	0.75	0.5	3.5026750	1	FBLN2
**PROCR.1**	4.1982	0.33393013401718	0.58	326	8.3965224	1	PROCR
**SOX17**	5.5963	0.543050845173141	564	292	1.1192657	1	SOX17
**PROX1**	1.6666	0.267066369422843	493	158	3.3332743	1	PROX1
**MS4A1**	7.1985	-0.591177615624922	131	405	1.4397049	1	MS4A1
**ANGPT1**	1.7229	-0.390768074866658	256	511	3.4459276	1	ANGPT1
**TBX2**	5.5450	-0.353580426755916	213	476	1.1090196	1	TBX2
**IGLV2-14.1**	9.8022	-0.317329721305886	1	0.26	1.9604461	1	IGLV2-14
**FAM110D**	1.3514	0.612681358937764	461	189	2.7028133	1	FAM110D
**NTRK2**	1.2854	-0.382798211369716	92	383	2.5708075	1	NTRK2
**CCR6**	3.2011	-0.282122563890972	34	307	6.4023191	1	CCR6
**CCL4**	0	4.0994806462047	851	572	0	2	CCL4
**CCL5**	0	3.45205089605453	895	556	0	2	CCL5
**NKG7**	0	2.97477775652203	778	465	0	2	NKG7
**GZMA**	0	2.76909196707708	782	0.51	0	2	GZMA
**DUSP2**	0	2.23753985322938	889	616	0	2	DUSP2
**CD3D**	0	2.05228333889116	0.82	561	0	2	CD3D
**ABL2.2**	0	-0.697665315766303	239	648	0	2	ABL2
**CPXM2.2**	0	-0.880189798974708	288	677	0	2	CPXM2
**PXDN.1**	0	-0.892189223232975	329	728	0	2	PXDN
**RAB31.1**	0	-0.898030995539774	181	673	0	2	RAB31
**INSR.1**	0	-0.944586384757183	154	613	0	2	INSR
**DLC1.2**	0	-0.997976367153339	272	667	0	2	DLC1

**NFIB.2**	0	-1.15138868646462	147	646	0	2	NFIB
**ICAM1.2**	0	-1.48993510938962	461	764	0	2	ICAM1
**KLF4.1**	0	-1.53029602274709	407	748	0	2	KLF4
**PDLIM1.2**	0	-1.66322152848648	241	676	0	2	PDLIM1
**PLPP3.1**	0	-1.75279461389911	429	754	0	2	PLPP3
**CD9.2**	0	-1.86151702449104	252	704	0	2	CD9
**HSPG2.2**	0	-1.94476937577139	326	726	0	2	HSPG2
**IER3.1**	0	-2.02362773904577	462	804	0	2	IER3
**TIMP1.1**	0	-2.03688788593814	354	748	0	2	TIMP1
**GSN.2**	0	-2.06616475654024	258	739	0	2	GSN
**EMP1.1**	0	-2.24736765378079	414	799	0	2	EMP1
**ADIRF.2**	0	-2.42917223444467	296	702	0	2	ADIRF
**CST3.2**	0	-2.54032048899102	283	0.78	0	2	CST3
**IGFBP4.2**	0	-2.60439143881027	349	765	0	2	IGFBP4
**SPARC.1**	0	-2.9725522792783	488	812	0	2	SPARC
**IGFBP7.2**	0	-3.44688471467114	356	772	0	2	IGFBP7
**SLC39A14.1**	1.7328	-0.473680263055533	167	606	3.4656766	2	SLC39A14
**RND3.1**	2.7236	-1.00066104696696	174	598	5.4472157	2	RND3
**COL4A2.1**	3.3743	-1.63315261176625	412	739	6.7487954	2	COL4A2
**COL4A1.1**	5.8098	-1.92707146959344	419	745	1.1619748	2	COL4A1
**SERPINH1.1**	3.2332	-1.17492168789107	413	741	6.4664525	2	SERPINH1
**ADAMTS4**	3.4052	-0.479573364082789	212	587	6.8105840	2	ADAMTS4
**CALD1.1**	4.2849	-2.70307527575228	356	714	8.5698263	2	CALD1
**ENG.2**	4.7840	-1.59906190645044	0.23	641	9.5681844	2	ENG
**RAMP3.2**	1.5637	-1.14818398748301	84	533	3.1275739	2	RAMP3
**TSC22D1.1**	1.7691	-1.38735303377561	0.3	697	3.5383714	2	TSC22D1
**CDKN1A**	1.6789	-1.00599938837376	553	813	3.3578992	2	CDKN1A
**A2M.2**	8.2068	-2.35518355080742	327	691	1.6413620	2	A2M
**WWTR1.2**	1.1506	-1.01445404433469	233	625	2.3013044	2	WWTR1
**HES1.2**	2.6587	-1.39243391257645	337	693	5.3174481	2	HES1
**EPAS1.2**	5.0079	-1.88185533191246	458	741	1.0015872	2	EPAS1
**CFH.1**	1.9660	-1.24708380322323	237	627	3.9320690	2	CFH

**CXCL2**	1.2338	-1.5625999996527	381	686	2.4676012	2	CXCL2
**HSPA1A.2**	5.3721	-1.2910371816254	307	705	1.0744215	2	HSPA1A
**KRT86**	5.1554	0.406453936181265	0.52	245	1.0310806	2	KRT86
**IL1R1.1**	1.1121	-0.830291997663681	234	642	2.2243889	2	IL1R1
**IL3RA.1**	6.3609	-0.803377843352341	255	613	1.2721883	2	IL3RA
**SOD2**	3.8316	-2.01664977801413	413	771	7.6632625	2	SOD2
**SPARCL1.2**	7.9061	-3.04771001628459	336	677	1.5812232	2	SPARCL1
**FSTL1.1**	8.9627	-1.55792772519085	448	742	1.7925544	2	FSTL1
**PECAM1.2**	2.3172	-1.71578998786458	361	706	4.6344354	2	PECAM1
**PKP4.1**	1.7086	-0.654994524476976	224	601	3.4173180	2	PKP4
**THBD.1**	9.8256	-0.919170557071156	199	589	1.9651247	2	THBD
**COL14A1.1**	1.1454	-1.58713147241598	361	673	2.2908099	2	COL14A1
**MYL9.1**	4.7100	-1.7224626000238	229	611	9.4200081	2	MYL9
**AQP1.2**	1.0081	-2.7355150228158	393	715	2.0162792	2	AQP1
**CALU.1**	2.4350	-0.929603139845945	278	657	4.8701138	2	CALU
**XBP1.1**	5.7248	-0.925893925210346	245	702	1.1449789	2	XBP1
**NNMT.1**	2.3293	-2.1910341056496	496	756	4.6587947	2	NNMT
**CDH13.1**	6.1393	-0.3894596785562	139	538	1.2278641	2	CDH13
**ECSCR.1.1**	7.1004	-1.20088074497119	237	609	1.4200950	2	ECSCR.1
**BMPR2.2**	1.6828	-1.00180718888574	307	653	3.3656858	2	BMPR2
**CLU.1**	5.6111	-1.66187432444318	311	649	1.1222371	2	CLU
**ACKR3**	8.9854	-0.60640826739562	323	642	1.7970834	2	ACKR3
**LGALS3.1**	1.3550	-1.41449424969635	0.36	733	2.7101438	2	LGALS3
**ADAMTS1.1**	8.8625	-1.61873770638131	434	712	1.7725008	2	ADAMTS1
**CAV1.2**	1.3389	-1.9721830940425	394	678	2.6778331	2	CAV1
**IFI27.2**	1.7245	-2.24549157647273	257	639	3.4490129	2	IFI27
**PRSS23.1**	4.0130	-1.8175065993373	487	758	8.0261868	2	PRSS23
**CEBPD.1**	6.0630	-1.90124160553954	283	662	1.2126073	2	CEBPD
**SGK1**	7.0888	-1.03908736833096	376	682	1.4177735	2	SGK1
**FAM167B.2**	2.6686	-0.361836293869105	115	503	5.3373456	2	FAM167B
**FBN1.1**	4.2588	-1.6181802230755	425	693	8.5176357	2	FBN1
**HSP90B1.1**	1.3708	-0.981804044871611	522	867	2.7417091	2	HSP90B1

**FN1**	2.4361	-1.18485120372363	0.32	0.63	4.8722715(	2	FN1
**FILIP1L.1**	2.5059	-1.3333376306244	299	624	5.0118672(	2	FILIP1L
**CTHRC1.1**	4.9308	-1.06250661402357	238	597	9.8617013(	2	CTHRC1
**GRN.1**	8.3087	-1.05611200255752	315	652	1.6617471	2	GRN
**FLT1.1**	2.8570	-1.00293103027873	154	532	5.7140923	2	FLT1
**LAMA4.1**	1.3972	-0.965612863525602	247	604	2.7945327	2	LAMA4
**SFRP1.1**	3.7281	-0.384126324766567	48	469	7.4563119	2	SFRP1
**AKAP12.1**	7.2959	-0.797510524302899	338	613	1.4591925	2	AKAP12
**PRDX4.1**	1.5394	-0.874254941284447	137	563	3.0789850	2	PRDX4
**RHOB.1**	5.0163	-1.13297577580191	349	656	1.0032661	2	RHOB
**IGFBP5**	2.9070	-2.7127255754649	0.33	661	5.8141451(	2	IGFBP5
**PCDH17.1**	2.1430	-0.927878684201125	232	584	4.2861685	2	PCDH17
**FBLN1.1**	3.2266	-1.26670627611751	301	647	6.4532609	2	FBLN1
**KCTD12.1**	1.2491	-1.07134804812468	336	621	2.4983379(	2	KCTD12
**SPRY4.1**	1.4318	-0.255247606368785	91	495	2.8637421	2	SPRY4
**CCL2**	5.2017	-1.79377065703012	389	668	1.0403461	2	CCL2
**MAP1B.1**	3.5234	-1.15689666441025	209	0.56	7.0468613(	2	MAP1B
**CD79A**	6.9998	-0.864091859036575	179	521	1.3999674(	2	CD79A
**FAM107A.2**	7.8112	-0.606820993081912	191	496	1.5622425(	2	FAM107A
**CSF2RB.2**	1.1731	-0.632562632476064	323	593	2.3463109	2	CSF2RB
**ATF3.1**	6.2846	-0.89501197850885	524	783	1.2569383	2	ATF3
**CTSB.1**	1.7248	-1.13349465404399	315	663	3.4497883(	2	CTSB
**TFPI**	1.2442	-0.894209916964679	346	638	2.4885993	2	TFPI
**BMP2**	2.4359	-0.35915897326604	247	573	4.8718345	2	BMP2
**INHBB.1**	2.7440	-0.321844412730825	264	563	5.4880933(	2	INHBB
**ROBO4.2**	3.0172	-0.535720624464691	75	479	6.0345513(	2	ROBO4
**S100A13.2**	2.7526	-1.04041018698282	224	576	5.5052395	2	S100A13
**SELP.1**	6.3570	-0.811464568774726	0.25	576	1.2714115	2	SELP
**GRASP.2**	7.4194	-0.761467628157315	282	605	1.4838866	2	GRASP
**PGF**	8.4713	-0.359514880171347	158	0.53	1.6942691	2	PGF
**PDGFRA.1**	1.3008	-0.826260756497822	307	628	2.6016125	2	PDGFRA
**HSPB1.2**	1.5505	-1.10485969558958	288	0.65	3.1011388	2	HSPB1

**ADAMTS9.1**	2.7896	-0.863772507973573	386	652	5.5793451	2	ADAMTS9
**SELE.2**	4.0500	-2.40845329970206	389	669	8.1000080	2	SELE
**RGS16.1**	7.6250	-1.22786620124336	422	691	1.5250000	2	RGS16
**GLUL.1**	4.2081	-1.16124054593753	292	642	8.4162564	2	GLUL
**ROBO1**	1.5582	-0.472805892319762	208	0.56	3.1165220	2	ROBO1
**NUAK1.2**	5.2272	-0.660623905561712	62	506	1.0454534	2	NUAK1
**CRIM1.2**	1.1240	-0.964826274180093	397	672	2.2480942	2	CRIM1
**NCOA7.2**	1.2895	-0.957158879981	329	658	2.5790682	2	NCOA7
**FGFR1**	4.0507	-0.93243087587222	293	623	8.1015138	2	FGFR1
**LGALS1.1**	8.1065	-1.42558582683406	516	824	1.6213015	2	LGALS1
**RHOJ.1**	1.4630	-0.472377875437771	257	586	2.9261534	2	RHOJ
**RNASE1.1**	2.4838	-1.20992318853916	0.28	607	4.9676908	2	RNASE1
**FKBP11.1**	9.5822	-0.462156926942659	174	551	1.9164536	2	FKBP11
**MGP**	6.4536	-2.04202875825049	309	618	1.2907250	2	MGP
**EMILIN1**	8.1764	-0.475563455043096	121	426	1.6352911	2	EMILIN1
**EPHA2.2**	1.0620	-0.251362130137895	87	458	2.1241516	2	EPHA2
**S100A16.2**	6.6376	-0.967572918289636	199	544	1.3275359	2	S100A16
**GNG11.2**	1.4035	-1.57547987555829	292	613	2.8071493	2	GNG11
**S100A11**	7.5598	-0.977567244568911	584	848	1.5119771	2	S100A11
**LGMN**	3.5713	-0.417520287204864	329	603	7.1426799	2	LGMN
**MYLK**	2.8685	-0.846359317632427	285	576	5.7370842	2	MYLK
**F3**	4.4699	-0.275962390876271	171	535	8.9399421	2	F3
**GATA2**	7.4055	-0.369188501087567	186	0.52	1.4811174	2	GATA2
**LGALS3BP**	9.3464	-0.625920002461996	251	568	1.8692873	2	LGALS3BP
**C2CD4B.2**	2.4111	-0.743432175951932	287	564	4.8223561	2	C2CD4B
**HMCN1**	5.1017	-0.321745157902049	148	505	1.0203572	2	HMCN1
**MCAM.2**	1.6252	-1.18299600635471	347	625	3.2504428	2	MCAM
**COL6A2.1**	3.9171	-2.29471710363055	406	675	7.8342590	2	COL6A2
**LEPR**	4.4661	-0.806453180442967	318	593	8.9323837	2	LEPR
**CYGB.1**	1.3350	-0.319174408846737	168	484	2.6700233	2	CYGB
**CCND1**	6.6157	-0.703426138358707	275	578	1.3231434	2	CCND1
**JDP2.1**	4.1719	-0.596771182713655	0.28	583	8.3439847	2	JDP2

**MDK**	2.0088	-0.68305340091148	267	576	4.0176584	2	MDK
**HEG1.2**	5.3291	-1.11927696224295	348	0.64	1.0658261	2	HEG1
**LTBP2.1**	1.3233	-0.599933750768732	307	603	2.6466195	2	LTBP2
**PRCP.1**	1.8440	-1.06256887253686	387	648	3.6881357	2	PRCP
**PLAC9**	2.4196	-0.788081119498475	213	529	4.8393747	2	PLAC9
**LMCD1**	3.5668	-0.711917755082959	354	626	7.1336781	2	LMCD1
**GGT5.1**	1.4368	-0.898881627230683	349	613	2.8736743	2	GGT5
**PHLDA2.1**	7.6876	-0.463155555799304	271	563	1.5375286	2	PHLDA2
**ZNF385D.1**	9.2344	-0.747919967877083	224	0.53	1.8468805	2	ZNF385D
**ITGA6.2**	1.5767	-1.25260693652671	367	647	3.1534845	2	ITGA6
**VCAM1.1**	1.6315	-0.626267123738025	405	681	3.2630401	2	VCAM1
**UNC5B.1**	6.6430	-0.320785225392024	165	472	1.3286012	2	UNC5B
**NPDC1.2**	9.7351	-1.2903304723157	283	589	1.9470245	2	NPDC1
**ITIH5**	1.3398	-0.33738773947499	141	482	2.6796458	2	ITIH5
**S1PR1.1**	2.8423	-0.366697896675415	282	555	5.6847403	2	S1PR1
**CFD**	6.2471	-2.04637357528993	0.34	603	1.2494269	2	CFD
**KIAA1217.1**	7.2899	-0.363775822091131	88	489	1.4579890	2	KIAA1217
**TM4SF1.2**	2.3299	-2.14161575272822	389	643	4.6599818	2	TM4SF1
**HTRA1.1**	6.6245	-1.16480670595104	0.37	629	1.3249001	2	HTRA1
**TNC.1**	8.5408	-0.3343230446159	134	447	1.7081658	2	TNC
**TMEM176A**	1.3892	-0.721680863722739	223	539	2.7784443	2	TMEM176A
**MPZL2.1**	8.6539	-0.463742228028538	0.19	0.46	1.7307899	2	MPZL2
**RAI14.2**	8.7122	-0.900645802972297	387	646	1.7424527	2	RAI14
**TGFBI**	2.7361	-0.425358636698258	218	511	5.4722460	2	TGFBI
**GSTP1.1**	3.8657	-0.816887870495736	448	733	7.7315383	2	GSTP1
**ADGRL4.2**	4.9199	-1.19891516784707	352	617	9.8398253	2	ADGRL4
**VEGFA**	8.6110	-0.462311420642261	204	525	1.7222066	2	VEGFA
**FBLN2.1**	3.0171	-1.44307257652402	268	557	6.0342282	2	FBLN2
**CD34.1**	2.6725	-0.921787666576498	239	0.54	5.3450704	2	CD34
**BASP1.1**	3.0487	-0.7357840112828	181	0.51	6.0974152	2	BASP1
**TNFSF10.2**	1.3623	-0.511780918254219	0.26	585	2.7247792	2	TNFSF10
**ADAM15.2**	9.1092	-0.924244997188582	0.28	558	1.8218584	2	ADAM15

**GLIS3.1**	1.2771	-0.301759035048917	128	504	2.5542424	2	GLIS3
**PRKCDBP**	1.8854	-0.923896755963979	0.19	536	3.7708028	2	PRKCDBP
**BHLHE41.1**	2.2684	-0.276423626784177	109	473	4.5368978	2	BHLHE41
**EFEMP1**	2.4631	-0.57820195301457	348	603	4.9263809	2	EFEMP1
**TYMP.1**	7.5122	-0.661039860174301	451	733	1.5024485	2	TYMP
**DST.1**	2.0025	-1.0123640544193	277	558	4.0050934	2	DST
**EDNRB.1**	3.8783	-0.380609502973573	76	463	7.7567085	2	EDNRB
**FAT1.1**	5.1174	-0.302320519730003	75	412	1.0234970	2	FAT1
**DUSP6.1**	1.3906	-0.581126765116333	407	664	2.7813608	2	DUSP6
**DIO2**	1.6544	-1.42732034774821	321	593	3.3088909	2	DIO2
**OGN.1**	4.9411	-0.321416347166808	104	0.43	9.8822144	2	OGN
**ECM1.1**	2.6484	-0.536241213947641	0.11	468	5.2968392	2	ECM1
**CXCL1**	3.5445	-1.22847314135505	272	537	7.0891936	2	CXCL1
**THBS2**	4.9531	-0.753678210903953	294	596	9.9063798	2	THBS2
**MECOM.1**	1.8028	-0.330315008833975	0.07	0.44	3.6057252	2	MECOM
**MOXD1**	5.0862	-0.325960204737686	0.08	0.43	1.0172556	2	MOXD1
**CPE**	1.1912	-0.954110538319183	254	551	2.3825720	2	CPE
**MRVI1**	4.7052	-0.257376790011871	67	417	9.4105859	2	MRVI1
**PPFIBP1.2**	1.2250	-0.667765441637569	195	494	2.4501729	2	PPFIBP1
**CFI.2**	1.5560	-0.398484531360231	76	466	3.1120301	2	CFI
**ID1**	2.5687	-1.13346738614145	324	0.6	5.1374060	2	ID1
**GAS6**	4.9324	-0.804894965751805	307	578	9.8649573	2	GAS6
**DNASE1L3.2**	1.5450	-0.730567858102246	127	422	3.0901430	2	DNASE1L3
**TPM1.1**	1.7582	-1.42100280421933	0.24	533	3.5164938	2	TPM1
**FSCN1**	3.4807	-0.477162842633643	297	547	6.9615791	2	FSCN1
**SEPP1**	9.5857	-0.931329785724504	213	524	1.9171596	2	SEPP1
**MASP1.1**	1.9019	-0.414568450296832	147	465	3.8038268	2	MASP1
**MT1M.1**	2.3372	-0.650489414671896	79	435	4.6744156	2	MT1M
**CD68**	8.1095	-0.357950096921742	276	541	1.6219029	2	CD68
**ACKR1.1**	1.9159	-2.50246093860007	317	579	3.8319832	2	ACKR1
**ANPEP.1**	1.5676	-0.329111282203707	142	404	3.1353054	2	ANPEP
**SERPINF1.1**	2.5883	-1.46979479987707	328	585	5.1767537	2	SERPINF1

**GRB10**	2.7992	-0.320219258484539	212	527	5.5984669	2	GRB10
**KYNU**	2.7009	-0.352230111457014	97	476	5.4019060	2	KYNU
**PTPRB**	4.4916	-0.607424595703197	289	566	8.9832968	2	PTPRB
**SMAD1**	5.6301	-0.412494425549062	0.06	469	1.1260277	2	SMAD1
**ALPL**	3.5386	-0.474449498672635	229	535	7.0772888	2	ALPL
**RAMP2.2**	1.4676	-1.52209691702296	233	0.51	2.9353046	2	RAMP2
**CEBPB**	1.9880	-0.838617493842772	391	0.67	3.9761885	2	CEBPB
**CLMP**	4.6528	-0.268292018029617	43	314	9.3057171	2	CLMP
**PLOD2**	4.8331	-0.408690173599649	0.21	521	9.6663392	2	PLOD2
**RGS3.1**	5.5739	-0.473954936449933	344	612	1.1147821	2	RGS3
**ADM5**	3.1334	-0.368750445392716	137	474	6.2669880	2	ADM5
**NUPR1.1**	3.4447	-0.567238843812337	114	436	6.8895525	2	NUPR1
**SLIT3.1**	4.5726	-0.590698216064009	144	459	9.1452634	2	SLIT3
**CARHSP1.2**	2.3914	-0.363749504737936	274	559	4.7829374	2	CARHSP1
**FAM13C.1**	6.6646	-0.476801114624038	0.03	404	1.3329347	2	FAM13C
**KDR**	1.0669	-0.555650296388406	217	481	2.1338677	2	KDR
**COL11A1.1**	1.7390	-0.607380541894334	97	443	3.4780894	2	COL11A1
**IGFBP6.1**	1.4739	-0.57678532910132	219	472	2.9478067	2	IGFBP6
**LIFR.1**	1.4199	-0.588887958505826	111	475	2.8398276	2	LIFR
**SOD3**	7.5183	-1.57680072683846	291	555	1.5036742	2	SOD3
**PLPP1**	3.3155	-0.568382599058529	295	561	6.6310451	2	PLPP1
**ENAH**	8.6895	-0.66950812742534	213	0.5	1.7379156	2	ENAH
**IL33.2**	2.9364	-0.538839588735505	133	445	5.8729993	2	IL33
**FRMD6**	3.9350	-0.571968065043271	0.12	421	7.8701535	2	FRMD6
**PRELP.1**	8.5450	-0.311392106745088	82	371	1.7090150	2	PRELP
**ERG.1**	2.9810	-0.466578726880174	208	466	5.9620119	2	ERG
**EDIL3**	5.4934	-0.790348782190903	297	591	1.0986997	2	EDIL3
**UACA**	1.6518	-0.706889218357706	186	496	3.3037548	2	UACA
**PCSK5**	7.2338	-0.460244252449026	293	547	1.4467721	2	PCSK5
**RALGPS2.1**	1.0661	-0.343263828319833	208	463	2.1323783	2	RALGPS2
**OLFML3**	2.1163	-0.488702728262382	202	0.48	4.2327778	2	OLFML3
**TXN.1**	2.7150	-0.604233893871079	409	711	5.4300265	2	TXN

**TM4SF18.1**	8.8670	-0.590774138469334	241	507	1.77341529	2	TM4SF18
**DIRAS3**	7.1552	-0.2818334322432	248	499	1.43105539	2	DIRAS3
**CD200.1**	3.9692	-0.493497871906589	272	552	7.93849515	2	CD200
**IGF1**	1.4284	-0.430768448001784	265	533	2.85696641	2	IGF1
**SDC1**	5.8996	-0.356330694184789	234	485	1.17992167	2	SDC1
**EGFL7.1**	1.0049	-0.805375755104386	192	455	2.00994444	2	EGFL7
**GPX3**	2.5234	-0.320858876598663	222	508	5.04681591	2	GPX3
**PID1**	7.1419	-0.315310720832478	37	313	1.42838210	2	PID1
**ENPP2.1**	7.7805	-0.284769897397143	167	466	1.55611942	2	ENPP2
**LMO4**	9.7716	-0.431230502882228	252	519	1.95432908	2	LMO4
**JUP.1**	1.9832	-0.25158935632851	124	403	3.96655159	2	JUP
**COL5A3.1**	2.3263	-0.274607340727334	224	485	4.65274855	2	COL5A3
**MEOX1.1**	1.3876	-0.36969526411925	135	402	2.77529512	2	MEOX1
**HHEX**	1.4517	-0.263641084888666	65	366	2.90346364	2	HHEX
**PLXNA2.2**	5.5498	-0.345312585283536	179	471	1.10996217	2	PLXNA2
**THSD7A.1**	7.7557	-0.654428931289471	173	441	1.55115921	2	THSD7A
**SRPX.1**	9.6144	-0.282028413913354	153	425	1.92289043	2	SRPX
**STX11**	2.6164	0.27689695843499	314	567	5.23285978	2	STX11
**NES.1**	2.5874	-0.449633030468243	56	399	5.17499257	2	NES
**SDC2.1**	4.4821	-0.473767343498657	109	394	8.96426525	2	SDC2
**IFI6.1**	1.7533	-0.332364375295905	221	478	3.50665603	2	IFI6
**CTSD.1**	6.8420	-0.330360726816782	305	564	1.36840708	2	CTSD
**LMO2.1**	3.9847	-0.660914563543195	155	0.44	7.96953641	2	LMO2
**ELOVL7.2**	3.9460	-0.346435932287806	52	367	7.89204890	2	ELOVL7
**MRC2**	5.2220	-0.595423519169361	0.19	464	1.04440059	2	MRC2
**PTGR1**	5.2967	-0.270385437996922	138	412	1.05935107	2	PTGR1
**SYNPO2**	1.1578	-0.379818161081779	168	433	2.31577031	2	SYNPO2
**PHACTR1**	1.3327	-0.347497121288513	153	441	2.66555533	2	PHACTR1
**RAPGEF5.1**	3.7405	-0.398426373326113	66	0.35	7.48102973	2	RAPGEF5
**IGLV3-21**	4.2273	-0.547784725163823	102	0.37	8.45465410	2	IGLV3-21
**CYP26B1**	7.0624	-0.307685943119908	62	346	1.41249270	2	CYP26B1
**LOX.1**	1.8036	-0.275352712575018	101	0.4	3.60723487	2	LOX

**RBP7**	5.8096	-0.287035980827638	64	0.32	1.1619386	2	RBP7
**CFD.1**	0	4.35120936886966	941	549	0	3	CFD
**APOD**	0	3.66600974108473	807	475	0	3	APOD
**IGFBP5.1**	0	3.51034659653914	973	602	0	3	IGFBP5
**VCAN**	0	3.48358583868415	977	633	0	3	VCAN
**CXCL14.1**	0	3.21859984843064	818	376	0	3	CXCL14
**PTGDS.1**	0	3.0339125089941	845	529	0	3	PTGDS
**FBLN1.2**	0	2.96773688012389	967	585	0	3	FBLN1
**COL6A1**	0	2.86207264178931	995	632	0	3	COL6A1
**DCN**	0	2.82870354602779	1	0.64	0	3	DCN
**COL6A2.2**	0	2.82582106469197	993	621	0	3	COL6A2
**C1R.1**	0	2.70727586936091	988	662	0	3	C1R
**C1S**	0	2.69011367027695	993	601	0	3	C1S
**COL6A3**	0	2.66963778584127	986	646	0	3	COL6A3
**CXCL12**	0	2.6661929830883	956	691	0	3	CXCL12
**PCOLCE**	0	2.59553568077024	966	474	0	3	PCOLCE
**FGF7**	0	2.55989192892103	838	365	0	3	FGF7
**COL14A1.2**	0	2.5182570284106	965	617	0	3	COL14A1
**RARRES2**	0	2.51077890971731	975	0.62	0	3	RARRES2
**LUM**	0	2.46549145797233	979	653	0	3	LUM
**CCDC80**	0	2.40810995316479	933	0.44	0	3	CCDC80
**AEBP1**	0	2.39209925570082	967	647	0	3	AEBP1
**SERPINF1.2**	0	2.38337664719119	969	528	0	3	SERPINF1
**CTSK**	0	2.38112356702028	957	552	0	3	CTSK
**NNMT.2**	0	2.35569807969906	977	711	0	3	NNMT
**ASPN**	0	2.34057142289052	767	454	0	3	ASPN
**COL5A2**	0	2.32090346921437	956	575	0	3	COL5A2
**MEG3.1**	0	2.29877124189445	861	375	0	3	MEG3
**MMP2**	0	2.24362749580416	951	609	0	3	MMP2
**FBN1.2**	0	2.19943038064312	953	644	0	3	FBN1
**LRP1**	0	2.19217272158622	935	519	0	3	LRP1
**MT2A.1**	0	2.08894044099391	939	633	0	3	MT2A

**CEBPD.2**	0	2.08359061641221	972	598	0	3	CEBPD
**PRRX1.2**	0	2.07471806095733	948	482	0	3	PRRX1
**IGFBP4.3**	0	2.04175363322215	992	704	0	3	IGFBP4
**ITGBL1.1**	0	1.98132823021925	827	315	0	3	ITGBL1
**FBLN2.2**	0	1.96270730550054	908	499	0	3	FBLN2
**THY1**	0	1.95862151205056	923	459	0	3	THY1
**COL5A1**	0	1.94885708428951	0.9	493	0	3	COL5A1
**TNFAIP6**	0	1.91258416477855	0.82	485	0	3	TNFAIP6
**SERPING1.1**	0	1.90690879182524	955	603	0	3	SERPING1
**MXRA8.1**	0	1.90160629407126	872	438	0	3	MXRA8
**SOD3.1**	0	1.89916305635673	927	498	0	3	SOD3
**TWIST1**	0	1.89260517327476	916	416	0	3	TWIST1
**FSTL1.2**	0	1.8912795699951	969	693	0	3	FSTL1
**HTRA1.2**	0	1.88790074860037	894	581	0	3	HTRA1
**GAS1**	0	1.86484775533384	0.8	481	0	3	GAS1
**FGFR1.1**	0	1.86082401770179	918	565	0	3	FGFR1
**MFAP4**	0	1.84776282902844	822	0.4	0	3	MFAP4
**MXRA5**	0	1.82712201521239	882	472	0	3	MXRA5
**THBS2.1**	0	1.82227172056783	0.8	548	0	3	THBS2
**PDGFRA.2**	0	1.80405415965018	871	575	0	3	PDGFRA
**OGN.2**	0	1.78798893008677	682	377	0	3	OGN
**EDIL3.1**	0	1.78585445336276	865	539	0	3	EDIL3
**CEBPB.1**	0	1.76356650731879	958	618	0	3	CEBPB
**CDH11**	0	1.76171548983215	885	521	0	3	CDH11
**FN1.1**	0	1.75053904556384	897	577	0	3	FN1
**CFH.2**	0	1.74663143875313	0.83	571	0	3	CFH
**OLFML3.1**	0	1.67628424023434	807	425	0	3	OLFML3
**C3**	0	1.6532983956275	741	391	0	3	C3
**OMD**	0	1.63463960276514	725	393	0	3	OMD
**FMOD**	0	1.61030993041977	868	546	0	3	FMOD
**TIMP1.2**	0	1.60273125258932	967	691	0	3	TIMP1
**MRC2.1**	0	1.59843266116691	861	403	0	3	MRC2

**IGF2**	0	1.59679635025131	787	533	0	3	IGF2
**SFRP1.2**	0	1.59238941681262	698	407	0	3	SFRP1
**BGN**	0	1.56998766265153	866	594	0	3	BGN
**MDK.1**	0	1.55164796627232	782	528	0	3	MDK
**WNT5A**	0	1.54646240711486	813	555	0	3	WNT5A
**TMEM176B**	0	1.5100510050501	813	472	0	3	TMEM176B
**SSPN**	0	1.50598221724136	802	483	0	3	SSPN
**RND3.2**	0	1.50571857502466	848	535	0	3	RND3
**SPATS2L.1**	0	1.49023180403464	861	504	0	3	SPATS2L
**PLAC9.1**	0	1.45911108469013	775	477	0	3	PLAC9
**CST3.3**	0	1.44378432167295	979	714	0	3	CST3
**ITGB5**	0	1.43087903444419	793	468	0	3	ITGB5
**SEPP1.1**	0	1.41744127002147	0.86	465	0	3	SEPP1
**ID4**	0	1.40840218312557	0.77	425	0	3	ID4
**CLEC11A**	0	1.39606509747882	747	443	0	3	CLEC11A
**CALD1.2**	0	1.39416945818789	993	654	0	3	CALD1
**SLIT3.2**	0	1.39103185572183	817	397	0	3	SLIT3
**C11orf96.1**	0	1.3800030744113	899	647	0	3	C11orf96
**PRRX2.1**	0	1.35351332075198	0.78	0.44	0	3	PRRX2
**PRSS12**	0	1.33586436724903	787	444	0	3	PRSS12
**FRMD6.1**	0	1.32396948384412	792	0.36	0	3	FRMD6
**PLEKHA5.1**	0	1.30088438852931	775	467	0	3	PLEKHA5
**CALU.2**	0	1.30025123743195	889	599	0	3	CALU
**LGALS3BP.1**	0	1.29662336759813	792	517	0	3	LGALS3BP
**CD248**	0	1.29557630891481	748	0.41	0	3	CD248
**LRRC15**	0	1.27643817996849	748	465	0	3	LRRC15
**LGALS3.2**	0	1.27053691602343	967	676	0	3	LGALS3
**PTGFR.1**	0	1.26055075528612	716	398	0	3	PTGFR
**PDGFRB.1**	0	1.25669915752316	856	537	0	3	PDGFRB
**ISLR.1**	0	1.24884255442054	759	347	0	3	ISLR
**BICC1**	0	1.23305545478915	759	479	0	3	BICC1
**EMILIN1.1**	0	1.21495178805316	751	368	0	3	EMILIN1

**CRISPLD2**	0	1.21160595325866	837	549	0	3	CRISPLD2
**FAM20C**	0	1.20800009408886	792	515	0	3	FAM20C
**PALLD**	0	1.19924517281447	792	501	0	3	PALLD
**SPON1**	0	1.18406247884241	761	488	0	3	SPON1
**DSEL**	0	1.14006951905116	744	0.46	0	3	DSEL
**NEGR1**	0	1.11514258789092	694	296	0	3	NEGR1
**SDC2.2**	0	1.09015030613001	719	338	0	3	SDC2
**STEAP1.1**	0	1.05422748629714	715	394	0	3	STEAP1
**ANK2.1**	0	1.04851007273057	689	281	0	3	ANK2
**MME**	0	1.02859275193107	724	386	0	3	MME
**CYP26B1.1**	0	1.01565309918062	665	291	0	3	CYP26B1
**LOX.2**	0	1.00494401776412	686	346	0	3	LOX
**STMN2**	0	0.890156153677964	596	257	0	3	STMN2
**CES1**	0	0.677089461532573	622	274	0	3	CES1
**PCSK1**	0	0.286160785839324	447	73	0	3	PCSK1
**GAS6.1**	1.3464	1.50730509282208	794	532	2.6928266	3	GAS6
**PRELP.2**	1.2737	1.31681086687696	678	316	2.5474944	3	PRELP
**COL16A1**	2.5972	1.17370756837049	707	0.42	5.1944059	3	COL16A1
**PLA2G2A**	6.4079	1.03855261027611	541	194	1.2815953	3	PLA2G2A
**TMEM176A.1**	4.1483	1.3665007482838	761	489	8.2966579	3	TMEM176A
**RORB**	2.0234	1.22544073465804	715	427	4.0468965	3	RORB
**BASP1.2**	9.6280	1.06092036939747	781	454	1.9256197	3	BASP1
**CHRDL1**	2.0152	0.509673712366733	615	291	4.0304202	3	CHRDL1
**SRPX.2**	8.2545	1.09880266232063	687	376	1.6509195	3	SRPX
**CRABP2.1**	2.0212	1.06971758612395	713	363	4.0425812	3	CRABP2
**FNDC1**	1.7261	0.535998183495822	608	295	3.4522946	3	FNDC1
**CRABP1.1**	1.4896	1.22723690643423	641	288	2.9793758	3	CRABP1
**ECM1.2**	3.0866	0.970235591936771	716	411	6.1733432	3	ECM1
**NTRK2.1**	1.5110	1.40400627336726	661	321	3.0221058	3	NTRK2
**MGP.1**	1.7597	2.38654145855077	823	0.57	3.5194486	3	MGP
**CHI3L2**	1.9037	1.01239518367569	624	303	3.8075923	3	CHI3L2
**CLMP.1**	9.2794	0.867792427580244	635	0.26	1.8558826	3	CLMP

**IL11RA**	4.2128	1.21738893813619	615	0.24	8.4257487	3	IL11RA
**RCN3.1**	7.9108	1.18290414187161	673	375	1.5821680	3	RCN3
**DST.2**	6.0467	0.933316603994488	802	509	1.2093559	3	DST
**COL11A1.2**	3.2765	2.18197385543337	664	0.39	6.5531677	3	COL11A1
**ADAMTS2**	1.1491	1.13156238423635	712	0.46	2.2983203	3	ADAMTS2
**HAPLN1**	5.1203	0.394274726302464	536	193	1.0240634	3	HAPLN1
**PCDH18**	2.8612	0.966441015889696	655	372	5.7225772	3	PCDH18
**DPT**	2.1126	1.25068071634985	654	348	4.2253795	3	DPT
**SGCB.1**	4.3644	0.907511302031224	696	412	8.7288953	3	SGCB
**HTRA3**	7.6548	0.767663477382377	643	362	1.5309718	3	HTRA3
**PID1.1**	1.7666	0.843831071206714	619	0.26	3.5333177	3	PID1
**GLDN**	3.7889	0.671616366984176	589	229	7.5779301	3	GLDN
**CH25H**	1.4519	1.035364014511	623	346	2.9038583	3	CH25H
**CDR1-AS**	1.2730	0.808804495308814	625	307	2.5461683	3	CDR1-AS
**MT1M.2**	6.7817	1.01368181761733	669	0.38	1.3563565	3	MT1M
**FBLN5**	3.0671	0.847555625308034	671	0.4	6.1342047	3	FBLN5
**ENPEP**	1.0457	1.05485854022181	665	0.41	2.0914571	3	ENPEP
**RHOBTB3**	5.1982	0.927744839310454	659	395	1.0396488	3	RHOBTB3
**PDGFRL.1**	5.7864	1.18202244675856	628	327	1.1572945	3	PDGFRL
**NID2**	1.1123	0.410077649721143	614	333	2.2246302	3	NID2
**PTX3**	1.7385	0.639256564218925	551	235	3.4770314	3	PTX3
**WISP2**	2.2809	1.35391234862334	615	337	4.5619062	3	WISP2
**MCTP1.1**	4.5055	-0.717596913535989	202	589	9.0110048	3	MCTP1
**NTRK3**	2.9130	0.348893880133928	519	174	5.8261040	3	NTRK3
**HAS1**	1.1062	0.26502012707473	0.51	211	2.2124128	3	HAS1
**ABCA8**	4.8426	1.20344173079045	558	175	9.6853171	3	ABCA8
**ADGRL4.3**	6.5073	-1.22700197436709	257	0.62	1.3014750	3	ADGRL4
**WFDC2**	7.8362	0.39869171611511	555	239	1.5672502	3	WFDC2
**MT1E**	8.5433	1.11207292469079	693	419	1.7086642	3	MT1E
**LYPD3**	2.8056	0.604530377186154	0.58	288	5.6112883	3	LYPD3
**PODXL**	3.4724	-0.990982367849221	375	645	6.9449310	3	PODXL
**SLC1A3**	4.0658	0.499017610619192	567	241	8.1316558	3	SLC1A3

**SELP.2**	4.1241	-0.812435132953963	221	573	8.2483173	3	SELP
**RP11-536I6.2**	1.6208	0.295838053073895	467	116	3.2417112	3	RP11-536I6.2
**ANPEP.2**	3.4045	0.81344560367612	628	359	6.8091276	3	ANPEP
**ABI3BP**	5.7833	1.21189087468288	632	366	1.1566610	3	ABI3BP
**CHRDL2**	2.7837	0.315609554928165	499	177	5.5674077	3	CHRDL2
**HAS2.1**	3.6922	1.0120248375507	596	294	7.3844580	3	HAS2
**FAT1.2**	3.5178	0.543176892985494	623	361	7.0357383	3	FAT1
**HBEGF**	1.6701	-0.624082157671387	369	661	3.3402911	3	HBEGF
**MFAP2.1**	3.3607	0.91346258589576	606	356	6.7215877	3	MFAP2
**NUPR1.2**	9.5387	0.867674449409416	655	385	1.9077494	3	NUPR1
**SDK1**	2.5142	0.314792529202782	556	305	5.0284090	3	SDK1
**S1PR3**	2.1508	0.54537932338768	533	249	4.3017224	3	S1PR3
**GZMB**	1.3368	-0.742166916766814	149	515	2.6736844	3	GZMB
**TMEM119**	1.2138	0.866424948309425	572	293	2.4276454	3	TMEM119
**KDR.1**	3.0976	-0.554360198120595	0.16	481	6.1953952	3	KDR
**SLC7A2.1**	8.4624	0.269978288420188	557	242	1.6924966	3	SLC7A2
**ADGRF5**	2.1693	-0.791736062816534	0.29	604	4.3386901	3	ADGRF5
**LMO4.1**	1.2569	0.591454379914318	737	474	2.5138223	3	LMO4
**SNAI2**	3.7196	0.773214205976194	562	298	7.4393010	3	SNAI2
**PTN**	1.4938	1.09867037515785	572	313	2.9876648	3	PTN
**ADIRF.3**	3.9844	0.276746580990706	899	645	7.9688649	3	ADIRF
**DCLK1**	4.2561	0.894959745291424	579	318	8.5122406	3	DCLK1
**AOAH**	4.5785	-0.280676164897835	63	455	9.1571556	3	AOAH
**FIBIN**	6.2967	0.347714822722939	459	124	1.2593566	3	FIBIN
**STAB1.1**	3.2566	-0.370540705132116	287	573	6.5132823	3	STAB1
**XCL1**	6.4741	-0.567377511879246	305	568	1.2948274	3	XCL1
**MALL.1**	1.3086	-0.639664977562134	291	0.6	2.6173590	3	MALL
**IGFBP7.3**	3.4249	0.29644523485259	969	714	6.8498039	3	IGFBP7
**FIGF**	4.0974	0.395735032312634	418	79	8.1948446	3	FIGF
**IL3RA.2**	1.0050	-0.734842120080352	306	603	2.0100823	3	IL3RA
**IRF4**	1.1862	-0.283420447641179	115	485	2.3724676	3	IRF4
**ACKR1.2**	1.8522	-2.65277312495486	286	578	3.7045336	3	ACKR1

**MPEG1**	9.9548	-0.307637082632909	123	446	1.99096415	3	MPEG1
**SELL**	4.6353	-0.564939240637291	288	0.57	9.27067980	3	SELL
**PLVAP.1**	4.1812	-1.60152975816905	329	602	8.36241065	3	PLVAP
**PDGFB**	6.0285	-0.320696183583924	216	548	1.20571158	3	PDGFB
**TIMP4**	9.3746	0.459872989628202	509	259	1.87493707	3	TIMP4
**GATA2.1**	2.3771	-0.35256036923687	208	512	4.75431490	3	GATA2
**THBD.2**	4.0373	-0.915957570990005	314	574	8.07460302	3	THBD
**UPP1**	6.3813	-0.568731109971988	308	568	1.27626602	3	UPP1
**F2RL2**	9.7874	0.271678090344813	487	234	1.95749419	3	F2RL2
**CRIP2.2**	1.9958	-0.98633192108636	359	629	3.99164853	3	CRIP2
**IGLV3-21.1**	7.9993	-0.544389116379826	72	369	1.59986660	3	IGLV3-21
**CD200.2**	3.2586	-0.468286440046618	271	547	6.51727191	3	CD200
**ICOS**	1.3872	-0.555121216667735	0.27	553	2.77445407	3	ICOS
**SLCO2A1.1**	1.4261	-1.13365956845348	383	649	2.85227588	3	SLCO2A1
**MMRN2.2**	2.6273	-0.707314998917818	198	466	5.25470080	3	MMRN2
**ADAMTS9.2**	7.7739	-0.694871862626233	0.37	649	1.55478490	3	ADAMTS9
**S1PR1.2**	6.7099	-0.366716159382157	279	551	1.34199062	3	S1PR1
**RHOJ.2**	4.2241	-0.254073358325324	297	578	8.44827064	3	RHOJ
**LTB**	1.9438	-1.3424671919352	217	505	3.88765543	3	LTB
**TYROBP**	1.0090	-0.942835065195476	175	437	2.01800018	3	TYROBP
**PTPRB.1**	1.5105	-0.623944421088743	308	0.56	3.02110582	3	PTPRB
**SOX18.1**	6.3978	-0.953832752550069	195	479	1.27956058	3	SOX18
**GRASP.3**	3.6505	-0.646379192685224	334	595	7.30100690	3	GRASP
**JAML**	2.2298	-0.49640095764879	197	463	4.45971079	3	JAML
**ADAM15.3**	5.4040	-0.814349805772453	294	552	1.08080521	3	ADAM15
**ITGB4.1**	1.2096	-0.539630750051732	197	453	2.41924974	3	ITGB4
**SIX1**	4.5224	0.476646692494513	442	163	9.04493285	3	SIX1
**PRSS35**	1.2070	0.728643093724824	409	83	2.41415225	3	PRSS35
**KRT18**	6.8747	-0.31658028093991	197	0.47	1.37494238	3	KRT18
**OSR1**	4.5173	0.291509202277453	394	101	9.03474325	3	OSR1
**GALNT5**	3.2653	0.414041880900223	431	158	6.53064908	3	GALNT5
**MMRN1.1**	3.6263	-0.421380097621553	164	427	7.25273333	3	MMRN1

**DIO3**	2.6350	0.28077277928937	0.37	55	5.2700738	3	DIO3
**LAMP3.2**	2.2276	-0.251221340452876	102	386	4.4552715	3	LAMP3
**ROBO2**	5.1069	0.488849955744531	426	175	1.0213998	3	ROBO2
**CORIN**	8.1253	0.656067257073471	402	109	1.6250637	3	CORIN
**SGCG**	3.4731	0.369810055111575	353	59	6.9462019	3	SGCG
**CXCL13**	0	3.87827672676983	881	624	0	4	CXCL13
**LUM.1**	0	3.6306328980143	1	651	0	4	LUM
**DCN.1**	0	3.520482823455	1	641	0	4	DCN
**IGFBP2.1**	0	3.32421821090009	984	559	0	4	IGFBP2
**CXCL1.1**	0	3.16822636036882	836	486	0	4	CXCL1
**COL6A3.1**	0	3.12000060736059	999	645	0	4	COL6A3
**COL6A1.1**	0	3.0714077580706	1	632	0	4	COL6A1
**DIO2.1**	0	3.03721748625968	961	535	0	4	DIO2
**WNT5A.1**	0	2.99696599968685	969	542	0	4	WNT5A
**COL5A2.1**	0	2.91501615118586	994	573	0	4	COL5A2
**C1S.1**	0	2.8926337691293	999	601	0	4	C1S
**RARRES2.1**	0	2.85867277117106	0.99	0.62	0	4	RARRES2
**C1R.2**	0	2.84978669336536	999	662	0	4	C1R
**APCDD1**	0	2.77043006821173	963	465	0	4	APCDD1
**COL6A2.3**	0	2.76372158270877	999	621	0	4	COL6A2
**CTSK.1**	0	2.71581342191215	993	549	0	4	CTSK
**THY1.1**	0	2.69833433587374	979	455	0	4	THY1
**AEBP1.1**	0	2.64803287395853	998	645	0	4	AEBP1
**COL12A1**	0	2.61905704228995	973	604	0	4	COL12A1
**FBN1.3**	0	2.54372623904959	0.99	642	0	4	FBN1
**FBLN2.3**	0	2.52246432438469	991	492	0	4	FBLN2
**MMP2.1**	0	2.39102297010095	989	607	0	4	MMP2
**TWIST1.1**	0	2.35018150240919	964	413	0	4	TWIST1
**FMOD.1**	0	2.34984669992629	979	537	0	4	FMOD
**PAPPA.1**	0	2.34074381076849	892	542	0	4	PAPPA
**PCOLCE.1**	0	2.29165458388496	0.99	473	0	4	PCOLCE
**SOD2.1**	0	2.28155931425224	996	716	0	4	SOD2

**GAS1.1**	0	2.26516870704968	887	474	0	4	GAS1
**MXRA5.1**	0	2.2486502220044	952	467	0	4	MXRA5
**COL5A1.1**	0	2.20566223425319	968	488	0	4	COL5A1
**SFRP2.1**	0	2.18341421412757	831	499	0	4	SFRP2
**IGF2.1**	0	2.18294229489308	0.93	522	0	4	IGF2
**FSTL1.3**	0	2.17327503406121	991	692	0	4	FSTL1
**PDGFRA.3**	0	2.17098137500101	953	569	0	4	PDGFRA
**TIMP1.3**	0	2.16399038601771	989	689	0	4	TIMP1
**IGFBP4.4**	0	2.15786529944724	999	704	0	4	IGFBP4
**SERPINF1.3**	0	2.14890347637591	989	526	0	4	SERPINF1
**PRSS12.1**	0	2.12142270732825	0.94	431	0	4	PRSS12
**TWIST2**	0	2.12081316263294	953	544	0	4	TWIST2
**IGFBP5.2**	0	2.09855251844961	956	604	0	4	IGFBP5
**CTHRC1.2**	0	2.09533594713623	931	533	0	4	CTHRC1
**MT2A.2**	0	2.08170975492549	934	634	0	4	MT2A
**FAM20C.1**	0	2.06228946263177	949	502	0	4	FAM20C
**CCDC80.1**	0	2.01136880200532	958	438	0	4	CCDC80
**CEBPB.2**	0	1.97085804944209	0.99	616	0	4	CEBPB
**ADM**	0	1.94156205665201	0.84	539	0	4	ADM
**FST**	0	1.92484466501114	877	0.41	0	4	FST
**CXCL12.1**	0	1.9022965143862	964	0.69	0	4	CXCL12
**NNMT.3**	0	1.89915013781253	995	0.71	0	4	NNMT
**THBS2.2**	0	1.88798811815075	919	539	0	4	THBS2
**PHLDA1**	0	1.84476573790454	963	638	0	4	PHLDA1
**SERPING1.2**	0	1.8280802904497	979	601	0	4	SERPING1
**MASP1.2**	0	1.82676630364363	878	399	0	4	MASP1
**RORB.1**	0	1.8265507643953	873	415	0	4	RORB
**C8orf4**	0	1.80415000352488	0.89	557	0	4	C8orf4
**PRSS23.2**	0	1.7935938782307	985	713	0	4	PRSS23
**PTGDS.2**	0	1.78053442546727	825	531	0	4	PTGDS
**TMEM176B.1**	0	1.77646203302756	921	463	0	4	TMEM176B
**LTBP2.2**	0	1.77292836358715	914	548	0	4	LTBP2

**PRRX1.3**	0	1.77187121615124	976	0.48	0	4	PRRX1
**RND3.3**	0	1.76996618790441	921	529	0	4	RND3
**CEBPD.3**	0	1.75828228141085	984	597	0	4	CEBPD
**FGFR1.2**	0	1.75763729310447	953	563	0	4	FGFR1
**MME.1**	0	1.73872432611497	875	374	0	4	MME
**EGFL6**	0	1.73721628626403	852	553	0	4	EGFL6
**SAA1**	0	1.72622189494167	608	239	0	4	SAA1
**CHI3L2.1**	0	1.71979317881369	0.75	293	0	4	CHI3L2
**FN1.2**	0	1.71516069476603	968	571	0	4	FN1
**FBLN1.3**	0	1.7136178617392	959	586	0	4	FBLN1
**BICC1.1**	0	1.7022012271465	917	466	0	4	BICC1
**ANGPTL2**	0	1.69055224902666	934	638	0	4	ANGPTL2
**CDH11.1**	0	1.68970866620876	938	517	0	4	CDH11
**MRC2.2**	0	1.68879153073239	926	398	0	4	MRC2
**CRABP1.2**	0	1.6860010024486	761	278	0	4	CRABP1
**TNFAIP6.1**	0	1.68372602516939	877	481	0	4	TNFAIP6
**BGN.1**	0	1.68009418254684	926	589	0	4	BGN
**ANTXR1**	0	1.67514363125339	904	492	0	4	ANTXR1
**CALU.3**	0	1.6575729631239	964	594	0	4	CALU
**IL1R1.2**	0	1.6536544137059	945	576	0	4	IL1R1
**PDGFRB.2**	0	1.64335507933852	959	529	0	4	PDGFRB
**STEAP1.2**	0	1.64256952254311	0.86	382	0	4	STEAP1
**F2R**	0	1.64077834510199	894	565	0	4	F2R
**DDR2**	0	1.63286135232128	919	519	0	4	DDR2
**VCAN.1**	0	1.61024169782371	963	634	0	4	VCAN
**TMEM176A.2**	0	1.60328622992774	875	0.48	0	4	TMEM176A
**ADAMTS2.1**	0	1.57690877063307	915	443	0	4	ADAMTS2
**CPE.1**	0	1.57346047590563	0.82	499	0	4	CPE
**GREM1**	0	1.57317904617835	735	427	0	4	GREM1
**EFEMP1.1**	0	1.56051145354561	844	557	0	4	EFEMP1
**SOD3.2**	0	1.55777324111887	958	496	0	4	SOD3
**PRRX2.2**	0	1.55549106179243	897	431	0	4	PRRX2

**LGALS3BP.2**	0	1.55343032455372	928	506	0	4	LGALS3BP
**ITGB5.1**	0	1.55180216850402	892	0.46	0	4	ITGB5
**MEG3.2**	0	1.5270334310633	868	375	0	4	MEG3
**FRMD6.2**	0	1.51900589929232	888	352	0	4	FRMD6
**C11orf96.2**	0	1.50728255714298	945	644	0	4	C11orf96
**CFH.3**	0	1.49790626880091	925	564	0	4	CFH
**EDIL3.2**	0	1.49249536476076	0.9	536	0	4	EDIL3
**PXDN.2**	0	1.48560667125973	928	672	0	4	PXDN
**COL14A1.3**	0	1.47956153462806	954	618	0	4	COL14A1
**STEAP2**	0	1.47840572082026	862	449	0	4	STEAP2
**LTBP1**	0	1.47797526810842	0.91	546	0	4	LTBP1
**NID1**	0	1.47605096427131	916	624	0	4	NID1
**DSEL.1**	0	1.47474480507257	876	0.45	0	4	DSEL
**LRP1.1**	0	1.46775980710282	935	519	0	4	LRP1
**PSD3**	0	1.46307914952395	887	406	0	4	PSD3
**CALD1.3**	0	1.45394731243005	998	654	0	4	CALD1
**SPATS2L.2**	0	1.44935680689348	916	499	0	4	SPATS2L
**BPGM**	0	1.44758350611613	877	486	0	4	BPGM
**RAB31.2**	0	1.44271519511604	915	604	0	4	RAB31
**EMILIN1.2**	0	1.43614657281433	0.86	0.36	0	4	EMILIN1
**TSPAN11**	0	1.42200246071832	832	424	0	4	TSPAN11
**LGALS3.3**	0	1.42152462780897	0.99	674	0	4	LGALS3
**PCSK5.1**	0	1.40573717611915	842	498	0	4	PCSK5
**LOXL2**	0	1.40422763270463	859	466	0	4	LOXL2
**CRABP2.2**	0	1.40156761820607	868	351	0	4	CRABP2
**MXRA8.2**	0	1.39804794199462	881	438	0	4	MXRA8
**SLC39A14.2**	0	1.38897762836586	895	539	0	4	SLC39A14
**SGCB.2**	0	1.38597850836487	871	398	0	4	SGCB
**PLEKHA5.2**	0	1.38348037835383	0.89	457	0	4	PLEKHA5
**CHN1**	0	1.38062489070536	0.89	566	0	4	CHN1
**LPAR1**	0	1.37106423858051	867	441	0	4	LPAR1
**LBP**	0	1.37076519929353	736	468	0	4	LBP

**C10orf10.1**	0	1.36438047623573	741	387	0	4	C10orf10
**MOXD1.1**	0	1.34290233696403	805	364	0	4	MOXD1
**MIR4435-2H(**	0	1.33855893219788	901	521	0	4	MIR4435-2H(
**VCAM1.2**	0	1.33575126669136	892	636	0	4	VCAM1
**MFAP4.1**	0	1.3216217856918	855	398	0	4	MFAP4
**ANGPT1.1**	0	1.30569480781151	799	454	0	4	ANGPT1
**PALLD.1**	0	1.29695591079669	877	494	0	4	PALLD
**APOE**	0	1.29354218939642	873	0.6	0	4	APOE
**LAMA4.2**	0	1.2861074063782	905	544	0	4	LAMA4
**S100A4.1**	0	1.2775225632354	994	731	0	4	S100A4
**TNFRSF12A.1**	0	1.27445832611232	863	599	0	4	TNFRSF12A
**COL4A1.2**	0	1.26864469399133	951	696	0	4	COL4A1
**NTM**	0	1.26089941465237	742	285	0	4	NTM
**CD82**	0	1.25479983757631	896	0.44	0	4	CD82
**PMEPA1**	0	1.25277304164786	871	606	0	4	PMEPA1
**ISLR.2**	0	1.24602927310683	829	341	0	4	ISLR
**PTK7.1**	0	1.2390498653019	875	479	0	4	PTK7
**SCARA5**	0	1.23408924409594	802	506	0	4	SCARA5
**ASPN.1**	0	1.2291575410666	793	452	0	4	ASPN
**MT1E.1**	0	1.20802085958478	781	412	0	4	MT1E
**FKBP10**	0	1.203134983697	821	464	0	4	FKBP10
**EFEMP2**	0	1.19834154820045	814	458	0	4	EFEMP2
**S100A13.3**	0	1.19795764875612	906	514	0	4	S100A13
**FILIP1L.2**	0	1.19560348632325	908	568	0	4	FILIP1L
**ECM1.3**	0	1.19134171795078	845	401	0	4	ECM1
**CYP7B1**	0	1.18576022201421	792	348	0	4	CYP7B1
**HTRA1.3**	0	1.1802228798227	909	0.58	0	4	HTRA1
**C3.1**	0	1.17930417527561	831	384	0	4	C3
**PLXDC1**	0	1.17680640221203	802	423	0	4	PLXDC1
**CP**	0	1.17466725728115	707	435	0	4	CP
**F3.1**	0	1.1736368126491	768	0.48	0	4	F3
**IL34**	0	1.16637701394269	804	413	0	4	IL34

**ITGBL1.2**	0	1.16229828987611	0.83	315	0	4	ITGBL1
**CTSB.2**	0	1.14045723226059	945	606	0	4	CTSB
**HTRA3.1**	0	1.13665526436035	777	351	0	4	HTRA3
**SPON1.1**	0	1.13456651400878	822	483	0	4	SPON1
**GGT5.2**	0	1.13012089954911	854	567	0	4	GGT5
**HNMT**	0	1.11988594631495	757	0.42	0	4	HNMT
**SPON2.1**	0	1.11820729273536	749	408	0	4	SPON2
**STAT1**	0	1.11719037178157	859	559	0	4	STAT1
**AKAP12.2**	0	1.11589734749529	846	566	0	4	AKAP12
**FAP**	0	1.11417774448667	776	345	0	4	FAP
**C2**	0	1.10352067690065	795	477	0	4	C2
**SVIL.1**	0	1.08860114205125	874	539	0	4	SVIL
**PLOD2.1**	0	1.07796248197302	837	464	0	4	PLOD2
**OLFML3.2**	0	1.07706610853791	807	426	0	4	OLFML3
**CD248.1**	0	1.07221982154725	817	405	0	4	CD248
**ROBO1.1**	0	1.06847605922616	809	504	0	4	ROBO1
**CYP26B1.2**	0	1.06431253809301	778	282	0	4	CYP26B1
**SGIP1**	0	1.06162686283598	771	479	0	4	SGIP1
**CSGALNACT**	0	1.06091150045217	842	573	0	4	CSGALNACT
**CPXM1**	0	1.06053460426656	767	491	0	4	CPXM1
**PDPN**	0	1.05513056699092	798	398	0	4	PDPN
**COL16A1.1**	0	1.05213407205129	796	413	0	4	COL16A1
**FAM20A**	0	1.04933554497912	766	364	0	4	FAM20A
**COL4A2.2**	0	1.03899044114702	0.94	0.69	0	4	COL4A2
**CLEC11A.1**	0	1.03674983211575	802	439	0	4	CLEC11A
**PID1.2**	0	1.03227876202228	799	246	0	4	PID1
**EML1.1**	0	1.03170579657319	772	281	0	4	EML1
**CLMP.2**	0	1.02602058200848	796	247	0	4	CLMP
**LAMA2**	0	1.02461849864793	741	416	0	4	LAMA2
**LMO4.2**	0	1.02070123220276	883	462	0	4	LMO4
**RHOBTB3.1**	0	1.01937715194077	761	387	0	4	RHOBTB3
**CRISPLD2.1**	0	1.00890685336362	886	546	0	4	CRISPLD2

**RCN3.2**	0	1.0074823830057	767	367	0	4	RCN3
**DKK3.1**	0	1.00735747017519	809	529	0	4	DKK 3
**OMD.1**	0	1.00567847706757	739	392	0	4	OMD
**ANPEP.3**	0	1.00525370766486	777	347	0	4	ANPEP
**SH3PXD2A.1**	0	1.00101358295546	0.84	543	0	4	SH3PXD2A
**LOXL1.1**	0	0.986816427609534	752	457	0	4	LOXL1
**MMP14**	0	0.983331277168791	788	531	0	4	MMP14
**ITGA8.1**	0	0.979509954837474	769	409	0	4	ITGA8
**COL11A1.3**	0	0.979354085917512	0.72	386	0	4	COL11A1
**TYMP.2**	0	0.97604584140981	962	686	0	4	TYMP
**LRRC15.1**	0	0.961398268916381	805	0.46	0	4	LRRC15
**LSAMP**	0	0.958934909250292	748	349	0	4	LSAMP
**CST3.4**	0	0.947948370985928	989	713	0	4	CST3
**VASN**	0	0.938926726078517	0.76	455	0	4	VASN
**FADS1**	0	0.938817483169922	746	477	0	4	FADS1
**GLUL.2**	0	0.936806662880453	933	583	0	4	GLUL
**FAM65C**	0	0.928509556737565	723	255	0	4	FAM65C
**GLIS3.2**	0	0.92325455271093	787	443	0	4	GLIS3
**SCPEP1.1**	0	0.916561222200541	827	397	0	4	SCPEP1
**SEMA5A.1**	0	0.912215456242304	777	306	0	4	SEMA5A
**TNS3.1**	0	0.910041347576417	754	481	0	4	TNS3
**JDP2.2**	0	0.908991247672534	831	532	0	4	JDP2
**PTGFR.2**	0	0.90524997890043	771	394	0	4	PTGFR
**DPT.1**	0	0.901373112454276	722	342	0	4	DPT
**HMCN1.1**	0	0.888593183208852	744	0.45	0	4	HMCN1
**SSPN.1**	0	0.887849504290093	797	484	0	4	SSPN
**COL27A1**	0	0.885773948095097	772	0.41	0	4	COL27A1
**DST.3**	0	0.865277859993317	875	503	0	4	DST
**MMP19**	0	0.86386994749209	804	513	0	4	MMP19
**NID2.1**	0	0.851794456367616	766	321	0	4	NID2
**FBLN5.1**	0	0.850730745257987	788	391	0	4	FBLN5
**LINC00152**	0	0.842475987180685	878	0.58	0	4	LINC00152

**ELOVL2**	0	0.839460022648758	755	434	0	4	ELOVL2
**SDK1.1**	0	0.829257056805537	714	292	0	4	SDK1
**NRCAM**	0	0.829038745485938	688	313	0	4	NRCAM
**KYNU.1**	0	0.828370242519707	765	414	0	4	KYNU
**SCUBE2**	0	0.825688178864117	728	285	0	4	SCUBE2
**KIAA1217.2**	0	0.823512972120383	756	427	0	4	KIAA1217
**VGLL3**	0	0.817008952979198	765	473	0	4	VGLL3
**PI15**	0	0.784906416580647	625	172	0	4	PI15
**SDC2.3**	0	0.76155885901632	744	336	0	4	SDC2
**BASP1.3**	0	0.746395507009624	834	0.45	0	4	BASP1
**NEGR1.1**	0	0.735870255567576	736	293	0	4	NEGR1
**SLC6A6.1**	0	0.726184668722103	847	559	0	4	SLC6A6
**GALNT15**	0	0.684576903590078	749	381	0	4	GALNT15
**WNT2**	0	0.664643396825644	686	296	0	4	WNT2
**UBD**	0	0.650103592748239	708	319	0	4	UBD
**SLIT3.3**	0	0.623047911953487	781	401	0	4	SLIT3
**EPSTI1.1**	0	0.609251936411166	0.75	406	0	4	EPSTI1
**SLC1A3.1**	0	0.577564796988209	651	235	0	4	SLC1A3
**CFD.2**	0	0.573771413458102	883	554	0	4	CFD
**ITGA11**	0	0.543751481369961	0.68	305	0	4	ITGA11
**TPM1.2**	0	0.537316968164049	0.87	476	0	4	TPM1
**CEMIP**	0	0.492771435411537	653	319	0	4	CEMIP
**RP11-125O1**	0	0.49103853893994	668	229	0	4	RP11-125O1
**F2RL2.1**	0	0.468093225734635	617	223	0	4	F2RL2
**IDO1**	0	0.44126318126257	625	268	0	4	IDO1
**WNT4**	0	0.438769065431406	671	277	0	4	WNT4
**TPM2.1**	0	0.437641865922302	878	381	0	4	TPM2
**APOD.1**	0	0.343685476747687	809	475	0	4	APOD
**NTRK3.1**	0	0.277645006555316	592	168	0	4	NTRK3
**MYL9.2**	0	0.253342083807523	885	0.55	0	4	MYL9
**GNAI1.1**	7.4816	0.868728283606799	683	278	1.4963293	4	GNAI1
**GPNMB**	3.2458	0.859088684486355	796	543	6.4917217	4	GPNMB

**ADRA2A.1**	3.5695	0.790161424585394	664	287	7.1391372	4	ADRA2A
**CTSD.2**	4.9925	0.875894692650229	887	511	9.9850213	4	CTSD
**MMP1**	4.3048	0.701047643534669	502	111	8.6096281	4	MMP1
**CDR1-AS.1**	5.2732	0.621846172115028	0.67	304	1.0546470	4	CDR1-AS
**MMP3**	5.7180	0.728421683429588	536	135	1.1436025	4	MMP3
**WFDC2.1**	8.9346	1.07506617826033	645	232	1.7869238	4	WFDC2
**CERCAM**	9.4010	0.868744520077845	721	404	1.8802125	4	CERCAM
**MFAP2.2**	3.1079	0.917304657009871	708	348	6.2158308	4	MFAP2
**CXCL14.2**	9.0452	0.890950922402291	742	383	1.8090420	4	CXCL14
**PAPSS2**	3.8308	0.747119649817628	0.7	404	7.6617917	4	PAPSS2
**OLFM2**	3.4473	1.0657871239272	708	407	6.8947311	4	OLFM2
**IGFBP6.2**	9.1170	1.12323861576803	738	425	1.8234055	4	IGFBP6
**GFPT2**	1.2124	0.350928453772831	637	327	2.4248603	4	GFPT2
**ZNF503.1**	5.1714	0.901943922431765	752	433	1.0342976	4	ZNF503
**COL5A3.2**	1.3611	1.00059499925167	713	0.44	2.7223528	4	COL5A3
**TPST1.1**	1.4953	0.82823902854468	711	408	2.9906169	4	TPST1
**CES1.1**	5.0612	0.259279206363967	621	275	1.0122411	4	CES1
**TMEM158**	1.6956	0.622969526952293	664	396	3.3912990	4	TMEM158
**NBL1**	6.7587	0.961935787309428	802	541	1.3517434	4	NBL1
**COL7A1**	3.6279	0.819675791203036	661	339	7.2559793	4	COL7A1
**LOX.3**	1.0684	0.68369756197787	685	347	2.1368087	4	LOX
**CTSL**	3.3859	0.77466118092675	807	556	6.7719519	4	CTSL
**UNC5B.2**	2.4225	0.88530541806513	714	421	4.8450295	4	UNC5B
**ANK2.2**	1.3887	0.611657139512905	647	285	2.7774423	4	ANK2
**SRPX.3**	1.4459	0.591755455107262	693	376	2.8918059	4	SRPX
**ABI3BP.1**	4.4308	1.07378993149978	676	362	8.8617964	4	ABI3BP
**TNC.2**	7.6390	1.29044411629127	686	396	1.5278006	4	TNC
**FAT1.3**	4.7170	0.621042463823535	696	355	9.4340451	4	FAT1
**TMEM45A.1**	4.2113	0.730027636267314	662	297	8.4226929	4	TMEM45A
**ALPL.1**	4.3103	0.59746044253708	749	487	8.6206803	4	ALPL
**COL8A1**	2.1041	0.878649627327675	694	438	4.2082506	4	COL8A1
**ADAM12**	8.3046	0.572710671388486	658	352	1.6609356	4	ADAM12

**NRG2**	2.6514	0.643364305864275	0.65	0.35	5.3029675	4	NRG2
**CRTAC1**	7.5870	0.715859951536334	645	358	1.5174044	4	CRTAC1
**FNDC1.1**	4.9434	0.281366971661222	608	295	9.8868283	4	FNDC1
**TRPA1**	1.4442	0.573307126551818	589	209	2.8884828	4	TRPA1
**CA12**	8.6408	0.511349916537626	569	167	1.7281763	4	CA12
**TBX3**	2.3733	0.991843707229387	699	439	4.7467241	4	TBX3
**MPEG1.1**	1.6763	-0.307471750430166	94	448	3.3526982	4	MPEG1
**SAA2**	2.3787	0.533883653228203	555	238	4.7574642	4	SAA2
**PLPP1.1**	3.3703	0.653818195949046	773	517	6.7406620	4	PLPP1
**CD24**	7.0202	0.652556956073185	661	324	1.4040513	4	CD24
**WISP1**	7.4525	0.555123404747247	584	192	1.4905037	4	WISP1
**S1PR3.1**	5.9752	0.255360984481236	587	245	1.1950467	4	S1PR3
**AKR1C1**	1.3170	0.3700596264731	573	224	2.6340987	4	AKR1C1
**INHBA**	8.3916	1.09138504657039	682	404	1.6783230	4	INHBA
**ENPEP.1**	1.0113	0.717654728384232	678	0.41	2.0227958	4	ENPEP
**SEMA6D**	1.0178	0.410209558452359	594	272	2.0356150	4	SEMA6D
**CCDC71L**	1.2947	0.682070240685099	718	447	2.5894686	4	CCDC71L
**ENAH.1**	6.7938	0.695055648199793	755	0.45	1.3587766	4	ENAH
**FGF7.1**	2.7779	0.321989699495954	689	378	5.5559364	4	FGF7
**RHOB.2**	3.0483	0.633530526815299	882	607	6.0966497	4	RHOB
**SEPP1.2**	5.8645	0.405697772827045	803	0.47	1.1729070	4	SEPP1
**SIX1.1**	1.7152	0.573608677769806	574	153	3.4304009	4	SIX1
**ADGRF5.1**	2.2045	-0.788536918179506	241	608	4.4090162	4	ADGRF5
**PDLIM4.1**	4.0317	0.498751579576674	705	429	8.0635716	4	PDLIM4
**CCND1.1**	2.8859	0.522871476817666	0.8	529	5.7718542	4	CCND1
**EPDR1**	3.1538	0.703536094191763	631	315	6.3076918	4	EPDR1
**IFI6.2**	2.4568	0.704131874561073	764	429	4.9137535	4	IFI6
**PERP**	1.3243	0.517456353621116	796	446	2.6487145	4	PERP
**NUPR1.3**	1.5645	0.620499773856537	714	0.38	3.1291179	4	NUPR1
**PCDH18.1**	3.3542	0.776001203731956	655	372	6.7084177	4	PCDH18
**NCOA7.3**	9.1488	0.54136530749291	882	606	1.8297673	4	NCOA7
**RP1-78O14.1**	7.2699	0.543750916471266	559	192	1.4539844	4	RP1-78O14.1

**EMID1**	2.6116	0.511052526562506	628	362	5.2232952		4	EMID1
**SNAI2.1**	4.8364	0.715604440042071	615	294	9.6729073		4	SNAI2
**HSD11B1**	1.9029	0.569092397719605	563	195	3.8059450		4	HSD11B1
**GLDN.1**	7.7211	0.30610805312145	572	0.23	1.5442282		4	GLDN
**TSC22D1.2**	7.6626	0.743939513850292	911	639	1.5325254		4	TSC22D1
**TIMP4.1**	5.8394	0.539812248299917	579	253	1.1678911		4	TIMP4
**TXNIP**	1.1033	-1.66235060466909	417	0.69	2.2066867		4	TXNIP
**FRZB.1**	6.5600	0.581326490407794	682	384	1.3120063		4	FRZB
**A2M.3**	1.5389	-2.38819300983825	388	0.68	3.0778455		4	A2M
**S1PR1.3**	6.5843	-0.497864096081176	219	556	1.3168751		4	S1PR1
**LIFR.2**	2.6029	0.302214627990344	703	0.42	5.2059141		4	LIFR
**CRTAM**	2.1332	-0.72014161372179	284	0.55	4.2664338		4	CRTAM
**WISP2.1**	1.6143	0.5701827943366	599	339	3.2287854		4	WISP2
**PTPRB.2**	2.2323	-0.629088399127406	274	563	4.4646080		4	PTPRB
**ALDH1A3.1**	1.7292	0.285505780910152	595	303	3.4585003		4	ALDH1A3
**MCTP1.2**	2.0705	-0.729865254742465	261	583	4.1411073		4	MCTP1
**DCLK1.1**	1.0030	0.389403740974792	0.6	317	2.0061232		4	DCLK1
**COL6A5**	6.6509	0.359432200861429	472	91	1.3301931		4	COL6A5
**TNFRSF11B**	2.1076	0.775822383844433	547	215	4.2153468		4	TNFRSF11B
**LRRN3**	1.1940	0.501961496284031	532	214	2.3881414		4	LRRN3
**GALNT5.1**	5.5979	0.433613599592449	518	151	1.1195943		4	GALNT5
**PTN.1**	1.9921	0.605701186941442	587	312	3.9842372		4	PTN
**SLAMF7**	1.3855	-0.388633480568188	302	568	2.7710849		4	SLAMF7
**CTSC**	1.1634	0.477704404562038	794	0.53	2.3269306		4	CTSC
**TXN.2**	1.4339	0.366618255678888	938	662	2.8678511		4	TXN
**FMO3**	4.1691	0.78366964830789	572	298	8.3383193		4	FMO3
**RND1.1**	2.7536	-0.330684716941697	292	547	5.5073996		4	RND1
**PALMD.1**	1.0024	-1.06101486730477	305	568	2.0049009		4	PALMD
**IRF4.1**	2.8386	-0.292528075280939	171	0.48	5.6773810		4	IRF4
**TMEM119.1**	2.4867	0.535094337216241	548	295	4.9735049		4	TMEM119
**CD9.3**	2.6771	0.31973870208749	932	0.64	5.3543646		4	CD9
**CLTB.1**	4.5883	0.284610119692719	799	541	9.1767110		4	CLTB

**GRASP.4**	1.3009	-0.71132164181723	0.33	595	2.6019685	4	GRASP
**LAMP3.3**	2.5202	-0.257135732765329	82	387	5.0404620	4	LAMP3
**HAS2.2**	5.3108	0.439229070427282	549	298	1.0621792	4	HAS2
**HBA1**	2.2149	-0.365376971333212	206	458	4.4299587	4	HBA1
**PTP4A3**	2.8984	-0.534123225675645	249	0.5	5.7968976	4	PTP4A3
**ABCA6**	6.0449	0.551846981677212	503	0.19	1.2089953	4	ABCA6
**IGFBP7.4**	4.5345	-0.332724179925831	971	714	9.0691250	4	IGFBP7
**IGLV3-25**	3.2873	-0.542302371769698	11	262	6.5746347	4	IGLV3-25
**CORIN.1**	4.2426	0.266521160451306	425	108	8.4852526	4	CORIN
**ROBO2.1**	3.1784	0.269884075904617	445	174	6.3569236	4	ROBO2
**EDNRA**	3.8919	0.396895415368408	461	195	7.7838761	4	EDNRA
**PODXL.1**	0	2.94472792767367	961	602	0	5	PODXL
**A2M.4**	0	2.73204611286595	991	636	0	5	A2M
**IFI27.3**	0	2.72159147338039	983	579	0	5	IFI27
**INSR.2**	0	2.64881291529066	901	549	0	5	INSR
**SLC9A3R2**	0	2.52450074440677	875	0.4	0	5	SLC9A3R2
**CLDN5.2**	0	2.50239909401304	834	419	0	5	CLDN5
**FLT1.2**	0	2.49478627795451	915	0.47	0	5	FLT1
**RAMP2.3**	0	2.43046336593437	952	454	0	5	RAMP2
**TIMP3.1**	0	2.42388923885549	958	643	0	5	TIMP3
**STC1**	0	2.34520912368715	0.85	599	0	5	STC1
**EFNB2**	0	2.29866952247345	831	438	0	5	EFNB2
**CLEC14A.1**	0	2.14350412606132	891	508	0	5	CLEC14A
**ADGRF5.2**	0	2.13341693094432	913	558	0	5	ADGRF5
**SOX18.2**	0	2.04198130259049	839	432	0	5	SOX18
**CRIP2.3**	0	2.01977819657435	931	587	0	5	CRIP2
**TM4SF1.3**	0	1.99151737171982	957	597	0	5	TM4SF1
**HEG1.3**	0	1.97771752590881	944	591	0	5	HEG1
**APOLD1.1**	0	1.94860361737236	908	625	0	5	APOLD1
**PLVAP.2**	0	1.93442590175188	877	561	0	5	PLVAP
**GNG11.3**	0	1.91589072567703	937	0.56	0	5	GNG11
**HYAL2.2**	0	1.91510349245646	899	476	0	5	HYAL2

**CDH5.1**	0	1.88554808407568	924	0.59	0	5	CDH5
**AQP1.3**	0	1.86889612070859	976	666	0	5	AQP1
**CD93.1**	0	1.85525677069732	954	651	0	5	CD93
**PALMD.2**	0	1.82929456964361	891	526	0	5	PALMD
**KDR.2**	0	1.80971645143802	834	432	0	5	KDR
**ESAM.2**	0	1.76414330161638	898	506	0	5	ESAM
**EPAS1.3**	0	1.74030089200683	969	698	0	5	EPAS1
**PECAM1.3**	0	1.73877365842669	967	655	0	5	PECAM1
**ICAM2.1**	0	1.73867054739712	865	515	0	5	ICAM2
**HSPG2.3**	0	1.69032111500308	956	672	0	5	HSPG2
**NPDC1.3**	0	1.67153377049192	923	537	0	5	NPDC1
**PLCB1**	0	1.64513232104975	818	0.32	0	5	PLCB1
**CAV1.3**	0	1.63016791486954	932	634	0	5	CAV1
**RNASE1.2**	0	1.62342270818123	882	557	0	5	RNASE1
**JAG2.1**	0	1.61541056902487	811	343	0	5	JAG2
**ADGRL4.4**	0	1.60756441872159	896	572	0	5	ADGRL4
**NES.2**	0	1.59697054033594	841	336	0	5	NES
**SEMA3G**	0	1.59077326351019	754	134	0	5	SEMA3G
**GSN.3**	0	1.57984287330445	956	678	0	5	GSN
**FAM107A.3**	0	1.57926873145143	858	443	0	5	FAM107A
**ENG.3**	0	1.55868739651134	916	582	0	5	ENG
**MAST4**	0	1.51753741852463	859	486	0	5	MAST4
**SPARCL1.3**	0	1.41671770809214	968	624	0	5	SPARCL1
**NFIB.3**	0	1.36078974190115	899	0.58	0	5	NFIB
**CCDC3.2**	0	1.35807474218795	848	443	0	5	CCDC3
**GJA5**	0	1.23260053955537	689	258	0	5	GJA5
**PLXNA2.3**	0	1.21447934704873	823	419	0	5	PLXNA2
**MPZL2.2**	1.2207	0.890265660476247	815	0.41	2.4414522	5	MPZL2
**ECSCR.1.2**	1.2771	1.41992827309015	879	555	2.5543094	5	ECSCR.1
**THBD.3**	1.1702	1.27675074764462	868	533	2.3404437	5	THBD
**EGFL7.2**	6.8553	1.56635691010327	821	405	1.3710736	5	EGFL7
**ID1.1**	1.4862	1.91822800415858	859	556	2.9724692	5	ID1

**MTUS1.1**	1.9077	1.64415123033454	833	488	3.8154384	5	MTUS1
**ITGB4.2**	1.0208	1.04117238994048	812	408	2.0416839	5	ITGB4
**ADGRG1.1**	1.8125	0.916490761014672	822	547	3.6250658	5	ADGRG1
**IGFBP3**	9.4197	2.45413429537401	764	0.46	1.8839488	5	IGFBP3
**PCDH17.2**	1.3363	1.43530022710448	847	532	2.6727336	5	PCDH17
**SPRY1.1**	1.8747	1.59775055081839	892	576	3.7495775	5	SPRY1
**KCNN3**	3.0192	1.33338144512475	0.8	506	6.0384807	5	KCNN3
**ADAM15.4**	6.8706	1.58955297422619	845	512	1.3741395	5	ADAM15
**CD34.2**	6.1381	1.56544922781735	824	492	1.2276287	5	CD34
**EMCN.2**	1.0887	1.41658293833689	845	505	2.1775159	5	EMCN
**THSD7A.2**	1.8026	1.72638779361737	785	393	3.6052903	5	THSD7A
**ALPL.2**	1.8036	1.32217243324934	0.8	488	3.6073810	5	ALPL
**LIMS2**	1.5332	1.15780481531181	812	519	3.0664916	5	LIMS2
**SGK1.1**	3.4612	1.45380285970032	902	637	6.9225710	5	SGK1
**CYYR1.1**	1.2793	1.50702569193305	786	395	2.5586984	5	CYYR1
**TNXB.1**	1.9176	1.03709946426145	815	514	3.8353402	5	TNXB
**ITGA6.3**	1.7715	1.49155690677529	892	604	3.5431480	5	ITGA6
**MALL.2**	1.9226	1.03335843565957	0.84	558	3.8452961	5	MALL
**IL3RA.3**	8.4117	1.19591873446625	845	563	1.6823407	5	IL3RA
**ARHGAP29.1**	4.9531	1.41676058136611	834	511	9.9063739	5	ARHGAP29
**MCAM.3**	3.1603	0.99517341522629	0.88	581	6.3206196	5	MCAM
**FLNB.1**	6.4966	1.16814964617037	847	0.57	1.2993335	5	FLNB
**EMP2.1**	3.3831	1.37471529571644	833	507	6.7662700	5	EMP2
**S1PR1.4**	1.7255	1.13427970313082	801	512	3.4511721	5	S1PR1
**CRYBG3.1**	1.0140	1.51635660261851	811	536	2.0281527	5	CRYBG3
**MMRN2.3**	1.9655	1.21406548509017	797	422	3.9310290	5	MMRN2
**BMPR2.3**	2.7189	1.21861618223154	872	604	5.4379024	5	BMPR2
**PTPRB.3**	1.6413	1.57237860925473	781	525	3.2827691	5	PTPRB
**CD9.4**	2.9243	1.21905458579085	923	646	5.8486151	5	CD9
**ADAMTS2.2**	7.9682	-0.454482887505188	65	506	1.5936435	5	ADAMTS2
**NOTCH4**	6.0019	1.59916390923742	725	381	1.2003878	5	NOTCH4
**JAG1.1**	1.8659	1.33013515267453	831	538	3.7318060	5	JAG1

**BCAM.1**	2.6250	1.30176475555104	815	501	5.2500125		5	BCAM
**DLC1.3**	3.0916	0.94446672055941	875	614	6.1832521		5	DLC1
**MECOM.2**	1.0528	1.45778230856395	715	385	2.1057088		5	MECOM
**LIMCH1.1**	4.4371	1.11415318636233	0.73	394	8.8742657		5	LIMCH1
**MAP1B.2**	6.8499	0.87362582183283	849	507	1.3699880		5	MAP1B
**VWF.2**	1.6141	0.501732018333037	875	561	3.2283108		5	VWF
**CRIM1.3**	3.0659	1.00838630646664	882	631	6.1319825		5	CRIM1
**PDLIM1.3**	2.2084	0.988592540035258	916	617	4.4168091		5	PDLIM1
**PIK3R3.1**	9.2750	1.21491538148823	756	482	1.8550096		5	PIK3R3
**TEK.2**	2.4421	0.917762911621276	749	424	4.8843242		5	TEK
**GJA1.2**	3.2982	0.942649829984021	805	453	6.5964736		5	GJA1
**NOS3.1**	3.4996	0.786737907281224	685	0.32	6.9992209		5	NOS3
**WWTR1.3**	2.0780	1.08768867216965	836	573	4.1561332		5	WWTR1
**SOD3.3**	6.8636	-1.60031501424098	177	554	1.3727394		5	SOD3
**MYCT1**	8.9989	1.00448780581483	0.74	461	1.7997949		5	MYCT1
**GRB10.1**	1.0671	0.977659535058064	744	482	2.1343300		5	GRB10
**PKP4.2**	1.7799	0.597426837244526	808	551	3.5598374		5	PKP4
**ZNF331.1**	5.1621	-1.41238796787045	393	712	1.0324232		5	ZNF331
**FAM167B.3**	9.1494	0.647417216518904	746	449	1.8298814		5	FAM167B
**LMO2.2**	2.3338	1.25215895561525	734	393	4.6676697		5	LMO2
**IL1R1.3**	4.8068	-0.809009529814468	285	625	9.6137133		5	IL1R1
**RAPGEF5.2**	1.3480	1.1001032995474	688	0.3	2.6960000		5	RAPGEF5
**RCAN1.1**	4.9779	0.886981453262084	0.77	514	9.9559020		5	RCAN1
**ACOT7**	2.1791	0.487519788615895	683	388	4.3583171		5	ACOT7
**GAS1.2**	5.0868	-0.870112305754116	211	525	1.0173660		5	GAS1
**LRRC15.2**	3.4602	-0.338610328404736	0.06	514	6.9204118		5	LRRC15
**FAM89A**	3.1706	0.904276626659435	707	454	6.3413642		5	FAM89A
**RAMP3.3**	1.1045	0.857406070876304	782	473	2.2091924		5	RAMP3
**COL14A1.4**	1.2295	-1.60519855039105	391	0.66	2.4591002		5	COL14A1
**SPRY4.2**	1.9600	0.663256169720603	725	0.44	3.9200457		5	SPRY4
**POU2F2**	2.9731	-0.647153431880582	174	495	5.9462075		5	POU2F2
**ARL 15**	4.3010	1.14958184037147	637	313	8.6021352		5	ARL 15

**RBP7.1**	1.7572	1.22782230137845	628	275	3.5145052	5	RBP7
**SDPR.1**	8.6095	0.817619031672548	701	429	1.7219048	5	SDPR
**PPFIBP1.3**	4.0234	0.86674363960051	732	449	8.0469248	5	PPFIBP1
**S100A16.3**	1.9128	0.893466732246514	772	495	3.8257990	5	S100A16
**STEAP2.1**	1.7466	-0.370911551375727	183	0.5	3.4932544	5	STEAP2
**VWA1.1**	5.3745	1.05261722558633	725	446	1.0749109	5	VWA1
**ASS1**	1.0212	0.83485222627012	616	262	2.0424145	5	ASS1
**JUP.2**	4.2829	0.427507345023419	653	359	8.5659063	5	JUP
**LTBP2.3**	2.3300	-0.580893358965276	315	593	4.6601725	5	LTBP2
**ROBO4.3**	1.2231	0.933383914480294	698	425	2.4463914	5	ROBO4
**NUAK1.3**	5.6693	0.824874356121143	708	449	1.1338616	5	NUAK1
**CXorf36.1**	9.2202	0.618841839789439	705	442	1.8440553	5	CXorf36
**UACA.1**	7.8796	1.13070859346323	705	452	1.5759239	5	UACA
**GPIHBP1**	7.0999	0.932932884420031	579	224	1.4199899	5	GPIHBP1
**COL13A1**	1.1094	0.339585737061228	575	311	2.2188496	5	COL13A1
**ERG.2**	1.7582	0.877268213110004	689	426	3.5165399	5	ERG
**KCNJ2**	3.0693	0.74382173061916	563	0.2	6.1386377	5	KCNJ2
**ELOVL7.3**	5.1399	0.420845039259062	646	318	1.0279896	5	ELOVL7
**SSUH2**	1.7902	0.401755355615971	454	0.05	3.5805045	5	SSUH2
**ANXA3**	1.8504	0.257202625040883	491	164	3.7008074	5	ANXA3
**TBX1.1**	3.6815	0.308845735883752	625	342	7.3630584	5	TBX1
**BMX**	1.1071	0.647773189756206	579	238	2.2142784	5	BMX
**GPNMB.1**	8.7579	-0.642105777640702	0.32	578	1.7515971	5	GPNMB
**CARHSP1.3**	4.4166	0.619239462542948	0.78	517	8.8333092	5	CARHSP1
**GJA4**	9.2528	1.85767164994552	604	295	1.8505773	5	GJA4
**INHBA.1**	2.8167	-0.344019093128321	73	448	5.6335599	5	INHBA
**CRABP2.3**	1.1527	-0.625291270913921	147	406	2.3055463	5	CRABP2
**ACE**	9.2091	1.0875520071425	587	294	1.8418365	5	ACE
**CYBB**	4.4564	-0.400228997122752	83	406	8.9128711	5	CYBB
**FILIP1.1**	7.4739	0.910422315863516	596	328	1.4947973	5	FILIP1
**ISLR.3**	1.5985	-0.388747853485202	138	394	3.1971058	5	ISLR
**F13A1**	1.6286	-0.310702075958081	122	436	3.2573133	5	F13A1

**SOX17.1**	9.5725	1.24370088133995	586	308	1.9145092	5	SOX17
**XCL2**	9.5048	-0.437488700181785	145	428	1.9009775	5	XCL2
**NEBL**	1.5161	0.576497564370703	505	172	3.0323985	5	NEBL
**GREM1.1**	1.1243	-0.30194290883226	204	467	2.2486615	5	GREM1
**NEGR1.2**	8.1822	-0.265022037989386	26	347	1.6364408	5	NEGR1
**PCDH18.2**	1.3942	-0.278430010875998	131	411	2.7885560	5	PCDH18
**C12orf75**	9.0442	-0.387294585330578	155	413	1.8088567	5	C12orf75
**AIF1L**	3.8579	0.718505180207409	541	285	7.7158878	5	AIF1L
**FCN3**	5.1820	0.651175119368387	0.49	173	1.0364073	5	FCN3
**PRELP.3**	2.1731	-0.310001440149367	101	0.36	4.3463678	5	PRELP
**TMEM178A**	6.7724	0.613632705404242	429	113	1.3544934	5	TMEM178A
**CD83**	0	2.94313849081227	947	584	0	6	CD83
**MS4A1.1**	0	2.80137368345029	887	339	0	6	MS4A1
**CD79A.1**	0	2.54371235525963	869	466	0	6	CD79A
**HLA-DRA.1**	0	2.13137581129091	982	677	0	6	HLA-DRA
**HLA-DQB1.1**	0	1.73361025221436	887	552	0	6	HLA-DQB1
**HLA-DPB1.2**	0	1.67088449894117	955	583	0	6	HLA-DPB1
**HLA-DPA1.1**	0	1.6058662503909	954	628	0	6	HLA-DPA1
**NFKBID**	0	1.33466313665285	909	582	0	6	NFKBID
**RAB11FIP1**	0	1.308017804705	909	562	0	6	RAB11FIP1
**RP1-313I6.12**	0	0.618762817485221	857	515	0	6	RP1-313I6.12
**WDFY4**	0	0.58617649300832	832	354	0	6	WDFY4
**RASSF6**	0	0.288408252714244	788	317	0	6	RASSF6
**MPEG1.2**	0	0.285953479371398	806	397	0	6	MPEG1
**BTNL9**	0	0.266456912719404	813	248	0	6	BTNL9
**NEGR1.3**	0	-0.262714882428216	79	343	0	6	NEGR1
**ABI3BP.2**	0	-0.378435327780214	41	408	0	6	ABI3BP
**MYCT1.1**	0	-0.399382932249485	75	504	0	6	MYCT1
**BPGM.1**	0	-0.418136604755803	121	541	0	6	BPGM
**NRP2**	0	-0.512323012487602	109	544	0	6	NRP2
**CXorf36.2**	0	-0.53519606361098	118	0.48	0	6	CXorf36
**FAM107A.4**	0	-0.601428281754452	43	495	0	6	FAM107A

**PRSS12.2**	0	-0.692375540979552	137	491	0	6	PRSS12
**CPA3**	0	-0.70944936062824	824	345	0	6	CPA3
**CSGALNACT**	0	-0.754635373387342	232	616	0	6	CSGALNACT
**CYP1B1.2**	0	-0.845172876867063	116	593	0	6	CYP1B1
**MYLK.1**	0	-0.905216004073254	193	571	0	6	MYLK
**JAG1.2**	0	-0.922380534214584	99	585	0	6	JAG1
**DST.4**	0	-1.02057485574916	0.1	559	0	6	DST
**SERPING1.3**	0	-1.43445562352451	146	0.66	0	6	SERPING1
**TPM1.3**	0	-1.45697867336428	81	533	0	6	TPM1
**NR2F2.2**	0	-1.54063615844736	111	563	0	6	NR2F2
**ENG.4**	0	-1.58791935836801	209	627	0	6	ENG
**TIMP3.2**	0	-1.81874393113181	273	687	0	6	TIMP3
**CCL2.1**	0	-1.90881526932699	283	665	0	6	CCL2
**EPAS1.4**	0	-1.96225136677294	0.34	738	0	6	EPAS1
**LGALS3.4**	0	-2.02348122459992	214	728	0	6	LGALS3
**ANXA1**	0	-2.20051319158044	424	836	0	6	ANXA1
**NNMT.4**	0	-2.35859169668905	348	756	0	6	NNMT
**RP11-1143G9**	6.5796	-0.286755581193965	0.17	467	1.3159333	6	RP11-1143G9
**IGFBP4.5**	1.4207	-2.76788786657325	382	748	2.8415298	6	IGFBP4
**VWF.3**	1.3337	-1.86418318747713	251	601	2.6674214	6	VWF
**PALLD.2**	1.9403	-0.727619923098556	169	545	3.8806328	6	PALLD
**HLA-DQA1**	1.7063	1.72680688773574	848	421	3.4127295	6	HLA-DQA1
**HLA-DRB1.2**	6.5769	1.17718414468753	958	657	1.3153924	6	HLA-DRB1
**SPNS2.2**	1.0581	-0.512522104713313	134	0.51	2.1163651	6	SPNS2
**EMP2.2**	2.2945	-0.870633007516195	162	0.55	4.5891347	6	EMP2
**CEBPD.4**	2.5451	-2.34661671468728	223	652	5.0903065	6	CEBPD
**CALCRL.2**	3.7314	-0.842589160715543	0.12	488	7.4629013	6	CALCRL
**IGFBP7.5**	8.5251	-3.42058988537887	365	756	1.7050215	6	IGFBP7
**EREG**	2.2305	-0.721637544639219	818	0.48	4.4611217	6	EREG
**CTSB.3**	2.8710	-1.25466319985753	236	656	5.7420760	6	CTSB
**EGR2**	1.2897	0.269751864743836	857	0.56	2.5794122	6	EGR2
**PALMD.3**	1.3814	-1.1378255429441	189	571	2.7629374	6	PALMD

**KIAA0125**	1.8821	1.04550164361416	758	344	3.7642542	6	KIAA0125
**PHLDA1.1**	6.9523	-1.51818966019541	343	683	1.3904791	6	PHLDA1
**GNLY**	3.9006	-1.46283945886208	163	0.45	7.8013479	6	GNLY
**SOD3.4**	4.6314	-1.58773811679628	197	552	9.2628496	6	SOD3
**BANK1**	4.9737	2.03658514320457	757	276	9.9475997	6	BANK1
**KLF4.2**	3.7560	-1.51268772162017	437	733	7.5121684	6	KLF4
**ADAMTS2.3**	4.3927	-0.459752516845957	0.22	495	8.7854753	6	ADAMTS2
**SGK1.2**	6.5914	-1.00835404312332	295	676	1.3182805	6	SGK1
**ECM1.4**	1.7837	-0.456279010337456	121	455	3.5675707	6	ECM1
**CD200.3**	2.5185	-0.415990521889801	203	546	5.0370788	6	CD200
**ANGPT2**	1.7807	-0.350595344186232	0.13	403	3.5615646	6	ANGPT2
**IL32**	2.9382	-1.81051557600388	356	738	5.8765277	6	IL32
**IGF2.2**	4.2578	-1.0842909167275	271	571	8.5157261	6	IGF2
**PIM2**	3.4795	0.444854433001228	852	472	6.9590863	6	PIM2
**DPT.2**	1.2240	-0.322215230678879	0.05	391	2.4480709	6	DPT
**CP.1**	1.5443	-0.341699163977564	188	472	3.0887843	6	CP
**LAPTM5**	1.0568	1.6831515187507	851	0.57	2.1137332	6	LAPTM5
**FAM167B.4**	2.0624	-0.345171548586582	183	485	4.1248338	6	FAM167B
**CAV1.4**	5.5956	-1.96847972194199	357	0.67	1.1191216	6	CAV1
**MFAP4.2**	5.0033	-0.6389176179748	169	449	1.0006779	6	MFAP4
**AQP1.4**	8.1524	-2.84767711113569	394	704	1.6304853	6	AQP1
**OLFM1.2**	1.0553	-0.461180526530197	32	0.42	2.1107700	6	OLFM1
**TNF**	3.6204	0.393518091561192	829	521	7.2409387	6	TNF
**C10orf10.2**	3.5786	-0.644607461482612	111	433	7.1573625	6	C10orf10
**S100A11.1**	5.5125	-1.62262886850573	0.58	839	1.1025179	6	S100A11
**THSD7A.3**	2.4988	-0.636063725326937	71	438	4.9977176	6	THSD7A
**SLC9A3R2.1**	1.4464	-0.865123356119447	0.13	448	2.8929027	6	SLC9A3R2
**TIMP1.4**	6.7949	-2.07057212906038	412	731	1.3589869	6	TIMP1
**UPP1.1**	6.0848	-0.621703799256995	0.24	567	1.2169797	6	UPP1
**CRISPLD2.2**	1.3131	-0.869247059785162	281	0.59	2.6263966	6	CRISPLD2
**ZNF385D.2**	9.0308	-0.717084020650504	169	523	1.8061614	6	ZNF385D
**TTN**	9.0963	0.504543753891235	0.78	0.44	1.8192723	6	TTN

**ITGB4.3**	1.0651	-0.559814942178306	139	451	2.1302779	6	ITGB4
**MAP1B.3**	1.2074	-1.11030158846233	254	545	2.4149904	6	MAP1B
**CD52**	5.4781	1.19801175198527	0.88	564	1.0956384	6	CD52
**GJA1.3**	7.6273	-0.634480287890171	203	491	1.5254793	6	GJA1
**CD9.5**	9.5356	-1.76529621789353	358	681	1.9071267	6	CD9
**EGFL7.3**	2.3103	-0.824521312315382	143	448	4.6207890	6	EGFL7
**TWIST1.2**	4.8306	-1.09985246819179	0.16	473	9.6613382	6	TWIST1
**CLEC14A.2**	7.1528	-1.13994991032902	273	547	1.4305781	6	CLEC14A
**NCOA7.4**	1.2784	-1.2729657541753	314	647	2.5568322	6	NCOA7
**CRABP2.4**	2.0976	-0.563952667316424	0.11	408	4.1952039	6	CRABP2
**AL928768.3**	6.5906	1.28472061936118	685	215	1.3181354	6	AL928768.3
**EFHD2.1**	3.3197	-0.520392482463265	251	591	6.6395078	6	EFHD2
**PHLDA3.1**	1.6769	-0.355209111060172	103	398	3.3539209	6	PHLDA3
**POU2AF1**	2.9837	0.793060866273099	778	0.51	5.9675713	6	POU2AF1
**MPZL2.3**	1.0009	-0.451198753878385	85	457	2.0019602	6	MPZL2
**BCL2A1**	9.7998	1.20668070292817	759	409	1.9599707	6	BCL2A1
**HBA1.1**	6.6180	0.484628547608555	784	417	1.3236051	6	HBA1
**TPM2.2**	1.7363	-1.48169862956825	138	436	3.4726233	6	TPM2
**ANPEP.4**	4.9378	-0.350478495420102	101	397	9.8756329	6	ANPEP
**ARHGAP29.2**	4.3864	-0.917896766808596	212	551	8.7728642	6	ARHGAP29
**CALD1.4**	5.6718	-2.62993533594451	439	696	1.1343795	6	CALD1
**MAST4.1**	1.3827	-0.604516846618414	245	526	2.7654294	6	MAST4
**ANTXR1.1**	1.0626	-0.600035573014585	181	545	2.1253101	6	ANTXR1
**HEG1.4**	5.7046	-1.25075186635522	357	629	1.1409254	6	HEG1
**PDLIM4.2**	3.5278	-0.273694297061329	122	0.47	7.0556737	6	PDLIM4
**CLEC11A.2**	6.2877	-0.461711386121786	164	486	1.2575516	6	CLEC11A
**PRRX2.3**	1.2379	-0.618634554493064	182	484	2.4758863	6	PRRX2
**C1QB.1**	2.9776	-0.259260499296091	802	469	5.9553973	6	C1QB
**IGFBP2.2**	1.0651	-1.98262062548321	301	0.61	2.1302424	6	IGFBP2
**CNKSR3.1**	6.1442	-0.457080999142379	177	513	1.2288576	6	CNKSR3
**MXRA8.3**	4.7174	-0.664823556097745	0.21	488	9.4348958	6	MXRA8
**CLDN5.3**	2.2077	-1.47226153135612	199	0.46	4.4154784	6	CLDN5

**IGLV1-51**	3.8955	-0.358624042586939	668	251	7.7911626	6	IGLV1-51
**HSPB1.3**	8.3296	-1.12026453432667	338	634	1.6659291	6	HSPB1
**FBLN5.2**	4.8020	-0.452204397930155	134	439	9.6040919	6	FBLN5
**POU2F2.1**	5.5354	1.99199655569444	738	459	1.1070953	6	POU2F2
**CYYR1.2**	1.4686	-0.578905940038438	181	434	2.9372625	6	CYYR1
**FMO3.1**	1.5322	-0.317425923377673	47	336	3.0645066	6	FMO3
**SDC2.4**	1.1771	-0.463059482858225	116	383	2.3542693	6	SDC2
**MMP14.1**	1.4824	-0.459085306555665	256	569	2.9649224	6	MMP14
**STEAP4.1**	3.7594	-0.456498780805065	132	428	7.5188782	6	STEAP4
**RORB.2**	6.7839	-0.520703525457157	0.18	466	1.3567953	6	RORB
**ID1.2**	3.7090	-1.34654227061033	0.32	591	7.4181379	6	ID1
**S100A4.2**	5.0810	-1.93324712690557	491	768	1.0162047	6	S100A4
**OMD.2**	1.0737	-0.404072681016848	147	436	2.1475515	6	OMD
**MT2A.3**	1.3258	-2.0888806551737	406	672	2.6517395	6	MT2A
**SERPINB9**	2.1373	0.349584473980911	878	597	4.2746538	6	SERPINB9
**RALGPS2.2**	1.2782	1.23264215871848	723	422	2.5565336	6	RALGPS2
**PLXDC1.1**	8.9495	-0.41135231408734	168	469	1.7899186	6	PLXDC1
**SPATS2L.3**	1.1427	-1.02307847373274	288	546	2.2854727	6	SPATS2L
**RHOBTB3.2**	2.9856	-0.349789734306558	109	434	5.9713136	6	RHOBTB3
**SEPP1.3**	3.0351	-0.928152227365753	207	513	6.0702462	6	SEPP1
**UNC5B.3**	1.3720	-0.316378243812837	83	466	2.7441401	6	UNC5B
**UACA.2**	3.8185	-0.68762126202275	0.22	483	7.6371928	6	UACA
**GSTP1.2**	5.6419	-0.886050267121713	422	725	1.1283969	6	GSTP1
**HLA-DMB**	7.1165	1.31503726641336	712	0.33	1.4233071	6	HLA-DMB
**IGLV3-1**	1.0764	0.496769018487604	0.69	355	2.1528346	6	IGLV3-1
**IGLV2-14.2**	1.8955	0.349930632839668	724	198	3.7911253	6	IGLV2-14
**IL33.3**	4.3858	-0.498844879135273	175	431	8.7717657	6	IL33
**MRC2.3**	1.7255	-0.600780072952204	0.19	454	3.4511261	6	MRC2
**SORBS2**	4.9873	-0.395109370922155	138	409	9.9746300	6	SORBS2
**PLOD2.2**	6.3067	-0.392520740278286	203	0.51	1.2613414	6	PLOD2
**SGIP1.1**	1.7624	-0.393407145905693	252	517	3.5248342	6	SGIP1
**PLAT**	3.1323	-0.294468412551663	194	469	6.2647576	6	PLAT

**BHLHE41.2**	8.8960	0.417604119447423	743	0.42	1.7792075	6	BHLHE41
**HPGD**	1.1560	-0.552191607152379	58	334	2.3120601	6	HPGD
**INHBA.2**	1.9287	-0.330273440554439	125	444	3.8575247	6	INHBA
**ADM5.1**	2.1166	-0.36917489110031	158	461	4.2333701	6	ADM5
**SPON2.2**	1.1313	-0.342237518596911	806	0.41	2.2626589	6	SPON2
**GRASP.5**	3.9574	0.39230011285661	0.86	557	7.9149632	6	GRASP
**NOTCH3**	1.9489	-0.634161930238561	122	0.4	3.8979520	6	NOTCH3
**BMP2.1**	1.4178	-0.401294134586993	213	564	2.8356411	6	BMP2
**SMOC2**	2.1517	-0.379702756142653	671	328	4.3034849	6	SMOC2
**PPFIBP1.4**	1.0381	-0.593278486558426	188	484	2.0762158	6	PPFIBP1
**TXN.3**	8.0990	-0.859203801268634	409	0.7	1.6198087	6	TXN
**RCAN2**	1.1327	-0.346719250505934	822	324	2.2655826	6	RCAN2
**CYP26B1.3**	2.0235	-0.314616244752409	46	337	4.0471069	6	CYP26B1
**CCL4L2**	6.0481	-1.12826170149257	802	516	1.2096201	6	CCL4L2
**GZMB.1**	8.2824	-0.510341455422752	729	471	1.6564923	6	GZMB
**MT1M.3**	4.8054	-0.585200285247265	835	375	9.6109196	6	MT1M
**TGFBI.1**	1.0641	-0.357185366673669	847	461	2.1283949	6	TGFBI
**SNCG.1**	3.4153	-0.616321131646207	152	0.48	6.8306682	6	SNCG
**CKS2**	4.9562	0.283919663692579	769	456	9.9125520	6	CKS2
**CD8A.1**	5.6950	-0.685889190067115	836	561	1.1390126	6	CD8A
**SPI1**	1.9240	0.334011610483356	637	309	3.8481088	6	SPI1
**LSP1**	6.6621	0.676251530666257	0.77	501	1.3324323	6	LSP1
**VASN.1**	4.1780	-0.285094608231836	822	457	8.3560268	6	VASN
**CCR6.1**	5.2557	0.577041979058709	0.63	251	1.0511533	6	CCR6
**CRTAM.1**	5.7700	-0.608335277052443	818	512	1.1540024	6	CRTAM
**PDGFA**	1.0580	-0.449908544019558	139	0.47	2.1161402	6	PDGFA
**EPSTI1.2**	7.3232	0.370754233727336	0.72	414	1.4646569	6	EPSTI1
**GRB10.2**	2.8129	-0.318010577755562	0.16	519	5.6258902	6	GRB10
**FAM13C.2**	4.0548	-0.45101335697616	71	388	8.1097759	6	FAM13C
**IGF1.1**	4.9611	-0.39144167491754	844	487	9.9222715	6	IGF1
**STAB1.2**	7.2700	-0.286285816473284	796	536	1.4540109	6	STAB1
**ENPEP.2**	2.2880	-0.320949180084837	146	448	4.5761433	6	ENPEP

**STEAP1.3**	3.4643	-0.440196256682517	84	439	6.9286607	6	STEAP1
**CXorf21**	4.7468	0.273682489105187	0.66	381	9.4936381	6	CXorf21
**SLIT3.4**	6.9431	-0.574458388800484	95	451	1.3886361	6	SLIT3
**SRPX.4**	6.6805	-0.262508300886874	72	421	1.3361095	6	SRPX
**FBXO32**	1.5198	-0.251097438223053	781	439	3.0397715	6	FBXO32
**COMP**	3.3224	-0.257594701437748	0.11	394	6.6449643	6	COMP
**FST.1**	3.4777	-0.444373376636265	122	466	6.9555209	6	FST
**ID4.1**	1.0265	-1.06634240388667	194	468	2.0530309	6	ID4
**TP53INP1**	4.3056	0.360243456913794	764	504	8.6112584	6	TP53INP1
**SERPINE2**	2.2673	-0.357426537296768	134	464	4.5346869	6	SERPINE2
**MECOM.3**	6.1514	-0.299764797746317	729	385	1.2302906	6	MECOM
**RGS5**	1.1497	-0.826700484104577	683	372	2.2994030	6	RGS5
**PROCR.2**	2.2692	-0.26010722645483	88	373	4.5385580	6	PROCR
**LY86**	2.0498	1.08533164542927	585	0.23	4.0996897	6	LY86
**ZNF503.2**	6.9804	-0.472559807887568	141	477	1.3960809	6	ZNF503
**OLFML2B**	1.0473	-0.465949853089075	849	596	2.0947813	6	OLFML2B
**IGLL5**	1.0137	0.316630925804091	574	309	2.0275749	6	IGLL5
**RBP7.2**	1.7650	-0.257648658969384	33	313	3.5301077	6	RBP7
**FCER1G**	5.3207	-0.71546992999252	662	393	1.0641414	6	FCER1G
**SYNPO2.1**	1.3396	-0.352293604304755	794	384	2.6793947	6	SYNPO2
**IL34.1**	8.6049	-0.276653277534286	186	459	1.7209887	6	IL34
**ANGPT1.2**	1.5117	-0.336578587965008	727	465	3.0234899	6	ANGPT1
**SFRP4.1**	1.4038	-0.39084284202861	669	0.39	2.8076698	6	SFRP4
**ROBO1.2**	4.8877	-0.378423090403536	833	509	9.7754154	6	ROBO1
**EDNRB.2**	3.5528	-0.325043290617093	714	409	7.1057431	6	EDNRB
**ISLR.4**	4.0687	-0.411862482859197	129	394	8.1375112	6	ISLR
**GPNMB.2**	8.3517	-0.525751329759714	848	544	1.6703548	6	GPNMB
**GZMA.1**	3.0365	-1.05916890848923	775	0.52	6.0731084	6	GZMA
**C1orf162**	3.8787	0.268962482880972	588	331	7.7575600	6	C1orf162
**IL3RA.4**	4.5714	-0.67863564105326	827	565	9.1428968	6	IL3RA
**PRF1**	3.5349	-0.287295358696478	716	362	7.0698302	6	PRF1
**PIK3R3.2**	1.1020	-0.326947992569021	815	0.48	2.2040721	6	PIK3R3

**SERPINF1.4**	4.9646	-1.29464061327665	799	547	9.9293528	6	SERPINF1
**CSF3.2**	2.5097	-0.47929779811178	808	511	5.0195458	6	CSF3
**CRABP1.3**	1.0699	-0.460837058839968	68	331	2.1398742	6	CRABP1
**PTGFR.3**	1.6689	-0.310432801656843	837	397	3.3378281	6	PTGFR
**RASD1**	4.0138	-0.579446618508266	838	511	8.0277116	6	RASD1
**GAS1.3**	1.7911	-0.889138092276378	782	488	3.5823317	6	GAS1
**JAML.1**	5.6109	-0.412898401686093	702	426	1.1221965	6	JAML
**PID1.3**	3.6499	-0.293833524814052	42	303	7.2999646	6	PID1
**C2CD4B.3**	4.0325	-0.679060839830853	776	524	8.0651777	6	C2CD4B
**PGF.1**	9.1703	-0.308432125192278	763	479	1.8340643	6	PGF
**LTBP4**	3.5388	-0.293769197142298	693	427	7.0776047	6	LTBP4
**SPIB**	5.9294	1.27678694124012	512	203	1.1858886	6	SPIB
**PLAU.1**	3.8310	-0.318279920266966	823	443	7.6620255	6	PLAU
**MMRN1.2**	4.7981	-0.418971548790233	754	385	9.5962712	6	MMRN1
**TWIST2.1**	1.4550	-0.633398374514623	831	559	2.9100887	6	TWIST2
**PDGFB.1**	2.0334	-0.289363184732244	838	503	4.0668060	6	PDGFB
**PKP4.3**	3.5698	-0.597859604958585	835	0.55	7.1396599	6	PKP4
**F13A1.1**	8.5878	-0.264586780147539	0.79	394	1.7175667	6	F13A1
**PRKAR2B**	2.8879	-0.251033873807733	831	443	5.7758045	6	PRKAR2B
**GLIS3.3**	3.3551	-0.281755154249949	836	447	6.7103522	6	GLIS3
**PRDX4.2**	1.7591	-0.326900316718092	817	505	3.5183345	6	PRDX4
**COL5A3.3**	4.4038	-0.297628912905507	825	438	8.8076439	6	COL5A3
**GPX3.1**	2.9682	-0.294033463659168	846	459	5.9364783	6	GPX3
**XCL2.1**	3.4967	-0.398478079383292	686	394	6.9935759	6	XCL2
**EDIL3.3**	1.1910	-0.805596880946646	801	549	2.3820383	6	EDIL3
**NREP**	4.3475	-0.337116918846697	755	478	8.6950409	6	NREP
**EFEMP2.1**	4.6427	-0.433409051175499	0.81	465	9.2855560	6	EFEMP2
**TIGIT**	8.8490	-0.57329131314012	843	562	1.7698115	6	TIGIT
**GZMH**	1.1827	-0.40211888537097	825	466	2.3654970	6	GZMH
**PDGFRA.4**	1.5980	-0.820233608272679	839	583	3.1961135	6	PDGFRA
**TNC.3**	1.6721	-0.322019476248239	696	401	3.3443539	6	TNC
**HNMT.1**	8.1911	-0.347020887989539	791	424	1.6382213	6	HNMT

**COL16A1.2**	6.6619	-0.357117859628777	784	421	0.0001332	6	COL16A1
**FAM129C**	3.9359	0.404748037498319	455	0.15	0.0078719	6	FAM129C
**XCL1.1**	4.1483	-0.517402784186253	851	528	0.0082966	6	XCL1
**LPAR1.1**	0.0002	-0.34249102794886	805	453	0.4100418	6	LPAR1
**MDK.2**	0.0002	-0.608842823218395	793	532	0.4429792	6	MDK
**CD14**	0.0003	-0.466315180014618	837	562	0.7293001	6	CD14
**MTUS1.2**	0.0023	-0.761810176493817	784	493	1	6	MTUS1
**MMP19.1**	0.0078	-0.317025341609679	815	518	1	6	MMP19
**IGHG1**	0	2.93713419462253	946	681	0	7	IGHG1
**MZB1**	0	2.60915013631498	999	0.6	0	7	MZB1
**XBP1.2**	0	1.93803573026241	993	0.64	0	7	XBP1
**DERL3**	0	1.79422271851148	916	545	0	7	DERL3
**SEC11C**	0	1.66965343802818	975	518	0	7	SEC11C
**FKBP11.2**	0	1.33385645064254	934	491	0	7	FKBP11
**PRDX4.3**	0	1.22188611064479	953	498	0	7	PRDX4
**RAB30**	0	0.653952808973027	898	464	0	7	RAB30
**SLAMF7.1**	0	0.638239584543813	901	526	0	7	SLAMF7
**HERPUD1**	3.7022	1.23312359844156	984	667	7.4044869	7	HERPUD1
**IGHG2**	4.2873	2.53353279949767	822	558	8.5747279	7	IGHG2
**TP53INP1.1**	5.5753	0.543620263815641	852	499	1.1150677	7	TP53INP1
**DNAAF1**	1.7595	0.901420959288177	792	532	3.5190341	7	DNAAF1
**LINC01480**	2.9545	0.310429980598512	713	243	5.9090160	7	LINC01480
**SPRR1B**	1.6216	-0.818948932216986	735	202	3.2433324	7	SPRR1B
**CD79A.2**	1.3065	1.18560472725312	832	469	2.6131090	7	CD79A
**SFN**	3.8679	-0.664818599188078	773	273	7.7359925	7	SFN
**KRT6C**	2.1933	-0.335071668059707	738	211	4.3866075	7	KRT6C
**RALGPS2.3**	8.0283	0.274647062338638	841	416	1.6056632	7	RALGPS2
**ELL2**	4.3593	0.558971807346764	886	0.61	8.7186482	7	ELL2
**CRCT1**	2.0452	-2.44714334359664	668	154	4.0904199	7	CRCT1
**CALML3**	8.5602	-0.276250432571327	686	199	1.7120446	7	CALML3
**POU2F2.2**	1.9571	0.283742977138825	837	454	3.9142232	7	POU2F2
**IGHV1-2**	2.1774	0.267709538647841	683	273	4.3549088	7	IGHV1-2

**HBA1.2**	3.2510	-0.404510909995357	825	416	6.5021544	7	HBA1
**IGHV1-18**	9.6579	0.286566651412329	682	317	1.9315992	7	IGHV1-18
**KRT19**	9.6052	-0.352804882288472	713	232	1.9210547	7	KRT19
**FAM46C**	3.8585	0.676545687768461	832	0.54	7.7171603	7	FAM46C
**FHL2**	7.2119	-0.302800947036657	206	461	1.4423996	7	FHL2
**KRT15**	2.0843	-0.705825651578279	646	208	4.1687407	7	KRT15
**S100A14**	1.9399	-0.767358684538366	657	225	3.8799370	7	S100A14
**HBA2**	1.5973	-1.00351156160997	819	0.45	3.1947682	7	HBA2
**CD84**	8.6519	-0.4011339817906	817	484	1.7303982	7	CD84
**KLRB1**	1.8088	-0.74123868999427	0.68	0.28	3.6176548	7	KLRB1
**KRT16**	2.0882	-0.832994982432856	799	313	4.1764466	7	KRT16
**MS4A1.2**	2.7600	-0.49147912875306	737	0.35	5.5201825	7	MS4A1
**SPRR2E**	3.6255	-0.807263162069503	0.65	221	7.2510356	7	SPRR2E
**FXYD3**	6.6729	-0.346606195895341	629	222	1.3345841	7	FXYD3
**SBSN**	1.0792	-0.328920880314919	595	168	2.1585012	7	SBSN
**KRT17**	4.6760	-0.549007145116697	728	332	9.3520809	7	KRT17
**CD8B**	3.1100	-0.411146998540317	809	471	6.2201020	7	CD8B
**IFNG**	3.0702	-0.393220270308198	809	385	6.1404239	7	IFNG
**AOAH.1**	2.7124	-0.253131808196603	809	401	5.4249085	7	AOAH
**TPSB2**	1.1545	-1.99002164382638	825	469	2.3090887	7	TPSB2
**NEGR1.4**	5.1092	-0.251015809752944	774	301	1.0218463	7	NEGR1
**S100A7**	1.3126	-1.9216444767088	724	0.37	2.6253726	7	S100A7
**TACSTD2**	4.0378	-0.321587014568305	703	0.28	8.0757336	7	TACSTD2
**CTLA4**	1.1729	-0.376734509764484	0.8	444	2.3459601	7	CTLA4
**KRT6A**	2.8422	-1.22279278357887	802	364	5.6844069	7	KRT6A
**PIM2.1**	1.2846	0.788500771853851	731	0.48	2.5692916	7	PIM2
**SPRR2A**	3.2729	-1.94923722873737	748	362	6.5459195	7	SPRR2A
**WISP2.2**	1.8791	-0.272288213960341	781	333	3.7583600	7	WISP2
**BHLHE41.3**	3.4196	0.443499617923147	696	424	6.8393574	7	BHLHE41
**GZMB.2**	4.2709	-0.581512591201179	747	471	8.5418481	7	GZMB
**TNFRSF4**	1.3539	-0.275381411164802	771	438	2.7079806	7	TNFRSF4
**NTRK2.2**	4.7502	-0.339452771393392	0.74	324	9.5004959	7	NTRK2

**DSP**	1.2024	-0.604321485645566	752	317	2.4049311		7	DSP
**PITX1**	7.0803	-0.27694977028454	577	222	1.4160782		7	PITX1
**CRTAM.2**	1.3802	-0.659228405237428	828	512	2.7604492		7	CRTAM
**SPRR2D**	2.2778	-1.60103518330865	638	252	4.5557264		7	SPRR2D
**IGKV1D-39**	4.4349	0.380967862555745	295	28	8.8698012		7	IGKV1D-39
**TRAC**	2.1422	-1.21008983881142	819	484	4.2845354		7	TRAC
**MAL**	1.5452	-0.464831736389219	658	298	3.0904095		7	MAL
**PLAC8**	1.7768	-0.260005180884167	644	306	3.5536281		7	PLAC8
**ICOS.1**	2.4173	-0.493822880671903	803	515	4.8346819		7	ICOS
**SERPINB9.1**	3.1785	-0.282155915236492	0.86	599	6.3571386		7	SERPINB9
**GADD45A**	1.5809	0.261486398279996	908	636	3.1619783		7	GADD45A
**CRABP2.5**	5.2212	-0.618742463244048	735	0.37	1.0442451		7	CRABP2
**IGHV5-51**	3.3387	0.36539967895693	598	279	6.6775771		7	IGHV5-51
**IGKV1-16**	3.6779	0.298715794867231	592	282	7.3559547		7	IGKV1-16
**SLC7A2.2**	1.2322	-0.393784480264709	669	242	2.4645006		7	SLC7A2
**PTN.2**	1.4512	-0.357628482556941	671	313	2.9025849		7	PTN
**HBB**	2.0378	-1.6432468776523	824	0.38	4.0756250		7	HBB
**LSP1.1**	2.1796	-0.375452016539361	813	0.5	4.3593040		7	LSP1
**GZMH.1**	9.8055	-0.436052189837863	763	471	1.9611063		7	GZMH
**RAB11FIP1.1**	6.1457	-0.451824129881751	861	566	1.2291470		7	RAB11FIP1
**C1QB.2**	7.7938	-0.46098409074922	806	469	1.5587629		7	C1QB
**LY6D**	9.8142	-0.36323689476718	573	255	1.9628490		7	LY6D
**CD3E**	2.4340	-0.976596858580521	811	513	4.8680714		7	CD3E
**CYP7B1.1**	1.1071	-0.281567995019025	777	358	2.2143668		7	CYP7B1
**CD7**	3.1486	-0.72110174375212	814	533	6.2972049		7	CD7
**CYBB.1**	2.2103	-0.342408931037985	678	369	4.4206808		7	CYBB
**HPGD.1**	4.7930	-0.565294598045777	658	297	9.5861667		7	HPGD
**ITGA6.4**	5.2767	-0.369758649309506	903	605	1.0553448		7	ITGA6
**TRBC1**	1.0093	-1.3592875486951	818	561	2.0186949		7	TRBC1
**S100A2**	1.2518	-1.29489611628412	757	376	2.5037065		7	S100A2
**LTB.1**	2.3579	-1.23299672055207	0.82	463	4.7159944		7	LTB
**ACTA2**	7.1906	-1.56983362619195	721	362	1.4381282		7	ACTA2

**MT1M.4**	3.0635	-0.62446449326129	777	0.38	6.12713818	7	MT1M
**XCL1.2**	5.8134	-0.550376563683385	797	533	1.16268409	7	XCL1
**GPM6B**	7.2348	-0.278541559778727	723	386	1.44696908	7	GPM6B
**CTSG**	1.2934	-0.559575166295598	789	455	2.58690383	7	CTSG
**SSPN.2**	5.9087	-0.27524769659206	752	493	1.18174594	7	SSPN
**ID4.2**	1.0246	-1.0626572688844	0.77	433	2.04928497	7	ID4
**UCP2**	6.0176	-0.556423326720409	785	482	1.20352818	7	UCP2
**PTGFR.4**	4.0462	-0.309196931270969	775	402	8.09249604	7	PTGFR
**KRT5**	4.7985	-1.34221394410528	786	0.39	9.59716428	7	KRT5
**CSTA**	2.8916	-0.90327092118398	575	265	5.78332843	7	CSTA
**CRYAB.1**	3.8542	-0.378430531816425	617	274	7.70845951	7	CRYAB
**INHBA.3**	1.3001	-0.323385473739656	775	405	2.60039901	7	INHBA
**TRBC2**	2.3638	-1.38059761282538	0.82	557	4.72768267	7	TRBC2
**PRELP.4**	1.2524	-0.302105819771258	722	322	2.50481841	7	PRELP
**TNFRSF9**	2.9211	-0.309762220461233	828	549	5.84222782	7	TNFRSF9
**MPEG1.3**	4.9683	-0.276659802422415	719	403	9.93670158	7	MPEG1
**S100B**	4.7173	-0.32085605682896	595	272	9.43467785	7	S100B
**COL17A1.1**	4.3768	-0.424547012384367	635	308	8.75367250	7	COL17A1
**COL11A1.4**	4.4017	-0.582327419821246	752	391	8.80346687	7	COL11A1
**CD8A.2**	7.9939	-0.829463596587472	818	563	1.59879283	7	CD8A
**CD3D.1**	1.2769	-1.30196709893025	827	0.57	2.55386469	7	CD3D
**IFI6.3**	9.0867	-0.353090703604534	791	435	1.81735921	7	IFI6
**TFF3**	1.1684	-0.279031632468	0.59	232	2.33697128	7	TFF3
**COMP.1**	1.2431	-0.253004930638194	737	356	2.48628749	7	COMP
**CPA3.1**	1.3023	-0.790784618828021	693	354	2.60467811	7	CPA3
**INPP4B**	4.1735	-0.406591618871228	709	376	8.34702284	7	INPP4B
**NOTCH3.1**	4.9659	-0.634728334596466	707	365	9.93191812	7	NOTCH3
**SLCO2B1**	1.3203	-0.252600405139416	803	431	2.64072719	7	SLCO2B1
**CCL4L2.1**	2.0973	-1.28614420256236	785	518	4.19476675	7	CCL4L2
**STEAP4.2**	3.3682	-0.456806202091831	745	391	6.73656867	7	STEAP4
**SPOCK2**	9.7055	-0.754419927766808	803	521	1.94111729	7	SPOCK2
**C1QA**	4.0973	-0.549555960439436	808	507	8.19465395	7	C1QA

**PHLDA3.2**	7.0292	-0.300639066420655	697	362	1.4058417	7	PHLDA3
**ALOX5AP**	5.4300	-0.673371195575201	686	0.43	1.0860113	7	ALOX5AP
**KRT13**	2.6835	-1.17518753280872	748	403	5.3670174	7	KRT13
**LBP.1**	2.8839	-0.366971853500607	785	471	5.7679093	7	LBP
**FAT1.4**	3.9451	-0.279989900832683	0.71	361	7.8902259	7	FAT1
**LCP1.1**	5.9577	-0.968893089035326	828	562	1.1915563	7	LCP1
**FYB**	9.7259	-1.25504358771115	833	571	1.9451905	7	FYB
**EDNRB.3**	9.8477	-0.347533966238929	802	405	1.9695439	7	EDNRB
**RASD1.1**	2.6758	-0.562212597407532	803	514	5.3517920	7	RASD1
**FRZB.2**	6.4688	-0.422798114300417	666	391	1.2937646	7	FRZB
**TNFSF13B.1**	2.3446	-0.28726401868155	742	432	4.6892044	7	TNFSF13B
**TNF.1**	2.9021	-0.396783559523594	814	523	5.8043700	7	TNF
**MXD1**	4.9641	-0.329494673944965	783	0.51	9.9282283	7	MXD1
**STEAP2.2**	8.1930	-0.367606063134515	781	463	1.6386085	7	STEAP2
**TNC.4**	4.3558	-0.333543209247387	766	397	8.7116018	7	TNC
**TPSAB1**	8.3651	-0.708891205030449	547	254	1.6730273	7	TPSAB1
**AKAP12.3**	1.8471	-0.590214973632164	834	573	3.6942814	7	AKAP12
**TGFBI.2**	1.4244	-0.443734548396709	768	467	2.8489703	7	TGFBI
**NET1**	2.5815	-0.406707481147471	0.73	418	5.1630190	7	NET1
**NES.3**	5.1978	-0.42747390661463	709	347	1.0395717	7	NES
**CPXM1.1**	4.2875	-0.264548624916628	775	497	8.5750489	7	CPXM1
**NDUFA4L2**	7.2476	-0.545028426750497	509	221	1.4495201	7	NDUFA4L2
**KRT14**	1.2644	-2.01368088992243	842	478	2.5289057	7	KRT14
**FBXO32.1**	1.8536	-0.292784490889833	719	443	3.7073068	7	FBXO32
**RHOBTB3.3**	3.0429	-0.393038105405644	712	397	6.0858509	7	RHOBTB3
**ACP5**	8.6942	-0.258717596497867	653	364	1.7388506	7	ACP5
**PPIF**	1.8687	-0.268004645795964	777	523	3.7375323	7	PPIF
**GREM1.2**	3.2888	-0.303221425624031	753	433	6.5776312	7	GREM1
**C12orf75.1**	7.2456	-0.424072845608779	677	381	1.4491294	7	C12orf75
**PPP1R12B**	1.0651	-0.318562037773657	624	0.37	2.1303297	7	PPP1R12B
**MTUS1.3**	1.4834	-0.689594866609319	794	493	2.9668528	7	MTUS1
**LGALS2**	2.7378	-0.305706103957163	547	282	5.4756268	7	LGALS2

**HMGB2**	6.2964	-0.688111827029561	0.82	467	1.2592942	7	HMGB2	
**SORBS2.1**	1.6478	-0.349168210828879	634	379	3.2956720	7	SORBS2	
**S100A8**	4.8405	-2.29615906412049	792	493	9.6811916	7	S100A8	
**PTPRB.4**	8.5001	-0.598607821222835	822	524	1.7000308	7	PTPRB	
**PLAT.1**	9.5365	-0.300385529788229	746	435	1.9073070	7	PLAT	
**EDNRA.1**	9.5913	-0.265293220233228	535	197	1.9182794	7	EDNRA	
**C1QC.1**	1.0629	-0.316346654412018	608	356	2.1258287	7	C1QC	
**KIAA1217.3**	4.8327	-0.330079280886408	758	434	9.6655757	7	KIAA1217	
**SFRP1.3**	7.5235	-0.364950145710622	0.78	409	1.5047071	7	SFRP1	
**MECOM.4**	2.9824	-0.318491438173506	736	386	5.9649306	7	MECOM	
**OLFM2.1**	3.8200	-0.281445795221387	681	415	7.6400210	7	OLFM2	
**EREG.1**	5.4353	-0.806921780552684	746	485	1.0870781	7	EREG	
**CPE.2**	7.1165	-0.921463873564703	774	508	1.4233158	7	CPE	
**CYGB.2**	1.3938	-0.296158839784675	708	439	2.7876772	7	CYGB	
**PHLDA2.2**	3.7370	-0.507011028472818	817	519	7.4741838	7	PHLDA2	
**LRRC15.3**	4.2184	-0.330363079174288	812	467	8.4368394	7	LRRC15	
**OGN.3**	7.9477	-0.302398431718479	675	384	1.5895583	7	OGN	
**CH25H.1**	1.1495	-0.409299949987792	628	352	2.2991666	7	CH25H	
**COL5A3.4**	1.0727	-0.301986426206343	765	443	2.1454209	7	COL5A3	
**STMN1**	1.4289	-0.653798744242481	664	0.39	2.8579370	7	STMN1	
**SMAD1.1**	3.6674	-0.399182962970752	744	412	7.3348357	7	SMAD1	
**ISLR.5**	7.7353	-0.411522674023023	692	0.36	1.5470784	7	ISLR	
**LTBP4.1**	9.8129	-0.375444238171985	692	427	1.9625807	7	LTBP4	
**VASN.2**	1.1513	-0.321550892572692	795	0.46	2.3027687	7	VASN	
**CFI.3**	1.2203	-0.402349830534963	757	411	2.4406339	7	CFI	
**IL33.4**	2.4922	-0.533273834386432	747	397	4.9844405	7	IL33	
**NEXN**	2.6444	-0.263918582391342	691	0.39	5.2889749	7	NEXN	
**UNC5B.4**	4.6051	-0.307239890911523	747	425	9.2102392	7	UNC5B	
**APLNR.1**	1.7433	-0.520425021554796	0.81	452	3.4867162	7	APLNR	
**CCL23**	2.8173	-0.253431373077871	793	429	5.6346179	7	CCL23	
**PLAU.2**	4.2246	-0.331211474712925	764	447	8.4492070	7	PLAU	
**MASP1.3**	4.3320	-0.407951985827822	747	417	8.6641767	7	MASP1	

**ESAM.3**	3.4695	-0.741493581106314	0.79	515	6.9391930	7	ESAM
**OLFML3.3**	5.3562	-0.466996654613075	708	0.44	1.0712449	7	OLFML3
**CP.2**	9.5570	-0.340926045621373	791	436	1.9114083	7	CP
**RPGR**	1.8164	-0.469336620030071	731	438	3.6328971	7	RPGR
**FRMD6.3**	3.6270	-0.536992077025373	634	379	7.2540061	7	FRMD6
**FCN1**	1.4517	-0.262282169460217	0.54	246	2.9035032	7	FCN1
**ROBO4.4**	2.2994	-0.500672128263241	743	424	4.5989419	7	ROBO4
**EFNB2.1**	6.5099	-0.60057196170747	721	446	1.3019912	7	EFNB2
**PLXNA2.4**	6.5386	-0.360516219825721	787	424	1.3077257	7	PLXNA2
**SERPINE2.1**	2.2936	-0.342528260590441	697	429	4.5872997	7	SERPINE2
**IGKV1-27**	2.8669	0.463793230913084	481	141	5.7339958	7	IGKV1-27
**GRB10.3**	1.2481	-0.305528356146626	787	481	2.4963115	7	GRB10
**FADS1.1**	2.1127	-0.305201787389815	758	482	4.2255648	7	FADS1
**CSRP2.1**	2.9425	-0.665439968993559	692	398	5.8850175	7	CSRP2
**RUNDC3B**	4.3845	-0.259880406212641	622	0.34	8.7690443	7	RUNDC3B
**MMP9**	8.8817	-0.306906110461597	652	0.39	1.7763503	7	MMP9
**LOX.4**	9.8595	-0.265681001402679	641	357	1.9719115	7	LOX
**CD52.1**	1.2388	-1.36316050436418	827	568	2.4777392	7	CD52
**ADM5.2**	1.4262	-0.376762360469525	0.75	425	2.8525827	7	ADM5
**TBX2.1**	1.4574	-0.331776466562124	696	429	2.9149484	7	TBX2
**CRABP1.4**	4.4366	-0.47954696838573	619	297	8.8732136	7	CRABP1
**SPINK7**	1.5362	-0.45407404378287	0.37	92	3.0724153	7	SPINK7
**CNKSR3.2**	1.7773	-0.459417430577434	753	478	3.5546937	7	CNKSR3
**FAM167B.5**	2.2314	-0.354731353720858	732	451	4.4629091	7	FAM167B
**HSPA2**	1.3407	-0.27144028118755	653	397	2.6815186	7	HSPA2
**FABP5**	2.0831	-1.37747424853853	0.78	481	4.1662808	7	FABP5
**S100A9**	3.1584	-2.94722487139161	793	531	6.3169292	7	S100A9
**SRPX.5**	5.7232	-0.271622675729765	684	383	1.1446596	7	SRPX
**MMRN2.4**	7.2971	-0.684519063744829	709	429	1.4594321	7	MMRN2
**FZD4.1**	1.0889	-0.365662704071237	694	0.37	2.1778456	7	FZD4
**GATA2.2**	3.7933	-0.346854002538845	741	474	7.5867097	7	GATA2
**MCTP1.3**	1.2621	-0.705767007994599	814	543	2.5242645	7	MCTP1

**STAB1.3**	1.3061	-0.365251012784113	801	536	2.6123365	7	STAB1
**PRKCDBP.1**	1.5432	-0.921492578794724	776	488	3.0865367	7	PRKCDBP
**COL5A1.2**	1.9639	-0.972460905440051	766	0.51	3.9279375	7	COL5A1
**SNCG.2**	2.0440	-0.606378735867132	701	447	4.0880965	7	SNCG
**MYCT1.2**	2.1294	-0.397331182737001	741	463	4.2589834	7	MYCT1
**LBH**	3.2808	-0.919975934507106	808	501	6.5616182	7	LBH
**EPSTI1.3**	5.5127	-0.357982428438643	669	418	0.0001102	7	EPSTI1
**DDIT4**	2.0979	-0.760421600332309	839	564	0.0004195	7	DDIT4
**TNXB.2**	3.7882	-0.491819653792454	0.8	517	0.0007576	7	TNXB
**CRYBG3.2**	4.7864	-0.547892614780958	793	538	0.0009572	7	CRYBG3
**PDGFB.2**	1.3134	-0.304372675875608	802	506	0.0026269	7	PDGFB
**NOSTRIN.1**	2.9345	-0.452023446744095	731	463	0.0058690	7	NOSTRIN
**PERP.1**	2.9543	-0.9120147436684	725	458	0.0059087	7	PERP
**FBLIM1.1**	3.1339	-0.282566710700983	745	474	0.0062678	7	FBLIM1
**MS4A6A**	7.2149	-0.358334699701073	714	449	0.0144298	7	MS4A6A
**MPZL2.4**	1.1396	-0.479818371367131	685	0.42	0.0227923	7	MPZL2
**PCSK5.2**	2.7113	-0.467535256683615	778	509	0.0542277	7	PCSK5
**TMEM176A.3**	8.9144	-0.732586434809028	761	495	0.1782894	7	TMEM176A
**IL34.2**	0.0003	-0.287929591368	708	427	0.6794254	7	IL34
**CDH13.2**	0.0003	-0.36227695019543	758	486	0.6850606	7	CDH13
**ADAMTS4.1**	0.0007	-0.459023439031185	791	538	1	7	ADAMTS4
**SPON1.2**	0.0007	-0.373178533192529	758	494	1	7	SPON1
**IGLV3-27**	0.0089	0.47271190201356	401	32	1	7	IGLV3-27
**IGHG1.1**	0	4.34155312685103	958	682	0	8	IGHG1
**IGHG3**	0	4.3330320871432	0.97	0.71	0	8	IGHG3
**IGHG2.1**	0	3.79385047042181	0.87	557	0	8	IGHG2
**MZB1.1**	0	3.45241474846241	998	602	0	8	MZB1
**XBP1.3**	0	2.63354751655191	992	642	0	8	XBP1
**DERL3.1**	0	2.46858399151384	972	544	0	8	DERL3
**SEC11C.1**	0	2.10439411376488	0.98	0.52	0	8	SEC11C
**FKBP11.3**	0	1.78443691721294	951	493	0	8	FKBP11
**PRDX4.4**	0	1.67316381778875	961	0.5	0	8	PRDX4

**FCRL5**	0	1.5893109307462	0.89	566	0	8	FCRL5
**JSRP1**	0	1.58625076953644	805	0.47	0	8	JSRP1
**HERPUD1.1**	0	1.5226702652135	978	669	0	8	HERPUD1
**CD79A.3**	0	1.35526025460155	904	467	0	8	CD79A
**SLAMF7.2**	0	1.1160463920871	871	0.53	0	8	SLAMF7
**ELL2.1**	4.7430	1.00597646986066	885	612	9.4860484	8	ELL2
**FAM46C.1**	2.6690	1.11314595678986	895	539	5.3380964	8	FAM46C
**RAB30.1**	1.4726	0.917101187942567	812	471	2.9452551	8	RAB30
**TP53INP1.2**	7.4130	1.09540795368199	815	503	1.4826155	8	TP53INP1
**PIM2.2**	3.9626	1.26330867946799	823	477	7.9253571	8	PIM2
**SPAG4**	3.9478	1.17007616242425	772	503	7.8956519	8	SPAG4
**ANXA1.1**	6.1494	-2.78990739475212	574	824	1.2298872	8	ANXA1
**REL**	4.5075	-1.97850479342191	562	822	9.0150344	8	REL
**SDC1.1**	3.4609	1.0590960859992	757	446	6.9218313	8	SDC1
**BHLHE41.4**	1.7501	0.696088605318717	736	423	3.5003247	8	BHLHE41
**IGHV1-69-2**	2.5807	0.288721606172129	0.58	243	5.1615478	8	IGHV1-69-2
**IGHV3-43**	1.0425	0.322819746433675	539	185	2.0851408	8	IGHV3-43
**RALGPS2.4**	3.1868	0.518800980324776	757	423	6.3736879	8	RALGPS2
**PTP4A3.1**	1.1287	1.18711455574343	735	467	2.2575856	8	PTP4A3
**SLCO2A1.2**	2.4742	-1.13111584692726	322	644	4.9484347	8	SLCO2A1
**ZBP1**	6.8829	0.850648275110058	662	397	1.3765824	8	ZBP1
**FBN1.4**	1.8377	-1.61809691455918	383	684	3.6755632	8	FBN1
**POU2F2.3**	1.2081	0.522874302233063	752	0.46	2.4163087	8	POU2F2
**IGHV1-2.1**	2.1306	0.369670954027181	545	283	4.2612580	8	IGHV1-2
**ANTXR1.2**	9.2345	-0.610069843901589	247	539	1.8469145	8	ANTXR1
**ITGA8.2**	7.0028	-0.250557534498268	166	451	1.4005642	8	ITGA8
**CDH11.2**	4.2129	-0.743012008908723	282	563	8.4259221	8	CDH11
**C3.2**	6.5347	-0.483734915913269	129	434	1.3069563	8	C3
**IGHGP**	7.9636	1.88169032717887	594	314	1.5927286	8	IGHGP
**BGN.2**	3.8548	-1.08957067259794	347	629	7.7097956	8	BGN
**CRCT1.1**	3.1864	-2.42431262856424	447	169	6.3729994	8	CRCT1
**CNKSR3.3**	3.1157	-0.461410911128674	245	506	6.2315427	8	CNKSR3

**TMEM176B.2**	1.6046	-0.945850853130708	218	513	3.2092270	8	TMEM176B
**MIR4435-2H**	9.9778	-0.399142653094992	263	565	1.9955738	8	MIR4435-2H
**CCDC3.3**	9.4908	-0.604274474643463	211	482	1.8981605	8	CCDC3
**CXCL1.2**	8.1749	-1.25052731458589	266	526	1.6349933	8	CXCL1
**NREP.1**	3.9681	-0.385808730001057	255	507	7.9363051	8	NREP
**IGLV5-48**	4.3466	0.306193604569356	381	79	8.6933907	8	IGLV5-48
**PLVAP.3**	0	2.17929757480659	948	562	0	9	PLVAP
**AQP1.5**	0	2.1197649308721	986	0.67	0	9	AQP1
**GNG11.4**	0	1.83293405149773	942	564	0	9	GNG11
**COL15A1.1**	0	1.82499489349725	958	651	0	9	COL15A1
**A2M.5**	0	1.80784536017581	969	641	0	9	A2M
**PECAM1.4**	0	1.74432304185793	952	0.66	0	9	PECAM1
**SPARCL1.4**	0	1.64487658383008	982	628	0	9	SPARCL1
**RAMP2.4**	0	1.6158460489817	0.91	463	0	9	RAMP2
**IFI27.4**	0	1.54628245772237	963	585	0	9	IFI27
**HSPG2.4**	1.3414	1.64259908067403	949	676	2.6828149	9	HSPG2
**TM4SF1.4**	1.2936	1.49434450454864	947	603	2.5872753	9	TM4SF1
**GSN.4**	3.5328	1.60149062253974	935	683	7.0656084	9	GSN
**ITGA6.5**	3.5527	1.64017381102721	898	607	7.1055539	9	ITGA6
**NPDC1.4**	4.0540	1.51044760934501	903	543	8.1080337	9	NPDC1
**CD93.2**	1.7470	1.44687904792954	908	658	3.4940069	9	CD93
**TIMP3.3**	5.2454	1.55627865080682	0.91	0.65	1.0490962	9	TIMP3
**ACKR1.3**	1.1472	1.1605851170227	899	537	2.2944629	9	ACKR1
**VWF.4**	1.1177	1.26728465061971	889	564	2.2355355	9	VWF
**EMCN.3**	5.3642	1.42767605248807	846	509	1.0728491	9	EMCN
**PCDH17.3**	2.7637	1.53294762684352	819	538	5.5274154	9	PCDH17
**CD34.3**	9.5847	1.49400303443333	817	497	1.9169405	9	CD34
**ADGRL4.5**	3.4938	1.45611439488285	861	579	6.9876514	9	ADGRL4
**CLEC14A.3**	2.2615	1.57703777856196	824	516	4.5230498	9	CLEC14A
**ECSCR.1.3**	1.0968	1.33164781453415	861	0.56	2.1936312	9	ECSCR.1
**HYAL2.3**	3.8061	1.42746395096378	839	485	7.6123231	9	HYAL2
**ENG.5**	6.8147	1.37308836148391	0.88	589	1.3629534	9	ENG

**DUSP23.2**	2.1073	1.30734421043932	831	464	4.21466055	9	DUSP23
**RAMP3.4**	1.1896	1.4279221921585	0.82	475	2.37931650	9	RAMP3
**SPRY1.2**	4.7595	1.51641557248953	0.85	582	9.51903362	9	SPRY1
**ROBO4.5**	3.8551	1.0276191058502	742	426	7.71033890	9	ROBO4
**ZNF385D.3**	4.3015	1.09564075536529	785	487	8.60304922	9	ZNF385D
**FLT1.3**	4.4822	1.2932206828982	787	482	8.96444468	9	FLT1
**PALMD.4**	4.1002	1.34756457726588	821	534	8.20059110	9	PALMD
**IGFBP7.6**	4.6886	0.984245409625787	993	0.72	9.37737048	9	IGFBP7
**CALCRL.3**	1.4548	1.42447124952331	764	451	2.90973080	9	CALCRL
**RNASE1.3**	2.7036	1.23029195326624	829	564	5.40738565	9	RNASE1
**ADAM15.5**	1.9946	1.14693764548833	816	517	3.98923060	9	ADAM15
**CAV1.5**	8.7041	1.15286925983465	908	639	1.74082707	9	CAV1
**ARHGAP29.3**	7.7150	1.30008883812526	783	518	1.54300004	9	ARHGAP29
**EGFL7.4**	1.4107	1.35959053439516	748	414	2.82157950	9	EGFL7
**PDLIM1.4**	7.0516	1.03729645019927	0.89	623	1.41033630	9	PDLIM1
**MMRN2.5**	2.3622	1.06673084462637	734	0.43	4.72447644	9	MMRN2
**PTGFR.5**	5.3788	-0.316347166608024	0.07	441	1.07577158	9	PTGFR
**CCDC3.4**	3.0508	0.904965906841935	736	454	6.10178600	9	CCDC3
**S100A4.3**	1.1380	-2.55545848957282	0.46	766	2.27613614	9	S100A4
**CLDN5.4**	9.3548	1.2401463492823	0.75	429	1.87096480	9	CLDN5
**PDGFRB.3**	2.2776	-1.02015952801019	177	581	4.55530787	9	PDGFRB
**TEK.3**	1.3801	0.745638023696619	698	431	2.76037514	9	TEK
**BCAM.2**	1.6898	1.13582867478235	771	508	3.37961379	9	BCAM
**ITGB4.4**	1.2352	0.888802164074695	692	0.42	2.47044254	9	ITGB4
**TSPAN7.2**	1.7840	1.03504865538091	718	458	3.56814112	9	TSPAN7
**ASS1.1**	9.6317	0.347497107241819	572	269	1.92634815	9	ASS1
**COL5A3.5**	2.4792	-0.299756822657038	89	0.48	4.95845769	9	COL5A3
**WISP2.3**	5.1352	-0.279584579526342	63	374	1.02705758	9	WISP2
**VWA1.2**	1.3383	1.21493022413616	702	451	2.67668080	9	VWA1
**MPZL2.5**	5.1021	0.590622702342648	686	422	1.02042540	9	MPZL2
**GJA1.4**	2.0131	0.739953726500748	717	462	4.02627117	9	GJA1
**CD9.6**	3.1461	0.855769146181836	0.9	0.65	6.29239429	9	CD9

**SOX18.3**	1.3212	1.29293680660894	698	445	2.6425916	9	SOX18
**S100A16.4**	8.5990	0.854318743245095	0.75	0.5	1.7198100	9	S100A16
**PLEKHA5.3**	5.4734	-0.572473668643925	147	508	1.0946964	9	PLEKHA5
**FAM107A.5**	5.4480	0.603962722977333	709	456	1.0896001	9	FAM107A
**ELOVL7.4**	3.4438	0.581359204580417	615	324	6.8876721	9	ELOVL7
**ADAMTS2.4**	2.7462	-0.445647163552886	177	495	5.4924665	9	ADAMTS2
**NES.4**	1.8686	0.852897890833423	613	355	3.7372144	9	NES
**ZNF331.2**	1.2190	-1.39038599865499	452	705	2.4380064	9	ZNF331
**DNASE1L3.3**	2.1039	0.772249863109978	642	382	4.2078147	9	DNASE1L3
**THSD7A.4**	6.0436	0.97339240712294	654	404	1.2087253	9	THSD7A
**RAB11FIP1.2**	1.2098	-0.751438639504867	337	595	2.4196203	9	RAB11FIP1
**TMEM176B.3**	7.4289	-0.896360306434537	209	513	1.4857876	9	TMEM176B
**FRZB.3**	1.7569	-0.681530596812645	115	422	3.5139531	9	FRZB
**LINC00152.1**	1.9175	-0.625843232220178	305	619	3.8350494	9	LINC00152
**CTSK.2**	3.6186	-1.5185818706353	327	596	7.2373449	9	CTSK
**CYP7B1.2**	1.4358	-0.271150149083978	0.12	395	2.8716187	9	CYP7B1
**INHBA.4**	1.0950	-0.340225459010761	171	438	2.1901298	9	INHBA
**LRRC15.4**	8.1841	-0.333899698458613	225	0.5	1.6368399	9	LRRC15
**OLFM2.2**	1.9592	-0.293551872505828	99	447	3.9185209	9	OLFM2
**RAB30.2**	6.2733	-0.27093376431222	0.25	501	1.2546700	9	RAB30
**MMP19.2**	4.7926	-0.315470894868584	297	547	9.5852766	9	MMP19
**C1orf162.1**	5.6428	-0.274220091768852	0.1	358	1.1285701	9	C1orf162
**MEG3.3**	6.7077	-0.855626347569544	169	426	1.3415598	9	MEG3
**SDC2.5**	8.1011	-0.464902756022695	127	0.38	1.6202317	9	SDC2
**FGF7.2**	1.9011	-0.729924159078733	156	414	3.8023138	9	FGF7
**PRSS12.3**	1.1671	-0.659512708044587	218	483	2.3342228	9	PRSS12
**FAT1.5**	4.5009	-0.297918497852742	0.12	394	9.0019905	9	FAT1
**LYZ**	0	5.65036459358661	999	585	0	10	LYZ
**HLA-DRA.2**	0	3.92212381093159	997	684	0	10	HLA-DRA
**AIF1**	0	3.69213009375979	0.99	368	0	10	AIF1
**HLA-DPB1.3**	0	3.63539466735255	982	0.59	0	10	HLA-DPB1
**HLA-DPA1.2**	0	3.61519622318341	982	634	0	10	HLA-DPA1

**HLA-DQB1.2**	0	3.50810303994227	983	556	0	10	HLA-DQB1
**TYROBP.1**	0	3.37465018317059	987	395	0	10	TYROBP
**HLA-DQA1.1**	0	3.34974739307123	956	426	0	10	HLA-DQA1
**CTSS.1**	0	3.2659012363135	996	618	0	10	CTSS
**HLA-DRB1.3**	0	3.15022032723183	982	662	0	10	HLA-DRB1
**HLA-DRB5.1**	0	3.1212325607957	988	735	0	10	HLA-DRB5
**FCER1G.1**	0	3.10409248971376	958	388	0	10	FCER1G
**PLAUR.1**	0	3.09859794041462	941	591	0	10	PLAUR
**CD14.1**	0	2.83581685818553	924	565	0	10	CD14
**LST1**	0	2.64963865226536	907	267	0	10	LST1
**FGL2**	0	2.63942597888468	934	454	0	10	FGL2
**GPR183**	0	2.5898351979525	934	531	0	10	GPR183
**CYBB.2**	0	2.53611806763253	865	368	0	10	CYBB
**PLEK.1**	0	2.52632393748003	911	389	0	10	PLEK
**SERPINA1**	0	2.45596086144187	0.88	337	0	10	SERPINA1
**RP11-1143G9**	0	2.39224252961766	913	432	0	10	RP11-1143G9
**FCGR2A**	0	2.37845152578642	0.94	0.4	0	10	FCGR2A
**C5AR1.1**	0	2.32838325602009	877	459	0	10	C5AR1
**CPVL**	0	2.30633173442093	896	393	0	10	CPVL
**CST3.5**	0	2.25360056276186	988	725	0	10	CST3
**LAPTM5.1**	0	2.18922937752215	982	571	0	10	LAPTM5
**CSF1R**	0	2.14126239722573	925	372	0	10	CSF1R
**TYMP.3**	0	2.11564594617099	978	696	0	10	TYMP
**MPEG1.4**	0	2.02006819835784	893	403	0	10	MPEG1
**ITGB2**	0	2.01944252105143	925	476	0	10	ITGB2
**HLA-DMB.1**	0	2.00124630343337	896	332	0	10	HLA-DMB
**CD300E**	0	1.92122544132548	814	359	0	10	CD300E
**MRC1**	0	1.91806287113255	855	467	0	10	MRC1
**FPR1**	0	1.86042184923803	837	358	0	10	FPR1
**CD163**	0	1.81804402166177	838	385	0	10	CD163
**OLR1**	0	1.81642211672552	753	217	0	10	OLR1
**FPR3**	0	1.76239640506169	856	362	0	10	FPR3

**LILRB2**	0	1.63739011726632	861	384	0	10	LILRB2
**RBM47**	0	1.60775859167431	878	448	0	10	RBM47
**CD86**	0	1.59733922620488	828	295	0	10	CD86
**FCGR3A**	0	1.39895472358771	802	305	0	10	FCGR3A
**PILRA**	0	1.05928569884359	794	232	0	10	PILRA
**TREM1**	0	0.970365348046012	713	132	0	10	TREM1
**FCGR1A**	0	0.947081741823482	785	225	0	10	FCGR1A
**AQP9**	0	0.925506141081538	712	175	0	10	AQP9
**CLEC4E**	0	0.89720448604776	698	174	0	10	CLEC4E
**SDS**	0	0.79506078275306	717	161	0	10	SDS
**CTB-61M7.2**	0	0.791250755238524	674	154	0	10	CTB-61M7.2
**LINC01272**	0	0.682979217810659	713	121	0	10	LINC01272
**MSR1**	0	0.666572274226673	0.68	114	0	10	MSR1
**CD209**	0	0.606685364444704	754	226	0	10	CD209
**MCEMP1**	0	0.375703883533541	593	76	0	10	MCEMP1
**MMP9.1**	2.8821	2.39808545209893	0.82	388	5.7642289	10	MMP9
**CLEC10A**	4.1089	2.5848908613216	802	265	8.2179777	10	CLEC10A
**ADAMDEC1**	8.2804	1.42167875630223	794	285	1.6560879	10	ADAMDEC1
**MS4A6A.1**	2.7057	2.61231633052488	862	449	5.4115237	10	MS4A6A
**LAIR1**	3.1388	1.20538730834457	816	347	6.2777077	10	LAIR1
**HCAR3**	2.5365	0.454692521503326	0.68	148	5.0730617	10	HCAR3
**EREG.2**	2.7371	4.1978642854282	839	487	5.4743661	10	EREG
**CXCL8**	1.9315	3.97360835587581	886	582	3.8630400	10	CXCL8
**SLC16A10**	1.7494	0.885116371590437	762	283	3.4988147	10	SLC16A10
**F13A1.2**	3.8544	2.02117216371427	844	0.4	7.7089600	10	F13A1
**FCN1.1**	3.7834	2.53012152039565	0.77	243	7.5669031	10	FCN1
**DSE.1**	1.6261	1.71893131567517	866	403	3.2523577	10	DSE
**TGFBI.3**	1.4529	1.87844917380592	882	469	2.9059479	10	TGFBI
**GRN.2**	1.4883	2.08676049690315	959	607	2.9767147	10	GRN
**CXCL16**	1.6381	1.6078485904618	868	508	3.2762273	10	CXCL16
**CD68.1**	1.0009	2.28668467450875	0.87	503	2.0019422	10	CD68
**VSIG4**	2.1092	0.3861439188618	648	109	4.2185101	10	VSIG4

**RNASE6**	2.7650	1.67234644550198	812	281	5.5301339	10	RNASE6
**IL1B**	1.3519	4.11015882736257	829	372	2.7038182	10	IL1B
**C1QB.3**	5.0521	3.13160677752793	837	475	1.0104307	10	C1QB
**ACP5.1**	8.8340	1.55703374409159	819	363	1.7668108	10	ACP5
**INSIG1**	9.0435	2.00773017045409	0.9	604	1.8087125	10	INSIG1
**SPI1.1**	9.3204	1.9391969323752	824	309	1.8640922	10	SPI1
**PLA2G7**	7.7705	1.26467801901768	774	361	1.5541173	10	PLA2G7
**VAMP8**	1.1323	1.87953701543848	901	387	2.2646102	10	VAMP8
**VEGFA.1**	5.2848	2.01637688432705	853	481	1.0569758	10	VEGFA
**MARCO**	2.2710	0.260037564336805	533	31	4.5420232	10	MARCO
**RGS2**	8.3096	2.06702063127516	923	607	1.6619399	10	RGS2
**HLA-DMA.1**	2.8899	2.13007097323246	889	458	5.7798107	10	HLA-DMA
**C1QA.1**	3.2997	3.43222342305057	0.83	512	6.5995088	10	C1QA
**CD83.1**	6.8770	1.84878146971282	906	593	1.3754166	10	CD83
**TNFSF13B.2**	2.0438	1.67842664442947	833	435	4.0877866	10	TNFSF13B
**MXD1.1**	1.0216	2.04220586456477	846	513	2.0433318	10	MXD1
**HLA-DQA2**	1.0490	2.19186917471471	857	485	2.0980673	10	HLA-DQA2
**ITGAX**	5.2836	1.12646368773354	739	242	1.0567288	10	ITGAX
**CTSB.4**	1.4364	2.52778555052362	972	618	2.8729405	10	CTSB
**LGALS2.1**	2.3411	1.94841534659771	779	278	4.6822463	10	LGALS2
**IGSF6**	2.3276	1.7390736620822	756	206	4.6552811	10	IGSF6
**S100A9.1**	4.1976	1.54847906949152	893	532	8.3953003	10	S100A9
**CLEC7A**	8.7448	1.5968093707653	761	0.23	1.7489633	10	CLEC7A
**C15orf48**	1.5675	2.09014853380332	766	261	3.1351418	10	C15orf48
**SLC11A1**	6.1228	1.36276556941572	744	315	1.2245762	10	SLC11A1
**C3AR1**	2.1830	0.944117567476658	746	328	4.3660483	10	C3AR1
**ADAP2**	1.0369	1.07813120997183	772	0.35	2.0738029	10	ADAP2
**AOAH.2**	4.0495	1.34421464711362	797	0.41	8.0990550	10	AOAH
**HAVCR2**	1.5752	0.885752635480807	744	311	3.1505330	10	HAVCR2
**MMP12**	4.5498	0.579744402731529	661	0.17	9.0997643	10	MMP12
**SLC8A1**	6.0379	1.16044031358396	778	398	1.2075972	10	SLC8A1
**RARRES1**	1.6060	0.875263676247067	739	0.28	3.2120693	10	RARRES1

**DSC2**	1.2317	0.538976491462576	717	253	2.46354352	10	DSC2
**PLA2G2D**	5.0784	0.778033924059198	672	226	1.01568368	10	PLA2G2D
**SLC43A2**	2.2413	1.29079428034458	774	399	4.48260792	10	SLC43A2
**NCF2**	1.4577	1.40967580792917	749	285	2.91548149	10	NCF2
**SOD2.2**	8.0622	1.97768169931042	987	728	1.61245685	10	SOD2
**PTAFR.1**	3.7154	0.996424729009508	775	367	7.43081827	10	PTAFR
**IFI30**	5.9756	1.58529880728065	746	345	1.19513493	10	IFI30
**C5AR2**	5.4441	0.387534484194602	717	314	1.08882167	10	C5AR2
**RAB31.3**	2.0986	1.39613336292318	902	617	4.19739494	10	RAB31
**IL10**	8.4816	1.11266419647463	724	348	1.69633654	10	IL10
**RASSF4**	1.8456	1.42038959564802	794	413	3.69136185	10	RASSF4
**RAB7B**	1.2462	0.28191872546787	685	247	2.49252888	10	RAB7B
**C1QC.2**	3.7031	2.58721251448022	737	356	7.40632629	10	C1QC
**PRSS23.3**	6.3997	-1.85586158870127	386	747	1.27994695	10	PRSS23
**GLUL.3**	8.4570	1.77501986022666	943	596	1.69140930	10	GLUL
**CCL3L3**	9.2814	2.05789518745363	733	409	1.85628383	10	CCL3L3
**PHACTR1.1**	1.6773	1.6210336679159	778	0.4	3.35466419	10	PHACTR1
**SGK1.3**	2.1571	1.9158139026277	909	644	4.31425459	10	SGK1
**GK**	1.5977	1.54119288379978	761	0.42	3.19546383	10	GK
**MMP19.3**	3.6513	1.18167850855851	806	525	7.30261108	10	MMP19
**ARL4C**	1.6957	1.71598107047254	934	682	3.39156559	10	ARL4C
**TFRC**	2.5075	1.66668441947578	861	595	5.01516647	10	TFRC
**METRNL**	3.5671	1.30046709189864	906	622	7.13430843	10	METRNL
**APOC1**	2.9114	2.28074064830856	746	0.41	5.82282460	10	APOC1
**EFHD2.2**	1.0320	1.55083589547438	857	0.56	2.06405774	10	EFHD2
**LCP1.2**	3.1227	1.34274382735451	889	565	6.24545848	10	LCP1
**LILRB5**	1.8577	0.356173442851338	559	0.16	3.71544409	10	LILRB5
**FYB.1**	2.7256	1.19969116610079	897	574	5.45129469	10	FYB
**STX11.1**	1.5690	1.13339057770792	787	534	3.13810210	10	STX11
**ATP1B1**	1.3481	0.653787037849579	762	476	2.69625211	10	ATP1B1
**MS4A7**	3.3178	1.71205245706281	0.71	254	6.63562439	10	MS4A7
**CLEC12A**	2.4901	0.567137534156034	626	133	4.98026049	10	CLEC12A

**IDO1.1**	1.6958	0.379821552565636	694	0.28	3.3916295	10	IDO1	
**PPIF.1**	1.3541	1.69667750985029	788	528	2.7082062	10	PPIF	
**ADGRG1.2**	2.4777	-0.383381121713589	192	578	4.9555646	10	ADGRG1	
**OTUD1**	6.8295	1.19933703338535	769	418	1.3659052	10	OTUD1	
**SLAMF8**	7.0186	0.849009148410686	708	369	1.4037375	10	SLAMF8	
**KCNMA1**	2.1031	0.357591691246068	726	296	4.2062460	10	KCNMA1	
**CCL3**	8.3563	2.90362541624956	0.74	0.47	1.6712672	10	CCL3	
**GPR84**	1.8148	0.587069812659994	639	258	3.6296361	10	GPR84	
**CAPG**	2.2046	1.46912249748499	771	369	4.4092189	10	CAPG	
**ADIRF.4**	3.6882	-2.59625462700781	315	678	7.3764516	10	ADIRF	
**IL1RN**	1.3016	2.13250331607726	671	199	2.6032915	10	IL1RN	
**MNDA**	1.9764	1.60979030181099	694	283	3.9528145	10	MNDA	
**PAPSS2.1**	1.9075	0.700622389264845	0.73	415	3.8151842	10	PAPSS2	
**ANPEP.5**	9.0243	0.624267928803707	734	366	1.8048620	10	ANPEP	
**STAB1.4**	1.4170	1.33693865081849	802	542	2.8340595	10	STAB1	
**ALDH2**	7.6574	1.33586132284096	734	313	1.5314892	10	ALDH2	
**SLCO2B1.1**	1.0771	1.22276761982834	733	442	2.1542845	10	SLCO2B1	
**HCK**	1.3490	1.17972047641139	665	228	2.6981907	10	HCK	
**ENPP2.2**	1.4630	0.960845932963413	734	427	2.9260735	10	ENPP2	
**SPINT2**	2.0250	1.18512760880607	731	313	4.0501363	10	SPINT2	
**SERPINB2**	2.8844	0.26766366306582	0.55	147	5.7688018	10	SERPINB2	
**MAFB**	1.7187	1.28064219996639	765	445	3.4374225	10	MAFB	
**HMOX1.1**	3.6854	1.29406874402328	733	448	7.3709003	10	HMOX1	
**ADGRE2**	2.7063	1.07866685971942	681	326	5.4126833	10	ADGRE2	
**GNA15**	7.3259	0.85968531957201	0.68	335	1.4651952	10	GNA15	
**KYNU.2**	1.6598	0.882368599081846	749	429	3.3196583	10	KYNU	
**SERPINB1**	2.2297	1.18635265029912	834	505	4.4594871	10	SERPINB1	
**CD36**	4.1086	0.735120701954628	594	127	8.2172071	10	CD36	
**RGS10**	9.7320	1.22652152925251	0.78	462	1.9464112	10	RGS10	
**CD1C**	5.2538	0.846385505450102	587	133	1.0507785	10	CD1C	
**MARCH1.1**	2.2937	1.11239225949292	688	342	4.5874763	10	MARCH1	
**CLEC5A**	1.4982	0.346167617070891	528	52	2.9964200	10	CLEC5A	

**ASPN.2**	2.4444	-0.75500397319593	165	0.49	4.8889316	10	ASPN
**ATF5**	2.5249	0.881576871539195	715	395	5.0498815	10	ATF5
**JAML.2**	3.8562	1.3636025512937	722	432	7.7125320	10	JAML
**GREM1.3**	5.7973	-0.289313703799973	131	463	1.1594736	10	GREM1
**NEURL1B.1**	4.2767	-0.474036188485655	149	526	8.5535839	10	NEURL1B
**MS4A4A**	4.0766	1.50071310539366	625	226	8.1533551	10	MS4A4A
**CRABP1.5**	5.5621	-0.392163847033976	689	301	1.1124388	10	CRABP1
**BASP1.4**	2.4763	1.39735109131324	771	468	4.9527099	10	BASP1
**NABP1**	1.0393	1.01353534223152	0.76	467	2.0787627	10	NABP1
**NLRP3**	1.3297	1.3684558835271	639	273	2.6595536	10	NLRP3
**APOBEC3A**	7.5103	0.545999927436393	658	0.36	1.5020745	10	APOBEC3A
**SIGLEC10**	5.0474	0.702305938071113	584	125	1.0094812	10	SIGLEC10
**TNXB.3**	2.1700	-0.485582114734628	109	549	4.3401528	10	TNXB
**STC1.1**	5.3148	-0.773735203308082	306	626	1.0629611	10	STC1
**CSTA.1**	8.6525	0.573327529251377	661	267	1.7305142	10	CSTA
**MYCT1.3**	1.1253	-0.400450611396182	53	495	2.2507309	10	MYCT1
**MIR3945HG**	2.2259	0.280564217455458	541	151	4.4519214	10	MIR3945HG
**AKAP12.4**	1.6430	-0.773338292548276	334	597	3.2861729	10	AKAP12
**ADAM28**	1.6409	0.750746541784021	0.68	394	3.2818601	10	ADAM28
**BCL2A1.1**	2.9296	1.67462708303142	685	0.42	5.8592770	10	BCL2A1
**TPM1.4**	8.9880	-1.36189714865457	207	518	1.7976030	10	TPM1
**FAM26F**	3.0352	1.09379906467221	618	222	6.0705396	10	FAM26F
**BCAM.3**	3.2274	-0.934650304067565	194	533	6.4549317	10	BCAM
**S100B.1**	1.4843	1.24438258753327	607	278	2.9686934	10	S100B
**TMEM176B.4**	1.2256	1.12820904821017	751	488	2.4513781	10	TMEM176B
**SLC39A8**	1.8926	0.348535596886222	728	461	3.7853687	10	SLC39A8
**SCARA5.1**	4.4922	-0.397771352767301	221	0.54	8.9844445	10	SCARA5
**RGS1**	3.2423	0.878284684594819	814	0.56	6.4846855	10	RGS1
**TMEM176A.4**	1.3321	0.931119040051325	757	501	2.6642654	10	TMEM176A
**FOLR2**	5.2450	1.03335612129296	631	288	1.0490166	10	FOLR2
**NRP2.1**	2.3572	0.253574392364471	775	509	4.7144931	10	NRP2
**CFP**	8.2922	0.909845009641057	612	264	1.6584449	10	CFP

**CD3E.1**	1.3117	-1.00773486452535	243	541	2.62355579	10	CD3E
**IGF2.3**	1.8691	-1.01664625226201	271	564	3.73833475	10	IGF2
**APLNR.2**	5.8793	-0.510454716796579	0.08	487	1.17587788	10	APLNR
**WNT5A.2**	2.6040	-1.08922427765663	333	584	5.20809692	10	WNT5A
**TRBC1.1**	1.2962	-1.39904127394648	307	586	2.59258840	10	TRBC1
**SFRP1.4**	1.2301	-0.354396811508404	161	0.44	2.46022320	10	SFRP1
**TFEC**	4.7076	0.711151669645671	551	124	9.41525735	10	TFEC
**IL4I1**	9.7947	0.908520793847346	599	312	1.95895542	10	IL4I1
**EMCN.4**	1.6381	-1.07133855497805	228	537	3.27624913	10	EMCN
**LILRB4**	2.0402	1.19147034883251	0.62	366	4.08045158	10	LILRB4
**CD2**	3.2158	-1.18996077837541	341	612	6.43169739	10	CD2
**HPGDS**	1.1417	-0.274503084240831	449	92	2.28345827	10	HPGDS
**LY86.1**	2.9831	0.897022087205494	0.6	237	5.96623185	10	LY86
**C1orf162.2**	6.7173	1.19950277519968	0.63	335	1.34346770	10	C1orf162
**OMD.3**	2.1935	-0.40129052918881	75	432	4.38719217	10	OMD
**CTSD.3**	5.1501	1.79068647978838	812	0.53	1.03003061	10	CTSD
**DIRAS3.1**	6.0541	-0.262802864855815	163	488	1.21083908	10	DIRAS3
**STEAP2.3**	3.0449	-0.327245590882706	239	0.49	6.08983698	10	STEAP2
**CEBPD.5**	6.3649	0.400875545006066	883	617	1.27299638	10	CEBPD
**DPT.3**	5.3854	-0.256380420927582	657	0.36	1.07709985	10	DPT
**KIAA1217.4**	1.6449	-0.33622758908151	168	463	3.28992259	10	KIAA1217
**BST2**	3.5871	0.574472962649961	832	532	7.17436059	10	BST2
**SPRY4.3**	5.3631	-0.251013679321008	82	472	1.07263068	10	SPRY4
**HCAR2**	7.2998	0.59513398554057	518	142	1.45996690	10	HCAR2
**FKBP11.4**	3.2496	-0.660395667596867	275	525	6.49932325	10	FKBP11
**IL18**	9.0310	0.879880821593943	569	227	1.80621333	10	IL18
**MTUS1.4**	5.0875	-0.800132893300131	269	519	1.01751778	10	MTUS1
**CD1E**	9.6828	0.661777139495996	465	45	1.93657253	10	CD1E
**NOSTRIN.2**	1.3708	-0.447134067718596	237	487	2.74175521	10	NOSTRIN
**SLIT3.5**	1.7232	-0.544441380549488	77	443	3.44644865	10	SLIT3
**IGLV3-1.1**	3.9051	-0.620501529254767	114	384	7.81025959	10	IGLV3-1
**PTX3.1**	3.4781	0.425486573749662	557	248	6.95624837	10	PTX3

**DSEL.2**	5.6356	-0.42093771548378	0.21	493	1.1271286	10	DSEL
**MASP1.4**	6.5377	-0.370261175600688	139	447	1.3075423	10	MASP1
**SGCB.3**	4.3722	-0.5018023032077	89	448	8.7445215	10	SGCB
**SCPEP1.2**	3.6405	0.479574016149506	671	421	7.2810344	10	SCPEP1
**NES.5**	1.0332	-0.416163731477686	24	0.38	2.0664447	10	NES
**IL34.3**	5.8627	-0.275461947181279	42	458	1.1725490	10	IL34
**MEOX1.2**	2.4193	-0.340390163899767	671	366	4.8386091	10	MEOX1
**PID1.4**	2.0482	0.49440958929414	582	277	4.0965682	10	PID1
**CSF2RA**	3.0587	0.985241466838148	531	138	6.1174753	10	CSF2RA
**IGLV3-21.2**	6.3974	-0.46555033929775	96	355	1.2794982	10	IGLV3-21
**LOX.5**	4.1020	-0.260830592996868	104	383	8.2040246	10	LOX
**NTRK2.3**	2.1131	-0.315284193767183	64	359	4.2263322	10	NTRK2
**NDUFA4L2.1**	2.1731	-0.559746793943141	486	228	4.3462878	10	NDUFA4L2
**CPA3.2**	5.6525	-0.723004148849649	103	383	1.1305007	10	CPA3
**VNN 1**	1.9068	0.308269445287792	451	165	3.8136569	10	VNN 1
**PTGFR.6**	2.1912	-0.299741443358525	105	435	4.3825547	10	PTGFR
**MFAP2.3**	3.5772	-0.268310835889626	82	387	7.1544963	10	MFAP2
**IL1A**	8.1539	0.488492277902807	0.46	162	1.6307882	10	IL1A
**ELOVL7.5**	2.5208	-0.316120912655506	607	328	5.0416217	10	ELOVL7
**PLAT.2**	6.8205	-0.316477399973476	208	462	1.3641096	10	PLAT
**CYP7B1.3**	2.0153	-0.290583657978842	44	395	4.0307233	10	CYP7B1
**S100A12**	9.0315	0.908495235176426	461	124	1.8063134	10	S100A12
**RUNDC3B.1**	6.4844	-0.262511036496358	76	366	1.2968923	10	RUNDC3B
**MEG3.4**	2.0865	-0.80158072542548	162	423	4.1731427	10	MEG3
**FCER1A**	9.6911	1.34746598927903	442	114	1.9382359	10	FCER1A
**ABI3BP.3**	9.8751	-0.356099765837386	68	398	1.9750357	10	ABI3BP
**ITGBL1.3**	2.2811	-0.573682118415841	78	365	4.5623232	10	ITGBL1
**SLC7A2.3**	1.3493	-0.387381648021225	18	276	2.6987664	10	SLC7A2
**P2RY13**	9.3770	0.67135144039422	413	78	0.0018754	10	P2RY13
**RNASE2**	1.2224	0.367969802477094	387	109	0.0244498	10	RNASE2
**SLC7A2.4**	0	2.2021047552409	846	246	0	11	SLC7A2
**GUCY1A2**	1.8339	1.01679207512225	764	204	3.6678101	11	GUCY1A2

**CALD1.5**	5.8659	2.12878918388884	984	0.67	1.1731862	11	CALD1
**NOTCH3.2**	7.5822	2.41075212590523	865	368	1.5164472	11	NOTCH3
**STEAP4.3**	5.1645	2.50347516590949	0.85	396	1.0329047	11	STEAP4
**NR2F2.3**	1.2842	2.58794144538266	914	524	2.5685243	11	NR2F2
**TAGLN.1**	2.1314	2.83987255876229	882	439	4.2628744	11	TAGLN
**ADIRF.5**	1.6317	1.9604203578914	968	654	3.2635591	11	ADIRF
**MYL9.3**	2.2588	2.29453922753802	902	565	4.5177754	11	MYL9
**C11orf96.3**	7.0561	2.73839515070173	935	657	1.4112311	11	C11orf96
**CRISPLD2.3**	1.0869	2.01748793175388	908	0.56	2.1738576	11	CRISPLD2
**ID4.3**	3.8482	2.65616818904481	862	438	7.6964997	11	ID4
**AGT**	2.8106	0.524268596280329	715	278	5.6213080	11	AGT
**MGP.2**	8.8575	1.70418125067535	922	578	1.7715109	11	MGP
**FILIP1L.3**	1.3121	1.96192634031258	915	583	2.6242786	11	FILIP1L
**IGFBP7.7**	7.2412	1.42929332952652	988	725	1.4482582	11	IGFBP7
**TPM2.3**	1.5493	2.04042077661893	839	404	3.0987931	11	TPM2
**RGS5.1**	2.0530	3.2478006323895	796	376	4.1060089	11	RGS5
**COL14A1.5**	6.1310	1.73224800458422	896	635	1.2262186	11	COL14A1
**ACTA2.1**	2.1892	2.26624215607447	809	367	4.3785131	11	ACTA2
**SGIP1.2**	1.5325	0.740194257288553	845	0.49	3.0651135	11	SGIP1
**TINAGL1.2**	7.3952	1.87511848645344	845	556	1.4790582	11	TINAGL1
**HLA-DRB1.4**	2.7319	-2.16350339949799	194	691	5.4638665	11	HLA-DRB1
**PRRX1.4**	7.3312	1.76686672323152	866	506	1.4662488	11	PRRX1
**S1PR3.2**	1.1143	0.285437518985034	738	255	2.2286133	11	S1PR3
**CPM**	4.9411	0.99044478517092	801	508	9.8823089	11	CPM
**CSF3.3**	2.5853	-0.543845026067361	95	543	5.1707499	11	CSF3
**CEBPD.6**	2.2350	1.75658604409496	964	615	4.4700837	11	CEBPD
**MT1M.5**	6.0498	1.97882030657856	784	389	1.2099760	11	MT1M
**MAP1B.4**	9.5543	1.98010876614458	823	518	1.9108770	11	MAP1B
**ENPEP.3**	2.5885	0.552059513647985	784	418	5.1770475	11	ENPEP
**TPM1.5**	1.3274	1.86536947437223	0.82	496	2.6548671	11	TPM1
**RNASE1.4**	2.8765	-1.10894373745195	216	589	5.7531347	11	RNASE1
**SDC2.6**	4.3228	0.771011427977324	794	353	8.6456836	11	SDC2

**CNN1**	8.5446	0.679849348007422	655	216	1.7089369	11	CNN1
**ADAMTS4.2**	2.7905	1.29757026374641	816	543	5.5810788	11	ADAMTS4
**FGF7.3**	2.8799	0.756251368513176	797	388	5.7598643	11	FGF7
**CLDN5.5**	3.1018	-1.50352847249025	102	456	6.2036501	11	CLDN5
**SOD3.5**	4.1936	1.61724489991352	863	0.52	8.3873530	11	SOD3
**MT1A**	1.0670	1.41178888881711	719	368	2.1340236	11	MT1A
**CSRP1**	1.1061	1.21911185912388	824	416	2.2123009	11	CSRP1
**PDGFRB.4**	6.9155	2.02172351080832	816	553	1.3831173	11	PDGFRB
**SYNPO2.2**	2.7218	2.00951715666212	748	397	5.4436589	11	SYNPO2
**FN1.3**	4.2298	0.810105278290034	885	592	8.4596937	11	FN1
**TNC.5**	2.6612	0.509422480894153	757	406	5.3224881	11	TNC
**MMP19.4**	6.6806	-0.276258154561514	135	549	1.3361357	11	MMP19
**AKAP12.5**	1.6802	0.677564164248155	855	578	3.3605592	11	AKAP12
**CH25H.2**	2.6025	0.411386514712616	0.75	354	5.2051489	11	CH25H
**SPARCL1.5**	2.2726	1.0431223578341	944	635	4.5452822	11	SPARCL1
**TGFBI.4**	7.9497	0.751182746089234	796	473	1.5899436	11	TGFBI
**KCTD12.2**	4.1304	-0.832382302433963	266	606	8.2609170	11	KCTD12
**PLAU.3**	3.2566	0.457072334760794	757	455	6.5133555	11	PLAU
**MYC**	8.0272	1.25315493118202	794	471	1.6054474	11	MYC
**SLC9A3R1**	5.8214	-0.305289474632215	141	435	1.1642810	11	SLC9A3R1
**SERPING1.4**	3.6024	0.98029176067567	889	621	7.2049184	11	SERPING1
**KCNE4**	1.0487	2.03506884808557	659	195	2.0974716	11	KCNE4
**MT2A.4**	3.6869	1.26608033272488	905	648	7.3738397	11	MT2A
**HLA-DPA1.3**	1.2663	-2.45142153883435	367	656	2.5326001	11	HLA-DPA1
**COL6A1.2**	4.7503	0.321837555427791	928	651	9.5007722	11	COL6A1
**PKP4.4**	6.2348	-0.537709579101536	281	576	1.2469768	11	PKP4
**RHOB.3**	7.7172	1.28119679109884	901	618	1.5434419	11	RHOB
**CD200.4**	2.6920	-0.45276130918596	258	535	5.3841691	11	CD200
**SPRY4.4**	1.5537	0.306525659305752	761	447	3.1075186	11	SPRY4
**NDUFA4L2.2**	3.2264	2.23419061130859	686	222	6.4528298	11	NDUFA4L2
**IFNG.1**	4.8679	-0.29087751599452	724	398	9.7359650	11	IFNG
**MLANA**	8.9115	0.518010411241485	505	167	1.7823069	11	MLANA

**ABCB1.1**	1.1097	-0.404299205266016	83	404	2.21956918	11	ABCB1
**DST.5**	1.4403	1.12500387330879	833	521	2.88061675	11	DST
**TWIST2.2**	5.8757	-0.429301400598353	268	585	1.17514662	11	TWIST2
**PHACTR1.2**	1.0294	-0.289234427724865	155	423	2.05897940	11	PHACTR1
**HSPA2.1**	1.2705	0.356957407917792	751	0.4	2.54119800	11	HSPA2
**ASPN.3**	8.3375	0.50467779083374	761	468	1.66750548	11	ASPN
**VWA1.3**	1.3104	-0.572654401690689	0.17	474	2.62098833	11	VWA1
**EGFL7.5**	5.5683	-0.800812571271545	124	441	1.11367933	11	EGFL7
**THSD7A.5**	6.9254	-0.600736321200448	101	427	1.38508257	11	THSD7A
**CCDC71L.1**	2.6501	0.497321521525315	781	457	5.30037420	11	CCDC71L
**MCTP1.4**	3.9515	-0.637893164109892	209	571	7.90317242	11	MCTP1
**PPP1R14A**	9.0877	1.50394888986886	675	343	1.81754020	11	PPP1R14A
**PCDH17.4**	4.3523	-0.85046297054281	266	561	8.70477578	11	PCDH17
**DES**	5.9444	0.275383870320541	633	0.31	1.18888442	11	DES
**IGKV4-1**	7.0094	-0.253350679203663	665	377	1.40189373	11	IGKV4-1
**ITGA7**	5.9018	1.37889471914824	676	369	1.18036910	11	ITGA7
**PRKCDBP.2**	1.7032	1.50129723870033	748	496	3.40658640	11	PRKCDBP
**THY1.2**	9.3469	0.594191570933879	812	484	1.86939004	11	THY1
**C2orf40**	1.8743	0.863449556683165	642	311	3.74868608	11	C2orf40
**FRZB.4**	2.5700	1.44862663785797	717	396	5.14008833	11	FRZB
**LINC01436**	6.9430	0.295622442082918	612	221	1.38860110	11	LINC01436
**RAMP1**	8.9151	1.40332908342465	649	264	1.78303167	11	RAMP1
**VWF.5**	1.5870	-1.73473072219514	245	592	3.17412521	11	VWF
**ADAM12.1**	5.5334	0.283485655882259	672	365	1.10669067	11	ADAM12
**TGFB3**	3.3863	0.267308619975603	673	349	6.77273480	11	TGFB3
**SMOC2.1**	1.0539	1.76163996859588	671	337	2.10798150	11	SMOC2
**MEG3.5**	2.1925	-0.450095465421595	777	401	4.38515962	11	MEG3
**IGFBP3.1**	2.7659	-0.475882383380051	219	488	5.53181885	11	IGFBP3
**ECSCR.1.4**	5.2387	-1.15926149370024	262	586	1.04774632	11	ECSCR.1
**MPZ**	8.7372	0.66616017464521	567	223	1.74745402	11	MPZ
**ANXA1.2**	9.8544	-1.39675041372885	561	0.82	1.97088443	11	ANXA1
**NPDC1.5**	9.9506	-0.971057358238879	299	0.57	1.99013308	11	NPDC1

**MYH11**	1.3166	1.67902527079953	672	322	2.6332482	11	MYH11
**C2CD4B.4**	3.7757	-0.710226171235616	289	547	7.5515539	11	C2CD4B
**PLCB4.1**	1.1241	0.375697711519046	694	402	2.2483892	11	PLCB4
**FAM13C.3**	7.1543	1.16148809569099	702	358	1.4308749	11	FAM13C
**SLIT3.6**	3.8373	0.893808735550892	717	0.42	7.6747567	11	SLIT3
**C10orf10.3**	8.2936	0.458946431952815	714	404	1.6587218	11	C10orf10
**SVIL.2**	1.0793	-0.418161387645952	321	572	2.1587953	11	SVIL
**LMOD1**	1.1500	0.829273202184443	578	164	2.3000427	11	LMOD1
**LAMA3**	5.5489	0.407856354687301	616	309	1.1097915	11	LAMA3
**CHMP1B**	1.5535	-0.508638189380236	432	685	3.1071283	11	CHMP1B
**NUPR1.4**	2.6969	1.03087510436893	683	396	5.3939435	11	NUPR1
**CTSG.1**	1.0633	-0.508066434480339	173	484	2.1267903	11	CTSG
**PLXDC1.2**	1.0988	0.747821481029383	694	443	2.1977876	11	PLXDC1
**TEK.4**	1.5112	-0.351804525754857	89	456	3.0224169	11	TEK
**EDNRA.2**	8.6645	1.25170316619399	603	202	1.7329128	11	EDNRA
**ALOX5AP.1**	1.1923	-0.706874946541659	161	454	2.3846910	11	ALOX5AP
**EMCN.5**	1.3910	-1.02579849507305	255	535	2.7820116	11	EMCN
**NOSTRIN.3**	9.4559	-0.409877455419755	173	488	1.8911955	11	NOSTRIN
**CRTAM.3**	9.3720	-0.660344254460411	224	541	1.8744001	11	CRTAM
**PCDH18.3**	1.0667	0.305810749324143	655	385	2.1335867	11	PCDH18
**ITGB4.5**	2.4019	-0.445566760320382	176	442	4.8038248	11	ITGB4
**SEC11C.2**	7.0842	-0.572801770669694	299	552	1.4168541	11	SEC11C
**CCDC102B**	4.2198	1.43157578169806	0.57	271	8.4396414	11	CCDC102B
**AVPR1A**	1.7811	0.743450458294198	528	0.15	3.5623407	11	AVPR1A
**HLA-DQA1.2**	3.5056	-1.41819290429871	0.16	456	7.0112137	11	HLA-DQA1
**MIR4435-2H**	4.3073	0.309264242412816	819	0.54	8.6147318	11	MIR4435-2H
**FGL2.1**	4.8950	-0.397185397432183	763	462	9.7901600	11	FGL2
**MS4A1.3**	1.1735	-0.491904464201	672	361	2.3471655	11	MS4A1
**CTSC.1**	7.6446	0.668376693209774	817	541	1.5289377	11	CTSC
**S1PR1.5**	9.7895	-0.474162387744827	265	539	1.9579002	11	S1PR1
**MMP9.2**	3.9100	-0.297203117643493	76	416	7.8201245	11	MMP9
**LRRC15.5**	1.2488	-0.276147116063928	732	478	2.4977397	11	LRRC15

**SERPINA1.1**	1.9484	-0.265677753292831	55	368	3.8969715	11	SERPINA1
**APOD.2**	2.2044	0.86432891852978	236	0.51	4.4088691	11	APOD
**SPRR2A.1**	1.3562	-1.79855118286294	0.66	375	2.7125444	11	SPRR2A
**NKG7.1**	1.1689	-1.08303517912268	735	485	2.3379451	11	NKG7
**FAM162B**	3.8200	1.43337542229406	515	196	7.6400239	11	FAM162B
**IGLV3-1.2**	1.8417	-0.592378595005858	102	384	3.6834849	11	IGLV3-1
**FST.2**	1.3241	-0.389763942844901	199	454	2.6483472	11	FST
**ADRA2A.2**	2.1231	1.02923820857821	563	307	4.2462874	11	ADRA2A
**POU2F2.4**	4.8382	-0.514202886139238	728	466	9.6765054	11	POU2F2
**CRYAB.2**	5.9833	1.39864724809363	0.56	285	1.1966765	11	CRYAB
**SCARA5.2**	8.2027	-0.318804316133877	276	537	1.6405541	11	SCARA5
**WFDC1**	3.9646	0.90801167747437	542	264	7.9293896	11	WFDC1
**SDC1.2**	6.6097	-0.291726441758281	732	452	1.3219485	11	SDC1
**RUNDC3B.2**	3.1252	-0.261096592944693	99	364	6.2505370	11	RUNDC3B
**C3.3**	1.5018	-0.421676115853789	0.17	427	3.0036728	11	C3
**ITGB2.1**	3.7380	-0.651663380864882	758	483	7.4761185	11	ITGB2
**CPVL.1**	2.4157	-0.495321119978292	167	0.42	4.8314480	11	CPVL
**HIGD1B**	1.6594	1.06894440283409	331	76	3.3188101	11	HIGD1B
**RP11-394O4.**	1.1459	0.892840876448155	419	111	2.2919777	11	RP11-394O4.
**EGFLAM**	2.5193	0.823994581005297	429	0.14	0.0503875	11	EGFLAM
**ABCC9**	0.0049	1.01209463852709	413	68	1	11	ABCC9
**TAGLN.2**	0	5.38050653863865	1	437	0	12	TAGLN
**ACTA2.2**	0	4.87769146823095	998	362	0	12	ACTA2
**MYH11.1**	0	4.35182112368628	994	312	0	12	MYH11
**TPM2.4**	0	4.26672262485818	1	0.4	0	12	TPM2
**MYL9.4**	0	4.18973590433474	1	562	0	12	MYL9
**TPM1.6**	0	3.7389416063804	997	491	0	12	TPM1
**C11orf96.4**	0	3.37973112559991	1	656	0	12	C11orf96
**CALD1.6**	0	3.15267566144479	1	0.67	0	12	CALD1
**RGS5.2**	0	3.10028420096175	975	371	0	12	RGS5
**MCAM.4**	0	3.01760536312748	997	586	0	12	MCAM
**MFGE8.1**	0	2.94620437832731	983	476	0	12	MFGE8

**NDUFA4L2.3**	0	2.87988760731279	0.91	216	0	12	NDUFA4L2
**SOD3.6**	0	2.78524257739271	995	516	0	12	SOD3
**ADIRF.6**	0	2.75079789313613	998	654	0	12	ADIRF
**PPP1R14A.1**	0	2.72211898993485	926	336	0	12	PPP1R14A
**FILIP1L.4**	0	2.69082635629826	988	581	0	12	FILIP1L
**SPARCL1.6**	0	2.68566959106907	1	634	0	12	SPARCL1
**NOTCH3.3**	0	2.5117987970375	988	365	0	12	NOTCH3
**CSRP1.1**	0	2.49188597629543	963	413	0	12	CSRP1
**IGFBP7.8**	0	2.48937929148127	1	725	0	12	IGFBP7
**MYLK.2**	0	2.44871801364248	965	536	0	12	MYLK
**FRZB.5**	0	2.4406978379891	957	389	0	12	FRZB
**TINAGL1.3**	0	2.40335705091173	0.98	552	0	12	TINAGL1
**MAP1B.5**	0	2.36621255463635	986	513	0	12	MAP1B
**NR2F2.4**	0	2.18426586325818	983	522	0	12	NR2F2
**SMOC2.2**	0	2.14796706584293	913	0.33	0	12	SMOC2
**RCAN2.1**	0	2.09667965941897	936	334	0	12	RCAN2
**PDGFA.1**	0	2.08386079404407	949	435	0	12	PDGFA
**CNN1.1**	0	1.88656433058581	884	209	0	12	CNN1
**SLC7A2.5**	0	1.80111885465821	926	245	0	12	SLC7A2
**SLIT3.7**	0	1.79908942953321	963	413	0	12	SLIT3
**DES.1**	0	1.18748846532331	861	303	0	12	DES
**AVPR1A.1**	0	1.09849020365945	784	143	0	12	AVPR1A
**SCIN**	0	0.567302455909224	858	198	0	12	SCIN
**ACAN**	0	0.446229428602059	818	201	0	12	ACAN
**LMOD1.1**	6.9808	2.0197657174929	862	155	1.3961660	12	LMOD1
**FBXO32.2**	1.0832	1.79195888582709	934	444	2.1664766	12	FBXO32
**PPP1R12B.1**	5.0461	2.20627282606873	0.92	367	1.0092377	12	PPP1R12B
**LBH.1**	2.3848	2.06127358994665	966	504	4.7696775	12	LBH
**CRISPLD2.4**	1.0258	1.9997900920886	968	559	2.0516901	12	CRISPLD2
**ID4.4**	2.0006	2.33964501350307	942	436	4.0013235	12	ID4
**PLAC9.2**	3.5063	2.0871965576831	945	486	7.0127927	12	PLAC9
**NEXN.1**	2.9104	2.01302846332181	887	392	5.8209324	12	NEXN

**PRKCDBP.3**	4.4240	2.06730836371071	946	0.49	8.8481399	12	PRKCDBP
**CPM.1**	5.6959	1.65135612501992	914	505	1.1391984	12	CPM
**SYNPO2.3**	2.4921	2.04312018451385	887	393	4.9842501	12	SYNPO2
**S100A4.4**	8.1964	1.75662514938522	1	743	1.6392808	12	S100A4
**COL14A1.6**	6.3233	1.7441188885361	989	633	1.2646640	12	COL14A1
**HES4**	7.3200	2.01333835007805	913	501	1.4640094	12	HES4
**NTN4**	1.8550	1.5539613989298	0.87	309	3.7101804	12	NTN4
**THBS1.1**	1.0498	1.71867091749227	953	584	2.0997289	12	THBS1
**MT1A.1**	1.3381	1.14898535855172	851	365	2.6763721	12	MT1A
**JAG1.3**	1.4040	1.82295911538703	943	544	2.8081419	12	JAG1
**WFDC1.1**	4.2481	1.74864718386734	835	255	8.4962802	12	WFDC1
**INHBA.5**	1.5942	0.892535239486812	876	411	3.1885291	12	INHBA
**ADAMTS1.2**	1.1671	1.92228398388498	982	677	2.3343429	12	ADAMTS1
**NET1.1**	7.3711	1.75306434792349	887	421	1.4742291	12	NET1
**TBX2.2**	1.1562	1.80739349224454	868	0.43	2.3124951	12	TBX2
**MGP.3**	2.5628	1.47730397332635	977	577	5.1256855	12	MGP
**LINC01436.1**	2.8423	0.446187135358808	781	217	5.6846179	12	LINC01436
**BGN.3**	3.1658	1.44257398080281	963	604	6.3316375	12	BGN
**BCAM.4**	5.0658	1.63066251877893	933	508	1.0131749	12	BCAM
**ENAH.2**	2.3665	1.63140556891484	913	0.46	4.7330385	12	ENAH
**CRYAB.3**	3.5075	1.82307112372584	848	276	7.0151560	12	CRYAB
**PCDH17.5**	1.0313	-0.897764320567565	123	565	2.0626336	12	PCDH17
**CCL8**	5.5197	0.909518476058567	822	441	1.1039550	12	CCL8
**CPE.3**	1.1428	1.70148145651915	0.9	511	2.2856290	12	CPE
**ITGA7.1**	7.9455	1.69812917503929	821	365	1.5891193	12	ITGA7
**FBLIM1.2**	8.5636	1.00076480507928	874	477	1.7127284	12	FBLIM1
**SDC2.7**	1.3774	1.39616692550877	864	352	2.7548569	12	SDC2
**PDGFRB.5**	3.8370	1.64367293760036	916	0.55	7.6740395	12	PDGFRB
**S1PR3.3**	6.2024	0.406804468206351	804	254	1.2404845	12	S1PR3
**ID3**	6.1920	1.7664092617551	974	661	1.2384192	12	ID3
**ADAMTS4.3**	5.0319	1.2477757187957	887	541	1.0063922	12	ADAMTS4
**SSTR2**	1.4879	0.545347587520576	816	0.39	2.9758958	12	SSTR2

**CAV1.6**	2.1319	1.31973250436122	991	641	4.2639777	12	CAV1
**GPX3.2**	8.5428	0.914234173563792	873	469	1.7085647	12	GPX3
**CSRP2.2**	2.7879	1.78547623590775	862	0.4	5.5759619	12	CSRP2
**SGIP1.3**	7.5001	0.788121386276529	884	489	1.5000230	12	SGIP1
**AKAP12.6**	4.7703	1.18050116269516	925	577	9.5407839	12	AKAP12
**RAMP1.1**	2.9453	1.88303228037115	813	259	5.8907886	12	RAMP1
**MT1M.6**	1.6437	1.90487499741251	845	388	3.2874023	12	MT1M
**IL6**	1.1042	1.51712502430921	919	0.66	2.2084796	12	IL6
**RHOB.4**	3.7193	1.70130423084458	959	617	7.4387836	12	RHOB
**PRRX1.5**	3.5088	1.38433294092788	937	505	7.0177241	12	PRRX1
**PALLD.3**	1.2306	1.48819239032959	888	511	2.4613836	12	PALLD
**EPAS1.5**	3.8786	1.4170320917362	0.98	707	7.7573404	12	EPAS1
**DKK3.2**	9.6667	1.05247378751733	893	539	1.9333477	12	DKK 3
**TGFBI.5**	1.5480	0.933151511119669	859	472	3.0960168	12	TGFBI
**TNFRSF12A.**	2.9153	0.828908535609062	905	0.61	5.8306490	12	TNFRSF12A
**TNC.6**	8.1140	0.353252950856281	842	404	1.6228067	12	TNC
**TIMP3.4**	2.1064	1.09226270405095	959	653	4.2129547	12	TIMP3
**C2orf40.1**	8.2396	1.26690849859354	778	308	1.6479301	12	C2orf40
**ACKR3.1**	3.4225	0.835884765056028	0.9	603	6.8451544	12	ACKR3
**AGT.1**	4.0626	0.375762751388901	727	278	8.1252836	12	AGT
**AOC3**	7.0121	1.45674326876845	787	318	1.4024245	12	AOC3
**GUCY1A2.1**	4.0517	0.75973975417585	721	207	8.1035546	12	GUCY1A2
**RGS16.2**	2.7668	1.634102723313	948	657	5.5336283	12	RGS16
**EDNRA.3**	1.6529	1.36918406691842	0.75	199	3.3058363	12	EDNRA
**ANTXR1.3**	1.6039	0.509361537221515	871	513	3.2078662	12	ANTXR1
**MRVI1.1**	5.6196	1.61495143868587	778	372	1.1239288	12	MRVI1
**FN1.4**	9.0977	0.880785375929021	916	592	1.8195479	12	FN1
**BCYRN1**	9.5745	0.374833072309817	801	429	1.9149051	12	BCYRN1
**ACTG2**	7.3614	1.30227048304238	691	171	1.4722961	12	ACTG2
**GEM**	3.8134	0.812325457198996	871	583	7.6269659	12	GEM
**NNMT.5**	2.6474	1.12589906544346	982	724	5.2949437	12	NNMT
**A2M.6**	1.2629	0.804401776142893	985	647	2.5259744	12	A2M

**IL33.5**	1.5023	-0.535976874580766	47	428	3.0047196	12	IL33
**SORBS2.2**	3.3317	1.85699483659855	778	381	6.6635744	12	SORBS2
**SPON1.3**	5.4611	-0.386177843329081	0.1	522	1.0922319	12	SPON1
**TPPP3**	2.1731	1.15246878335445	764	415	4.3463082	12	TPPP3
**TM4SF18.2**	1.3341	-0.602178949001607	126	494	2.6682408	12	TM4SF18
**PLAU.4**	1.7361	0.516251955809978	832	453	3.4722517	12	PLAU
**COL15A1.2**	7.9106	-1.90246496395255	387	675	1.5821291	12	COL15A1
**CEBPD.7**	8.6723	1.16791625610065	975	616	1.7344651	12	CEBPD
**LTBP1.1**	1.8574	1.3734526325989	0.83	566	3.7148510	12	LTBP1
**MYC.1**	7.8527	1.02024851580128	838	0.47	1.5705454	12	MYC
**FAM107A.6**	8.8333	-0.580961436798413	84	0.48	1.7666717	12	FAM107A
**SULF1**	2.6496	0.370042847247676	809	528	5.2993519	12	SULF1
**APOLD1.2**	3.7248	0.6315008980314	904	634	7.4497310	12	APOLD1
**MSX1**	6.2821	1.01704438052198	861	549	1.2564384	12	MSX1
**KRT18.1**	1.9569	0.782940826474826	802	437	3.9138587	12	KRT18
**CLDN5.6**	2.8777	-1.47300143335198	81	456	5.7555264	12	CLDN5
**CASQ2**	7.2291	1.28143004069417	658	108	1.4458239	12	CASQ2
**CLEC14A.4**	1.0755	-1.14274809737388	139	544	2.1510769	12	CLEC14A
**INPP4B.1**	1.2464	1.35877321875199	769	382	2.4928486	12	INPP4B
**GUCY1A3**	2.4069	1.32141558072993	758	424	4.8139033	12	GUCY1A3
**DST.6**	3.7505	0.948412045205113	885	0.52	7.5010325	12	DST
**GPR183.1**	1.1459	-1.3391192291641	124	559	2.2918575	12	GPR183
**KCNN3.1**	1.3113	-0.426425230957193	55	539	2.6227453	12	KCNN3
**MT2A.5**	5.5654	1.1566036965183	954	647	1.1130979	12	MT2A
**PHLDA1.2**	2.8780	1.03422295699217	0.94	654	5.7560671	12	PHLDA1
**PHLDA2.3**	3.1049	1.5970756172108	781	528	6.2098169	12	PHLDA2
**HOPX**	7.0867	1.10486314994966	767	317	1.4173501	12	HOPX
**FILIP1.2**	5.1216	1.3337272396399	758	331	1.0243245	12	FILIP1
**SERPING1.5**	1.6939	0.793795982827301	931	0.62	3.3879022	12	SERPING1
**AEBP1.2**	3.8101	0.387863247147717	931	664	7.6202847	12	AEBP1
**PLVAP.4**	1.9354	-1.57465642282922	288	0.59	3.8709391	12	PLVAP
**HLA-DPA1.4**	2.2598	-2.68096125523783	338	657	4.5197662	12	HLA-DPA1

**CCND1.2**	3.1133	1.2949627228202	825	541	6.22663358	12	CCND1
**CARMN**	3.3071	1.20285854587355	712	296	6.61422055	12	CARMN
**PDE5A**	6.0284	1.42967180205971	724	247	1.20568300	12	PDE5A
**ITGA6.6**	9.5218	-1.46648220723278	337	631	1.90436821	12	ITGA6
**IGKV3-15**	1.8692	-0.307907654079079	671	253	3.73850200	12	IGKV3-15
**ZNF503.3**	3.1441	0.68225161257869	828	445	6.28829290	12	ZNF503
**NTRK2.4**	3.7578	1.12779285725286	746	335	7.51578561	12	NTRK2
**NUPR1.5**	5.9447	1.19841089692459	792	394	1.18894879	12	NUPR1
**KCNE4.1**	9.8344	1.3751148136243	695	195	1.96688712	12	KCNE4
**HLA-DRB1.5**	5.0908	-2.35851189558923	0.41	683	1.01816083	12	HLA-DRB1
**RAMP2.5**	7.3986	-1.55467304708937	0.15	496	1.47972447	12	RAMP2
**PLN**	2.1169	1.51094645157579	671	98	4.23388509	12	PLN
**MOXD1.2**	5.4896	-0.319275562375012	35	409	1.09792173	12	MOXD1
**BARX1**	1.4124	0.261138020689288	721	336	2.82485914	12	BARX1
**LEPR.1**	1.8795	-0.776557353032202	283	577	3.75904664	12	LEPR
**CEBPB.3**	2.9470	0.579012291456414	959	635	5.89416153	12	CEBPB
**ADRA2A.3**	1.0909	1.26383363985035	0.72	302	2.18196731	12	ADRA2A
**C10orf10.4**	2.4885	0.623230068738882	787	402	4.97717162	12	C10orf10
**TWIST2.3**	4.4330	-0.538067720538028	271	585	8.86609380	12	TWIST2
**PLCE1**	5.7489	1.24945632103328	708	331	1.14979983	12	PLCE1
**RAI14.3**	6.4487	-0.827168571035294	352	631	1.28975801	12	RAI14
**HSPB1.4**	3.7733	0.960771717962394	914	607	7.54672300	12	HSPB1
**FAP.1**	9.2211	-0.306295356473664	32	389	1.84422447	12	FAP
**CRIM1.4**	7.1688	0.631529989731135	0.91	638	1.43376325	12	CRIM1
**ENPEP.4**	1.9235	0.432621992509642	757	0.42	3.84715971	12	ENPEP
**ELOVL7.6**	2.1385	-0.340167044131839	57	347	4.27709233	12	ELOVL7
**CCL2.2**	3.4294	0.839232466357352	0.89	635	6.85889890	12	CCL2
**PDE3A**	6.2557	1.17491027367679	669	187	1.25114500	12	PDE3A
**CD52.2**	9.1158	-1.56872007781479	337	591	1.82316961	12	CD52
**STEAP2.4**	3.8745	-0.355247066304235	173	491	7.74909582	12	STEAP2
**MT1X**	5.5247	0.629950495708404	858	511	1.10495345	12	MT1X
**CRYBG3.3**	5.5890	-0.445410065094812	248	563	1.11781147	12	CRYBG3

**AIF1.1**	9.5333	-0.782234154140424	83	401	1.90667045		12	AIF1
**FADS1.2**	3.8832	0.276522410039671	799	488	7.76656140		12	FADS1
**EREG.3**	1.0748	-0.755830378875753	775	491	2.14966013		12	EREG
**ITGB4.6**	2.4286	-0.547007121564936	129	443	4.85726619		12	ITGB4
**INHBB.2**	2.1060	-0.325841715213395	75	0.55	4.21200343		12	INHBB
**ALPL.3**	1.8638	-0.469581256112042	51	521	3.72778558		12	ALPL
**UCP2.1**	8.0496	-0.789871310454695	217	508	1.60992615		12	UCP2
**TPSB2.1**	3.2054	-1.91278870974241	769	0.48	6.41082158		12	TPSB2
**RORB.3**	1.5127	-0.521184422799322	201	457	3.02558984		12	RORB
**TSPAN7.3**	1.2313	-0.746534245598671	141	481	2.46261053		12	TSPAN7
**SEPT4**	1.0857	1.38421375531347	0.68	317	2.17141409		12	SEPT4
**CCL4L2.2**	1.6199	-1.35888596624671	74	548	3.23992888		12	CCL4L2
**GPRC5C**	1.2190	1.07688478052905	669	312	2.43818285		12	GPRC5C
**ASPN.4**	4.5272	-0.262558972364798	0.77	468	9.05447889		12	ASPN
**KYNU.3**	3.5611	-0.314759055874504	196	449	7.12238242		12	KYNU
**RERGL**	5.4351	0.861730068423198	608	69	1.08703567		12	RERGL
**CDH6**	8.7746	1.17842848662422	643	199	1.75493900		12	CDH6
**LBP.2**	9.2517	-0.368111120316876	83	502	1.85035618		12	LBP
**PDLIM4.3**	1.1481	-0.29873037824403	64	462	2.29622148		12	PDLIM4
**GZMH.2**	4.3879	-0.455625324824545	28	502	8.77593655		12	GZMH
**GSN.5**	1.6695	0.350050705017041	942	687	3.33908025		12	GSN
**FYB.2**	2.6120	-1.37017377290311	311	594	5.22408207		12	FYB
**SLC38A11**	5.1294	0.938337070214592	613	129	1.02589913		12	SLC38A11
**EMP2.3**	4.4309	0.542604673345267	838	518	8.86183593		12	EMP2
**HSPA2.2**	1.1213	0.315512257716488	721	401	2.24276893		12	HSPA2
**CSF3.4**	1.2335	-0.53956014861677	273	536	2.46705650		12	CSF3
**STEAP4.4**	1.8064	1.56799248075697	686	402	3.61284805		12	STEAP4
**CDH5.2**	3.1862	-0.873390161115266	352	619	6.37248779		12	CDH5
**HMCN1.2**	4.3501	-0.307815394840992	11	487	8.70029512		12	HMCN1
**TSC22D1.3**	1.8832	0.530488920958105	0.91	652	3.76645320		12	TSC22D1
**S100A16.5**	8.6928	-1.01766639843004	234	521	1.73857282		12	S100A16
**PTGR1.1**	1.3470	0.464764686200619	708	376	2.69406615		12	PTGR1

**NTM.1**	6.7254	-0.313853654503349	3	0.33	1.3450836	12	NTM
**PRDX4.5**	6.4464	-0.723544475830703	263	532	1.2892994	12	PRDX4
**SERPINF1.5**	1.4846	0.522467806943348	824	553	2.9692118	12	SERPINF1
**TNFRSF9.1**	2.8146	-0.344862845326044	303	573	5.6293872	12	TNFRSF9
**PDK4**	2.8435	0.286832111632143	648	333	5.6871291	12	PDK4
**CTLA4.1**	2.1187	-0.416638779564163	0.04	478	4.2374927	12	CTLA4
**SERPINE2.2**	3.4180	-0.343505061211898	52	457	6.8361909	12	SERPINE2
**GJA4.1**	9.6822	1.10263088562672	662	303	1.9364498	12	GJA4
**LSP1.2**	9.8202	-0.917130688047398	142	529	1.9640590	12	LSP1
**COX4I2**	3.5451	1.21419461355825	634	243	7.0903153	12	COX4I2
**OMD.4**	5.8169	-0.385138584914753	717	409	1.1633844	12	OMD
**FLT1.4**	4.9580	-0.881536671272102	234	505	9.9160772	12	FLT1
**FAT1.6**	2.9783	1.01707031201168	677	371	5.9566564	12	FAT1
**AQP3**	3.1900	-0.507726599027211	132	419	6.3801570	12	AQP3
**NOSTRIN.4**	3.9684	-0.43077488695659	55	491	7.9369330	12	NOSTRIN
**NUAK1.4**	6.4319	-0.306207356814794	776	455	1.2863871	12	NUAK1
**CD9.7**	1.7778	0.5110673621209	0.92	654	3.5556134	12	CD9
**TEK.5**	2.6452	-0.359497251802338	8	458	5.2904636	12	TEK
**IGFBP4.6**	4.2622	0.271127010269122	969	719	8.5245032	12	IGFBP4
**PDLIM3**	7.3605	1.06648237059841	665	359	1.4721125	12	PDLIM3
**EGFL7.6**	1.9531	-0.819436467346807	149	0.44	3.9063929	12	EGFL7
**NRGN**	5.9036	1.21334525045804	654	382	1.1807361	12	NRGN
**BICC1.2**	4.7507	-0.485186587443862	0.11	513	9.5014358	12	BICC1
**GUCY1B3**	1.0805	1.12292151456139	639	371	2.1610659	12	GUCY1B3
**CCL3L3.1**	7.2468	-0.251102688076194	9	435	1.4493767	12	CCL3L3
**PLEK.2**	1.4583	-0.539047546719636	127	418	2.9167122	12	PLEK
**LST1.1**	3.1087	-0.412796405630117	17	0.3	6.2174494	12	LST1
**MMRN1.3**	3.5901	-0.376503573918485	695	397	7.1803558	12	MMRN1
**ADAMTS2.5**	7.6979	-0.336835003813335	744	471	1.5395964	12	ADAMTS2
**ENC1**	1.6031	-0.287569618477001	145	525	3.2063606	12	ENC1
**KRT17.1**	2.1861	-0.505310810610718	691	343	4.3722208	12	KRT17
**XCL2.2**	3.7384	-0.372851370933017	11	424	7.4768370	12	XCL2

**BATF**	3.6890	-0.679567051543121	254	522	7.3780827	12	BATF
**AOAH.3**	5.0769	-0.278009075253981	5	438	1.0153852	12	AOAH
**ERG.3**	1.9876	-0.451642962366194	152	451	3.9753415	12	ERG
**SLCO2B1.2**	3.5910	-0.257550672011735	26	466	7.1821553	12	SLCO2B1
**HTRA3.2**	3.1572	-0.269277583112561	106	393	6.3145133	12	HTRA3
**CORO1A**	8.3686	-1.15043449577899	159	514	1.6737262	12	CORO1A
**JAML.3**	1.0778	-0.467860186973168	93	454	2.1556177	12	JAML
**MECOM.5**	2.2717	-0.312534080261777	0.06	417	4.5434530	12	MECOM
**CYBB.3**	7.5386	-0.352759062198576	631	378	1.5077248	12	CYBB
**CPVL.2**	4.4629	-0.506635933325251	12	425	8.9259305	12	CPVL
**THSD7A.6**	6.6708	-0.643140119986859	57	428	1.3341764	12	THSD7A
**FMO3.2**	8.8128	1.01616436138873	625	309	1.7625774	12	FMO3
**CHI3L2.2**	5.6798	-0.489167312130446	602	319	1.1359725	12	CHI3L2
**SERPINA1.2**	1.0127	-0.260158875813468	0.02	368	2.0254847	12	SERPINA1
**IGKV1-5**	1.2681	-0.327728314670924	688	363	2.5362266	12	IGKV1-5
**IGLV3-1.3**	4.2745	-0.757625790959235	14	386	8.5490982	12	IGLV3-1
**MMP9.3**	4.5017	-0.300448302545767	0.02	417	9.0034671	12	MMP9
**ABCB1.2**	6.0111	-0.404202186071096	8	405	1.2022342	12	ABCB1
**ADM5.3**	9.4530	-0.372224656980086	145	453	1.8906073	12	ADM5
**PALMD.5**	9.8877	-0.665214941991003	816	0.54	1.9775497	12	PALMD
**BCL2A1.2**	1.0325	-0.482099401833247	173	438	2.0650182	12	BCL2A1
**KDR.3**	3.8319	-0.529540521834583	78	468	7.6638026	12	KDR
**ADAP2.1**	1.2344	0.78896981104093	609	358	2.4688734	12	ADAP2
**NEGR1.5**	1.2487	-0.250991512084272	31	337	2.4975005	12	NEGR1
**FST.3**	1.8729	-0.453079884448105	123	456	3.7458718	12	FST
**CXorf36.3**	3.5387	-0.497066852210426	55	471	7.0775863	12	CXorf36
**HPGD.2**	4.2831	-0.543711410195242	583	309	8.5662821	12	HPGD
**C1QC.3**	3.8661	-0.291654144312114	632	362	7.7323597	12	C1QC
**IGKV1-12**	4.3053	-0.477729715917376	66	397	8.6107412	12	IGKV1-12
**MFAP2.4**	9.7753	-0.274776817164512	26	387	1.9550700	12	MFAP2
**CRABP1.6**	8.3286	-0.476144560967953	8	325	1.6657317	12	CRABP1
**JSRP1.1**	7.0824	-0.286074338842045	225	495	1.4164841	12	JSRP1

**CCL21**	2.7124	-1.39335149333142	711	448	5.4248138	12	CCL21
**S100B.2**	3.8047	-0.333154121542452	38	298	7.6094852	12	S100B
**ACP5.2**	7.5159	-0.282510153919544	23	391	1.5031801	12	ACP5
**MPZL2.6**	1.5677	-0.46472841739054	167	444	3.1354441	12	MPZL2
**CH25H.3**	1.1014	0.376418354512017	0.62	359	2.2029802	12	CH25H
**DNASE1L3.4**	1.3154	-0.73275096103559	126	404	2.6309902	12	DNASE1L3
**PREX2.1**	6.6411	-0.492343647287221	0.15	438	1.3282275	12	PREX2
**LTBP4.2**	1.3884	-0.277920327102147	706	434	2.7768289	12	LTBP4
**PLAC8.1**	3.3177	-0.338515306780935	23	335	6.6355609	12	PLAC8
**CP.3**	2.2342	-0.334473075009293	179	465	4.4684841	12	CP
**IGLV1-47**	1.9195	-0.267418297736357	8	288	3.8390256	12	IGLV1-47
**HMOX1.2**	1.7995	-0.272631723223199	162	468	3.5990183	12	HMOX1
**EPSTI1.4**	1.7757	-0.370427868433822	155	441	3.5515647	12	EPSTI1
**KLRD1**	9.4445	-0.290653216484144	5	359	1.8889151	12	KLRD1
**CD84.1**	4.4293	-0.44982264413484	801	493	8.8587776	12	CD84
**MME.2**	1.1084	-0.468818419512175	0.13	421	2.2168451	12	MME
**POU2AF1.1**	2.3969	-0.259366077664361	789	518	4.7938149	12	POU2AF1
**LGALS2.2**	4.7008	-0.300105342586359	21	305	9.4016734	12	LGALS2
**IGLL5.1**	5.0185	-0.985924071099885	66	333	1.0037125	12	IGLL5
**IGLV1-51.1**	1.2479	-0.458447373902178	0	285	2.4958730	12	IGLV1-51
**KCNAB1**	9.9281	0.796891577936918	511	252	1.9856228	12	KCNAB1
**SPON2.3**	9.2155	-0.390251116401304	677	426	1.8431073	12	SPON2
**HHEX.1**	1.4067	-0.269744598984511	67	347	2.8134356	12	HHEX
**POU2F2.5**	2.5112	-0.603113435028041	732	467	5.0224639	12	POU2F2
**CLMP.3**	8.8636	-0.255271826841109	11	298	1.7727231	12	CLMP
**PID1.5**	9.9371	-0.287426669545138	12	297	1.9874263	12	PID1
**RP11-394O4.**	1.5336	0.722231801417191	466	0.11	3.0673897	12	RP11-394O4.
**IGHGP.1**	3.0860	-0.707948168501391	41	338	0.0006172	12	IGHGP
**KLRB1.1**	5.1398	-0.788414148301434	55	311	0.0010279	12	KLRB1
**RP11-91J3.3**	3.0063	0.438924243215772	383	54	0.0060127	12	RP11-91J3.3
**FOXS1**	0.0001	0.555086879484904	381	85	0.2036749	12	FOXS1
**HIGD1B.1**	0.0068	0.870716020337377	395	74	1	12	HIGD1B

**ASPM**	8.2952	0.839441219546294	828	179	1.6590504	13	ASPM
**TROAP**	2.9597	0.380765892904576	722	184	5.9195781	13	TROAP
**HIST1H1B**	1.3179	0.730046723478646	839	299	2.6358049	13	HIST1H1B
**KRT5.1**	1.9508	2.71225922869509	867	402	3.9016206	13	KRT5
**RORB.4**	1.0094	-0.537808911102965	85	458	2.0189212	13	RORB
**CASC5**	4.3515	0.406111033043935	833	329	8.7031399	13	CASC5
**CTLA4.2**	1.8593	0.308908234182588	909	454	3.7186905	13	CTLA4
**ITGBL1.4**	8.2969	-0.674621507266653	74	361	1.6593982	13	ITGBL1
**KRT14.1**	2.7674	2.77211138090838	869	0.49	5.5348880	13	KRT14
**ZWINT**	8.8058	0.554523328955859	0.85	0.39	1.7611637	13	ZWINT
**SERPINB4**	1.4935	0.642915210879506	725	216	2.9870486	13	SERPINB4
**PLOD2.3**	5.3824	-0.372583633638325	125	501	1.0764989	13	PLOD2
**STEAP1.4**	8.1398	-0.400041192639764	123	425	1.6279741	13	STEAP1
**KIF11**	1.1995	0.407708191746952	792	318	2.3991067	13	KIF11
**MRC2.4**	2.6279	-0.516831128019254	95	446	5.2559273	13	MRC2
**NCAPG**	1.7999	0.358590156852537	657	113	3.5998718	13	NCAPG
**ALDH3A1**	4.6458	1.47240522229174	763	236	9.2916374	13	ALDH3A1
**IL2RA**	3.9787	0.257578490269689	839	0.41	7.9574653	13	IL2RA
**FABP5.1**	9.4909	2.63711335702659	862	489	1.8981930	13	FABP5
**TNFRSF18**	1.5305	0.499805472839583	0.9	505	3.0611349	13	TNFRSF18
**S100A2.1**	5.6810	2.6954280200418	805	388	1.1362105	13	S100A2
**OMD.5**	1.3371	-0.46415359530768	119	425	2.6743155	13	OMD
**CCNB1**	1.7231	0.613786256258356	773	321	3.4463642	13	CCNB1
**HMMR**	4.1551	0.410399264682709	653	156	8.3103572	13	HMMR
**SPON2.4**	2.7790	-0.260969810037692	148	0.44	5.5581679	13	SPON2
**CDCA3**	5.4316	0.363643719878333	699	216	1.0863316	13	CDCA3
**MMP2.2**	1.1276	-1.67947587597863	0.25	645	2.2553870	13	MMP2
**ENAH.3**	1.4150	-0.545133724484158	146	481	2.8301090	13	ENAH
**ANK2.3**	1.5695	-0.26029619332192	59	318	3.1390735	13	ANK2
**CENPA**	2.1833	0.315269911643424	691	267	4.3666751	13	CENPA
**VWA1.4**	3.3707	-0.589743780414135	142	471	6.7414977	13	VWA1
**CDC20**	1.9105	0.390870174124575	0.71	208	3.8211356	13	CDC20

**TMEM176B.5**	2.4500	-0.844821788624587	167	506	4.9001116	13	TMEM176B
**IGLV6-57**	2.3756	-0.319663253663841	835	476	4.7512396	13	IGLV6-57
**SPRY1.3**	3.9625	-1.24434682741389	203	604	7.9251705	13	SPRY1
**FRMD6.4**	2.3437	-0.436430653593778	144	399	4.6874553	13	FRMD6
**CD7.1**	5.3351	0.43265484167731	894	541	1.0670324	13	CD7
**ZNF503.4**	6.2790	-0.261793040540304	155	464	1.2558158	13	ZNF503
**CALU.4**	2.7003	-0.954581037768659	267	0.63	5.4007075	13	CALU
**SERPINB3**	7.3434	0.706908836801576	737	316	1.4686852	13	SERPINB3
**IGKV4-1.1**	4.1860	-0.324174368568526	809	377	8.3721642	13	IGKV4-1
**SGIP1.4**	5.4217	-0.378861638310362	216	508	1.0843491	13	SGIP1
**CLU.2**	9.1556	-1.50184954796263	284	626	1.8311243	13	CLU
**KRT17.2**	1.5310	0.968969701293713	752	345	3.0620965	13	KRT17
**CCNB2**	1.7869	0.425507722647344	725	331	3.5738415	13	CCNB2
**EGR3**	5.4603	-0.674605479359792	369	673	1.0920622	13	EGR3
**CP.4**	3.6752	-0.33954537090754	178	462	7.3505198	13	CP
**FILIP1L.5**	3.7820	-1.3640599094224	269	601	7.5640230	13	FILIP1L
**RCAN1.2**	4.6097	-0.661547994103635	237	537	9.2195360	13	RCAN1
**SFN.1**	7.9388	2.71852543936664	737	291	1.5877742	13	SFN
**F13A1.3**	4.9854	-0.379269378855882	167	422	9.9708738	13	F13A1
**TIMP1.5**	3.1236	-1.89126223781306	428	719	6.2473812	13	TIMP1
**ITGB4.7**	8.0400	-0.359079232971021	186	439	1.6080081	13	ITGB4
**SEMA5A.2**	6.5414	-0.258622335593658	51	348	1.3082803	13	SEMA5A
**CENPF**	1.6163	1.32610429949694	805	467	3.2326448	13	CENPF
**C1R.3**	8.9874	-2.06187059355397	428	694	1.7974961	13	C1R
**KRT6A.1**	1.6343	2.57476147554079	746	381	3.2687636	13	KRT6A
**ROBO1.3**	3.5401	-0.304042527213999	233	534	7.0803649	13	ROBO1
**LRP1.2**	2.9225	-0.686046994737554	271	557	5.8450940	13	LRP1
**RHOB.5**	6.1291	-0.906898431948614	331	635	1.2258221	13	RHOB
**EGR1.1**	2.1615	-1.41675442796595	489	801	4.3230027	13	EGR1
**SVIL.3**	1.9378	-0.625737813248373	271	571	3.8756906	13	SVIL
**PGF.2**	2.7966	-0.339453823377849	235	501	5.5932863	13	PGF
**FBLN2.4**	3.2953	-1.39396609258092	239	537	6.5906841	13	FBLN2

**HES1.3**	4.3810	-0.950724903362736	345	668	8.7620957	13	HES1
**CRYBG3.4**	1.4976	-0.60378106192057	286	559	2.9952781	13	CRYBG3
**KIF23**	1.6416	0.30733364260578	708	268	3.2832462	13	KIF23
**AKR1B10**	1.1813	0.852266723647478	678	0.25	2.3626743	13	AKR1B10
**GBP1.1**	2.4300	-0.368148059030363	282	576	4.8600154	13	GBP 1
**BUB1**	2.4548	0.274311131371487	706	265	4.9097030	13	BUB1
**HN1**	4.9666	1.1296377073467	892	508	9.9332967	13	HN1
**FBLN5.3**	1.6113	-0.463055669229588	142	427	3.2227735	13	FBLN5
**IGLV3-21.3**	1.1038	-0.277822518626021	708	337	2.2077203	13	IGLV3-21
**RBP1**	2.3749	0.42592253625893	742	373	4.7499669	13	RBP1
**PTGDS.3**	1.1165	-1.89725308495768	288	0.56	2.2331756	13	PTGDS
**HIST1H1C**	1.9955	0.700434533171082	845	426	3.9911965	13	HIST1H1C
**HES2**	1.2372	0.307840441831848	638	252	2.4745153	13	HES2
**TGFBR3.1**	1.9990	-0.579411332114167	328	582	3.9981719	13	TGFBR3
**NUSAP1**	8.0366	1.0036660248311	729	345	1.6073351	13	NUSAP1
**CA2**	1.4503	0.337388021810411	697	375	2.9006875	13	CA2
**CEP55**	2.4328	0.481612812248035	593	0.14	4.8656801	13	CEP55
**DCN.2**	2.9956	-2.73462107950278	356	675	5.9913204	13	DCN
**HMGB2.1**	3.7770	1.85766595662498	0.86	478	7.5540942	13	HMGB2
**IL1B.1**	7.1262	-0.570070482849923	0.72	381	1.4252513	13	IL1B
**RHOBTB3.4**	2.6523	-0.312486754005896	133	422	5.3047418	13	RHOBTB3
**CLCA2**	1.4058	0.709000682944088	627	155	2.8117333	13	CLCA2
**PHLDA2.4**	2.0004	0.740016916398213	822	529	4.0009449	13	PHLDA2
**MZB1.2**	9.9497	-0.994809474869387	873	617	1.9899585	13	MZB1
**XCL2.3**	3.1626	0.341652101161685	0.75	403	6.3253738	13	XCL2
**AREG**	1.1810	-0.306430864541106	913	637	2.3621512	13	AREG
**STEAP2.5**	1.7367	-0.285093940641518	814	473	3.4734848	13	STEAP2
**APLNR.3**	6.6898	-0.380866909500733	807	464	1.3379769	13	APLNR
**HMCN1.3**	1.5913	-0.28459667487932	225	478	3.1827489	13	HMCN1
**ANKRD37**	4.4824	0.468184106619278	0.79	483	8.9648179	13	ANKRD37
**THSD7A.7**	1.5479	-0.600846151235888	131	423	3.0959332	13	THSD7A
**FGFBP1**	2.3229	0.856991293743559	629	193	4.6459793	13	FGFBP1

**FKBP10.1**	3.2408	-0.425023847790299	189	499	6.4816629	13	FKBP10
**PPL**	1.8193	0.300315012547663	684	0.28	3.6387359	13	PPL
**VASN.3**	3.7994	-0.319436512702901	0.18	485	7.5989387	13	VASN
**ANTXR1.4**	1.5834	-0.37099805062919	278	529	3.1669483	13	ANTXR1
**C12orf75.2**	6.6585	0.923745042279287	756	389	1.3317163	13	C12orf75
**SERINC2**	2.3262	0.317943783476078	646	277	4.6525838	13	SERINC2
**IRF6**	4.1330	0.433378977999989	636	249	8.2660440	13	IRF6
**EDN1.1**	4.2059	-0.353099904989312	752	344	8.4118305	13	EDN1
**CDKN3.1**	1.8969	0.537678241740987	739	393	3.7939455	13	CDKN3
**LCP1.3**	2.3527	0.362085761524875	0.89	0.57	4.7054653	13	LCP1
**CPA3.3**	4.4510	-0.663597286083517	739	364	8.9021046	13	CPA3
**KRT13.1**	1.1113	2.07067523067886	0.68	416	2.2226741	13	KRT13
**BATF.1**	1.8948	0.417476496308636	811	507	3.7896381	13	BATF
**DERL3.2**	4.3674	-0.450344821463295	0.82	0.56	8.7348910	13	DERL3
**HMGA1**	5.2415	0.647925296579678	767	464	1.0483186	13	HMGA1
**SDC1.3**	1.0349	0.601191097315483	737	455	2.0699310	13	SDC1
**TPSB2.2**	3.2106	-1.84729060437553	824	481	6.4213364	13	TPSB2
**JCHAIN**	2.5439	-0.949841608064757	786	506	5.0878143	13	JCHAIN
**MOXD1.3**	3.8911	-0.284397154477677	725	0.39	7.7823425	13	MOXD1
**PRC1**	8.1214	0.603709046764153	642	344	1.6242878	13	PRC1
**C10orf99**	1.0731	0.448619699846893	593	174	2.1462516	13	C10orf99
**UBE2C**	2.3467	0.804707593741708	0.54	92	4.6935875	13	UBE2C
**SPC25**	2.5079	0.263557644800281	504	96	5.0159645	13	SPC25
**TM4SF18.3**	2.1139	-0.46261486725877	786	475	4.2278153	13	TM4SF18
**SELP.3**	2.8587	-0.635350428446569	814	0.54	5.7174057	13	SELP
**TEK.6**	8.0197	-0.259822548512126	788	436	1.6039546	13	TEK
**UCP2.2**	5.2808	0.324104010227366	822	492	1.0561726	13	UCP2
**SPRR2D.1**	1.1924	-0.438095679677833	617	266	2.3848227	13	SPRR2D
**FSCN1.1**	1.9905	0.380271947210954	792	517	3.9811227	13	FSCN1
**LYZ.1**	8.4512	-1.7496021978176	883	594	1.6902405	13	LYZ
**CFI.4**	2.2778	-0.266707445972392	0.72	424	4.5556249	13	CFI
**S100B.3**	6.7260	0.412063562873611	633	283	1.3452101	13	S100B

**IGHG2.2**	8.4773	-1.41527869035741	831	567	1.6954785	13	IGHG2
**CLSPN**	1.3891	0.582531446816787	663	383	2.7782956	13	CLSPN
**IL33.6**	4.4508	-0.378406386224825	716	409	8.9017859	13	IL33
**FAM107A.7**	2.1968	-0.551518781987008	157	475	4.3937216	13	FAM107A
**NUF2**	2.7708	0.363731432052622	602	306	5.5416306	13	NUF2
**PHACTR1.3**	1.6619	-0.272727730022564	809	405	3.3238534	13	PHACTR1
**NOSTRIN.5**	1.8160	-0.361693507879543	765	471	3.6321570	13	NOSTRIN
**CHN1.1**	3.5305	-0.390881452220367	896	584	7.0610566	13	CHN1
**TK1**	4.5150	0.916494560213335	612	283	9.0300803	13	TK1
**VAMP8.1**	1.1437	1.05112880031949	716	399	2.2874953	13	VAMP8
**ST14**	1.2878	0.334576716317002	589	223	2.5757388	13	ST14
**FXYD3.1**	1.9119	2.00465197539514	591	237	3.8238737	13	FXYD3
**MASP1.5**	7.2962	-0.391765569860354	761	428	1.4592452	13	MASP1
**TNS4**	1.1679	0.388797483899444	606	0.29	2.3358017	13	TNS4
**GZMB.3**	1.3716	0.258777688811489	835	478	2.7432299	13	GZMB
**BIRC5**	9.9685	0.594681257999724	612	229	1.9937116	13	BIRC5
**CXorf36.4**	1.0395	-0.424821475777158	0.79	451	2.0790690	13	CXorf36
**LY6D.1**	1.1753	1.52784624442757	606	265	2.3506217	13	LY6D
**ENPP2.3**	1.6453	-0.250732955192348	841	429	3.2906621	13	ENPP2
**GATA2.3**	3.0173	-0.286626267826895	797	482	6.0346303	13	GATA2
**KLF5**	3.7465	0.940434722723459	646	396	7.4931558	13	KLF5
**EFHD2.3**	5.7166	0.374274940104875	833	565	1.1433257	13	EFHD2
**PTPRB.5**	1.2528	-0.434699040135619	799	535	2.5056044	13	PTPRB
**KRT4**	2.6831	0.302787223895676	553	201	5.3662298	13	KRT4
**SPRR1B.1**	2.6935	0.497826429268837	589	224	5.3870008	13	SPRR1B
**SCARA5.3**	1.1573	-0.41484331638563	799	522	2.3146559	13	SCARA5
**KCNN3.2**	1.8760	-0.315880235217013	803	517	3.7521346	13	KCNN3
**MPZL2.7**	2.0197	0.262177923883394	731	429	4.0395089	13	MPZL2
**KIAA0101**	4.0630	1.21738459950064	614	309	8.1260581	13	KIAA0101
**RASSF4.1**	6.3887	-0.259556937677509	756	0.42	1.2777468	13	RASSF4
**FGL2.2**	9.2746	-0.414099038204828	794	465	1.8549284	13	FGL2
**CD52.3**	2.7350	0.258601620154979	837	577	5.4700812	13	CD52

**CLEC14A.5**	3.4991	-0.9110199292799	809	525	6.9982781	13	CLEC14A
**STEAP4.5**	3.2691	-0.416463171480606	674	405	6.5383535	13	STEAP4
**RGS2.1**	5.3763	-0.271376320813065	894	613	1.0752604	13	RGS2
**IGLV3-1.4**	1.6796	-0.637709901179318	669	368	3.3593730	13	IGLV3-1
**KRT15.1**	2.0591	2.30543335393924	561	225	4.1182687	13	KRT15
**FDCSP**	6.0234	1.66391219459119	583	296	1.2046978	13	FDCSP
**BCL2A1.3**	6.4361	-0.333768198159265	684	424	1.2872258	13	BCL2A1
**CD200.5**	1.0142	-0.442898348196268	854	518	2.0284103	13	CD200
**ANGPT2.1**	1.5534	-0.256633318087882	686	0.38	3.1068268	13	ANGPT2
**S100A14.1**	4.1968	1.60720531327754	568	242	8.3936920	13	S100A14
**LGMN.1**	4.5096	-0.265317440431276	831	572	9.0193249	13	LGMN
**MME.3**	6.5202	-0.416517998584693	714	405	1.3040552	13	MME
**PCDH17.6**	1.5610	-0.788565416529383	0.82	545	3.1221894	13	PCDH17
**IGLL5.2**	1.7058	-0.473320317764399	595	318	3.4116584	13	IGLL5
**GBP 6**	2.0429	0.831754110881221	547	0.22	4.0859546	13	GBP 6
**FABP4**	3.1957	0.269143117290454	549	221	6.3914780	13	FABP4
**RCN3.3**	4.0410	-0.302055487329439	699	391	8.0821118	13	RCN3
**EMCN.6**	1.2548	-0.847516012103445	0.78	0.52	2.5097518	13	EMCN
**HLA-DQA1.3**	1.5743	-0.500309116658199	767	439	3.1486902	13	HLA-DQA1
**KIF20B**	2.0377	0.417701360519212	701	401	4.0755061	13	KIF20B
**AKR1C1.1**	6.2485	0.632048359970258	549	243	1.2497032	13	AKR1C1
**C19orf33**	7.8528	0.640112777623606	555	296	1.5705610	13	C19orf33
**HLA-DQB1.3**	1.4553	-0.528095032347981	824	566	2.9106541	13	HLA-DQB1
**MFAP2.5**	3.0914	-0.269654111240059	695	368	6.1828682	13	MFAP2
**DMKN**	3.6828	0.317558201988054	515	238	7.3656020	13	DMKN
**EGFL7.7**	2.5815	-0.552105839729882	708	424	5.1630257	13	EGFL7
**F2R.1**	3.8186	-0.497691032650473	869	584	7.6373218	13	F2R
**C2.1**	3.7581	-0.306709130919966	0.78	495	7.5162599	13	C2
**C1QB.4**	8.9446	-0.37859872241188	773	482	1.7889203	13	C1QB
**CXCL9**	6.4746	-0.427423538016975	879	601	1.2949276	13	CXCL9
**SBSN.1**	6.7264	0.445521143374268	0.5	185	1.3452989	13	SBSN
**CTSL.1**	7.5155	-0.330546336286292	0.82	0.57	1.5031018	13	CTSL

**KRT19.1**	2.7559	1.23727767307748	542	253	5.5119775(	13	KRT19
**KCNQ1OT1**	3.3738	-0.378438292682804	782	464	6.7477872	13	KCNQ1OT1
**ICAM2.2**	4.1319	-0.311847260169203	807	0.53	8.2638805(	13	ICAM2
**PTK7.2**	9.1432	-0.285744261987258	756	503	1.8286586(	13	PTK7
**IGF1.2**	1.2198	-0.41218968435324	799	501	2.4396104(	13	IGF1
**SERPINB5**	4.7163	0.619028311551226	506	241	9.4327010	13	SERPINB5
**FILIP1.3**	8.7552	-0.340653349827238	631	338	1.7510402	13	FILIP1
**VEGFA.2**	7.0786	-0.358214039025217	763	489	1.4157273	13	VEGFA
**VSNL1**	1.1749	0.841610287830774	453	148	2.3498865	13	VSNL1
**NFKBID.1**	2.8298	-0.315631251818262	871	595	5.6597351	13	NFKBID
**PDGFRB.6**	1.0432	-0.976407077608196	856	555	2.0865845	13	PDGFRB
**FAM25A**	2.8870	-0.326583821046644	373	79	5.7741752	13	FAM25A
**RHOJ.3**	7.0349	-0.429702447440679	822	0.55	0.0001406	13	RHOJ
**KLK11**	1.8755	0.410697917327745	0.46	0.12	0.0003751(	13	KLK11
**PLEKHA5.4**	3.1788	-0.331177287778756	754	484	0.0006357	13	PLEKHA5
**AURKB**	3.2573	0.540057754958675	494	216	0.0006514(	13	AURKB
**GJB3**	0.0003	0.310185814966608	352	71	0.6875041	13	GJB3
**PRRG4**	0.0004	0.589041727730808	483	206	0.8704629	13	PRRG4
**APOD.3**	0.0015	-1.42153112069164	748	495	1	13	APOD
**RRM2**	0.0025	0.756683815296337	475	171	1	13	RRM2
**IRX4**	0.0078	0.320104805960578	407	65	1	13	IRX4
**HCAR3.1**	0	0.61712707660804	952	154	0	14	HCAR3
**CLDN10**	0	0.3428266890471	947	134	0	14	CLDN10
**EPPK1**	0	0.303004476902002	921	137	0	14	EPPK1
**UBE2C.1**	3.2338	0.473642977786799	0.93	88	6.4676390	14	UBE2C
**CTSV**	1.8713	0.2644048778529	904	0.11	3.7426515	14	CTSV
**FGFBP1.1**	3.4269	1.81093836916343	983	189	6.8538646	14	FGFBP1
**ESCO2**	9.1264	0.267139880891662	941	203	1.8252807	14	ESCO2
**HAS3**	1.1785	0.418910628299063	949	301	2.3570307	14	HAS3
**SERPINB4.1**	9.8249	0.508060697053814	952	215	1.9649912(	14	SERPINB4
**ELF3**	3.0930	0.627191585928907	949	181	6.1861523	14	ELF3
**CDC20.1**	1.4419	0.28677237679559	933	207	2.8839260(	14	CDC20

**AKR1B10.1**	1.6226	1.21766881832574	972	247	3.2452232	14	AKR1B10
**KRT14.2**	9.0731	5.54698690219961	989	0.49	1.8146367	14	KRT14
**KRT17.3**	3.4241	1.70325186139891	989	343	6.8483844	14	KRT17
**SFN.2**	1.0810	3.34364987723056	986	289	2.1621284	14	SFN
**CLCA2.1**	1.0008	1.36574267635436	938	152	2.0016494	14	CLCA2
**RRM2.1**	4.0965	0.294231275360252	916	165	8.1931417	14	RRM2
**KRT5.2**	4.0337	4.56338929923083	978	403	8.0675798	14	KRT5
**SERPINB3.1**	2.7439	0.582477782915089	961	315	5.4879327	14	SERPINB3
**SERPINB5.1**	3.2776	1.40170808157598	952	235	6.5553751	14	SERPINB5
**S100A2.2**	6.5781	3.79406686136198	983	387	1.3156261	14	S100A2
**MKI67**	7.8223	0.448949966319362	935	271	1.5644632	14	MKI67
**APOBEC3A.1**	3.2110	0.250280216574576	933	361	6.4221486	14	APOBEC3A
**CA2.1**	1.0060	0.566693807267692	978	372	2.0120852	14	CA2
**CCNB1.1**	2.4692	0.568681265135574	972	0.32	4.9385400	14	CCNB1
**PPP1R14C**	1.3470	0.362484971941339	899	153	2.6940231	14	PPP1R14C
**HES2.1**	6.4979	0.53764484721351	927	0.25	1.2995871	14	HES2
**THSD4**	1.9004	0.575284296257634	972	425	3.8009368	14	THSD4
**PERP.2**	7.5948	2.76212662966502	986	464	1.5189717	14	PERP
**ST14.1**	1.2157	0.710055422989081	944	219	2.4315904	14	ST14
**KLF5.1**	2.2638	1.90391131106432	969	391	4.5276924	14	KLF5
**TOP2A**	2.1670	0.716732206474603	935	337	4.3340911	14	TOP2A
**F13A1.4**	1.2517	-0.399619458393801	14	424	2.5034177	14	F13A1
**DSP.1**	1.0016	3.14600826783196	961	331	2.0032165	14	DSP
**OMD.6**	1.1759	-0.469549507391081	28	425	2.3518919	14	OMD
**SFRP1.5**	1.2734	0.783866358965647	969	421	2.5468156	14	SFRP1
**CENPF.1**	1.6890	0.821503999211829	952	466	3.3780380	14	CENPF
**CDK1**	2.3129	0.333142638277123	904	211	4.6258970	14	CDK1
**RBP1.1**	6.7672	0.532463265409057	955	371	1.3534585	14	RBP1
**SPRR2D.2**	2.2638	-0.575470413181101	958	262	4.5276829	14	SPRR2D
**ALDH3A1.1**	2.4175	1.82387408011855	921	237	4.8350511	14	ALDH3A1
**IGKV1-5.1**	1.2991	-0.262806445740087	955	363	2.5982014	14	IGKV1-5
**CDCP1**	1.4438	0.530380244491719	874	142	2.8877377	14	CDCP1

**CD248.2**	5.3859	-0.394694887521381	53	443	1.0771905	14	CD248
**SPINT2.1**	3.1355	1.87070409975136	947	317	6.2711469	14	SPINT2
**BIRC5.1**	6.3336	0.45134532767627	0.89	226	1.2667324	14	BIRC5
**KRT6A.2**	1.1911	3.53746964549156	947	379	2.3823533	14	KRT6A
**WISP2.4**	2.3603	-0.302586114790277	59	364	4.7207708	14	WISP2
**S100A9.2**	2.3738	1.67354261592414	978	538	4.7477658	14	S100A9
**LRRC15.6**	1.6375	-0.34167168310278	0.11	493	3.2750549	14	LRRC15
**DMKN.1**	4.0673	0.51700943591531	899	233	8.1346400	14	DMKN
**EREG.4**	8.4031	-0.630945658223156	955	492	1.6806216	14	EREG
**SEMA5A.3**	2.7775	-0.311183942737816	73	346	5.5551254	14	SEMA5A
**CDH1**	2.5314	0.526456611684616	871	229	5.0629732	14	CDH1
**SERINC2.1**	3.5523	0.550306039481464	924	274	7.1047789	14	SERINC2
**COL17A1.2**	1.2399	2.70322644947148	921	316	2.4799838	14	COL17A1
**FXYD3.2**	1.7286	2.19982255943487	916	234	3.4573053	14	FXYD3
**FRZB.6**	6.7985	-0.776197386553043	51	413	1.3597093	14	FRZB
**MT1A.2**	1.2894	-0.268865818517509	76	385	2.5789871	14	MT1A
**ITGA2.2**	1.7375	0.807629974950139	955	371	3.4751879	14	ITGA2
**ANKRD37.1**	3.1594	0.701422200386024	966	482	6.3189708	14	ANKRD37
**DSC3**	1.1835	2.06134731803587	879	179	2.3670171	14	DSC3
**AKR1C1.2**	2.0775	1.2932332420464	0.91	239	4.1550511	14	AKR1C1
**CBR1**	1.8072	2.0860799348261	913	294	3.6144892	14	CBR1
**C12orf75.3**	1.5305	1.36300872138461	969	387	3.0611340	14	C12orf75
**MMP2.3**	5.2729	-1.64629981015638	98	645	1.0545932	14	MMP2
**OAS1**	7.9714	0.430739297267995	913	269	1.5942854	14	OAS1
**PRC1.1**	7.1415	0.48821412799537	902	341	1.4283093	14	PRC1
**RND3.4**	2.5918	1.64690980196586	994	552	5.1836833	14	RND3
**LAMC2.1**	1.7259	0.34945236205918	935	357	3.4519376	14	LAMC2
**FMOD.2**	3.5214	-0.915823607564255	107	578	7.0429019	14	FMOD
**ISLR.6**	1.2744	-0.434807708321146	96	383	2.5488408	14	ISLR
**FBLN5.4**	3.3975	-0.480021926051588	59	427	6.7950760	14	FBLN5
**PITX1.1**	3.5254	1.65106388978626	893	0.23	7.0509173	14	PITX1
**SLC9A3R1.1**	5.0705	0.953909264897173	944	417	1.0141073	14	SLC9A3R1

**GUCY1A3.1**	5.3508	-0.310375744963189	53	441	1.0701644	14	GUCY1A3
**FCGR2A.1**	1.3981	-0.273107205910753	67	426	2.7962937	14	FCGR2A
**LAD1**	2.0905	0.91484534069298	862	0.25	4.1811514	14	LAD1
**CXCL14.3**	2.5834	2.85471936797394	927	402	5.1668670	14	CXCL14
**TNC.7**	2.7953	0.251910440303031	955	409	5.5907441	14	TNC
**CCDC80.2**	1.6103	-1.24517995263356	81	485	3.2207113	14	CCDC80
**C19orf33.1**	2.5243	0.623777954688151	885	292	5.0486635	14	C19orf33
**ITPKC**	3.3991	0.611832568244071	961	511	6.7983808	14	ITPKC
**COL6A3.2**	4.2365	-2.35431102909518	197	681	8.4730872	14	COL6A3
**PVRL1**	2.7294	0.758936031194144	879	352	5.4588718	14	PVRL1
**IL1B.2**	3.2151	-0.618814110753634	919	0.38	6.4302964	14	IL1B
**CDS1**	5.1109	0.378630778242559	826	193	1.0221860	14	CDS1
**NPR 3**	8.9541	0.754448867722736	823	162	1.7908347	14	NPR 3
**IGLV3-21.4**	1.6340	-0.536424770517544	944	335	3.2681323	14	IGLV3-21
**SGIP1.5**	1.6743	-0.375719215826771	28	0.51	3.3487748	14	SGIP1
**PHLDA2.5**	1.1935	0.861335582591821	963	529	2.3871759	14	PHLDA2
**GPX2**	1.8772	0.575894613517163	823	232	3.7544342	14	GPX2
**NTM.2**	3.7805	-0.309545872339666	53	325	7.5610487	14	NTM
**SDC4**	8.1275	0.355272045639038	952	549	1.6255132	14	SDC4
**GJB2**	2.0026	1.05309675677134	862	252	4.0053150	14	GJB2
**FSCN1.2**	1.2457	0.75848953304099	975	516	2.4915304	14	FSCN1
**S100A8.1**	1.3428	1.02400902043886	935	502	2.6857680	14	S100A8
**FCER1G.2**	2.1395	-0.791598973478475	73	414	4.2790390	14	FCER1G
**DST.7**	5.7766	2.27940425737041	935	525	1.1553363	14	DST
**TUBA1C.1**	6.7542	1.29031191439269	997	643	1.3508502	14	TUBA1C
**CXADR**	1.0776	1.2209984172733	831	163	2.1552638	14	CXADR
**CSTB**	1.6245	0.884199148337812	983	0.62	3.2491625	14	CSTB
**CP.5**	4.8325	-0.360022054014784	152	461	9.6650752	14	CP
**FAM83B**	9.8078	0.448713087960159	767	122	1.9615797	14	FAM83B
**SLC7A5**	3.1694	0.517644888201087	949	523	6.3388329	14	SLC7A5
**C10orf99.1**	5.7895	0.344569578758008	817	173	1.1579004	14	C10orf99
**ABI3BP.4**	5.8466	-0.324681171029553	112	391	1.1693274	14	ABI3BP

**PTN.3**	1.8879	1.06972315432336	896	323	3.7759769	14	PTN
**KRT15.2**	4.6802	3.64286562060408	846	222	9.3605638	14	KRT15
**GSTP1.3**	7.4566	1.6440108867133	986	702	1.4913386	14	GSTP1
**STEAP4.6**	9.5742	-0.307449775029083	935	402	1.9148400	14	STEAP4
**IGLV6-57.1**	1.7587	-0.602813487700365	955	476	3.5174443	14	IGLV6-57
**FADS1.3**	2.5135	-0.348868359132059	0.07	505	5.0271864	14	FADS1
**MMP9.4**	1.2767	-0.319750709132194	84	0.41	2.5535838	14	MMP9
**IRF6.1**	2.1214	0.709780099698819	829	248	4.2429307	14	IRF6
**CYGB.3**	3.3587	-0.308118834739432	0.09	461	6.7174488	14	CYGB
**IGKV4-1.2**	2.0723	-0.344567473685395	947	377	4.1446346	14	IGKV4-1
**THY1.3**	5.7236	-1.38634210609224	104	502	1.1447250	14	THY1
**MYH11.2**	1.7116	-0.811572061645914	902	324	3.4232094	14	MYH11
**IGKV1-12.1**	4.5347	-0.416514332292508	947	377	9.0694350	14	IGKV1-12
**SLC2A1**	5.1439	1.16741337191945	871	506	1.0287913	14	SLC2A1
**MS4A6A.2**	2.2597	-0.345957846697753	79	471	4.5194865	14	MS4A6A
**FABP5.2**	2.9843	3.13193421679802	904	491	5.9687292	14	FABP5
**CD9.8**	9.4972	1.34258144424039	983	657	1.8994522	14	CD9
**COL11A1.5**	1.0253	-0.567146067910146	107	417	2.0506012	14	COL11A1
**THBS1.2**	1.6954	-0.785821211592769	166	603	3.3908944	14	THBS1
**PREX2.2**	1.9176	-0.470573641745406	73	435	3.8352377	14	PREX2
**HOPX.1**	2.8805	1.20438144634735	0.89	321	5.7611247	14	HOPX
**IGF2.4**	1.4044	-1.09126833971983	194	559	2.8088986	14	IGF2
**FERMT1**	2.7671	0.840736257035182	789	0.17	5.5342084	14	FERMT1
**PHGDH**	4.8444	0.768107168438639	829	195	9.6889281	14	PHGDH
**SDC1.4**	6.7176	0.828665493821595	0.91	454	1.3435205	14	SDC1
**CYP24A1**	1.2287	0.293215644154593	739	117	2.4574672	14	CYP24A1
**SMAD1.2**	2.3554	0.323650309960367	958	422	4.7109642	14	SMAD1
**HN1.1**	3.7365	0.976168657933137	983	509	7.4730735	14	HN1
**KRT19.2**	6.0791	1.52959505387709	843	249	1.2158246	14	KRT19
**CNFN**	1.8506	-0.42631233126999	817	0.18	3.7012628	14	CNFN
**MFAP4.3**	2.5532	-0.634090421416848	121	438	5.1064156	14	MFAP4
**S100A14.2**	1.2013	1.85827120178126	851	239	2.4026970	14	S100A14

**PRRG4.1**	1.4345	1.02918179497271	806	202	2.8691499	14	PRRG4
**SERPINE2.3**	1.3884	0.300751630434465	907	437	2.7768677	14	SERPINE2
**IFNG.2**	1.9412	-0.412468229768163	65	415	3.8825188	14	IFNG
**PAPPA.2**	2.7581	0.362041208972026	935	563	5.5162210	14	PAPPA
**SCARA5.4**	4.9665	-0.460784128306649	216	534	9.9330608	14	SCARA5
**MT1X.1**	7.7481	2.33930654481609	896	515	1.5496235	14	MT1X
**EHF**	2.3706	1.12346498624524	801	136	4.7413951	14	EHF
**NID1.1**	6.4121	-0.728185990920743	236	653	1.2824213	14	NID1
**NQO1**	7.3630	0.79701051239428	865	377	1.4726124	14	NQO1
**KIAA1217.5**	5.4637	0.398628668623704	941	444	1.0927425	14	KIAA1217
**CXCL13.1**	9.8595	-2.40434397869009	0.23	651	1.9719014	14	CXCL13
**C1QA.2**	3.7780	-0.557352756568328	132	0.53	7.5560211	14	C1QA
**SERPINB1.1**	2.3585	0.91731667476041	0.98	509	4.7171476	14	SERPINB1
**CD34.4**	2.9114	-0.586264576694036	0.14	519	5.8228008	14	CD34
**SPI1.2**	6.7655	-0.300458066421386	67	333	1.3531064	14	SPI1
**ENC1.1**	1.1098	-0.26373938017658	171	519	2.2197915	14	ENC1
**AQP3.1**	1.4816	2.00217858831006	857	402	2.9632995	14	AQP3
**TUSC3**	2.2777	0.387236403412108	851	267	4.5554039	14	TUSC3
**CYP26B1.4**	4.2200	-0.254035434397559	924	309	8.4400676	14	CYP26B1
**PKP3**	1.9315	1.19294970782693	792	121	3.8630289	14	PKP3
**NDRG2**	1.4925	0.257536261595125	893	318	2.9850496	14	NDRG2
**RORB.5**	1.9565	-0.557158849510735	194	454	3.9131063	14	RORB
**PPL.1**	3.4030	0.394104780400838	851	279	6.8061667	14	PPL
**DSG3**	5.1432	1.32919415952959	798	235	1.0286505	14	DSG3
**IL1R2**	1.4347	0.424156742887292	809	348	2.8694681	14	IL1R2
**RHOD**	1.6139	0.618703664278074	829	322	3.2279520	14	RHOD
**HSPB1.5**	1.8096	1.22116185342083	975	611	3.6192838	14	HSPB1
**TYMS**	1.5219	0.359971403237881	871	313	3.0439769	14	TYMS
**MEOX1.3**	1.7932	-0.32280088940134	129	381	3.5864767	14	MEOX1
**PRELP.5**	1.2261	-0.255239594617546	924	334	2.4523483	14	PRELP
**VASN.4**	4.7391	-0.30986044928246	0.18	484	9.4783512	14	VASN
**IGKV3-15.1**	1.0211	-0.313275023774811	767	258	2.0422592	14	IGKV3-15

**HMGA1.1**	1.5387	0.350772758964665	0.93	463	3.0774224	14	HMGA1
**PROM2**	3.0023	0.397796768912946	767	203	6.0046815	14	PROM2
**PCNA**	3.4406	0.853050927356531	0.89	396	6.8812637	14	PCNA
**CALCRL.4**	1.2869	-0.64624464477157	194	471	2.5739469	14	CALCRL
**FILIP1L.6**	1.3339	-1.38427607233825	96	602	2.6678696	14	FILIP1L
**SELP.4**	3.3756	-0.45278866976804	958	539	6.7512172	14	SELP
**PCOLCE.2**	5.8163	-1.49851651137617	138	519	1.1632733	14	PCOLCE
**CRISPLD2.5**	4.1966	-0.810573587434744	258	577	8.3933515	14	CRISPLD2
**MASP1.6**	8.7266	-0.355108293930311	935	427	1.7453369	14	MASP1
**CPE.4**	9.8140	-0.813940716012008	188	529	1.9628180	14	CPE
**NFKBID.2**	1.3293	-0.693715755483235	219	607	2.6586648	14	NFKBID
**C5orf66-AS1**	6.7324	0.260733437196491	722	128	1.3464978	14	C5orf66-AS1
**TUBA4A**	1.3745	0.378922621581206	972	531	2.7491923	14	TUBA4A
**XCL1.3**	1.1432	-0.501686652342243	952	0.54	2.2864534	14	XCL1
**TP63**	1.5151	1.0769143342065	781	221	3.0302662	14	TP63
**IL33.7**	1.2423	-0.2751498750581	0.93	407	2.4846864	14	IL33
**TNS4.1**	1.2745	0.617432699488257	772	289	2.5491654	14	TNS4
**S100A16.6**	5.4188	1.14180319004291	933	505	1.0837709	14	S100A16
**CD3D.2**	6.8954	-1.33632413668703	211	591	1.3790988	14	CD3D
**JDP2.3**	9.1618	0.435230085769066	966	548	1.8323716	14	JDP2
**BCL11A**	1.4531	0.795704793913321	784	226	2.9063330	14	BCL11A
**ALDH2.1**	3.2585	1.01796613190783	851	319	6.5171126	14	ALDH2
**TM4SF18.4**	1.2487	-0.297840021311887	949	474	2.4974869	14	TM4SF18
**TNF.2**	1.8939	-0.25748368938218	952	532	3.7878681	14	TNF
**IL20RB**	4.9540	0.613229854435703	0.77	0.21	9.9080640	14	IL20RB
**COL6A1.3**	2.1936	-2.66073370786531	281	667	4.3872255	14	COL6A1
**MYCT1.4**	5.7906	-0.259366176536477	947	471	1.1581277	14	MYCT1
**PIK3R3.3**	8.4490	-0.339123381908601	185	505	1.6898017	14	PIK3R3
**DUSP14**	2.9720	0.402420229971867	896	507	5.9441294	14	DUSP14
**CCL20**	4.9700	0.894391189469943	781	313	9.9401237	14	CCL20
**VSNL1.1**	8.0155	1.10841863250186	758	145	1.6031195	14	VSNL1
**FCRL5.1**	1.0641	-0.303761111207753	952	576	2.1282396	14	FCRL5

**FGFR1.3**	1.4322	-0.973927126205997	287	598	2.8644586	14	FGFR1
**VCAN.2**	2.2689	-2.24004117848698	388	664	4.5378777	14	VCAN
**JAG1.4**	5.3578	0.482936698546225	958	549	1.0715731	14	JAG1
**CXCL12.2**	5.7473	-2.16435579222042	0.41	716	1.1494745	14	CXCL12
**CXorf36.5**	1.0901	-0.286902709318662	944	0.45	2.1802841	14	CXorf36
**SDPR.2**	2.6296	-0.495772162046669	177	0.45	5.2593661	14	SDPR
**SLC7A1**	7.5779	0.947634977348065	817	0.45	1.5155950	14	SLC7A1
**PTPRB.6**	9.7972	-0.413740132147829	947	534	1.9594499	14	PTPRB
**MMRN2.6**	1.7894	-0.493794467954457	115	451	3.5789502	14	MMRN2
**KDR.4**	4.4759	-0.385193586556543	947	448	8.9519151	14	KDR
**ROBO4.6**	5.7513	-0.340885985330553	961	433	1.1502651	14	ROBO4
**LAMA3.1**	1.6144	0.43686646313864	789	311	3.2288320	14	LAMA3
**LAMB3**	1.8727	1.1274694782557	778	281	3.7454715	14	LAMB3
**MPZL2.8**	2.1670	0.547505836663299	868	428	4.3340320	14	MPZL2
**DHCR24**	2.2726	0.661135026189951	798	393	4.5452453	14	DHCR24
**PDGFRB.7**	8.6056	-0.999143633293526	278	566	1.7211203	14	PDGFRB
**PDZK1IP1**	1.8003	0.729636341942791	0.75	187	3.6006132	14	PDZK1IP1
**CHI3L2.3**	2.2619	-0.497554287473622	76	332	4.5238366	14	CHI3L2
**LRP1.3**	2.3070	-0.740936078479047	0.25	556	4.6141253	14	LRP1
**LTBP2.4**	2.6226	-0.367827146654072	966	569	5.2452637	14	LTBP2
**FLT1.5**	3.3331	-0.507439429350701	955	489	6.6662110	14	FLT1
**FAM25A.1**	5.0633	-0.307696506244235	716	74	1.0126657	14	FAM25A
**VAMP8.2**	1.6331	0.639815524799677	885	398	3.2663780	14	VAMP8
**EFEMP2.2**	2.3087	-0.274103619137633	202	0.49	4.6175709	14	EFEMP2
**APOC1.1**	3.1378	-0.348708690733666	138	427	6.2756789	14	APOC1
**PTGR1.2**	4.0444	0.771690167027517	831	379	8.0889752	14	PTGR1
**TFPI.1**	9.0462	-0.596917661592752	236	617	1.8092461	14	TFPI
**DAPL1**	1.6397	1.05270502926941	772	292	3.2795458	14	DAPL1
**SLIT3.8**	2.3616	-0.475173310534052	952	421	4.7233043	14	SLIT3
**CD3E.2**	3.4616	-1.03677320661012	166	536	6.9232116	14	CD3E
**NEXN.2**	1.0318	-0.258135521059856	79	413	2.0636066	14	NEXN
**TRIM29**	1.9871	1.17509934646099	767	214	3.9743344	14	TRIM29

**DDR2.1**	3.6216	-0.662384449080448	281	554	7.24320581	14	DDR2
**GBP6.1**	6.2891	1.1233163927944	753	218	1.25782543	14	GBP 6
**PCDH17.7**	1.2579	-0.545114042846241	961	545	2.51592398	14	PCDH17
**ENPEP.5**	7.5304	-0.314273220571692	51	437	1.50609527	14	ENPEP
**ITGB2.2**	5.4032	-0.736749199640504	171	498	1.08064357	14	ITGB2
**JUP.3**	2.5417	1.05420498229952	789	0.37	5.08345573	14	JUP
**STC1.2**	4.5639	-0.567031423234116	958	608	9.12798394	14	STC1
**ATP1B1.1**	2.6609	0.312208442771968	0.82	481	5.32195428	14	ATP1B1
**TACSTD2.1**	3.0972	1.53083397962842	0.77	295	6.19452661	14	TACSTD2
**FBLN2.5**	3.5587	-1.31096927260583	0.25	535	7.11745040	14	FBLN2
**ZNF185**	5.1649	0.546402587073016	742	266	1.03299704	14	ZNF185
**MYC.2**	7.0040	0.976247468711689	848	475	1.40080932	14	MYC
**CD84.2**	1.2196	-0.452585970143906	0.18	508	2.43923773	14	CD84
**CD79A.4**	1.2236	-0.633099218873664	913	482	2.44728480	14	CD79A
**AREG.1**	2.5680	-0.436213125449129	969	638	5.13617027	14	AREG
**CDH5.3**	7.4250	-0.559106933514131	958	604	1.48500971	14	CDH5
**FAM20C.2**	4.9025	-0.626354909124289	278	0.54	9.80506239	14	FAM20C
**LBP.3**	7.6125	-0.368038666862297	93	496	1.52251643	14	LBP
**PLAUR.2**	6.9532	-0.261901752516828	933	598	1.39064349	14	PLAUR
**TNXB.4**	1.1531	-0.350961692118963	955	525	2.30621584	14	TNXB
**KIAA0101.1**	1.1679	0.654201582669617	739	308	2.33586395	14	KIAA0101
**LINC01133**	9.8985	0.479689281060536	694	0.07	1.97971529	14	LINC01133
**PKP1**	1.0624	1.48785623511284	0.73	216	2.12487091	14	PKP1
**PDLIM1.5**	1.7407	0.546634204818151	986	0.63	3.48155560	14	PDLIM1
**EMCN.7**	1.9418	-0.692058021547638	961	518	3.88366738	14	EMCN
**INSR.3**	3.1543	-0.499086883896556	958	563	6.30868901	14	INSR
**TMEM176A.5**	5.3371	-0.642274757073872	261	514	1.06742644	14	TMEM176A
**SELL.1**	1.1611	-0.36162509100182	952	541	2.32221699	14	SELL
**TNFSF10.3**	1.5237	0.846015037293808	907	549	3.04757289	14	TNFSF10
**EML1.2**	1.8438	-0.274265001710328	67	323	3.68779650	14	EML1
**ROBO1.4**	3.0010	-0.288862168691362	0.27	532	6.00209438	14	ROBO1
**ALPL.4**	3.8427	-0.456136847606747	213	512	7.68548032	14	ALPL

**SELE.3**	4.3160	-1.65536234653597	961	637	8.6321980	14	SELE
**COL7A1.1**	5.2324	0.528550655009535	761	356	1.0464917	14	COL7A1
**POSTN.1**	1.2764	-0.958609097045263	958	547	2.5529621	14	POSTN
**WNT10A**	3.8961	0.484251268164023	711	0.24	7.7923758	14	WNT10A
**GZMK**	1.3897	-1.30278744981693	941	583	2.7795531	14	GZMK
**JAML.4**	1.4494	-0.396223930000984	879	435	2.8989185	14	JAML
**CD83.2**	1.8264	-0.742023506122188	963	599	3.6529258	14	CD83
**CCL4L2.3**	3.5161	-1.33019843074383	281	537	7.0323364	14	CCL4L2
**GGT5.3**	5.1289	-0.812939629941297	267	594	1.0257939	14	GGT5
**IGFBP3.2**	5.4737	0.339068467343835	787	474	1.0947515	14	IGFBP3
**GNLY.1**	1.3771	-1.49447866027094	174	438	2.7543864	14	GNLY
**CRABP1.7**	1.7341	-0.466167753837707	28	0.32	3.4682016	14	CRABP1
**SPRY1.4**	3.4375	-1.057719351414	312	0.6	6.8750417	14	SPRY1
**MS4A1.4**	9.7361	-0.410628114748406	817	364	1.9472340	14	MS4A1
**MT1E.2**	2.2043	0.98490971792749	823	434	4.4087083	14	MT1E
**GNA15.1**	6.4319	0.277104042566819	0.75	341	1.2863811	14	GNA15
**RCAN1.3**	3.6575	-0.543685726573674	0.27	534	7.3151137	14	RCAN1
**TFAP2A**	7.7974	0.95689034087795	697	174	1.5594947	14	TFAP2A
**SPINT1**	2.9989	0.513544099436529	713	229	5.9978796	14	SPINT1
**IGFBP2.3**	3.7973	0.327912539136139	879	587	7.5947037	14	IGFBP2
**ADGRL4.6**	4.0335	-0.831729284753433	958	586	8.0671934	14	ADGRL4
**ERG.4**	4.6171	-0.371744959260005	899	434	9.2343978	14	ERG
**AKR1C2**	5.8154	0.458320221378973	705	181	1.1630921	14	AKR1C2
**CPXM1.2**	9.0188	-0.253967635705767	0.93	505	1.8037631	14	CPXM1
**CLEC14A.6**	6.7895	-0.761238468605331	933	524	1.3579057	14	CLEC14A
**GZMA.2**	1.6724	-1.17459918119213	281	539	3.3448267	14	GZMA
**THSD7A.8**	2.2381	-0.56970015416611	62	423	4.4762176	14	THSD7A
**MSX1.1**	1.5524	-0.620978951093508	0.27	563	3.1049994	14	MSX1
**PLVAP.5**	2.4241	-0.932123892276038	938	574	4.8483751	14	PLVAP
**ITGA6.7**	2.4995	0.274685344334022	952	616	4.9990456	14	ITGA6
**PDGFRA.5**	3.5757	-0.812441407196361	351	603	7.1514089	14	PDGFRA
**FAT2**	4.1181	0.603233956702127	0.68	111	8.2362353	14	FAT2

**LGALS3BP.3**	1.1639	0.377483874851889	846	533	2.3278958	14	LGALS3BP
**S100A13.4**	4.3019	0.312524689343074	972	536	8.6038413	14	S100A13
**SOX17.2**	4.4465	-0.268041181001934	67	0.33	8.8931964	14	SOX17
**ESAM.4**	4.8853	-0.53771730074934	933	523	9.7706847	14	ESAM
**IL18.1**	1.3120	0.77491027145639	711	232	2.6241658	14	IL18
**CTLA4.3**	1.7219	-0.3761912327374	0.91	457	3.4439503	14	CTLA4
**RNASE1.5**	2.1892	-0.708531604384233	949	571	4.3784159	14	RNASE1
**COL12A1.1**	2.8293	-1.05878796075088	958	626	5.6586830	14	COL12A1
**SAA1.1**	3.1370	0.824254443916127	719	0.26	6.2740940	14	SAA1
**CST7**	7.2186	-1.03384474143807	938	0.61	1.4437240	14	CST7
**ECSCR.1.5**	1.2011	-0.805690211510262	935	569	2.4022449	14	ECSCR.1
**DUSP2.1**	1.5119	-1.4911084611028	357	647	3.0238241	14	DUSP2
**CCR7**	1.5528	-0.550469299855251	952	628	3.1056140	14	CCR7
**RAB27B**	3.0201	0.507977235869482	691	253	6.0403979	14	RAB27B
**NKG7.2**	4.0389	-1.20837656831016	211	499	8.0778177	14	NKG7
**LYPD3.1**	8.2171	0.855088007697365	733	303	1.6434364	14	LYPD3
**PLEK2**	8.3335	0.432903118839063	677	144	1.6667012	14	PLEK2
**RAB11FIP1.3**	1.1470	-0.401361765993674	961	576	2.2940576	14	RAB11FIP1
**FAM110C**	3.7712	0.375070940671444	671	183	7.5424424	14	FAM110C
**TMPRSS11D**	1.0697	0.453682416669126	694	215	2.1395218	14	TMPRSS11D
**ARHGAP29.4**	1.0817	-0.603270069979565	949	524	2.1634194	14	ARHGAP29
**EDNRB.4**	1.1494	-0.317399054263301	67	433	2.2988209	14	EDNRB
**PIM2.3**	1.3640	-0.386601354464171	947	487	2.7281860	14	PIM2
**TNFRSF21**	3.2813	0.435397707392102	0.77	461	6.5627049	14	TNFRSF21
**CALML3.1**	4.0981	1.46247844950029	691	218	8.1963022	14	CALML3
**SLCO2A1.3**	9.4129	-0.540348696537457	913	623	1.8825839	14	SLCO2A1
**VWF.6**	1.1161	-1.10234875524768	947	574	2.2323782	14	VWF
**ADAMTS4.4**	2.0661	-0.353608234026169	949	545	4.1322152	14	ADAMTS4
**TPSAB1.1**	2.2438	-0.633426346638588	542	265	4.4876057	14	TPSAB1
**DUSP6.2**	2.9202	-0.283835051913627	972	634	5.8404162	14	DUSP6
**PTPRF**	3.8726	0.841113999445837	705	407	7.7452876	14	PTPRF
**LY6D.2**	1.0379	2.22145108618974	694	266	2.0758547	14	LY6D

**ITGB4.8**	1.4851	0.38270059926502	792	427	2.97035644	14	ITGB4
**FAP.2**	3.1683	-0.29408205674354	34	383	6.33661979	14	FAP
**MT2A.6**	3.2785	0.286065697158515	0.93	652	6.55714493	14	MT2A
**CLDN5.7**	8.2128	-1.00322552321825	888	437	1.64256720	14	CLDN5
**MAFB.1**	9.9732	0.529607716287682	775	452	1.99464760	14	MAFB
**IGLV1-51.2**	1.4481	-0.443702481227732	725	268	2.89625370	14	IGLV1-51
**CPVL.3**	1.8468	-0.435928180590431	848	404	3.69366673	14	CPVL
**RAMP3.5**	1.9473	-0.625231793136818	935	484	3.89467255	14	RAMP3
**IGFBP5.3**	2.0899	-0.716426974128237	0.89	626	4.17997941	14	IGFBP5
**TXN.4**	4.8736	1.0704442160072	947	679	9.74733780	14	TXN
**LMO4.3**	5.2093	0.48256486376731	862	488	1.04186813	14	LMO4
**MZB1.3**	1.2108	-1.47134782058737	955	617	2.42176639	14	MZB1
**SPNS2.3**	6.7024	-0.340675222524016	0.91	0.48	1.34048655	14	SPNS2
**CTSK.3**	9.9924	-1.25831830088579	947	577	1.99849600	14	CTSK
**STMN1.1**	1.5847	0.918490301250805	747	0.4	3.16953962	14	STMN1
**EMP2.4**	1.9485	0.626127427913919	829	522	3.89719609	14	EMP2
**PRF1.1**	2.3416	-0.261713820645521	781	376	4.68330511	14	PRF1
**GREM1.4**	3.5491	-0.285861684676987	48	458	7.09836740	14	GREM1
**PCDH7**	9.5639	0.774461480993694	654	172	1.91279784	14	PCDH7
**EGFL7.8**	1.1940	-0.476393122424561	893	423	2.38806718	14	EGFL7
**CLTB.2**	2.4797	0.554354466039344	879	555	4.95945311	14	CLTB
**WNT5A.3**	2.8357	-0.436369636576552	823	0.57	5.67148197	14	WNT5A
**MMRN1.4**	5.5625	-0.283507363887499	81	412	1.11250415	14	MMRN1
**KRT16.1**	8.9651	3.15622678549955	699	335	1.79302888	14	KRT16
**CAPG.1**	4.6222	0.383644167103682	764	377	9.24444560	14	CAPG
**POU2F2.6**	8.5070	-0.505659076765518	888	468	1.70140632	14	POU2F2
**GCLC**	1.5950	0.863604185174287	705	419	3.19007512	14	GCLC
**KRT6B**	2.2703	0.459415377040147	669	247	4.54076802	14	KRT6B
**FHL2.1**	2.3404	0.377733202671788	736	441	4.68093404	14	FHL2
**SCPEP1.3**	8.2692	0.405694354148595	809	424	1.65384220	14	SCPEP1
**LMCD1.1**	9.3019	-0.506582837709703	938	595	1.86039241	14	LMCD1
**ACKR1.4**	1.4533	-1.41399861108224	935	549	2.90669562	14	ACKR1

**ITGB8**	1.9909	0.373298282726004	666	254	3.98184714	14	ITGB8
**ADGRF5.3**	2.1049	-0.620777960837619	938	574	4.20980914	14	ADGRF5
**TMPRSS11A**	3.1042	0.414095458056815	0.66	159	6.20855442	14	TMPRSS11A
**TGM2.1**	6.4861	-0.575355798004919	955	607	1.29723141	14	TGM2
**JSRP1.2**	7.5537	-0.258612222453091	865	0.48	1.51075850	14	JSRP1
**C2.2**	1.7946	-0.300152467296177	947	494	3.58933504	14	C2
**TNFSF13B.3**	2.3838	-0.27315164783453	919	442	4.76778438	14	TNFSF13B
**GLTP**	2.7596	0.309416514144792	775	436	5.51937561	14	GLTP
**TPM2.5**	4.0550	-0.803980227115948	815	412	8.11015744	14	TPM2
**WWTR1.4**	4.8352	-0.481084508267238	958	583	9.67059102	14	WWTR1
**RAI14.4**	5.8991	-0.383163333669625	963	616	1.17982042	14	RAI14
**GPR87**	6.4944	0.253212615663705	326	28	1.29889882	14	GPR87
**LTB.2**	9.2840	-0.82107912958765	871	476	1.85681193	14	LTB
**COL15A1.3**	4.3203	-1.32494530261811	935	662	8.64072568	14	COL15A1
**HSPA2.3**	6.8703	0.732061787424511	694	406	1.37407044	14	HSPA2
**S1PR1.6**	1.0135	-0.327083416909235	949	523	2.02712841	14	S1PR1
**DPT.4**	1.2879	-0.294414967746631	124	376	2.57590208	14	DPT
**LTBP1.2**	1.9244	-0.401294801705655	837	0.57	3.84885228	14	LTBP1
**TPM1.7**	2.0717	-1.09783686479131	952	499	4.14357950	14	TPM1
**C10orf10.5**	9.2433	-0.428828799953029	154	418	1.84866364	14	C10orf10
**FZD4.2**	9.3641	-0.298934761440037	132	393	1.87283830	14	FZD4
**TAGLN.3**	9.7250	-2.16869467485739	865	447	1.94500900	14	TAGLN
**CCL21.1**	2.9553	-0.769975799491503	163	461	5.91061505	14	CCL21
**CKB**	3.6643	0.497174449550768	674	298	7.32879722	14	CKB
**KCNJ2.1**	4.8502	0.258795691418627	649	215	9.70048752	14	KCNJ2
**RASSF4.2**	5.7736	-0.287679318202602	919	419	1.15473648	14	RASSF4
**DERL3.3**	5.8268	-0.611515494460263	882	0.56	1.16536152	14	DERL3
**KRT6C.1**	7.5806	1.62879710786312	618	234	1.51613070	14	KRT6C
**SPINK5**	1.4548	1.35873344553431	654	225	2.90965938	14	SPINK5
**RGS3.2**	1.4323	-0.517811569267576	958	581	2.86466540	14	RGS3
**SOX18.4**	1.8952	-0.712146144616702	885	0.45	3.79051860	14	SOX18
**CRYBG3.5**	3.0111	-0.270691965518524	907	547	6.02235542	14	CRYBG3

**TSPAN11.1**	4.0826	-0.26865104775866	121	461	8.1652142	14	TSPAN11
**FMO2**	4.8769	0.266669549383309	663	0.28	9.7538663	14	FMO2
**RHOJ.4**	5.9193	-0.356704734354937	944	549	1.1838639	14	RHOJ
**HMGB2.2**	7.7672	0.588369042994254	815	481	1.5534572	14	HMGB2
**BCL2A1.4**	8.0485	-0.428020216358386	815	423	1.6097011	14	BCL2A1
**MCAM.5**	2.9582	-0.905762319194964	921	594	5.9164288	14	MCAM
**ADRB2**	4.2222	0.526610783779099	671	0.34	8.4444688	14	ADRB2
**FGFR3**	7.4921	0.441474706068663	652	297	1.4984209	14	FGFR3
**UBE2T**	1.0517	0.305910505482714	0.66	326	2.1035757	14	UBE2T
**SERPINH1.2**	1.5889	-0.331250117674101	969	707	3.1779513	14	SERPINH1
**CTNNBIP1**	1.2841	0.642103797958604	677	331	2.5682281	14	CTNNBIP1
**IMPA2**	3.4266	0.582305356379882	643	243	6.8533627	14	IMPA2
**PLAC8.2**	8.0372	-0.282149117354196	705	318	1.6074586	14	PLAC8
**CKS2.1**	8.8025	0.315078351257503	775	469	1.7605068	14	CKS2
**PODXL.2**	9.1012	-0.730311231675472	919	619	1.8202453	14	PODXL
**TM4SF1.5**	3.0845	-0.526827531776438	913	614	6.1691145	14	TM4SF1
**HIST1H4C**	5.9825	0.541257172238437	983	721	1.1965138	14	HIST1H4C
**FDCSP.1**	7.9549	0.658252033878269	697	296	1.5909888	14	FDCSP
**CD93.3**	9.4942	-1.16154361399322	941	665	1.8988591	14	CD93
**NUAK1.5**	1.4603	-0.33678558962986	815	459	2.9206416	14	NUAK1
**SOX2**	2.7375	0.486042865842259	587	56	5.4751770	14	SOX2
**NUPR1.6**	6.6142	0.353330362920655	722	0.4	1.3228544	14	NUPR1
**CENPW**	6.7024	0.638042741139644	0.64	346	1.3404983	14	CENPW
**NES.6**	6.7791	-0.364470046291166	817	0.36	1.3558375	14	NES
**CD200.6**	7.4233	-0.419717778921437	879	0.52	1.4846715	14	CD200
**CRABP2.6**	7.7547	0.970516054838213	0.68	385	1.5509526	14	CRABP2
**LYZ.2**	1.4961	-2.24366997906286	938	595	2.9922140	14	LYZ
**RAB31.4**	1.5517	-0.407765492011411	935	622	3.1035741	14	RAB31
**CRCT1.2**	3.1519	-2.23991734276349	0.57	176	6.3038004	14	CRCT1
**VCAM1.3**	3.9490	-0.774707113260205	961	0.65	7.8980490	14	VCAM1
**DDR1**	6.0995	0.700223368594116	0.61	317	1.2199096	14	DDR1
**SFRP4.2**	2.5578	-0.41288246572877	93	411	5.1157190	14	SFRP4

**PRCP.2**	9.0469	-0.469390088181901	0.93	619	1.80939998	14	PRCP
**FKBP11.5**	1.6050	-0.36274623708405	902	509	3.21015551	14	FKBP11
**PTTG1**	9.9764	0.780819938313165	638	364	1.99528855	14	PTTG1
**CYR61.1**	3.2824	-1.12539603651743	969	713	6.56488009	14	CYR61
**RAMP2.6**	2.2571	-1.02173919276817	0.84	479	4.51438732	14	RAMP2
**IL7R**	4.2164	-1.62497229250746	947	604	8.43295940	14	IL7R
**RCAN2.2**	4.7261	-0.327794558905382	28	358	9.45239555	14	RCAN2
**ADAMTS2.6**	4.8789	-0.418188670610575	222	484	9.75790508	14	ADAMTS2
**IL3RA.5**	5.2205	-0.555463905236875	904	575	1.04410179	14	IL3RA
**LINC00152.2**	5.8947	-0.603807518320532	952	597	1.17895795	14	LINC00152
**RP11-1143G9**	6.2630	-0.257735001072974	775	444	1.25261545	14	RP11-1143G9
**PTHLH**	8.2194	0.3506462036096	624	351	1.64389770	14	PTHLH
**CDH3**	1.5525	0.320934148714678	579	112	3.10502687	14	CDH3
**HBA2.1**	1.6578	-1.07530347348124	0.86	464	3.31569783	14	HBA2
**FILIP1.4**	2.2631	-0.390500950840381	685	338	4.52628001	14	FILIP1
**SLC6A6.2**	4.4523	-0.368910710145298	904	576	8.90465184	14	SLC6A6
**IGLC3**	8.4641	-3.66303448468984	961	0.67	1.69282661	14	IGLC3
**PXDN.3**	1.0490	-0.693012369511498	961	687	2.09802903	14	PXDN
**KRT8**	1.3238	0.847929998060891	584	249	2.64775568	14	KRT8
**RAB25**	1.5260	0.483533396040814	0.59	162	3.05213828	14	RAB25
**PTP4A3.2**	1.6054	-0.428646248273751	882	474	3.21098070	14	PTP4A3
**IGLV3-1.5**	1.7490	-0.754779688630416	733	368	3.49803225	14	IGLV3-1
**FAM46C.2**	2.5218	-0.659594918178085	938	0.55	5.04364075	14	FAM46C
**ICAM2.3**	3.0122	-0.553021423307969	961	529	6.02446887	14	ICAM2
**HTRA1.4**	5.1867	-0.44056982067727	862	601	1.03735703	14	HTRA1
**GJB5**	5.5640	0.267486985770293	559	123	1.11281578	14	GJB5
**HYAL2.4**	6.8544	-0.493412191726576	784	497	1.37089519	14	HYAL2
**CSRP2.3**	1.3956	0.402316908650878	683	0.41	2.79133867	14	CSRP2
**ANPEP.6**	1.4605	-0.339361610150005	51	385	2.92117833	14	ANPEP
**ACKR3.2**	2.0353	-0.403967445244173	899	607	4.07073027	14	ACKR3
**CCDC71L.2**	2.6277	-0.305924882498475	823	461	5.25541474	14	CCDC71L
**HTRA3.3**	3.6823	-0.259734337994773	96	389	7.36479442	14	HTRA3

**PRLR**	4.4256	0.278633247814771	542	84	8.8512151	14	PRLR
**A2M.7**	5.1863	-1.60068753149377	935	653	1.0372670	14	A2M
**RGS1.1**	5.4394	-1.30994736899023	0.86	564	1.0878925	14	RGS1
**LST1.2**	5.5588	-0.367262059343477	601	285	1.1117793	14	LST1
**SPOCK2.1**	9.3993	-0.722997705982142	927	0.53	1.8798676	14	SPOCK2
**CYBB.4**	1.2425	-0.378485350147543	0.73	0.38	2.4850157	14	CYBB
**CCL4.1**	1.5080	-2.74606175678368	916	592	3.0160914	14	CCL4
**AOAH.4**	1.9941	-0.263812162624088	767	418	3.9883841	14	AOAH
**DSC2.1**	2.5794	0.919556180856881	596	265	5.1588128	14	DSC2
**TIGIT.1**	2.8830	-0.608450128376192	888	573	5.7661984	14	TIGIT
**CTSC.2**	1.9972	-0.425062902737381	921	544	3.9945816	14	CTSC
**SEC11C.3**	2.4386	-0.536008679883084	857	538	4.8773617	14	SEC11C
**CPA3.4**	2.5979	-0.622156765370555	677	368	5.1959771	14	CPA3
**AQP1.6**	3.2440	-1.54012739896559	938	681	6.4881660	14	AQP1
**TP53AIP1**	3.2929	0.628967834046609	553	75	6.5858584	14	TP53AIP1
**ADH7**	4.8168	0.337466285965887	388	85	9.6337691	14	ADH7
**PECAM1.5**	6.5688	-1.3718957395551	961	669	1.3137638	14	PECAM1
**GRASP.6**	1.0701	-0.708461799086853	919	569	2.1402591	14	GRASP
**RGS2.2**	1.2455	-0.95099310091702	941	613	2.4911410	14	RGS2
**LAMA4.3**	1.7415	-0.822561728904821	949	565	3.4831220	14	LAMA4
**MAFF.1**	2.0450	-0.377462673782242	947	0.68	4.0901072	14	MAFF
**ADIRF.7**	2.3636	-0.909323584588092	969	659	4.7272313	14	ADIRF
**SLC47A2**	4.7193	0.263420105290076	525	61	9.4386871	14	SLC47A2
**C8orf4.1**	6.2848	-1.09751395278544	913	577	0.0001256	14	C8orf4
**CITED2**	1.0189	-0.357034184887923	812	517	0.0002037	14	CITED2
**AIF1.2**	1.1103	-0.687899174806553	646	387	0.0002220	14	AIF1
**CPXM2.3**	1.8614	-0.646225506806156	949	636	0.0003722	14	CPXM2
**EDIL3.4**	1.9444	-0.732265049026377	0.86	559	0.0003888	14	EDIL3
**LCP1.4**	2.6945	-1.03521254354229	865	572	0.0005389	14	LCP1
**LGALS2.3**	2.8309	-0.28979393469439	581	292	0.0005661	14	LGALS2
**APOLD1.3**	2.9124	-0.710526977303266	958	637	0.0005824	14	APOLD1
**C2CD4B.5**	3.2960	-0.582129893177047	787	535	0.0006592	14	C2CD4B

**KLK11.1**	3.6053	0.506548579869458	551	121	0.0007210	14	KLK11
**XBP1.4**	4.5911	-0.866609499764598	963	654	0.0009182	14	XBP1
**COL4A2.3**	4.7471	-1.23212834580128	963	705	0.0009494	14	COL4A2
**CD14.2**	4.9250	-0.46094007790484	865	573	0.0009850	14	CD14
**SPRR2E.1**	1.4359	-0.48096768959434	531	0.24	0.0028718	14	SPRR2E
**LEPR.2**	1.4850	-0.623625167234171	843	563	0.0029700	14	LEPR
**DLC1.4**	1.5949	-0.777087347495007	955	625	0.0031898	14	DLC1
**KCTD12.3**	1.7713	-0.743250707475562	0.89	0.59	0.0035426	14	KCTD12
**SFRP2.2**	2.6403	-0.833540619645383	865	518	0.0052806	14	SFRP2
**NMU**	3.7574	0.330953826060023	416	0.08	0.0075149	14	NMU
**RARRES2.2**	8.0229	-1.64791656282971	924	643	0.0160458	14	RARRES2
**ENG.6**	1.2114	-1.0616235642952	921	597	0.0242280	14	ENG
**DUSP5**	2.2491	-0.478970546806482	924	603	0.0449830	14	DUSP5
**C1QC.4**	9.2687	-0.288797298169806	632	366	0.1853746	14	C1QC
**BANK1.1**	0.0001	-0.290566078201886	584	0.3	0.2717440	14	BANK1
**FOXE1**	0.0001	0.328564106667539	528	208	0.2763718	14	FOXE1
**FAM13C.4**	0.0001	-0.346075090363211	663	365	0.2892989	14	FAM13C
**FGL2.3**	0.0001	-0.497192266915786	798	466	0.3027408	14	FGL2
**TMEM54**	0.0001	0.370365003315259	565	0.27	0.3883845	14	TMEM54
**CD8A.3**	0.0005	-0.810952106170283	876	572	1	14	CD8A
**INSIG1.1**	0.0006	-0.572722093695434	862	611	1	14	INSIG1
**IRX4.1**	0.0010	0.319183127584377	424	66	1	14	IRX4
**ZNF750**	0.0011	0.520993394310633	433	157	1	14	ZNF750
**CAPNS2**	0.0012	0.507174659439742	528	185	1	14	CAPNS2
**HERPUD1.2**	0.0021	-1.0087960475632	935	0.68	1	14	HERPUD1
**PITX2**	0.0043	0.470337539603483	0.52	136	1	14	PITX2
**BATF.2**	0.0046	-0.595338478765649	806	509	1	14	BATF
**CA12.1**	0.0047	0.318621610781544	449	193	1	14	CA12
**CD52.4**	0.0066	-1.03613370557861	829	578	1	14	CD52
**HLA-DRA.3**	2.4222	3.19845126343798	988	691	4.8444960	15	HLA-DRA
**XCR1**	1.3729	0.659257408631807	693	155	2.7458317	15	XCR1
**HLA-DPB1.4**	6.8060	3.45715316368072	966	599	1.3612019	15	HLA-DPB1

**HLA-DPA1.5**	1.4717	3.33345879066778	963	642	2.9434565	15	HLA-DPA1
**PADI2**	7.6147	0.452012233102834	675	164	1.5229420	15	PADI2
**HLA-DQB1.4**	1.3505	2.86496697507682	954	565	2.7010549	15	HLA-DQB1
**MPEG1.5**	5.7129	1.9497519962525	845	414	1.1425894	15	MPEG1
**HLA-DQA1.4**	1.4736	2.85036799432717	907	439	2.9473412	15	HLA-DQA1
**AIF1.3**	2.1853	2.41411305764406	858	384	4.3706562	15	AIF1
**HLA-DRB1.6**	8.3128	2.60343633000349	954	0.67	1.6625760	15	HLA-DRB1
**LYZ.3**	4.6090	3.09235243066043	898	596	9.2181121	15	LYZ
**TYROBP.2**	5.6455	1.80649142838437	858	0.41	1.1291137	15	TYROBP
**RAB11FIP1.4**	1.1001	1.90317308526671	913	577	2.2003986	15	RAB11FIP1
**CLEC4E.1**	5.3659	0.279485227040137	622	187	1.0731956	15	CLEC4E
**RP11-1143G9**	3.1807	1.55961877588593	793	444	6.3614352	15	RP11-1143G9
**LGALS2.4**	6.1452	2.47012753769994	755	289	1.2290580	15	LGALS2
**GPR183.2**	1.0162	2.17595275036465	0.91	0.54	2.0325107	15	GPR183
**SCT**	1.0202	0.875627103925726	616	0.16	2.0405548	15	SCT
**FCER1G.3**	6.2552	1.48049241899908	814	402	1.2510403	15	FCER1G
**WDFY4.1**	1.7386	1.51415174788048	759	377	3.4773011	15	WDFY4
**EGLN3**	9.9038	1.20590953329941	749	355	1.9807706	15	EGLN3
**FAM129C.1**	1.6247	0.536944761490846	653	0.16	3.2494368	15	FAM129C
**SPP1**	6.8197	0.402294041872064	616	172	1.3639518	15	SPP1
**IDO1.2**	6.4972	1.69806444917669	718	289	1.2994426	15	IDO1
**CLEC7A.1**	7.7114	0.903180588728182	0.7	242	1.5422836	15	CLEC7A
**CD86.1**	3.2749	1.00446397442579	715	309	6.5499082	15	CD86
**HLA-DMA.2**	9.5242	1.75616671173479	0.87	467	1.9048575	15	HLA-DMA
**MARCH1.2**	1.6937	0.974483028275347	743	348	3.3874191	15	MARCH1
**FCER1A.1**	2.2240	0.529040304518211	601	119	4.4480543	15	FCER1A
**PLEK.3**	4.0604	1.83162578213155	789	403	8.1208320	15	PLEK
**CXCL16.1**	2.4921	1.09212231095576	789	517	4.9843748	15	CXCL16
**SERPINA1.3**	2.3861	0.80021347973949	706	352	4.7723206	15	SERPINA1
**SHD**	2.4977	0.472419171843793	579	0.16	4.9955260	15	SHD
**VMO1**	9.2791	0.984567369535924	0.7	331	1.8558386	15	VMO1
**GREM1.5**	2.6389	-0.292742623186491	65	457	5.2778573	15	GREM1

**CD36.1**	3.2951	0.395981980302121	616	137	6.5902121	15	CD36
**CLEC10A.1**	3.8086	0.924480406903405	659	279	7.6172622	15	CLEC10A
**CPVL.4**	4.9298	2.83646957699606	755	406	9.8596542	15	CPVL
**JAML.5**	1.8650	1.33336847449412	0.78	437	3.7300041	15	JAML
**SHTN1**	1.8817	1.11468026357608	0.7	336	3.7634081	15	SHTN1
**TGFBI.6**	6.6410	1.10965245678637	799	479	1.3282148	15	TGFBI
**DSE.2**	3.5277	1.00333503959571	768	415	7.0554487	15	DSE
**HLA-DQA2.1**	6.3148	2.07329474178094	789	494	1.2629718	15	HLA-DQA2
**IFI30.1**	2.8535	1.11833351771244	706	355	5.7070088	15	IFI30
**C1QC.5**	9.9709	0.706522117988366	684	366	1.9941846	15	C1QC
**SLC7A11**	1.9556	0.54246683625483	697	0.34	3.9113096	15	SLC7A11
**HCK.1**	3.4975	0.866903847883589	675	237	6.9951549	15	HCK
**DAPP1**	6.0301	1.55893526218056	0.78	454	1.2060377	15	DAPP1
**ADAP2.2**	1.7910	0.530304215880265	706	0.36	3.5820573	15	ADAP2
**CD1E.1**	2.1188	0.350639155958069	511	54	4.2377193	15	CD1E
**LILRB2.1**	6.5114	0.387382791961947	687	397	1.3022845	15	LILRB2
**CYBB.5**	1.5603	1.09596896192295	721	381	3.1206811	15	CYBB
**NFKBID.3**	4.2482	1.13215640497561	854	597	8.4965626	15	NFKBID
**HMGA1.2**	1.6919	0.77193346024861	783	466	3.3839050	15	HMGA1
**LCP1.5**	1.8867	1.57096421681257	845	573	3.7734219	15	LCP1
**C1orf162.3**	3.8521	1.32769813573674	709	0.34	7.7043381	15	C1orf162
**CTSS.2**	2.7297	1.22197133718429	904	628	5.4595094	15	CTSS
**SPI1.3**	2.0162	1.66224430245566	706	322	4.0325203	15	SPI1
**CD68.2**	1.2580	0.9487233811934	771	512	2.5161933	15	CD68
**TNFSF13B.4**	1.5645	0.815109906477997	749	445	3.1290993	15	TNFSF13B
**GRN.3**	3.5966	1.19983866955155	0.92	616	7.1933812	15	GRN
**EPAS1.6**	4.6924	-1.5568135133833	344	721	9.3849765	15	EPAS1
**UCP2.3**	3.0720	1.51834128376509	786	495	6.1440460	15	UCP2
**GK.1**	4.3591	0.42433131204372	721	428	8.7182953	15	GK
**VAMP8.3**	3.1780	1.40280309845851	783	0.4	6.3561722	15	VAMP8
**S100B.4**	4.1765	2.35486559132125	659	285	8.3530077	15	S100B
**NABP1.1**	4.9328	0.908466247417946	786	473	9.8656878	15	NABP1

**CLEC9A**	1.0245	1.07881165633224	622	256	2.0490983	15	CLEC9A
**FPR3.1**	5.4401	0.346623933919018	647	376	1.0880298	15	FPR3
**MMP9.5**	1.1516	1.19909629414719	666	0.4	2.3033453	15	MMP9
**SLC8A1.1**	3.0439	0.550918857446105	703	408	6.0878648	15	SLC8A1
**C1R.4**	9.9814	-2.10406501216116	372	693	1.9962963	15	C1R
**ENPP2.4**	1.0290	0.477284188104916	721	434	2.0581898	15	ENPP2
**ASPN.5**	2.4278	-0.806011964329573	167	483	4.8556021	15	ASPN
**LRRC26**	1.2528	0.259087987016345	489	121	2.5056908	15	LRRC26
**HLA-DMB.2**	3.3123	1.36288102797433	718	347	6.6246203	15	HLA-DMB
**CD3E.3**	5.4431	-0.964220695640438	155	535	1.0886315	15	CD3E
**BCL11A.1**	6.3906	1.2168490452822	632	229	1.2781358	15	BCL11A
**CSF2RA.1**	1.0849	1.08627842390623	601	145	2.1699660	15	CSF2RA
**SMPD3**	1.2849	0.792579026809433	625	226	2.5699273	15	SMPD3
**RORB.6**	3.1435	-0.5337316204406	111	455	6.2870208	15	RORB
**RAB7B.1**	9.9110	0.652131209199924	619	258	1.9822058	15	RAB7B
**C15orf48.1**	3.1537	1.31338505268213	638	274	6.3074752	15	C15orf48
**ALDH2.2**	6.5837	1.27120797341843	712	322	1.3167441	15	ALDH2
**LSP1.3**	1.1708	1.57730440171897	771	513	2.3417543	15	LSP1
**CLNK**	2.7669	0.392568085209864	588	227	5.5338578	15	CLNK
**FPR1.1**	4.5301	0.48825272913969	641	371	9.0602680	15	FPR1
**CADM1**	6.8467	0.781491313113425	709	425	1.3693480	15	CADM1
**ITGB2.3**	3.2385	1.12139465666576	749	488	6.4771276	15	ITGB2
**TSPAN11.2**	4.1502	-0.260373929742847	176	459	8.3005320	15	TSPAN11
**SLAMF8.1**	4.4892	0.496221984158393	635	378	8.9785921	15	SLAMF8
**FAM26F.1**	2.2337	0.736313928538854	591	232	4.4674104	15	FAM26F
**PLXDC1.3**	2.1323	-0.375930166933768	99	457	4.2646593	15	PLXDC1
**MYBL2**	2.1474	0.640618958355178	582	215	4.2948635	15	MYBL2
**RGS13**	1.5186	0.317098988557994	628	301	3.0373336	15	RGS13
**EFNB2.2**	1.8627	-0.540042246726776	102	467	3.7255220	15	EFNB2
**BCL2A1.5**	3.6932	1.08743420461511	687	425	7.3864510	15	BCL2A1
**RASSF4.3**	8.5484	0.883690537548511	684	424	1.7096854	15	RASSF4
**LINC00996**	1.6573	0.428061828331553	594	0.25	3.3147480	15	LINC00996

**RGS1.2**	2.1298	0.962948795016543	833	565	4.2596293	15	RGS1
**IGFBP4.7**	3.6377	-2.22884645897723	471	731	7.2754110	15	IGFBP4
**MYCT1.5**	9.4082	-0.354557786425603	183	483	1.8816438	15	MYCT1
**ATF5.1**	1.9828	0.907986729092723	675	403	3.9657884	15	ATF5
**FCN1.2**	3.8343	0.54840773741859	585	258	7.6687813	15	FCN1
**ADIRF.8**	4.4416	-1.85792390458923	381	669	8.8833310	15	ADIRF
**GNA15.2**	8.0409	0.81703981298426	625	343	1.6081892	15	GNA15
**TM4SF1.6**	3.8047	-1.62063865848257	307	624	7.6095130	15	TM4SF1
**FAT1.7**	9.1600	-0.267401249535578	121	385	1.8320101	15	FAT1
**TINAGL1.4**	1.4948	-0.629669618684229	272	0.57	2.9897258	15	TINAGL1
**LTBP1.3**	1.8636	-0.650720655488768	325	578	3.7273171	15	LTBP1
**KIAA0101.2**	1.7242	0.318560622616626	594	311	3.4484691	15	KIAA0101
**PKP4.5**	2.3276	-0.522181307245332	285	571	4.6552279	15	PKP4
**CLEC4C**	4.1848	0.512971735851315	0.52	162	8.3697016	15	CLEC4C
**BICC1.3**	2.9459	-0.473695586943677	235	505	5.8918373	15	BICC1
**FBLN2.6**	3.2744	-1.41912174081227	226	535	6.5488473	15	FBLN2
**EFEMP2.3**	3.5190	-0.440425993136583	186	0.49	7.0381511	15	EFEMP2
**MNDA.1**	4.0868	0.990931022829703	576	294	8.1736798	15	MNDA
**APOLD1.4**	4.4008	-0.762472474779698	393	646	8.8016667	15	APOLD1
**PECAM1.6**	1.2978	-1.19103568629618	362	679	2.5957779	15	PECAM1
**RNASE6.1**	4.7788	0.903411911906773	598	296	9.5577790	15	RNASE6
**CD24.1**	1.4512	-0.545975203813208	99	354	2.9025731	15	CD24
**NCF2.1**	4.0444	0.31965132944213	563	298	8.0889067	15	NCF2
**LST1.3**	2.1490	1.24451362646032	594	286	4.2980369	15	LST1
**LBH.2**	7.9194	-0.723147375478447	211	523	1.5838902	15	LBH
**MYL9.5**	1.1836	-1.41618386107314	322	0.58	2.3673279	15	MYL9
**CD80**	1.7159	0.260078091672938	576	309	3.4318881	15	CD80
**BGN.4**	6.2759	-1.00194919212239	341	619	1.2551953	15	BGN
**ID4.5**	3.0600	-1.03933104949447	173	456	6.1201608	15	ID4
**ITGAX.1**	7.1518	0.460140592286587	548	255	1.4303724	15	ITGAX
**MTUS1.5**	3.7115	-0.601148848753221	238	514	7.4230462	15	MTUS1
**SPINT2.2**	3.8096	0.870488457442592	632	323	7.6192200	15	SPINT2

**VWA1.5**	5.182	-0.559035077826907	176	468	1.0364760	15	VWA1
**SPIB.1**	1.853	1.09797290039185	557	216	3.7076356	15	SPIB
**ABHD5**	2.916	-0.260823794273335	263	537	5.8332732	15	ABHD5
**BCAM.5**	6.932	-0.6337430901925	272	525	1.3865418	15	BCAM
**CD69**	1.112	-1.42981148267603	313	586	2.2252746	15	CD69
**APOD.4**	1.621	-1.41954625971586	251	504	3.2429763	15	APOD
**PRRX1.6**	1.139	-1.25034405281391	272	522	2.2795803	15	PRRX1
**SGCB.4**	1.064	-0.437572431952937	176	438	2.1293703	15	SGCB
**BST2.1**	7.388	0.497920824066558	817	538	1.4777697	15	BST2
**TNXB.5**	1.841	-0.432073948388292	272	537	3.6825647	15	TNXB
**SIGLEC10.1**	2.119	0.389875570604367	483	137	4.2395523	15	SIGLEC10
**PCOLCE.3**	4.542	-1.46046554762285	266	516	9.0840392	15	PCOLCE
**LY86.2**	3.043	0.71773733598245	529	246	6.0869536	15	LY86
**IGSF6.1**	2.5921	0.437310707313398	474	223	5.1842019	15	IGSF6
**LAMP5**	2.8941	0.263020935744604	514	207	5.7883136	15	LAMP5
**CD2.1**	5.344	-1.06929041780277	353	606	1.0688057	15	CD2
**ST14.2**	7.323	0.455901701527913	508	227	1.4647215	15	ST14
**TXNIP.1**	5.114	-0.606872519708879	415	674	1.0229236	15	TXNIP
**PKIB**	1.999	0.398424056183821	474	216	3.9988336	15	PKIB
**CRABP1.8**	3.236	-0.410688640074611	0.57	311	6.4727942	15	CRABP1
**TIFAB**	0.0002	0.328202762325866	359	63	0.4224316	15	TIFAB
**XXbac-BPG1**	0	0.842854536389351	734	49	0	16	XXbac-BPG1
**CCL1**	0	0.358514826248342	714	24	0	16	CCL1
**CMA1**	1.381	1.79223723236702	822	93	2.7621457	16	CMA1
**HPGDS.1**	7.443	3.95188980562513	914	94	1.4886674	16	HPGDS
**CPA3.5**	3.388	5.53171980464699	993	364	6.7771463	16	CPA3
**TPSAB1.2**	1.089	5.35629512199148	0.98	0.26	2.1791988	16	TPSAB1
**TPSB2.3**	2.230	7.55740140503348	997	482	4.4615936	16	TPSB2
**SLC18A2**	2.340	3.21726646961409	885	168	4.6806592	16	SLC18A2
**UTS2**	5.794	2.95350689115187	849	137	1.1589618	16	UTS2
**MS4A2**	9.1992	3.42586722836232	895	0.16	1.8398546	16	MS4A2
**HPGD.3**	4.793	4.5077506202652	928	309	9.5868063	16	HPGD

**RGS13.1**	1.2978	2.31663135513775	855	298	2.5956463	16	RGS13
**CLNK.1**	4.9661	1.35148056434015	812	224	9.9323269	16	CLNK
**FCER1G.4**	2.4440	3.03287551317201	918	401	4.8880673	16	FCER1G
**CTSG.2**	7.9191	4.89625503711672	888	467	1.5838305	16	CTSG
**PKIB.1**	3.3941	0.314612375709101	757	212	6.7883469	16	PKIB
**TYROBP.3**	2.7669	2.39184393002132	898	0.41	5.5338395	16	TYROBP
**AREG.2**	1.9468	4.34107126937504	908	0.64	3.8936729	16	AREG
**HDC**	8.4738	2.571609787008	789	0.31	1.6947793	16	HDC
**GATA2.4**	6.2841	2.78862016406907	855	484	1.2568255	16	GATA2
**ABL2.3**	3.4426	-0.678424423122278	69	618	6.8853920	16	ABL2
**CLU.3**	6.4929	2.21033686889791	931	614	1.2985808	16	CLU
**TNFRSF9.2**	1.8664	1.29559556685255	842	0.56	3.7328535	16	TNFRSF9
**LTC4S**	8.3005	2.50756423791778	783	0.23	1.6601088	16	LTC4S
**POSTN.2**	1.4681	-1.09011063906819	36	562	2.9363252	16	POSTN
**RGS2.3**	2.2836	2.31715288104479	905	615	4.5673466	16	RGS2
**TTN.1**	1.9745	-0.26775770595018	59	465	3.9491789	16	TTN
**ADGRF5.4**	4.7699	-0.669949944975547	125	587	9.5399989	16	ADGRF5
**ADAMTS1.3**	1.2134	-1.53290356491575	79	695	2.4269094	16	ADAMTS1
**GPR65**	5.0573	1.59602949931379	812	0.34	1.0114668	16	GPR65
**ELOVL7.7**	1.2276	-0.27331784869454	66	342	2.4552883	16	ELOVL7
**GRB10.4**	2.0487	-0.264680973838321	102	504	4.0975021	16	GRB10
**SLC12A8**	6.8566	0.40623957390678	734	292	1.3713304	16	SLC12A8
**MALL.3**	4.1854	-0.579414303986578	46	584	8.3708309	16	MALL
**APOC1.2**	9.0564	0.932982228250401	789	417	1.8112925	16	APOC1
**SH3PXD2A.2**	1.7376	-0.392075674539523	69	573	3.4752010	16	SH3PXD2A
**IL7R.1**	2.0634	-1.87634910796917	86	618	4.1268212	16	IL7R
**FILIP1L.7**	3.1826	-1.26654684419061	132	0.6	6.3652373	16	FILIP1L
**CYR61.2**	1.1678	-1.8207696140594	332	723	2.3356157	16	CYR61
**ARHGAP29.5**	3.8418	-0.699470263599776	86	538	7.6837387	16	ARHGAP29
**KIT**	6.7084	2.63012083519665	757	0.25	1.3416868	16	KIT
**ADAMTS9.3**	2.7012	-0.808571490245539	194	634	5.4025076	16	ADAMTS9
**HEY1**	5.0576	0.897055973643886	0.75	373	1.0115275	16	HEY1

**TPSD1**	1.5998	0.911518400324164	618	146	3.19974039	16	TPSD1
**ADAM12.2**	1.7017	0.740166055918216	773	369	3.40354523	16	ADAM12
**TNFSF13B.5**	3.7612	-0.275450901771824	79	455	7.52241014	16	TNFSF13B
**MT1M.7**	5.3437	-0.501776288820646	0.03	408	1.06874239	16	MT1M
**RGS1.3**	5.8573	2.05092828162541	868	565	1.17147569	16	RGS1
**ADIRF.9**	8.2637	-1.83778165710818	178	672	1.65275464	16	ADIRF
**PTPRB.7**	2.8650	-0.31895135521796	145	546	5.73010311	16	PTPRB
**ROBO4.7**	9.8713	-0.479715771312717	46	448	1.97426520	16	ROBO4
**ALOX5AP.2**	8.1355	1.98592970452166	816	439	1.62710693	16	ALOX5AP
**MASP1.7**	2.1102	-0.406573286504541	33	441	4.22051111	16	MASP1
**IGLV1-47.1**	7.5388	-0.258305072318645	0.74	272	1.50776949	16	IGLV1-47
**CAPG.2**	9.9817	2.22929494553667	786	378	1.99635885	16	CAPG
**ZNF385D.4**	1.1217	-0.642365869590234	36	509	2.24347045	16	ZNF385D
**GNG11.5**	4.8116	-1.32231186551503	112	0.59	9.62331113	16	GNG11
**SLIT3.9**	2.1122	-0.460918849883794	82	435	4.22440960	16	SLIT3
**LGALS3.5**	2.9417	2.0581126597862	951	695	5.88354504	16	LGALS3
**SYNPO2.4**	6.6864	-0.368060706900149	118	412	1.33729258	16	SYNPO2
**CD2.2**	2.3145	-1.39185482828832	69	0.61	4.62912305	16	CD2
**GPNMB.3**	7.0417	-0.55116247967781	181	568	1.40835101	16	GPNMB
**ANGPTL2.1**	1.9119	-0.630536131580019	92	669	3.82397849	16	ANGPTL2
**CPM.2**	1.9920	0.641045113623695	796	514	3.98410155	16	CPM
**TMEM176B.6**	3.6913	1.34153438235179	836	493	7.38271550	16	TMEM176B
**GEM.1**	8.7323	-0.586797636330931	0.22	598	1.74647913	16	GEM
**PDGFRB.8**	3.4035	-0.897289512849387	148	567	6.80705542	16	PDGFRB
**TNFAIP2.1**	3.6742	-0.716052853226893	266	0.64	7.34850438	16	TNFAIP2
**TGFBR3.2**	1.2504	-0.547961380542533	59	584	2.50090542	16	TGFBR3
**IGLV3-21.5**	1.8271	-0.506485067612807	717	0.34	3.65438351	16	IGLV3-21
**IL32.1**	2.1286	-1.99395026641062	388	0.72	4.25738871	16	IL32
**CAV1.7**	2.7712	-1.33038509855564	109	0.66	5.54245079	16	CAV1
**CRISPLD2.6**	3.1792	-0.71604891369474	197	577	6.35855605	16	CRISPLD2
**PLEKHA5.5**	1.9909	-0.425231782086355	49	497	3.98194479	16	PLEKHA5
**ADGRL4.7**	2.8018	-0.937733944224011	62	0.6	5.60366181	16	ADGRL4

**IGKV1-12.2**	3.8242	-0.320319384365734	0.74	382	7.6485401	16	IGKV1-12
**BCL2A1.6**	8.0126	0.712337168543437	0.76	425	1.6025225	16	BCL2A1
**CERCAM.1**	1.1202	-0.328194249085906	43	434	2.2404878	16	CERCAM
**PDLIM1.6**	1.6102	-1.28655804921403	168	643	3.2204586	16	PDLIM1
**KRT1**	3.7602	1.61311113901574	674	181	7.5204132	16	KRT1
**C1orf162.4**	5.0312	1.12834310211086	717	0.34	1.0062400	16	C1orf162
**RGS3.3**	9.4829	-0.456894719026583	168	593	1.8965879	16	RGS3
**SERPINB9.2**	1.4951	-0.54845866744439	0.25	619	2.9903448	16	SERPINB9
**RHOJ.5**	3.9071	-0.36519494747111	62	563	7.8143406	16	RHOJ
**MITF**	4.0958	0.997645210109083	681	291	8.1916147	16	MITF
**CPXM2.4**	8.5708	-0.782600868088575	72	0.65	1.7141707	16	CPXM2
**INHBB.3**	4.5271	-0.292825109022911	53	543	9.0542274	16	INHBB
**MIR202HG**	1.6137	0.420502071956207	266	7	3.2274966	16	MIR202HG
**FYB.3**	2.9683	-1.17863054948785	211	591	5.9367379	16	FYB
**ENAH.4**	6.7378	-0.572615256282099	39	0.48	1.3475662	16	ENAH
**IL1RL1**	1.2577	2.29166802224829	701	319	2.5154548	16	IL1RL1
**COL11A1.6**	1.5243	-0.403153917157147	753	406	3.0487690	16	COL11A1
**CCND1.3**	6.3838	-0.504809522535886	125	556	1.2767779	16	CCND1
**NBL1.1**	7.7876	-0.710334876688314	214	566	1.5575365	16	NBL1
**CLEC14A.7**	3.9839	-0.742709013032342	62	538	7.9678644	16	CLEC14A
**INSIG1.2**	1.2653	-0.275468721680423	214	621	2.5307361	16	INSIG1
**IGFBP6.3**	1.6883	-0.524916944392902	105	454	3.3766499	16	IGFBP6
**RGS5.3**	1.8748	-0.821109801590488	0.03	395	3.7496232	16	RGS5
**CD34.5**	4.8447	-0.735712222690075	207	517	9.6894323	16	CD34
**CLEC12A.1**	1.1068	0.417777822452567	612	145	2.2137336	16	CLEC12A
**GAS1.4**	2.0272	-0.907610878148632	102	511	4.0545759	16	GAS1
**ATP1B1.2**	3.8690	0.485631742167249	753	483	7.7380654	16	ATP1B1
**PECAM1.7**	4.0352	-1.58774417800827	395	678	8.0704725	16	PECAM1
**TINAGL1.5**	7.1124	-0.705054837990714	191	571	1.4224812	16	TINAGL1
**LYZ.4**	1.3744	-2.0042560686065	339	604	2.7488155	16	LYZ
**PAPPA.3**	2.0677	-0.585324519854356	46	576	4.1355278	16	PAPPA
**PODXL.3**	3.4708	-0.859100725965934	95	632	6.9417564	16	PODXL

**CRIP2.4**	3.5166	-0.982898837400642	253	614	7.0333882	16	CRIP2
**KCTD12.4**	4.5912	-0.911090778435551	99	602	9.1825313	16	KCTD12
**S100A13.5**	8.5482	-0.511924353763318	168	549	1.7096553	16	S100A13
**ACKR1.5**	9.1765	-1.84671673076119	227	0.56	1.8353075	16	ACKR1
**CDH5.4**	1.3431	-0.768681092048265	92	618	2.6862402	16	CDH5
**ITGA6.8**	2.4386	-1.27345063836977	257	627	4.8772346	16	ITGA6
**HEG1.5**	2.6280	-1.00901456199954	247	618	5.2561267	16	HEG1
**ECSCR.1.6**	2.8298	-0.840431468145035	86	582	5.6597977	16	ECSCR.1
**MAFB.2**	4.5294	-0.393553059799022	86	463	9.0588489	16	MAFB
**PRCP.3**	8.9251	-0.912500908422517	237	0.63	1.7850276	16	PRCP
**VASN.5**	9.3350	-0.313573371661956	39	485	1.8670093	16	VASN
**PKP4.6**	1.8586	-0.545957758089691	227	572	3.7173979	16	PKP4
**IL6.1**	5.7282	-0.859197063533032	257	674	1.1456407	16	IL6
**PRKCDBP.4**	6.0777	-0.642338499808439	79	0.51	1.2155566	16	PRKCDBP
**PLVAP.6**	6.9056	-1.09864382066613	66	588	1.3811213	16	PLVAP
**TSPAN7.4**	7.4236	-0.51223390201564	53	477	1.4847229	16	TSPAN7
**ID3.1**	1.1682	-1.25594366045465	309	676	2.3364293	16	ID3
**S1PR1.7**	1.3173	-0.368759155779876	247	534	2.6347431	16	S1PR1
**EIF4E**	1.8719	-0.35794218979918	105	491	3.7439880	16	EIF4E
**SLCO2A1.4**	1.8732	-0.894638562877738	102	636	3.7464001	16	SLCO2A1
**VAMP8.4**	6.4109	1.19449934943909	0.78	401	1.2821969	16	VAMP8
**CCL21.2**	9.1158	-1.59161458600878	53	462	1.8231614	16	CCL21
**BST2.2**	2.8177	1.31849704444027	882	538	5.6355566	16	BST2
**ANGPT1.3**	5.3755	-0.312227646636563	66	486	1.0751128	16	ANGPT1
**ODC1**	2.6664	-0.491856423890797	283	688	5.3329644	16	ODC1
**TGM2.2**	3.5586	-0.595071834409441	118	621	7.1172756	16	TGM2
**CLEC11A.3**	1.2407	-0.400891140024083	49	473	2.4815898	16	CLEC11A
**CD8A.4**	1.9478	-0.8927527815452	92	584	3.8957893	16	CD8A
**MFGE8.2**	4.5124	-0.656258804880021	155	497	9.0249415	16	MFGE8
**ACKR3.3**	4.5390	-0.562845641046279	95	0.62	9.0780856	16	ACKR3
**EGFL6.1**	7.4918	-0.737840338203344	82	583	1.4983690	16	EGFL6
**PERP.3**	2.6388	-0.813373452197785	105	478	5.2777332	16	PERP

**CCL4L2.4**	3.4604	-1.14356673230286	164	539	6.92082408	16	CCL4L2
**NPDC1.6**	4.5801	-1.06354595702699	138	567	9.16021155	16	NPDC1
**RP11-354E11**	2.0983	1.88280913181861	615	152	4.19669419	16	RP11-354E11
**ADCYAP1**	7.2342	2.93931872324266	671	338	1.44685267	16	ADCYAP1
**PCOLCE.4**	7.9984	-1.3910381270899	59	519	1.59968820	16	PCOLCE
**TIGIT.2**	1.4504	-0.607679491084086	86	586	2.90095587	16	TIGIT
**BGN.5**	1.6660	-0.95818114426538	89	623	3.33204748	16	BGN
**ADGRG1.3**	5.4187	-0.371472061818094	59	571	1.08374224	16	ADGRG1
**RORB.7**	5.5346	-0.384570405391514	0.75	445	1.10693290	16	RORB
**CYP1B1.3**	7.3061	-0.697357404132645	102	571	1.46122211	16	CYP1B1
**C2CD4B.6**	1.9952	-0.689328095763754	0.27	543	3.99041332	16	C2CD4B
**ISLR.7**	2.4049	-0.304788615635634	737	373	4.80988323	16	ISLR
**IFI27.5**	2.7119	-1.69131288094131	207	609	5.42381954	16	IFI27
**STAT1.1**	3.5714	-0.279587585933411	257	586	7.14296720	16	STAT1
**TMEM176A.6**	3.7129	0.361258642781337	793	506	7.42596350	16	TMEM176A
**PTGDS.4**	6.1603	-1.66392456997228	191	559	1.23206604	16	PTGDS
**CD82.1**	6.2037	0.937969390159817	766	471	1.24075270	16	CD82
**CEBPD.8**	1.0783	-1.65060754650697	0.24	633	2.15660540	16	CEBPD
**ITGB2.4**	1.1903	-0.549526517131475	171	497	2.38067437	16	ITGB2
**TWIST2.4**	2.9125	-0.653055485494865	86	582	5.82515491	16	TWIST2
**CARHSP1.4**	2.9377	-0.267175667771299	197	538	5.87543329	16	CARHSP1
**CTLA4.4**	3.0968	-0.345542015636512	72	0.47	6.19365695	16	CTLA4
**CCL5.1**	7.8639	-1.94254158815142	115	594	1.57279915	16	CCL5
**ICAM2.4**	9.5833	-0.653962409656785	247	0.54	1.91667661	16	ICAM2
**INPP4B.2**	2.4535	-0.275814787163353	737	389	4.90714745	16	INPP4B
**HLA-DMA.3**	3.2995	-0.772950231279685	141	478	6.59913472	16	HLA-DMA
**CXCL13.2**	7.2448	-2.24014901722766	395	647	1.44896443	16	CXCL13
**S100A16.7**	1.0086	-0.974754948501866	62	519	2.01728785	16	S100A16
**EGFL7.9**	1.3028	-0.603450223728898	125	435	2.60571195	16	EGFL7
**G0S2**	1.6367	-0.440906771719069	757	474	3.27349389	16	G0S2
**F2R.2**	4.3727	-0.7220553577306	273	595	8.74549622	16	F2R
**CP.6**	5.8248	-0.275292702629842	747	451	1.16496134	16	CP

**SELP.5**	6.3061	-0.683914796585955	69	553	1.2612393	16	SELP
**NRP2.2**	6.4780	-0.361597362286211	0.25	523	1.2956179	16	NRP2
**C1orf186**	6.5625	2.33405224241996	645	261	1.3125049	16	C1orf186
**MT1X.2**	9.1276	-0.79001010226396	204	526	1.8255244	16	MT1X
**STEAP1.5**	9.4813	-0.369594270035312	0.75	414	1.8962601	16	STEAP1
**WNT5A.4**	2.4420	-1.17484121248287	69	582	4.8840549	16	WNT5A
**CYBB.6**	3.7006	-0.319508106551683	46	391	7.4013408	16	CYBB
**LTBP1.4**	7.6503	-0.605787627697915	76	581	1.5300648	16	LTBP1
**TRBC2.1**	1.2809	-1.50988697791052	299	576	2.5618021	16	TRBC2
**ICAM1.3**	1.3628	-1.03269148907137	487	0.74	2.7256598	16	ICAM1
**CD200.7**	2.0244	-0.513034119288681	56	533	4.0489398	16	CD200
**SERPINF1.6**	1.3829	-1.06177781618846	266	566	2.7659941	16	SERPINF1
**ADM.1**	1.4469	-0.579501110763345	89	569	2.8939231	16	ADM
**CPE.5**	3.5195	-0.81271127350269	66	0.53	7.0391836	16	CPE
**FMOD.3**	7.1889	-0.884326158194933	92	577	1.4377958	16	FMOD
**CXCL14.4**	1.9895	-1.5780747400406	95	415	3.9791367	16	CXCL14
**CHN1.2**	1.3408	-0.640681225724745	118	598	2.6817044	16	CHN1
**SERPINE2.4**	1.4414	-0.289195432172986	194	448	2.8828608	16	SERPINE2
**MIR4435-2H**	1.7065	-0.303331369054486	303	553	3.4130484	16	MIR4435-2H
**ADAMTS4.5**	2.6690	-0.459548114538065	102	559	5.3380820	16	ADAMTS4
**LRRC15.7**	4.9274	-0.29205090340675	82	493	9.8549849	16	LRRC15
**ID2**	1.1678	0.764176300037587	845	582	2.3356588	16	ID2
**MEOX1.4**	2.6828	-0.352288659348307	26	382	5.3656996	16	MEOX1
**IGF2.5**	7.7770	-1.05515135387238	72	0.56	1.5554176	16	IGF2
**ICOS.2**	5.5198	-0.542589003623563	115	538	1.1039795	16	ICOS
**LPAR1.2**	6.9164	-0.341040908954794	46	0.48	1.3832978	16	LPAR1
**BICC1.4**	6.9519	-0.460968863261168	76	507	1.3903978	16	BICC1
**PREX2.3**	9.3229	-0.370935239293474	753	425	1.8645986	16	PREX2
**DIO2.2**	9.7890	-1.38377238985709	105	575	1.9578004	16	DIO2
**CRYBG3.6**	2.1114	-0.54362815173567	283	557	4.2229917	16	CRYBG3
**PCDH17.8**	2.1543	-0.786906626675868	109	558	4.3087224	16	PCDH17
**TNXB.6**	2.3889	-0.448948780057995	79	539	4.7779873	16	TNXB

**CXCL6**	8.8481	-0.257273493764373	102	521	1.76962808		16	CXCL6
**CXorf36.6**	9.2721	-0.371435204444333	86	464	1.85442841		16	CXorf36
**FILIP1.5**	1.1971	-0.261209918052837	674	339	2.39437295		16	FILIP1
**CD8B.1**	1.9335	-0.444502288867465	72	497	3.86710088		16	CD8B
**THBD.4**	2.8613	-0.830798351403856	105	0.56	5.72274327		16	THBD
**DAPP1.1**	4.1827	-0.270280970161195	72	465	8.36546985		16	DAPP1
**FRZB.7**	6.9851	-0.49003046499169	62	412	1.39703177		16	FRZB
**CPXM1.3**	8.0281	-0.2616859996234	99	518	1.60562332		16	CPXM1
**NOSTRIN.6**	8.9760	-0.442475584654129	33	484	1.79520584		16	NOSTRIN
**GZMH.3**	1.1478	-0.445225008488415	69	493	2.29571399		16	GZMH
**GZMK.1**	1.2611	-1.33830027216924	342	593	2.52225547		16	GZMK
**CDKN1C**	1.6128	-0.288401162469062	174	482	3.22563060		16	CDKN1C
**MMP9.6**	4.6096	-0.286103089036189	79	409	9.21939170		16	MMP9
**MRC2.5**	5.2832	-0.560453140687233	43	444	1.05665560		16	MRC2
**LTB.3**	2.3547	-0.99267014110893	138	488	4.70950193		16	LTB
**DSP.2**	3.0852	-0.483708399341352	89	345	6.17041872		16	DSP
**PDGFB.3**	6.2744	-0.29237789471305	92	529	1.25489452		16	PDGFB
**RND1.2**	9.1996	-0.326671556168111	79	534	1.83993942		16	RND1
**HMCN1.4**	9.9650	-0.302519356118736	72	478	1.99300978		16	HMCN1
**EMCN.8**	2.7649	-0.791630284422102	59	533	5.52999878		16	EMCN
**CXCL10**	2.7993	-0.297051323106196	62	473	5.59866802		16	CXCL10
**MS4A1.5**	3.0664	-0.395161222642357	69	376	6.13293249		16	MS4A1
**STEAP2.6**	3.8734	-0.34583277672872	89	486	7.74690520		16	STEAP2
**GSN.6**	4.8230	-1.04006654326252	411	699	9.64617558		16	GSN
**ALPL.5**	6.8073	-0.416977532642298	109	513	1.36146227		16	ALPL
**THY1.4**	1.5689	-1.26596581918595	53	501	3.13792411		16	THY1
**SFRP1.6**	1.9917	-0.332601909487347	59	435	3.98349580		16	SFRP1
**APLNR.4**	2.3495	-0.386078289770133	56	478	4.69913129		16	APLNR
**CLDN5.8**	3.7928	-1.21958756592041	56	0.45	7.58572693		16	CLDN5
**S100A2.3**	4.3140	-0.92835874295739	148	401	8.62806291		16	S100A2
**ADAMTS2.7**	6.5074	-0.439471679889021	53	486	1.30148598		16	ADAMTS2
**MYLK.3**	6.6566	-0.778881067568816	86	556	1.33133654		16	MYLK

**HSPB1.6**	6.7806	-0.647392684952624	322	621	1.35612748	16	HSPB1
**MYCT1.6**	8.3654	-0.292227576622797	43	485	1.67309143	16	MYCT1
**IGHGP.2**	1.0720	-0.388834258190121	625	324	2.14402055	16	IGHGP
**HLA-DQA2.2**	2.6212	-0.427976866309837	0.76	495	5.24255550	16	HLA-DQA2
**TSPAN11.3**	2.8935	-0.275193872652798	82	461	5.78717720	16	TSPAN11
**SPOCK2.2**	5.1933	-0.806006004239457	109	543	1.03866395	16	SPOCK2
**PTK7.3**	5.7424	-0.397083078260727	59	516	1.14849423	16	PTK7
**PCSK5.3**	7.7520	-0.436640964856933	102	0.53	1.55041270	16	PCSK5
**CNKSR3.4**	8.3074	-0.397095099975804	56	499	1.66149668	16	CNKSR3
**CPVL.5**	1.2794	-0.339075141515778	89	417	2.55891364	16	CPVL
**RASSF4.4**	1.6480	-0.293474161123787	132	432	3.29606724	16	RASSF4
**PLXDC1.4**	1.6911	-0.387077493818542	0.03	458	3.38226680	16	PLXDC1
**IL1B.3**	2.3610	-0.76765769688678	39	394	4.72217844	16	IL1B
**GZMA.3**	4.2357	-1.14590551776829	283	538	8.47142089	16	GZMA
**TM4SF18.5**	5.3635	-0.473724905279624	43	489	1.07271678	16	TM4SF18
**PLOD2.4**	6.2161	-0.341270469698889	89	498	1.24322904	16	PLOD2
**FST.4**	6.8500	-0.426403312220858	72	451	1.37001702	16	FST
**IGFBP3.3**	6.9640	-0.530828237251852	66	485	1.39280190	16	IGFBP3
**LIMS2.1**	8.5922	-0.464470708525792	95	544	1.71844623	16	LIMS2
**ITPKC.1**	2.1962	-0.260959774601686	82	525	4.39246004	16	ITPKC
**TEK.7**	5.1790	-0.251618697514583	59	0.45	1.03580903	16	TEK
**OGN.4**	5.2855	-0.276093741439188	59	405	1.05710598	16	OGN
**IRF8**	5.7994	-0.304941662993087	39	445	1.15988522	16	IRF8
**RCAN1.4**	5.9468	-0.722278566584639	122	536	1.18936702	16	RCAN1
**PPFIBP1.5**	7.3882	-0.549420285972559	72	472	1.47764848	16	PPFIBP1
**ADM5.4**	1.1663	-0.283173997288986	79	449	2.33269247	16	ADM5
**ANGPT2.2**	1.2891	-0.363545626751202	49	392	2.57829562	16	ANGPT2
**FBXO32.3**	2.5696	-0.392858136579894	62	465	5.13928744	16	FBXO32
**IGKV1-5.2**	3.2022	-0.330289039588589	651	369	6.40458520	16	IGKV1-5
**ITGB5.2**	3.3687	-0.546288798294432	53	0.5	6.73755294	16	ITGB5
**BMP2.2**	4.9795	-0.327671750625552	224	548	9.95902367	16	BMP2
**DUSP23.3**	5.3438	-0.308807048680885	234	486	1.06876905	16	DUSP23

**FAM167B.6**	7.2018	-0.330398138849803	59	473	1.4403743		16	FAM167B
**RAMP2.7**	8.4236	-0.991928320699761	102	0.49	1.6847368		16	RAMP2
**FLT1.6**	1.0734	-0.774996441098547	69	503	2.1469473		16	FLT1
**ESAM.5**	1.1217	-0.580429834306454	89	537	2.2434846		16	ESAM
**TNS3.2**	1.1588	-0.309664042098669	66	508	2.3177883		16	TNS3
**SPRR2A.2**	1.3719	-1.64428835542296	128	388	2.7438543		16	SPRR2A
**PALMD.6**	1.4963	-0.987394790652009	95	555	2.9926849		16	PALMD
**EMP2.5**	1.5039	-0.744353260264477	76	534	3.0078501		16	EMP2
**IRF4.2**	2.1877	-0.255447547998423	89	462	4.3755389		16	IRF4
**PDPN.1**	2.4605	-0.314289322394557	49	434	4.9210337		16	PDPN
**LOXL2.1**	3.1576	-0.402056964713082	79	502	6.3152104		16	LOXL2
**PTGFR.7**	8.1816	-0.276217825531326	59	428	1.6363243		16	PTGFR
**TAGLN.4**	9.3497	-1.41918222633374	105	459	1.8699598		16	TAGLN
**CDH13.3**	1.0633	-0.352384514916728	72	507	2.1266453		16	CDH13
**IRF1**	1.1454	-0.410926863058329	487	793	2.2908496		16	IRF1
**KDR.5**	1.1814	-0.425936505031702	0.74	452	2.3629407		16	KDR
**INHBA.6**	2.0180	-0.315228359412628	69	431	4.0361149		16	INHBA
**XCL2.4**	2.5810	-0.443215795609828	59	416	5.1621188		16	XCL2
**GPX3.3**	2.7158	-0.307863822172626	59	488	5.4317875		16	GPX3
**IL34.4**	5.3534	-0.288264406147113	62	449	1.0706925		16	IL34
**FAM107A.8**	1.1110	-0.522496430874846	89	474	2.2221057		16	FAM107A
**CXCL1.3**	1.2752	-1.23313843760836	95	519	2.5505896		16	CXCL1
**ASPN.6**	2.3545	-0.68158162044177	92	483	4.7091845		16	ASPN
**PRRX1.7**	3.8536	-1.24827904248374	86	524	7.7073521		16	PRRX1
**MX1**	6.2874	-0.308974069847005	76	364	1.2574814		16	MX1
**MAP1B.6**	1.1687	-0.964709251263165	207	532	2.3375901		16	MAP1B
**LIF**	1.2323	0.796218061193545	553	285	2.4646350		16	LIF
**DNASE1L3.5**	1.4528	-0.611861187964161	95	399	2.9056413		16	DNASE1L3
**HYAL2.5**	1.5559	-0.957631243458304	0.24	506	3.1118854		16	HYAL2
**MAL.1**	1.8460	-0.462095565377087	59	322	3.6921603		16	MAL
**KIAA1217.6**	1.9539	-0.347793845649101	46	458	3.9078117		16	KIAA1217
**LOXL1.2**	2.9813	-0.290161562682606	79	485	5.9627864		16	LOXL1

**SNCG.3**	4.1110	-0.470394125496593	56	467	8.2221693	16	SNCG
**NR2F2.5**	4.6809	-1.27453509372624	89	543	9.3618076	16	NR2F2
**MECOM.6**	5.0343	-0.299164530418903	49	411	1.0068669	16	MECOM
**SPNS2.4**	6.6644	-0.426926538249985	79	494	1.3328813	16	SPNS2
**KRT17.4**	1.4430	-0.556592787502121	53	359	2.8860965	16	KRT17
**COL5A3.6**	2.9622	-0.298777730972934	79	466	5.9244343	16	COL5A3
**PLXNA2.5**	3.0264	-0.313707216462451	72	449	6.0529450	16	PLXNA2
**OLFML3.4**	5.4261	-0.38296556846806	72	0.46	1.0852303	16	OLFML3
**IL1RN.1**	1.4287	-0.285581703814449	0.5	212	2.8574710	16	IL1RN
**MMRN2.7**	1.8758	-0.625779690601707	46	451	3.7517725	16	MMRN2
**NET1.2**	2.5360	-0.365021098956258	36	441	5.0720328	16	NET1
**SFRP4.3**	4.3303	-0.386147172007732	79	411	8.6607521	16	SFRP4
**TNC.8**	8.4290	-0.2689362780776	56	423	1.6858060	16	TNC
**OLFM1.3**	1.6243	-0.395645908049521	36	403	3.2487362	16	OLFM1
**OMD.7**	1.9164	-0.358312983107031	69	424	3.8328419	16	OMD
**SPON2.5**	3.2489	-0.378229861945314	49	439	6.4979485	16	SPON2
**TBX2.3**	5.2804	-0.301250221834379	89	449	1.0560934	16	TBX2
**RP11-1143G**	5.7847	-0.306793711403457	56	455	1.1569574	16	RP11-1143G
**MOXD1.4**	8.4082	-0.308959212446011	69	403	1.6816530	16	MOXD1
**TBX3.1**	8.7133	-0.277873760845063	69	465	1.7426789	16	TBX3
**BCAM.6**	1.2062	-0.705144239535998	135	526	2.4125548	16	BCAM
**RAMP3.6**	1.3874	-0.866306053878718	95	497	2.7748478	16	RAMP3
**CLEC2B**	1.8058	-0.31754461015277	0.27	523	3.6117473	16	CLEC2B
**SGIP1.6**	5.3919	-0.316180221455884	0.75	498	1.0783933	16	SGIP1
**SMOC2.3**	6.5805	-0.358974350903	36	353	1.3161184	16	SMOC2
**ITIH5.1**	7.7842	-0.287387742724783	62	456	1.5568440	16	ITIH5
**VWA1.6**	8.2721	-0.708658814048604	49	0.47	1.6544299	16	VWA1
**CCDC3.5**	1.1005	-0.467484100672403	69	474	2.2010436	16	CCDC3
**CD248.3**	1.4618	-0.326650029680622	66	442	2.9237128	16	CD248
**CRABP2.7**	2.4123	-0.562262123350882	36	395	4.8247070	16	CRABP2
**COL16A1.3**	3.0015	-0.341208842987555	46	448	6.0031531	16	COL16A1
**CHI3L2.4**	7.5083	-0.534651731756952	62	332	1.5016782	16	CHI3L2

**ITGA8.3**	1.0688	-0.275925167347555	76	442	2.13768763	16	ITGA8
**IFNG.3**	1.2663	-0.363916918806651	89	414	2.53279312	16	IFNG
**KYNU.4**	6.4796	-0.357776348359331	66	447	1.29593750	16	KYNU
**CYGB.4**	7.9514	-0.274325788007665	62	0.46	1.59029878	16	CYGB
**PLAC8.3**	9.7889	-0.306184974671087	592	321	1.95779371	16	PLAC8
**IGLV3-1.6**	1.5261	-0.704422308134111	49	379	3.05225788	16	IGLV3-1
**SDC1.5**	2.7759	-0.2956135515766	743	457	5.55198638	16	SDC1
**PSD3.1**	4.1719	-0.394038260409208	59	449	8.34388909	16	PSD3
**MME.4**	4.9951	-0.435294789466798	66	417	9.99026645	16	MME
**COL5A1.3**	7.9989	-0.91654915177702	224	529	1.59979204	16	COL5A1
**COL17A1.3**	1.8485	-0.3338473401667	53	0.33	3.69703999	16	COL17A1
**WISP2.5**	3.2300	-0.261673112718048	36	363	6.46000618	16	WISP2
**MPZL2.9**	4.5220	-0.386954348504579	82	0.44	9.04415754	16	MPZL2
**C3.4**	5.5947	-0.425314666001324	59	423	1.11894259	16	C3
**COMP.2**	7.4949	-0.253460609162433	66	382	1.49898603	16	COMP
**FHL2.2**	1.2114	-0.252598578036729	76	452	2.42297887	16	FHL2
**IGKV4-1.3**	2.2914	-0.364052895876334	82	391	4.58298499	16	IGKV4-1
**FZD4.3**	4.7831	-0.304232477176581	82	393	9.56621997	16	FZD4
**SLC9A3R2.2**	5.7186	-0.523476687644922	62	435	1.14373821	16	SLC9A3R2
**SDC2.8**	7.3903	-0.408781431610721	658	363	1.47807297	16	SDC2
**PRF1.2**	1.3994	-0.282420638400788	69	387	2.79895509	16	PRF1
**FBLN5.5**	1.5248	-0.37061600693304	59	426	3.04978514	16	FBLN5
**C10orf10.6**	1.9422	-0.518549414191786	678	0.41	3.88453531	16	C10orf10
**CCDC80.3**	2.8620	-0.98463284977424	105	483	5.72405873	16	CCDC80
**CRABP1.9**	3.6653	-0.423721005618416	56	319	7.33077598	16	CRABP1
**FGF7.4**	4.6825	-0.642524164936173	655	398	9.36515874	16	FGF7
**LIFR.3**	7.2841	-0.422044289774408	79	447	1.45683774	16	LIFR
**NOTCH3.4**	1.1435	-0.571187045764316	658	0.38	2.28717960	16	NOTCH3
**MFAP4.4**	1.1568	-0.542322044632181	102	438	2.31366540	16	MFAP4
**JAG2.2**	1.7804	-0.25573276149783	53	377	3.56099712	16	JAG2
**CYYR1.3**	4.3539	-0.431430775782064	49	424	8.70791584	16	CYYR1
**HLA-DMB.3**	5.4394	-0.518360144615652	62	357	0.00010878	16	HLA-DMB

**CFI.5**	6.3799	-0.350414741285867	0.74	426	0.0001275	16	CFI
**SOX18.5**	7.9248	-0.592810633259934	102	462	0.0001584	16	SOX18
**THSD7A.9**	1.0798	-0.550987085244932	76	422	0.0002159	16	THSD7A
**DSEL.3**	1.4918	-0.404280426535295	207	486	0.0002983	16	DSEL
**PLAC9.3**	2.0126	-0.435053515845847	0.25	504	0.0004025	16	PLAC9
**SOX17.3**	3.3855	-0.275175168263747	59	329	0.0006771	16	SOX17
**NES.7**	4.2500	-0.359830239547719	56	372	0.0008500	16	NES
**SGCB.5**	2.0502	-0.465135147330337	79	439	0.0041005	16	SGCB
**ABCB1.3**	3.6237	-0.291175402535931	69	398	0.0072475	16	ABCB1
**PPP1R14A.2**	4.4192	-0.374527958650821	62	358	0.0088384	16	PPP1R14A
**NOTCH4.1**	4.6917	-0.320070465551434	69	408	0.0093835	16	NOTCH4
**IGLL5.3**	4.8689	-0.886426778463285	43	329	0.0097378	16	IGLL5
**FAM13C.5**	5.1563	-0.381814956567248	49	374	0.0103126	16	FAM13C
**RCN3.4**	5.6080	-0.308179356700901	36	403	0.0112160	16	RCN3
**LIMCH1.2**	1.0873	-0.291609808236598	76	419	0.0217479	16	LIMCH1
**TACSTD2.2**	0.0001	-0.282341094152231	39	307	0.2486556	16	TACSTD2
**GJA4.2**	0.0004	-0.365366841389694	46	318	0.9889676	16	GJA4
**RCAN2.3**	0.0006	-0.308309369127233	23	358	1	16	RCAN2
**SORBS2.3**	0.0008	-0.322624577464268	95	397	1	16	SORBS2
**CYP26B1.5**	0.0028	-0.300644051809299	43	324	1	16	CYP26B1
**ITGBL1.5**	0.0047	-0.520136375193597	36	359	1	16	ITGBL1
**KLK12**	0	2.68477024944255	989	0.15	0	17	KLK12
**IVL**	0	1.17303559796688	985	163	0	17	IVL
**SLC5A1**	0	0.890016756779996	967	135	0	17	SLC5A1
**LINC01214**	0	0.751903995476284	945	49	0	17	LINC01214
**IL36RN**	0	0.499044886801505	978	91	0	17	IL36RN
**CTC-518B2.8**	0	0.403981503912476	985	117	0	17	CTC-518B2.8
**SMIM22**	0	0.279271865597099	982	88	0	17	SMIM22
**HCAR3.2**	7.6743	0.28381409920673	978	157	1.5348759	17	HCAR3
**FAM83A**	2.7233	1.71776833065709	916	91	5.4466584	17	FAM83A
**RP11-60L3.1**	1.8244	0.273321169164703	887	149	3.6489330	17	RP11-60L3.1
**PRSS3**	2.2376	3.09778165540629	971	192	4.4752667	17	PRSS3

**ALOX12**	7.9167	0.905118228007725	898	0.17	1.5833428	17	ALOX12
**TMPRSS4**	1.6098	0.75960229108127	865	97	3.2197332	17	TMPRSS4
**GJB5.1**	3.0467	0.431005884295443	876	121	6.0934839	17	GJB5
**CLCA2.2**	2.4026	0.940677134205782	982	155	4.8053687	17	CLCA2
**FGFBP1.2**	5.7386	1.43340241212034	985	192	1.1477288	17	FGFBP1
**SCEL**	9.5514	1.57654052239993	934	156	1.9102946	17	SCEL
**TGM3**	4.1799	2.09988730033057	916	136	8.3599120	17	TGM3
**SERPINB3.2**	3.2465	2.78606521932088	985	317	6.4931926	17	SERPINB3
**SERPINB4.2**	3.4630	2.01220052167775	934	218	6.9261380	17	SERPINB4
**CRCT1.3**	2.7967	8.2720780171434	982	172	5.5934087	17	CRCT1
**S100A8.2**	9.7072	6.92091787285767	1	503	1.9414419	17	S100A8
**S100A9.3**	1.0211	7.10280939481163	1	0.54	2.0422307	17	S100A9
**KRT17.5**	2.7435	4.39962087021976	993	346	5.4871166	17	KRT17
**SPRR2A.3**	2.6497	7.38019466292756	993	376	5.2995362	17	SPRR2A
**CDA**	3.4854	2.12003581716386	1	321	6.9709793	17	CDA
**S100A7.1**	7.3790	7.39172504921505	993	382	1.4758077	17	S100A7
**SERPINB5.2**	4.3409	1.31120937212703	974	238	8.6819739	17	SERPINB5
**KRTDAP**	1.0628	3.265578985759	927	106	2.1256893	17	KRTDAP
**PROM2.1**	2.9333	0.659646914641611	931	203	5.8666045	17	PROM2
**ELF3.1**	6.8319	2.087273922846	934	184	1.3663959	17	ELF3
**SPRR1B.2**	1.4348	5.58669287975904	964	222	2.8697094	17	SPRR1B
**KRT6A.3**	1.5191	4.57960473195604	989	381	3.0383586	17	KRT6A
**TMPRSS11E**	1.0180	2.07099939470875	934	252	2.0361513	17	TMPRSS11E
**KRT19.3**	4.4218	3.48212681913744	974	0.25	8.8437031	17	KRT19
**SERPINB2.1**	6.6196	0.645418983241328	891	153	1.3239224	17	SERPINB2
**S100A14.3**	5.6968	4.79208629354624	971	0.24	1.1393720	17	S100A14
**CSTB.1**	1.7001	6.17414426807269	993	621	3.4003902	17	CSTB
**TTC22**	1.0946	0.482066338621392	909	218	2.1892614	17	TTC22
**KRT13.2**	2.7470	5.27987098861868	964	415	5.4941685	17	KRT13
**TGFA**	3.4411	0.647815074710623	927	249	6.8823055	17	TGFA
**CLIC3**	1.1738	2.4518031025105	967	303	2.3476067	17	CLIC3
**OMD.8**	6.3431	-0.466912092205444	15	424	1.2686336	17	OMD

**SPRR2D.3**	8.5924	6.98090126107509	949	265	1.7184889	17	SPRR2D
**ADRA2A.4**	4.1252	-0.38010597130133	15	319	8.2504516	17	ADRA2A
**CYSRT1**	1.1248	2.13774809396326	894	141	2.2496030	17	CYSRT1
**ADGRF1**	8.4484	0.883181972052097	755	72	1.6896928	17	ADGRF1
**CA2.2**	1.7167	0.311394204921357	974	375	3.4335501	17	CA2
**CLDN4**	1.0182	1.81774309269848	923	0.2	2.0364364	17	CLDN4
**IL18.2**	1.2106	2.03224335867575	956	231	2.4213694	17	IL18
**CRNN**	2.9854	3.78375514859885	912	154	5.9708668	17	CRNN
**PI3**	8.9735	2.61816339503025	0.92	0.39	1.7947162	17	PI3
**TMPRSS11D.**	1.0760	1.9693221773669	909	214	2.1521432	17	TMPRSS11D
**IL1A.1**	3.3009	1.59723053757768	887	163	6.6019936	17	IL1A
**SLC26A9**	5.2541	0.31778192043127	807	139	1.0508374	17	SLC26A9
**SPRR3**	5.3107	8.80282490935093	942	359	1.0621537	17	SPRR3
**SBSN.2**	8.2634	3.9885276394605	0.92	183	1.6526839	17	SBSN
**ANKRD37.2**	3.1004	2.01281595213581	993	483	6.2009197	17	ANKRD37
**F13A1.5**	1.2989	-0.311133948862692	36	422	2.5979724	17	F13A1
**GJB6**	4.5487	1.14931741920495	898	124	9.0974643	17	GJB6
**SLC2A1.1**	6.0003	0.488763064787313	985	506	1.2000782	17	SLC2A1
**PRRX1.8**	3.7436	-1.41310443105017	47	524	7.4873508	17	PRRX1
**FAM25A.2**	8.2183	4.44685332531622	858	75	1.6436796	17	FAM25A
**C5orf66-AS1.**	3.7174	0.834623085792538	832	128	7.4349554	17	C5orf66-AS1
**SPINT1.1**	1.0270	1.50747725734508	909	228	2.0541510	17	SPINT1
**COL6A1.4**	1.4166	-2.88452412951146	91	668	2.8333444	17	COL6A1
**S100A12.1**	3.3849	1.85778789287032	818	128	6.7698131	17	S100A12
**ANXA3.1**	5.0141	0.569174712461757	869	175	1.0028248	17	ANXA3
**CD248.4**	9.7569	-0.399644139360661	29	442	1.9513936	17	CD248
**CNFN.1**	1.5697	5.91732321435725	909	181	3.1394824	17	CNFN
**MYH11.3**	2.3403	-0.647949406070357	971	325	4.6807850	17	MYH11
**SERPINB1.2**	4.9077	2.97351208002812	982	511	9.8155765	17	SERPINB1
**IL36G**	4.9858	2.0125245167379	861	229	9.9717782	17	IL36G
**IGLV6-57.2**	5.5783	-0.521470870770341	978	478	1.1156714	17	IGLV6-57
**RHCG**	9.5524	3.32700643456821	898	185	1.9104836	17	RHCG

**PRSS22**	1.6837	2.15085012270336	858	184	3.3674358	17	PRSS22
**CSTA.2**	1.5946	5.26233891134955	923	274	3.1892093	17	CSTA
**TMPRSS11A.**	1.8959	1.13004497726785	0.88	158	3.7919855	17	TMPRSS11A
**LMTK3**	2.4973	0.717158409300592	905	257	4.9947517	17	LMTK3
**SDCBP2**	4.2852	1.31225674671345	894	214	8.5705497	17	SDCBP2
**PPL.2**	4.6398	1.55865380177531	938	0.28	9.2797339	17	PPL
**DHRS9**	6.3087	0.491920249848866	861	182	1.2617541	17	DHRS9
**LCN2**	1.8567	2.34638293838567	0.88	192	3.7134241	17	LCN2
**PERP.4**	2.6060	2.54543294934679	949	467	5.2121162	17	PERP
**CALML3.2**	2.1199	3.03270814832451	909	217	4.2398876	17	CALML3
**IL1B.4**	4.0501	0.929525629712831	938	382	8.1002547	17	IL1B
**IGKV1-5.3**	5.4622	-0.307135522483675	971	365	1.0924538	17	IGKV1-5
**FMO2.1**	9.2219	1.34380664317032	905	278	1.8443974	17	FMO2
**CD24.2**	1.6919	2.82758505861479	927	342	3.3838561	17	CD24
**ITPKC.2**	2.7136	0.75917734019395	982	512	5.4272535	17	ITPKC
**RHOD.1**	3.3986	1.32513426966772	0.92	323	6.7973258	17	RHOD
**HMGA1.3**	5.9690	1.12750026758733	982	464	1.1938006	17	HMGA1
**SEMA5A.4**	1.8126	-0.296379607684367	29	346	3.6253606	17	SEMA5A
**PHLDA2.6**	6.2880	1.37881025041445	993	0.53	1.2576004	17	PHLDA2
**THBS1.3**	1.1931	-0.949839939716652	117	602	2.3863380	17	THBS1
**HSPB1.7**	2.9577	2.19125717350593	967	612	5.9154350	17	HSPB1
**CDH1.1**	8.0117	0.403280109043022	872	232	1.6023509	17	CDH1
**SULT2B1**	5.2791	1.43586588737173	872	223	1.0558313	17	SULT2B1
**RBP1.2**	6.2047	0.805063849700789	912	374	1.2409510	17	RBP1
**RORB.8**	1.2830	-0.559470701334968	22	455	2.5661586	17	RORB
**SPRR2B**	2.3318	1.44411150823548	814	0.09	4.6636859	17	SPRR2B
**LYNX1**	5.1209	2.92014576965344	891	355	1.0241818	17	LYNX1
**SPINK7.1**	7.7712	4.75649782385559	0.81	98	1.5542599	17	SPINK7
**MAL.2**	8.9792	3.97628644012419	909	0.31	1.7958571	17	MAL
**EHF.1**	2.2011	1.63478819930599	872	137	4.4022370	17	EHF
**S100A2.4**	1.1242	4.28807495444147	0.92	0.39	2.2484893	17	S100A2
**PITX1.2**	1.4404	2.44854324535122	898	233	2.8808786	17	PITX1

**SMOC2.4**	2.0408	-0.364352654825226	11	353	4.0816508	17	SMOC2
**MARCH1.3**	3.4040	-0.265488608275633	84	358	6.8081539	17	MARCH1
**ITGBL1.6**	6.4247	-0.631362031790337	55	358	1.2849547	17	ITGBL1
**SLPI**	1.4147	3.55322843534241	883	244	2.8294498	17	SLPI
**CP.7**	1.9187	-0.343270730877141	18	461	3.8374256	17	CP
**HES2.2**	4.5651	0.439316716399359	865	253	9.1303725	17	HES2
**PLAC9.4**	2.2612	-0.721478009515106	47	506	4.5225585	17	PLAC9
**RTKN2**	3.5581	0.290583152661816	876	0.3	7.1162791	17	RTKN2
**FGFR1.4**	5.3114	-1.02506709736921	99	599	1.0622807	17	FGFR1
**RHOB.6**	3.0741	-1.32102520873858	131	634	6.1482534	17	RHOB
**S100A16.8**	5.7629	2.33388919262627	942	506	1.1525810	17	S100A16
**KRT24**	6.3502	1.19351811464379	788	82	1.2700444	17	KRT24
**C12orf75.4**	1.2806	0.627247203978348	985	389	2.5613402	17	C12orf75
**PREX2.4**	1.5104	-0.540008004759396	0.04	434	3.0208830	17	PREX2
**CCDC80.4**	1.8210	-1.22800547139338	0.04	484	3.6421776	17	CCDC80
**IGLV1-51.3**	2.1831	-0.443821958713501	956	267	4.3662742	17	IGLV1-51
**GLTP.1**	6.1805	2.524761891547	945	435	1.2361064	17	GLTP
**MMP2.4**	1.5485	-1.7268778863613	128	642	3.0971456	17	MMP2
**AIF1L.1**	5.1621	0.754297767006471	898	293	1.0324268	17	AIF1L
**COL5A2.2**	3.1122	-1.75230294611834	106	611	6.2244990	17	COL5A2
**CXADR.1**	3.4555	0.590859417991143	0.85	166	6.9110051	17	CXADR
**ENC1.2**	3.5240	-0.324836750311081	106	519	7.0480706	17	ENC1
**CYP24A1.1**	3.7501	0.281871834467423	781	118	7.5003772	17	CYP24A1
**GPR157**	1.1348	0.382228128853174	905	446	2.2697451	17	GPR157
**KRT16.2**	1.7662	3.89790706126513	883	334	3.5324622	17	KRT16
**SOD3.7**	3.1736	-1.5548525510916	88	537	6.3472184	17	SOD3
**LRRC15.8**	3.4432	-0.330164885079508	0.12	491	6.8864530	17	LRRC15
**EGR3.1**	8.8933	-0.71724664536085	208	672	1.7786694	17	EGR3
**ROBO1.5**	1.2281	-0.482974924939863	117	533	2.4563721	17	ROBO1
**EGR2.1**	1.3123	-0.32759754952828	164	583	2.6247337	17	EGR2
**MRC2.6**	1.5431	-0.585692042458789	99	443	3.0862266	17	MRC2
**DHCR24.1**	5.3545	0.691718476738296	887	393	1.0709137	17	DHCR24

**COL16A1.4**	4.1557	-0.356538993142829	77	447	8.3115677	17	COL16A1
**DMKN.2**	4.7972	1.28285133292213	847	236	9.5945974	17	DMKN
**APOBEC3A.2**	1.0222	0.977840321755937	836	365	2.0445349	17	APOBEC3A
**FYB.4**	2.4431	-1.35412400119654	131	592	4.8862697	17	FYB
**UPP1.2**	3.1457	2.14026822129082	982	542	6.2914554	17	UPP1
**TACSTD2.3**	9.7657	2.29634764805156	0.88	296	1.9531580	17	TACSTD2
**SPRR2E.2**	2.0128	5.46733727035893	847	237	4.0256008	17	SPRR2E
**NCCRP1**	3.5718	2.0940275267049	777	0.1	7.1436375	17	NCCRP1
**PPIF.2**	6.8009	0.805106337795528	971	532	1.3601938	17	PPIF
**RND3.5**	1.6596	1.29629033495182	982	554	3.3192137	17	RND3
**KLK11.2**	2.7171	1.48279314366374	814	119	5.4342161	17	KLK11
**HS3ST1**	4.2603	0.596994396484943	883	326	8.5206360	17	HS3ST1
**FAM3B**	1.3747	0.611265883936379	796	167	2.7494143	17	FAM3B
**DUSP14.1**	1.3853	1.17467599947565	931	508	2.7706467	17	DUSP14
**LCE3E**	2.2609	1.52650962744575	427	18	4.5218703	17	LCE3E
**FBLN5.6**	4.5713	-0.471630399133481	15	426	9.1426889	17	FBLN5
**GAS6.2**	6.1509	-0.825557985545695	113	558	1.2301860	17	GAS6
**EFEMP2.4**	3.2319	-0.429598099473216	128	0.49	6.4638068	17	EFEMP2
**NID1.2**	3.9251	-0.733101199731927	182	652	7.8503257	17	NID1
**CYGB.5**	5.0966	-0.317210983987342	11	0.46	1.0193397	17	CYGB
**SPON2.6**	6.1540	-0.369395853930481	139	437	1.2308112	17	SPON2
**VNN1.1**	6.4710	0.492692290591266	821	167	1.2942110	17	VNN 1
**LAMA3.2**	1.2304	0.751384818894292	901	311	2.4608114	17	LAMA3
**GUCY1A3.2**	1.5329	-0.28749365894111	58	0.44	3.0658485	17	GUCY1A3
**C19orf33.2**	2.5278	0.966169969217714	861	294	5.0556269	17	C19orf33
**GZMA.4**	1.6969	-1.07621780234504	69	541	3.3938186	17	GZMA
**SGIP1.7**	2.6041	-0.373100343962503	15	508	5.2083701	17	SGIP1
**PRSS8**	4.3648	0.565113817854692	759	65	8.7296384	17	PRSS8
**ABI3BP.5**	5.4573	-0.375807080499561	18	391	1.0914709	17	ABI3BP
**CLTB.3**	7.0946	1.78840684549508	0.92	556	1.4189317	17	CLTB
**FAM110C.1**	1.4555	0.36695283007033	796	184	2.9111216	17	FAM110C
**JAML.6**	2.2552	-0.336431261275384	971	436	4.5105929	17	JAML

**C15orf48.2**	6.9151	3.74817321222711	839	272	1.3830219	17	C15orf48
**WISP2.6**	1.0621	-0.286290254798868	18	363	2.1243050	17	WISP2
**HBEGF.1**	1.0896	0.362611355048971	985	634	2.1793420	17	HBEGF
**HMCN1.5**	2.3343	-0.310787540074461	0.12	477	4.6687884	17	HMCN1
**HIST1H1D**	5.6894	-0.346281114483079	95	382	1.1378961	17	HIST1H1D
**SLURP1**	1.0264	1.36825655885929	741	88	2.0529456	17	SLURP1
**ANK2.4**	5.8288	-0.258650584164356	18	316	1.1657769	17	ANK2
**IL1RN.2**	1.1813	3.06379702951048	825	208	2.3627535	17	IL1RN
**MYL9.6**	1.5759	-1.62786756932899	172	581	3.1518074	17	MYL9
**GPRC5A**	2.3649	0.916943683071278	909	512	4.7298491	17	GPRC5A
**CD8A.5**	2.3713	-0.523727894556903	978	572	4.7427290	17	CD8A
**LTBP4.3**	6.7785	-0.568791615777485	139	446	1.3557185	17	LTBP4
**CD3E.4**	1.1270	-1.02646952325404	135	535	2.2541166	17	CD3E
**LYPD3.2**	3.2270	1.74025102008932	858	304	6.4540606	17	LYPD3
**PRSS12.4**	7.7730	-0.623896888733503	135	475	1.5546077	17	PRSS12
**ID3.2**	1.0051	-1.70985454320159	0.23	676	2.0102692	17	ID3
**XDH**	2.1060	0.340338133432295	704	54	4.2121095	17	XDH
**SPRR2F**	3.3123	2.38284049741006	792	187	6.6246708	17	SPRR2F
**FERMT1.1**	3.5510	0.566087822246295	781	173	7.1020246	17	FERMT1
**ANGPTL4**	1.9272	0.537361935051644	901	455	3.8544323	17	ANGPTL4
**EPHA2.3**	2.4067	0.767018289876826	0.88	418	4.8134199	17	EPHA2
**CCDC3.6**	3.8267	-0.582047163915605	139	472	7.6534689	17	CCDC3
**PRSS27**	9.9729	1.57351133841706	799	205	1.9945812	17	PRSS27
**VCAN.3**	2.8465	-2.32548241733955	369	663	5.6930679	17	VCAN
**ISLR.8**	5.7588	-0.415592635295989	84	383	1.1517666	17	ISLR
**IGLV3-21.6**	7.3987	-0.545003290479996	949	338	1.4797599	17	IGLV3-21
**ANTXR1.5**	8.2375	-0.596262355875683	135	529	1.6475121	17	ANTXR1
**SERINC2.2**	2.8667	1.06806813689005	825	278	5.7335088	17	SERINC2
**IGKV1-12.3**	1.0796	-0.46612911395849	953	379	2.1593852	17	IGKV1-12
**NEGR1.6**	1.4498	-0.257412157555996	69	331	2.8997671	17	NEGR1
**TUBA1C.2**	1.6329	1.37504432483104	974	645	3.2658269	17	TUBA1C
**NET1.3**	3.6525	0.48450768474501	938	429	7.3050024	17	NET1

**IRF1.1**	3.9969	-1.78429610710935	445	793	7.99392912	17	IRF1
**RPTN**	4.1514	0.314604685981984	318	7	8.30282748	17	RPTN
**TGM1**	6.8566	1.83716287232112	781	153	1.37133544	17	TGM1
**MAL2**	7.1070	2.01377182481378	799	181	1.42140430	17	MAL2
**JCHAIN.1**	7.8304	-1.16157233132099	949	506	1.56608877	17	JCHAIN
**ALPL.6**	1.4114	-0.427186495921011	106	512	2.82294179	17	ALPL
**FMO3.3**	1.4952	-0.258066255673248	931	311	2.99055161	17	FMO3
**PDZK1IP1.1**	1.8293	1.58913929126497	799	188	3.65877202	17	PDZK1IP1
**GDF15**	3.2488	0.302655838914627	883	329	6.49766314	17	GDF15
**RHOBTB3.5**	7.2258	-0.402959276678097	55	0.42	1.44516918	17	RHOBTB3
**GZMK.2**	9.0121	-0.790581424736658	974	584	1.80243462	17	GZMK
**CTSG.3**	1.1771	-0.49456326591193	967	467	2.35436655	17	CTSG
**HOPX.2**	1.6102	3.79395343885664	847	324	3.22059250	17	HOPX
**TNFRSF18.1**	1.7786	-0.294003370994999	978	508	3.55734821	17	TNFRSF18
**TRIM16**	2.5625	0.65971894103	869	392	5.12505391	17	TRIM16
**PCDH18.4**	1.0243	-0.272072642630361	113	397	2.04862337	17	PCDH18
**PPP1R14C.1**	3.3492	0.44243139746082	0.77	157	6.69847698	17	PPP1R14C
**FCER1G.5**	6.3643	-0.742223572786471	66	413	1.27286104	17	FCER1G
**PLAT.3**	1.3181	1.43965894638482	894	447	2.63626421	17	PLAT
**FGFR3.1**	1.4918	0.521663760799708	814	296	2.98360553	17	FGFR3
**IL33.8**	2.3864	-0.355549168015702	971	409	4.77294660	17	IL33
**MXRA8.4**	2.5103	-0.673386515919733	99	477	5.02079610	17	MXRA8
**HLA-DRB1.7**	3.9104	-2.07854545807953	277	679	7.82090393	17	HLA-DRB1
**RALGPS2.5**	4.1100	0.436737623992232	934	433	8.22016623	17	RALGPS2
**CEACAM1**	4.4674	1.00376558243992	781	207	8.93487925	17	CEACAM1
**CCL3.1**	7.1822	-0.437753326981515	117	484	1.43645711	17	CCL3
**MACC1**	1.0095	0.33408110229657	799	388	2.01918915	17	MACC1
**FADS1.4**	2.5990	-0.345652138743464	0.04	503	5.19812948	17	FADS1
**ALDH1A3.2**	4.7550	0.98712383429617	894	318	9.51002518	17	ALDH1A3
**LAMC2.2**	5.6419	1.16429933213464	909	359	1.12838522	17	LAMC2
**ST14.3**	6.0968	0.673500375668678	796	224	1.21937462	17	ST14
**RAB11FIP1.5**	9.7453	0.682841841585254	964	577	1.94907021	17	RAB11FIP1

**CCR7.1**	1.5428	-0.284898055400144	978	629	3.0856848	17	CCR7
**KLF5.2**	1.7104	0.918092019043243	836	396	3.4209853	17	KLF5
**MEOX1.5**	3.1501	-0.379862958447832	51	381	6.3003729	17	MEOX1
**MPZL2.10**	3.3094	0.708216537467871	909	429	6.6188857	17	MPZL2
**TBX2.4**	3.7942	-0.284606981479481	971	437	7.5885009	17	TBX2
**ATP1B1.3**	7.0579	1.15070761628512	869	482	1.4115952	17	ATP1B1
**MOXD1.5**	1.4102	-0.291483628109929	964	0.39	2.8204327	17	MOXD1
**COL3A1**	2.2862	-4.22458393202461	489	0.78	4.5724557	17	COL3A1
**NOSTRIN.7**	2.3679	-0.316427797487709	974	471	4.7359105	17	NOSTRIN
**COL11A1.7**	7.2548	-0.560845593770263	139	415	1.4509779	17	COL11A1
**SPINT2.3**	8.0196	1.56138703552063	843	321	1.6039228	17	SPINT2
**CXCL8.1**	1.0261	0.494291941192067	945	588	2.0523434	17	CXCL8
**NREP.2**	1.5314	-0.370634870115923	0.15	499	3.0628589	17	NREP
**ZNF385D.5**	2.0658	-0.749710111725918	117	507	4.1317818	17	ZNF385D
**ITGA2.3**	2.6501	0.493544740175122	883	374	5.3002514	17	ITGA2
**TUBA4A.1**	3.8399	0.477434826433329	982	532	7.6799693	17	TUBA4A
**RBM47.1**	4.3733	0.652451393957944	839	459	8.7466257	17	RBM47
**SGCB.6**	4.9571	-0.52237739798166	47	439	9.9143809	17	SGCB
**IGFBP6.4**	8.6406	0.288065757492701	0.92	443	1.7281257	17	IGFBP6
**CLDN1**	1.3831	0.901456506470303	766	0.17	2.7662010	17	CLDN1
**C1S.2**	2.6766	-1.96792982548914	263	636	5.3533405	17	C1S
**DNAAF1.1**	1.0872	-0.265443996192223	974	541	2.1744387	17	DNAAF1
**TEK.8**	1.5579	-0.266474569009992	978	437	3.1158853	17	TEK
**LAD1.1**	1.9125	1.18230023977907	785	253	3.8250897	17	LAD1
**HLA-DPB1.5**	2.6594	-2.20847263231716	248	609	5.3188329	17	HLA-DPB1
**THY1.5**	4.0935	-1.4045505159056	139	0.5	8.1870674	17	THY1
**MXD1.2**	4.6046	1.70892407285287	858	521	9.2092227	17	MXD1
**PLXDC1.5**	1.1560	-0.370452552994727	58	457	2.3121952	17	PLXDC1
**APLNR.5**	1.8266	-0.417041310080622	971	466	3.6532876	17	APLNR
**TMEM45A.2**	2.7403	0.469983993918918	0.85	318	5.4807348	17	TMEM45A
**CDS1.1**	8.1606	0.273784567794403	763	196	1.6321288	17	CDS1
**GGT5.4**	1.4319	-0.892966380089044	223	594	2.8639739	17	GGT5

**LRP1.4**	1.5578	-0.753466355716836	237	555	3.1157905	17	LRP1
**SPRY1.5**	3.0675	-1.29168780952925	255	0.6	6.1351936	17	SPRY1
**GJB2.1**	1.0275	1.4648924706359	777	255	2.0551063	17	GJB2
**SPOCK2.3**	2.3397	-0.4024171640355	964	531	4.6794434	17	SPOCK2
**APCDD1.1**	7.0422	-1.57052064350002	153	507	1.4084531	17	APCDD1
**ECM1.5**	1.1949	2.02993956548992	865	429	2.3899973	17	ECM1
**GLUL.4**	1.2519	2.47891879968497	974	604	2.5039214	17	GLUL
**ENPEP.6**	1.5855	-0.301147857951566	18	435	3.1711352	17	ENPEP
**SERPINB13**	8.3789	0.939083892146428	752	261	1.6757821	17	SERPINB13
**HN1.2**	4.5669	0.973466123696382	927	511	9.1339424	17	HN1
**FXYD3.3**	5.2182	1.98531408602299	788	238	1.0436423	17	FXYD3
**MAP1B.7**	1.0920	-1.11671803575552	172	532	2.1840879	17	MAP1B
**PLAUR.3**	1.9334	0.473178915158446	934	599	3.8669480	17	PLAUR
**CD7.2**	1.1864	-0.576409433282849	0.96	543	2.3729116	17	CD7
**NOTCH4.2**	1.3175	-0.304243449575511	971	395	2.6351420	17	NOTCH4
**LIMS2.2**	4.0651	-0.473498863421261	285	541	8.1303359	17	LIMS2
**CST7.1**	2.1416	-0.803917807346347	974	611	4.2832514	17	CST7
**CARHSP1.5**	5.5232	2.11652393836434	883	528	1.1046528	17	CARHSP1
**SPNS2.5**	8.8309	0.942760756969369	942	482	1.7661930	17	SPNS2
**GAS1.5**	3.2055	-0.863635819244762	226	509	6.4111679	17	GAS1
**TRBC2.2**	3.6259	-1.44875504825197	285	576	7.2518056	17	TRBC2
**ASPG**	5.4866	0.508730470661616	697	98	1.0973212	17	ASPG
**SFN.3**	1.2933	3.1133652771845	781	295	2.5866660	17	SFN
**GREM1.6**	2.1164	-0.295029196321718	102	455	4.2328464	17	GREM1
**HSPA1A.3**	1.3856	-1.24275561605746	339	673	2.7712708	17	HSPA1A
**ALDH3A1.2**	1.8578	1.36945250121549	763	241	3.7157634	17	ALDH3A1
**ID2.1**	2.2081	-0.992276850650452	281	0.59	4.4163014	17	ID2
**SCNN1B**	2.6262	0.40167108399399	766	265	5.2524098	17	SCNN1B
**RIPK4**	3.1689	0.274263892912037	708	0.15	6.3378964	17	RIPK4
**MALL.4**	3.4447	1.15749699527903	861	572	6.8894973	17	MALL
**ICOS.3**	1.3463	-0.448084951597774	967	526	2.6927301	17	ICOS
**FABP5.3**	1.9311	3.38030634564019	803	494	3.8622422	17	FABP5

**SVIL.4**	2.2705	-0.734959326019705	274	568	4.5410824		17	SVIL
**KLK13**	6.7290	0.78891061357265	693	127	1.3458112		17	KLK13
**PVRL4**	2.6101	0.769224315377627	715	0.16	5.2203583		17	PVRL4
**KDR.6**	1.0020	-0.466435099828841	967	0.45	2.0041757		17	KDR
**GCHFR**	1.4149	0.871173065879932	774	322	2.8298679		17	GCHFR
**KRT4.1**	2.1879	2.234043422595	0.73	202	4.3758529		17	KRT4
**FBLN2.7**	4.0634	-1.43488886949415	153	535	8.1269843		17	FBLN2
**RGS5.4**	5.4027	-0.673545934111604	869	384	1.0805555		17	RGS5
**APOC1.3**	6.1324	-0.361159519579414	128	426	1.2264949		17	APOC1
**VAMP8.5**	9.8451	1.12864677284279	825	401	1.9690250		17	VAMP8
**TMPRSS11B**	1.6995	1.67148172703809	686	85	3.3991671		17	TMPRSS11B
**IGF2.6**	2.2678	-1.08053631426667	252	557	4.5357220		17	IGF2
**HPGD.4**	1.0211	0.476554564034882	781	312	2.0422833		17	HPGD
**BGN.6**	1.1556	-1.05539248251756	328	619	2.3113801		17	BGN
**TGFBI.7**	1.6121	-0.404665444806182	0.23	487	3.2242902		17	TGFBI
**GPR183.3**	1.8900	-1.24895729769755	299	549	3.7800685		17	GPR183
**RAB25.1**	1.9606	1.63259779220529	0.73	161	3.9212800		17	RAB25
**TUSC3.1**	1.4337	0.35947458673486	0.81	0.27	2.8675666		17	TUSC3
**SOX17.4**	2.6910	-0.287793730634173	15	0.33	5.3820989		17	SOX17
**AQP3.2**	9.8617	2.09435892980472	759	405	1.9723438		17	AQP3
**CXorf36.7**	2.9771	-0.423164087235875	967	452	5.9542616		17	CXorf36
**RP11-350J20**	1.0067	0.536304069210273	653	115	2.0134139		17	RP11-350J20
**SDC1.6**	3.9016	0.849031238481097	796	457	7.8033687		17	SDC1
**PKP4.7**	2.4008	-0.287867439745952	978	561	4.8016567		17	PKP4
**KRT5.3**	3.4549	3.02296253580424	766	408	6.9098168		17	KRT5
**ITGB8.1**	6.8718	0.286044061140774	755	255	1.3743680		17	ITGB8
**IGKV4-1.4**	7.4705	-0.354706762820926	872	0.38	1.4941147		17	IGKV4-1
**MASP1.8**	8.3248	-0.388201188081187	938	429	1.6649740		17	MASP1
**MMRN2.8**	1.4057	-0.626957039640655	197	448	2.8114605		17	MMRN2
**XCL1.4**	1.5791	-0.506923015892003	974	542	3.1583813		17	XCL1
**FLT1.7**	1.8449	-0.778373851066017	971	491	3.6898771		17	FLT1
**CRABP2.8**	2.3448	2.13869458916284	781	385	4.6897816		17	CRABP2

**LAMB3.1**	3.2824	1.30445112074268	0.73	284	6.5649892	17	LAMB3
**STC1.3**	4.6205	-0.64386819269709	971	609	9.2411012	17	STC1
**FABP4.1**	5.8431	0.803072789444096	0.73	222	1.1686309	17	FABP4
**LOXL2.2**	7.2035	-0.431745566087474	113	501	1.4407175	17	LOXL2
**EFHD2.4**	8.0372	0.561095649671542	931	567	1.6074425	17	EFHD2
**C4orf26**	3.3674	1.77056487452795	453	168	6.7349036	17	C4orf26
**SLC12A8.1**	3.9850	0.783975113990989	737	292	7.9700425	17	SLC12A8
**C1QA.3**	7.6185	-0.488542246796026	237	527	1.5237074	17	C1QA
**KRT78**	8.5975	2.07212706354143	675	79	1.7195063	17	KRT78
**SLAMF7.3**	1.8634	-0.307802854426552	974	542	3.7269006	17	SLAMF7
**CXCL1.4**	2.9553	0.382326728114992	887	508	5.9106188	17	CXCL1
**KRT6B.1**	8.9196	2.58203151234839	715	248	1.7839364	17	KRT6B
**DSEL.4**	1.0485	-0.460053188912715	212	486	2.0971652	17	DSEL
**CTLA4.5**	1.5177	-0.360711537147629	916	458	3.0354755	17	CTLA4
**CTSD.4**	2.7288	0.708646993768403	942	535	5.4576607	17	CTSD
**TMEM45B**	4.3111	0.538928400545218	682	135	8.6222835	17	TMEM45B
**ACTA2.3**	6.2170	-1.41595465289272	843	376	1.2434060	17	ACTA2
**RAB31.5**	9.9256	0.307849852419252	953	623	1.9851370	17	RAB31
**FN1.5**	1.0519	-1.16278338698868	321	605	2.1038451	17	FN1
**FBLN1.4**	1.3251	-0.707264083855675	894	611	2.6502753	17	FBLN1
**MUC1**	1.5289	1.02938522445762	788	429	3.0578572	17	MUC1
**ROBO4.8**	1.1514	-0.447441255898872	967	435	2.3029526	17	ROBO4
**ODAM**	1.2735	1.95431930006035	0.42	86	2.5470878	17	ODAM
**CALU.5**	1.2576	-0.949395122490232	0.35	625	2.5152700	17	CALU
**KRT15.3**	1.4098	2.49218595038182	0.69	226	2.8197982	17	KRT15
**MMP12.1**	1.7621	1.37261442906243	631	182	3.5242982	17	MMP12
**ITGA8.4**	1.9322	-0.252931283942399	967	0.43	3.8645658	17	ITGA8
**RASD1.2**	3.4185	-0.636248286636772	0.27	534	6.8371694	17	RASD1
**ADIRF.10**	3.4694	1.63260815308523	996	0.66	6.9388217	17	ADIRF
**ICAM2.5**	4.3664	-0.390476370642942	978	0.53	8.7329740	17	ICAM2
**MCTP1.5**	6.4844	-0.628666880494395	974	553	1.2968800	17	MCTP1
**MYCT1.7**	1.0315	-0.320608454218371	934	473	2.0631460	17	MYCT1

**FZD4.4**	2.1535	-0.335987799085737	44	393	4.30713580	17	FZD4
**RDH10**	6.5350	1.07689895548373	821	532	1.30701042	17	RDH10
**PKP1.1**	7.3959	1.3549576710728	704	218	1.47918055	17	PKP1
**HES1.4**	1.4716	-0.820627979111334	394	664	2.94333334	17	HES1
**VMO1.1**	2.9359	0.992221410116796	788	331	5.87192092	17	VMO1
**CCL21.3**	6.3651	-1.2831168527869	51	461	1.27303710	17	CCL21
**NABP1.2**	1.0950	0.402059164957704	818	474	2.19006571	17	NABP1
**KCNN3.3**	1.5074	-0.333873747593063	956	518	3.01486351	17	KCNN3
**GJB3.1**	3.0892	0.360279688793581	657	0.07	6.17858940	17	GJB3
**CXCL10.1**	3.5747	-0.289800648464648	139	472	7.14947634	17	CXCL10
**CD68.3**	3.6376	0.498555805441721	785	513	7.27520372	17	CD68
**PCDH17.9**	3.7780	-0.79128722506306	971	546	7.55613751	17	PCDH17
**FAM167B.7**	4.0404	-0.317432623582921	953	461	8.08083164	17	FAM167B
**LCP1.6**	4.0464	-0.861897847767717	974	572	8.09281451	17	LCP1
**RPGR.1**	4.8859	-0.312525915981608	898	448	9.77187961	17	RPGR
**FKBP10.2**	6.2699	-0.484930887875329	193	496	1.25399031	17	FKBP10
**MBOAT2**	7.4165	0.694409737735452	704	322	1.48331580	17	MBOAT2
**OLFM1.4**	1.8215	-0.368818631804285	0.12	401	3.64315353	17	OLFM1
**DUSP6.3**	2.3790	-0.482338309445704	974	636	4.75813579	17	DUSP6
**SFRP4.4**	2.7692	-0.413557086998888	15	411	5.53855001	17	SFRP4
**CDH13.4**	3.5579	-0.283119140988146	967	495	7.11580837	17	CDH13
**AREG.3**	6.3518	-0.856530983170076	949	0.64	1.27036532	17	AREG
**CD2.3**	7.5222	-1.01373694878139	964	598	1.50445172	17	CD2
**SPINK5.1**	1.1609	2.78572624124249	686	226	2.32198853	17	SPINK5
**PIM1**	1.8914	0.835495644782742	927	615	3.78282740	17	PIM1
**COL5A1.4**	2.5395	-0.884907138019344	277	528	5.07905251	17	COL5A1
**GZMH.4**	4.3037	-0.341845004576904	0.81	483	8.60745421	17	GZMH
**CTNNBIP1.1**	4.8184	1.40373474588138	693	332	9.63692743	17	CTNNBIP1
**DUSP5.1**	5.9516	0.86197151158584	967	604	1.19033918	17	DUSP5
**CDH5.5**	6.0143	-0.779384951868249	964	606	1.20286193	17	CDH5
**CLEC14A.8**	6.0669	-0.990666434021837	967	526	1.21338111	17	CLEC14A
**LTBP1.5**	7.7722	-0.574821165203893	949	569	1.55445480	17	LTBP1

**LTBP2.5**	1.3386	-0.462296721111928	978	571	2.6773456		17	LTBP2
**OAS1.1**	2.1832	0.342500744169583	712	275	4.3664144		17	OAS1
**TRIM29.1**	6.8186	1.22563760153206	682	217	1.3637275		17	TRIM29
**TMPRSS2**	8.3388	0.882593449187336	361	26	1.6677687		17	TMPRSS2
**KRT8.1**	1.7721	0.577792446333178	682	249	3.5443397		17	KRT8
**AC019349.5**	2.1560	0.337105776637195	639	81	4.3120292		17	AC019349.5
**RGS2.4**	1.0272	-0.765531207526319	982	614	2.0545712		17	RGS2
**FILIP1.6**	1.5661	-0.386063839135617	828	338	3.1323651		17	FILIP1
**CCL20.1**	1.9985	0.95899982796106	0.69	316	3.9970857		17	CCL20
**CXCL17**	8.2026	0.700205871629434	628	62	1.6405305		17	CXCL17
**DIO2.3**	1.6296	-0.871918130604589	854	564	3.2593465		17	DIO2
**IGKV3-15.2**	1.7920	-0.320361091397285	624	262	3.5840401		17	IGKV3-15
**PRDM1**	1.9871	0.301015928024328	964	634	3.9743137		17	PRDM1
**ECSCR.1.7**	8.6371	-1.02152293407501	971	0.57	1.7274353		17	ECSCR.1
**SELE.4**	2.2247	-2.28543544389254	974	638	4.4494765		17	SELE
**TMEM176A.7**	3.1598	-0.389663448166203	949	504	6.3196449		17	TMEM176A
**SDC2.9**	3.4300	-0.463406271319969	47	372	6.8601073		17	SDC2
**INPP4B.3**	4.6007	-0.283690621455587	847	388	9.2014791		17	INPP4B
**POSTN.3**	6.2249	-1.14152255852162	971	549	1.2449817		17	POSTN
**ENPP2.5**	7.3952	-0.281425007961011	0.96	431	1.4790564		17	ENPP2
**KCTD12.5**	1.4612	-0.762179130946128	978	0.59	2.9225508		17	KCTD12
**MGST1**	2.0416	0.278755025416528	686	331	4.0832052		17	MGST1
**ADAMTS2.8**	3.1477	-0.439978240843744	128	484	6.2954967		17	ADAMTS2
**LGALS3BP.4**	7.1021	-0.326426964318811	836	534	1.4204209		17	LGALS3BP
**CRTAM.4**	1.0817	-0.554361165882954	832	526	2.1635990		17	CRTAM
**TNXB.7**	1.3555	-0.446294180496702	938	527	2.7111416		17	TNXB
**TIGIT.3**	1.6115	-0.580665022331489	912	574	3.2230606		17	TIGIT
**PLVAP.7**	2.0521	-1.30810621740598	967	575	4.1043880		17	PLVAP
**ANGPT1.4**	2.2485	-0.34658182637634	843	475	4.4970508		17	ANGPT1
**ANPEP.7**	7.1622	-0.336658170107208	109	383	1.4324560		17	ANPEP
**DERL3.4**	8.1161	-0.682663663659262	964	0.56	1.6232336		17	DERL3
**THBD.5**	8.7023	-0.425786272690401	0.88	549	1.7404798		17	THBD

**CD8B.2**	1.4708	-0.385759628693574	777	487	2.9417910	17	CD8B
**GNA15.3**	1.6038	0.353856936930033	675	343	3.2076757	17	GNA15
**NES.8**	1.8520	-0.396783460900564	836	361	3.7040360	17	NES
**BATF.3**	3.7657	-0.552053821240159	916	508	7.5315232	17	BATF
**ERO1A**	4.2044	1.54468451108208	715	0.43	8.4088313	17	ERO1A
**CLDN7**	7.2578	1.06408031869438	635	187	1.4515656	17	CLDN7
**BASP1.5**	8.2732	-0.257017660344542	828	475	1.6546448	17	BASP1
**C2.3**	8.7971	-0.319961851446664	971	495	1.7594238	17	C2
**ODC1.1**	4.5310	-0.294186375374821	982	678	9.0621446	17	ODC1
**DNASE1L3.6**	9.6227	2.84505185706163	697	391	1.9245591	17	DNASE1L3
**DSP.3**	1.0816	1.59849591824937	668	337	2.1633123	17	DSP
**KRT80**	1.1215	0.791190000666168	606	58	2.2431818	17	KRT80
**ERG.5**	1.1807	-0.439451624800086	887	436	2.3615821	17	ERG
**TMEM54.1**	1.3829	0.875626057936681	664	0.27	2.7659291	17	TMEM54
**POU2F2.7**	1.4192	-0.560201821519401	909	469	2.8384163	17	POU2F2
**PDGFB.4**	1.5442	-0.261500849214946	931	517	3.0885855	17	PDGFB
**LCE3D**	1.5809	3.35141557452453	398	62	3.1618926	17	LCE3D
**MECOM.7**	2.0968	-0.273520313185924	781	401	4.1936635	17	MECOM
**BCAM.7**	2.4647	-0.772654115352687	923	515	4.9295177	17	BCAM
**ADGRF5.5**	2.7275	-0.635207299388239	934	575	5.4551185	17	ADGRF5
**GPR87.1**	3.3718	0.368686771490478	354	29	6.7437206	17	GPR87
**S100A13.6**	3.9097	0.302073882247578	869	539	7.8195611	17	S100A13
**PRRG4.2**	3.9857	0.743254436139552	639	206	7.9715610	17	PRRG4
**JSRP1.3**	8.9637	-0.274033690517868	934	481	1.7927582	17	JSRP1
**ADM5.5**	1.0704	-0.28285559382425	766	439	2.1408317	17	ADM5
**GBP6.2**	1.3581	1.21980298184399	642	222	2.7163792	17	GBP 6
**TNFRSF21.1**	1.8330	0.427123088426596	741	462	3.6660194	17	TNFRSF21
**OGN.5**	6.1833	-0.301928953686438	0.77	396	1.2366793	17	OGN
**CST6**	1.2415	0.840229143725465	599	114	2.4831930	17	CST6
**DENND2C**	3.3538	0.590878293785572	635	286	6.7077347	17	DENND2C
**PTPRB.8**	5.7539	-0.509710632242749	876	536	1.1507844	17	PTPRB
**AOAH.5**	6.6409	-0.260561301818811	923	418	1.3281895	17	AOAH

**IRF4.3**	6.6577	-0.256130655373033	894	0.45	1.3315531	17	IRF4
**TMEM40**	8.8374	1.80280714221587	613	103	1.7674892	17	TMEM40
**RCAN2.4**	1.4448	-0.357481894460622	18	357	2.8897882	17	RCAN2
**IL20RB.1**	1.9819	0.423604037617077	635	214	3.9639663	17	IL20RB
**VWF.7**	2.2081	-1.59743895813172	898	576	4.4163302	17	VWF
**SELP.6**	3.2314	-0.726943170102583	818	542	6.4629919	17	SELP
**NCOA7.5**	5.3775	-0.549038093876465	978	623	1.0755054	17	NCOA7
**HIST1H2AC**	5.4311	0.867608805063099	675	425	1.0862300	17	HIST1H2AC
**JUP.4**	5.9754	1.34313065412988	0.65	374	1.1950960	17	JUP
**RAMP3.7**	6.8224	-0.91331960371388	891	486	1.3644823	17	RAMP3
**CFH.4**	8.8457	-0.455812594747863	923	587	1.7691548	17	CFH
**FAP.3**	1.2341	-0.302351342017063	0.12	381	2.4683946	17	FAP
**LRG1.1**	2.7376	0.311905143487003	693	301	5.4752785	17	LRG1
**CCL4.2**	4.0896	-2.43370222361009	883	594	8.1792373	17	CCL4
**INHBB.4**	4.3594	-0.304735418898579	923	0.53	8.7189088	17	INHBB
**IGHG3.1**	5.6201	-2.8805590867576	978	0.72	1.1240340	17	IGHG3
**PPFIBP1.6**	7.7715	-0.300705740370776	832	462	1.5543135	17	PPFIBP1
**CD200.8**	8.8919	-0.495924437278431	971	0.52	1.7783991	17	CD200
**INSR.4**	1.2247	-0.696802028032448	938	565	2.4494701	17	INSR
**CPXM1.4**	1.3171	-0.26596964406191	938	507	2.6343403	17	CPXM1
**RAB27B.1**	1.4388	0.415694928675854	0.62	256	2.8776976	17	RAB27B
**CAPG.3**	1.6273	0.416596935360531	682	0.38	3.2547752	17	CAPG
**SLC9A3R1.2**	2.2422	0.68895152739638	675	422	4.4844883	17	SLC9A3R1
**NPDC1.7**	3.8073	-0.876862143385832	905	556	7.6146477	17	NPDC1
**ADAM15.6**	5.5623	-0.505566580888782	858	528	1.1124790	17	ADAM15
**PTP4A3.3**	1.2324	-0.520363259066178	953	475	2.4649945	17	PTP4A3
**IGLV3-1.7**	2.3931	-0.767216140444939	785	369	4.7863829	17	IGLV3-1
**PHACTR1.4**	3.3680	-0.292498648947207	872	408	6.7360614	17	PHACTR1
**S1PR1.8**	3.8169	-0.422419382486023	956	525	7.6339139	17	S1PR1
**FDCSP.2**	4.6417	4.45695696099072	595	298	9.2834787	17	FDCSP
**GZMB.4**	1.8589	-0.408125675915504	752	483	3.7178548	17	GZMB
**COL12A1.2**	2.3116	-1.28908551737793	971	627	4.6232132	17	COL12A1

**RNASE1.6**	3.1464	-1.09632778125612	945	572	6.2929057	17	RNASE1
**TP63.1**	3.4513	0.284787725207769	609	225	6.9026420	17	TP63
**AQP1.7**	9.0315	-2.2372245681084	974	682	1.8063040	17	AQP1
**LY6D.3**	1.0170	2.11707411965298	0.62	268	2.0341389	17	LY6D
**CDCP1.1**	1.1773	0.395482255866726	599	148	2.3547873	17	CDCP1
**FUT3**	1.3004	0.377690039620201	292	9	2.6008400	17	FUT3
**AKR1C1.3**	1.5591	0.560548327773366	609	246	3.1182055	17	AKR1C1
**PSCA**	2.2088	0.972958742234458	343	48	4.4176857	17	PSCA
**IL7R.2**	3.1613	-1.56840236336119	898	606	6.3227439	17	IL7R
**CRB3**	6.1447	0.512872075007455	584	159	1.2289421	17	CRB3
**LMCD1.2**	8.6504	-0.63895243932342	971	596	1.7300993	17	LMCD1
**COL5A3.7**	1.2751	-0.2814763304326	803	456	2.5502094	17	COL5A3
**BCL2A1.7**	2.4681	-0.366890954782703	708	426	4.9362918	17	BCL2A1
**SAA1.2**	7.0644	0.660466844695531	573	263	1.4128955	17	SAA1
**ADAMTS4.6**	1.1184	-0.436969373179971	942	547	2.2368736	17	ADAMTS4
**HMOX1.3**	1.1915	1.09225574283731	723	455	2.3830191	17	HMOX1
**NUAK1.6**	1.2794	-0.587273092358139	883	0.46	2.5588914	17	NUAK1
**NDRG2.1**	2.3992	0.502993444090355	602	324	4.7984004	17	NDRG2
**EGFL7.10**	2.3996	-0.583242313526689	748	426	4.7993952	17	EGFL7
**CYYR1.4**	2.4208	-0.50801742853366	763	414	4.8417665	17	CYYR1
**CLDN5.9**	2.8539	-1.35070463316249	745	441	5.7079517	17	CLDN5
**MZB1.4**	6.1515	-1.62449385881552	0.96	618	0.0001230	17	MZB1
**KLK10**	7.3832	0.960138942097926	569	135	0.0001476	17	KLK10
**TM4SF18.6**	7.8383	-0.468392196071277	0.73	479	0.0001567	17	TM4SF18
**TNFSF13B.6**	1.3168	-0.261360407959478	883	444	0.0002633	17	TNFSF13B
**ZNF331.3**	1.4718	-1.00830719394222	982	689	0.0002943	17	ZNF331
**GRB10.5**	1.6932	-0.273505811166736	916	493	0.0003386	17	GRB10
**TINAGL1.6**	1.8976	-0.538460947462208	828	562	0.0003795	17	TINAGL1
**ITGA6.9**	2.5429	-0.602131795000471	938	617	0.0005085	17	ITGA6
**TWIST1.3**	2.6120	-1.05608664017808	763	451	0.0005224	17	TWIST1
**PMEPA1.1**	3.6563	-0.501278819801177	894	623	0.0007312	17	PMEPA1
**GJA4.3**	4.1373	-0.343744480430702	62	318	0.0008274	17	GJA4

**CCL18**	5.8381	-0.624843695918556	931	631	0.0011676	17	CCL18
**YOD1**	6.3100	0.307021371207646	635	375	0.0012620	17	YOD1
**AKR1B10.2**	6.5950	1.2540352439637	584	255	0.0013190	17	AKR1B10
**PECAM1.8**	1.8000	-1.71241877199769	974	0.67	0.0036001	17	PECAM1
**IMPA2.1**	3.0266	0.784830486256588	566	245	0.0060533	17	IMPA2
**DSC3.1**	4.4543	0.786847069995643	562	186	0.0089086	17	DSC3
**IGHA1**	4.8522	-3.20746222503869	978	694	0.0097045	17	IGHA1
**TFRC.1**	8.9480	0.701279233470756	887	601	0.0178960	17	TFRC
**KRT6C.2**	2.1872	2.7956998346131	569	236	0.0437446	17	KRT6C
**COL15A1.4**	2.8065	-1.69436968199205	967	662	0.0561312	17	COL15A1
**SERPINE1**	6.4140	-0.749963020869823	974	724	0.1282811	17	SERPINE1
**CD83.3**	7.4508	-1.02378529523957	916	601	0.1490168	17	CD83
**SELL.2**	9.4131	-0.492866980589244	836	544	0.1882623	17	SELL
**RARRES2.3**	0.0001	-1.8188160884277	0.96	644	0.2262036	17	RARRES2
**SLIT3.10**	0.0001	-0.548211712065391	766	425	0.2388613	17	SLIT3
**SERPINH1.3**	0.0001	-0.998358360167244	974	708	0.3224620	17	SERPINH1
**F2R.3**	0.0002	-0.728712143952211	883	586	0.5161131	17	F2R
**SERPINB9.3**	0.0006	-0.446139360342462	898	0.61	1	17	SERPINB9
**EVPL**	0.0009	0.411618020389626	431	0.08	1	17	EVPL
**STAB1.5**	0.0009	-0.330465482810973	832	547	1	17	STAB1
**TGM2.3**	0.0010	-0.555232264406082	869	0.61	1	17	TGM2
**NDUFA4L2.4**	0.0013	0.358302664638712	566	233	1	17	NDUFA4L2
**ASPN.7**	0.0013	-0.784333471238105	752	474	1	17	ASPN
**TNS3.3**	0.0022	-0.317912290951939	854	497	1	17	TNS3
**IGLC3.1**	0.0024	-3.80247622329386	974	671	1	17	IGLC3
**EPSTI1.5**	0.0025	-0.256216337980333	715	428	1	17	EPSTI1
**TPM1.8**	0.0029	-1.27503845790601	799	502	1	17	TPM1
**MUC21**	0.0043	1.18711558431065	394	77	1	17	MUC21
**NMU.1**	0.0059	0.638485298444239	438	81	1	17	NMU
**ADAMTS1.4**	0.0087	-1.54886875374726	978	682	1	17	ADAMTS1
**FGL2.4**	0.0097	-0.503229989806409	891	467	1	17	FGL2
**HP**	0	2.14513363435218	0.5	14	0	18	HP

**STAB2**	3.6781	0.794482773557286	0.57	0.05	7.3562230	18	STAB2
**GPR182**	5.4409	0.470000916542193	0.5	67	1.0881821	18	GPR182
**TFPI.2**	1.7097	3.01356091995221	917	607	3.4194598	18	TFPI
**FN1.6**	2.7215	2.15693756776773	946	598	5.4430520	18	FN1
**PDPN.2**	1.4514	2.32103755133505	843	423	2.9028079	18	PDPN
**GNG11.6**	4.6251	2.0233432644831	959	579	9.2503843	18	GNG11
**IGFBP7.9**	1.0820	2.03752775978946	1	0.73	2.1641257	18	IGFBP7
**CLDN5.10**	1.7327	1.83740688028867	917	439	3.4655390	18	CLDN5
**ACKR3.4**	2.4557	2.03147982002392	876	609	4.9115610	18	ACKR3
**ACKR2**	1.1769	0.864887702224913	0.64	213	2.3539226	18	ACKR2
**NR2F1**	3.9523	1.37921922690608	752	251	7.9047145	18	NR2F1
**ECSCR.1.8**	2.1573	1.68272810247477	921	571	4.3146778	18	ECSCR.1
**RAMP2.8**	3.4560	1.51627076186565	938	0.48	6.9121003	18	RAMP2
**LAMA4.4**	3.6698	1.4141152904612	0.93	567	7.3396442	18	LAMA4
**EFEMP1.2**	1.5008	2.11512083781649	864	576	3.0017498	18	EFEMP1
**TIMP3.5**	5.3396	1.47629813370531	942	659	1.0679321	18	TIMP3
**UNC5B.5**	3.8160	1.5971077349323	822	439	7.6320335	18	UNC5B
**NID1.3**	2.2833	1.19325552128376	913	643	4.5666141	18	NID1
**PPFIBP1.7**	5.0473	2.2518433427271	847	462	1.0094661	18	PPFIBP1
**AKAP12.7**	1.8675	2.09141605146653	868	584	3.7350819	18	AKAP12
**NFIB.4**	5.3939	1.24392643807873	955	596	1.0787969	18	NFIB
**SEMA3D**	1.1061	0.883730225269895	636	194	2.2123263	18	SEMA3D
**ARHGAP29.6**	1.1570	1.41023811277951	901	527	2.3140547	18	ARHGAP29
**CCDC80.5**	9.6793	1.25343657522236	901	473	1.9358642	18	CCDC80
**LYVE1**	2.3117	1.46183891046858	744	373	4.6235582	18	LYVE1
**CXCL2.1**	4.1099	1.64933264860566	909	655	8.2198679	18	CXCL2
**CCR7.2**	6.2589	-0.769625655374597	178	639	1.2517867	18	CCR7
**TM4SF1.7**	9.0523	1.44041980475492	938	616	1.8104734	18	TM4SF1
**TFF3.1**	2.1267	3.7925733491748	702	247	4.2535204	18	TFF3
**LOX.6**	2.6855	1.09808624466553	777	368	5.3710302	18	LOX
**CLU.4**	1.7514	1.41462185595192	926	615	3.5028915	18	CLU
**CCL2.3**	2.3111	1.60386050321292	917	639	4.6223487	18	CCL2

**PGM5**	3.891(	1.64948858314158	0.76	341	7.7821136!	18	PGM5
**PROX1.1**	3.489:	2.33173318501987	682	193	6.9787722	18	PROX1
**NR2F2.6**	1.494	1.51474748410369	897	532	2.9888236	18	NR2F2
**EGFL7.11**	2.020(	1.58435410118674	822	426	4.0401504!	18	EGFL7
**LIF.1**	1.122	0.450936922427689	669	285	2.2456847	18	LIF
**CNKSR3.5**	2.847:	1.53326259876526	806	489	5.6947396!	18	CNKSR3
**CCL21.4**	7.520	7.21096160594527	748	453	1.5040430!	18	CCL21
**HYAL2.6**	1.687	1.37989588624105	876	498	3.3756994	18	HYAL2
**SLC39A14.3**	3.708(	0.561256998042338	847	563	7.4160397	18	SLC39A14
**MMRN1.5**	5.931	2.93297654433876	731	403	1.1862514	18	MMRN1
**CEMIP.1**	1.201	0.749986602786224	694	341	2.4036671	18	CEMIP
**GJA1.5**	2.735	1.19276039930698	835	0.47	5.4712993	18	GJA1
**RGS3.4**	4.117:	1.23157090681044	847	584	8.2346414	18	RGS3
**RAI14.5**	8.480	1.03975909473986	893	619	1.6960842	18	RAI14
**SDPR.3**	5.4771	2.38833934924643	0.76	442	1.0954376	18	SDPR
**PDLIM1.7**	7.130(	1.30690908806416	942	632	1.4260018	18	PDLIM1
**NRP2.3**	7.309	1.91777658011851	793	516	1.4619911	18	NRP2
**CALCRL.5**	1.288	1.21561706028646	814	463	2.5770777	18	CALCRL
**ENG.7**	1.295	0.6594361515318	0.95	599	2.5914620!	18	ENG
**EDNRB.5**	1.409	0.810831251610435	748	423	2.8197590	18	EDNRB
**HSPB1.8**	9.290	0.994227671052284	955	613	1.8581588!	18	HSPB1
**SEMA3A**	1.186	1.25777318944081	0.69	328	2.3738551	18	SEMA3A
**MFAP2.6**	1.222	1.17977050475317	727	371	2.4458131	18	MFAP2
**S100A16.9**	1.914	1.10319610974824	876	508	3.8292424	18	S100A16
**CD36.2**	1.402	0.68926097419472	529	0.14	2.8057307	18	CD36
**CD9.9**	2.699(	1.11626307400126	946	659	5.3980691!	18	CD9
**CEACAM1.1**	3.452	0.277141744884564	533	211	6.9055976	18	CEACAM1
**TSPAN11.4**	1.103	0.428946097298007	756	452	2.2072132	18	TSPAN11
**CALU.6**	1.742	0.914556258554627	909	619	3.4852795	18	CALU
**MYCT1.8**	4.091	0.992496326203869	764	475	8.1830187	18	MYCT1
**CALD1.7**	2.342(	0.57837604909195	988	677	4.6841683	18	CALD1
**CTHRC1.3**	3.884	0.86031910611605	851	0.56	7.7684628!	18	CTHRC1

**PLXNA2.6**	4.5730	0.605574810052037	752	0.44	9.1460539	18	PLXNA2
**CRIM1.5**	7.7439	0.666363832304336	913	643	1.5487938	18	CRIM1
**PLOD2.5**	8.4836	0.507193326421437	798	489	1.6967356	18	PLOD2
**GGT5.5**	1.2231	0.744228711623326	855	586	2.4463901	18	GGT5
**LMO2.3**	1.3548	1.2395559578499	756	0.41	2.7097106	18	LMO2
**FRMD6.5**	3.0255	0.917875953570549	0.76	389	6.0511569	18	FRMD6
**LINC00636**	1.1427	0.491331768987971	504	135	2.2854481	18	LINC00636
**F2RL3**	6.0162	0.579811185890445	0.62	296	1.2032582	18	F2RL3
**ALDH1A1**	9.1160	0.960441051231276	678	428	1.8232037	18	ALDH1A1
**FSCN1.3**	1.1400	1.25993975160277	777	521	2.2800544	18	FSCN1
**C1S.3**	1.6422	0.502964714513583	884	628	3.2844430	18	C1S
**MAP1B.8**	2.8543	0.780782540610901	851	524	5.7086453	18	MAP1B
**ADIRF.11**	3.6970	1.21169627759948	942	661	7.3941320	18	ADIRF
**RAB30.3**	7.4025	-0.282878243472813	0.14	493	1.4805120	18	RAB30
**C1QB.5**	1.5530	-0.419952374275259	153	492	3.1060040	18	C1QB
**CD52.5**	2.9073	-1.51916868313323	194	587	5.8146239	18	CD52
**KCTD12.6**	6.4526	0.683297374573617	855	592	1.2905306	18	KCTD12
**CAV1.8**	7.3958	1.12522942130468	905	649	1.4791674	18	CAV1
**COL6A2.4**	1.1967	0.489309093038473	921	647	2.3935592	18	COL6A2
**ELN**	2.0164	1.1186888757249	711	424	4.0328295	18	ELN
**TNFSF10.4**	2.4280	0.958605828526585	855	552	4.8560318	18	TNFSF10
**GRN.4**	3.3351	0.869572049603921	909	617	6.6703498	18	GRN
**DYSF.1**	4.1098	0.868056203127054	0.69	424	8.2197429	18	DYSF
**EMID1.1**	4.2998	0.543407850343782	665	379	8.5996234	18	EMID1
**ABI3BP.6**	5.1923	1.0760768863612	698	383	1.0384697	18	ABI3BP
**BMX.1**	6.4902	0.473264839272383	0.62	254	1.2980501	18	BMX
**ANPEP.8**	3.6733	0.284373066962742	727	376	7.3466754	18	ANPEP
**CARHSP1.6**	5.8075	0.890052363207327	806	0.53	1.1615013	18	CARHSP1
**JAG1.5**	6.7909	0.646077360922845	822	553	1.3581881	18	JAG1
**TSPAN15**	1.1536	0.790357474946119	0.64	303	2.3073250	18	TSPAN15
**WWTR1.5**	5.6054	0.75281941032535	847	586	1.1210852	18	WWTR1
**ZNF385D.6**	1.6777	0.377083542944498	798	498	3.3554803	18	ZNF385D

**PLCE1.1**	2.3432	0.316753824599696	0.62	0.34	4.6865015	18	PLCE1
**MTUS1.6**	2.9858	0.885922080528254	798	506	5.9717827	18	MTUS1
**CD2.4**	5.2407	-1.4020288597456	318	606	1.0481419	18	CD2
**CD34.6**	2.5472	0.923297993297514	785	509	5.0945267	18	CD34
**IGFBP4.8**	1.6897	0.756280149871716	975	724	3.3794292	18	IGFBP4
**SNCG.4**	3.0503	1.62225841395287	715	458	6.1007548	18	SNCG
**CFI.6**	4.2858	0.723410815561347	715	427	8.5716242	18	CFI
**C3.5**	6.0052	0.261027401273513	694	415	1.2010470	18	C3
**SFRP1.7**	7.6919	0.327543190553746	707	427	1.5383875	18	SFRP1
**CD82.2**	3.7419	0.431316389839857	756	472	7.4839695	18	CD82
**MIR4435-2H**	4.1675	0.373687331996901	826	546	8.3350510	18	MIR4435-2H
**DST.8**	5.7821	0.681120844750042	864	528	1.1564330	18	DST
**TRBC2.3**	8.4520	-1.4633589267025	0.24	576	1.6904156	18	TRBC2
**IL34.5**	4.5688	0.269671846193234	694	0.44	9.1376870	18	IL34
**SNCA.1**	8.8644	0.825456606765601	645	366	1.7728898	18	SNCA
**ZNF503.5**	1.6780	0.265200988763611	723	454	3.3561463	18	ZNF503
**KRT8.2**	1.7025	0.265055146938675	566	251	3.4050991	18	KRT8
**ARL15.1**	6.6675	0.265882181858102	599	0.33	1.3335171	18	ARL 15
**SVIL.5**	8.2435	0.681855219874744	814	561	1.6487133	18	SVIL
**TYROBP.4**	2.2514	-0.900154617039957	99	421	4.5029153	18	TYROBP
**FKBP10.3**	1.2686	0.482824068983882	744	489	2.5372012	18	FKBP10
**TPSB2.4**	1.6381	-1.71520112165809	153	493	3.2762585	18	TPSB2
**CLTB.4**	2.7489	0.565074958829704	872	557	5.4978769	18	CLTB
**LAMC2.3**	3.1661	0.300108149456639	616	364	6.3322439	18	LAMC2
**ID1.3**	3.1894	0.738678161170218	851	572	6.3788935	18	ID1
**RGS1.4**	4.0986	-1.72126169554762	277	572	8.1973384	18	RGS1
**COLEC12**	4.4811	1.07392226230512	612	358	8.9623734	18	COLEC12
**PDLIM3.1**	7.3082	0.503843094919534	616	365	1.4616498	18	PDLIM3
**IL1RN.3**	1.5239	-0.361563487077716	521	213	3.0478401	18	IL1RN
**SEPP1.4**	2.1512	0.60944024687988	744	493	4.3025254	18	SEPP1
**THY1.6**	2.8248	0.370619072571251	773	492	5.6496477	18	THY1
**S100A13.7**	8.3615	0.698840617337322	802	541	1.6723021	18	S100A13

**EPHA2.4**	1.2047	0.424090877178239	674	421	2.40943999	18	EPHA2
**TBX1.2**	4.8058	1.13198153000608	624	356	9.61160525	18	TBX1
**CEBPD.9**	3.2722	0.44619604754788	888	624	6.54441029	18	CEBPD
**GZMB.5**	3.3723	-0.695613852818458	145	0.49	6.74474065	18	GZMB
**FABP4.2**	5.4369	2.86028123354641	537	225	1.08738301	18	FABP4
**FBLN2.8**	5.5550	0.555066100933038	0.81	527	1.11100121	18	FBLN2
**STMN1.2**	6.6543	0.896306041149086	657	403	1.33086932	18	STMN1
**IFI6.4**	6.8512	0.422790951273104	764	451	1.37024038	18	IFI6
**CPVL.6**	4.9267	-0.423168711754537	161	415	9.85347567	18	CPVL
**TRAC.1**	7.2437	-1.28125429348415	236	506	1.44874089	18	TRAC
**CTSD.5**	8.7439	0.425000064846321	0.81	537	1.74879985	18	CTSD
**HPGD.5**	9.1377	-0.378887097732976	574	315	1.82755748	18	HPGD
**PTX3.2**	5.2951	0.797515175044787	0.55	256	1.05903889	18	PTX3
**ITGB2.5**	3.9304	-0.722846442406497	178	496	7.86089324	18	ITGB2
**DAPP1.2**	1.2362	-0.321663227522459	153	463	2.47245468	18	DAPP1
**PIEZO2**	1.5020	0.932758217539008	529	189	3.00416718	18	PIEZO2
**CGNL1**	2.5717	0.500387941996545	533	266	5.14350559	18	CGNL1
**MXRA5.2**	1.5466	0.318224399670967	773	501	3.09338919	18	MXRA5
**GNLY.2**	1.7459	-1.58167731555682	178	436	3.49187204	18	GNLY
**CTLA4.6**	1.9958	-0.42001257177139	157	468	3.99178452	18	CTLA4
**MMP9.7**	9.3718	-0.280964329226633	136	407	1.87437985	18	MMP9
**MEG3.6**	2.5329	0.377150067253769	0.69	0.41	5.06599890	18	MEG3
**RGS5.5**	9.0188	-0.845136406687111	0.14	393	1.80377620	18	RGS5
**GADD45A.1**	1.3599	-0.346899437221439	401	654	2.71998682	18	GADD45A
**HIST1H1D.1**	8.9971	-0.288344826727249	95	381	1.79942357	18	HIST1H1D
**PRF1.3**	1.4685	-0.31446313720366	87	386	2.93704479	18	PRF1
**IGKV4-1.5**	2.2109	-0.365270642277015	0.14	0.39	0.00044219	18	IGKV4-1
**TAGLN.5**	5.6839	1.92101277360258	957	452	1.13678737	19	TAGLN
**ACTA2.4**	4.1711	1.88992522961542	926	0.38	8.34226358	19	ACTA2
**NR2F2.7**	4.7639	1.52143414376136	989	535	9.52780101	19	NR2F2
**MAP1B.9**	8.7900	1.41109261542943	989	526	1.75800113	19	MAP1B
**TPM2.6**	6.3168	1.64444528015144	957	416	1.26337284	19	TPM2

**MYL9.7**	3.2054	1.69692917566943	947	574	6.4109560	19	MYL9
**ADAMTS4.7**	6.4056	1.08491730111371	936	0.55	1.2811244	19	ADAMTS4
**ADIRF.12**	7.8796	1.53689861738582	1	663	1.5759364	19	ADIRF
**C11orf96.5**	1.2979	1.22716183017133	1	665	2.5958867	19	C11orf96
**A2M.8**	2.5257	1.55519589331	1	656	5.0514541	19	A2M
**ITGA7.2**	3.5937	0.885980992960014	0.84	377	7.1874585	19	ITGA7
**SYNPO2.5**	6.2855	1.19131754577197	851	406	1.2571085	19	SYNPO2
**TINAGL1.7**	6.3448	1.14901191183441	947	563	1.2689705	19	TINAGL1
**ADAMTS1.5**	1.5706	1.37388515710233	979	685	3.1412492	19	ADAMTS1
**MYLK.4**	5.0782	1.15882228640345	957	547	1.0156579	19	MYLK
**TIMP3.6**	1.0611	1.23737747089135	1	661	2.1223978	19	TIMP3
**EPAS1.7**	6.2069	1.28552678128651	1	714	1.2413940	19	EPAS1
**TPM1.9**	9.0992	1.50586382058211	947	504	1.8198457	19	TPM1
**PODXL.4**	2.4151	1.05760081889933	968	623	4.8302243	19	PODXL
**PDGFA.2**	3.8342	0.992866589991446	883	449	7.6685535	19	PDGFA
**NOTCH3.5**	6.0054	1.22336902663543	862	382	1.2010960	19	NOTCH3
**SMOC2.5**	6.3115	1.18904029820236	0.84	346	1.2623119	19	SMOC2
**RGS5.6**	7.4473	2.07359306079088	862	388	1.4894679	19	RGS5
**ID3.3**	1.0426	1.54025619604168	979	669	2.0853766	19	ID3
**SLC7A2.6**	1.4868	0.954905491134626	819	264	2.9737946	19	SLC7A2
**SPARCL1.7**	1.9531	1.42099348010581	989	644	3.9062252	19	SPARCL1
**CPE.6**	1.9945	1.06352611761715	926	521	3.9891913	19	CPE
**MCAM.6**	2.3815	1.44316628114873	915	598	4.7631716	19	MCAM
**ADGRF5.6**	3.0175	1.04356225950203	947	578	6.0351756	19	ADGRF5
**MSX1.2**	4.5324	0.965590782452884	926	557	9.0649466	19	MSX1
**DYSF.2**	1.4404	0.353006492821692	872	425	2.8809403	19	DYSF
**RAMP2.9**	1.8160	1.09509427748601	979	483	3.6320194	19	RAMP2
**CNN1.2**	2.2690	0.459811853384394	0.67	228	4.5381693	19	CNN1
**EDNRA.4**	4.1019	0.918314283028537	787	213	8.2039943	19	EDNRA
**ID4.6**	5.5270	1.08801526639167	915	449	1.1054131	19	ID4
**RGS16.3**	5.6231	1.20060462683381	979	665	1.1246236	19	RGS16
**CCL8.1**	1.2147	0.355380697700863	0.84	452	2.4294689	19	CCL8

**IGFBP7.10**	2.1670	1.26271929577805	989	732	4.33412056	19	IGFBP7
**RHOJ.6**	2.5873	0.454886434666141	947	554	5.17479334	19	RHOJ
**CD93.4**	3.5596	0.911352114807313	989	669	7.11926947	19	CD93
**HSPG2.5**	5.3863	1.00870161906147	1	688	1.07726624	19	HSPG2
**CALD1.8**	6.2877	1.15829715331311	989	679	1.25754091	19	CALD1
**PRKCDBP.5**	9.6999	1.13251512729801	926	502	1.93999020	19	PRKCDBP
**JAG1.6**	1.3927	0.988144344887994	926	554	2.78552048	19	JAG1
**AQP1.8**	1.8978	1.06585023980325	1	684	3.79569880	19	AQP1
**SLIT3.11**	2.1041	0.670763192526033	904	428	4.20835570	19	SLIT3
**APOLD1.5**	2.1427	0.899341829365915	926	641	4.28543381	19	APOLD1
**NUAK1.7**	7.1574	0.660663130962308	894	463	1.43148795	19	NUAK1
**CRIP2.5**	1.2553	0.946534459673392	968	607	2.51077205	19	CRIP2
**PLVAP.8**	1.6652	1.01232948202718	947	579	3.33040322	19	PLVAP
**FRZB.8**	3.9066	1.08235921362376	851	405	7.81320410	19	FRZB
**LIMS2.3**	5.5978	0.711889401000773	894	536	1.11957309	19	LIMS2
**PDGFRB.9**	6.2101	0.753887659628524	936	0.56	1.24203360	19	PDGFRB
**MRVI1.2**	1.0827	0.830054596938712	798	383	2.16552130	19	MRVI1
**SOD3.8**	1.4341	0.840815086293226	947	529	2.86823475	19	SOD3
**COL4A2.4**	2.3738	0.89259150991494	1	708	4.74770910	19	COL4A2
**BCAM.8**	2.4128	1.08497319933658	904	519	4.82567974	19	BCAM
**ENG.8**	3.2507	0.852887400543652	979	601	6.50145265	19	ENG
**CAV1.9**	5.6195	0.895951932298457	1	0.65	1.12391295	19	CAV1
**RAMP3.8**	5.6537	0.901367575018528	926	0.49	1.13075933	19	RAMP3
**NPDC1.8**	7.1436	1.00257490962574	957	559	1.42872434	19	NPDC1
**BMPR2.4**	8.6634	0.932964789281745	947	619	1.73268930	19	BMPR2
**IL6.2**	9.1059	0.832233460243543	947	667	1.82119708	19	IL6
**ADGRG1.4**	9.4161	0.40039103527323	936	562	1.88323133	19	ADGRG1
**STEAP4.7**	1.1686	1.12118613299871	819	409	2.33735071	19	STEAP4
**HES1.5**	1.1981	1.1488390154519	968	659	2.39624494	19	HES1
**MGP.4**	1.2768	0.69749026605884	979	588	2.55367730	19	MGP
**COL15A1.5**	1.4105	0.779640123198119	957	665	2.82102414	19	COL15A1
**MT1M.8**	1.4146	0.777389807702615	0.84	0.4	2.82935148	19	MT1M

**MYC.3**	1.9245	0.777135377897094	904	0.48	3.8490968	19	MYC
**FAM167B.8**	2.7110	0.443860750806811	851	465	5.4220086	19	FAM167B
**CRISPLD2.7**	2.8844	0.794635490828279	926	0.57	5.7689276	19	CRISPLD2
**CYP1B1.4**	5.4138	0.488332145203346	936	563	1.0827757	19	CYP1B1
**PALMD.7**	5.6304	0.754072577594026	936	547	1.1260968	19	PALMD
**RCAN2.5**	5.9059	0.980469979625643	809	351	1.1811913	19	RCAN2
**PECAM1.9**	7.6655	0.919288749248501	979	673	1.5331066	19	PECAM1
**TGM2.4**	1.0605	0.477813031283228	957	612	2.1211390	19	TGM2
**CCDC3.7**	2.4810	0.892867922134868	872	466	4.9621720	19	CCDC3
**NDUFA4L2.5**	2.5823	1.5543702123475	755	235	5.1647011	19	NDUFA4L2
**COL14A1.7**	2.8728	0.454694410696833	947	643	5.7456976	19	COL14A1
**FLT1.8**	4.8260	0.768319670617832	904	495	9.6521818	19	FLT1
**NEURL1B.2**	4.8680	0.744757954826769	862	511	9.7360421	19	NEURL1B
**HEG1.6**	7.1610	0.881647863253141	936	612	1.4322146	19	HEG1
**CRIM1.6**	1.4011	0.823385925441564	936	645	2.8022955	19	CRIM1
**GNG11.7**	1.4640	0.824718100731519	979	581	2.9281607	19	GNG11
**VWF.8**	2.0238	0.920493550628006	904	579	4.0477851	19	VWF
**NET1.4**	2.1547	1.00334999231507	0.83	434	4.3095986	19	NET1
**SLCO2A1.5**	2.6857	0.670201076135841	915	627	5.3714379	19	SLCO2A1
**CDH5.6**	8.1588	0.633204604160887	926	609	1.6317744	19	CDH5
**ADAMTS9.4**	1.0016	0.545238623913158	904	627	2.0033494	19	ADAMTS9
**FLNB.2**	1.2794	0.539122422861125	947	586	2.5589722	19	FLNB
**RNASE1.7**	1.7991	0.852812115640192	904	576	3.5982137	19	RNASE1
**MMRN2.9**	2.5012	0.810126128737778	872	443	5.0025756	19	MMRN2
**ESAM.6**	2.6707	0.899011821743735	862	529	5.3415646	19	ESAM
**GJA1.6**	2.7013	0.415795271548417	915	472	5.4027917	19	GJA1
**MYH11.4**	3.2176	2.05276955756337	755	332	6.4353342	19	MYH11
**ROBO4.9**	1.3900	0.430390560131434	851	0.44	2.7800068	19	ROBO4
**TM4SF1.8**	1.4291	0.823626617902725	936	618	2.8582885	19	TM4SF1
**COL4A1.3**	1.7590	0.900724447461395	979	714	3.5180287	19	COL4A1
**PLAC9.5**	1.8370	0.714287086049732	851	498	3.6741501	19	PLAC9
**ID1.4**	4.2651	1.14239270908601	904	573	8.5303865	19	ID1

**NFIB.5**	4.6891	0.801893693139711	915	598	9.3782376	19	NFIB
**RAMP1.2**	6.1780	0.767373295381264	713	274	1.2356172	19	RAMP1
**RHOB.7**	6.1968	0.79103768589572	936	626	1.2393673	19	RHOB
**TEK.9**	9.0559	0.481964915641202	819	443	1.8111838	19	TEK
**PDGFB.5**	9.2114	0.520473475462249	819	521	1.8422832	19	PDGFB
**NES.9**	1.1996	0.795679326809478	809	365	2.3993872	19	NES
**EGFL7.12**	1.4762	0.808151749724021	851	429	2.9525872	19	EGFL7
**FILIP1L.8**	2.2529	0.929604377024926	904	592	4.5058513	19	FILIP1L
**SELE.5**	2.5941	0.891435837418237	894	642	5.1883037	19	SELE
**TGFBR3.3**	3.3479	0.594937557665354	883	575	6.6958980	19	TGFBR3
**ACKR1.6**	3.3900	0.976901872539584	883	554	6.7801528	19	ACKR1
**EGR3.2**	3.5267	0.444272359275507	947	665	7.0535610	19	EGR3
**IFI27.6**	5.0004	0.890788099888641	968	602	1.0000871	19	IFI27
**AVPR1A.2**	5.6525	0.394288475099552	553	161	1.1305068	19	AVPR1A
**SORBS2.4**	6.9568	0.872267943069556	777	391	1.3913620	19	SORBS2
**ZNF503.6**	7.0205	0.295164121962456	862	455	1.4041062	19	ZNF503
**KRT18.2**	8.5980	0.403814056414123	809	447	1.7196107	19	KRT18
**ACKR3.5**	8.8586	0.355941780387405	872	611	1.7717393	19	ACKR3
**CLDN5.11**	9.7435	0.847201332423659	862	443	1.9487038	19	CLDN5
**KCTD12.7**	1.1058	0.706251129608844	894	594	2.2116436	19	KCTD12
**CLEC14A.9**	1.3745	0.610536342192006	872	0.53	2.7490977	19	CLEC14A
**TSC22D1.4**	1.4488	0.755109407539217	957	659	2.8976368	19	TSC22D1
**CD34.7**	1.8322	0.765584906041367	851	511	3.6645974	19	CD34
**WWTR1.6**	2.2571	0.58136438276659	926	588	4.5142894	19	WWTR1
**MPZL2.11**	2.5165	0.410982287907507	819	434	5.0331617	19	MPZL2
**GRB10.6**	2.5670	0.371344630631106	819	497	5.1341153	19	GRB10
**PCDH17.10**	2.9458	0.807453678207588	851	0.55	5.8916628	19	PCDH17
**NUPR1.7**	2.9536	0.480190008655877	851	404	5.9073934	19	NUPR1
**PRRX1.9**	4.6704	0.485330271315078	915	516	9.3409493	19	PRRX1
**ADAP2.3**	5.2074	0.348438036901585	702	364	1.0414823	19	ADAP2
**SPNS2.6**	6.4719	0.735967811478278	809	486	1.2943918	19	SPNS2
**INSR.5**	6.8678	0.694340945051326	872	569	1.3735616	19	INSR

**ARHGAP29.7**	7.2732	0.644662071033232	872	529	1.4546536	19	ARHGAP29
**HSPA1A.4**	8.5260	0.697708001908982	989	667	1.7052084	19	HSPA1A
**CRABP1.10**	8.5600	-0.474206408558253	32	316	1.7120022	19	CRABP1
**GSN.7**	9.3201	0.595857331421394	979	694	1.8640248	19	GSN
**ZNF385D.7**	1.0799	0.731686081995108	0.83	0.5	2.1598481	19	ZNF385D
**MCTP1.6**	1.1061	0.614662306013595	851	557	2.2123729	19	MCTP1
**ADGRL4.8**	1.1658	0.747037146785636	872	591	2.3317473	19	ADGRL4
**THBD.6**	1.4887	0.795978697985085	872	552	2.9774023	19	THBD
**NGFR**	1.5341	0.256552799266035	713	414	3.0683769	19	NGFR
**ADAM15.7**	1.7158	0.759559787583795	862	531	3.4316927	19	ADAM15
**KCNE4.2**	1.7572	1.0193076010408	691	209	3.5144577	19	KCNE4
**GRASP.7**	2.3540	0.643876760532365	894	574	4.7081600	19	GRASP
**PPP1R14A.3**	2.8637	1.13045586902856	734	352	5.7274140	19	PPP1R14A
**BGN.7**	2.9614	0.480247293414157	904	614	5.9229147	19	BGN
**ECSCR.1.9**	3.0594	0.524403871131737	894	573	6.1189832	19	ECSCR.1
**SNCG.5**	4.5106	0.727060769031957	809	459	9.0213964	19	SNCG
**TSPAN7.5**	6.3735	0.561156584731796	0.83	469	1.2747067	19	TSPAN7
**MFGE8.3**	9.0131	0.937263938026337	809	491	1.8026209	19	MFGE8
**EMCN.9**	1.0200	0.678115777025454	851	524	2.0400318	19	EMCN
**EGFLAM.1**	1.0952	0.325694737703166	606	147	2.1904177	19	EGFLAM
**ITGA6.10**	1.3975	0.663964238619229	915	0.62	2.7950794	19	ITGA6
**MT1A.3**	2.0957	0.494120847011106	691	379	4.1915795	19	MT1A
**TBX2.5**	2.7088	0.769548975600474	723	443	5.4177465	19	TBX2
**CEBPD.10**	3.0742	0.509564937177284	979	625	6.1485140	19	CEBPD
**IGFBP4.9**	3.2351	0.498806670184727	1	725	6.4703302	19	IGFBP4
**LBH.3**	3.3391	0.62569593049759	872	516	6.6782657	19	LBH
**SSTR2.1**	3.8241	0.402883006603969	713	401	7.6483986	19	SSTR2
**SERPINH1.4**	3.9064	0.388756843257175	989	0.71	7.8128260	19	SERPINH1
**DUSP23.4**	4.0519	0.587037282823516	851	481	8.1039459	19	DUSP23
**DST.9**	4.9051	0.435935695309598	915	0.53	9.8103440	19	DST
**CCL2.4**	5.7442	0.823701598394652	894	641	1.1488433	19	CCL2
**PHLDA1.3**	6.1214	0.527569527603811	947	662	1.2242933	19	PHLDA1

**LMOD1.2**	6.6641	0.786877029419807	649	175	1.3328265	19	LMOD1
**SOX18.6**	1.0277	0.765130278784833	819	456	2.0554353	19	SOX18
**ELOVL7.8**	1.0721	0.55312337342325	755	336	2.1442511	19	ELOVL7
**MAST4.2**	1.0961	0.612620135260877	0.84	508	2.1922274	19	MAST4
**EMP2.6**	1.3820	0.673102842004218	872	526	2.7641421	19	EMP2
**PDE5A.1**	1.4947	0.687377609358356	713	259	2.9894028	19	PDE5A
**HYAL2.7**	1.6593	0.782320240611013	0.84	501	3.3187546	19	HYAL2
**MT2A.7**	2.5935	0.493707649042373	957	655	5.1871897	19	MT2A
**CD200.9**	2.8763	0.588161202786109	809	525	5.7526678	19	CD200
**RASD1.3**	2.9230	0.364857290350855	819	529	5.8461482	19	RASD1
**PGF.3**	2.9764	0.635103591909028	777	494	5.9528079	19	PGF
**PPP1R12B.2**	3.7304	0.98412427692535	723	383	7.4608823	19	PPP1R12B
**PLCB4.2**	4.1140	0.397179269974437	745	0.41	8.2280293	19	PLCB4
**TPPP3.1**	4.3488	0.430044490965869	766	424	8.6977582	19	TPPP3
**C2orf40.2**	4.4102	0.626883792584523	713	321	8.8205489	19	C2orf40
**RAI14.6**	7.3563	0.375330263934012	904	621	1.4712787	19	RAI14
**PLXNA2.7**	7.6686	0.353928904571193	798	442	1.5337227	19	PLXNA2
**PTPRB.9**	1.0660	0.586385015979589	809	539	2.1321305	19	PTPRB
**SERPING1.6**	1.2853	0.32614070636376	936	629	2.5706380	19	SERPING1
**GUCY1A3.3**	1.3172	0.836888742410775	734	433	2.6345646	19	GUCY1A3
**TFPI.3**	1.3848	0.328359737966141	862	0.61	2.7696486	19	TFPI
**FAM13C.6**	1.4712	0.6301064540764	777	368	2.9425865	19	FAM13C
**RCAN1.5**	2.1542	0.46632315417453	0.83	528	4.3084343	19	RCAN1
**SPRY1.6**	2.3448	0.749852846925565	851	594	4.6897414	19	SPRY1
**NRARP**	2.8520	0.467243771269806	766	489	5.7041732	19	NRARP
**CSRP1.2**	3.5658	0.766026233367859	766	428	7.1317933	19	CSRP1
**CPM.3**	4.0962	0.47269606301132	777	517	8.1925123	19	CPM
**PDLIM1.8**	5.0965	0.555819925006447	915	634	1.0193005	19	PDLIM1
**ADRA2A.5**	5.2071	0.757183077435849	691	314	1.0414244	19	ADRA2A
**CYYR1.5**	1.1072	0.592659639709382	766	417	2.2145163	19	CYYR1
**ENAH.5**	1.5133	0.465694323546246	798	472	3.0267330	19	ENAH
**LMO2.4**	1.5995	0.561103778374834	787	412	3.1991084	19	LMO2

**CCL21.5**	1.7562	-1.21250344572288	787	455	3.5124665		19	CCL21
**PPFIBP1.8**	2.2322	0.433330001810516	0.83	465	4.4644092		19	PPFIBP1
**ITGBL1.7**	2.3283	-0.287366376854763	755	352	4.6567375		19	ITGBL1
**THSD7A.10**	2.3990	0.605527919135364	755	415	4.7981876		19	THSD7A
**SOX4**	2.5245	0.715885165896919	851	583	5.0490383		19	SOX4
**ABCC9.1**	3.3022	0.54769505212553	553	77	6.6044724		19	ABCC9
**PLCE1.2**	4.5727	0.65951739074999	691	341	9.1454989		19	PLCE1
**ERG.6**	4.7225	0.566254272656403	745	0.44	9.4451376		19	ERG
**CHN1.3**	4.7858	0.492251286284194	0.84	0.59	9.5716379		19	CHN1
**PIK3R3.4**	5.3544	0.447815622563803	787	498	1.0708927		19	PIK3R3
**HES4.1**	5.5656	0.73684494890135	777	513	1.1131392		19	HES4
**CSRP2.4**	7.2057	0.68297444275988	766	413	1.4411504		19	CSRP2
**ITGB4.9**	1.0094	0.423444853395906	755	432	2.0189254		19	ITGB4
**CXorf36.8**	1.6763	0.558932696991243	734	457	3.3526476		19	CXorf36
**PREX2.5**	2.7673	0.456676871016201	745	428	5.5347996		19	PREX2
**CD9.10**	4.6076	0.457003347817691	947	661	9.2153801		19	CD9
**IGKV4-1.6**	4.9339	-0.310458440135244	0.66	386	9.8679079		19	IGKV4-1
**NOSTRIN.8**	6.4961	0.479118839400478	734	476	1.2992299		19	NOSTRIN
**FZD4.5**	1.3518	0.417316755882879	723	387	2.7037608		19	FZD4
**CCL19**	2.3714	0.656368770427956	894	627	4.7429741		19	CCL19
**FAM162B.1**	2.6810	0.642299410999319	596	205	5.3620675		19	FAM162B
**CNKSR3.6**	3.0075	0.557573627081187	755	492	6.0151883		19	CNKSR3
**HSPB1.9**	3.6855	0.39688464684923	947	615	7.3711487		19	HSPB1
**HOPX.3**	3.9697	0.250823189300859	723	329	7.9394161		19	HOPX
**CALCRL.6**	4.2046	0.594377698985225	766	465	8.4092086		19	CALCRL
**RBP7.3**	4.7817	0.51122598938807	681	295	9.5634524		19	RBP7
**DES.2**	5.0878	0.409756124347128	617	319	1.0175715		19	DES
**RALGAPA2**	5.7648	0.319870841404694	766	0.48	1.1529618		19	RALGAPA2
**GLUL.5**	6.3000	0.27684792543515	926	608	1.2600194		19	GLUL
**GJC1**	6.7675	0.679040130752213	691	426	1.3535196		19	GJC1
**COX4I2.1**	9.1848	0.665170913054276	0.66	254	1.8369614		19	COX4I2
**MEOX1.6**	1.1061	0.385070841632493	681	376	2.2123211		19	MEOX1

**CXCL3**	1.1613	-0.278570800032528	0.83	571	2.3226957	19	CXCL3
**GPX3.4**	1.9372	0.297527611161584	755	0.48	3.8744361	19	GPX3
**SERPINF1.7**	2.0472	-0.323698747151149	872	0.56	4.0945200	19	SERPINF1
**ITIH5.2**	2.1336	0.382301957922594	702	449	4.2672758	19	ITIH5
**FBXO32.4**	3.4849	0.405308718213593	723	458	6.9698701	19	FBXO32
**C10orf10.7**	4.2469	0.486895358167374	734	412	8.4938650	19	C10orf10
**SLC9A3R2.3**	6.6669	1.01444596070246	723	428	1.3333952	19	SLC9A3R2
**GUCY1B3.1**	9.3423	0.522950510833503	0.66	378	1.8684780	19	GUCY1B3
**VWA1.7**	1.3628	0.481516229844447	734	462	2.7256187	19	VWA1
**SDC2.10**	1.3632	0.328152040075272	0.67	366	2.7264737	19	SDC2
**ANGPT2.3**	1.6608	0.480879528318663	691	386	3.3217508	19	ANGPT2
**MAP3K7CL**	1.6859	0.436124957814869	0.67	406	3.3719662	19	MAP3K7CL
**KCNJ8**	1.6994	0.270650271060674	489	85	3.3989741	19	KCNJ8
**CARMN.1**	1.9043	0.735272779911276	628	307	3.8087783	19	CARMN
**SDPR.4**	1.9649	0.288268297958458	723	445	3.9298935	19	SDPR
**IL33.9**	2.4004	0.447337925309996	702	415	4.8008593	19	IL33
**NTN4.1**	9.3878	0.328401093529905	0.66	325	0.0001877	19	NTN4
**CAMK2N1**	1.0038	0.389515911922485	755	0.45	0.0002007	19	CAMK2N1
**IGFBP2.4**	1.5157	-0.72151499619844	883	0.59	0.0003031	19	IGFBP2
**OLFM1.5**	2.0664	0.364395873991002	691	396	0.0004132	19	OLFM1
**LIMCH1.3**	2.6054	0.416937397217742	691	413	0.0005210	19	LIMCH1
**OGN.6**	2.7860	-0.290322511228973	681	399	0.0005572	19	OGN
**MXRA8.5**	3.0527	-0.321629628827348	734	471	0.0006105	19	MXRA8
**INPP4B.4**	3.5817	0.4699377968389	681	393	0.0007163	19	INPP4B
**GJA4.4**	4.5327	0.598622168242708	638	313	0.0009065	19	GJA4
**FAM89A.1**	4.7804	0.315967894620897	723	468	0.0009560	19	FAM89A
**CARHSP1.7**	5.3315	0.298760687556827	787	532	0.0010663	19	CARHSP1
**RAPGEF5.3**	6.6520	0.444774234661847	0.67	323	0.0013304	19	RAPGEF5
**LIFR.4**	9.4950	0.473743670371849	691	0.44	0.0018990	19	LIFR
**HLA-DRA.4**	1.0768	-0.883975097290232	947	694	0.0021536	19	HLA-DRA
**SLC38A11.1**	1.3399	0.308234140910994	511	142	0.0026798	19	SLC38A11
**IGLV3-21.7**	1.5999	-0.562288173227402	0.67	344	0.0031999	19	IGLV3-21

**FAM110D.1**	2.0204	0.442290751422731	596	221	0.0040409	19	FAM110D
**FILIP1.7**	3.4584	0.728117377927998	649	343	0.0069168	19	FILIP1
**CASQ2.1**	5.4885	0.25173533516575	489	124	0.0109770	19	CASQ2
**RP11-1143G9**	5.6372	-0.29285855230408	777	448	0.0112744	19	RP11-1143G9
**PRF1.4**	5.9462	-0.259027152559832	649	381	0.0118924	19	PRF1
**CRYAB.4**	7.6169	0.353887680638949	606	292	0.0152339	19	CRYAB
**FCN1.3**	7.7620	-0.267624130463167	574	261	0.0155241	19	FCN1
**TPSB2.5**	1.4057	-1.94978143911712	787	488	0.0281140	19	TPSB2
**CXCL1.5**	1.7405	-1.16346592015245	809	512	0.0348100	19	CXCL1
**DNAAF1.2**	2.1667	-0.260195528410426	819	545	0.0433355	19	DNAAF1
**NTRK2.5**	4.8524	0.312415816322545	606	347	0.0970491	19	NTRK2
**SEPT4.1**	5.1095	0.721396099110792	596	327	0.1021902	19	SEPT4
**AOC3.1**	5.1115	0.404489061983225	596	331	0.1022301	19	AOC3
**GUCY1A2.2**	5.1384	0.579283946229174	511	221	0.1027680	19	GUCY1A2
**TIMP1.6**	5.5323	-0.369267049965192	968	711	0.1106479	19	TIMP1
**SOX17.5**	0.0001	0.613570841817568	585	324	0.2450214	19	SOX17
**MPEG1.6**	0.0001	-0.265120130989504	713	0.42	0.2838572	19	MPEG1
**OMD.9**	0.0002	-0.383098995902068	0.67	417	0.4869678	19	OMD
**TNF.3**	0.0002	-0.360339958195616	0.84	538	0.5334992	19	TNF
**MIR4435-2H**	0.0003	-0.258771577144171	0.83	548	0.6128041	19	MIR4435-2H
**RP11-394O4.**	0.0005	0.529628725218437	447	0.12	1	19	RP11-394O4.
**HEY1.1**	0.0017	0.271741814037443	628	377	1	19	HEY1
**SDC1.7**	0.0032	-0.346401087443286	734	0.46	1	19	SDC1
**CCDC102B.1**	0.0052	0.491377637716387	532	0.28	1	19	CCDC102B
**ITGB2.6**	0.0060	-0.697634034337254	0.83	491	1	19	ITGB2
**GZMA.5**	0.0076	-1.05737814765689	787	534	1	19	GZMA
**FAM64A**	9.3779	0.692560249755634	857	95	1.8755936	20	FAM64A
**DLGAP5**	5.2987	1.50637103206499	857	86	1.0597507	20	DLGAP5
**HMMR.1**	7.4433	1.84656663959116	1	166	1.4886611	20	HMMR
**UBE2C.2**	4.6476	2.06068360574607	929	102	9.2952499	20	UBE2C
**CENPA.1**	8.5390	0.879704595308288	1	276	1.7078062	20	CENPA
**NEK2**	2.3934	0.695042757604039	786	7	4.7868719	20	NEK2

**CDC20.2**	3.3724	1.37312458817485	1	219	6.7449326	20	CDC20
**KIF20A**	1.2575	0.507557153789307	857	184	2.5150604	20	KIF20A
**HIST1H3B**	1.9765	0.496914006217024	857	221	3.9530386	20	HIST1H3B
**KIFC1**	3.6866	1.10031978634313	1	259	7.3733961	20	KIFC1
**APLN.1**	9.1197	0.733184915056599	1	236	1.8239533	20	APLN
**KIF4A**	2.3764	1.125622551585	929	257	4.7529287	20	KIF4A
**DIAPH3**	4.9366	1.52754214820479	929	208	9.8732547	20	DIAPH3
**HAPLN1.1**	6.6459	0.3731855281574	857	219	1.3291987	20	HAPLN1
**UHRF1**	8.0922	0.631821829778645	857	184	1.6184533	20	UHRF1
**CKAP2L**	9.5317	0.607461284425034	857	247	1.9063539	20	CKAP2L
**CDCA3.1**	1.3643	1.1452281198848	929	226	2.7287876	20	CDCA3
**CDK1.1**	1.5197	1.71124183701197	929	222	3.0394621	20	CDK1
**NRGN.1**	2.6721	1.4394036064225	1	0.39	5.3442063	20	NRGN
**CENPF.2**	4.0574	3.39794774791282	1	474	8.1148215	20	CENPF
**STMN1.3**	4.4329	2.83031391040169	1	405	8.8659579	20	STMN1
**PTTG1.1**	4.8489	2.16424496810935	1	368	9.6978235	20	PTTG1
**TOP2A.1**	6.3407	2.54654742448224	929	346	1.2681588	20	TOP2A
**NUSAP1.1**	6.8814	2.15102382955449	929	353	1.3762840	20	NUSAP1
**MKI67.1**	1.4601	2.58516154936468	929	282	2.9203055	20	MKI67
**TYMS.1**	1.6523	2.03941804311831	929	322	3.3046288	20	TYMS
**CDCA8**	2.1632	1.22779185216123	929	329	4.3265253	20	CDCA8
**AURKB.1**	2.3847	0.823934679029216	857	221	4.7694891	20	AURKB
**SPC25.1**	2.9001	0.666607518058294	714	105	5.8003881	20	SPC25
**HMGB2.3**	3.5511	2.50705345211757	1	486	7.1022211	20	HMGB2
**CDKN3.2**	3.6902	1.83622232948769	929	0.4	7.3805323	20	CDKN3
**COL17A1.4**	4.1700	1.49618987778962	1	326	8.3401771	20	COL17A1
**ESCO2.1**	5.3748	1.03864900724877	857	215	1.0749679	20	ESCO2
**MYBL2.1**	7.9483	0.681990514664806	786	0.22	1.5896654	20	MYBL2
**TGM2.5**	8.5490	1.74215196328209	1	613	1.7098141	20	TGM2
**APLNR.6**	1.3635	1.01340791334575	1	472	2.7270123	20	APLNR
**PRC1.2**	1.3723	2.14610856015109	929	0.35	2.7446350	20	PRC1
**TPX2**	1.8437	2.27668768384965	857	0.17	3.6875963	20	TPX2

**LAMP3.4**	1.8586	0.961011795175591	929	364	3.7172123	20	LAMP3
**ADAM15.8**	2.2509	1.79180607513318	1	532	4.5019120	20	ADAM15
**FGF18**	3.3806	0.30713732062972	857	364	6.7612050	20	FGF18
**SDPR.5**	3.7171	1.32975742177252	1	445	7.4343742	20	SDPR
**GTSE1**	3.7228	1.37858261197703	857	156	7.4457728	20	GTSE1
**CLSPN.1**	5.2181	0.878943524512341	929	389	0.0001043	20	CLSPN
**PRR11**	5.5212	1.42068588340628	857	267	0.0001104	20	PRR11
**SELE.6**	5.7605	1.91649576692298	1	643	0.0001152	20	SELE
**HIST1H2BC**	6.1748	0.417402778895775	929	398	0.0001234	20	HIST1H2BC
**S1PR1.9**	6.1992	0.975122778404951	1	0.53	0.0001239	20	S1PR1
**HYAL2.8**	6.4154	1.72212296433745	1	502	0.0001283	20	HYAL2
**CALCRL.7**	6.4271	1.59336889682613	1	466	0.0001285	20	CALCRL
**ASPM.1**	6.5543	2.15833110895158	857	193	0.0001310	20	ASPM
**MPZL2.12**	6.6800	1.62563048831524	1	435	0.0001336	20	MPZL2
**PRCP.4**	7.5699	1.69306209538631	1	624	0.0001513	20	PRCP
**VWF.9**	7.9195	1.99394008262595	1	0.58	0.0001583	20	VWF
**ADAMTS9.5**	8.0813	1.51340601399902	1	628	0.0001616	20	ADAMTS9
**FAM167B.9**	9.3956	1.18014494935449	929	467	0.0001879	20	FAM167B
**CNKSR3.7**	1.0000	0.753851631759524	1	493	0.0002000	20	CNKSR3
**COL13A1.1**	1.0716	0.468623269727635	857	327	0.0002143	20	COL13A1
**COL15A1.6**	1.1846	1.8129906127083	1	666	0.0002369	20	COL15A1
**RRM2.2**	1.2121	1.4494112356675	786	178	0.0002424	20	RRM2
**RAMP3.9**	1.2527	1.57005793514845	1	491	0.0002505	20	RAMP3
**KIAA0101.3**	1.2736	1.98423528512801	857	315	0.0002547	20	KIAA0101
**KIF20B.1**	1.3215	1.70226095957444	929	407	0.0002643	20	KIF20B
**CASC5.1**	1.5465	1.06016012622936	857	0.34	0.0003093	20	CASC5
**TUBA1C.3**	1.5715	1.79820389876289	1	649	0.0003143	20	TUBA1C
**ANLN**	1.6427	1.09908152670572	786	138	0.0003285	20	ANLN
**ACKR3.6**	1.7928	0.965525887349064	1	612	0.0003585	20	ACKR3
**TK1.1**	1.8349	1.15195317757908	857	0.29	0.0003669	20	TK1
**ADGRL4.9**	1.8358	1.58432883377387	1	592	0.0003671	20	ADGRL4
**RAB3C.1**	1.9777	0.664660353225548	857	356	0.0003955	20	RAB3C

**RNASE1.8**	2.0123	1.61198704905891	1	577	0.0004024	20	RNASE1
**BMPR2.5**	2.5629	1.68507446648722	1	621	0.0005125	20	BMPR2
**FLNB.3**	2.8375	1.05658168427461	1	587	0.0005675	20	FLNB
**DLC1.5**	3.1384	1.54288095859736	1	0.63	0.0006276	20	DLC1
**SPRY4.5**	3.2477	0.501072202833186	929	458	0.0006495	20	SPRY4
**COL4A2.5**	3.2888	1.60927053197025	1	709	0.0006577	20	COL4A2
**HIST1H1D.2**	3.3467	1.25862610482354	857	378	0.0006693	20	HIST1H1D
**ECSCR.1.10**	3.3471	1.46091032663755	1	575	0.0006694	20	ECSCR.1
**PBK**	3.5354	1.44682022930133	786	279	0.0007070	20	PBK
**MGST1.1**	3.5374	0.281205365413873	857	336	0.0007074	20	MGST1
**HHEX.2**	3.7203	0.521665792341314	929	338	0.0007440	20	HHEX
**CLDN11.1**	3.7255	0.80448169557	857	0.35	0.0007451	20	CLDN11
**ICAM4**	4.2009	0.441351904128639	929	422	0.0008401	20	ICAM4
**MAP1B.10**	4.2012	1.39090944468547	1	527	0.0008402	20	MAP1B
**OSR2**	4.3417	-0.256998488508503	0	391	0.0008683	20	OSR2
**OLFM1.6**	4.6103	1.02438701230952	929	397	0.0009220	20	OLFM1
**ITGA6.11**	4.7272	1.54503133500344	1	621	0.0009454	20	ITGA6
**HEG1.7**	4.8144	1.48530641446707	1	613	0.0009628	20	HEG1
**ERG.7**	5.0525	1.02875670750702	929	441	0.0010105	20	ERG
**BUB1.1**	5.3153	0.830647669086309	786	275	0.0010630	20	BUB1
**PRKCDBP.6**	5.5614	1.19220063313191	1	504	0.0011122	20	PRKCDBP
**MCAM.7**	6.1071	1.07771627658873	1	599	0.0012214	20	MCAM
**ROBO4.10**	6.1176	0.940963540783119	929	442	0.0012235	20	ROBO4
**FAM110D.2**	6.5248	0.365545573564441	857	222	0.0013049	20	FAM110D
**LHX6.1**	6.8898	0.688644372502711	857	395	0.0013779	20	LHX6
**INHBB.5**	8.5515	0.533794000750735	929	535	0.0017103	20	INHBB
**NEURL1B.3**	8.7314	0.928357317825539	929	512	0.0017462	20	NEURL1B
**ABL2.4**	9.4996	1.23449208773898	1	0.61	0.0018999	20	ABL2
**TSPAN7.6**	9.6247	0.773001419225563	1	471	0.0019249	20	TSPAN7
**DYSF.3**	1.0384	0.891124736093005	857	427	0.0020769	20	DYSF
**NID1.4**	1.0959	1.03149161092284	1	646	0.0021919	20	NID1
**SLCO2A1.6**	1.1060	0.966986903762004	1	628	0.0022120	20	SLCO2A1

**UPP1.3**	1.2030	1.0695861984023	929	548	0.0024061	20	UPP1
**FAM101B**	1.2319	0.485633561532889	929	499	0.0024638	20	FAM101B
**APOLD1.6**	1.3992	0.844107553096045	1	642	0.0027985	20	APOLD1
**CASC15**	1.5832	0.693778645973597	857	0.36	0.0031664	20	CASC15
**PECAM1.10**	1.6942	1.52346588742038	1	674	0.0033885	20	PECAM1
**LIMS2.4**	1.7612	0.947479705710928	929	537	0.0035224	20	LIMS2
**PCDH17.11**	1.8442	1.86041354785605	929	551	0.0036884	20	PCDH17
**CLEC14A.10**	2.1085	1.64607799076864	929	531	0.0042170	20	CLEC14A
**THBD.7**	2.1918	1.02643315596072	1	553	0.0043837	20	THBD
**NOTCH4.3**	2.2256	0.340184583478999	929	402	0.0044513	20	NOTCH4
**ACOT7.1**	2.3221	0.998594453924741	857	406	0.0046442	20	ACOT7
**CCNB1.2**	2.3435	1.77286996566901	857	331	0.0046870	20	CCNB1
**EPHA2.5**	2.5870	0.671076704706923	857	424	0.0051740	20	EPHA2
**CENPE**	2.6281	1.44647981606061	857	405	0.0052562	20	CENPE
**ENG.9**	2.7173	1.65516254795105	929	603	0.0054347	20	ENG
**SORBS2.5**	2.8404	0.7523194433146	929	393	0.0056809	20	SORBS2
**PTPRB.10**	2.8423	0.896693834664857	929	0.54	0.0056846	20	PTPRB
**FAM107A.9**	3.2565	0.430447351793789	1	468	0.0065131	20	FAM107A
**A2M.9**	3.7497	1.44957045511596	1	657	0.0074995	20	A2M
**BIRC5.2**	3.9903	1.29929669200402	786	237	0.0079807	20	BIRC5
**PDLIM1.9**	4.2284	1.28155063899796	1	635	0.0084568	20	PDLIM1
**NUAK1.8**	4.2605	0.663212062553186	929	465	0.0085211	20	NUAK1
**SOX4.1**	4.8027	1.1883038620768	1	584	0.0096054	20	SOX4
**GNG11.8**	6.0019	1.19777055296413	1	583	0.0120039	20	GNG11
**THBS1.4**	6.0096	0.427676464471563	1	595	0.0120193	20	THBS1
**MAST4.3**	6.0169	0.486155393080311	1	509	0.0120338	20	MAST4
**LYVE1.1**	7.0995	0.412240114932173	786	377	0.0141990	20	LYVE1
**FAM43A**	7.4506	0.380878275198219	929	452	0.0149012	20	FAM43A
**CD93.5**	8.6071	1.54370690472437	929	0.67	0.0172142	20	CD93
**HIST1H1C.1**	9.6150	1.05257386848322	857	436	0.0192300	20	HIST1H1C
**CCNA2**	9.7518	1.55197575622735	786	154	0.0195036	20	CCNA2
**MMRN2.10**	1.0694	0.578249219885479	929	445	0.0213888	20	MMRN2

**EMCN.10**	1.0819	0.735362920301154	1	525	0.0216397	20	EMCN
**ITGA2.4**	1.0869	0.69398310073099	857	381	0.0217382	20	ITGA2
**YOD1.1**	1.1355	0.275608940159602	786	379	0.0227112	20	YOD1
**CLDN5.12**	1.1437	0.286602205142194	1	444	0.0228758	20	CLDN5
**TEK.10**	1.1699	0.564212890156552	857	444	0.0233995	20	TEK
**SOX18.7**	1.1811	0.954548787214122	929	457	0.0236233	20	SOX18
**ESAM.7**	1.1847	0.937487314147049	929	0.53	0.0236947	20	ESAM
**CCNB2.1**	1.1926	1.07823787302747	786	339	0.0238520	20	CCNB2
**NFIB.6**	1.2180	1.12551981695825	929	0.6	0.0243606	20	NFIB
**TM4SF1.9**	1.2316	1.1240780038104	1	619	0.0246336	20	TM4SF1
**CDH5.7**	1.3159	0.873108890947562	929	0.61	0.0263198	20	CDH5
**FSTL1.4**	1.3885	1.05386558891029	1	715	0.0277716	20	FSTL1
**BUB1B**	1.4950	0.663946759986082	714	154	0.0299004	20	BUB1B
**IL3RA.6**	1.6650	0.812523624024219	929	0.58	0.0333000	20	IL3RA
**PLAT.4**	1.6671	-0.388191439579025	71	453	0.0333429	20	PLAT
**MCTP1.7**	1.7499	0.619371303610294	929	558	0.0349982	20	MCTP1
**ELN.1**	2.0377	0.342437907485459	857	427	0.0407555	20	ELN
**DUSP23.5**	2.0981	0.928816512245324	929	482	0.0419629	20	DUSP23
**HIST1H1B.1**	2.1177	0.80119876293363	714	311	0.0423541	20	HIST1H1B
**CPE.7**	2.1186	-0.896819486648044	71	523	0.0423734	20	CPE
**GRASP.8**	2.1271	0.832136162643352	929	575	0.0425437	20	GRASP
**IFI27.7**	2.2381	1.04859830810554	1	603	0.0447638	20	IFI27
**CXCL8.2**	2.4272	-0.664367266560671	929	593	0.0485450	20	CXCL8
**RAPGEF5.4**	2.5985	0.914603831626812	857	324	0.0519705	20	RAPGEF5
**PLK1**	2.6488	1.02999096298283	786	274	0.0529779	20	PLK1
**ICAM1.4**	2.7015	0.97309501647933	1	736	0.0540314	20	ICAM1
**CENPW.1**	2.8341	1.10496553208687	786	0.35	0.0566836	20	CENPW
**GATA2.5**	2.8757	0.485378121598142	857	489	0.0575145	20	GATA2
**SLC6A6.3**	2.9758	0.723317148692182	929	581	0.0595169	20	SLC6A6
**CALU.7**	3.0414	0.84669424134439	1	622	0.0608293	20	CALU
**CYP1B1.5**	3.2224	0.354497055125378	929	564	0.0644498	20	CYP1B1
**VWA1.8**	3.4190	0.899928675332179	857	463	0.0683809	20	VWA1

**RAI14.7**	3.4570	0.85160518775209	929	622	0.0691415(	20	RAI14
**PIK3R3.5**	3.6093	0.591708722643106	857	499	0.0721862	20	PIK3R3
**RCAN1.6**	3.8349	0.457067069556356	929	0.53	0.0766986	20	RCAN1
**FAM111B**	3.8701	0.295047497538301	643	192	0.0774030(	20	FAM111B
**MYCT1.9**	3.8868	0.738525457787858	857	478	0.0777368	20	MYCT1
**NDC80**	3.9667	0.876224719865803	786	281	0.0793358	20	NDC80
**MALL.5**	4.0172	1.12147424586946	857	576	0.0803457	20	MALL
**PLVAP.9**	4.0557	1.58359921955239	857	0.58	0.0811157(	20	PLVAP
**ADGRF5.7**	4.2905	0.335885244995903	929	0.58	0.0858115	20	ADGRF5
**NR2F2.8**	4.3879	0.82303793184106	1	536	0.0877583	20	NR2F2
**GJA1.7**	4.4279	0.398061970979838	929	474	0.0885590	20	GJA1
**GLTP.2**	4.4659	0.614536420110335	929	441	0.0893186(	20	GLTP
**CRIP2.6**	4.6758	0.94170642600401	929	608	0.0935169	20	CRIP2
**SPINK5.2**	4.7757	0.366261952584467	786	232	0.0955148	20	SPINK5
**PCNA.1**	4.8862	1.02025982057308	786	404	0.0977247	20	PCNA
**ELOVL7.9**	4.9951	0.345202600702228	857	338	0.0999025	20	ELOVL7
**AQP1.9**	5.0154	0.876740777222671	1	685	0.1003083	20	AQP1
**LAMC2.4**	5.1206	0.295051372273521	786	366	0.1024123	20	LAMC2
**ACKR1.7**	5.4184	0.608577602280398	929	555	0.1083687	20	ACKR1
**JAG1.7**	5.5189	0.68567613802453	929	556	0.1103791	20	JAG1
**NPDC1.9**	5.6081	1.11037174702227	929	0.56	0.1121633	20	NPDC1
**NOSTRIN.9**	5.6520	0.787840961401449	857	477	0.1130411	20	NOSTRIN
**NREP.3**	5.6537	0.770806973471126	857	494	0.1130742	20	NREP
**CARHSP1.8**	5.9284	0.772729458737365	929	533	0.1185693	20	CARHSP1
**PALMD.8**	5.9411	0.764897634152582	929	548	0.1188230	20	PALMD
**IGFBP7.11**	6.4587	1.074376800361	1	733	0.1291759	20	IGFBP7
**PLXNA2.8**	6.6511	0.901478006089962	857	444	0.1330223	20	PLXNA2
**WWTR1.7**	6.6518	0.847895696353118	929	589	0.1330378	20	WWTR1
**CFI.7**	6.7263	0.630257630600567	857	0.43	0.1345263(	20	CFI
**PREX2.6**	7.0788	0.710263735342025	857	429	0.1415774	20	PREX2
**LCP1.7**	7.3483	-1.25448564846185	143	577	0.1469676	20	LCP1
**STAB1.6**	7.5912	0.34261669094251	857	551	0.1518245	20	STAB1

**ADAMTS4.8**	7.6489	0.454390730192401	929	552	0.1529795	20	ADAMTS4
**CKS2.2**	7.6678	0.807788477408953	857	474	0.1533561	20	CKS2
**PRSS23.4**	8.4252	0.924789504201142	1	733	0.1685047	20	PRSS23
**CRYBG3.7**	8.6846	0.652701502816428	929	553	0.1736939	20	CRYBG3
**PDGFRA.6**	9.2266	-0.838249570411112	214	599	0.1845335	20	PDGFRA
**CXorf36.9**	9.3297	0.370441991943541	857	458	0.1865943	20	CXorf36
**KCTD12.8**	9.6415	0.675343494957517	1	595	0.1928310	20	KCTD12
**CSRP2.5**	9.9476	0.5134279715305	857	414	0.1989522	20	CSRP2
**CRIM1.7**	0.0001	0.705902052729064	1	646	0.2058824	20	CRIM1
**FSCN1.4**	0.0001	0.893673871763598	857	523	0.2142965	20	FSCN1
**FRZB.9**	0.0001	-0.616127313854746	71	407	0.2519881	20	FRZB
**POU2F2.8**	0.0001	-0.695507214798339	71	475	0.2558399	20	POU2F2
**KCNN3.4**	0.0001	0.30144930632628	857	524	0.2703961	20	KCNN3
**BMP2.3**	0.0001	0.284523386973263	857	543	0.2971326	20	BMP2
**PLAU.5**	0.0001	0.547762365590615	857	465	0.2972566	20	PLAU
**S100A16.10**	0.0001	0.733491896401578	929	512	0.3115837	20	S100A16
**DUSP6.4**	0.0001	0.452097737454628	929	0.64	0.3430566	20	DUSP6
**EMP2.7**	0.0001	0.585037981283407	929	527	0.3453006	20	EMP2
**APCDD1.2**	0.0002	-0.402476976422139	1	502	0.4019770	20	APCDD1
**EFNB2.3**	0.0002	0.277971877457662	857	462	0.4152614	20	EFNB2
**NOS3.2**	0.0002	0.359785937621913	786	342	0.4206932	20	NOS3
**GRN.5**	0.0002	0.735236949221454	929	0.62	0.4396164	20	GRN
**IFNG.4**	0.0002	-0.475781342278182	143	409	0.4414646	20	IFNG
**SGK1.4**	0.0002	0.568102142266905	929	653	0.4564747	20	SGK1
**TMEM176A.8**	0.0002	-0.739511266469815	143	0.51	0.4612869	20	TMEM176A
**SERPINH1.5**	0.0002	0.614510056301269	1	711	0.4748922	20	SERPINH1
**RALGAPA2.1**	0.0002	0.353354061470791	857	481	0.5099470	20	RALGAPA2
**EIF4E.1**	0.0002	0.494434399873778	857	485	0.5127075	20	EIF4E
**IQGAP3**	0.0002	0.532801443590992	571	119	0.5464679	20	IQGAP3
**LGALS3BP.5**	0.0002	-0.736491159495046	143	539	0.5643219	20	LGALS3BP
**ZWINT.1**	0.0003	1.1572776404653	714	0.4	0.6089391	20	ZWINT
**OMD.10**	0.0003	-0.413887837595413	71	419	0.6277353	20	OMD

**COL6A2.5**	0.0003	-0.295692440475995	1	0.65	0.6334451	20	COL6A2
**FLT1.9**	0.0003	0.541298520408147	857	497	0.6351157	20	FLT1
**FZD4.6**	0.0003	0.426455290658125	786	388	0.6523625	20	FZD4
**IFIT3**	0.0003	0.381671745567584	786	297	0.6706407	20	IFIT3
**EGFL7.13**	0.0003	0.770990513845803	857	0.43	0.7429922	20	EGFL7
**PIEZO2.1**	0.0003	0.58984168824752	643	193	0.7487113	20	PIEZO2
**BCAM.9**	0.0003	0.471187154410681	929	0.52	0.7702913	20	BCAM
**PLCB1.1**	0.0004	0.369730554552889	786	0.35	0.8794071	20	PLCB1
**CD200.10**	0.0004	0.574570291621683	857	526	0.8952252	20	CD200
**FJX1**	0.0004	0.398431059578498	714	409	0.8971309	20	FJX1
**ISLR.9**	0.0004	-0.41814296575901	71	379	0.9450078	20	ISLR
**CTSB.5**	0.0004	0.601052223920058	1	631	0.9486156	20	CTSB
**SPON2.7**	0.0004	-0.527766482079532	143	434	0.9565830	20	SPON2
**CCDC71L.3**	0.0004	0.334757058153751	857	467	0.9718207	20	CCDC71L
**KIF23.1**	0.0005	1.02500743711418	714	277	1	20	KIF23
**HTRA1.5**	0.0005	0.510563628362607	929	605	1	20	HTRA1
**SMAD1.3**	0.0005	0.620938558547547	786	431	1	20	SMAD1
**EPAS1.8**	0.0005	0.610096670497341	1	715	1	20	EPAS1
**MASP1.9**	0.0005	-0.417415109820535	143	436	1	20	MASP1
**MTUS1.7**	0.0005	0.495474895854545	929	509	1	20	MTUS1
**SERPINF1.8**	0.0006	-1.5304543448491	214	562	1	20	SERPINF1
**CDH13.5**	0.0006	0.769086930048678	786	501	1	20	CDH13
**TROAP.1**	0.0006	0.819768634789516	714	196	1	20	TROAP
**INSR.6**	0.0007	0.274742552189206	857	0.57	1	20	INSR
**NCAPG.1**	0.0007	0.625594657222912	643	125	1	20	NCAPG
**GAS2L3**	0.0007	0.368782823174364	714	285	1	20	GAS2L3
**YPEL2.1**	0.0007	0.291396973309069	857	463	1	20	YPEL2
**HES1.6**	0.0007	0.620296257826824	929	0.66	1	20	HES1
**SFRP2.3**	0.0008	-0.947481500707367	214	525	1	20	SFRP2
**C1QC.6**	0.0008	-0.329604948847645	71	371	1	20	C1QC
**BHLHE41.5**	0.0009	-0.276897011194277	143	439	1	20	BHLHE41
**NRP2.4**	0.0009	0.612022444712348	786	519	1	20	NRP2

**TNFSF10.5**	0.0009	0.415334066603874	1	555	1	20	TNFSF10
**PDLIM4.4**	0.0009	0.437217810971042	786	0.45	1	20	PDLIM4
**TMEM176B.7**	0.0010	-0.89962372974228	143	498	1	20	TMEM176B
**ICAM2.6**	0.0010	0.388980618015067	857	536	1	20	ICAM2
**COTL1**	0.0011	0.55824188730896	929	661	1	20	COTL1
**ATF5.2**	0.0012	0.361880684244486	786	407	1	20	ATF5
**MS4A6A.3**	0.0013	-0.368654743928561	214	465	1	20	MS4A6A
**S100A13.8**	0.0013	0.434087691213612	929	543	1	20	S100A13
**CYYR1.6**	0.0015	0.958015778083395	786	419	1	20	CYYR1
**KLF4.3**	0.0015	0.28475568087481	1	716	1	20	KLF4
**PTN.4**	0.0015	-0.390613225165803	0	333	1	20	PTN
**ABCB1.4**	0.0015	0.430615490959661	786	393	1	20	ABCB1
**ITGA10**	0.0015	0.375142583113678	714	379	1	20	ITGA10
**FAM20A.1**	0.0015	-0.263120513464428	143	395	1	20	FAM20A
**JDP2.4**	0.0016	0.442006349153455	857	555	1	20	JDP2
**HN1.3**	0.0016	1.39024513438199	786	517	1	20	HN1
**DNASE1L3.7**	0.0017	0.431768206167628	786	395	1	20	DNASE1L3
**NCOA7.6**	0.0018	0.359262120991187	929	627	1	20	NCOA7
**QPCT**	0.0027	0.525797868200521	643	337	1	20	QPCT
**C2.4**	0.0028	-0.33502323910991	214	501	1	20	C2
**G0S2.1**	0.0028	-0.276316530987889	786	478	1	20	G0S2
**CEP55.1**	0.0030	1.00963712859807	643	0.15	1	20	CEP55
**GLUL.6**	0.0030	0.270225015949451	1	609	1	20	GLUL
**RAMP1.3**	0.0030	-0.292162349437793	0	277	1	20	RAMP1
**FGFR1.5**	0.0032	0.287048481969257	857	592	1	20	FGFR1
**CCDC80.6**	0.0032	-1.24931203852552	214	478	1	20	CCDC80
**AQP3.3**	0.0033	-0.713253404596018	143	0.41	1	20	AQP3
**PPP1R14A.4**	0.0041	-0.315173062149982	714	354	1	20	PPP1R14A
**ITGB5.3**	0.0043	-0.624059713368153	143	494	1	20	ITGB5
**ADAMTS2.9**	0.0045	-0.436860430117842	214	479	1	20	ADAMTS2
**FBN1.5**	0.0052	-0.328435716593924	929	668	1	20	FBN1
**EDIL3.5**	0.0052	0.289440622400512	857	564	1	20	EDIL3

**NQO1.1**	0.0055	0.509515881031214	714	385	1	20	NQO1
**CD34.8**	0.0055	0.45258076880742	786	512	1	20	CD34
**PPFIBP1.9**	0.0055	0.297290366621251	786	466	1	20	PPFIBP1
**ARHGAP29.8**	0.0057	0.50810088444356	786	531	1	20	ARHGAP29
**SPARCL1.8**	0.0058	-0.290326637301666	1	645	1	20	SPARCL1
**GSTP1.4**	0.0059	0.480364640144235	1	707	1	20	GSTP1
**DEPDC1**	0.0059	0.888362718469829	571	0.07	1	20	DEPDC1
**IGLV1-40.1**	0.0066	-0.379396673323005	571	181	1	20	IGLV1-40
**LRP1.5**	0.0071	-0.833811342877809	286	551	1	20	LRP1
**CAV1.10**	0.0076	0.532230758160191	929	652	1	20	CAV1
**HSPA1B.1**	0.0080	0.252900905040377	1	626	1	20	HSPA1B
**APOD.5**	0.0088	-1.333999923196	786	0.5	1	20	APOD
**GZMB.6**	0.0090	-0.603295451908297	786	486	1	20	GZMB
**TUSC3.2**	0.0091	0.322358744088073	643	277	1	20	TUSC3
**KIF15**	0.0094	0.922794201263028	643	214	1	20	KIF15
**CTSC.3**	0.0096	-1.09035765890257	286	551	1	20	CTSC
